# Build–Couple–Transform: A Paradigm for Lead-like Library Synthesis with Scaffold Diversity

**DOI:** 10.1021/acs.jmedchem.2c00897

**Published:** 2022-08-09

**Authors:** Mélanie Uguen, Gemma Davison, Lukas J. Sprenger, James H. Hunter, Mathew P. Martin, Shannon Turberville, Jessica E. Watt, Bernard T. Golding, Martin E. M. Noble, Hannah L. Stewart, Michael J. Waring

**Affiliations:** † Cancer Research UK Newcastle Drug Discovery Unit, Newcastle University Centre for Cancer, Chemistry, School of Natural and Environmental Sciences, Newcastle University, Bedson Building, Newcastle upon Tyne NE1 7RU, U.K.; ‡ Cancer Research UK Newcastle Drug Discovery Unit, Newcastle University Centre for Cancer, Translational and Clinical Research Institute, Faculty of Medical Sciences, Newcastle University, Paul O’Gorman Building, Newcastle upon Tyne NE2 4HH, U.K.; § Chemistry, School of Natural and Environmental Sciences, Newcastle University, Bedson Building, Newcastle upon Tyne NE1 7RU, U.K.

## Abstract

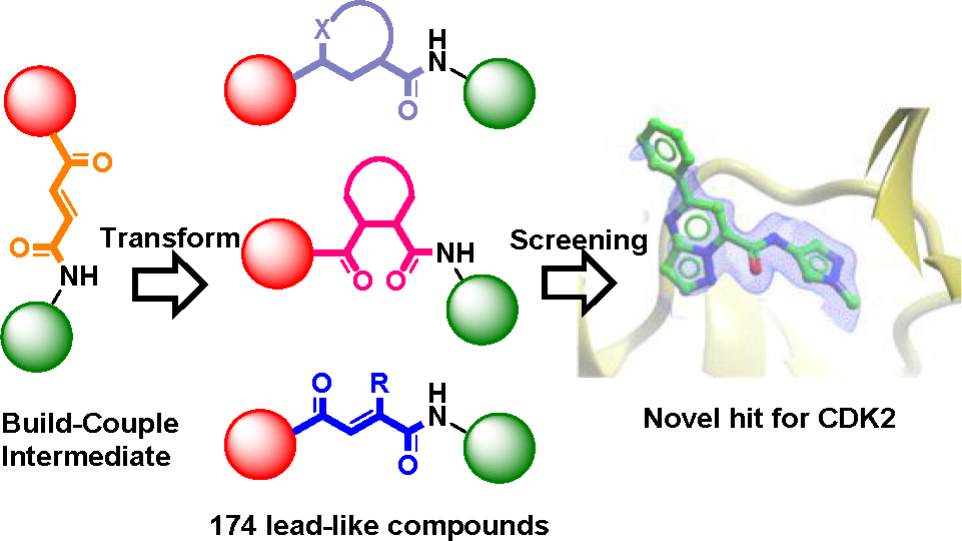

High-throughput screening provides one of the most common ways of finding hit compounds. Lead-like libraries, in particular, provide hits with compatible functional groups and vectors for structural elaboration and physical properties suitable for optimization. Library synthesis approaches can lead to a lack of chemical diversity because they employ parallel derivatization of common building blocks using single reaction types. We address this problem through a “build–couple–transform” paradigm for the generation of lead-like libraries with scaffold diversity. Nineteen transformations of a 4-oxo-2-butenamide scaffold template were optimized, including 1,4-cyclizations, 3,4-cyclizations, reductions, and 1,4-additions. A pool-transformation approach efficiently explored the scope of these transformations for nine different building blocks and synthesized a >170-member library with enhanced chemical space coverage and favorable drug-like properties. Screening revealed hits against CDK2. This work establishes the build–couple–transform concept for the synthesis of lead-like libraries and provides a differentiated approach to libraries with significantly enhanced scaffold diversity.

## Introduction

High-throughput screening (HTS) is commonly a key initial step in the search for small molecule modulators of biological targets:^1^ the synthesis of lead-like libraries is critical to generate compound collections for HTS.^2^ Lead-likeness is a concept developed to provide guidance on the ideal physicochemical properties of library members and, in part, to address the observation that lead-to-candidate optimization programs often result in increased molecular weight and lipophilicity.^3,4^ Lead-like compounds can be loosely defined as those containing functional groups and physicochemical properties compatible with in vivo activity and safety. Their desirable properties, such as low lipophilicity (e.g., clogP ≤ 4.5), size (molecular weight of ≤450), and hydrogen bonding capacity (number of hydrogen bond donors of ≤5 and acceptors of ≤8),^5,6^ permit further optimization within the property space considered most desirable for drug candidates.^3,7^ The production of large numbers of compounds that are chemically diverse and also fit within the criteria for lead-likeness is challenging.^8−10^

**Scheme 1:**
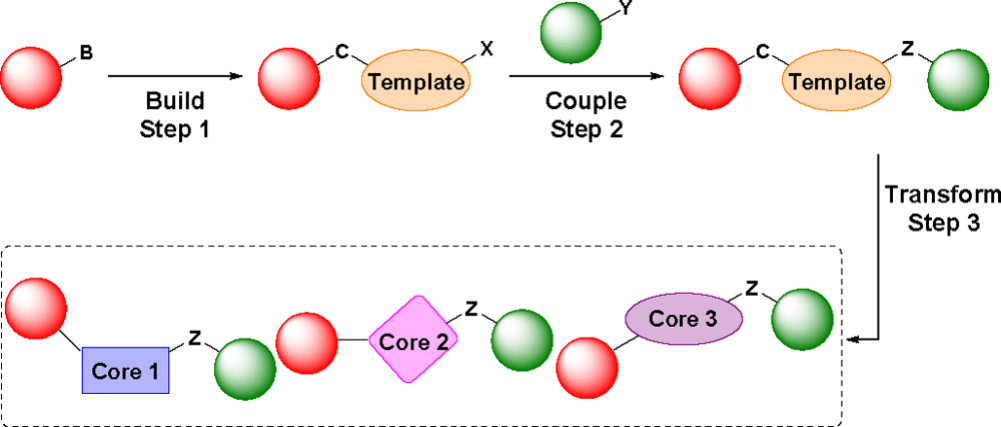
Library Design Using the Build–Couple–Transform Paradigm That Generates Diverse Cores in the Transform Step

The majority of screening libraries are constructed using common synthetic steps that rely on the addition of readily available building blocks to a common scaffold (Scheme 1). While this is a well-established approach, screening sets made from such libraries rely on the combination of multiple libraries to generate core diversity.^11,12^

Conceptually, the ideal approach generates a diverse set of central scaffolds from a precursor template, already bearing a range of substituents, through a set of late-stage chemical transformations. This idea is exemplified by the build–couple–pair algorithm that is used extensively in diversity oriented synthesis:^13−16^ building blocks are assembled (build), joined (couple), and then linked in a third operation (pair) to generate final compounds, often macrocycles, with the templates inspired by natural products.^17−20^

We aimed to evolve this concept to provide an approach for the generation of a library containing compounds with more traditional lead-like scaffolds focused on different heteroaromatic templates. Ideally, the resulting cores would populate a diverse set of geometries, presenting the substituents via different vectors while also containing differing functionality within the cores.

This concept is much harder to realize than linear library synthesis based on a single scaffold. We surmised that it could be achieved using a similar build–couple strategy in which a central template with functionalities capable of multiple reaction modes is generated in the build step, with diverse building blocks appended in the couple phase. This central template could then be transformed into multiple diverse scaffolds using an array of different reactions (evolved from pair and referred to herein as transform). Hence, this build–couple–transform protocol could give rise efficiently to a highly diverse lead-like library (Scheme 1).

## Results and Discussion

The implementation of the build–couple–transform library design requires the identification of a central template with multiple modes of reactivity. We selected a 4-oxo-2-butenamide system **1** for this purpose.

**Scheme 2:**
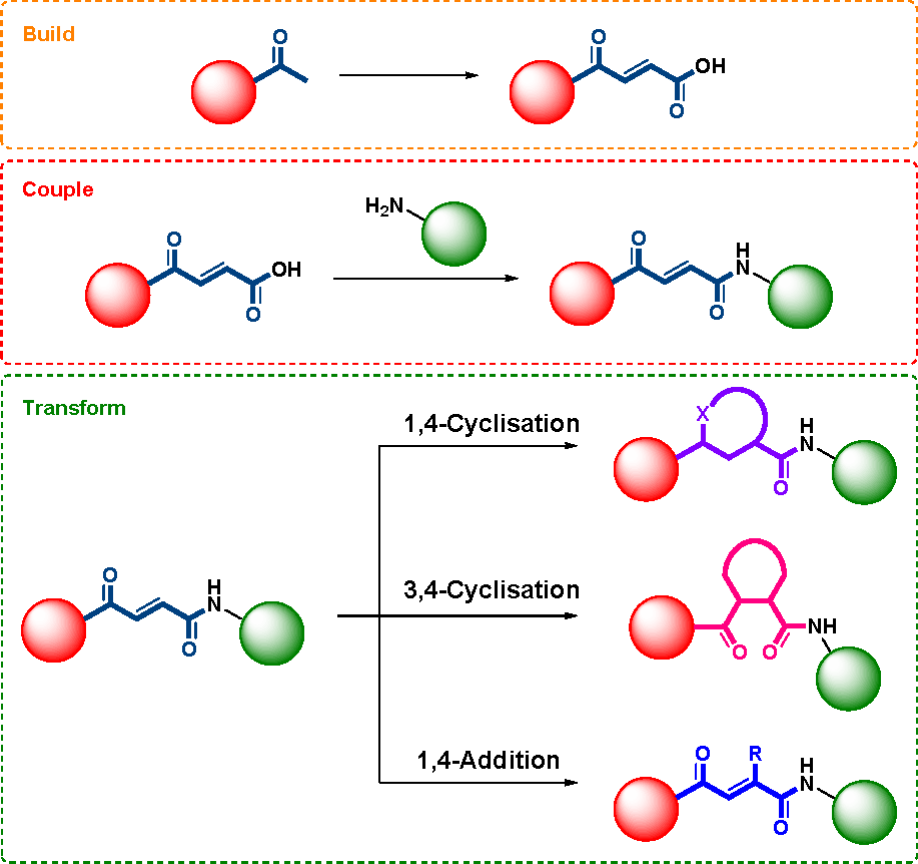
Illustration of the Transformations To Form Diverse Cores Based on the Ketoenamide Template Described

This moiety is attractive because it is capable of undergoing addition or condensation reactions at multiple points, which can also be combined to give multiple modes of cyclization (Scheme 2). 1,4-Cyclization and 3,4-cyclization reactions give diverse cyclic templates with differentiated defined geometries and shapes (from disk-like to more spherical) and differing vectors and patterns of hydrogen bond donors and acceptors. Simple 1,4-additions to the enone system and selective reduction reactions generate further diversity. Accordingly, we investigated the synthesis of a set of 4-oxo-2-butenamide templates and the development and optimization of a range of transformations within the above categories that could be employed in the transform step.

### Build–Couple Steps

A total of 11 4-oxo-2-butenamide templates were synthesized in the build–couple stage of the library synthesis, with the build stage generating carboxylic acid building block **2** and with the couple stage linking the carboxylic acid **2** with the second amine building block **6**.

**Scheme 3:**
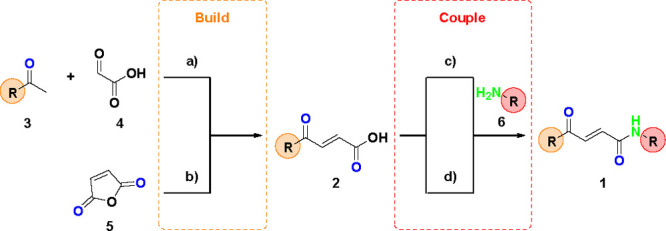
Synthetic Route for the 4-Oxo-2-butenamide Intermediates **1** Reactants and conditions: (a) pyrrolidine (1.0 equiv), AcOH (1.0 equiv), MeOH, microwave, 60 °C, 8 h *or* TsOH (1.0 equiv), dioxane, microwave, 160 °C, 1 h; (b) R (1.0 equiv), AlCl_3_ (1.5 equiv), DCM, rt, 5 h (R = electron rich aromatic group); (c) NH_2_R (1.0 equiv), HATU (1.5 equiv), DIPEA (1.2 equiv), DCM, rt, 18 h; (d) NH_2_R (1.3 equiv), POCl_3_ (1.3 equiv), NEt_3_ (3.0 equiv), THF, rt, 3 h.

For those carboxylic acids (**2**) not commercially available, the α,β-unsaturated system was accessed via an aldol condensation between the appropriate methylketone **3** and glyoxylic acid **4** using microwave conditions with pyrrolidinium acetate or *p*-toluenesulfonic acid (Scheme 3a).^21^ Alternatively, electron rich arenes directly underwent Friedel–Crafts acylation with maleic anhydride **5** to generate carboxylic acids **2** in moderate yields (Scheme 3b).

The couple step then added the second building block by combining the carboxylic acid **2** with the appropriate amine **6**. This was achieved using either HATU (Scheme 3c) or phosphorus(V) oxychloride (Scheme 3d) mediated amide coupling. Both synthetic routes gave excellent yields to the ketoenamide scaffolds **1**.

Two scaffolds were selected on which to develop and validate the transform chemistry: (1) with both building blocks consisting of a simple phenyl ring (**1a**); (2) with building blocks comprising polysubstituted methoxyphenyls (**1b**). A further nine scaffolds were chosen to establish scope and tolerance of drug-like functionality to provide the basis of a lead-like library. Four scaffolds were chosen with the amide building block remaining as a phenyl ring and the ketone building block containing methoxyphenyl (**1c**), cyanophenyl (**1d**), cyclohexyl (**1e**), or tetrahydropyran-4-yl (**1f**). The final five scaffolds maintained the phenyl ketone building block and explored the scope of amide substituents with a piperidine (**1h**), a morpholine (**1i**), an ether chain (**1j**), an aminopyrazole (**1k**), or a benzyl group (**1l**) (see Scheme 7).

### Development of the Transform Reactions: 1,4-Cyclizations

**Scheme 4:**
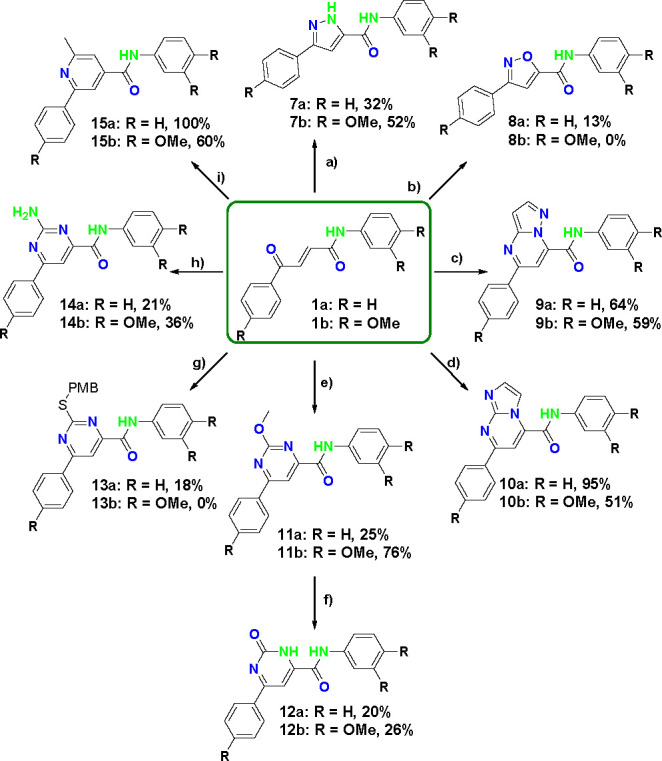
1,4-Cyclization Transformations of Compounds **1a** and **1b** Reactants and conditions: (a) NH_2_NH_2_.H_2_O (2.0 equiv), EtOH, reflux, 2 h, then MnO_2_ (12 equiv), DCM, reflux, 2 h; (b) NH_2_OH·HCl (2.0 equiv), NaOAc (4.0 equiv), DMSO, 60 °C, 6 h, then MnO_2_ (12 equiv), DCM, reflux, 2 h; (c) 3-aminopyrazole (1.3 equiv), DMF, 110 °C, 3 days; (d) 2-aminoimidazole sulfate (2.0 equiv), NaHCO_3_ (3.0 equiv), DMF, 110 °C, 3 days; (e) methyl carbamimidate (1.3 equiv), NaHCO_3_ (4.0 equiv), DMF, 75 °C, 5 h; (f) HCl (20 equiv), EtOH, microwave, 100 °C, 2 h; (g) 2-PMB-isothiouronium chloride (1.3 equiv), NaHCO_3_ (4.0 equiv), DMF, 75 °C, 6 h; (h) guanidine carbonate (1.1 equiv), DMSO, 60 °C, 3 h; (i) *N*-(methylacetyl)pyridinium chloride (1.0 equiv), NH_4_OAc (3.0 equiv), EtOH, 75 °C, 2 h.

Taking advantage of the Michael acceptor property of the 4-oxo-2-butenamide template of compound **1**, a total of eight one-step 1,4-cyclization reactions were developed (Scheme 4). Drug-like scaffolds can be generated via late-stage diversification: pyrazoles, isoxazoles, pyrimidines, pyrimidones, pyridines, imidazopyrimidines, and pyrazolopyrimidines. These various heterocycles provide distinctly varied cores with differing polarity, H-bond donor/acceptor combinations and orientations, substituent vector geometry.

It was anticipated that pyrazole **7** could be accessed by the condensation of tosylhydrazide with the Michael acceptor templates **1a** and **1b**.^22^ However, diimiide reduction dominated over the cyclization, despite attempts to optimize the reaction (Table S1). The pyrazole-containing compounds **7a** and **7b** were finally obtained by condensation of hydrazine with the ketoenamide (**1**) without the addition of base. The reaction yielded a mixture of pyrazole and dihydropyrazole. Treatment of the crude mixture with manganese dioxide allowed the conversion of the dihydropyrazole to the pyrazole such that compounds **7a** and **7b** were obtained with 32% and 52% yields.

Isoxazole **8** was obtained in an analogous manner: reacting hydroxylamine with common intermediates **1a** and **1b**, after optimization of the required base (Table S2). Manganese dioxide oxidation converted remaining dihydroisoxazole into the desired isoxazole **8a** in 13% yield.

Pyrazolo[1,5-*a*]pyrimidine **9** and imidazo[1,2-*a*]pyridine **10** were accessed by condensation of the corresponding amino heterocycle across the Michael acceptor (**1**).

3-Aminopyrazole condensed with ketoenamides **1a** and **1b** to give pyrazolo[1,5-*a*]pyrimidines **9a** and **9b** in 64% and 59% yields. Reactions of **1a** and **1b** with 2-aminoimidazole hemisulfate in the presence of sodium hydrogen carbonate gave rise to **10a** and **10b** in 95% and 51% yields, respectively.

Attempts to obtain pyrimidones and mercaptopyrimides by condensation of templates **1a** and **1b** with urea and thiourea gave unclean reaction profiles with dimeric adducts as the major products (Table S3). However, methyl carbamimidate hydrochloride and 2-(4-methoxybenzyl)isothiouronium chloride in the presence of sodium bicarbonate (other bases seemed to modify the reaction outcome, Table S4) yielded 2-methoxypyrimidines **11a** and **11b** in respective yields of 25% and 76% and 2-PMB-thiopyrimidine **13a** in a yield of 18%. 2-PMB-thiopyrimidine **13b** was not formed, instead an adduct from the Michael addition of PMB thiolate to **1b** was obtained. Deprotection of 2-methoxypyrimidines **11a** and **11b** using hydrochloric acid unveiled amides **12a** and **12b** with yields of 20% and 26%, respectively.

It was envisaged that differently functionalized pyrimidine rings could be formed by the reaction of guanidium salts with intermediate **1**. Hence, guanidine carbonate was reacted with **1a** and **1b** to form 2-aminopyrimidine **14**, in the absence of base, which was linked to the observation of some polymeric byproducts (Table S5). Interestingly, some reduced alkene byproduct was also obtained during the reaction. The products **14a** and **14b** were successfully obtained in respective 21% and 36% yields.

Reaction of **1a** and **1b** with 1-(2-oxopropyl)pyridinium (Kröhnke pyridine synthesis) yielded compounds **15a** and **15b** in 100% and 60% yields, respectively.

This set of reactions provided the first eight transform procedures. Notably, these 1,4-cyclizations afford five-membered and six-membered rings, as well as fused ring heterocycles with different properties, especially the number and conformation of H-bond donors and acceptors.

### 3,4-Cyclization Transformations

**Scheme 5:**
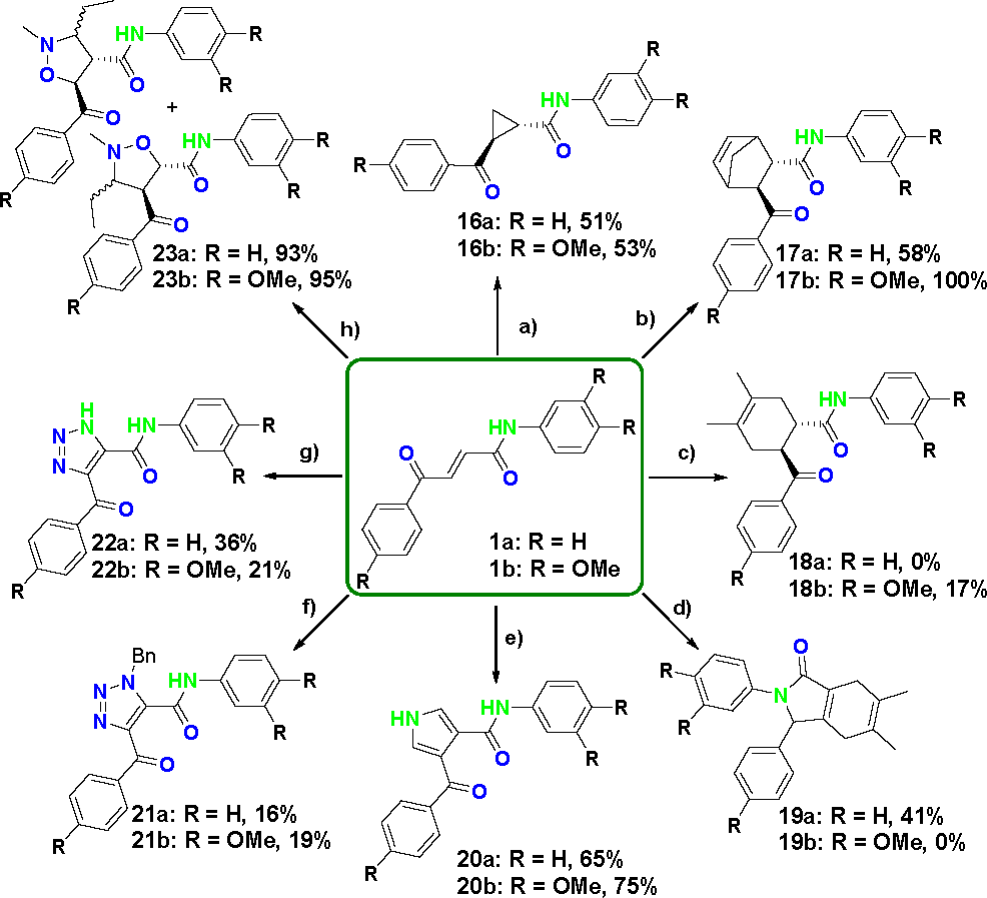
3,4-Cyclization Transformations of Compounds **1a** and **1b** Reactants and conditions: (a) Me_3_S(O)I (1.2 equiv), NaH (1.2 equiv), DMSO, rt, 2 h; (b) cyclopentadiene (20.0 equiv), Yb(OTf)_3_ (1.2 equiv), MeCN, rt, 2 h; (c) 2,3-dimethylbuta-1,3-diene (10 equiv), Yb(OTf)_3_ (1.2 equiv), MeCN, 110 °C, 30 min; (d) 2,3-dimethylbuta-1,3-diene (10.0 equiv), Yb(OTf)_3_ (1.2 equiv), MeCN, 110 °C, microwave, 30 min; (e) TosMIC (1.1 equiv), NaH (2.2 equiv), Et_2_O, DMSO, rt, 1.5 h; (f) BnN_3_ (2.0 equiv), K_2_CO_3_ (2.0 equiv), H_2_O, dioxane, 80 °C, 18 h; (g) NaN_3_ (1.0 equiv), CuO (1.0 equiv), DMF, 80 °C, 48 h; (g) *N*-(propylidene)methylnitrone (2.4 equiv), DCM, 60 °C, 18 h.

The common intermediate **1** contains an electron deficient alkene that can undergo 3,4-cycloaddition reactions with electron rich partners. Seven different reactions were developed to give access to both aromatic and nonaromatic three- to six-membered rings, providing increased three-dimensionality (Scheme 5).

Classic Corey–Chaykovski conditions transformed the alkene bond into a cyclopropane to give compounds **16a** and **16b** in 51% and 53% yields.

Diels–Alder reactions were chosen since they would give access to partially saturated six-membered rings with increased lipophilicity and three-dimensionality.^23−25^ Furthermore, variation of the diene would alter the conformation of products. Optimization was carried out with 2,3-dimethylbuta-1,3-diene (Tables S6 and S7), ultimately identifying ytterbium(III) trifluoromethanesulfonate as the optimal Lewis acid. For cyclopentadiene this gave the expected bicycloheptenes **17a** and **17b** in 58% and 100% yields, respectively. Surprisingly, for 2,3-dimethylbuta-1,3-diene, the expected cyclohexene **18a** was not formed and instead the product was fused-lactam **19a** (structure confirmed by NMR and X-ray) arising by intramolecular condensation of the amide nitrogen with the ketone, after the initial Diels–Alder reaction, followed by dehydration and isomerization of the resulting double bond. This serendipitous outcome gave rise to a unique conformation for the scaffold, which added additional diversity to the library resulting in lactam **19a** in 41% yield. For electron-rich scaffold **1b**, the expected cyclohexene **18b** was isolated in 17% yield.

Given the electron-deficient nature of the 4-oxo-2-butenamide alkene, the Van Leusen synthesis was an attractive strategy to access pyrrole scaffold. TosMIC was applied with sodium hydride to generate pyrroles **20a** and **20b** in 65% and 75% yields, respectively.

A five-membered heterocycle prevalent in drug discovery is triazole. Although traditionally accessed through an azide–alkyne [3 + 2] cycloaddition, azide–alkene [3 + 2] cycloadditions also have precedent for combining an electron-deficient alkene with an electron-rich azide.^26^ Benzylazide was reacted with common intermediate **1** to provide triazoles **21a** and **21b** in 16% and 19% yields. More recent literature suggests copper promoted aerobic oxidative azide–alkene cycloadditions are possible for electron-deficient olefins, as present in common intermediate **1** with a broader range of azides.^27^ Thus, after the copper additive was optimized (Table S8) copper(II) oxide gave triazoles **22a** and **22b** in 36% and 21% yields.

Finally, it was envisaged that [3 + 2] cycloaddition strategies using various 1,3-dipoles with the electron-deficient olefin could be used to access saturated five-membered heterocycles. This was exemplified using *N*-(propylidene)methylnitrone, which gave rise to both diastereomers of each regioisomer. The mixture of isomers was isolated in 93% and 95% yields for **23a** and **23b**, respectively. Subsequent semipreparative HPLC separated the isomers (see Supporting Information).

Seven different one-step 3,4-cyclization transformations were successfully established. Thus, both aromatic and aliphatic three- to six-membered rings, as well as some fused bicyclic compounds, were produced, with three-dimensional stereochemical, physicochemical, and pharmacophoric differentiation.

### Reductive Transformations

**Scheme 6:**
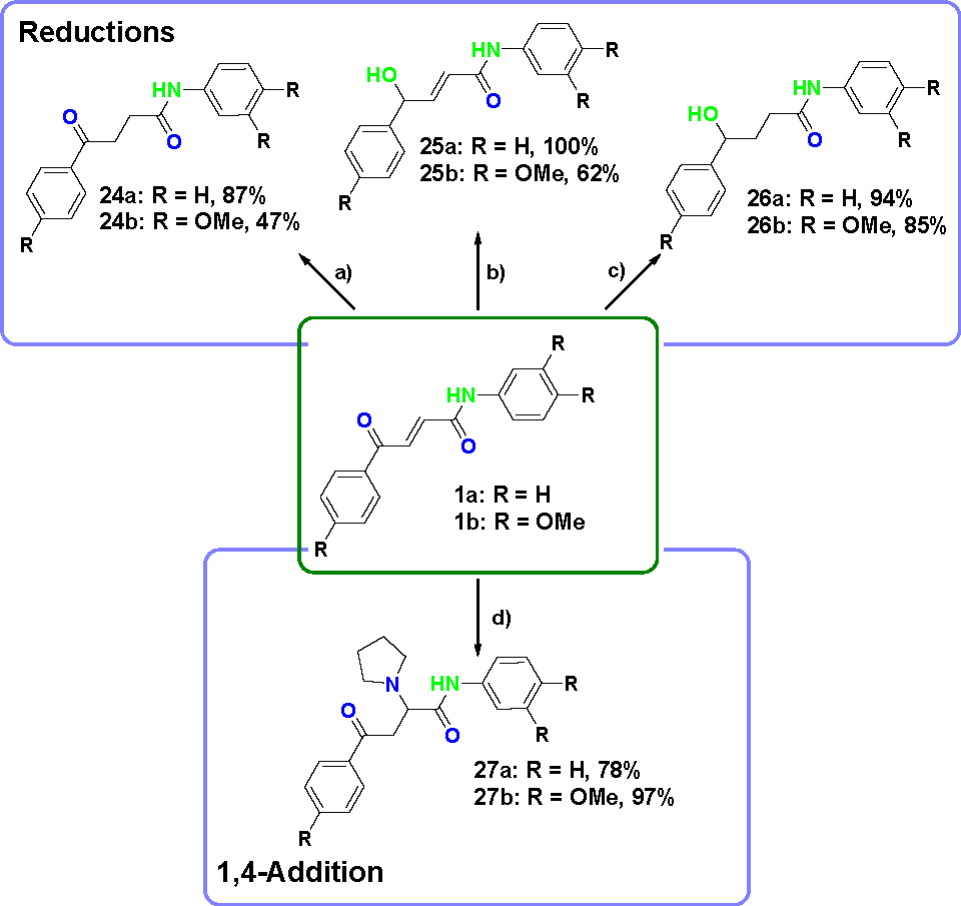
1,4-Addition and Reduction Transformations of Compounds **1a** and **1b** Reactants and conditions: (a) H_2_, Pd/C (10 mol %), MeOH, H-Cube, rt, 30 min; (b) CeCl_3_·7H_2_O(1.0 equiv), then NaBH_4_ (1.0 equiv), DCM, MeOH, rt, 15 min; (c) NiCl_2_·6H_2_O (0.5 equiv), NaBH_4_ (5.0 equiv), DCM, MeOH, rt, 1.5 h; (d) pyrrolidine (1.5 equiv), EtOH, rt, 5 min.

The presence of the ketone and the conjugated alkene within the 4-oxo-2- butenamide scaffold offers the possibility of further transformations to additional core scaffolds. Three reduction products were obtained from intermediate **1** (Scheme 6). These compounds provide different properties to the set of compounds increasing the targeted chemical space.

Selective reduction of the conjugated alkene was possible by catalytic hydrogenation with palladium on carbon to give compounds **24a** and **24b** in 87% and 47% yields.

Selective reduction of the ketone can be performed using Luche reduction conditions, yielding compounds **25a** and **25b** in 100% and 62%.

Reduction of the α,β-unsaturated ketone was achieved using nickel chloride and sodium borohydride to give compounds **26a** and **26b** in 94% and 85% yields. The three reduction products have different geometries and physicochemical properties covering a broader region of chemical space.

### 1,4-Addition Transformations

The conjugated alkene also allows the selective reaction of position 4 through 1,4-addition (Scheme 6). 1,4-Addition of pyrrolidine to common intermediate **1** provided products **27a** and **27b** in respective yields of 70% and 97%.

The resulting compounds underwent retro-Michael additions in the presence of acid and were unstable at room temperature. Nevertheless, the 1,4-addition reaction led to two exemplar compounds with increased size, aromaticity, and lipophilicity while keeping the heteroatoms of the starting material in place.

### Summary of Transformations

Overall, 19 one-step transformations were applied to two exemplar 4-oxo-2-butenamide scaffolds utilizing the high reactivity of this Michael acceptor to form a collection of structurally diverse scaffolds with both aromatic and saturated ring systems. Furthermore, the transformations described have introduced various hydrogen bond donors and acceptors, increased the three-dimensionality, and tuned the lipophilicity of the compounds. A second step was exemplified by deprotection of the methoxy forming pyrimidones **12a** and **12b** to demonstrate the potential for further elaboration. This extended the library to a total of 20 available scaffolds, with diverse structures and properties, by applying a range of transformations to one common intermediate.

### Library Synthesis: Pooled Transformations

**Scheme 7:**
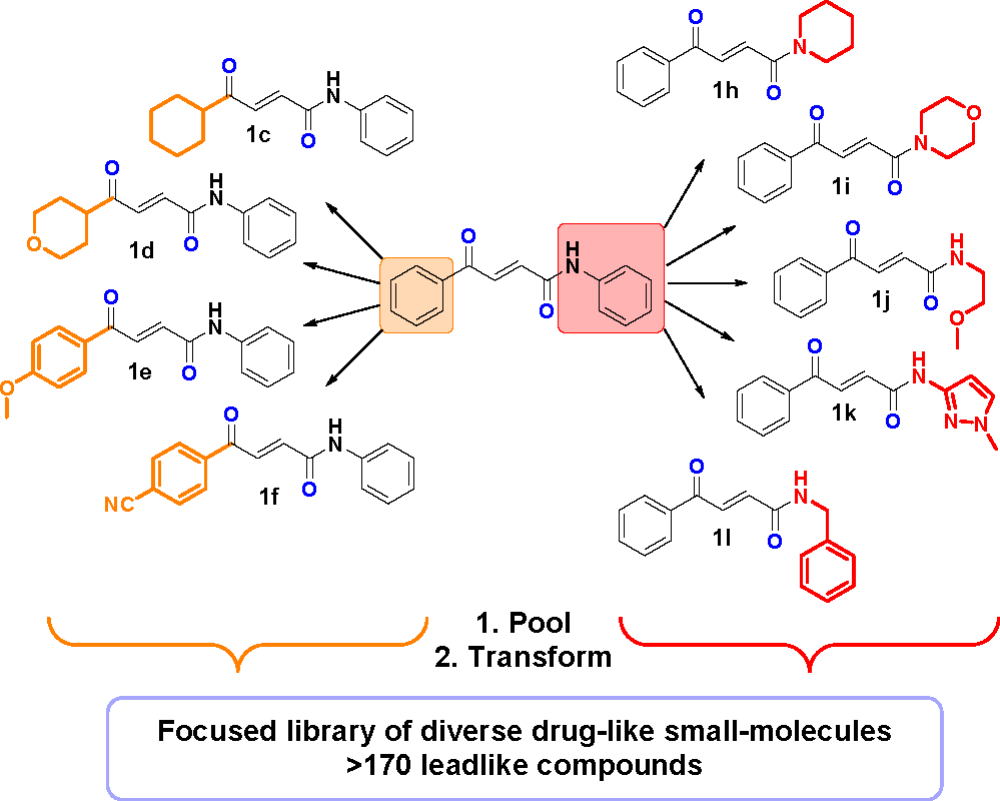
Ketoenamide Intermediates Demonstrating the Scope of the Ketone Building Block (Orange) and Amide Building Block (Red) Pooled and Transformed To Generate a Focused Lead-like Library of 174 Diverse Small Molecules

With the 19 one-step transformations fully optimized, focus moved toward demonstrating both the scope of the chemistry and its application to library synthesis. The nine 4-oxo-2-butenamide scaffolds **1c**–**1l** were pooled into two sets: four with variation of the ketone building block (orange) and five with variation of the amide building block (red) and the transformations applied to these pools (Scheme 7).

The pooled transformations were analyzed by HPLC and HRMS to ensure complete conversion to the desired scaffolds (see Supporting Information). It was expected that given the clean reaction profiles, these pooled mixtures could be used for biological screens, such as employing surface plasmon resonance (SPR) or X-ray crystallography without further purification or separation.

The application of the 19 one-step transformations to the two pools of four and five intermediates led to a diverse library of 174 compounds (Table 1).

**Table 1:**
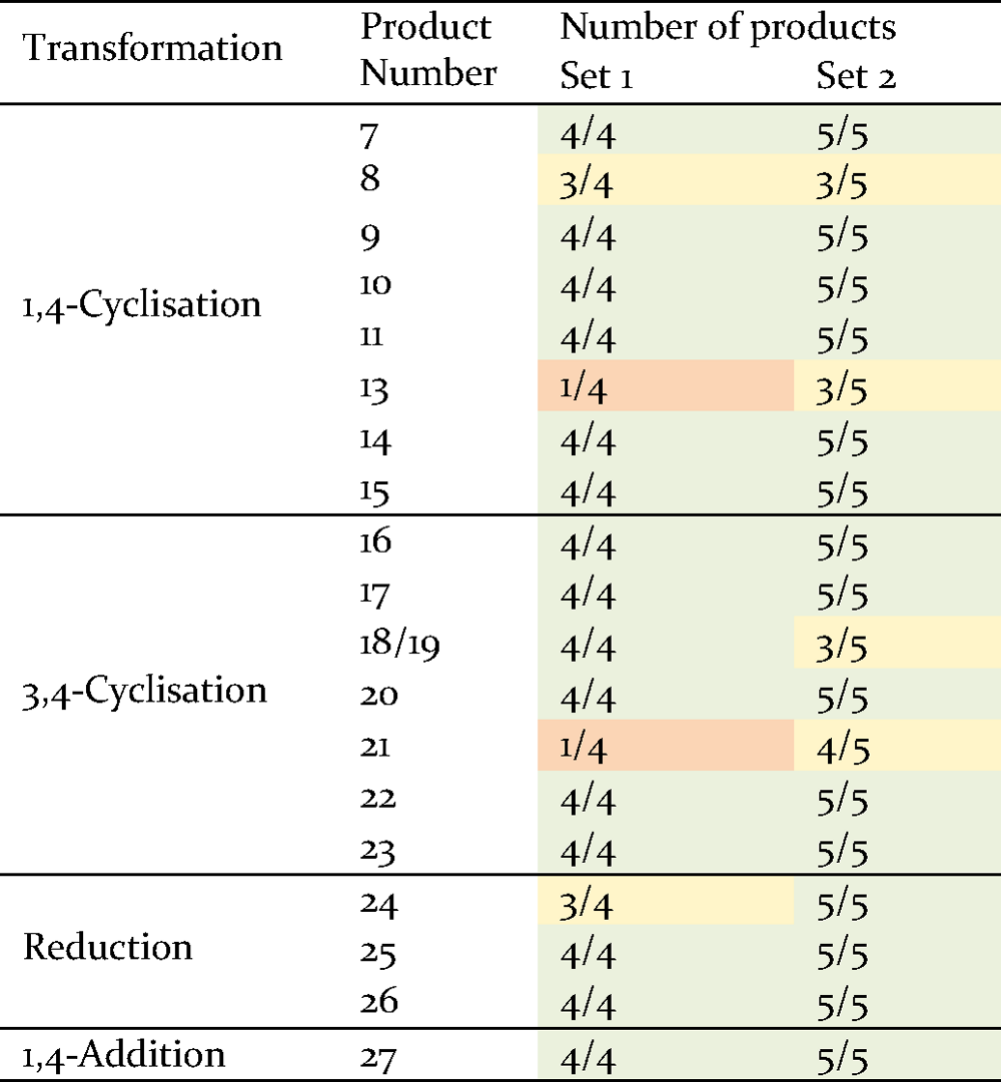
Scope Evaluation of the 19 DOS Transformations^a^

a Green (100% of products observed), yellow (>50% of products observed), orange (>20% of products observed), red (0% of products observed).

The resulting mixtures of the pooled transformations after 1,4-cyclizations on both sets of compounds showed the formation of all desired products for pyrazole, aminopyrimidine, methoxypyrimidine, pyridine, pyrazolo[1,5-*a*]pyrimidine, and imidazo[1,2-*a*]pyrimidine. These results demonstrate the wide scope and robustness of the transformations. After application of the conditions to form both the isoxazole and PMB-thiopyrimidine cycles, only six and five out of the expected nine products were observed respectively. The isoxazole transformation appeared incompatible with electron-deficient substrates, whereas the PMB thiolate underwent a Michael addition instead of the desired pyrimidine ring formation.

Cyclopropanation, pyrrole, isoxazolidine, and cyclopentadiene Diels–Alder 3,4-cyclizations yielded all the desired products after pooled transformations, suggesting a large substrate compatibility. Although electron-deficient substituted amides did not provide the desired triazole, all the other products were obtained. The benzyltriazole [3 + 2] cycloaddition failed to provide products for electron-deficient and aliphatic substituted ketones, whereas all triazoles were formed irrespective of the amide substituent. Finally, the Diels–Alder reaction with 2,3-dimethyl-1,3-butadiene required electron-withdrawing substrates to form cyclohexene adducts, which underwent spontaneous cyclization to scaffold **19**, as observed with precursor **1a**.

All expected products were obtained by applying the three reduction transformations to both sets of pooled compounds. Finally, the 1,4-addition transformation successfully generated all the desired products demonstrating the compatibility of this transformation with a large range of substrates.

The pooled transformations demonstrated that the different 19 one-step reactions are able to generate the desired products when applied to a common intermediate with various properties. Thirteen transformations are compatible with all types of building blocks and proved to be reliable. The six other transformations were observed to be compatible with most substrates, although careful molecule design would enhance the reliability of the reactions as described above. Therefore, we demonstrated the potential of the build–couple–transform protocol to efficiently provide a library of diverse small molecules with desirable drug-like properties.

### Assessment of Diversity

A range of physicochemical properties were calculated for the transformed library and for the 4-oxo-2-butenamide precursors (Table 2). All 174 compounds of the library were compliant with both Lipinski’s rule of 5 and Kopple’s guidelines^28,29^ and therefore lie wholly within drug-like chemical space. Moreover, 42.7% of the library compounds were within lead-like chemical space, exhibiting desirable properties for drug discovery start points.

**Table 2:** Mean Physicochemical Properties Calculated for the Transformed Library, the 4-Oxo-2-butenamide Templates, and the Enamine Discovery Set^30^

property	ideal^a^	this work	templates	Enamine
clogP	–1 to 3	2.2	2.0	1.3
MW	200–350 or <450	326	264	332
HBA	<8	5.1	3.9	6.2
HBD	<5	1.0	0.8	1.3
HAC	14 to 26	24	20	24
Fsp^3^	high	0.38	0.21	0.56
nAr	≤3	2.0	1.6	1.7
RBC	<10	4.8	4.5	4.4
tPSA	<140	64.5	54.2	79.4

a Based on guidelines.^5,6^

Pleasingly, the chosen transformations resulted in comparable lipophilicity relative to the starting templates (2.0–2.2) and number of hydrogen bond acceptors and donors (3.8–4.9 and 0.84–1.3, respectively) without a large increase in molecular weight (264–326). Furthermore, this library demonstrates comparable physicochemical properties when compared to existing commercial drug-like libraries as shown by comparison with the Enamine Discovery Diversity Set (Table 2). In addition, the shape index and molecular flexibility are maintined after transformations, despite many of these involving cyclizations (Table 3). However, the molecular complexity and drug-likeness are both seen to increase, demonstrating the utility of these transformations for generating useful small molecules for screening libraries.

**Table 3:** Mean Shape Properties Calculated for the Transformed Library and the 4-Oxo-2-butenamide Templates Compared to the Enamine Discovery Set^30^

property^a^	this work	templates	Enamine Discovery Set
molecular flexibility	0.39	0.40	0.49
molecular complexity	0.79	0.59	0.78
shape index	0.57	0.70	0.57
drug-likeness	2.0	–0.086	2.9

a Druglikeness calculated by summing the score value of drug-like fragments in the compound as compared to a database taken from marketed drugs.

**Figure 1:**
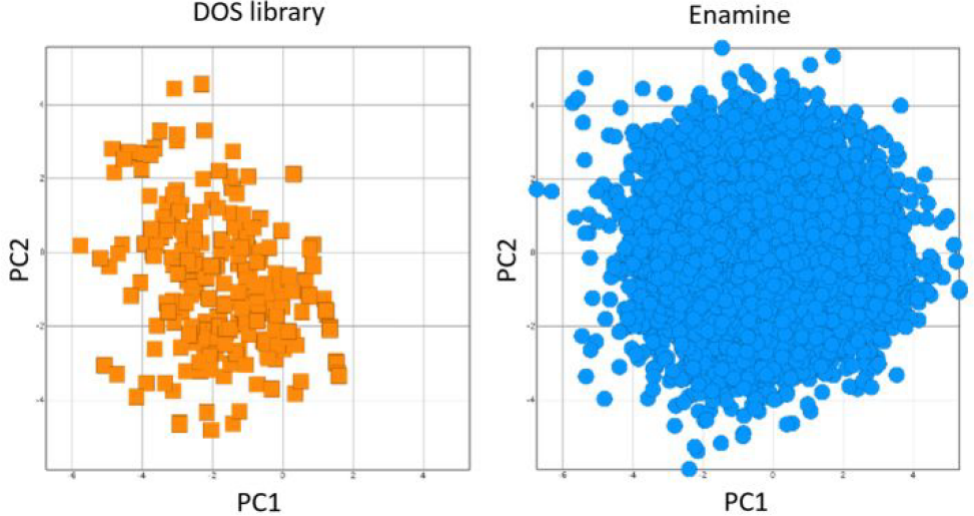
PCA analysis plots of our DOS library (orange) compared with the Enamine Discovery Set (blue) demonstrating the high chemical coverage of our DOS library. See Supporting Information for further details.

Visualization of the chemical space coverage was performed by comparison of PCA and PMI analysis plots of our DOS library with the commercial Enamine Diversity Set. Whereas the Enamine Diversity Set contains 10 240 compounds with enhanced diversity, our DOS library, made up of 174 compounds with 20 different scaffolds, covered half the Enamine chemical space defined by the PCA with 30 times fewer compounds (Figure 1).

**Figure 2:**
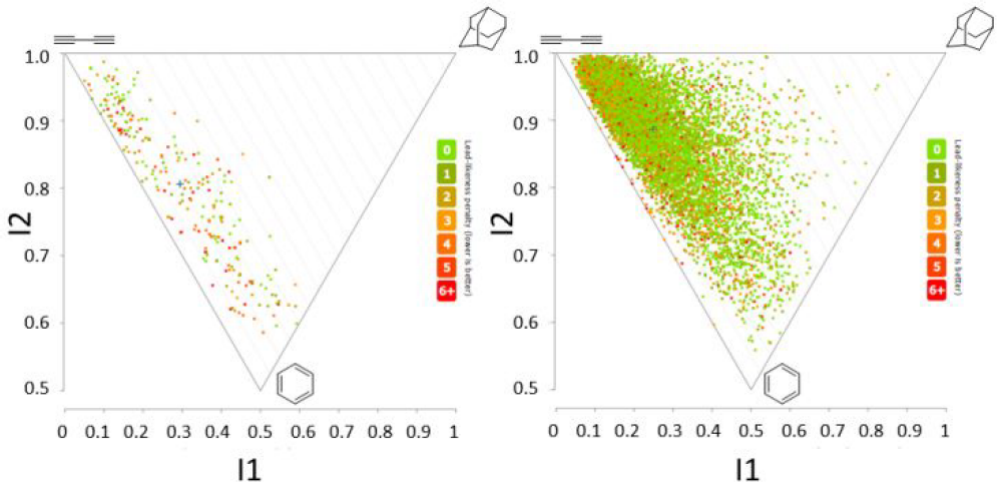
PMI analysis plots of our first DOS library (left) compared with the Enamine Discovery Set (right) showing a comparable shape distribution for the DOS library. Mean PMI coordinates for each library are denoted by (**+**): DOS (**I1** = 0.294, **I2** = 0.806), Enamine (**I1** = 0.248, **I2** = 0.887). See Supporting Information for further details.

The PMI analysis shows that the DOS library has a good distribution between rod and flat shapes (Figure 2).

Comparison with the PMI analysis of the Enamine Diversity Set shows a better general distribution of the shapes for our library with no redundancy. Again, this suggests that the DOS protocol is able to provide a library with an enhanced coverage of different molecular shapes while maintaining a small library size.

### Biological Analysis: DOS library screening using SPR against CDK2

**Figure 3:**
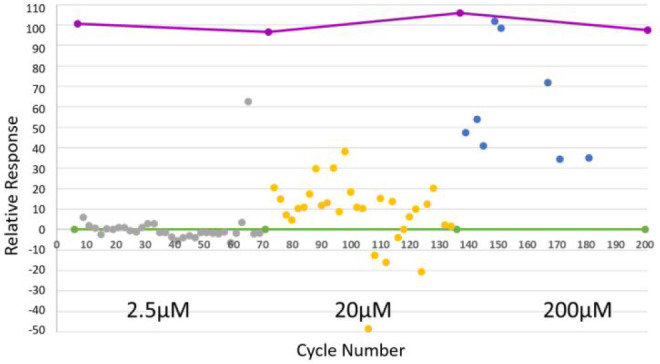
Assessment of DOS library through SPR: screening of DOS library against CDK2. Screening was performed at three concentrations 2.5 μM (gray), 20 μM (yellow), 200 μM (blue), with positive control compound SU9516 (pink) and negative control DMSO (green).

To further demonstrate biological relevance, each pool of four or five compounds was screened against a representative protein target (cyclin dependent kinase 2, CDK2) by SPR at three concentrations (2.5, 20, and 200 μM) (Figure 3).

**Figure 4:**
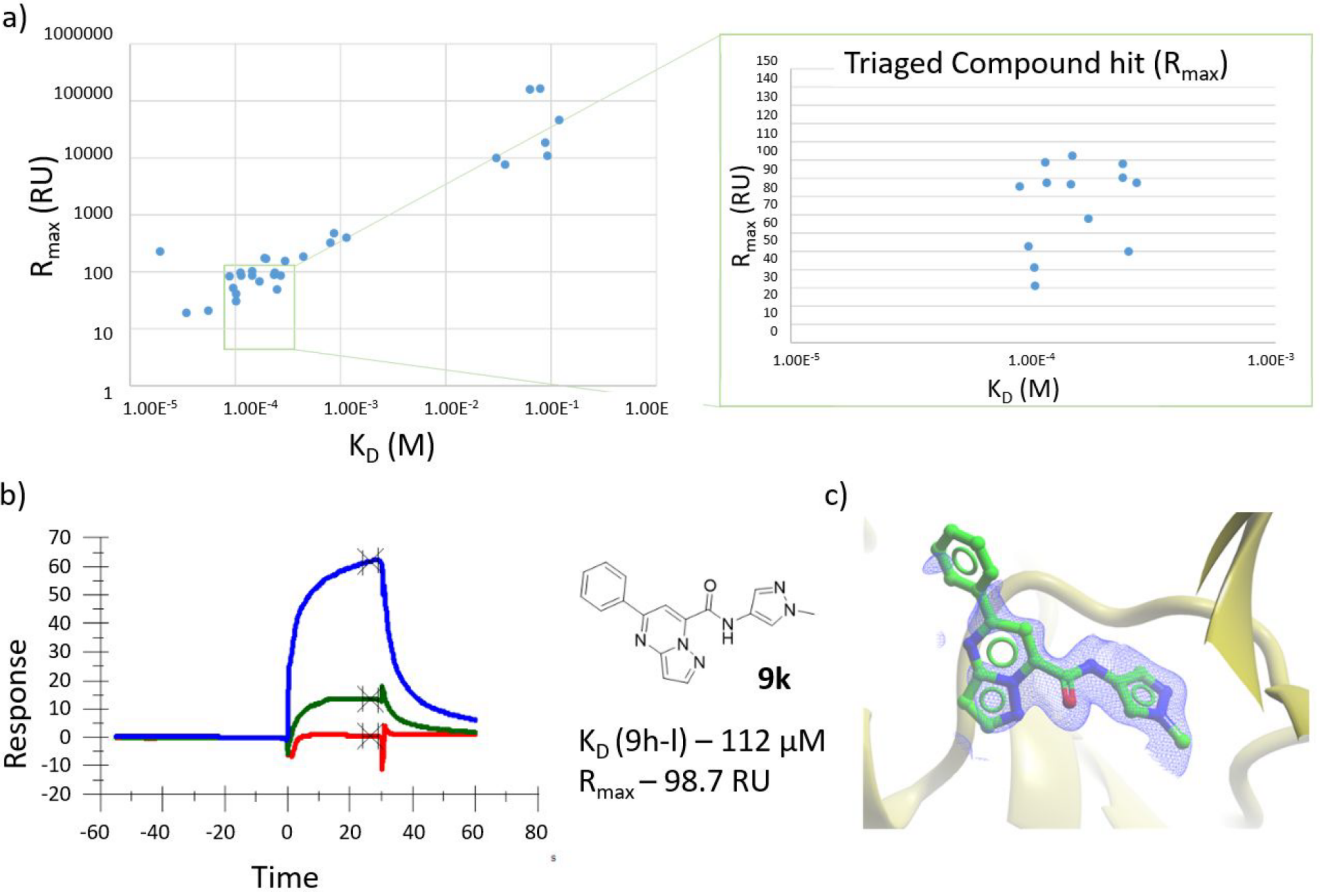
Assessment of DOS library compound pools through SPR and crystallography against exemplar target CDK2. (a) The fitted data were triaged by plotting predicted *R*_max_ and *K*_D_ for each pool to remove outliers. The projected *R*_max_ (RU) of the library with average molecular weight ∼302 Da against the CDK2 immobilized surface of ∼8100 RU is ∼72 (RU). Therefore an estimated ±50% window of was used. (b) Of the 13 triaged pools, we show the SPR sensogram of pool **9h**–**l** over the three point titration (2.5, 20, 200 μM) with a projected *K*_D_ and *R*_max_ of 112 μM and 98.7 RU, respectively. (c) Result of crystal diffusion experiment where pool **9h**–**l** was soaked into an apo CDK2 crystal and X-ray data collected. Compound **9k** was identified as the hit compound through matching of the electron density. **9k** (green stick), CDK2 (gold ribbon), and 2*F*_o_ – *F*_c_ electron density map (blue mesh) (PDB code 7ZPC).

The projected *R*_max_ of the library, with average molecular weight of ∼302 Da against the CDK2 immobilized surface of ∼8100 RU, is ∼72 RU. Sensograms of the screening results were fitted over the three concentrations and triaged by plotting predicted *R*_max_ and *K*_D_ for each pool to remove outliers, where an estimated ±50% window was used (Figure 4a). Thirteen screening pools from the library were identified as potential hits, having *R*_max_ between 31 and 102 RU and *K*_D_ values for the pool between 87 and 270 μM. For example, pool **9h**–**l** had a projected *K*_D_ and *R*_max_ of 112 μM and 98.7 RU, respectively (Figure 4b).

11 of the 13 triaged pools were taken into crystallographic soaking experiments with the exemplar protein CDK2. The kinase domain has been structurally well characterized^31^ with the orthosteric ATP-binding pocket sitting between the N- and C-lobes in which many small molecule hits have been shown to bind.^32^ Other allosteric binding sites have also been identified with small molecule and fragment screening^33^ at sites with potential biological relevance in the development of inhibitors with allosteric modes of action.

A positive hit was identified in crystallographic experiments from compound pool **9h**–**l**. The compound containing the methylpyrazole carboxamide substituent (**9k**) was the only compound to match the electron density of the 2*F*_o_ – *F*_c_ map (Figure 4c).

Analysis of the X-ray structure showed that this compound shows a novel pharmacophore, with the methylpyrazole group embedded toward the back of the orthosteric pocket forming a hydrogen bond network with the backbones of Glu-81 and Leu-83 and π-stacking with Tyr-19. The NH of the amide linker interacts with the hinge via the carbonyl of Leu-83, with the expected acceptor interaction with the hinge instead forming between Asn-3 and a nitrogen in the phenylpyrazolo[1,5-α]pyrimidine ring. The phenylpyrazolo[1,5-α]pyrimidine interacts with the backbone carbonyl of His-84 and NH of Asp-86 resulting in rotation of this core scaffold relative to the methylpyrazole, orientating the phenyl ring toward the top of the opening to interact with the side chain of Glu-8 via van der Waals forces. Reduced density, however, was observed for the phenyl group most likely due to its positioning at the opening of the pocket where it is solvent exposed.

## Conclusions

We have developed a build–couple–transform algorithm within the diversity-oriented synthesis paradigm for the generation of a structurally diverse library of lead-like small molecules. The transformations were developed and validated on two of the build–couple intermediates and then expanded via a pool-transform strategy such that a library of 174 compounds with 20 unique scaffolds was generated. The physicochemical and shape properties of the library were analyzed and demonstrated that the resulting library had excellent drug-likeness and chemical diversity. The mean properties of the library compared very favorably with both the ideal lead-like/drug-like ranges and with existing commercial libraries. This small library has an enhanced chemical space coverage and shape distribution, ideal for hit discovery. Screening of the DOS library using SPR against CDK2 was used to triage the pools of compounds, with 13 of the screened cocktails identified as potential hits. Eleven of these were taken into crystallographic soaking experiments, resulting in the elucidation of a novel hit for CDK2.

Overall, this work provides the opportunity to rapidly improve the chemical space coverage of screening libraries, particularly through the use of pooled synthesis. Furthermore, we have demonstrated that analogues can be screened as pooled mixtures to find hits. Hence the build–couple–transform paradigm provides a new conceptual approach to the synthesis of lead-like libraries with demonstrated biological relevance.

## Experimental Section

### General Information

Chemicals were purchased from commercial suppliers and used without further purification. Thin layer chromatography (TLC) was performed on aluminum plates coated with 60 F_254_ silica from Merck. Flash chromatography was carried out using a Biotage SP4, Biotage Isolera, or Varian automated flash system with Silicycle or GraceResolve normal phase silica gel prepacked columns. Fractions were collected at 254 nm or if necessary on all wavelengths between 200 and 400 nm. Microwave irradiation was performed in a Biotage Initiator Sixty in sealed vials. Reactions were irradiated at 2.45 GHz and were able to reach temperatures between 60 and 250 °C. Heating was at a rate of 2–5 °C/s, and the pressure was able to reach 20 bar. Final compound purity is >95%.

### Analytical Equipment

Melting points were measured using a Stuart automatic melting point SMP40 apparatus. Fourier transform infrared (FTIR) spectra were measured using an Agilent Cary 630 FTIR instrument. The abbreviations for peak description are as follows: b = broad; w = weak; m = medium; s = strong. Ultraviolet (UV) spectra were recorded on a Hitachi U-2900 spectrophotometer and were performed in ethanol. High resolution mass spectrometry (HRMS) was provided by the ESPRC National Mass Spectrometry Service, University of Wales, Swansea, or conducted using an Agilent 6550 iFunnel QTOF LC–MS with an Agilent 1260 Infinity UPLC system. The sample was eluted on Acquity UPLC BEH C18 (1.7 μm, 2.1 mm × 50 mm) with a flow rate of 0.7 mL/min and run at a gradient of 1.2 min 5–95% 0.1% aq HCOOH in MeCN.

LC–MS analyses were conducted using a Waters Acquity UPLC system with photodiode array (PDA) and evaporating light scattering detector (ELSD). When a 2 min gradient was used, the sample was eluted on an Acquity UPLC BEH C18, 1.7 μm, 2.1 mm × 50 mm, with a flow rate of 0.6 mL/min using 5–95% 0.1% HCOOH in MeCN. Analytical purity of compounds was determined using Waters XTerra RP18, 5 μm (4.6 mm × 150 mm) column at 1 mL/min using either 0.1% aq ammonia and MeCN or 0.1% aq HCOOH and MeCN with a gradient of 5–100% over 15 min.

^1^H NMR spectra were obtained using a Bruker Avance III 500 spectrometer using a frequency of 500 MHz. ^13^C spectra were acquired using the Bruker Avance III 500 spectrometer operating at a frequency of 126 MHz. The abbreviations for spin multiplicity are as follows: s = singlet; d = doublet; t = triplet; q = quartet; p = quintuplet; h = sextuplet; m = multiplet. Combinations of these abbreviations are employed to describe more complex splitting patterns (e.g., dd = doublet of doublets).

### General Procedures. Build. General Procedure 1 (**2**)

In a microwave vial, acetyl derivative (1 equiv), glyoxylic acid monohydrate (3 equiv), and TsOH monohydrate (1 equiv) were dissolved in dioxane (2.5 M). The vial was closed and heated in the microwave for 1 h at 160 °C using the low absorption mode. 2 M HCl aqueous solution was added to the mixture if the product was not zwitterionic. This was extracted 3 times with DCM. Combined organic phases were dried (MgSO_4_) and concentrated under pressure. The crude material was purified by flash chromatography.

### General Procedure 2 (**2**)

In a microwave vial, methyl ketone-containing compound (1 equiv), glyoxylic acid monohydrate (3 equiv), and acetic acid (1 equiv) were dissolved in MeOH (2.5 M), then pyrrolidine (1 equiv) was added. The vial was sealed and the mixture stirred for 5 min. The mixture was then irradiated using the microwave (MW) at 60 °C for 8 h. The solvent was removed under vacuum, and the crude material was purified by flash chromatography.

### General Procedure 3 (**2**)

A solution of maleic anhydride **5** (1.0 equiv) and aluminum trichloride (1.5 equiv) in dichloromethane (2.2 M) was stirred at room temperature until all solids had dissolved. The aromatic substrate (1.5 equiv) was then added dropwise and the reaction stirred at room temperature until completed. The reaction was slowly poured into hydrochloric acid (0.2 M aq solution) and extracted with dichloromethane. The combined organic layers were dried (MgSO_4_) and concentrated under reduced pressure. The crude material was purified by flash column chromatography.

### Couple. General Procedure 4 (**1**)

In a RB flask, carboxylic acid **2** (1.5 equiv), HATU (1.5 equiv), and DIPEA (1.2 equiv) were dissolved in anhydrous dichloromethane (4 M). The mixture was stirred for 1 h at rt under inert atmosphere. Amine **6** (1.0 equiv) was then slowly added. The mixture was stirred overnight at rt under inert atmosphere. Water was then added to the mixture, which was extracted with DCM. Combined organic phases were washed with saturated brine, dried (MgSO_4_), and concentrated under pressure. The crude material was purified by flash chromatography.

### General Procedure 5 (**1**)

A solution of carboxylic acid **2** (1.0 equiv) and amine **6** (1.3 equiv) in THF (0.25 M) was stirred at 0 °C for 20 min before the dropwise addition phosphorus(V) oxychloride (1.3 equiv) followed by triethylamine (3.0 equiv). The reaction was stirred at 0 °C for 30 min, then warmed to room temperature and stirred for 3 h. Once complete, the reaction was quenched with water and extracted with ethyl acetate. The combined organic layers were washed with brine, dried (MgSO_4_), and concentrated under reduced pressure. The crude material was purified by flash column chromatography.

### Transformations: 1,4-Cyclizations. General Procedure 6 (**7**)

In a RB flask, ketoenamide **1** (1.0 equiv) and hydrazine monohydrate (2.0 equiv) were dissolved in EtOH (0.25 M). The mixture was stirred at 60 °C for 2 h. Solvent was removed under vacuum. The crude was dissolved in DCM (0.05 M). MnO_2_ (12.0 equiv) was added. The mixture was stirred at 60 °C for 2 h before being filtered through a Celite pad. Solvent was removed under vacuum. The crude material was purified by flash chromatography.

### General Procedure 7 (**8**)

A solution of ketoenamide **1** (1.0 equiv), hydroxylamine hydrochloride (2.0 equiv), and sodium acetate (4.0 equiv) in DMSO (0.13 M) was stirred at 60 °C overnight. The reaction was cooled to room temperature, quenched with water, and extracted with dichloromethane. The combined organic layers were washed with brine, dried (MgSO_4_), and concentrated under reduced pressure. The residue was dissolved in dichloromethane (0.04 M) and manganese(IV) dioxide (12.0 equiv) and stirred at 60 °C for 4 h. The crude material was purified by flash column chromatography.

### General Procedure 8 (**9**)

A solution of ketoenamide **1** (1.0 equiv) and 3-aminopyrazole (1.3 equiv) in DMF (0.15 M) was stirred at 110 °C for 3 days. The reaction was cooled to room temperature, then concentrated under reduced pressure. The crude material was purified by flash column chromatography.

### General Procedure 9 (**10**)

A solution of ketoenamide **1** (1.0 equiv), 2-aminoimidazole sulfate (2.0 equiv), and sodium hydrogen carbonate (3.0 equiv) in DMF (0.15 M) was stirred at 110 °C for 3 days. The reaction was cooled to room temperature, then concentrated under reduced pressure. The crude material was purified by flash column chromatography.

### General Procedure 10 (**11**)

In a RB-flask, ketoenamide **1** (1.0 equiv), methyl carbamimidate hydrochloride (1.3 equiv), and NaHCO_3_ (4.0 equiv) were dissolved in DMF (1.2 M). The mixture was stirred at 75 °C for 5 h. The solvent was removed under vacuum, and the crude mixture was purified by flash chromatography.

### General Procedure 11 (**12**)

In a RB-flask, methoxypyrimidine **11** (1.0 equiv) was dissolved 1.25 M HCl (20 equiv) in EtOH. The mixture was heated at 100 °C for 2 h under microwave irradiation. The solvent was removed under vacuum, and the crude material was purified by flash chromatography.

### General Procdure 12 (**13**)

In a RB-flask, ketoenamide **1** (1.0 equiv), 2-(4-methoxybenzyl)isothiouronium chloride (1.3 equiv), and NaHCO_3_ (4.0 equiv) were dissolved in DMF (0.8 M). The mixture was stirred at 80 °C for 3 h. An amount of 10 mL of water was then added to the mixture, which was extracted with EtOAc. Combined organic phases were washed with brine (aq solution) before being dried (MgSO_4_) and concentrated under vacuum. The crude material was purified by flash chromatography.

### General Procedure 13 (**14**)

In a RB-flask, ketoenamide **1** (1.0 equiv) and guanidine carbonate (1.1 equiv) were dissolved in DMF (0.16 M). The mixture was stirred at 80 °C for 3 h. The solvent was removed under vacuum, and the crude material was purified by flash chromatography.

### General Procedure 14 (**15**)

In a RB-flask, ketoenamide **1** (1.0 equiv), *N*-acetonylpyridinium chloride (1.0 equiv), and NH_4_OAc (3.0 equiv) were dissolved in EtOH (0.06 M). The mixture was stirred at 75 °C for 2 h. The solvent was removed under vacuum. The crude material was purified by flash chromatography.

### Transformations: 3,4-Cyclizations. General Procedure 15 (**16**)

Trimethylsulfonium iodide (1.0 equiv) and sodium hydride (1.0 equiv) were purged with nitrogen (×3) before the addition of DMSO (0.3 M). The reaction was stirred at room temperature for 1 h before the addition of ketoenamide **1** (1.0 equiv) in DMSO (0.3 M). The reaction was stirred at room temperature until complete, then quenched by the addition of saturated aqueous ammonium chloride solution. The aqueous was extracted with ethyl acetate, and the combined organic layers were washed with brine, dried (MgSO_4_), and concentrated under reduced pressure. The crude material was purified by flash column chromatography.

### General Procedure 16 (**17**)

In a RB flask, ketoenamide **1** (1.0 equiv) and Yb(OTf)_3_ (1.2 equiv) were dissolved in MeCN (0.1 M). Freshly distilled cyclopentadiene (20.0 equiv) was added. The reaction was stirred at rt for 2 h and then concentrated under vacuum. The crude material was purified by flash chromatography.

### General Procedure 17 (**19**)

In a RB flask, ketoenamide **1** (1.0 equiv), 2,3-dimethyl-1,3-butadiene (10.0 equiv), and Yb(OTf)_3_ (1.2 equiv) were dissolved in MeCN (0.6 M). The mixture was heated at 110 °C for 30 min and then concentrated under vacuum. The crude material was purified by flash chromatography.

### General Procedure 18 (**20**)

Ketoenamide **1** (1.0 equiv) and TosMIC (1.1 equiv) were purged with nitrogen (×3), dissolved in diethyl ether (0.15 M) and DMSO (0.3 M), and stirred at room temperature for 15 min. This solution was then added to a solution of sodium hydride (2.2 equiv) in diethyl ether (0.3 M), and the resulting reaction was stirred at room temperature for 30 min. The reaction was quenched with saturated aqueous ammonium chloride and extracted with ethyl acetate. The combined organic layers were washed with brine, dried (MgSO_4_), and concentrated under reduced pressure. The crude material was purified by flash column chromatography.

### General Procedure 19 (**21**)

A solution of ketoenamide **1** (1.0 equiv) and K_2_CO_3_ (2.0 equiv) was dissolved in 1 mL of H_2_O/dioxane 4:1 (0.08 M). BnN_3_ (2.0 equiv) was then added, and the mixture was stirred at 80 °C overnight under inert atmosphere. H_2_O was added to the mixture, and this was extracted with DCM. Combined organic phases were washed with saturated brine aqueous solution, dried (MgSO_4_), and concentrated under vacuum. The crude material was purified by flash chromatography.

### General Procedure 20 (**22**)

In a RB flask, ketoenamide **1** (1.0 equiv), NaN_3_ (1.0 equiv), and CuO (1.0 equiv) were dissolved in anhydrous DMF (0.2 M). The mixture was stirred at 80 °C for 4 h under inert atmosphere. Saturated NH_4_Cl aq solution was added to the mixture, which was extracted with DCM. Combined organic phases were washed with saturated brine aqueous solution, dried (MgSO_4_), and concentrated under vacuum. The crude material was purified by flash chromatography.

### General Procedure 21 (**23**)

In a RB flask, ketoenamide **1** (1.0 equiv) and *N*-methylpropan-1-imine oxide (2.2 equiv) were dissolved in DCM (0.16 M). The mixture was stirred at 60 °C for 18 h under inert atmosphere. The solvent was removed under vacuum. The crude material was purified by flash chromatography.

### Transformations: Reductions. General Procedure 22 (**24**)

In a RB-flask, ketoenamide **1** (1.0 equiv) was dissolved in MeOH (0.04 M). The mixture was passed through the H-cube (full H2, rt) for 30 min. Solvent was removed under vacuum. The crude material was purified by flash chromatography.

### General Procedure 23 (**25**)

In a RB-flask, ketoenamide **1** (1.0 equiv) and CeCl_3_·7H_2_O (1.0 equiv) were dissolved in 4 mL of DCM/MeOH 1:1 (0.1 M). The mixture was stirred at rt until complete dissolution. Then NaBH_4_ (1.0 equiv) was added portionwise and then stirred for 15 min. Saturated NH_4_Cl aq. solution was added to the mixture, which was extracted with DCM. Combined organic phases were washed with water before being dried (MgSO_4_). The solvent was removed under vacuum. The crude material was purified by flash chromatography.

### General Procedure 24 (**26**)

In a MW vial, ketoenamide **1** (1.0 equiv) was dissolved in anhydrous DCM/MeOH 1:1 (0.1 M). NaBH_4_ (3.0 equiv) was added portionwise and then stirred at rt for 1 h under inert atmosphere. NiCl_2_·6H_2_O (0.5 equiv) followed by NaBH_4_ (2.0 equiv) was added, and stirring continued at rt for 30 min. Saturated NH_4_Cl aq solution was added to the mixture, which was extracted with DCM. Combined organic phases were washed with water before being dried (MgSO_4_). The solvent was removed under vacuum. The crude mixture was purified by flash chromatography.

### Transformations: 1,4-Additions. General Procedure 25 (**27**)

In a RB-flask, ketoenamide **1** (1.0 equiv) was dissolved in anhydrous EtOH (0.32 M). Pyrrolidine (2.0 equiv) was slowly added. The mixture was stirred at rt for 5 min under inert atmosphere. The solvent was then removed under vacuum. The crude material was purified by flash chromatography.

### Compound Data. Build. (*E*)-4-(4-Methoxyphenyl)-4-oxobut-2-enoic Acid (**2b**)

Compound **10** could be obtained following general procedures 1 or 3. General procedure 1: Flash chromatography (0–10% 0.1 AcOH in MeOH in DCM) provided **2b** as a bright yellow solid (2.59 g, 12.56 mmol, 94%). General procedure 3: Reverse phase flash chromatography (10–50% MeCN/H_2_O) yielded compound **2b** as a bright yellow solid (693 mg, 3.36 mmol, 66%). *R*_*f*_ = 0.65 (80% EtOAc in PE); mp = 180–182 °C; UV λ_max_ (EtOH/nm) 287.2, 223.6, 200.0; FTIR (cm^–1^) ν 2840 (b, m, O–H acid), 1699 (s, C=O acid), 1661 (s, C=O ketone), 1592 (s, C=C alkene), 1511 (s, C=C aromatic), 1420 (s, O–H acid); ^1^H NMR (chloroform-*d*, 500 MHz) δ 3.90 (3H, s), 6.88 (1H, d, *J* = 15.5 Hz), 6.97–7.02 (2H, m), 7.97–8.05 (3H, m); ^13^C NMR (chloroform-*d*, 126 MHz) δ 55.77, 114.39, 129.6, 130.61, 131.55, 138.77, 164.57, 169.84, 187.48; MS(ES+) *m*/*z* 207.2. Data are in accordance with ref (21).

### (*E*)-4-(4-Cyanophenyl)-4-oxobut-2-enoic Acid (**2c**)

Compound **2c** was obtained following general procedure 1. Normal phase flash chromatography (0–10% 0.1% AcOH in MeOH in DCM) yielded compounds **2c** as a pale yellow solid (443 mg, 2.19 mmol, 32%). *R*_*f*_ = 0.12 (10% MeOH in DCM); mp = 134–140 °C; UV λ_max_ (EtOH/nm) 256.4; FTIR (cm^–1^) ν 3063 (b, m, O–H acid), 2231 (w, C≡N); ^1^H NMR (methanol-*d*_4_, 500 MHz) δ 6.83 (1H, d, *J* = 15.6 Hz), 7.91 (1H, d, *J* = 15.6 Hz), 7.93 (2H, d, *J* = 8.4 Hz), 8.17 (2H, d, *J* = 8.4 Hz); ^13^C NMR (methanol-*d*_4_, 126 MHz) δ 117.86, 118.87, 130.43, 133.93, 135.05, 136.84, 141.19, 168.23, 190.30; MS (ES+) *m*/*z* = 201.1 [M – H]^−^; HRMS calcd for C_11_H_7_NO_3_ 200.0348 [M + H]^+^, found 200.0363. Data in accordance with ref (21).

### (*E*)-4-Cyclohexyl-4-oxobut-2-enoic Acid (**2d**)

Compound **2d** was obtained following general procedure 2. Flash chromatography (0–15% 0.1% AcOH in MeOH in DCM) yielded **2d** as a beige solid (277 mg, 1.52 mmol, 38%). *R*_*f*_ = 0.32 (5% MeOH in DCM); mp = 113–115 °C; UV λ_max_ (EtOH/nm) 330.8, 219.8; FTIR (cm^–1^) ν 3062 (b, m, O–H acid), 2922 (s, C–H alkane), 2851 (s, C–H alkane), 1660 (s, C=O acid), 1427 (s, O–H acid); ^1^H NMR (methanol-*d*_4_, 500 MHz) δ 1.05–1.21 (5H, m), 1.51–1.67 (5H, m), 2.50 (1H, tt, *J* = 10.7, 3.4 Hz), 6.42 (1H, d, *J* = 15.9 Hz), 6.91 (1H, d, *J* = 15.9 Hz); ^13^C NMR (methanol-*d*_4_, 126 MHz) δ 26.49, 29.31, 50.35, 132.21, 139.56, 168.50, 204.38; MS(ES+) *m*/*z* 183.1 [M + H]^+^; HRMS calcd for C_10_H_14_O_3_ [M – H]^−^ 181.0870, found 181.0870. Data are in accordance with ref (21).

### (*E*)-4-Oxo-4-(tetrahydro-2*H*-pyran-4-yl)but-2-enoic Acid (**2e**)

Compound **2e** was obtained following general procedure 2. Flash chromatography (0–10% 0.1% AcOH in MeOH in DCM) yielded **2e** as an orange solid (321 mg, 1.74 mmol, 38%). *R*_*f*_ = 0.41 (5% MeOH in DCM_2_); mp = 90–92 °C; UV λ_max_ (EtOH/nm) 243.0, 201.7; FTIR (cm^–1^) ν 3067 (b, m, O–H acid), 2963 (m, C–H alkane), 2841 (m, C–H alkane), 1669 (s, C=O acid), 1424 (s, O–H acid); ^1^H NMR (methanol-*d*_4_, 500 MHz) δ 1.53 (2H, dtd, *J* = 13.6, 11.5, 4.4 Hz), 1.71 (2H, ddd, *J* = 13.3, 4.1, 2.1 Hz), 2.93 (1H, tt, *J* = 11.3, 3.8 Hz), 3.42 (2H, td, *J* = 11.6, 2.2 Hz), 3.87 (2H, ddd, *J* = 11.6, 4.3, 2.3 Hz), 6.62 (1H, d, *J* = 15.9 Hz), 7.07 (1H, d, *J* = 15.9 Hz); ^13^C NMR (methanol-*d*_4_, 126 MHz) δ 29.06, 47.01, 68.07, 132.75, 139.07, 168.44, 202.59; MS(ES+) *m*/*z* 185.1 [M + H]^+^; HRMS calcd for C_9_H_12_O_4_ [M + H]^+^ 183.0663, found 183.0644. Data are in accordance with ref (21).

### Couple. (*E*)-4-Oxo-N,4-diphenylbut-2-enamide (**1a**)

Compound **1a** could be obtained following general procedure 4 or 5. General procedure 4: Normal phase flash chromatography (0–20% EtOAc in PE) yielded **1a** as a bright yellow solid (930 mg, 3.70 mmol, quant). General procedure 5: Normal phase flash chromatography (0–20% EtOAc in PE) yielded **1a** as a bright yellow solid (830 mg, 3.30 mmol, 58%). *R*_*f*_ = 0.24 (20% EtOAc in PE); mp = 148–150 °C; UV λ_max_ (EtOH/nm) 237.2, 202.1; FTIR (cm^–1^) ν 3336 (m, N–H amide), 1650 (s, C=O amide), 1596 (s, C=O ketone); ^1^H NMR (chloroform-*d*, 500 MHz) δ 7.16 (1H, t, *J* = 7.5 Hz), 7.35 (2H, t, *J* = 7.9 Hz), 7.40 (1H, d, *J* = 14.9 Hz), 7.50 (2H, t, *J* = 7.7 Hz), 7.62 (1H, t, *J* = 7.1 Hz), 7.70 (2H, d, *J* = 8.2 Hz), 8.04 (2H, dd, *J* = 8.3, 1.1 Hz), 8.13 (1H, d, *J* = 14.9 Hz), 8.74 (1H, s); ^13^C NMR (chloroform-*d*, 126 MHz) δ 120.38, 125.17, 129.08, 129.24, 133.81, 134.19, 136.53, 136.92, 137.85, 162.33, 190.31; MS(ES+) *m*/*z* 252.2 [M + H]^+^; HRMS calcd for C_16_H_13_NO_2_ [M + Na]^+^ 274.0838, found 274.0768.

### (*E*)-*N*-(3,4-Dimethoxyphenyl)-4-(4-methoxyphenyl)-4-oxobut-2-enamide (**1b**)

Compound **1b** was obtained following general procedure 5. Normal phase flash chromatography (0–5% MeOH in DCM_2_) yielded compound **1b** as a bright yellow solid (406 mg, 1.19 mmol, 81%). *R*_*f*_ = 0.59 (5% MeOH in DCM); mp = 158–160 °C; UV λ_max_ (EtOH/nm) 322.4, 240.2, 201.8; FTIR (cm^–1^) ν 3270 (m, N–H amide), 1642 (s, C=O amide), 1599 (s, C=O ketone), 1547 (s, C=C alkene), 1510 (s, C=C aromatic); ^1^H NMR (chloroform-*d*, 500 MHz) δ 3.89 (3H, s), 3.90 (3H, s), 3.91 (3H, s), 6.85 (1H, d, *J* = 8.6, 2.5 Hz), 6.95–7.04 (3H, m), 7.15 (1H, d, *J* = 14.8 Hz), 7.53 (1H, d, *J* = 2.5 Hz), 8.03–8.14 (3H, m); ^13^C NMR (chloroform-*d*, 126 MHz) δ 55.74, 56.01, 56.22, 104.99, 111.42, 112.33, 114.34, 130.03, 131.56, 131.68, 133.58, 135.92, 146.42, 149.18, 162.32, 164.55, 188.40; MS(ES+) *m*/*z* 342.3 [M + H]^+^; HRMS calcd for C_19_H_19_NO_5_ [M + H]^+^ 342.1339, found 342.1337.

### (*E*)-4-(4-Cyanophenyl)-4-oxo-*N*-phenylbut-2-enamide (**1c**)

Compound **1c** was obtained following general procedure 5. Reverse phase flash chromatography (30–50% 0.1% HCO_2_H in MeCN in H_2_O) yielded compound **1c** as a yellow solid (858 mg, 3.11 mmol, 69%). Trituration provided the desired product pure (425 mg, 1.54 mmol, 34%). *R*_*f*_ = 0.41 (50% MeCN in H_2_O); mp = 204–206 °C ;UV λ_max_ (EtOH/nm) 250.0, 202.3; FTIR (cm^–1^) ν 3280 (m, N–H amide), 2228 (w, C≡N), 1643 (s, C=O amide), 1600 (s, C=O ketone), 1538 (s, C=C aromatic); ^1^H NMR (chloroform-*d*, 500 MHz) δ 7.05 (1H, t, *J* = 7.4 Hz), 7.16 (1H, dd, *J* = 15.0, 2.5 Hz), 7.22–7.30 (2H, m), 7.58 (2H, d, *J* = 7.9 Hz), 7.75 (2H, d, *J* = 7.9 Hz), 7.91 (1H, dd, *J* = 15.1, 2.5 Hz), 8.06 (2H, dd, *J* = 8.5, 2.5 Hz); ^13^C NMR (chloroform-*d*, 126 MHz) δ 116.67, 117.74, 120.11, 124.93, 128.94, 129.21, 132.21, 132.71, 137.72, 137.89, 139.88, 162.30, 189.02; MS(ES+) *m*/*z* 277.3 [M + H]^+^; HRMS calcd for C_17_H_12_N_2_O_2_ [M + H]^+^ 277.0972, found 277.0959.

### (*E*)-4-Cyclohexyl-4-oxo-*N*-phenylbut-2-enamide (**1d**)

Compound **1d** was obtained following general procedure 4. Reverse phase flash chromatography (50–60% MeCN in H_2_O) yielded **1d** with HPLC purity of 93% as a pale yellow solid (831 mg, 3.23 mmol, 93%). Trituration with DCM provided **39** as a white solid (559 mg, 2.17 mmol, 70%). *R*_*f*_ = 0.33 (20% EtOAc in PE); UV λ_max_ (EtOH/nm) 293.4, 227.2, 201.7; FTIR (cm^–1^) ν 3312 (m, N–H amide), 2922 (m, C–H alkane), 2851 (m, C–H alkane), 1690 (s, C=O amide), 1650 (s, C=O ketone), 1599 (s, C=C aromatic), 1532 (c, C=C aromatic); ^1^H NMR (chloroform-*d*, 500 MHz) δ 1.17–1.27 (1H, m), 1.29–1.44 (4H, m), 1.66–1.74 (1H, m), 1.82 (2H, dt, *J* = 12.3, 3.3 Hz), 1.92 (2H, d, *J* = 12.3 Hz), 2.58 (1H, tt, *J* = 10.8, 2.9 Hz), 6.97 (1H, d, *J* = 15.1 Hz), 7.16 (1H, t, *J* = 7.4 Hz), 7.32–7.40 (3H, m), 7.62 (2H, d, *J* = 7.8 Hz), 7.78 (1H, s); ^13^C NMR (chloroform-*d*, 126 MHz) δ 25.66, 25.92, 28.21, 50.78, 120.21, 125.22, 129.30, 133.83, 136.13, 137.61, 162.19, 202.81; MS(ES+) *m*/*z* 258.3; [M + H]^+^; HRMS calcd for C_16_H_19_NO_2_ [M + H]^+^ 258.1489, found 258.1492.

### (*E*)-4-Oxo-*N*-phenyl-4-(tetrahydro-*2H*-pyran-4-yl)but-2-enamide (**1e**)

Compound **1e** was obtained following general procedure 4. Reverse phase flash chromatography (30–40% MeCN in H_2_O) yielded **1e** with HPLC purity of 94% as a yellow solid (1.14 g, 4.40 mmol, 85%). Trituration with DCM provided **40** as a pale yellow solid (738 mg, 2.84 mmol, 59%). *R*_*f*_ = 0.55 (20% EtOAc in PE); UV λ_max_ (EtOH/nm) 244.2, 201.6; FTIR (cm^–1^) ν 3287 (m, N–H amide), 2948 (m, C–H alkane), 2843 (m, C–H alkane), 1694 (s, C=O amide), 1648 (s, C=O ketone), 1599 (s, C=C aromatic), 1531 (s, C=C aromatic); ^1^H NMR (methanol-*d*_4_, 500 MHz) δ 1.61 (2H, dtd, *J* = 13.6, 11.2, 4.4 Hz), 1.74 (2H, ddq, *J* = 13.6, 4.4, 2.4 Hz), 2.75 (1H, tt, *J* = 11.2, 3.8 Hz), 3.39 (2H, td, *J* = 11.6, 2.4 Hz), 3.91 (2H, ddd, *J* = 11.2, 4.4, 2.4 Hz), 6.94 (1H, d, *J* = 15.3 Hz), 7.02 (1H, tt, *J* = 7.5, 1.1 Hz), 7.19 (1H, d, *J* = 15.3 Hz), 7.21–7.25 (2H, m), 7.54 (2H, dq, *J* = 8.5, 1.8, 1.1 Hz); ^13^C NMR (methanol-*d*_4_, 126 MHz) δ 27.59, 46.96, 67.03 120.07, 124.79, 128.89, 134.37, 135.15, 137.96, 162.55, 201.43; MS(ES+) *m*/*z* 260.2; [M + H]^+^; HRMS calcd for C_15_H_17_NO_3_ [M + H]^+^ 260.1281, found 260.1282.

### (*E*)-4-(4-Methoxyphenyl)-4-oxo-*N*-phenylbut-2-enamide (**1f**)

Compound **1f** was obtained following general procedure 4. Normal phase flash chromatography (0–50% EtOAc in PE) yielded compound **1f** with some side-product as a yellow solid (295 mg, 1.05 mmol, 95%). Trituration with DCM provided **32** as a pale yellow solid (145 mg, 0.52 mmol, 47%). *R*_*f*_ = 0.40 (30% EtOAc in PE); mp = 189–191 °C; UV λ_max_ (EtOH/nm) 317.0, 267.0, 201.5; FTIR (cm^–1^) ν 3320 (m, N–H amide), 1650 (s, C=O amide), 1594 (s, C=O ketone), 1541 (s, C=C aromatic); ^1^H NMR (chloroform-*d*, 500 MHz) δ 3.88 (3H, s,), 6.95 (2H, d, *J* = 8.6 Hz), 7.15 (2H, t, *J* = 7.4 Hz), 7.34 (1H, t, *J* = 7.8 Hz), 7.42 (1H, d, *J* = 14.9 Hz), 7.71 (2H, d, *J* = 7.9 Hz), 8.05 (2H, d, *J* = 8.7 Hz), 8.13 (1H, d, *J* = 14.9 Hz), 8.97 (1H, s); ^13^C NMR (chloroform-*d*, 126 MHz) δ 55.72, 114.32, 120.37, 125.00, 129.18, 129.99, 131.61, 133.81, 135.98, 138.03, 162.59, 164.54, 188.49; MS(ES+) *m*/*z* 282.2; [M + H]^+^; HRMS calcd for C_17_H_15_NO_3_ [M + H]^+^ 282.1125, found 282.1100.

### (*E*)-1-Phenyl-4-(piperidin-1-yl)but-2-ene-1,4-dione (**1h**)

Compound **1h** was obtained following general procedure 5. Flash chromatography (0–50% EtOAc in petrol then 0–10% IPA in petrol) yielded compound **1h** as a pale yellow crystalline solid (204 mg, 0.838 mmol 39%). *R_f_* = 0.39 (10% IPA in petrol); mp 85–88 °C; UV λ_max_ (EtOH/nm) 268.4; IR ν_max_ (cm^–1^) 3018 (w, C–H), 2926 (m, C–H), 2858 (m, C–H), 1623 (s, C=O), 1605 (s, C=O); ^1^H NMR (methanol-*d*_4_, 500 MHz) δ 1.59–1.68 (4H, m), 1.68–1.75 (2H, m), 3.63 (2H, t, *J* = 5.5 Hz), 3.67 (2H, t, *J* = 5.5 Hz), 7.51–7.58 (3H, m), 7.64–7.68 (1H, m), 7.82 (1H, d, *J* = 15.2 Hz), 8.04–8.06 (2H, m); ^13^C NMR (methanol-*d*_4_, 126 MHz) δ 25.42, 26.76, 27.78, 44.57, 48.56, 129.87, 130.06, 134.54, 134.85, 134.95, 138.28, 166.08, 191.25; MS(ES^+^) *m*/*z* = 244.2 [M + H]^+^; HRMS calcd for C_15_H_17_NO_2_ [M + H]^+^ 244.1332, found 244.1475.

### (*E*)-1-Morpholino-4-phenylbut-2-ene-1,4-dione (**1i**)

Compound **1i** was obtained following general procedure 5. Flash chromatography (0–80% EtOAc in petrol), crystallization, and trituration with EtOAc yielded compound **1i** as a pale yellow-white solid (222 mg, 0.905 mmol, 36%). *R_f_* = 0.40 (80% EtOAc in petrol); mp 136–139 °C; UV λ_max_ (EtOH/nm) 270.4; IR ν_max_ (cm^–1^) 3062 (w, C–H), 2967 (m, C–H), 2929 (m, C–H), 1671 (w, C=C), 1631 (s, C=O), 1600 (s, C=O); ^1^H NMR (methanol-*d*_4_, 500 MHz) δ 3.68–3.74 (8H, m), 7.49–7.58 (3H, m), 7.67 (1H, t, *J* = 7.5 Hz), 7.88 (2H, d, *J* = 15.2 Hz), 8.06 (2H, d, *J* = 7.7 Hz); ^13^C NMR (methanol-*d*_4_, 126 MHz) δ 43.89, 47.81, 67.66, 67.90, 129.89, 130.08, 133.59, 135.00, 135.55, 138.24, 166.34, 191.18; MS(ES^+^) *m*/*z* = 246.2 [M + H]^+^; HRMS calcd for C_14_H_15_NO_3_ [M + H]^+^ 246.1125, found 246.1330.

### (*E*)-*N*-(2-Methoxyethyl)-4-oxo-4-phenylbut-2-enamide (**1j**)

Compound **1j** was obtained following general procedure 5. Flash chromatography (0–70% EtOAc in petrol), crystallization, and trituration from EtOAc yielded compound **1j** as a white solid (150 mg, 0.643 mmol 25%). *R_f_* = 0.23 (70% EtOAc in petrol); mp 108–111 °C; UV λ_max_ (EtOH/nm) 266.4; IR ν_max_ (cm^–1^) 3301 (m, N–H amide), 3067 (w, C–H), 2886 (w, C–H), 2832 (w, C–H),1634 (s, C=O), 1553 (s, C=O); ^1^H NMR (methanol-*d*_4_, 500 MHz,) δ 3.37 (3H, s), 3.48–3.54 (4H, m), 7.04 (1H, d, *J* = 15.3 Hz), 7.55 (2H, t, *J* = 7.8 Hz), 7.64–7.68 (1H, m), 7.88 (1H, d, *J* = 15.3 Hz), 8.02–8.05 (2H, m); ^13^C NMR (methanol-*d*_4_, 126 MHz) δ 40.70, 58.95, 71.76, 129.85, 130.04, 134.13, 134.92, 136.50, 138.33, 166.81, 191.53; MS(ES^+^) *m*/*z* = 234.2 [M + H]^+^; HRMS calcd for C_13_H_15_NO_3_ [M + H]^+^ 234.1125, found 234.1339.

### (*E*)-*N*-(1-Methyl-1*H*-pyrazol-3-yl)-4-oxo-4-phenylbut-2-enamide (**1k**)

Compound **1k** was obtained following general procedure 5. Trituration with DCM yielded compound **1k** as a creamy white solid (609 mg, 2.38 mmol 99%). *R_f_* = 0.23 (70% EtOAc in petrol); mp 217–221 °C; UV λ_max_ (EtOH/nm) 232.4; IR ν_max_ (cm^–1^) 3222 (m, N–H amide), 3132 (w, C–H), 3041 (w, C–H),1657 (s, C=O), 1575 (s, C=O); ^1^H NMR (DMSO-*d*_6_, 500 MHz) δ 3.77 (3H, s), 6.60 (1H, d, *J* = 2.2 Hz), 7.20 (1H, d, *J* = 15.3 Hz), 7.57–7.62 (3H, m), 7.68–7.73 (1H, m), 7.89 (1H, d, *J* = 15.3 Hz), 8.02–8.06 (2H, m), 11.09 (1H, s); ^13^C NMR (DMSO-*d*_6_, 126 MHz) δ 38.40, 96.95, 128.66, 129.00, 131.17, 132.87, 133.78, 136.00, 136.57, 146.47, 160.87, 189.64; MS(ES^+^) *m*/*z* = 256.2 [M + H]^+^; HRMS calcd for C_14_H_13_N_3_O_2_ [M + H]^+^ 256.1081 found 256.1355.

### (*E*)-*N*-Benzyl-4-oxo-4-phenylbut-2-enamide (**1l**)

Compound **1l** was obtained following general procedure 5. Flash chromatography (0–50% EtOAc in petrol), crystallization, and trituration with EtOAc yielded compound **1l** as a white solid (328 mg, 1.24 mmol, 50%). *R_f_* = 0.56 (50% EtOAc in petrol); mp 148–151 °C; UV λ_max_ (EtOH/nm) 264.2; IR ν_max_ (cm^–1^) 3288 (m, N–H amide), 1671 (m, C=C), 1633 (s, C=O), 1543 (s, C=O); ^1^H NMR (methanol-*d*_4_, 500 MHz) δ 4.51 (2H,s), 7.06 (1H, d, *J* = 15.3 Hz), 7.24–7.29 (1H, m), 7.31–7.36 (4H, m), 7.55 (2H, t, *J* = 7.8 Hz), 7.64–7.68 (1H, m), 7.92 (1H, d, *J* = 15.3 Hz), 8.02–8.05 (2H, m); ^13^C NMR (methanol-*d*_4_, 126 MHz) δ 44.60, 128.46, 128.79, 129.67, 129.86, 130.05, 134.33, 134.93, 136.45, 138.31, 139.42, 166.55, 191.47; MS(ES^+^) *m*/*z* = 266.2 [M + H]^+^ ; HRMS calcd for C_17_H_15_NO_2_ [M + H]^+^ 266.3120 found 266.1369.

### Transformations: 1,4-Cyclizations. *N*,3-Diphenyl-1*H*-pyrazole-5-carboxamide (**7a**)

Compound **7a** was obtained following general procedure 6. Flash chromatography yielded compound **7a** as a white solid (18.4 mg, 69.9 μmol, 18%). *R*_*f*_ = 0.24 (DCM); mp = 250–252 °C; UV λ_max_ (EtOH/nm) 263.0, 205.4; FTIR (cm^–1^) ν 3379 (m, N–H pyrazole), 3125 (m, N–H amide), 2923 (w, C–H), 2119 (w, C=C), 1657 (C=O amide); ^1^H NMR (DMSO-*d*_6_, 500 MHz) δ 7.11 (1H, t, *J* = 7.4 Hz), 7.38 (3H, dt, *J* = 15.7, 7.6 Hz), 7.49 (2H, t, *J* = 7.6 Hz), 7.87–7.79 (4H, m), 10.13 (1H, s); ^13^C NMR (DMSO-*d*_6_, 126 MHz) δ 103.51, 120.72, 124.08, 125.79, 128.82, 129.10, 129.48, 132.46, 139.15, 146.96, 148.06, 158.01; MS(ES+) *m*/*z* 264.2 [M + H]^+^; HRMS calcd for C_16_H_13_N_3_O [M + H]^+^ 264.1131, found 264.1132.

### *N*-(3,4-Dimethoxyphenyl)-3-(4-methoxyphenyl)-1*H*-pyrazole-5-carboxamide (**7b**)

Compound **7b** was obtained following general procedure 6. Flash chromatography (30–70% EtOAc in PE) yielded compound **7b** as a white solid (54.1 mg, 0.153 mmol, 52%). *R*_*f*_ = 0.55 (70% EtOAc in PE); mp = 225–226 °C; UV λ_max_ (EtOH/nm) = 271.0, 202.8; FTIR (cm^–1^) ν 3387 (m, N–H pyrazole), 3127 (m, N–H amide), 2835 (w, C–H), 2112 (w, C=C), 1649 (s, C=O); ^1^H NMR (DMSO-*d*_6_, 500 MHz) δ_H_ = 3.74 (3H, s), 3.76 (3H, s), 3.81 (3H, s), 6.93 (1H, d, *J* = 8.8 Hz), 7.05 (2H, d, *J* = 8.9 Hz), 7.15 (1H, s), 7.40 (1H, dd, *J* = 8.7, 2.4 Hz), 7.51 (1H, d, *J* = 2.4 Hz), 7.77 (2H, d, *J* = 8.8 Hz), 9.95 (1H, s); ^13^C NMR (DMSO-*d*_6_, 126 MHz) δ_C_ = 55.67, 55.86, 56.17, 102.67, 105.88, 112.37, 112.61, 114.86, 123.05, 127.22, 132.73, 145.53, 145.99, 146.48, 148.92, 159.66, 159.79; MS(ES+) *m*/*z* 354.4 [M + H]^+^; HRMS calcd for C_19_H_19_N_3_O_4_ [M + H]^+^ 354.1448, found 354.1461.

### *N*,3-Diphenylisoxazole-5-carboxamide (**8a**)

Compound **8a** was obtained following general procedure 7. Flash chromatography (0–40% EtOAc in PE) yielded compound **8a** as a white solid (14 mg, 0.05 mmol, 13%). *R*_*f*_ = 0.67 (60% EtOAc in PE); mp = 180–182 °C; UV λ_max_ (EtOH/nm) 268.6, 203.7; FTIR (cm^–1^) ν 3343 (m, N–H amide), 1675 (s, C=O amide), 1595 (s, C=C aromatic), 1314 (s, C–N aromatic), 1243 (s, C–O aromatic); ^1^H NMR (chloroform-*d*, 500 MHz) δ 7.06 (1H, s), 7.19 (1H, t, *J* = 7.5 Hz), 7.40 (2H, t, *J* = 7.8 Hz), 7.46–7.55 (3H, m), 7.69 (2H, d, *J* = 7.9 Hz), 7.83 (2H, dd, *J* = 7.4, 2.3 Hz), 8.58 (1H, s); ^13^C NMR (chloroform-*d*, 126 MHz) δ 99.37, 120.19, 125.18, 126.13, 126.80, 129.33, 129.36, 131.03, 137.09, 156.79, 159.56, 172.23; MS(ES+) *m*/*z* 265.2 [M + H]^+^; HRMS calcd for C_16_H_12_N_2_O_2_ [M + H]^+^ 264.0972, found 265.0901.

### *N*,5-Diphenylpyrazolo[1,5-*a*]pyrimidine-7-carboxamide (**9a**)

Compound **9a** was obtained following general procedure 8. Flash chromatography (10–50% EtOAc in PE) yielded compound **9a** as a yellowish solid (80 mg, 0.25 mmol, 64%). *R*_*f*_ = 0.63 (50% EtOAc in PE); mp = 172–174 °C; UV λ_max_ (EtOH/nm) 321.8, 272.0, 201.3; FTIR (cm^–1^) ν 3032 (m, N–H amide), 1680 (s, C=O amide), 1602 (s, C=C aromatic), 1562 (s, C=C aromatic); ^1^H NMR (chloroform-*d*, 500 MHz) δ 6.90 (1H, d, *J* = 2.5 Hz), 7.22 (1H, t, *J* = 7.5 Hz), 7.43 (2H, t, *J* = 7.8 Hz), 7.50–7.55 (3H, m), 7.85 (2H, d, *J* = 8.0 Hz), 8.17–8.23 (2H, m), 8.25 (1H, d, *J* = 2.5 Hz), 8.39 (1H, s), 12.73 (1H, s); ^13^C NMR (chloroform-*d*, 126 MHz) δ 98.10, 108.40, 121.02 (C_15_ and C_19_), 125.51, 127.59 (C_4_ and C_6_), 129.20 (C_1_ and C_3_), 129.31 (C_16_ and C_18_), 131.06, 136.68, 137.29, 137.60, 144.03, 150.32, 156.85, 156.87, 156.97 (C_7_). MS(ES+) *m*/*z* 315.2 [M + H]^+^; HRMS calcd for C_19_H_14_N_4_O [M + H]^+^ 315.1241, found 315.1127.

### *N*-(3,4-Dimethoxyphenyl)-5-(4-methoxyphenyl)pyrazolo[1,5-*a*]pyrimidine-7-carboxamide (**9b**)

Compound **9b** was obtained following general procedure 8. Flash chromatography (0–10 MeOH in EtOAc) yielded compound **9b** as a yellowish solid (69.9 mg, 0.173 mmol, 59%). *R_f_* = 0.16 (DCM); mp = 195–197 °C; UV λ_max_ (EtOH/nm) 293.8 FTIR (cm^–1^) ν 3061 (w, C–H), 2920 (w, C–H), 2843 (w, C–H), 1674 (s, C=O), 1600 (s, C=N); ^1^H NMR (chloroform-*d*, 500 MHz) δ_H_ = 3.84 (3H, s), 3.85 (3H, s), 3.91 (3H, s), 6.80 (1H, d, *J* = 2.5 Hz), 6.84 (1H, d, *J* = 8.6 Hz), 6.98 (2H, d, *J* = 8.8 Hz), 7.21 (1H, d, *J* = 2.4 Hz), 7.59 (1H, d, *J* = 2.5 Hz), 8.12 (2H, d, *J* = 8.9 Hz), 8.17 (1H, d, *J* = 2.5 Hz), 8.28 (1H, s), 12.64 (1H, s); ^13^C NMR (chloroform-*d*, 126 MHz) δ_C_ = 55.51, 56.03, 56.13, 97.49, 105.26, 107.74, 111.27, 112.97, 114.49, 129.01, 129.11, 131.08, 137.09, 143.81, 146.62, 150.16, 156.37, 156.75, 161.45, 162.10; MS(ES+) *m*/*z* 405.3; [M + H]^+^; HRMS calcd for C_22_H_20_N_4_O_4_ [M + H]^+^ 405.1557, found 405.1573

### *N*,7-Diphenylimidazo[1,2-*a*]pyridine-5-carboxamide (**10a**)

Compound **10a** was obtained following general procedure 9. Flash chromatography (10–50% EtOAc in PE) yielded compound **10a** as a yellowish solid (120 mg, 0.38 mmol, 95%). *R*_*f*_ = 0.53 (60% EtOAc in PE); mp = 115–117 °C; UV λ_max_ (EtOH/nm) 339.4, 319.2, 268.6, 201.4; FTIR (cm^–1^) ν 1675 (s, C=O amide), 1596 (s, C=C aromatic), 1520 (s, C=C aromatic); ^1^H NMR (chloroform-*d*, 500 MHz) δ 7.18 (2H, t, *J* = 7.7 Hz), 7.24 (1H, t, *J* = 7.5 Hz), 7.30 (1H, t, *J* = 7.3 Hz), 7.42 (2H, t, *J* = 7.9 Hz), 7.45 (1H, d, *J* = 1.5 Hz), 7.54 (1H, s), 7.71 (2H, d, *J* = 7.5 Hz), 7.88 (1H, d, *J* = 1.5 Hz), 7.91 (2H, d, *J* = 8.0 Hz), 11.10 (1H, s); ^13^C NMR (chloroform-*d*, 126 MHz) δ 105.52, 112.42, 121.23, 125.62, 127.10, 128.82, 129.25, 130.92, 134.90, 135.75, 137.78, 148.92, 155.94, 160.11, 161.94; MS(ES+) *m*/*z* 315.2 [M + H]^+^; HRMS calcd for C_19_H_14_N_4_O [M + H]^+^ 315.1241, found 315.1163.

### *N*-(3,4-Dimethoxyphenyl)-7-(4-methoxyphenyl)imidazo[1,2*-a*]pyrimidine-5-carboxamide (**10b**)

Compound **10b** was obtained following general procedure 9. Flash chromatography (0–10% MeOH in DCM) yielded compound **10b** as an orange solid (60.7 mg, 0.150 mmol, 51%). *R*_*f*_ = 0.39 (5% MeOH in DCM); mp = 237–240 °C; UV λ_max_ (EtOH/nm) 266.8, 200.6; FTIR (cm^–1^) ν 2931 (w, C–H), 1675 (s, C=O), 1600 (s, C=C), 1560 (s, C=O), 1510 (s, C=O); ^1^H NMR (DMSO-*d*_6_, 500 MHz) δ 3.77 (3H, s), 3.78 (3H, s), 3.86 (3H, s), 7.01 (1H, d, *J* = 8.7 Hz), 7.16 (2H, d, *J* = 9.0 Hz), 7.38 (1H, dd, *J* = 8.7, 2.4 Hz), 7.44 (1H, d, *J* = 2.4 Hz), 7.82 (1H,d, *J* = 1.5 Hz), 8.20 (1H, s), 8.34–8.26 (2H, m), 10.92 (1H, s); ^13^C NMR (DMSO-*d*_6_, 126 MHz) δ 55.93, 56.00, 56.18, 105.70, 106.21, 112.34, 113.48, 114.92, 129.34, 131.69, 136.06, 137.84, 146.54, 149.03, 149.49, 155.17, 159.82, 162.01, 162.81; MS(ES+) *m*/*z* 405.2 [M + H]^+^; HRMS calcd for C_22_H_20_N_4_O_4_ [M + H]^+^ 405.1557, found 405.1561.

### Methyl Carbamimidate Hydrochloride (**11′**)

In a RB-flask, 5 mL of anhydrous MeOH under inert atmosphere was cooled in an ice bath. Acetyl chloride (1.98 mL, 27.8 mmol) was added dropwise and then stirred at 0 °C for 20 min. A solution of cyanamide (1.00 g, 23.79 mmol) dissolved in 3 mL of anhydrous MeOH was prepared and cooled in an ice bath. To this, the methanolic hydrochloride solution was added dropwise. The mixture was warmed to rt and then stirred overnight at rt under inert atmosphere. The solvent was removed under vacuum, and the product was dried in the vacuum oven overnight. This yielded **11′** as a white solid (2.54 g, 22.98 mmol, 99%). *R*_*f*_ = 0.36 (10% MeOH in DCM); mp = 95–97 °C; UV λ_max_ (EtOH/nm) 230.0, 203.6; FTIR (cm^–1^) ν 3025 (b, s, N–H amine), 1680 (s, C=N), 1573 (s, N–H amine), 1452 (s, C–H methoxy), 1290 (s, C–N amine), 1207 (C–N); ^1^H NMR (DMSO-*d*_6_, 500 MHz) δ 3.95 (3H, s); ^13^C NMR (DMSO-*d*_6_, 126 MHz) δ 57.66, 162.67. NMR data from literature: ^1^H NMR (300 MHz, D_2_O) δ 4.04 (s, 3H); ^13^C NMR (100 MHz, D_2_O) δ 57.4, 162.9.

### 2-Methoxy-*N*,6-diphenylpyrimidine-4-carboxamide (**11a**)

Compound **11a** was obtained following general procedure 10. Flash chromatography (50–20% EtOAc in PE) yielded compound **11a** as a yellow solid (40 mg, 0.13 mmol, 25%). *R*_*f*_ = 0.27 (20% EtOAc in PE); mp = 145–146 °C; UV λ_max_ (EtOH/nm) 314.2, 240.4, 200.0; FTIR (cm^–1^) ν 3348 (m, N–H amide), 1681 (s, C=O amide), 1580 (s, C=C aromatic), 1520 (s, C=C aromatic); ^1^H NMR (chloroform-*d*, 500 MHz) δ 4.22 (3H, d, *J* = 2.1 Hz, H_23_), 7.19 (1H, t, *J* = 7.4 Hz, H_17_), 7.41 (2H, t, *J* = 7.9 Hz), 7.49–7.59 (3H, m), 7.78 (2H, d, *J* = 7.9 Hz), 8.24 (2H, dd, *J* = 7.8, 1.0 Hz), 8.32 (1H, s), 9.75 (1H, s); ^13^C NMR (chloroform-*d*, 126 MHz) δ 55.48, 108.16, 120.12, 125.07, 127.72, 129.16, 129.32, 131.99, 135.98, 137.24, 159.86, 160.62, 165.24, 169.10; MS(ES+) *m*/*z* 306.2 [M + H]^+^; HRMS calcd for C_18_H_15_N_3_O_2_ [M + H]^+^ 306.1237, found 306.1152.

### *N*-(3,4-Dimethoxyphenyl)-2-methoxy-6-(4-methoxyphenyl)pyrimidine-4-carboxamide (**11b**)

Compound **11b** was obtained following general procedure 10. Normal phase flash chromatography (30–50% EtOAc in PE) yielded compound **11b** as a yellow solid (18 mg, 0.05 mmol, 76%). *R*_*f*_ = 0.53 (60% EtOAc in PE); mp = 156–157 °C; UV λ_max_ (EtOH/nm) 328.8, 264.2, 237.2; FTIR (cm^–1^) ν 3341 (m, N–H amide), 1677 (s, C=O amide), 1581 (s, C=C aromatic), 1508 (s, C=C aromatic); ^1^H NMR (chloroform-*d*, 500 MHz) δ 3.90 (3H, s), 3.90 (3H, s), 3.95 (3H, s), 4.20 (3H, s), 6.89 (1H, d, *J* = 8.6 Hz), 7.03 (2H, dt, *J* = 9.0, 2.9, 2.0 Hz), 7.20 (1H, dd, *J* = 8.6, 2.4 Hz), 7.58 (1H, d, *J* = 2.4 Hz), 8.22 (2H, dd, *J* = 6.7, 2.0 Hz), 8.24 (1H, s), 9.69 (1H, s); ^13^C NMR (chloroform-*d*, 126 MHz) δ 55.41, 55.65, 56.13, 56.29, 104.70, 107.23, 111.60, 112.09, 114.55, 128.49, 129.46, 130.99, 146.45, 149.35, 159.58, 160.58, 162.97, 165.16, 168.57; MS(ES+) *m*/*z* 396.4 [M + H]^+^; HRMS calcd for C_21_H_21_N_3_O_5_ [M + H]^+^ 396.1557, found 396.1561.

### 2-Oxo-*N*,6-diphenyl-2,3-dihydropyrimidine-4-carboxamide (**12a**)

Compound **12a** was obtained following general procedure 11. Flash chromatography (20–60% EtOAc in PE) yielded compound **12a** as a beige solid (2.3 mg, 0.01 mmol, 20%). *R*_*f*_ = 0.67 (80% MeOH in DCM); mp = 228–230 °C, UV λ_max_ (EtOH/nm) 347.2, 308.4, 253.0, 200.0; FTIR (cm^–1^) ν 3325 (m, N–H amide), 1644 (s, C=O amide), 1600 (s, C=C aromatic), 1525 (s, C=C aromatic); ^1^H NMR (chloroform-*d*, 500 MHz) δ 7.20 (1H, tt, *J* = 7.3, 1.1 Hz), 7.41 (2H, t, *J* = 8.0 Hz), 7.66 (3H, dd, *J* = 5.2, 1.9 Hz), 7.71 (1H, s), 7.79 (2H, d, *J* = 7.9 Hz), 7.98–8.05 (2H, m), 9.84 (1H, s), 13.07 (1H, s); ^13^C NMR (chloroform-*d*, 126 MHz) δ 100.66, 120.14, 125.28, 127.59, 129.35, 130.01, 130.68, 133.25, 137.12, 159.43, 159.76, 160.45, 165.40; MS(ES+) *m*/*z* 292.2 [M + H]^+^; HRMS calcd for C_17_H_13_N_3_O_2_ [M + H]^+^ 292.1084, found 292.1202.

### *N*-(3,4-Dimethoxyphenyl)-6-(4-methoxyphenyl)-2-oxo-2,3-dihydropyrimidine-4-carboxamide (**12b**)

Compound **12b** was obtained following general procedure 11. Flash chromatography (0–10% MeOH in DCM) yielded compound **12b** as an orange solid (30.3 mg, 79.4 μmol, 26%). *R_f_* = 0.51 (10% MeOH in DCM); mp = 238–240 °C; UV λ_max_ (EtOH/nm) 352.0, 203.8; FTIR (cm^–1^) ν 2917 (m, C–H), 2847 (w, C–H), 2118 (w, C=C), 1650 (s, C=O amide), 1601 (s, C=O pyridine); ^1^H NMR (DMSO-*d*_6_, 500 MHz) δ 3.76 (3H, s), 3.77 (3H, s), 3.87 (3H, s), 6.96 (1H, d, *J* = 8.7 Hz), 7.13 (2H, d, *J* = 8.7 Hz), 7.48 (1H, dd, *J* = 8.7, 2.4 Hz), 7.57 (1H, d, *J* = 2.4 Hz), 8.10 (2H, d, *J* = 8.4 Hz), 10.40 (1H, s); ^13^C NMR (DMSO-*d*_6_, 126 MHz) δ 55.88, 55.99, 56.13, 105.83, 108.52, 112.26, 112.76, 114.88, 114.98, 129.83, 131.77, 140.42, 145.26, 146.12, 148.96, 161.13, 162.89, 181.44; MS(ES+) *m*/*z* 382.2 [M + H]^+^; HRMS calcd for C_20_H_19_N_3_O_5_ [M + H]^+^ 382.1397, found 382.1409.

### 2-(4-Methoxybenzyl)isothiouronium Chloride (**13′**)

In a RB-flask, thiourea (1.50 g, 19.7 mmol) was dissolved in 10 mL of anhydrous THF under inert atmosphere. The mixture was cooled down in an ice bath. PMBCl (2.73 mL, 19.7 mmol) was then added dropwise. The mixture was warmed to rt and stirred for 1 h. It was then stirred at 65 °C for 5 h under inert atmosphere. The mixture was filtered, and the obtained solid was washed with ether and dried overnight in the vacuum oven. This yielded **13′** as a white powder (4.41 g, 19.01 mmol, 96%). *R*_*f*_ = 0.24 (5% MeOH in DCM); mp = 168–170 °C; UV λ_max_ (EtOH/nm) 229.8, 204.8; FTIR (cm^–1^) ν 3071 (s, N–H amine), 2942 (s, N–H), 1649 (s, C=N), 1625 (s, N–H amine), 1509 (s, C–H CH_2_); ^1^H NMR (DMSO-*d*_6_, 500 MHz) δ 3.59 (3H, s), 4.34 (2H, s), 6.78 (2H, d, *J* = 8.6 Hz), 7.21 (2H, d, *J* = 8.6 Hz), 9.18 (4H, s); ^13^C NMR (DMSO-*d*_6_, 126 MHz) δ 33.91, 55.21, 114.25, 126.72, 130.43, 159.02, 169.42; MS(ES+) *m*/*z* 197.2 [M + H]^+^. NMR data from literature: ^1^H NMR (DMSO-*d*_6_, 300 MHz) δ 9.28 (s, 4H), 7.36 (d, 2H, *J* = 8.7 Hz), 6.93 (d, 2 H, *J* = 8.7 Hz), 4.47 (s, 2H), 3.75 (s, 3H).

### 2-Methoxy-*N*,6-diphenylpyrimidine-4-carboxamide (**13a**)

Compound **13a** was obtained following general procedure 12. Flash chromatography (0–20% EtOAc in PE) yielded compound **13a** as a yellow solid (30 mg, 0.07 mmol, 18%). *R*_*f*_ = 0.43 (40% EtOAc in PE); mp = 125–127 °C; UV λ_max_ (EtOH/nm) 268.8, 200.8; FTIR (cm^–1^) ν 3375 (m, N–H amide), 2927 (m, C–H alkane), 1687 (s, C=O amide), 1602 (s, C=C aromatic), 1572 (s, C=C aromatic), 1508 (s, C=C aromatic); ^1^H NMR (chloroform-*d*, 500 MHz) δ 3.79 (3H, s), 4.55 (2H, s), 6.87 (2H, d, *J* = 8.7 Hz), 7.19 (1H, t, *J* = 7.4 Hz), 7.37–7.45 (4H, m), 7.50–7.60 (3H, m), 7.73 (2H, d, *J* = 8.0 Hz), 8.22 (2H, dd, *J* = 7.6, 1.2 Hz), 8.29 (1H, s), 9.68 (1H, s); ^13^C NMR (chloroform-*d*, 126 MHz) δ 35.38, 55.44, 109.67, 114.29, 120.17, 125.12, 127.73, 129.11, 129.22, 129.32, 130.01, 131.98, 135.97, 137.18, 157.71, 159.12, 160.55, 166.71, 171.57; MS(ES+) *m*/*z* 428.3 [M + H]^+^; HRMS calcd for C_25_H_21_N_3_O_2_S [M + H]^+^ 428.1427, found 428.1429.

### 2-Amino-*N*,6-diphenylpyrimidine-4-carboxamide (**14a**)

Compound **14a** was obtained following general procedure 13. Flash chromatography (5–20% EtOAc in PE) yielded compound **14a** as a yellow solid (24 mg, 0.08 mmol, 21%). *R*_*f*_ = 0.44 (40% EtOAc in PE); mp = 127–129 °C; UV λ_max_ (EtOH/nm) 339.6, 236.0, 200.0; FTIR (cm^–1^) ν 3310 (m, N–H amine), 3185 (m, N–H amide), 1683 (s, C=O amide), 1633 (s, C=C aromatic), 1522 (s, C=C aromatic); ^1^H NMR (chloroform-*d*, 500 MHz) δ 5.26 (2H, s), 7.17 (1H, t, *J* = 7.4 Hz), 7.40 (2H, t, *J* = 7.7 Hz), 7.51 (3H, dd, *J* = 5.7, 1.8 Hz), 7.77 (2H, d, *J* = 8.0 Hz), 7.99 (1H, s), 8.09–8.15 (2H, m), 9.77 (1H, s); ^13^C NMR (chloroform-*d*, 126 MHz) δ 105.35, 119.95, 124.84, 127.51, 128.26, 129.03, 129.29, 131.38, 136.73, 137.47, 158.43, 161.23, 162.52, 168.31; MS(ES+) *m*/*z* 291.2 [M + H]^+^; HRMS calcd for C_17_H_14_N_4_O [M + H]^+^ 291.1241, found 291.1157.

### 2-Amino-*N*-(3,4-dimethoxyphenyl)-6-(4-methoxyphenyl)pyrimidine-4-carboxamide (**14b**)

Compound **14b** was obtained following general procedure 13. Flash chromatography (30–70% EtOAc in PE) yielded compound **14b** as a yellow oily film (8 mg, 0.02 mmol, 36%). *R*_*f*_ = 0.23 (40% EtOAc in PE); UV λ_max_ (EtOH/nm) 349.0, 294.4, 240.8, 206.2; FTIR (cm^–1^) ν 3292 (m, N–H amide), 1539 (s, C–N amine), 1509 (s, C=C aromatic); ^1^H NMR (chloroform-*d*, 500 MHz) δ 3.88 (3H, s), 3.90 (3H, s), 3.95 (3H, s), 5.23 (2H, s), 6.88 (1H, d, *J* = 8.6 Hz), 7.01 (2H, dt, *J* = 8.8, 2.8 Hz), 7.14 (1H, dd, *J* = 8.6, 2.5 Hz), 7.63 (1H, d, *J* = 2.5 Hz), 7.93 (1H, s), 8.11 (2H, dt, *J* = 9.0, 2.9, 1.9 Hz), 9.70 (1H, s); ^13^C NMR (chloroform-*d*, 126 MHz) δ 55.60, 56.11, 56.29, 104.49, 104.61, 111.60, 111.87, 114.43, 129.07, 129.18, 131.24, 146.29, 149.35, 158.18, 161.10, 162.38, 162.54, 167.56; MS(ES+) *m*/*z* 381.4 [M + H]^+^; HRMS calcd for C_20_H_20_N_4_O_4_ [M + H]^+^ 381.1561, found 381.1564.

### *N*-Acetonylpyridinium Chloride (**15′**)

In a RB-flask, pyridine (4.9 mL, 60.8 mmol) was added to 10 mL of anhydrous THF under inert atmosphere. Chloroacetone (5.0 mL, 73.0 mmol) was added dropwise. The mixture was stirred at rt for 24 h under an inert atmosphere. Solvent was removed under vacuum. Product was dried in vacuum oven overnight. This yielded **15′** as a white solid (8.50 g, 49.53 mmol, 97%). *R*_*f*_ = 0.36 (5% MeOH in DCM); mp = 210–212 °C; UV λ_max_ (EtOH/nm) 399.8, 260.2, 204,8; FTIR (cm^–1^) ν 2931 (m, C–H alkane), 1730 (s, C=O ketone), 1631 (s, C=C aromatic); ^1^H NMR (DMSO-*d*_6_, 500 MHz) δ 2.32 (3H, s), 5.94 (2H, d, *J* = 3.4 Hz), 8.15–8.32 (2H, m), 8.68 (1H, tt, *J* = 7.9, 1.4 Hz), 8.98 (2H, dt, *J* = 6.6, 1.9 Hz); ^13^C NMR (DMSO-*d*_6_, 126 MHz) δ 27.14, 68.16, 127.66, 145.92, 146.14, 199.44. NMR data from literature: ^1^H NMR (DMSO-*d*_6_, 400 MHz) δ ppm 2.35 (s, 3 H), 5.93 (s, 2 H), 8.25 (t, *J* = 6.95 Hz, 2 H), 8.71 (t, *J* = 7.73 Hz, 1 H), 8.99 (d, *J* = 5.87 Hz, 2 H).

### 2-Methyl-*N*,6-diphenylisonicotinamide (**15a**)

Compound **15a** was obtained following general procedure 14. Normal phase flash chromatography (10–30% EtOAc in PE) yielded compound **15a** as an orange solid (118 mg, 0.40 mmol, quant.). *R*_*f*_ = 0.60 (60% EtOAc in PE); mp = 156–158 °C; UV λ_max_ (EtOH/nm) 253.4, 211.0; FTIR (cm^–1^) ν 3240 (m, N–H amide), 1649 (s, C=O amide), 1595 (s, C=C aromatic), 1523 (s, C=C aromatic); ^1^H NMR (chloroform-*d*, 500 MHz) δ 2.68 (3H, s), 7.19 (1H, t, *J* = 7.5 Hz), 7.38 (2H, t, *J* = 7.8 Hz), 7.39–7.50 (3H, m), 7.66 (2H, d, *J* = 7.9 Hz), 7.87 (1H, s), 8.00 (2H, d, *J* = 7.2 Hz), 8.09 (1H, s); ^13^C NMR (chloroform-*d*, 126 MHz) δ 24.92, 115.13, 118.77, 120.55, 125.26, 127.22, 128.29, 129.32, 129.48, 137.53, 138.93, 143.48, 158.28, 159.80, 164.67; MS(ES+) *m*/*z* 289.2 [M + H]^+^; HRMS calcd for C_19_H_16_N_2_O [M + H]^+^ 289.13.36, found 289.1264.

### *N*-(3,4-Dimethoxyphenyl)-2-(4-methoxyphenyl)-6-methylisonicotinamide (**15b**)

Compound **15b** was obtained following general procedure 14. Flash chromatography (10–50% EtOAc in PE) yielded compound **15b** as a beige solid (13 mg, 0.03 mmol, 60%). *R*_*f*_ = 0.44 (60% EtOAc in PE); UV λ_max_ (EtOH/nm) 269.2, 205.6; FTIR (cm^–1^) ν 3338 (w, N–H amide), 2928 (m, C–H methyl), 1650 (s, C=O amide), 1604 (s, C=C aromatic), 1508 (s, C=C aromatic); ^1^H NMR (chloroform-*d*, 500 MHz) δ 2.68 (3H, s), 3.87 (3H, s), 3.89 (3H, s), 3.92 (3H, s), 6.87 (1H, d, *J* = 8.6 Hz), 7.00 (2H, dt, *J* = 8.9, 3.0, 2.0 Hz), 7.04 (1H, dd, *J* = 8.6, 2.5 Hz), 7.39 (1H, d, *J* = 1.7 Hz), 7.47 (1H, d, *J* = 2.5 Hz), 7.84–7.88 (2H, m), 8.00 (2H, dt, *J* = 8.9, 3.0, 2.0 Hz); ^13^C NMR (chloroform-*d*, 126 MHz) δ 24.98, 55.53, 56.13, 56.27, 105.38, 111.51, 112.54, 114.29, 114.35, 117.82, 128.55, 131.11, 131.56, 143.39, 146.61, 149.34, 157.95, 159.63, 160.94, 164.54; MS(ES+) *m*/*z* 379.4 [M + H]^+^; HRMS calcd for C_22_H_22_N_2_O_4_ [M + H]^+^ 379.1653, found 379.1670.

### Transformations: 3,4-Cyclizations. *trans*-2-Benzoyl-*N*-phenylcyclopropane-1-carboxamide (**16a**)

Compound **16a** was obtained following general procedure 15. Normal phase flash chromatography (0–10% EtOAc in PE) yielded compound **16a** as a white solid (27 mg, 0.10 mmol, 51%). *R*_*f*_ = 0.17 (10% EtOAc in PE); mp = 139–142 °C; UV λ_max_ (EtOH/nm) = 246.0, 201.0; FTIR (cm^–1^) ν 3288 (m, N–H amide), 1649 (s, C=O amide), 1597 (s, C=C aromatic); ^1^H NMR (chloroform-*d*, 500 MHz) δ 1.62 (1H, td, ^*3*^*J*_trans_ = 5.7, ^*2*^*J*_gem_ = 3.0 Hz), 1.76 (1H, ddd, ^*3*^*J*_cis_ = 9.0, ^*3*^*J*_trans_ = 5.7, ^*2*^*J*_gem_ = 3.0 Hz), 2.41–2.49 (1H, m), 3.33 (1H, ddd, ^*3*^*J*_cis_ = 9.0, ^*3*^*J*_trans_ = 5.7, ^*3*^*J*_trans_ = 3.7 Hz), 7.10 (1H, t, *J* = 7.5 Hz), 7.31 (2H, t, *J* = 7.9 Hz), 7.47 (2H, t, *J* = 7.8 Hz), 7.54–7.61 (3H, m, H_2_), 8.04 (2H, dd, *J* = 8.6, 0.8 Hz), 8.45–8.49 (1H, m); ^13^C NMR (chloroform-*d*, 126 MHz) δ 18.22, 26.11, 27.82, 119.99, 124.47, 128.53, 128.86, 129.12, 133.72, 137.06, 138.13, 169.16, 199.03; MS(ES+) *m*/*z* 266.2 [M + H]^+^; HRMS calcd for C_17_H_15_NO_2_ [M + H]^+^ 266.1176, found 266.1170.

### *trans*-*N*-(3,4-Dimethoxyphenyl)-2-(4-methoxybenzoyl)cyclopropane-1-carboxamide (**16b**)

Compound **16b** was obtained following general procedure 11. Normal phase flash chromatography (20–50% EtOAc in PE) yielded compound **16b** as a yellow solid (55.3 mg, 0.156 mmol, 53%). *R*_*f*_ = 0.61 (50% EtOAc in PE); mp = 120–122 °C; UV λ_max_ (EtOH/nm) = 270.6, 212.0; FTIR (cm^–1^) ν 3275 (m, N–H amide), 1646 (s, C=O amide), 1599 (s, C=O ketone), 1510 (s, C=C aromatic); ^1^H NMR (chloroform-*d*, 500 MHz) δ 1.59 (1H, ddd, ^*3*^*J*_cis_ = 8.8, ^*3*^*J*_trans_ = 5.7, ^*2*^*J*_gem_ = 3.3 Hz), 1.70 (1H, ddd, ^*3*^*J*_cis_ = 8.8, ^*3*^*J*_trans_ = 5.7, ^*2*^*J*_gem_ = 3.3 Hz), 2.33 (1H, tdd, ^*3*^*J*_cis_ = 8.9, ^*3*^*J*_trans_ = 5.7, *J* = 1.9 Hz), 3.25 (1H, ddd, ^*3*^*J*_cis_ = 9.0, ^*3*^*J*_trans_ = 5.7, ^*3*^*J*_trans_ = 3.8 Hz), 3.85 (3H, s), 3.86 (3H, s), 3.88 (3H, s), 6.80 (1H, d, *J* = 8.6 Hz), 6.89 (1H, dd, *J* = 8.6, 2.5 Hz), 6.95 (2H, dt, *J* = 8.8, 2.7, 2.2 Hz), 7.41 (1H, d, *J* = 2.5 Hz), 7.92 (1H, s), 8.03 (2H, dt, *J* = 8.9, 2.6 Hz); ^13^C NMR (chloroform-*d*, 126 MHz) δ 17.76, 25.71, 27.40, 55.68, 56.03, 56.27, 104.93, 111.52, 111.75, 114.05, 130.20, 130.86, 131.79, 146.05, 149.24, 164.09, 169.07, 196.75; MS(ES+) *m*/*z* 356.4 [M + H]^+^; HRMS calcd for C_20_H_21_NO_5_ [M + H]^+^ 356.1496, found 356.1495.

### (*rac*)-*trans*-3-Benzoyl-*N*-phenylbicyclo[2.2.1]hept-5-ene-2-carboxamide (**17a**)

Compound **17a** was obtained following general procedure 16. Normal phase flash chromatography (5–15% EtOAc in PE) yielded compound **17a** as an inseparable mixture of isomers a white solid (37 mg, 0.12 mmol, 58%). *R*_*f*_ = 0.63 (50% EtOAc in PE); UV λ_max_ (EtOH/nm) 243.4, 200.9; mp = 251–253 °C; FTIR (cm^–1^) ν 3339 (m, N–H amide), 2970 (m, C–H alkane) 1655 (s, C=O amide), 1594 (s, C=O ketone), 1532 (s, C=C aromatic); MS(ES+) *m*/*z* 318.3 [M + H]^+^; HRMS calcd for C_21_H_19_NO_2_ [M + H]^+^ 340.1308, found 340.1304.

Diastereoisomer 1, major: ^1^H NMR (chloroform-*d*, 500 MHz) δ 1.54 (1H, d, *J* = 8.4 Hz), 2.19 (1H, d, *J* = 8.4 Hz), 2.90 (1H, d, ^3^*J*_*trans*_ = 4.4 Hz), 3.16 (1H, s), 3.36 (1H, s), 4.28 (1H, t, ^3^*J*_*trans*_ = 4.4 Hz), 5.86 (1H, q, *J* = 2.8 Hz), 6.32 (1H, d, *J* = 2.8 Hz), 7.08 (1H, t, *J* = 7.3 Hz), 7.30 (2H, t, *J* = 7.9 Hz), 7.49 (4H, m), 7.57 (1H, t, *J* = 7.4 Hz), 7.97 (2H, d, *J* = 7.5 Hz); ^13^C NMR (chloroform-*d*, 126 MHz) δ 47.76, 47.83, 48.05, 48.39, 52.79, 119.66, 124.23, 128.43, 128.70, 129.02, 133.16, 134.12, 136.78, 137.12, 137.78, 172.64, 200.53.

Diastereoisomer 2, minor: ^1^H NMR (chloroform-*d*, 500 MHz) δ 1.43–1.49 (1H, m), 1.69 (1H, d, *J* = 8.7 Hz), 3.12 (1H, d, *J* = 3.1 Hz), 3.31 (1H, s), 3.68 (1H, t, ^3^*J*_*trans*_ = 4.2 Hz), 3.83 (1H, dd, ^3^*J*_*trans*_ = 4.4, *J* = 1.4 Hz), 6.24 (1H, dd, *J* = 5.6, 2.7 Hz), 6.48 (1H, dd, *J* = 5.7, 3.1 Hz), 7.07 (1H, m), 7.29 (2H, m), 7.44–7.52 (4H, m), 7.55 (1H, m), 8.05 (2H, d, *J* = 7.7 Hz).

### *N*-(3,4-Dimethoxyphenyl)-3-(4-methoxybenzoyl)bicyclo[2.2.1]hept-5-ene-2-carboxamide (**17b**)

Compound **17b** was obtained following general procedure 16. Flash chromatography (0–10% MeOH in DCM) yielded compound **17b** as gray solid (119 mg, 0.293 mmol, 100%). *R_f_* = 0.61 (4% MeOH in DCM); mp = 158–160 °C; UV λ_max_ (EtOH/nm) = 266.4, 210.0, 203.2; IR ν_max_ = 3256 (w, N–H amide), 3198 (w, C–H), 3141 (w, C–H), 2970 (w, C–H), 2841 (w, C–H), 1668 (m, C=C), 1649 (s, C=O amide), 1593 (s, C=O ketone); MS(ES+) *m*/*z* 406.2; [M + H]^+^; HRMS calcd for C_24_H_25_NO_5_ [M + H]^+^ 408.1805, found 408.1817.

Major isomer: ^1^H NMR (chloroform-*d*, 500 MHz) δ 1.48 (1H, dq, J = 8.5, 1.8 Hz), 2.15 (1H, d, *J* = 8.3 Hz), 2.86 (1H, dd, *J* = 4.7, 1.6 Hz), 3.09 (1H, d, *J* = 1.1 Hz), 3.29 (1H, p, *J* = 2.0 Hz), 3.78 (3H, s), 3.80 (3H, s), 3.81 (3H, s), 4.18 (1H, dd, *J* = 4.7, 3.4 Hz), 5.79 (1H, dd, *J* = 5.6, 2.8 Hz), 6.24 (1H, dd, *J* = 5.6, 3.1 Hz), 6.73–6.69 (1H, m), 6.79 (1H, dd, *J* = 8.6, 2.4 Hz), 6.89 (2H, d, *J* = 8.9 Hz), 7.36–7.29 (1H, m), 7.43 (1H, s), 7.93 (2H, d, *J* = 8.9 Hz); ^13^C NMR (chloroform-*d*, 126 MHz) δ 47.648.2, 48.2, 48.4, 52.3, 55.5, 55.9, 56.1, 104.5, 111.2, 111.4, 113.9, 129.6, 130.8, 131.8, 134.0, 137.8, 145.6, 149.0, 163.6, 172.7, 198.4, 1.63 (d, J = 8.6 Hz, 1H), 3.04 (s, 1H), 3.20 (s, 1H),

Minor isomer: ^1^H NMR (chloroform-*d*, 500 MHz) δ 1.40 (1H, dd, *J* = 8.6, 1.5 Hz), 3.57 (1H, dd, *J* = 4.7, 3.5 Hz), 3.65 (1H, dd, *J* = 4.8, 1.2 Hz), 6.20 (1H, dd, *J* = 5.6, 2.8 Hz), 6.44 (1H, dd, *J* = 5.6, 3.1 Hz), 6.74–6.69 (1H, m), 6.88 (2H, d, *J* = 9.1 Hz), 7.36–7.30 (1H, m), 7.97 (2H, d, *J* = 8.9 Hz); ^13^C NMR (chloroform-*d*, 126 MHz) δ 47.0, 47.6, 48.4, 49.1, 50.6, 55.5, 55.9, 56.1, 104.5, 111.2, 111.4, 113.9, 129.6, 131.0, 131.8, 135.1, 137.1, 145.6, 149.0, 163.6, 172.7, 198.4.

### 2-(3,4-Dimethoxyphenyl)-3-(4-methoxyphenyl)-5,6-dimethyl-2,3,4,7-tetrahydro-1*H*-isoindol-1-one (**18b**)

Compound **18b** was obtained following general procedure 17. Flash chromatography (10–30% EtOAc in PE) yielded compound **18b** as gray solid (20.9 mg, 49.3 μmol, 17%). *R*_*f*_ = 0.46 (50% EtOAc in PE); mp = 77–79 °C; UV λ_max_ (EtOH/nm) 266.6; FTIR (cm^–1^) ν 3317 (br, N–H), 2910 (w, C–H), 2839 (w, C–H), 1662 (m, C=C), 1600 (s, C=O), 1512 (s, C=O); ^1^H NMR (chloroform-*d*, 500 MHz) δ 1.65 (3H, s), 1.71 (3H, s), 2.12 (1H, t, *J* = 14.7 Hz), 2.27 (1H, s), 2.30 (1H, s), 2.59 (1H, t, *J* = 14.7 Hz), 2.97 (1H, td, *J* = 11.3, 5.4 Hz), 3.82 (3H, s), 3.84 (3H, s), 3.88 (3H, s), 3.93 (1H, td, *J* = 11.5, 5.6 Hz), 6.75 (1H, d, *J* = 8.6 Hz), 6.82 (1H, dd, *J* = 8.6, 2.4 Hz), 6.94 (2H, d, *J* = 9.0 Hz), 7.23 (1H, d, *J* = 2.4 Hz), 7.53 (1H, s), 8.00 (1H, d, *J* = 9.0 Hz); ^13^C NMR (chloroform-*d*, 126 MHz) δ 18.64, 18.72, 34.90, 36.25, 44.41, 44.80, 55.49, 55.83, 56.11, 104.88, 111.25, 111.78, 113.83, 123.90, 124.51, 129.24, 130.97, 131.65, 145.66, 148.91, 163.76, 173.13, 202.24; MS(ES+) *m*/*z* 424.4 [M + H]^+^; HRMS calcd for C_25_H_29_NO_5_ [M + H]^+^ 424.2118, found 424.2102.

### 5,6-Dimethyl-2,3-diphenyl-2,3,4,7-tetrahydro-1*H*-isoindol-1-one (**19a**)

Compound **19a** was obtained following general procedure 17. Normal phase flash chromatography (0–10% MeOH in DCM_2_) yielded compound **19a** as white crystals (34 mg, 0.11 mmol, 41%). *R*_*f*_ = 0.37 (10% MeOH in DCM); mp = 229–231 °C; UV λ_max_ (EtOH/nm) = 200.3; FTIR (cm^–1^) ν 2855 (w, C–H alkane), 1672 (s, C=O amide), 1596 (s, C=C aromatic), 1494 (s, C=C aromatic), 1452 (m, C–H methyl), 1358 (s, C–N amide), 1121 (s, C–N amide); ^1^H NMR (chloroform-*d*, 500 MHz) δ 1.65 (3H, s), 1.75 (3H, s), 2.45 (1H, dt, *J* = 22.6, 7.9 Hz), 2.83 (1H, dt, *J* = 22.6, 7.9 Hz), 2.96 (2H, d, *J* = 7.9 Hz), 5.45 (1H, s), 7.02 (1H, t, *J* = 7.4 Hz), 7.18 (2H, d, *J* = 7.5 Hz), 7.21–7.29 (3H, m), 7.31 (2H, t, *J* = 7.4 Hz), 7.55 (2H, d, *J* = 8.1 Hz); ^13^C NMR (chloroform-*d*, 126 MHz) δ 18.79, 18.87, 28.86, 31.33, 67.64, 120.99, 121.81, 123.55, 124.06, 126.87, 128.40, 128.87, 129.12, 129.22, 136.18, 138.13, 152.29, 170.70; MS(ES+) *m*/*z* 316.3 [M + H]^+^; HRMS calcd for C_22_H_21_NO [M + H]^+^ 316.1696, found 316.1683.

### 4-Benzoyl-*N*-phenyl-1*H*-pyrrole-3-carboxamide (**20a**)

Compound **20a** was obtained following general procedure 18. Normal phase flash chromatography (20–80% EtOAC in PE) yielded compound **20a** as a beige crystalline solid (75 mg, 0.26 mmol, 65%). *R*_*f*_ = 0.35 (60% EtOAc in PE); mp = 157–159 °C; UV λ_max_ (EtOH/nm) 247.2, 202.3; FTIR (cm^–1^) ν 3116 (m, N–H amide), 2940 (m, N–H pyrrole), 1611 (s, C=C amide), 1557 (s, C=C ketone), 1500 (s, C=C aromatic), 1378 (s, C–N aromatic), 1317 (s, C–N amide), 1167 (s, C–N amide), 1087 (s, C=C aromatic); ^1^H NMR (chloroform-*d*, 500 MHz) δ 6.91 (1H, t, *J* = 7.4 Hz), 7.06 (1H, d, *J* = 2.2 Hz), 7.16 (2H, t, *J* = 7.8 Hz), 7.31 (2H, t, *J* = 7.6 Hz), 7.41 (1H, t, *J* = 7.4 Hz), 7.53 (1H, d, *J* = 2.2 Hz), 7.54–7.58 (2H, m), 7.60 (2H, d, *J* = 8.0 Hz); ^13^C NMR (chloroform-*d*, 126 MHz) δ 119.91, 120.20, 120.88, 123.77, 128.10, 128.63, 128.86, 131.75, 133.24, 138.48, 139.85, 162.45, 194.58; MS(ES+) *m*/*z* 291.2 [M + H]^+^; HRMS calcd for C_18_H_14_N_2_O_2_ [M + H]^+^ 291.1128, found 291.1132.

### *N*-(3,4-Dimethoxyphenyl)-4-(4-methoxybenzoyl)-1*H*-pyrrole-3-carboxamide (**20b**)

Compound **20b** was obtained following general procedure 18. Flash chromatography (0–10% MeOH in DCM) yielded compound **20b** as a yellow solid (82.2 mg, 0.216 mmol, 75%). *R_f_* = 0.47 (10% MeOH in DCM); mp = 191–193 °C; UV λ_max_ (EtOH/nm) 271.2, 202.8; FTIR (cm^–1^) ν 3264 (s, N–H amide), 3067 (w, N–H pyrrole), 3004 (w, C–H), 2950 (w, C–H), 1661 (m, C=O, amide), 1592 (s, C=O ketone), 1562 (s, C=N); ^1^H NMR (chloroform-*d*, 500 MHz) δ 3.87 (3H, s), 3.89 (3H, s), 3.90 (3H, s), 6.83 (1H, d, *J* = 8.7 Hz), 6.96 (2H, d, *J* = 8.8 Hz), 7.21 (1H, t, *J* = 2.6 Hz), 7.28 (1H, dd, *J* = 8.7, 2.4 Hz), 7.54 (1H, d, *J* = 2.4 Hz), 7.83–7.75 (3H, m), 11.20 (1H, s), 12.40 (s, 1H); ^13^C NMR (chloroform-*d*, 126 MHz) δ 55.5, 55.9, 56.1, 105.4, 111.4, 112.8, 113.7, 120.4, 128.2, 131.7, 132.1, 145.7, 149.0, 162.6, 163.1, 193.1; MS(ES+) *m*/*z* 381.2 [M + H]^+^; HRMS calcd for C_21_H_20_N_2_O_5_ [M + H]^+^ 381.1445, found 381.1429.

### 5-Benzoyl-1-benzyl-*N*-phenyl-1*H*-1,2,3-triazole-4-carboxamide (**21a**)

Compound **21a** was obtained following general procedure 19. Normal phase flash chromatography (0–10% EtOAc in PE) yielded compound **21a** as white crystals (5 mg, 0.01 mmol, 16%). *R*_*f*_ = 0.27 (10% EtOAc in PE); mp = 160–162 °C; UV λ_max_ (EtOH/nm) v 258.8, 200.5; FTIR (cm^–1^) ν 3028 (m, N–H amide), 2923 (m, C–H alkane), 1680 (s, C=O amide), 1622 (s, C=O ketone), 1596 (s, C=C aromatic), 1564 (s, C=C aromatic); ^1^H NMR (chloroform-*d*, 500 MHz) δ 6.21 (2H, s), 7.19 (1H, tt, *J* = 7.4, 1.1 Hz), 7.29–7.37 (3H, m), 7.40 (2H, tt, *J* = 8.5, 7.4, 1.9 Hz), 7.45–7.51 (2H, m), 7.54 (2H, t, *J* = 8.0 Hz), 7.67 (1H, tt, *J* = 8.0, 1.1 Hz_2_), 7.78 (2H, dd, *J* = 8.2, 1.0 Hz), 8.21 (2H, dd, *J* = 8.5, 1.1 Hz), 12.05 (1H, s); ^13^C NMR (chloroform-*d*, 126 MHz) δ 54.46, 120.82, 125.38, 128.55, 128.72, 128.80, 128.97, 129.28, 131.65, 134.22, 134.31, 135.13, 136.69, 137.56, 142.96, 154.75, 190.66; MS(ES+) *m*/*z* 383.3 [M + H]^+^; HRMS calcd for C_23_H_18_N_4_O_2_ [M + H]^+^ 383.1503, found 383.1484.

### 1-Benzyl-*N*-(3,4-dimethoxyphenyl)-4-(4-methoxybenzoyl)-1*H*-1,2,3-triazole-5-carboxamide (**21b**).

Compound **21b** was obtained following general procedure 19. Flash chromatography (0–10% MeOH in DCM_2_) yielded compound **21b** as a brown solid (26.1 mg, 55.2 μmol, 19%). *R_f_* = 0.24 (DCM); mp = 121–122 °C; UV λ_max_ (EtOH/nm) v 303.2, 202.4; FTIR (cm^–1^) ν 2924 (m, C–H), 2852 (m, C–H), 1674 (s, C=O amide), 1594 (s, C=O ketone), 1564 (s, C=C aromatic); ^1^H NMR (DMSO-*d*_6_, 500 MHz) δ 3.82 (3H, s), 3.85 (3H, s), 3.88 (3H, s), 6.13 (2H, s), 6.80 (1H, d, *J* = 8.7 Hz), 6.94 (1H, d, *J* = 9.0 Hz), 7.21 (1H, dd, *J* = 8.6, 2.5 Hz), 7.30–7.24 (3H, m), 7.34 (1H, d, *J* = 2.4 Hz), 7.41 (2H, dd, *J* = 8.0, 1.6 Hz), 8.21 (1H, d, *J* = 9.0 Hz), 11.98 (1H, s); ^13^C NMR (chloroform-*d*, 126 MHz) δ 54.17, 55.66, 56.07, 56.11, 105.12, 111.30, 112.92, 113.81, 128.52, 128.65, 128.80, 129.23, 130.99, 133.94, 134.28, 135.14, 143.13, 146.50, 149.12, 154.57, 164.68, 188.33; MS(ES+) *m*/*z* 473.3 [M + H]^+^; HRMS calcd for C_26_H_24_N_4_O_5_ [M + H]^+^ 473.1819 found 473.1806.

### 4-Benzoyl-*N*-phenyl-1*H*-1,2,3-triazole-5-carboxamide (**22a**)

Compound **22a** was obtained following general procedure 20. Normal phase flash chromatography (0–10% MeOH in EtOAc) yielded **22a** as a beige solid (21 mg, 0.07 mmol, 36%). *R*_*f*_ = 0.18 (5% MeOH in DCM); mp = 201–203 °C; UV λ_max_ (EtOH/nm) 252.4, 200.6; FTIR (cm^–1^) ν 3395 (b, m, N–H amide), 2923 (m, N–H triazole), 1620 (s, C=O amide), 1595 (s, C=C aromatic), 1568 (s, C=C aromatic), 1397 (s, C–N aromatic); ^1^H NMR (methanol-*d*_4_, 500 MHz) δ 7.09 (1H, t, *J* = 7.4 Hz), 7.32 (2H, t, *J* = 7.8 Hz), 7.49 (2H, t, *J* = 7.6 Hz), 7.61 (1H, t, *J* = 7.4 Hz), 7.76 (2H, d, *J* = 7.9 Hz), 8.11 (2H, d, *J* = 7.7 Hz); ^13^C NMR (methanol-*d*_4_, 126 MHz) δ 121.43, 125.52, 129.22, 130.00, 131.89, 134.21, 139.46, 139.63, 142.90, 144.15, 161.30, 192.72; MS(ES+) *m*/*z* 293.2 [M – H]^+^; HRMS calcd for C_16_H_12_N_4_O_2_ [M + H]^+^ 293.1033, found 293.1024.

### *N*-(3,4-Dimethoxyphenyl)-4-(4-methoxybenzoyl)-1*H*-1,2,3-triazole-5-carboxamide (**22b**)

Compound **22b** was obtained following general procedure 20. Normal phase flash chromatography (0–10% MeOH in EtOAc) yielded **22b** as a beige solid (23.0 mg, 60.2 μmol, 21%). *R*_*f*_ = 0.18 (10% MeOHc in DCM); mp = 216–218 °C; UV λ_max_ (EtOH/nm) 300.8, 200.2 FTIR (cm^–1^) 3127 (w, N–H), 2918 (w, C–H), 2849 (w, C–H), 1628 (s, C=O), 1574 (s, C=O), 1511 (s, C=C); ^1^H NMR (DMSO-*d*_*6*_, 500 MHz) δ 3.74 (6H, s), 3.87 (3H, s), 6.93 (1H, d, *J* = 8.7 Hz), 7.11 (2H, d, *J* = 8.5 Hz), 7.25 (1H, d, *J* = 8.8 Hz), 7.43 (1H, s), 8.06 (2H, s), 10.86 (1H, s); ^13^C NMR (DMSO-*d*_6_, 126 MHz) δ 55.89, 56.13, 56.17, 105.40, 112.39, 112.46, 114.38, 129.78, 132.32, 133.12, 141.87, 145.86, 145.88, 149.02, 157.83, 164.20, 186.73; MS(ES+) *m*/*z* 383.2 [M + H]^+^; HRMS calcd for C_19_H_18_N_4_O_5_ [M + H]^+^ 383.1350, found 383.1363.

### (*Z*)-*N*-Methylpropan-1-imine Oxide (**23′**)

In a RB flask, *N*-hydroxylamine (835 mg, 10.0 mmol) and NaOMe (540 mg, 10.0 mmol) were dissolved in 15 mL of anhydrous EtOH. The mixture was stirred at rt under inert atmosphere for 10 min before propanal (725 μL, 10.0 mmol) was added dropwise. The mixture was stirred for 1 h at rt under inert atmosphere. The solvent was removed under vacuum. The crude was dissolved in DCM and filtered. The solvent of the filtrate was removed under vacuum. This was purified by normal flash chromatography (0–5% MeOH in DCM) and yielded **23′** as a transparent oil (195 mg, 2.24 mmol, 22%). *R*_*f*_ = 0.12 (5% MeOH in DCM); UV λ_max_ (EtOH/nm) 233.8; FTIR (cm^–1^) ν 2967 (m, C–H alkane), 2877 (m, C–H alkane), 1659 (s, C=N imine), 1602 (s, N–O), 1458 (s, C–H CH_2_), 1398 (s, C–H methyl), 1187 (s, C–N); ^1^H NMR (chloroform-*d*, 500 MHz) δ 1.03 (3H, t, *J* = 7.7 Hz), 2.42 (2H, p, *J* = 7.1 Hz), 3.61 (3H, s), 6.61 (1H, t, *J* = 5.7 Hz); ^13^C NMR (chloroform-*d*, 126 MHz) δ 9.82, 20.28, 52.25, 141.89.

### (3*S*,4*R*,5*R*)/(3*R*,4*S*,5*S*)-5-Benzoyl-3-ethyl-2-methyl-*N*-phenylisoxazolidine-4-carboxamide (**23a-A**)

Compound **23a** was obtained following general procedure 21. Normal phase flash chromatography (0–10% EtOAc in PE) yielded compounds **23a-A**, **23a-C**, and **23a-D** as a mixture. Semipreparative HPLC yielded **23a-A** as transparent film (16 mg, 0.05 mmol, 16%). *R*_*f*_ = 0.15 (10% EtOAc in PE); ^1^H NMR (chloroform-*d*, 500 MHz) δ 1.02 (3H, t, *J* = 7.5 Hz), 1.64 (2H, dq, *J* = 14.8, 7.4 Hz), 2.86 (4H, s), 3.77 (1H, dd, ^3^*J*_*cis*_ = 5.9, ^3^*J*_*trans*_ = 2.5 Hz), 5.57 (1H, d, ^3^*J*_*trans*_ = 2.6 Hz), 7.13 (1H, t, *J* = 7.4 Hz), 7.35 (2H, t, *J* = 7.7 Hz), 7.50 (2H, t, *J* = 7.7 Hz), 7.60 (3H, m, H_2_), 8.03 (2H, d, *J* = 7.7 Hz), 9.11 (1H, s); ^13^C NMR (chloroform-*d*, 126 MHz) δ 11.55, 21.05, 44.05, 56.98, 70.98, 80.68, 120.14, 124.53, 129.01, 129.18, 129.28, 134.16, 134.22, 137.91, 168.75, 195.22 ; MS(ES+) *m*/*z* 339.3 [M + H]^+^.

### (3*R*,4*R*,5*R*)/(3*S*,4*S*,5*S*)-4-Benzoyl-3-ethyl-2-methyl-*N*-phenylisoxazolidine-5-carboxamide (**23a-B**)

Compound **23a-B** was obtained following the above procedure for **23a-A**. Normal phase flash chromatography (0–10% EtOAc in PE) yielded compound **23a-B** as a white solid (21 mg, 0.06 mmol, 16%). *R*_*f*_ = 0.20 (10% EtOAc in PE); ^1^H NMR (chloroform-*d*, 500 MHz) δ 0.84 (3H, t, *J* = 7.5 Hz), 1.51 (1H, dq, *J* = 14.6, 7.5 Hz), 1.61 (1H, dtd, *J* = 14.6, 7.5, 4.6 Hz), 2.88 (3H, s), 3.28 (1H, q, ^3^*J*_*trans*_ = 7.7, 4.6 Hz), 4.53 (1H, dd, ^3^*J*_*trans*_ = 6.7, ^3^*J*_*trans*_ = 3.3 Hz), 4.61 (1H, d, ^3^*J*_*trans*_ = 3.2 Hz), 7.14 (1H, t, *J* = 7.4 Hz), 7.36 (2H, t, *J* = 7.9 Hz), 7.52 (2H, t, *J* = 7.6 Hz), 7.56–7.64 (3H, m, H_2_), 8.26 (2H, d, *J* = 7.1 Hz), 8.72 (1H, s); ^13^C NMR (chloroform-*d*, 126 MHz) δ 10.48, 24.82, 44.17, 59.07, 72.48, 79.54, 120.01, 124.71, 128.98, 129.19, 129.37, 133.84, 136.10, 137.33, 170.26, 198.01; MS(ES+) *m*/*z* 339.3 [M + H]^+^.

### (3*R*,4*R*,5*R*)/(3*S*,4*S*,5*S*)-5-Benzoyl-3-ethyl-2-methyl-*N*-phenylisoxazolidine-4-carboxamide (**23a-C**)

Compound **23a-C** was obtained following the above procedure for **23a-A**. Normal phase flash chromatography (0–10% EtOAc in PE) yielded compounds **23a-A**, **23a-C**, and **23a-D** as a mixture. Semipreparative HPLC yielded **23a-C** as transparent film (9 mg, 0.03 mmol, 15%). *R*_*f*_ = 0.15 (10% EtOAc in PE); ^1^H NMR (500 MHz, chloroform-*d*) δ 0.85 (3H, t, *J* = 7.3 Hz), 1.10 (1H, s), 1.47 (1H, ddq, *J* = 14.5, 9.7, 7.3 Hz), 2.89 (3H, s), 3.37 (1H, d, *J* = 15.2 Hz), 4.99 (1H, s), 5.36 (1H, d, ^3^*J*_*trans*_ = 7.0 Hz), 7.13 (1H, t, *J* = 7.4 Hz), 7.34 (2H, t, *J* = 7.8 Hz), 7.51 (2H, t, *J* = 7.6 Hz), 7.55 (2H, d, *J* = 8.0 Hz), 7.62 (1H, t, *J* = 7.4 Hz), 8.05 (2H, d, *J* = 7.6 Hz), 8.27 (1H, s); ^13^C NMR (126 MHz, chloroform-*d*) δ 11.48, 22.90, 46.42, 55.72, 72.73, 79.95, 119.70, 124.85, 128.52, 129.09, 129.26, 133.90, 136.92, 137.19, 170.14, 196.22; MS(ES+) *m*/*z* 339.3 [M + H]^+^.

### (3*S*,4*R*,5*R*)/(3*R*,4*S*,5*S*)-4-Benzoyl-3-ethyl-2-methyl-*N*-phenylisoxazolidine-5-carboxamide (**23a-D**)

Compound **23a-D** was obtained following the above procedure for **23a-A**. Normal phase flash chromatography (0–10% EtOAc in PE) yielded compounds **23a-A**, **23a-C**, and **23a-D** as a mixture. Semipreparative HPLC yielded **23a-D** as transparent film (22 mg, 0.07 mmol, 33%). *R*_*f*_ = 0.15 (10% EtOAc in PE); mp = 96–98 °C; UV λ_max_ (EtOH/nm) 245.5, 202.0; FTIR (cm^–1^) ν 3056 (w, b, N–H amide), 1661 (s, C=O amide), 1592 (s, C=O ketone), 1523 (s, C=C aromatic); ^1^H NMR (chloroform-*d*, 500 MHz) δ 1.00 (3H, t, *J* = 7.5 Hz), 1.56 (1H, dt, *J* = 14.7, 7.4 Hz), 1.69 (1H, dt, *J* = 14.4, 7.3 Hz), 2.83 (3H, s), 3.29 (1H, q, ^3^*J*_*cis*_ = 7.1 Hz), 3.70 (1H, t, ^3^*J*_*cis*_ = 7.0 Hz), 5.28 (1H, d, ^3^*J*_*trans*_ = 6.5 Hz), 7.11 (1H, t, *J* = 7.4 Hz), 7.32 (2H, t, *J* = 7.8 Hz), 7.47 (2H, t, *J* = 7.7 Hz), 7.55 (2H, d, *J* = 8.0 Hz), 7.60 (1H, t, *J* = 7.4 Hz), 8.11 (2H, d, *J* = 8.2 Hz); ^13^C NMR (chloroform-*d*, 126 MHz) δ 10.65, 25.68, 44.25, 57.71, 72.27, 82.53, 119.85 (C_15_ and C_19_), 124.64, 128.65, 129.17, 130.12, 134.19, 134.79, 137.83, 169.12, 199.49; MS(ES+) *m*/*z* 339.3 [M + H]^+^; HRMS calcd for C_20_H_22_N_2_O_6_ [M + H]^+^ 339.1703, found 339.1692.

### (3S,4*R*,5*R*)-*N*-(3,4-Dimethoxyphenyl)-3-ethyl-5-(4-methoxybenzoyl)-2-methylisoxazolidine-4-carboxamide (**23b-A**)

Compound **23b** was obtained following general procedure 21. Normal phase flash chromatography (0–10% EtOAc in PE) yielded compound **23b-A** as a white solid (41.9 mg, 97.9 μmol, 13%). *R*_*f*_ = 0.42 (50% EtOAc in PE); mp = 141–142 °C; UV λ_max_ (EtOH/nm) 272.8; FTIR (cm^–1^) ν 3330 (m, N–H), 2932 (m, C–H), 2836 (m, C–H), 1667 (s, C=O), 1597 (s, C=O), 1510 (s, C=C); ^1^H NMR (chloroform-*d*, 500 MHz) δ 0.78 (3H, t, *J* = 7.4 Hz), 1.40–1.50 (1H, m), 1.51–1.61 (1H, m), 2.82 (3H, s), 3.22 (1H, s), 3.80 (3H, s), 3.80 (3H, s), 3.84 (3H, s), 4.40 (1H, dd, *J*_*trans,cis*_ = 6.7, 3.5 Hz), 4.52 (1H, d, *J*_*cis*_ = 3.3 Hz), 6.75 (1H, d, *J* = 8.6 Hz), 6.91 (2H, d, *J* = 8.9 Hz), 7.30 (1H, d, *J* = 2.4 Hz), 8.18 (2H, d, *J* = 8.9 Hz), 8.58 (1H, s); ^13^C NMR (chloroform-*d*, 126 MHz) δ 10.44, 24.73, 44.10, 55.54, 56.01, 56.15, 58.57, 72.43, 104.69, 111.33, 111.91, 114.05, 128.86, 130.86, 131.68, 146.03, 149.12, 164.06, 169.79, 196.08; MS(ES+) *m*/*z* 429.3 [M + H]^+^; HRMS calcd for C_23_H_28_N_2_O_6_ [M + H]^+^ 429.2020, found 429.22041.

### (3*R*,4*R*,5*R*)-*N*-(3,4-Dimethoxyphenyl)-3-ethyl-4-(4-methoxybenzoyl)-2-methylisoxazolidine-5-carboxamide (**23b-B**)

Compound **23b-B** was obtained following the above procedure for **23b-A**. Normal phase flash chromatography (0–10% EtOAc in PE) yielded compounds **23b-B**, **23b-C**, and **23b-D** as a mixture. Semipreparative HPLC yielded **23b-B** as transparent film (35.5 mg, 82.5 μmol, 11%). *R*_*f*_ = 0.23 (50% EtOAc in PE); mp = 119–120 °C; UV λ_max_ (EtOH/nm) 282.2, 271.2, 211.4; FTIR (cm^–1^) ν 3296 (m, N–H), 2933 (w, C–H), 2836 (w, C–H), 1654 (s, C=O), 1598 (s, C=O), 1511 (s, C=C); ^1^H NMR (chloroform-*d*, 500 MHz) δ 0.94 (3H, t, *J* = 7.4 Hz), 1.47–1.59 (1H, m), 1.64 (1H, q, *J* = 7.0 Hz), 2.79 (3H, s), 3.27 (1H, d, *J*_*trans*_ = 7.4 Hz), 3.67 (1H, t, *J*_*trans*_ = 7.1 Hz), 3.78 (3H, s), 3.80 (3H, s), 3.81 (3H, s), 5.20 (1H, d, *J*_*trans*_ = 6.8 Hz), 6.72 (1H, d, *J* = 8.6 Hz), 6.83–6.92 (3H, m), 7.31 (1H, d, *J* = 2.4 Hz), 8.04 (2H, d, *J* = 8.9 Hz), 8.17 (1H, s); ^13^C NMR (chloroform-*d*, 126 MHz) δ 10.60, 14.21, 25.49, 44.12, 55.55, 55.92, 56.12, 57.32, 72.17, 82.56, 104.46, 111.24, 111.54, 113.81, 127.62, 131.48, 132.53, 145.86, 149.00, 164.35, 168.57, 196.92; MS(ES+) *m*/*z* 429.3 [M + H]^+^; HRMS calcd for C_23_H_28_N_2_O_6_ [M + H]^+^ 429.2020, found 429.22041.

### (3*R*,4*R*,5*R*)-*N*-(3,4-Dimethoxyphenyl)-3-ethyl-5-(4-methoxybenzoyl)-2-methylisoxazolidine-4-carboxamide (**23b-C**)

Compound **23b-C** was obtained following the above procedure for **23b-A**. Normal phase flash chromatography (0–10% EtOAc in PE) yielded compounds **23b-B**, **23b-C**, and **23b-D** as a mixture. Semipreparative HPLC yielded **23b-C** as transparent film (26.9 mg, 62.9 μmol, 9%). *R*_*f*_ = 0.23 (50% EtOAc in PE); UV λ_max_ (EtOH/nm) 273.4, 211.2, 200.6; FTIR (cm^–1^) ν 3326 (m, N–H), 2933 (m, C–H), 2836 (w, C–H), 1665 (s, C=O), 1597 (s, C=O), 1510 (s, C=C); ^1^H NMR (chloroform-*d*, 500 MHz) δ 0.80 (3H, t, *J* = 5.3 Hz), 0.98–1.10 (1H, m), 1.39 (1H, s), 2.85 (3H, s), 3.28 (1H, s), 3.79 (3H, s), 3.80 (3H, s), 3.82 (3H, s), 4.86 (1H, t, *J*_*trans*_ = 6.9 Hz), 5.26 (1H, br, s), 6.74 (1H, d, *J* = 8.5 Hz), 6.78–6.86 (1H, m), 6.91 (3H, d, *J* = 8.5 Hz), 7.41 (1H, s), 7.91–8.02 (2H, m), 8.18 (1H, d, *J* = 15.0 Hz); ^13^C NMR (chloroform-*d*, 126 MHz) δ 11.39, 22.62, 46.36, 55.16, 55.58, 55.95, 56.13, 58.46, 72.65, 79.88, 104.39, 111.30, 111.37, 114.14, 129.61, 130.83, 131.34, 146.06, 149.12, 164.11, 169.82, 193.35; MS(ES+) *m*/*z* 429.4 [M + H]^+^; HRMS calcd for C_23_H_28_N_2_O_6_ [M + H]^+^ 429.2020, found 429.1998.

### (3*S*,4*R*,5*R*)-*N*-(3,4-Dimethoxyphenyl)-3-ethyl-4-(4-methoxybenzoyl)-2-methylisoxazolidine-5-carboxamide (**23b-D**)

Compound **23b-D** was obtained following the above procedure for **23b-A**. Normal phase flash chromatography (0–10% EtOAc in PE) yielded compounds **23b-B**, **23b-C**, and **23b-D** as a mixture. Semipreparative HPLC yielded **23b-D** as transparent film (22.2 mg, 51.9 μmol, 7%). *R*_*f*_ = 0.23 (50% EtOAc in PE); UV λ_max_ (EtOH/nm) 282.2; FTIR (cm^–1^) ν 3295 (br, N–H), 2934 (w, C–H), 1655 (s, C=O), 1597 (s, C=O), 1510 (S, C=C); ^1^H NMR (chloroform-*d*, 500 MHz) δ 0.97 (3H, t, *J* = 7.4 Hz), 1.60 (2H, d, *J* = 9.0 Hz), 2.80 (3H, s), 2.88 (1H, s), 3.79 (7H, d, *J* = 3.0 Hz), 3.82 (3H, s), 5.51 (1H, s), 6.74 (1H, d, *J* = 8.6 Hz), 6.85–6.91 (2H, m), 6.91–6.96 (1H, m), 7.31 (1H, d, *J* = 2.2 Hz), 7.95 (2H, d, *J* = 8.3 Hz), 9.01 (1H, s); ^13^C NMR (chloroform-*d*, 126 MHz) δ 11.46, 21.07, 44.13, 55.57, 55.90, 55.99, 56.16, 56.45, 71.19, 80.79, 104.84, 111.33, 111.91, 114.10, 127.13, 131.37, 131.51, 131.62, 145.86, 149.05, 164.25, 168.24, 193.4; MS(ES+) *m*/*z* 429.3 [M + H]^+^; HRMS calcd for C_23_H_28_N_2_O_6_ [M + H]^+^ 429.2020, found 429.22041.

### Transformations: Reductions. 4-Oxo-*N*,4-diphenylbutanamide (**24a**)

Compound **24a** was obtained following general procedure 22. Flash chromatography (0–20% EtOAc in PE) yielded compound **24a** as a beige solid (88 mg, 0.35 mmol, 87%). *R*_*f*_ = 0.34 (20% EtOAc in PE); UV λ_max_ (EtOH/nm) 243.2, 204.3; FTIR (cm^–1^) ν 3309 (m, N–H amide), 1699 (s, C=O amide), 1657 (s, C=O ketone), 1595 (s, C=C aromatic), 1531 (s, C=C aromatic), 1442 (s, C–H CH_2_); ^1^H NMR (chloroform-*d*, 500 MHz) δ 2.82 (2H, t, *J* = 6.4 Hz), 3.46 (2H, t, *J* = 6.4 Hz), 7.08 (1H, t, *J* = 7.7 Hz), 7.30 (2H, t, *J* = 8.2, 7.7 Hz), 7.47 (2H, t, *J* = 7.8 Hz), 7.52 (2H, d, *J* = 7.7 Hz), 7.58 (1H, t, *J* = 7.7 Hz), 7.87 (1H, s), 8.00 (2H, d, *J* = 7.7 Hz); ^13^C NMR (chloroform-*d*, 126 MHz) δ 31.57, 34.26, 119.91, 124.29, 128.27, 128.81, 129.07, 133.60, 136.52, 138.10, 170.59, 199.41; MS(ES−) *m*/*z* 252.1 [M – H]^−^; HRMS calcd for C_16_H_15_NO_2_ [M + Na]^+^ 276.0995, found 276.0919.

### *N*-(3,4-Dimethoxyphenyl)-4-(4-methoxyphenyl)-4-oxobutanamide (**24b**)

Compound **24b** was obtained following general procedure 22. Flash chromatography (20–100% EtOAc in PE) yielded compound **24b** as a white solid (10 mg, 0.03 mmol, 47%). *R*_*f*_ = 0.24 (5% MeOH in DCM), mp = 148–150 °C; UV λ_max_ (EtOH/nm) 265.4, 211.4; FTIR (cm^–1^) ν 3287 (m, N–H amide), 2908 (m, C–H alkane), 2836 (m, C–H alkane), 1670 (s, C=O amide), 1649 (s, C=O ketone) 1596 (s, C=C aromatic), 1507 (s, C=C aromatic); ^1^H NMR (chloroform-*d*, 500 MHz) δ 2.78 (2H, t, *J* = 6.4 Hz), 3.41 (2H, t, *J* = 6.4 Hz), 3.84 (3H, s), 3.86 (3H, s), 3.87 (3H, s), 6.77 (1H, d, *J* = 8.6 Hz), 6.88 (1H, dd, *J* = 8.6, 2.4 Hz), 6.94 (2H, dt, *J* = 5.0, 2.8 Hz), 7.34 (1H, d, *J* = 2.4 Hz), 7.79 (1H, s), 7.98 (2H, dt, *J* = 4.9, 2.8 Hz); ^13^C NMR (chloroform-*d*, 126 MHz) δ 31.68, 33.97, 55.64, 56.04, 56.27, 104.97, 111.50, 111.79, 113.97, 129.65, 130.57, 131.90, 145.85, 149.17, 163.95, 170.59, 197.88; MS(ES+) *m*/*z* 344.3 [M + H]^+^; HRMS calcd for C_19_H_21_NO_5_ [M + H]^+^ 344.1496, found 344.1504.

### (*E*)-4-Hydroxy-*N,*4-diphenylbut-2-enamide (**25a**)

Compound **25a** was obtained following general procedure 23. Compound **25a** was yielded as a white solid (101 mg, 0.40 mmol, 100%). *R*_*f*_ = 0.18 (20% EtOAc in PE); UV λ_max_ (EtOH/nm) v 270.8, 201.9; FTIR (cm^–1^) 3314 (m, N–H amide), 3255 (m, O–H alcohol) 2921 (m, C–H alkane), 1672 (s, C=O amide), 1598 (s, C=C aromatic); ^1^H NMR (chloroform-*d*, 500 MHz) δ 5.78 (1H, d, *J* = 4.9 Hz), 6.77 (1H, dd, *J* = 15.2, 1.8 Hz), 7.45 (1H, dd, *J* = 15.2, 4.9 Hz), 7.51 (1H, t, *J* = 7.5 Hz), 7.71 (3H, m), 7.75–7.83 (4H, m), 8.01 (2H, d, *J* = 8.1 Hz); ^13^C NMR (chloroform-*d*, 126 MHz) δ 72.89, 119.92, 122.39, 124.00, 126.37, 127.61, 128.30, 128.51, 138.11, 141.34, 145.91, 164.80; MS(ES+) *m*/*z* 254.2 [M + H]^+^; HRMS calcd for C_16_H_15_NO_2_ [M + H]^+^ 254.1179, found 254.1175.

### (*E*)-*N*-(3,4-Dimethoxyphenyl)-4-hydroxy-4-(4-methoxyphenyl)but-2-enamide (**25b**)

Compound **25b** was obtained following general procedure 23. Flash chromatography (50–70% EtOAc in PE) afforded **25b** as a white solid (14.6 mg, 0.04 mmol, 62%). *R*_*f*_ = 0.18 (60% EtOAc in PE); mp = 151–154 °C; UV λ_max_ (EtOH/nm) v 300.4, 205.8; FTIR (cm^–1^) ν 3254 (b, m, O–H alcohol), 2837 (m, C–H alkane), 1670 (s, C=O amide), 1605 (s, C=C aromatic), 1510 (s, C=C aromatic); ^1^H NMR (methanol-*d*_4_, 500 MHz) δ 3.79 (3H, s), 3.81 (3H, s), 3.82 (3H, s), 5.31 (1H, dd, *J* = 5.0, 1.7 Hz), 5.49 (1H, s), 6.34 (1H, dd, *J* = 15.2 Hz, 1.7 Hz), 6.87–6.94 (3H, m), 6.99 (1H, dd, *J* = 15.2, 5.0 Hz), 7.09 (1H, dd, *J* = 8.7 Hz, *J* = 2.4 Hz), 7.30 (2H, d, *J* = 8.7 Hz), 7.39 (1H, d, *J* = 2.4 Hz); ^13^C NMR (methanol-*d*_4_, 126 MHz) δ 55.72, 56.40, 56.77, 73.90, 106.64, 113.31, 113.67, 114.98, 123.56, 129.09, 133.66, 135.40, 147.37, 147.72, 150.45, 160.90, 166.27; MS(ES+) *m*/*z* 344.2 [M + H]^+^; HRMS calcd for C_19_H_22_NO_5_ [M + H]^+^ 344.1492, found 344.1495.

### 4-Hydroxy-*N*,4-diphenylbutanamide (**26a**)

Compound **26a** was obtained following general procedure 24. Normal phase flash chromatography (10–40% EtOAc in PE) yielded **26a** as a yellow oil (96 mg, 0.38 mmol, 94%). *R*_*f*_ = 0.12 (30% EtOAc in PE); mp = 145–147 °C; UV λ_max_ (EtOH/nm) 242.6, 202.7; FTIR (cm^–1^) ν 3291 (b, m, O–H alcohol), 3075 (m, N–H amide), 1666 (s, C=O amide), 1596 (s, C=C aromatic); ^1^H NMR (chloroform-*d*, 500 MHz) δ 1.99–2.17 (2H, m), 2.40–2.53 (2H, m), 4.75 (1H, dd, *J* = 8.1, 4.3 Hz), 7.09 (1H, t, *J* = 7.4 Hz), 7.26 (3H, m), 7.32 (4H, d, *J* = 4.4 Hz), 7.49 (2H, d, *J* = 7.9 Hz), 8.24 (1H, s); ^13^C NMR (chloroform-*d*, 126 MHz) δ 33.94, 34.35, 73.59, 120.20, 124.40, 125.81, 127.56, 128.51, 128.99, 137.96, 144.21, 172.31; MS(ES+) *m*/*z* 254.2 [M - H]^+^; HRMS calcd for C_16_H_17_NO_2_ [M + Na]^+^ 278.1151, found 278.1149.

### *N*-(3,4-dDimethoxyphenyl)-4-hydroxy-4-(4-methoxyphenyl)butanamide (**26b**)

Compound **26b** was obtained following general procedure 24. Normal phase flash chromatography (35–55% EtOAc in PE) yielded compound **26b** as a white solid (26 mg, 0.08 mmol, 85%). *R*_*f*_ = 0.19 (50% EtOAc in petrol ether); mp = 147–150 °C; UV λ_max_ (EtOH/nm) v 254.0, 209.0; FTIR (cm^–1^) ν 3395 (b, m, O–H alcohol), 3287 (m, N–H amide), 2929 (m, C–H alkane), 2834 (m, C–H alkane), 1658 (s, C=O amide), 1605 (s, C=C aromatic), 1510 (s, C=C aromatic); ^1^H NMR (chloroform-*d*, 500 MHz) δ 2.09–2.18 (2H, m), 2.44–2.52 (2H, m), 3.80 (3H, s), 3.85 (3H, s), 3.87 (3H, s), 4.78 (1H, dd, *J* = 7.4, 5.2 Hz), 7.43 (1H, s), 6.76–6.85 (2H, m), 6.86–6.90 (2H, m), 7.27–7.31 (2H, m), 7.33 (1H, d, *J* = 2.4 Hz); ^13^C NMR (chloroform-*d*, 126 MHz) δ 34.11, 34.32, 55.45, 56.08, 56.29, 73.42, 105.17, 111.50, 111.95, 114.05, 127.13, 131.61, 136.45, 146.06, 149.24, 159.25, 171.55; MS(ES−) *m*/*z* 344.1 [M – H]^−^.

### Transformations: 1,4-Additions. 4-Oxo-*N*,4-diphenyl-2-(pyrrolidin-1-yl)butanamide (**27a**)

Compound **27a** was obtained following general procedure 25. Amine-functionalized silica flash chromatography (20–50% EtOAc in PE) yielded compound **27a** (40 mg, 0.12 mmol, 70%) as a yellow oil. *R*_*f*_ = 0.28 (50% EtOAc in PE); UV λ_max_ (EtOH/nm) 240.8, 200.0; FTIR (cm^–1^) ν 3312 (w, N–H amide), 2961 (w, C–H alkane), 1674 (s, C=O amide), 1596 (s, C=C ketone), 1515 (s, C=C aromatic); ^1^H NMR (chloroform-*d*, 500 MHz) δ 1.83 (4H, h, *J* = 6.3 Hz), 2.71 (4H, dtd, *J* = 14.1, 8.6, 4.1 Hz), 3.06 (1H, dd, *J* = 16.8, 5.3 Hz), 3.68–3.77 (1H, m), 4.35 (1H, dd, *J* = 6.7, 5.2 Hz), 7.08 (1H, t, *J* = 7.4 Hz), 7.30 (2H, t, *J* = 7.9 Hz), 7.47 (2H, t, *J* = 7.7 Hz), 7.52–7.59 (3H, m), 7.99–8.05 (2H, m), 9.22 (1H, s); ^13^C NMR (chloroform-*d*, 126 MHz) δ 23.88, 34.63, 49.91, 61.50, 119.57, 124.14, 128.37, 128.72, 129.05, 133.22, 137.08, 137.91, 170.98, 198.45; MS(ES+) *m*/*z* 323.3 [M – H]^+^; HRMS calcd for C_20_H_22_N_2_O_2_ [M + H]^+^ 323.1757, found 323.1677.

### *N*-(3,4-Dimethoxyphenyl)-4-(4-methoxyphenyl)-4-oxo-2-(pyrrolidin-1-yl)butanamide (**27b**)

Compound **27b** was obtained following general procedure 25. Amine-functionalized silica flash chromatography (30% DCM in PE) yielded compound **27b** as a yellow solid (117 mg, 0.283 mmol, 97%). *R*_*f*_ = 0.32 (60% DCM in PE, amine-functinalised silica); UV λ_max_ (EtOH/nm) 267.6; FTIR (cm^–1^) ν 3316 (br, N–H), 2933 (w, C–H), 2833 (w, C–H), 1668 (s, C=O), 1597 (s, C=O), 1509 (s, C=C); ^1^H NMR (chloroform-*d*, 500 MHz) δ 1.68–1.80 (5H, m), 2.57–2.71 (4H, m), 3.00 (1H, dd, *J* = 16.8, 5.4 Hz), 3.59 (1H, dd, *J* = 16.8, 6.5 Hz), 3.77 (3H, s), 3.78 (3H, s), 3.79 (3H, s), 4.21 (1H, dd, *J* = 6.4, 5.3 Hz), 6.71 (1H, d, *J* = 8.6 Hz), 6.81 (1H, dd, *J* = 8.6, 2.5 Hz), 6.86 (2H, d, *J* = 8.9 Hz), 7.33 (1H, d, *J* = 2.4 Hz), 7.93 (2H, d, *J* = 8.9 Hz), 9.07 (1H, s); ^13^C NMR (chloroform-*d*, 126 MHz) δ 23.76, 34.62, 49.90, 55.49, 55.93, 56.12, 61.40, 104.28, 111.21, 111.26, 113.76, 129.88, 130.56, 131.67, 145.55, 149.02, 163.56, 170.78, 196.75; MS(ES+) *m*/*z* 413.3 [M + H]^+^; HRMS calcd for C_28_H_28_N_2_O_5_ [M + H]^+^ 413.2071, found 413.2086.

## Supporting Information Available

Summary of reaction optimization results, chemical procedures, analytical data, HPLC chromatograms, and NMR spectra; computational analysis of molecular diversity of the pooled library; biological methods for protein expression; SPR and X-ray crystallography for CDK2 (PDF)
Molecular formula strings with associated biological data and calculated properties (CSV)

## Accession Codes

PDB code 7ZPC is compound **9k** in complex with CDK2. Authors will release the atomic coordinates upon article publication.

## Abbreviations Used

ATP: adenosine triphosphate
CDK2: cyclin-dependent kinase 2
DOS: diversity-oriented synthesis
HATU: hexafluorophosphate azabenzotriazole tetramethyl uronium
H-bond: hydrogen bond
HPLC: high-performance liquid chromatography
HRMS: high resolution mass spectrometry
HTS: high-throughput screening
PCA: principal component analysis
PMB: *para*-methoxybenzyl
PMI: principle moments of inertia
SPR: surface plasmon resonance
