# Supplementary material for: Build–Couple–Transform: A Paradigm for Lead-like Library Synthesis with Scaffold Diversity
Source: J Med Chem. 2022 Aug 9;65(16):11322–39. doi: 10.1021/acs.jmedchem.2c00897 (PMC9421646; doi:10.1021/acs.jmedchem.2c00897)
Supplement: Supplementary file 1 — jm2c00897_si_001.pdf [file jm2c00897_si_001.pdf]

Supporting Information

Build-Couple-Transform: a paradigm for lead-like library synthesis with scaffold diversity

Mélanie Uguen,[a] Gemma Davison,[a] Lukas J. Sprenger,[a] James H. Hunter,[a] Mathew P. Martin,[b] Shannon Turberville,[b] Jessica E. Watt,[b] Bernard T. Golding,[c] Martin E. M. Noble,[b] Hannah L. Stewart*[a] and Michael J. Waring*[a]

[a] M. Uguen, G. Davison, L. J. Sprenger, J. H. Hunter, H. L. Stewart, M. J. Waring,* Cancer Research UK Newcastle Drug Discovery Unit, Newcastle University Centre for Cancer, Chemistry, School of Natural and Environmental Sciences, Newcastle University, Bedson Building, Newcastle upon Tyne NE1 7RU, U.K,*

[b] M. P. Martin, S. Tuberville, J. E. Watt, M. E. M. Noble, Cancer Research UK Newcastle Drug Discovery Unit, Newcastle University Centre for Cancer, Translational and Clinical Research Institute, Faculty of Medical Sciences, Newcastle University, Paul O'Gorman Building, Newcastle upon Tyne NE2 4HH, U.K.

[c] B. T. Golding, Chemistry, School of Natural and Environmental Sciences, Newcastle University, Bedson Building, Newcastle upon Tyne NE1 7RU, U.K.

** hannah.stewart@newcastle.ac.uk; mike.waring@newcastle.ac.uk*

1. Optimisation Tables 4

1.1 Optimisation of Pyrazole Transformation 4

1.2 Optimisation of Isoxazole Transformation 4

1.3 Optimisation of Pyrimidone and Mercaptopyrimidine 5

1.4 Optimisation of 2-aminopyrmidine 6

1.5 Optimisation of Diels Alder Transformations 6

1.6 Optimisation of Triazole 8

2. Chemical Procedures 9

2.1. General Information 9

2.2. Analytical Equipment 9

2.3. General Procedures 9

2.3.1. Build 9

2.3.2. Couple 9

2.3.3. Transformations: 1,4-Cyclisations 10

2.3.4. Transformations: 3,4-Cyclisations 10

2.3.5. Transformations: Reductions 11

2.3.6. Transformations: 1,4-Additions 11

2.4. Compound Data 12

2.4.1. Build 12

2.4.2. Couple 14

2.4.3. Transformations: 1,4-Cyclisations 18

2.4.4. Transformations: 3,4-Cyclisations 24

2.4.5. Transformations: Reductions 32

2.4.6. Transformations: 1,4-Additions 34

2.5. NMR spectra 35

2.5.1. Build 35

2.5.3. Transformations: 1,4-Cyclisations 50

2.5.4. Transformations: 3,4-Cyclisations 70

2.5.5. Transformations: Reductions 92

3. Scope Evaluation of the DOS Transformations 100

3.1 Analysis of Starting Materials 100

3.1.1. HPLC Analyses of Starting Materials of Set 1 Before Being Pooled 100

3.1.2. HPLC Analyses of Set 1 After Being Pooled....104
3.1.3. HPLC Analyses of Starting Materials of Set 2 Before Being Pooled....105
3.2. HRMS and HPLC Analysis of DOS transformation on Pools 1 & 2 110
3.2.1. Transformations: 1,4-Cyclisations:....110
3.2.2. Transformations: 3,4-Cycloadditions 134
3.2.3. Transformations: Reductions....155
3.2.4. Transformations: 1,4-Additions....164
4. Computational Analysis 167
4.1 General details 167
4.2 Compound collections analysed 167
3.2.1. Transformations: 1,4-Cyclisations:....168
3.2.2. Transformations: 3,4-Cycloadditions 173
3.2.3. Transformations: Reductions 182
3.2.4. Transformations: 1,4-Additions....183
5. Materials and Methods for Biological Evaluation 184
5.1 Expression and purification of CDK2. 184
5.2 Surface Plasmon resonance Screening of DOS library against CDK2. 184
5.3 Crystalisation and ligand soaking of CDK2. 184
6. References....185

1. Optimisation Tables

1.1 Optimisation of Pyrazole Transformation

Table S1. *Optimisation of the transformation to pyrazole.*

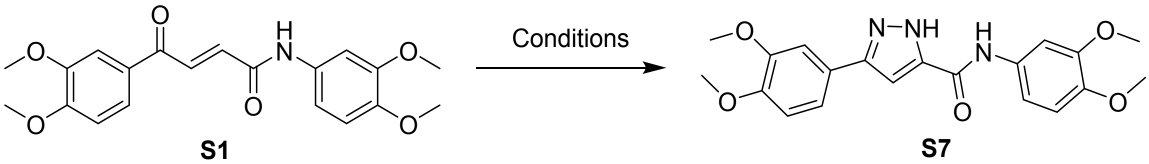

Entry	Conditions	Yield after purification
1	$NH_2NH_2{\cdot}H_2O$ (2 eq.), EtOH, reflux, 18 h	36%
2	$TsNHNH_2$ (1.2 eq), K_2CO_3 (1.5 eq.), EtOH, 75 °C, 3 h	18%
3	$TsNHNH_2$ (1.1 eq), NaOAc (1.1 eq.), EtOH, 80 °C, 3 h	None
4	TsNHNH2 (1.2 eq), K_2CO_3 (1.5 eq.), I_2 (2 mol-%), EtOH, 75 °C, 3 h	33%
5	$TsNHNH_2$ (2 eq), I_2 (2 eq.), EtOH, reflux, 2 h	None
6	$TsNHNH_2$ (1.1 eq), EtOH, reflux, 3 h then NaOH (1.1 eq.), reflux, 4 h	Good conversion
7	1) $NH_2NH_2{\cdot}H_2O$ (2 eq.), EtOH, reflux, 2 h<br>2) MnO_2 (12 eq.), DCM, reflux, 2 h	94%

1.2 Optimisation of Isoxazole Transformation

Table S2. *Optimisation of the transformation to isoxazole.*

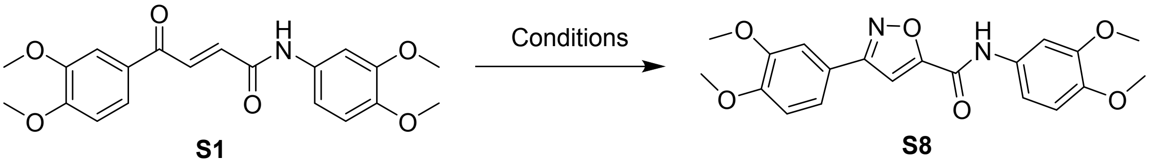

Entry	Conditions	Yield after purification
1	TsNHOH (1.5 eq.), K_2CO_3, MeOH/H_2O (6:1), 60 °C, o/n.	23%
2	$NH_2OH{\cdot}HCl$ (2 eq.), NaOH (4 eq.), EtOH, reflux, 6 h.	None
3	$NH_2OH{\cdot}HCl$ (2 eq.), NaOH (4 eq.), DMSO, 100 °C, 6 h.	24%
4	$NH_2OH{\cdot}HCl$ (2 eq.), NaOAc (4 eq.), DMSO, 60 °C, 3 h.	63%
5	1) $NH_2OH{\cdot}HCl$ (2 eq.), NaOAc (4 eq.), DMSO, 60 °C, 3 h.<br>2) MnO_2 (10 eq.), 60 °C, 2 h.	75%

1.3 Optimisation of Pyrimidone and Mercaptopyrimidine

Table S3. *Optimisation of the transformation to pyrimidone and mercaptopyrimidine.*

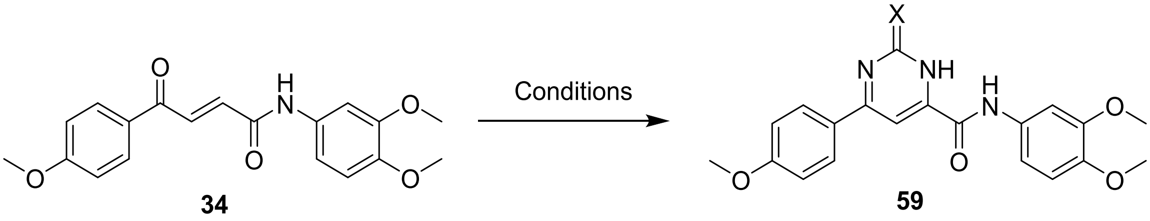

Entry	X	Conditions	Yield after purification
1	O	Urea (1.1 eq.), NaOH 2 M aq. solution (3.1 eq.), EtOH, 60 °C, 6 h.	None
2	O	Urea (3 eq.), 40% KOH methanolic solution (3 eq.), reflux, 6 h.	None
3	O	Urea (10 eq.), HCl 4 M in dioxane (3 eq.), EtOH, reflux, 6 h.	None
4	S	Thiourea (1.1 eq.), NaOH 2 M aq. solution (3.1 eq.), EtOH, 60 °C, 6 h.	None

Table S4. *Optimisation of the transformation to pyrimidine and 2-methoxypyrimidine.*

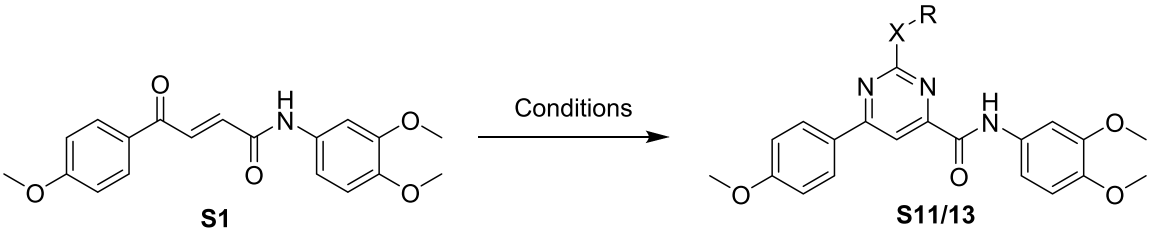

Entry	X	R	Conditions	Yield after purification
1	O	Me	Methyl carbamimidate (2 eq.), NaOAc (4 eq.), DMSO, 60 °C, 6 h.	Not isolated
2	O	Me	Methyl carbamimidate (1.3 eq.), NaHCO3 (4 eq.), DMF, 75 °C, 5 h.	76%
1	S	PMB	2-PMB-isothiouronium chloride (2 eq.), NaOAc (4 eq.), DMSO, 60°C, 6h.	None
2	S	PMB	2-PMB-isothiouronium chloride (1 eq.), NaOAc (1 eq.), DMF, 75°C, 5h.	None
3	S	PMB	2-PMB-isothiouronium chloride (1.3 eq.), $NaHCO_3$ (4 eq.), DMF, 75°C, 6h.	Traces

1.4 Optimisation of 2-aminopyrmidine

Table S5. *Optimisation of the transformation to 2-aminopyrimidine.*

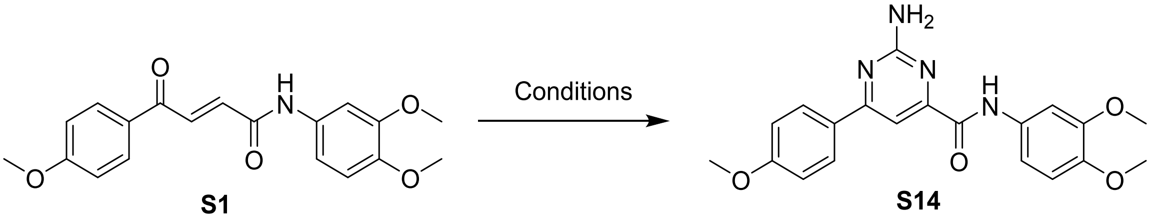

Entry	Conditions	Yield after purification
1	Guanidine HCl (3 eq.), NaOH 2 M aq. (4 eq.), EtOH, 65 °C, o/n.	25%
2	Guanidine HCl (1.1 eq.), NaOH 2 M aq. (3.1 eq.), EtOH, 110 °C, 15 min, MW.	None
3	Guanidine carbonate (1.1 eq.), NaOH 2 M aq. (3.1 eq.), EtOH, 80 °C, 3 h.	None
4	Guanidine carbonate (1.1 eq.), NaOAc (3.1 eq.), DMSO, 60 °C, 3 h.	Not isolated
5	Guanidine carbonate (1.1 eq.), DMSO, 60 °C, 3 h.	36%

1.5 Optimisation of Diels Alder Transformations

Table S61. *Optimisation of the Diels Alder transformation.*

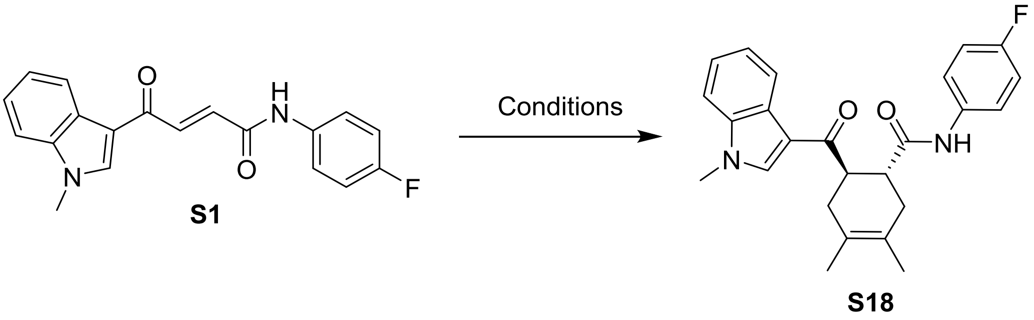

Entry	Conditions	Yield after purification
1	2,3-dimethyl-1,3-butadiene (2 eq.), toluene/DCM 10:1, rt, 48 h.	No product
2	2,3-dimethyl-1,3-butadiene (2 eq.), $Yb(OTf)_3$ (1.2 eq.), MeCN, 85 °C, 4 h.	No product
3	2,3-dimethyl-1,3-butadiene (2 eq.), $Yb(OTf)_3$ (1.2 eq.), MeCN, 30 °C, 24 h.	Low conversion
4	2,3-dimethyl-1,3-butadiene (2 eq.), $Yb(OTf)_3$ (1.2 eq.), MeCN, MW, 50 °C, 2.5 h.	41%
5	2,3-dimethyl-1,3-butadiene (2 eq.), $Yb(OTf)_3$ (1.2 eq.), MeCN, MW, 80 °C, 4.5 h.	48%

Table S72. *Optimisation of the Diels Alder transformation.*

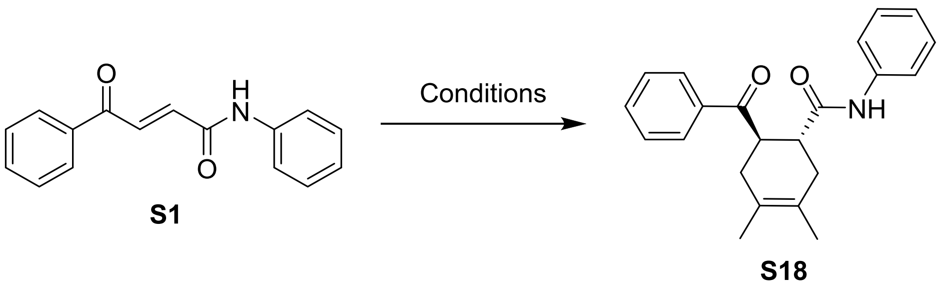

Entry	Conditions	Yield after purification
1	2,3-dimethyl-1,3-butadiene (2 eq.), $Yb(OTf)_3$ (1.2 eq.), MeCN, MW, 80 °C, 4.5 h.	No product
2	2,3-dimethyl-1,3-butadiene (2 eq.), $Yb(OTf)_3$ (1.2 eq.), MeCN, MW, 110 °C, 0.5 h.	No product
3	2,3-dimethyl-1,3-butadiene (2 eq.), SPGS-550-M 2% in water, 60 °C, 6 h.	No conversion

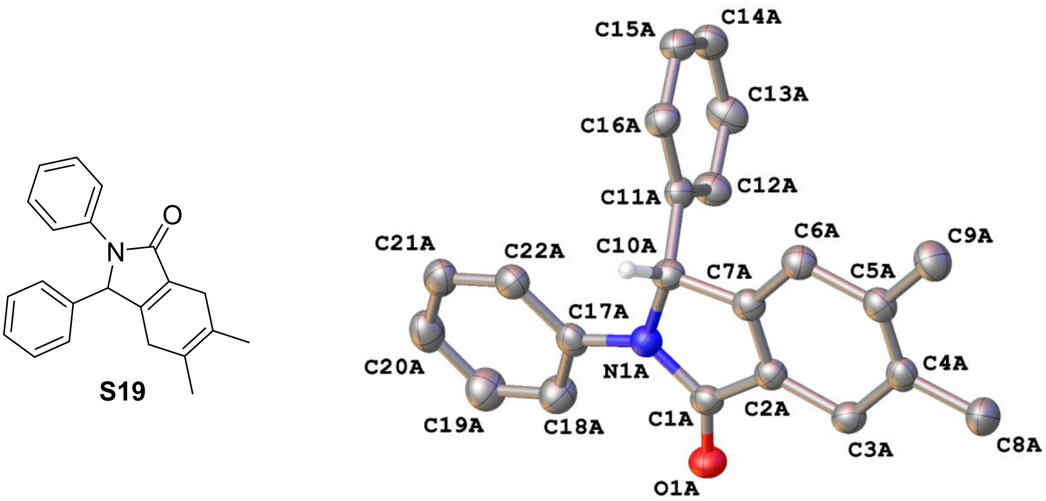

Figure S1. *Structure and small molecule X-ray crystallography structure of side-product* ***S19****.*

1.6 Optimisation of Triazole

Table S8. *Conditions used for the transformation to triazole.*

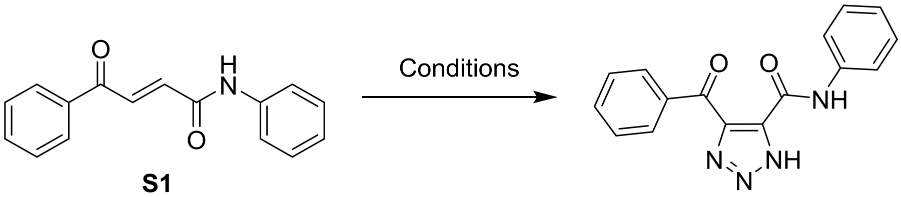

S1

S21

Entry	Conditions	Yield after purification
1	NaN_3 (1 eq.), $CuCl_2$ (1 eq.), DMF, 80°C, 48h, inert atmosphere.	Not isolated
2	NaN_3 (1 eq.), CuO (1 eq.), DMF, 80°C, 48h, inert atmosphere.	36%

2. Chemical Procedures

2.1. General Information

Chemicals were purchased from commercial suppliers and used without further purification. Thin layer chromatography (TLC) was performed on aluminium plates coated with 60 F_{254} silica from Merck. Flash chromatography was carried out using a Biotage SP4, Biotage Isolera or Varian automated flash system with Silicycle or GraceResolve normal phase silica gel pre-packed columns. Fractions were collected at 254 nm or if necessary on all wavelengths between 200 and 400 nm. Microwave irradiation was performed in a Biotage Initiator Sixty in sealed vials. Reactions were irradiated at 2.45 GHz and were able to reach temperatures between 60 and 250 °C. Heating was at a rate of 2-5 °C/s and the pressure was able to reach 20 bars.

2.2. Analytical Equipment

Melting points were measured using a Stuart automatic melting point SMP40 apparatus. Fourier Transform InfraRed (FTIR) spectra were measured using an Agilent Cary 630 FTIR. The abbreviations for peak description are as follows: b = broad; w = weak; m = medium and s = strong. Ultraviolet (UV) spectra were recorded on a Hitachi U-2900 spectrophotometer and were performed in ethanol. High resolution mass spectrometry (HRMS) was provided by the ESPRC National Mass Spectrometry Service, University of Wales, Swansea or conducted using an Agilent 6550 iFunnel QTOF LC-MS with an Agilent 1260 Infinity UPLC system. The sample was eluted on Acquity UPLC BEH C18 (1.7µm, 2.1 x 50mm) with a flow rate of 0.7 mL/min and run at a gradient of 1.2 min 5-95% 0.1% aq. HCOOH in MeCN.

LC-MS analyses were conducted using a Waters Acquity UPLC system with photo diode array (PDA) and evaporating light scattering detector (ELSD). When a 2 min gradient was used, the sample was eluted on an Acquity UPLC BEH C18, 1.7µm, 2.1 x 50mm, with a flow rate of 0.6 ml/min using 5-95% 0.1% HCOOH in MeCN. Analytical purity of compounds was determined using Waters XTerra RP18, 5 µm (4.6 × 150 mm) column at 1 ml/min using either 0.1% aq. ammonia and MeCN or 0.1% aq. HCOOH and MeCN with a gradient of 5-100% over 15 min.

^{1}H NMR spectra were obtained using a Bruker Avance III 500 spectrometer using a frequency of 500 MHz. ^{13}C spectra were acquired using the Bruker Avance III 500 spectrometer operating at a frequency of 126 MHz. The abbreviations for spin multiplicity are as follows: s = singlet; d = doublet; t = triplet; q = quartet, p = quintuplet, h = sextuplet and m = multiplet. Combinations of these abbreviations are employed to describe more complex splitting patterns (e.g. dd = doublet of doublets).

2.3. General Procedures

2.3.1. Build

General Procedure 1 (2): In a microwave vial, acetyl derivative (1 eq.), glyoxylic acid monohydrate (3 eq.) and TsOH monohydrate (1 eq.) were dissolved in dioxane (2.5 M). The vial was closed and heated in the microwave for 1 h at 160 °C using the low absorption mode. 2 M HCl aqueous solution was added to the mixture if the product was not zwitterionic. This was extracted 3 times with CH_2Cl_2. Combined organic phases were dried over $MgSO_4$ and concentrated under pressure. The crude material was purified by flash chromatography.

General Procedure 2 (2): In a microwave vial, methyl ketone-containing compound (1 eq.), glyoxylic acid monohydrate (3 eq.) and acetic acid (1 eq.) were dissolved in MeOH (2.5 M) then pyrrolidine (1 eq.) was added. The vial was sealed and the mixture stirred for 5 min. The mixture was then irradiated using the MW at 60 °C for 8 h. The solvent was removed under vacuum and the crude material was purified by flash chromatography.

General Procedure 3 (2): A solution of maleic anhydride **5** (1.0 eq.) and trichloroalumnium (1.5 eq.) in dichloromethane (2.2 M) was stirred at room temperature until all solids had dissolved. The aromatic substrate (1.5 eq.) was then added dropwise, and the reaction stirred at room temperature until completed. The reaction was slowly poured into hydrochloric acid (0.2 M aq. sol.) and extracted with dichloromethane. The combined organic layers were dried ($MgSO_4$) and concentrated under reduced pressure. The crude material was purified by flash column chromatography.

2.3.2. Couple

General Procedure 4 (1): In a RB flask, carboxylic acid **2** (1.5 eq.), HATU (1.5 eq.) and DIPEA (1.2 eq.) were dissolved in anhydrous dichloromethane (4 M). The mixture was stirred for 1 h at rt under inert atmosphere. Amine **6** (1.0 eq) was then slowly added. The mixture was stirred overnight at rt under inert atmosphere. Water was then added to the mixture which was extracted with CH_2Cl_2. Combined organic phases were washed with saturated brine and dried over MgSO4 and concentrated under pressure. The crude material was purified by flash chromatography.

General Procedure 5 (1): A solution of carboxylic acid **2** (1.0 eq.) and amine **6** (1.3 eq.) in THF (0.25 M) was stirred at 0 °C for 20 minutes before the dropwise addition phosphorus (V) oxychloride (1.3 eq.) followed by triethylamine (3.0 eq.). The reaction was stirred at 0 °C for 30 minutes, then warmed to room temperature and stirred for 3 hours. Once complete, the reaction was quenched with water and extracted with ethyl acetate. The combined organic layers were washed with brine, dried ($MgSO_4$) and concentrated under reduced pressure. The crude material was purified by flash column chromatography.

2.3.3. Transformations: 1,4-Cyclisations

General Procedure 6 (7): In a RB-flask, ketoenamide **1** (1.0 eq.) and hydrazine monohydrate (2.0 eq.) were dissolved in EtOH (0.25 M). The mixture was stirred at 60 °C for 2 h. Solvent was removed under vacuum. The crude was dissolved in CH_2Cl_2 (0.05 M). MnO_2 (12.0 eq.) was added. The mixture was stirred at 60 °C for 2 h before being filtered through a celite pad. Solvent was removed under vacuum. The crude material was purified by flash chromatography.

General Procedure 7 (8): A solution of ketoenamide **1** (1.0 eq.), hydroxylamine hydrochloride (2.0 eq.) and sodium acetate (4.0 eq.) in DMSO (0.13 M) was stirred at 60 °C overnight. The reaction was cooled to room temperature, quenched with water and extracted with dichloromethane. The combined organic layers were washed with brine, dried ($MgSO_4$) and concentrated under reduced pressure. The residue was dissolved in dichloromethane (0.04 M) and manganese (IV) dioxide (12.0 eq.) and stirred at 60 °C for 4 hours. The crude material was purified by flash column chromatography.

General Procedure 8 (9): A solution of ketoenamide **1** (1.0 eq.) and 3-aminopyrazole (1.3 eq.) in DMF (0.15 M) was stirred at 110 °C for 3 days. The reaction was cooled to room temperature then concentrated under reduced pressure. The crude material was purified by flash column chromatography.

General Procedure 9 (10): A solution of ketoenamide **1** (1.0 eq.), 2-aminoimidazole sulfate (2.0 eq.) and sodium hydrogen carbonate (3.0 eq.) in DMF (0.15 M) was stirred at 110 °C for 3 days. The reaction was cooled to room temperature then concentrated under reduced pressure. The crude material was purified by flash column chromatography.

General Procedure 10 (11): In a RB-flask, ketoenamide **1** (1.0 eq.), methyl carbamimidate hydrochloride (1.3 eq.) and $NaHCO_3$ (4.0 eq.) were dissolved in DMF (1.2 M). The mixture was stirred at 75 °C for 5 h. The solvent was removed under vacuum and the crude mixture was purified by flash chromatography.

General Procedure 11 (12): In a RB-flask, methoxypyrimidine **11** (1.0 eq.) was dissolved 1.25M HCl (20 eq.) in EtOH. The mixture was heated at 100 °C for 2 h under microwave irradiation. The solvent was removed under vacuum and the crude material was purified by flash chromatography.

General Procdure 12 (13): In a RB-flask, ketoenamide **1** (1.0 eq.), 2-(4-methoxybenzyl)isothiouronium chloride (1.3 eq.) and $NaHCO_3$ (4.0 eq.) were dissolved in DMF (0.8 M). The mixture was stirred at 80 °C for 3 h. 10 mL of water were then added to the mixture, which was extracted with EtOAc. Combined organic phases were washed brine (aq. sol.) before being dried (MgSO4) and concentrated under vacuum. The crude material was purified by flash chromatography.

General Procedure 13 (14): In a RB-flask, ketoenamide **1** (1.0 eq.) and guanidine carbonate (1.1 eq.) were dissolved in DMF (0.16 M). The mixture was stirred at 80 °C for 3 h. The solvent was removed under vacuum and the crude material was purified by flash chromatography.

General Procedure 14 (15): In a RB-flask, ketoenamide **1** (1.0 eq.), *N*-acetonylpyridinium chloride (1.0 eq.) and NH_4OAc (3.0 eq.) were dissolved in EtOH (0.06 M). The mixture was stirred at 75 °C for 2 h. The solvent was removed under vacuum. The crude material was purified by flash chromatography.

2.3.4. Transformations: 3,4-Cyclisations

General Procedure 15 (16): Trimethylsulfonium iodide (1.0 eq.) and sodium hydride (1.0 eq.) were purged with nitrogen (x 3) before the addition of DMSO (0.3 M). The reaction was stirred at room temperature for 1 hour, before

the addition of ketoenamide **1** (1.0 eq.) in DMSO (0.3 M). The reaction was stirred at room temperature until complete then quenched by the addition of saturated aqueous ammonium chloride solution. The aqueous was extracted with ethyl acetate and the combined organic layers were washed with brine, dried ($MgSO_4$) and concentrated under reduced pressure. The crude material was purified by flash column chromatography.

General Procedure 16 (17): In a RB flask, ketoenamide **1** (1.0 eq.) and $Yb(OTf)_3$ (1.2 eq.) were dissolved in MeCN (0.1 M). Freshly distilled cyclopentadiene (20.0 eq.) was added. The reaction was stirred at rt for 2 h and then concentrated under vacuum. The crude material was purified by flash chromatography.

General Procedure 17 (19): In a RB flask, ketoenamide **1** (1.0 eq.), 2,3-dimethyl-1,3-butadiene (10.0 eq.) and $Yb(OTf)_3$ (1.2 eq.) were dissolved in MeCN (0.6 M). The mixture was heated at 110 °C for 30 min and then concentrated under vacuum. The crude material was purified by flash chromatography.

General procedure 18 (20): Ketoenamide **1** (1.0 eq.) and TosMIC (1.1 eq.) were purged with nitrogen (x 3) then dissolved in diethyl ether (0.15 M) and DMSO (0.3 M) and stirred at room temperature for 15 minutes. This solution was then added to a solution of sodium hydride (2.2 eq.) in diethyl ether (0.3 M) and the resulting reaction was stirred at room temperature for 30 minutes. The reaction was quenched with saturated aqueous ammonium chloride and extracted with ethyl acetate. The combined organic layers were washed with brine, dried ($MgSO_4$) and concentrated under reduced pressure. The crude material was purified by flash column chromatography.

General Procedure 19 (21): A solution of ketoenamide **1** (1.0 eq.) and K_2CO_3 (2.0 eq.) were dissolved in 1 mL of H_2O/dioxane 4:1 (0.08 M). BnN_3 (2.0 eq.) was then added and the mixture was stirred at 80 °C overnight under inert atmosphere. H_2O was added to the mixture and this was extracted with CH_2Cl_2. Combined organic phases were washed saturated brine aqueous solution, dried over MgSO4 and concentrated under vacuum. The crude material was purified by flash chromatography.

General Procedure 20 (22): In a RB flask, ketoenamide **1** (1.0 eq.), NaN_3 (1.0 eq.) and CuO (1.0 eq.) were dissolved in anhydrous DMF (0.2 M). The mixture was stirred at 80 °C for 4 h under inert atmosphere. Saturated NH4Cl aq. solution was added to the mixture which was extracted with CH_2Cl_2. Combined organic phases were washed with saturated brine aqueous solution, dried over MgSO4, and concentrated under vacuum. The crude material was purified by flash chromatography.

General Procedure 21 (23): In a RB flask, ketoenamide **1** (1.0 eq.) and *N*-methylpropan-1-imine oxide (2.2 eq.) were dissolved in CH_2Cl_2 (0.16 M). The mixture was stirred at 60 °C for 18 h under inert atmosphere. The solvent was removed under vacuum. The crude material was purified by flash chromatography.

2.3.5. Transformations: Reductions

General Procedure 22 (24): In a RB-flask, ketoenamide **1** (1.0 eq.) was dissolved in MeOH (0.04 M). The mixture was passed through the H-cube (full H2, rt) for 30 min. Solvent was removed under vacuum. The crude material was purified by flash chromatography.

General Procedure 23 (25): In a RB-flask, ketoenamide **1** (1.0 eq.) and $CeCl_3{\cdot}7H_2O$ (1.0 eq.) were dissolved in 4 mL of CH_2Cl_2/MeOH 1:1 (0.1 M). The mixture was stirred at rt until complete dissolution. Then $NaBH_4$ (1.0 eq.) was added portionwise then stirred for 15 min. Saturated NH_4Cl aq. solution was added to the mixture, which was extracted with CH_2Cl_2. Combined organic phases were washed with water before being dried over MgSO4. The solvent was removed under vacuum. The crude material was purified by flash chromatography.

General Procedure 24 (26): In a MW vial, ketoenamide **1** (1.0 eq.) was dissolved in anhydrous CH_2Cl_2/MeOH 1:1 (0.1 M). $NaBH_4$ (3.0 eq.) was added portionwise then stirred at rt for 1 h under inert atmosphere. $NiCl_2.6H_2O$ (0.5 eq.) followed by $NaBH_4$ (2.0 eq.) were added and stirring continued at rt for 30 min. Saturated NH4Cl aq. solution was added to the mixture, which was extracted with CH_2Cl_2. Combined organic phases were washed with water before being dried over MgSO4. The solvent was removed under vacuum. The crude mixture was purified by flash chromatography.

2.3.6. Transformations: 1,4-Additions

General Procedure 25 (27): In a RB-flask, ketoenamide **1** (1.0 eq.) was dissolved in anhydrous EtOH (0.32 M). Pyrrolidine (2.0 eq.) was slowly added. The mixture was stirred at rt for 5 min under inert atmosphere. The solvent was then removed under vacuum. The crude material was purified by flash chromatography.

2.4. Compound Data

2.4.1. Build

(*E*)-4-(4-methoxyphenyl)-4-oxobut-2-enoic acid (2b)

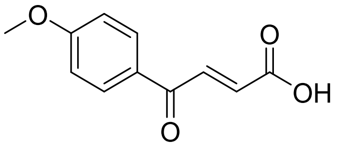

Compound 10 could be obtained following general procedures 1 or 3:

General procedure 1: Flash chromatography (0 to 10% 0.1 AcOH in MeOH in CH_2Cl_2) provided **2b** as a bright yellow solid (2.59 g, 12.56 mmol, 94%).

General Procedure 3: Reverse phase flash chromatography (10 to 50% MeCN/H_2O) yielded compound **2b** as a bright yellow solid (693 mg, 3.36 mmol, 66%).

R_f = 0.65 (80% EtOAc in PE); mp = 180-182 °C; UV λ_{max} (EtOH/nm) 287.2, 223.6, 200.0; FTIR (cm^{-1}) ν 2840 (b, m, O-H acid), 1699 (s, C=O acid), 1661 (s, C=O ketone), 1592 (s, C=C alkene), 1511 (s, C=C aromatic), 1420 (s, O-H acid); ^1H NMR (Chloroform-*d*, 500 MHz) δ 3.90 (3H, s), 6.88 (1H, d, *J* = 15.5 Hz), 6.97 – 7.02 (2H, m), 7.97 – 8.05 (3H, m); ^{13}C NMR (Chloroform-*d*, 126 MHz) δ 55.77, 114.39, 129.6, 130.61, 131.55, 138.77, 164.57, 169.84, 187.48; MS(ES+) *m/z* 207.2.

Data in accordance to reference.[1]

(*E*)-4-(4-cyanophenyl)-4-oxobut-2-enoic acid (2c)

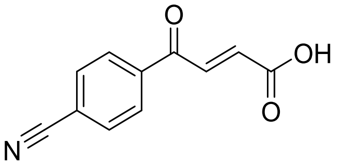

Compound **2c** was obtained following General Procedure 1. Normal phase flash chromatography (0 to 10% 0.1% AcOH in MeOH in CH_2Cl_2) yielded compounds **2c** as a pale yellow solid (443 mg, 2.19 mmol, 32%).

R_f = 0.12 (10% MeOH in CH_2Cl_2); mp = 134-140 °C; UV λ_{max} (EtOH/nm) 256.4; FTIR (cm^{-1}) ν 3063 (b, m, O-H acid), 2231 (w, C≡N); ^1H NMR (Methanol-d4, 500 MHz) δ 6.83 (1H, d, *J* = 15.6 Hz), 7.91 (1H, d, *J* = 15.6 Hz), 7.93 (2H, d, *J* = 8.4 Hz), 8.17 (2H, d, *J* = 8.4 Hz); ^{13}C NMR (Methanol-d_4, 126 MHz) δ 117.86, 118.87, 130.43, 133.93, 135.05, 136.84, 141.19, 168.23, 190.30; MS (ES+) m/z = 201.1 $[M – H]^-$; HRMS calcd for $C_{11}H_7NO_3$ 200.0348 [M+H]+ found 200.0363.

Data in accordance to reference.[1]

(*E*)-4-Cyclohexyl-4-oxobut-2-enoic acid (2d)

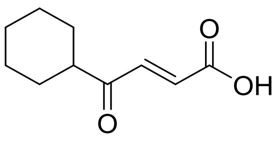

Compound **2d** was obtained following General procedure 2. Flash chromatography (0 to 15% 0.1% AcOH in MeOH in CH_2Cl_2) yielded **2d** as a beige solid (277 mg, 1.52 mmol, 38%).

R_f = 0.32 (5% MeOH in CH_2Cl_2); mp = 113-115 °C; UV λ_{max} (EtOH/nm) 330.8, 219.8; FTIR (cm^{-1}) ν 3062 (b, m, O-H acid), 2922 (s, C-H alkane), 2851 (s, C-H alkane), 1660 (s, C=O acid), 1427 (s, O-H acid); ^1H NMR (Methanol-d_4, 500 MHz) δ 1.05 – 1.21 (5H, m), 1.51 – 1.67 (5H, m), 2.50 (1H, tt, *J* = 10.7, 3.4 Hz), 6.42 (1H, d, *J* = 15.9 Hz), 6.91 (1H, d, *J* = 15.9 Hz); ^{13}C NMR (Methanol-d_4, 126 MHz) δ 26.49, 29.31, 50.35, 132.21, 139.56, 168.50, 204.38; MS(ES+) *m/z* 183.1 $[M + H]^+$; HRMS calcd for $C_{10}H_{14}O_3$ $[M - H]^-$ 181.0870, found 181.0870.

Data in accordance to reference.[1]

(*E*)-4-Oxo-4-(tetrahydro-2H-pyran-4-yl)but-2-enoic acid (2e)

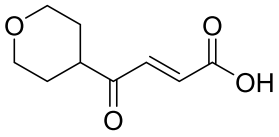

Compound **2e** was obtained following General procedure 2. Flash chromatography (0 to 10% 0.1% AcOH in MeOH in CH_2Cl_2) yielded **2e** as an orange solid (321 mg, 1.74 mmol, 38%).

R_f = 0.41 (5% MeOH in CH_2Cl_2); mp = 90-92 °C; UV λ_{max} (EtOH/nm) 243.0, 201.7; FTIR (cm^{-1}) ν 3067 (b, m, O-H acid), 2963 (m, C-H alkane), 2841 (m, C-H alkane), 1669 (s, C=O acid), 1424 (s, O-H acid); ^{1}H NMR (Methanol-d_4, 500 MHz) δ 1.53 (2H, dtd, *J* = 13.6, 11.5, 4.4 Hz), 1.71 (2H, ddd, *J* = 13.3, 4.1, 2.1 Hz), 2.93 (1H, tt, *J* = 11.3, 3.8 Hz), 3.42 (2H, td, *J* = 11.6, 2.2 Hz), 3.87 (2H, ddd, *J* = 11.6, 4.3, 2.3 Hz), 6.62 (1H, d, *J* = 15.9 Hz), 7.07 (1H, d, *J* = 15.9 Hz); ^{13}C NMR (Methanol-d_4, 126 MHz) δ 29.06, 47.01, 68.07, 132.75, 139.07, 168.44, 202.59; MS(ES+) *m/z* 185.1 $[M + H]^+$; HRMS calcd for $C_9H_{12}O_4$ $[M + H]^+$ 183.0663, found 183.0644.

Data in accordance to reference.[1]

2.4.2. Couple

(*E*)-4-Oxo-N,4-diphenylbut-2-enamide (1a)

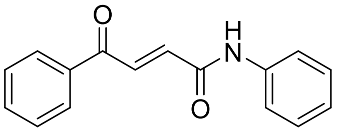

Compound **1a** could be obtained following General procedures 4 or 5

General procedure 4: Normal phase flash chromatography (0 to 20% EtOAc in PE) yielded **1a** as a bright yellow solid (930 mg, 3.70 mmol, quant.).

General procedure 5: Normal phase flash chromatography (0 to 20% EtOAc in PE) yielded **1a** as a bright yellow solid (830 mg, 3.30 mmol, 58%).

R_f = 0.24 (20% EtOAc in PE); mp = 148-150 °C; UV λ_{max} (EtOH/nm) 237.2, 202.1; FTIR (cm^{-1}) ν 3336 (m, N-H amide), 1650 (s, C=O amide), 1596 (s, C=O ketone); ^{1}H NMR (Chloroform-*d*, 500 MHz) δ 7.16 (1H, t, *J* = 7.5 Hz), 7.35 (2H, t, *J* = 7.9 Hz), 7.40 (1H, d, *J* = 14.9 Hz), 7.50 (2H, t, *J* = 7.7 Hz), 7.62 (1H, t, *J* = 7.1 Hz), 7.70 (2H, d, *J* = 8.2 Hz), 8.04 (2H, dd, *J* = 8.3, 1.1 Hz), 8.13 (1H, d, *J* = 14.9 Hz), 8.74 (1H, s); ^{13}C NMR (Chloroform-*d*, 126 MHz) δ 120.38, 125.17, 129.08, 129.24, 133.81, 134.19, 136.53, 136.92, 137.85, 162.33, 190.31; MS(ES+) *m/z* 252.2 [M + H]$^+$; HRMS calcd for $C_{16}H_{13}NO_2$ [M + Na]$^+$ 274.0838, found 274.0768.

(*E*)-*N*-(3,4-dimethoxyphenyl)-4-(4-methoxyphenyl)-4-oxobut-2-enamide (1b)

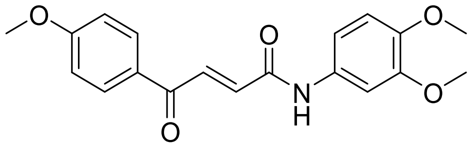

Compound **1b** was obtained following General procedure 5. Normal phase flash chromatography (0 to 5% MeOH in CH_2Cl_2) yielded compound **1b** as a bright yellow solid (406 mg, 1.19 mmol, 81%).

R_f = 0.59 (5% MeOH in CH_2Cl_2); mp = 158-160 °C; UV λ_{max} (EtOH/nm) 322.4, 240.2, 201.8; FTIR (cm^{-1}) ν 3270 (m, N-H amide), 1642 (s, C=O amide), 1599 (s, C=O ketone), 1547 (s, C=C alkene), 1510 (s, C=C aromatic); ^{1}H NMR (Chloroform-*d*, 500 MHz) δ 3.89 (3H, s), 3.90 (3H, s), 3.91 (3H, s), 6.85 (1H, d, *J* = 8.6, 2.5 Hz), 6.95 – 7.04 (3H, m), 7.15 (1H, d, *J* = 14.8 Hz), 7.53 (1H, d, *J* = 2.5 Hz), 8.03 – 8.14 (3H, m); 13C NMR (Chloroform-*d*, 126 MHz) δ 55.74, 56.01, 56.22, 104.99, 111.42, 112.33, 114.34, 130.03, 131.56, 131.68, 133.58, 135.92, 146.42, 149.18, 162.32, 164.55, 188.40; MS(ES+) *m/z* 342.3 [M + H]$^+$; HRMS calcd for $C_{19}H_{19}NO_5$ [M + H]$^+$ 342.1339, found 342.1337.

(*E*)-4-(4-Cyanophenyl)-4-oxo-*N*-phenylbut-2-enamide (1c)

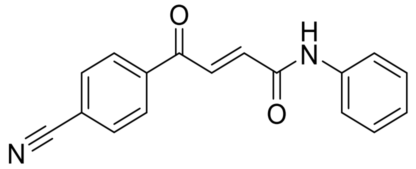

Compound **1c** was obtained following General procedure 5. Reverse phase flash chromatography (30 to 50% 0.1% HCO_2H in MeCN in H_2O) yielded compound **1c** as a yellow solid (858 mg, 3.11 mmol, 69%). Trituration with CH_2Cl_2 provided the desired product pure (425 mg, 1.54 mmol, 34%).

R_f = 0.41 (50% MeCN in H_2O); mp = 204-206 °C ;UV λ_{max} (EtOH/nm) 250.0, 202.3; FTIR (cm^{-1}) ν 3280 (m, N-H amide), 2228 (w, C≡N), 1643 (s, C=O amide), 1600 (s, C=O ketone), 1538 (s, C=C aromatic); ^{1}H NMR (Chloroform-*d*, 500 MHz) δ 7.05 (1H, t, *J* = 7.4 Hz), 7.16 (1H, dd, *J* = 15.0, 2.5 Hz), 7.22 – 7.30 (2H, m), 7.58 (2H, d, *J* = 7.9 Hz), 7.75 (2H, d, *J* = 7.9 Hz), 7.91 (1H, dd, *J* = 15.1, 2.5 Hz), 8.06 (2H, dd, *J* = 8.5, 2.5 Hz); ^{13}C NMR (Chloroform-*d*, 126 MHz) δ 116.67, 117.74, 120.11, 124.93, 128.94, 129.21, 132.21, 132.71, 137.72, 137.89, 139.88, 162.30, 189.02; MS(ES+) *m/z* 277.3 [M + H]$^+$; HRMS calcd for $C_{17}H_{12}N_2O_2$ [M + H]$^+$ 277.0972, found 277.0959.

(*E*)-4-Cyclohexyl-4-oxo-*N*-phenylbut-2-enamide (1d)

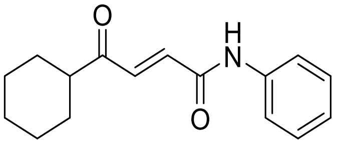

Compound **1d** was obtained following General procedure 4. Reverse phase flash chromatography (50 to 60% MeCN in H_2O) yielded **1d** with HPLC purity of 93% as a pale yellow solid (831 mg, 3.23 mmol, 93%). Trituration with CH_2Cl_2 provided **39** as a white solid (559 mg, 2.17 mmol, 70%).

R_f = 0.33 (20% EtOAc in PE); UV λ_{max} (EtOH/nm) 293.4, 227.2, 201.7; FTIR (cm^{-1}) ν 3312 (m, N-H amide), 2922(m, C-H alkane), 2851(m, C-H alkane), 1690 (s, C=O amide), 1650 (s, C=O ketone), 1599 (s, C=C aromatic), 1532 (c, C=C aromatic); ^{1}H NMR (Chloroform-*d*, 500 MHz) δ 1.17 – 1.27 (1H, m), 1.29 – 1.44 (4H, m), 1.66 – 1.74 (1H, m), 1.82 (2H, dt, *J* = 12.3, 3.3 Hz), 1.92 (2H, d, *J* = 12.3 Hz), 2.58 (1H, tt, *J* = 10.8, 2.9 Hz), 6.97 (1H, d, *J* = 15.1 Hz), 7.16 (1H, t, *J* = 7.4 Hz), 7.32 – 7.40 (3H, m), 7.62 (2H, d, *J* = 7.8 Hz), 7.78 (1H, s); ^{13}C NMR (Chloroform-*d*, 126 MHz) δ 25.66 , 25.92, 28.21 , 50.78, 120.21, 125.22, 129.30, 133.83, 136.13, 137.61, 162.19, 202.81; MS(ES+) *m/z* 258.3; [M + H]$^+$; HRMS calcd for $C_{16}H_{19}NO_2$ [M + H]$^+$ 258.1489, found 258.1492.

(*E*)-4-oxo-*N*-phenyl-4-(tetrahydro-2*H*-pyran-4-yl)but-2-enamide (1e)

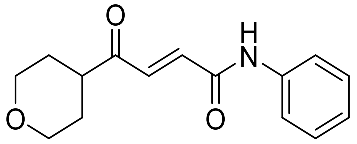

Compound **1e** was obtained following General procedure 4. Reverse phase flash chromatography (30 to 40% MeCN in H_2O) yielded **1e** with HPLC purity of 94% as a yellow solid (1.14 g, 4.40 mmol, 85%). Trituration with CH_2Cl_2 provided **40** as a pale yellow solid (738 mg, 2.84 mmol, 59%).

R_f = 0.55 (20% EtOAc in PE); UV λ_{max} (EtOH/nm) 244.2, 201.6; FTIR (cm^{-1}) ν 3287 (m, N-H amide), 2948 (m, C-H alkane), 2843 (m, C-H alkane), 1694 (s, C=O amide), 1648 (s, C=O ketone), 1599 (s, C=C aromatic), 1531 (s, C=C aromatic); ^{1}H NMR (Methanol-d_4, 500 MHz) δ 1.61 (2H, dtd, *J* = 13.6, 11.2, 4.4 Hz), 1.74 (2H, ddq, *J* = 13.6, 4.4, 2.4 Hz), 2.75 (1H, tt, *J* = 11.2, 3.8 Hz), 3.39 (2H, td, *J* = 11.6, 2.4 Hz), 3.91 (2H, ddd, *J* = 11.2, 4.4, 2.4 Hz), 6.94 (1H, d, *J* = 15.3 Hz), 7.02 (1H, tt, *J* = 7.5, 1.1 Hz), 7.19 (1H, d, *J* = 15.3 Hz), 7.21 – 7.25 (2H, m), 7.54 (2H, dq, *J* = 8.5, 1.8, 1.1 Hz); ^{13}C NMR (Methanol-d_4, 126 MHz) δ 27.59, 46.96, 67.03 120.07, 124.79, 128.89, 134.37, 135.15, 137.96, 162.55, 201.43; MS(ES+) *m/z* 260.2; [M + H]$^+$; HRMS calcd for $C_{15}H_{17}NO_3$ [M + H]$^+$ 260.1281, found 260.1282.

(*E*)-4-(4-methoxyphenyl)-4-oxo-*N*-phenylbut-2-enamide (1f)

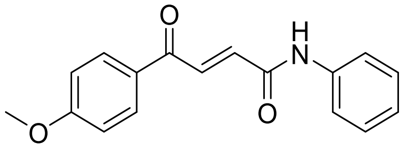

Compound **1f** was obtained following General procedure 4. Normal phase flash chromatography (0 to 50% EtOAc in PE) yielded compound **1f** with some side-product as a yellow solid (295 mg, 1.05 mmol, 95%). Trituration with CH_2Cl_2 provided **32** as a pale yellow solid (145 mg, 0.52 mmol, 47%).

R_f = 0.40 (30% EtOAc in PE); mp = 189-191 °C; UV λ_{max} (EtOH/nm) 317.0, 267.0, 201.5; FTIR (cm^{-1}) ν 3320 (m, N-H amide), 1650 (s, C=O amide), 1594 (s, C=O ketone), 1541 (s, C=C aromatic); ^{1}H NMR (Chloroform-*d*, 500 MHz) δ 3.88 (3H, s,), 6.95 (2H, d, *J* = 8.6 Hz), 7.15 (2H, t, *J* = 7.4 Hz), 7.34 (1H, t, *J* = 7.8 Hz), 7.42 (1H, d, *J* = 14.9 Hz), 7.71 (2H, d, *J* = 7.9 Hz), 8.05 (2H, d, *J* = 8.7 Hz), 8.13 (1H, d, *J* = 14.9 Hz), 8.97 (1H, s); ^{13}C NMR (Chloroform-*d*, 126 MHz) δ 55.72, 114.32, 120.37, 125.00, 129.18, 129.99, 131.61, 133.81, 135.98, 138.03, 162.59, 164.54, 188.49; MS(ES+) *m/z* 282.2; [M + H]$^+$; HRMS calcd for $C_{17}H_{15}NO_3$ [M + H]$^+$ 282.1125, found 282.1100.

(*E*)-1-Phenyl-4-(piperidin-1-yl)but-2-ene-1,4-dione (1h)

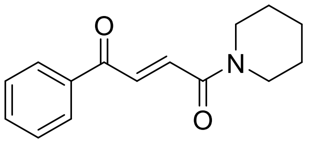

Compound **1h** was obtained following General procedure 5. Flash chromatography (0-50% EtOAc in petrol then 0-10% IPA in petrol) yielded compound **1h** as a pale yellow crystalline solid (204 mg, 0.838 mmol 39%).

Rf = 0.39 (10% IPA in petrol); mp: 85-88 °C; UV λmax (EtOH/nm) 268.4; IR νmax (cm^{-1}) 3018 (w, C-H), 2926 (m, C-H), 2858 (m, C-H), 1623 (s, C=O), 1605 (s, C=O); ^{1}H NMR (Methanol-d^4, 500 MHz) δ 1.59 – 1.68 (4H, m), 1.68 – 1.75 (2H, m), 3.63 (2H, t, *J* = 5.5 Hz), 3.67 (2H, t, *J* = 5.5 Hz), 7.51 – 7.58 (3H, m), 7.64 – 7.68 (1H, m), 7.82 (1H, d, *J* = 15.2 Hz), 8.04 – 8.06 (2H, m); ^{13}C NMR (Methanol-d^4, 126 MHz) δ 25.42, 26.76, 27.78, 44.57, 48.56, 129.87, 130.06, 134.54, 134.85, 134.95, 138.28, 166.08, 191.25; MS(ES$^+$) *m/z* = 244.2 [M + H]$^+$; HRMS calcd for $C_{15}H_{17}NO_2$ [M +H]$^+$ 244.1332 found 244.1475.

(*E*)-1-Morpholino-4-phenylbut-2-ene-1,4-dione (1i)

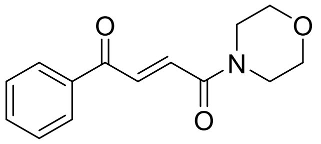

Compound **1i** was obtained following General procedure 5. Flash chromatography (0-80% EtOAc in petrol), crystallisation and trituration with EtOAc yielded compound **1i** as a pale yellow-white solid (222 mg, 0.905 mmol, 36%).

Rf = 0.40 (80% EtOAc in petrol); mp: 136-139 °C; UV λmax (EtOH/nm) 270.4; IR νmax (cm-1) 3062 (w, C-H), 2967 (m, C-H), 2929 (m, C-H), 1671 (w, C=C), 1631 (s, C=O), 1600 (s, C=O); ^{1}H NMR (Methanol-d^4, 500 MHz) δ 3.68 – 3.74 (8H, m), 7.49 – 7.58 (3H, m), 7.67 (1H, t, *J* = 7.5 Hz), 7.88 (2H, d, *J* = 15.2 Hz), 8.06 (2H, d, *J* = 7.7 Hz); ^{13}C NMR (Methanol-d^4, 126 MHz) δ 43.89, 47.81, 67.66, 67.90, 129.89, 130.08, 133.59, 135.00, 135.55, 138.24, 166.34, 191.18; MS(ES$^+$) *m/z* = 246.2 [M + H]$^+$; HRMS calcd for $C_{14}H_{15}NO_3$ [M + H]$^+$ 246.1125 found 246.1330.

(*E*)-*N*-(2-Methoxyethyl)-4-oxo-4-phenylbut-2-enamide (1j)

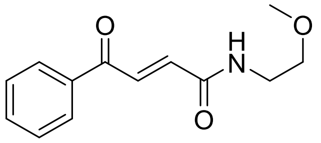

Compound **1j** was obtained following general procedure 5. Flash chromatography (0-70% EtOAc in petrol), crystallisation and trituration from EtOAc yielded compound **1j** as a white solid (150 mg, 0.643 mmol 25%).

Rf = 0.23 (70% EtOAc in petrol); mp: 108-111 °C; UV λmax (EtOH/nm) 266.4; IR νmax (cm^{-1}) 3301 (m, N-H amide), 3067 (w, C-H), 2886 (w, C-H), 2832 (w, C-H),1634 (s, C=O), 1553 (s, C=O); ^{1}H NMR (Methanol-d^4, 500 MHz,) δ 3.37 (3H, s), 3.48 – 3.54 (4H, m), 7.04 (1H, d, *J* = 15.3 Hz), 7.55 (2H, t, *J* = 7.8 Hz), 7.64 – 7.68 (1H, m), 7.88 (1H, d, *J* = 15.3 Hz), 8.02 – 8.05 (2H, m); ^{13}C NMR (Methanol-d^4, 126 MHz) δ 40.70, 58.95, 71.76, 129.85, 130.04, 134.13, 134.92, 136.50, 138.33, 166.81, 191.53; MS(ES$^+$) *m/z* = 234.2 [M + H]$^+$; HRMS calcd for $C_{13}H_{15}NO_3$ [M + H]$^+$ 234.1125 found 234.1339.

(*E*)-*N*-(1-Methyl-1H-pyrazol-3-yl)-4-oxo-4-phenylbut-2-enamide (1k)

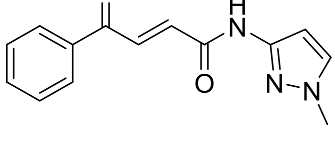

Compound **1k** was obtained following General procedure 5. Trituration with DCM yielded compound **1k** as a creamy white solid (609 mg, 2.38 mmol 99%).

Rf = 0.23 (70% EtOAc in petrol); mp: 217-221 °C; UV λmax (EtOH/nm) 232.4; IR νmax (cm^{-1}) 3222 (m, N-H amide), 3132 (w, C-H), 3041 (w, C-H),1657 (s, C=O), 1575 (s, C=O); ^{1}H NMR (DMSO-d_6, 500 MHz) δ 3.77 (3H, s), 6.60 (1H, d, *J* = 2.2 Hz), 7.20 (1H, d, *J* = 15.3 Hz), 7.57 – 7.62 (3H, m), 7.68 – 7.73 (1H, m), 7.89 (1H, d, *J* = 15.3 Hz), 8.02 – 8.06 (2H, m), 11.09 (1H, s); ^{13}C NMR (DMSO-d_6, 126 MHz) δ 38.40, 96.95, 128.66, 129.00, 131.17, 132.87, 133.78, 136.00, 136.57, 146.47, 160.87, 189.64; MS(ES$^+$) *m*/*z* = 256.2 [M + H]$^+$; HRMS calcd for $C_{14}H_{13}N_3O_2$ [M + H]$^+$ 256.1081 found 256.1355.

(*E*)-*N*-Benzyl-4-oxo-4-phenylbut-2-enamide (1l)

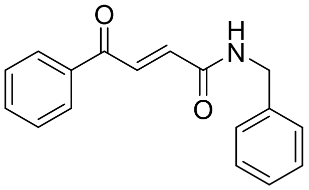

Compound **1l** was obtained following general procedure 5. Flash chromatography (0-50% EtOAc in petrol), crystallisation and trituration with EtOAc yielded compound **1l** as a white solid (328 mg, 1.24 mmol, 50%).

R_f = 0.56 (50% EtOAc in petrol); mp: 148-151 °C; UV λmax (EtOH/nm) 264.2; IR νmax (cm^{-1}) 3288 (m, N-H amide),1671 (m, C=C), 1633 (s, C=O), 1543 (s, C=O); ^{1}H NMR (Methanol-d^4, 500 MHz) δ 4.51 (2H,s), 7.06 (1H, d, *J* = 15.3 Hz), 7.24 – 7.29 (1H, m), 7.31 – 7.36 (4H, m), 7.55 (2H, t, *J* = 7.8 Hz), 7.64 – 7.68 (1H, m), 7.92 (1H, d, *J* = 15.3 Hz), 8.02 – 8.05 (2H, m); ^{13}C NMR (Methanol-d^4, 126 MHz) δ 44.60, 128.46, 128.79, 129.67, 129.86, 130.05, 134.33, 134.93, 136.45, 138.31, 139.42, 166.55, 191.47; MS(ES$^+$) *m*/*z* = 266.2 [M + H]$^+$; HRMS calcd for $C_{17}H_{15}NO_2$ [M + H]$^+$ 266.3120 found 266.1369.

2.4.3. Transformations: 1,4-Cyclisations

N,3-diphenyl-1H-pyrazole-5-carboxamide (7a)

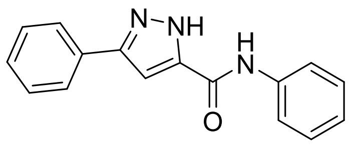

Compound **7a** was obtained following General procedure 6. Flash chromatography (xx% xxx in xx) yielded compound **7a** as a white solid (18.4 mg, 69.9 μmol, 18%).

R_f = 0.24 (CH_2Cl_2); mp = 250-252 °C; UV λ_{max} (EtOH/nm) 263.0, 205.4; FTIR (cm^{-1}) ν 3379 (m, N-H pyrazole), 3125 (m, N-H amide), 2923w, C-H), 2119 (w, C=C), 1657 (C=O amide); ^1H NMR (DMSO-d_6, 500 MHz) δ 7.11 (1H, t, *J* = 7.4 Hz), 7.38 (3H, dt, *J* = 15.7, 7.6 Hz), 7.49 (2H, t, *J* = 7.6 Hz), 7.87 – 7.79 (4H, m), 10.13 (1H, s) ; ^{13}C NMR (DMSO-d_6, 126 MHz) δ 103.51, 120.72, 124.08, 125.79, 128.82, 129.10, 129.48, 132.46, 139.15, 146.96, 148.06, 158.01; MS(ES+) *m/z* 264.2 [M + H]$^+$; HRMS calcd for $C_{16}H_{13}N_3O$ [M + H]$^+$ 264.1131, found 264.1132.

***N*-(3,4-dimethoxyphenyl)-3-(4-methoxyphenyl)-1*H*-pyrazole-5-carboxamide (7b)**

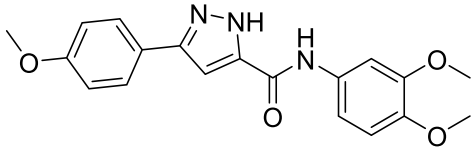

Compound **7b** was obtained following General procedure 6. Flash chromatography (30 to 70% EtOAc in PE) yielded compound **7b** as a white solid (54.1 mg, 0.153 mmol, 52%).

R_f = 0.55 (70% EtOAc in PE); mp = 225-226 °C; UV λ_{max} (EtOH/nm) = 271.0, 202.8; FTIR (cm^{-1}) ν 3387 (m, N-H pyrazole), 3127 (m, N-H amide), 2835 (w, C-H), 2112 (w, C=C), 1649 (s, C=O); ^1H NMR (DMSO-d_6, 500 MHz): δ_H = 3.74 (3H, s), 3.76 (3H, s), 3.81 (3H, s), 6.93 (1H, d, *J* = 8.8 Hz), 7.05 (2H, d, *J* = 8.9 Hz), 7.15 (1H, s), 7.40 (1H, dd, *J* = 8.7, 2.4 Hz), 7.51 (1H, d, *J* = 2.4 Hz), 7.77 (2H, d, *J* = 8.8 Hz), 9.95 (1H, s); ^{13}C NMR (DMSO-d_6, 126 MHz): δ_C = 55.67, 55.86, 56.17, 102.67, 105.88, 112.37, 112.61, 114.86, 123.05, 127.22, 132.73, 145.53, 145.99, 146.48, 148.92, 159.66, 159.79; MS(ES+) *m/z* 354.4 [M + H]$^+$; HRMS calcd for $C_{19}H_{19}N_3O_4$ [M + H]$^+$ 354.1448, found 354.1461.

***N*,3-Diphenylisoxazole-5-carboxamide (8a)**

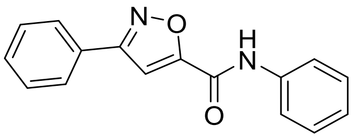

Compound **8a** was obtained following General procedure 7. Flash chromatography (0 to 40% EtOAc in PE) yielded compound **8a** as a white solid (14 mg, 0.05 mmol, 13%).

R_f = 0.67 (60% EtOAc in PE); mp = 180-182 °C; UV λ_{max} (EtOH/nm) 268.6, 203.7; FTIR (cm^{-1}) ν 3343 (m, N-H amide), 1675 (s, C=O amide), 1595 (s, C=C aromatic), 1314 (s, C-N aromatic), 1243 (s, C-O aromatic); ^1H NMR (Chloroform-*d*, 500 MHz) δ 7.06 (1H, s), 7.19 (1H, t, *J* = 7.5 Hz), 7.40 (2H, t, *J* = 7.8 Hz), 7.46 – 7.55 (3H, m), 7.69 (2H, d, *J* = 7.9 Hz), 7.83 (2H, dd, *J* = 7.4, 2.3 Hz), 8.58 (1H, s); ^{13}C NMR (Chloroform-*d*, 126 MHz) δ 99.37, 120.19, 125.18, 126.13, 126.80, 129.33, 129.36, 131.03, 137.09, 156.79, 159.56, 172.23; MS(ES+) *m/z* 265.2 [M + H]$^+$; HRMS calcd for $C_{16}H_{12}N_2O_2$ [M + H]$^+$ 264.0972, found 265.0901.

***N*,5-Diphenylpyrazolo[1,5-*a*]pyrimidine-7-carboxamide (9a)**

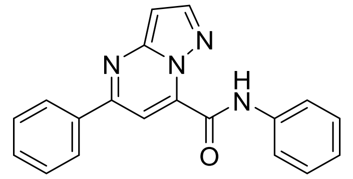

Compound **9a** was obtained following General procedure 8. Flash chromatography (10 to 50% EtOAc in PE) yielded compound **9a** as a yellowish solid (80 mg, 0.25 mmol, 64%).

R_f = 0.63 (50% EtOAc in PE); mp = 172-174 °C; UV λ_{max} (EtOH/nm) 321.8, 272.0, 201.3; FTIR (cm^{-1}) ν 3032 (m, N-H amide), 1680 (s, C=O amide), 1602 (s, C=C aromatic), 1562 (s, C=C aromatic); ^{1}H NMR (Chloroform-*d*, 500 MHz) δ 6.90 (1H, d, *J* = 2.5 Hz), 7.22 (1H, t, *J* = 7.5 Hz), 7.43 (2H, t, *J* = 7.8 Hz), 7.50 – 7.55 (3H, m), 7.85 (2H, d, *J* = 8.0 Hz), 8.17 – 8.23 (2H, m), 8.25 (1H, d, *J* = 2.5 Hz), 8.39 (1H, s), 12.73 (1H, s); ^{13}C NMR (Chloroform-*d*, 126 MHz) δ 98.10, 108.40, 121.02 (C_{15} and C_{19}), 125.51, 127.59 (C_4 and C_6), 129.20 (C_1 and C_3), 129.31 (C_{16} and C_{18}), 131.06, 136.68, 137.29, 137.60, 144.03, 150.32, 156.85, 156.87, 156.97 (C_7). MS(ES+) *m/z* 315.2 [M + H]$^+$; HRMS calcd for $C_{19}H_{14}N_4O$ [M + H]$^+$ 315.1241, found 315.1127.

***N*-(3,4-dimethoxyphenyl)-5-(4-methoxyphenyl)pyrazolo[1,5-*a*]pyrimidine-7-carboxamide (9b)**

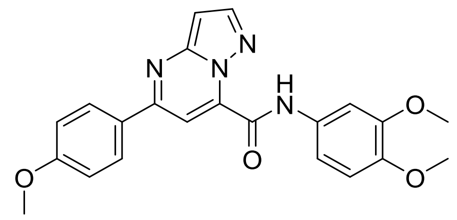

Compound **9b** was obtained following General procedure 8. Flash chromatography (0 to 10 MeOH in EtOAc) yielded compound **9b** as a yellowish solid (69.9 mg, 0.173 mmol, 59%).

R_f = 0.16 (CH_2Cl_2); mp = 195-197 °C; UV λ_{max} (EtOH/nm) 293.8 FTIR (cm^{-1}) ν 3061 (w, C-H), 2920 (w, C-H), 2843 (w, C-H), 1674 (s, C=O), 1600 (s, C=N); ^{1}H NMR (Chloroform-*d*, 500 MHz): δ_H = 3.84 (3H, s), 3.85 (3H, s), 3.91 (3H, s), 6.80 (1H, d, *J*=2.5 Hz), 6.84 (1H, d, *J* = 8.6 Hz), 6.98 (2H, d, *J* = 8.8 Hz), 7.21 (1H, d, *J* = 2.4 Hz), 7.59 (1H, d, *J* = 2.5 Hz), 8.12 (2H, d, *J* = 8.9 Hz), 8.17 (1H, d, *J* = 2.5 Hz), 8.28 (1H, s), 12.64 (1H, s)^{13}C NMR (Chloroform-*d*, 126 MHz): δ_C = 55.51, 56.03, 56.13, 97.49, 105.26, 107.74, 111.27, 112.97, 114.49, 129.01, 129.11, 131.08, 137.09, 143.81, 146.62, 150.16, 156.37, 156.75, 161.45, 162.10; MS(ES+) *m/z* 405.3; [M + H]$^+$; HRMS calcd for $C_{22}H_{20}N_4O_4$ [M + H]$^+$ 405.1557, found 405.1573

***N*,7-Diphenylimidazo[1,2-*a*]pyridine-5-carboxamide (10a)**

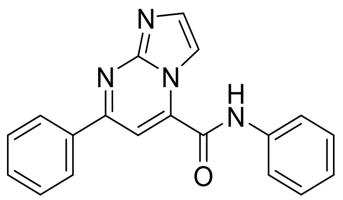

Compound **10a** was obtained following General procedure 9. Flash chromatography (10 to 50% EtOAc in PE) yielded compound **10a** as a yellowish solid (120 mg, 0.38 mmol, 95%).

R_f = 0.53 (60% EtOAc in PE); mp = 115-117 °C; UV λ_{max} (EtOH/nm) 339.4, 319.2, 268.6, 201.4; FTIR (cm^{-1}) ν 1675 (s, C=O amide), 1596 (s, C=C aromatic), 1520 (s, C=C aromatic); ^{1}H NMR (Chloroform-*d*, 500 MHz) δ 7.18 (2H, t, *J* = 7.7 Hz), 7.24 (1H, t, *J* = 7.5 Hz), 7.30 (1H, t, *J* = 7.3 Hz), 7.42 (2H, t, *J* = 7.9 Hz), 7.45 (1H, d, *J* = 1.5 Hz), 7.54 (1H, s), 7.71 (2H, d, *J* = 7.5 Hz), 7.88 (1H, d, *J* = 1.5 Hz), 7.91 (2H, d, *J* = 8.0 Hz), 11.10 (1H, s); ^{13}C NMR (Chloroform-*d*, 126 MHz) δ 105.52, 112.42, 121.23, 125.62, 127.10, 128.82, 129.25, 130.92, 134.90, 135.75, 137.78, 148.92, 155.94, 160.11, 161.94; MS(ES+) *m/z* 315.2 [M + H]$^+$; HRMS calcd for $C_{19}H_{14}N_4O$ [M + H]$^+$ 315.1241, found 315.1163.

***N*-(3,4-dimethoxyphenyl)-7-(4-methoxyphenyl)imidazo[1,2-*a*]pyrimidine-5-carboxamide (10b)**

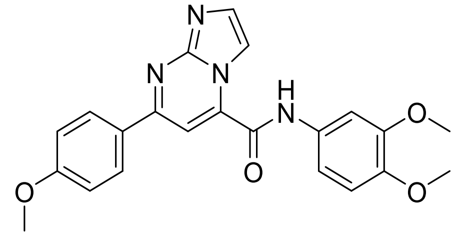

Compound **10b** was obtained following General procedure 9. Flash chromatography (0 to 10% MeOH in DCM) yielded compound **10b** as an orange solid (60.7 mg, 0.150 mmol, 51%).

R_f = 0.39 (5% MeOH in CH2Cl2); mp = 237-240 °C; UV λ_{max} (EtOH/nm) 266.8, 200.6; FTIR (cm^{-1}) ν 2931 (w, C-H), 1675 (s, C=O), 1600 (s, C=C), 1560 (s, C=O), 1510 (s, C=O); ^{1}H NMR (DMSO-d_6, 500 MHz) δ 3.77 (3H, s), 3.78 (3H, s), 3.86 (3H, s), 7.01 (1H, d, *J* = 8.7 Hz), 7.16 (2H, d, *J* = 9.0 Hz), 7.38 (1H, dd, *J* = 8.7, 2.4 Hz), 7.44 (1H, d, *J* = 2.4 Hz), 7.82 (1H,d, *J* = 1.5 Hz), 8.20 (1H, s), 8.34 – 8.26 (2H, m), 10.92 (1H, s); ^{13}C NMR (DMSO-d_6, 126 MHz) δ 55.93, 56.00, 56.18, 105.70, 106.21, 112.34, 113.48, 114.92, 129.34, 131.69, 136.06, 137.84, 146.54, 149.03, 149.49, 155.17, 159.82, 162.01, 162.81; MS(ES+) *m/z* 405.2 [M + H]$^+$; HRMS calcd for $C_{22}H_{20}N_4O_4$ [M + H]$^+$ 405.1557, found 405.1561.

Methyl carbamimidate hydrochloride (11')

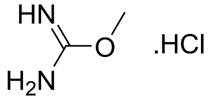

In a RB-flask, 5 mL of anhydrous MeOH under inert atmosphere was cooled in an ice bath. Acetyl chloride (1.98 mL, 27.8 mmol) was added dropwise then stirred at 0 °C for 20 min. A solution of cyanamide (1.00 g, 23.79 mmol) dissolved in 3 mL of anhydrous MeOH was prepared and cooled in an ice bath. To this, the methanolic hydrochloride solution was added dropwise. The mixture was warmed to rt then stirred overnight at rt under inert atmosphere. The solvent was removed under vacuum and the product was dried in the vacuum oven overnight. This yielded **11'** as a white solid (2.54 g, 22.98 mmol, 99%).

R_f = 0.36 (10% MeOH in CH_2Cl_2); mp = 95-97 °C; UV λ_{max} (EtOH/nm) 230.0, 203.6; FTIR (cm^{-1}) ν 3025 (b, s, N-H amine), 1680 (s, C=N), 1573 (s, N-H amine), 1452 (s, C-H methoxy), 1290 (s, C-N amine), 1207 (C-N); ^{1}H NMR (DMSO-d_6, 500 MHz) δ 3.95 (3H, s); ^{13}C NMR (DMSO-d_6, 126 MHz) δ 57.66, 162.67.

NMR data from literature:[123] ^{1}H NMR (300 MHz, D_2O) δ 4.04 (s, 3H); ^{13}C NMR (100 MHz, D_2O) δ 57.4, 162.9.

2-Methoxy-*N*,6-diphenylpyrimidine-4-carboxamide (11a)

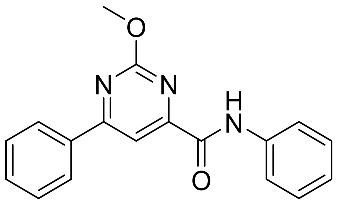

Compound **11a** was obtained following General procedure 10. Flash chromatography (50 to 20% EtOAc in PE) yielded compound **11a** as a yellow solid (40 mg, 0.13 mmol, 25%).

R_f = 0.27 (20% EtOAc in PE); mp = 145-146 °C; UV λ_{max} (EtOH/nm) 314.2, 240.4, 200.0; FTIR (cm^{-1}) ν 3348 (m, N-H amide), 1681 (s, C=O amide), 1580 (s, C=C aromatic), 1520 (s, C=C aromatic); ^{1}H NMR (Chloroform-*d*, 500 MHz) δ 4.22 (3H, d, *J* = 2.1 Hz, H_{23}), 7.19 (1H, t, *J* = 7.4 Hz, H_{17}), 7.41 (2H, t, *J* = 7.9 Hz), 7.49 – 7.59 (3H, m), 7.78 (2H, d, *J* = 7.9 Hz), 8.24 (2H, dd, *J* = 7.8, 1.0 Hz), 8.32 (1H, s), 9.75 (1H, s); ^{13}C NMR (Chloroform-*d*, 126 MHz) δ 55.48, 108.16, 120.12, 125.07, 127.72, 129.16, 129.32, 131.99, 135.98, 137.24, 159.86, 160.62, 165.24, 169.10; MS(ES+) *m/z* 306.2 [M + H]$^+$; HRMS calcd for $C_{18}H_{15}N_3O_2$ [M + H]$^+$ 306.1237, found 306.1152.

***N*-(3,4-Dimethoxyphenyl)-2-methoxy-6-(4-methoxyphenyl)pyrimidine-4-carboxamide (11b)**

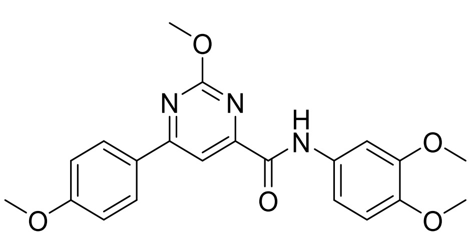

Compound **11b** was obtained following General procedure 10. Normal phase flash chromatography (30 to 50% EtOAc in PE) yielded compound **11b** as a yellow solid (18 mg, 0.05 mmol, 76%).

R_f = 0.53 (60% EtOAc in PE); mp = 156-157 °C; UV λ_{max} (EtOH/nm) 328.8, 264.2, 237.2; FTIR (cm^{-1}) ν 3341 (m, N-H amide), 1677 (s, C=O amide), 1581 (s, C=C aromatic), 1508 (s, C=C aromatic); ^{1}H NMR (Chloroform-*d*, 500 MHz) δ 3.90 (3H, s), 3.90 (3H, s), 3.95 (3H, s), 4.20 (3H, s), 6.89 (1H, d, *J* = 8.6 Hz), 7.03 (2H, dt, *J* = 9.0, 2.9, 2.0 Hz), 7.20 (1H, dd, *J* = 8.6, 2.4 Hz), 7.58 (1H, d, *J* = 2.4 Hz), 8.22 (2H, dd, *J* = 6.7, 2.0 Hz), 8.24 (1H, s), 9.69 (1H, s); ^{13}C NMR (Chloroform-*d*, 126 MHz) δ 55.41, 55.65, 56.13, 56.29, 104.70, 107.23, 111.60, 112.09, 114.55, 128.49, 129.46 , 130.99, 146.45, 149.35, 159.58, 160.58, 162.97, 165.16, 168.57; MS(ES+) *m/z* 396.4 [M + H]$^+$; HRMS calcd for $C_{21}H_{21}N_3O_5$ [M + H]$^+$ 396.1557, found 396.1561.

2-Oxo-*N*,6-diphenyl-2,3-dihydropyrimidine-4-carboxamide (12a)

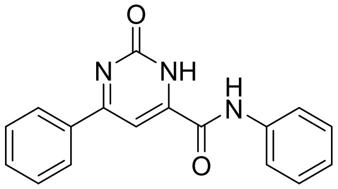

Compound **12a** was obtained following General procedure 11. Flash chromatography (20 to 60% EtOAc in PE) yielded compound **12a** as a beige solid (2.3 mg, 0.01 mmol, 20%).

R_f = 0.67 (80% MeOH in CH_2Cl_2); mp = 228-230 °C, UV λ_{max} (EtOH/nm) 347.2, 308.4, 253.0, 200.0; FTIR (cm^{-1}) ν 3325 (m, N-H amide), 1644 (s, C=O amide), 1600 (s, C=C aromatic), 1525 (s, C=C aromatic); ^{1}H NMR (Chloroform-*d*, 500 MHz) δ 7.20 (1H, tt, *J* = 7.3, 1.1 Hz), 7.41 (2H, t, *J* = 8.0 Hz), 7.66 (3H, dd, *J* = 5.2, 1.9 Hz), 7.71 (1H, s), 7.79 (2H, d, *J* = 7.9 Hz), 7.98 – 8.05 (2H, m), 9.84 (1H, s), 13.07 (1H, s); ^{13}C NMR (Chloroform-*d*, 126 MHz) δ 100.66, 120.14, 125.28, 127.59, 129.35, 130.01, 130.68, 133.25, 137.12, 159.43, 159.76, 160.45, 165.40; MS(ES+) *m/z* 292.2 [M + H]$^+$; HRMS calcd for $C_{17}H_{13}N_3O_2$ [M + H]$^+$ 292.1084, found 292.1202.

***N*-(3,4-dimethoxyphenyl)-6-(4-methoxyphenyl)-2-oxo-2,3-dihydropyrimidine-4-carboxamide (12b)**

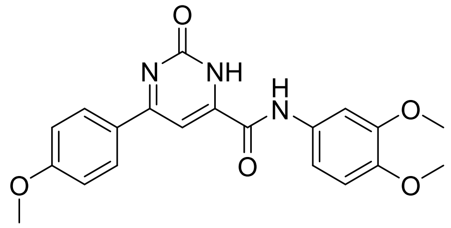

Compound **12b** was obtained following General procedure 11. Flash chromatography (0 to 10% MeOH in DCM) yielded compound **12b** as an orange solid (30.3 mg, 79.4 µmol, 26%).

R_f = 0.51 (10% MeOH in CH_2Cl_2); mp = 238-240 °C; UV λ_{max} (EtOH/nm) 352.0, 203.8; FTIR (cm^{-1}) ν 2917 (m, C-H), 2847 (w, C-H), 2118 (w, C=C), 1650 (s, C=O amide), 1601 (s, C=O pyridine); ^{1}H NMR (DMSO-d_6, 500 MHz): δ 3.76 (3H, s), 3.77 (3H, s), 3.87 (3H, s), 6.96 (1H, d, *J* = 8.7 Hz), 7.13 (2H, d, *J* = 8.7 Hz), 7.48 (1H, dd, *J* = 8.7, 2.4 Hz), 7.57 (1H, d, *J* = 2.4 Hz), 8.10 (2H, d, *J* = 8.4 Hz), 10.40 (1H, s); ^{13}C NMR (DMSO-d_6, 126 MHz): δ 55.88, 55.99, 56.13, 105.83, 108.52, 112.26, 112.76, 114.88, 114.98, 129.83, 131.77, 140.42, 145.26, 146.12, 148.96, 161.13, 162.89, 181.44; MS(ES+) *m/z* 382.2 [M + H]$^+$; HRMS calcd for $C_{20}H_{19}N_3O_5$ [M + H]$^+$ 382.1397, found 382.1409.

2-(4-Methoxybenzyl)isothiouronium chloride (13')

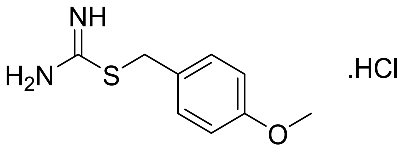

In a RB-flask, thiourea (1.50 g, 19.7 mmol) was dissolved in 10 mL of anhydrous THF under inert atmosphere. Cool down in an ice bath. PMBCl (2.73 mL, 19.7 mmol) was then added dropwise. The mixture was warmed to rt and stirred for an 1h. It was then stirred at 65 °C for 5 h under inert atmosphere. The mixture was filtered and the obtained solid was washed with ether and dried overnight in the vacuum oven. This yielded **13'** as a white powder (4.41 g, 19.01 mmol, 96%).

R_f = 0.24 (5% MeOH in CH_2Cl_2); mp =168-170 °C; UV λ_{max} (EtOH/nm) 229.8, 204.8; FTIR (cm^{-1}) ν 3071 (s, N-H amine), 2942 (s, N-H), 1649 (s, C=N), 1625 (s, N-H amine), 1509 (s, C-H CH_2); ^1H NMR (DMSO-d_6, 500 MHz) δ 3.59 (3H, s), 4.34 (2H, s), 6.78 (2H, d, *J* = 8.6 Hz), 7.21 (2H, d, *J* = 8.6 Hz), 9.18 (4H, s); ^{13}C NMR (DMSO-d_6, 126 MHz) δ 33.91, 55.21, 114.25, 126.72, 130.43, 159.02, 169.42; MS(ES+) *m/z* 197.2 $[M + H]^+$.

NMR data from literature:[123] ^1H NMR (DMSO-d_6, 300 MHz) δ 9.28 (s, 4H), 7.36 (d, 2H, *J* = 8.7 Hz), 6.93 (d, 2 H, *J* = 8.7 Hz), 4.47 (s, 2H), 3.75 (s, 3H).

2-Methoxy-*N*,6-diphenylpyrimidine-4-carboxamide (13a)

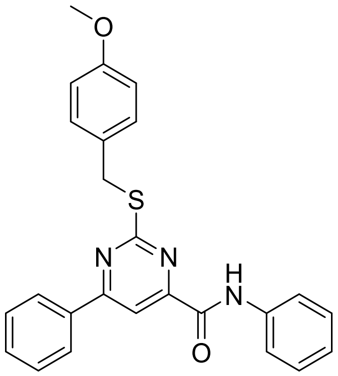

Compound **13a** was obtained following General procedure 12. Flash chromatography (0 to 20% EtOAc in PE) yielded compound **13a** as a yellow solid (30 mg, 0.07 mmol, 18%).

R_f = 0.43 (40% EtOAc in PE); mp = 125-127 °C; UV λ_{max} (EtOH/nm) 268.8, 200.8; FTIR (cm^{-1}) ν 3375 (m, N-H amide), 2927 (m, C-H alkane), 1687 (s, C=O amide), 1602 (s, C=C aromatic), 1572 (s, C=C aromatic), 1508 (s, C=C aromatic); ^1H NMR (Chloroform-*d*, 500 MHz) δ 3.79 (3H, s), 4.55 (2H, s), 6.87 (2H, d, *J* = 8.7 Hz), 7.19 (1H, t, *J* = 7.4 Hz), 7.37 – 7.45 (4H, m), 7.50 – 7.60 (3H, m), 7.73 (2H, d, *J* = 8.0 Hz), 8.22 (2H, dd, *J* = 7.6, 1.2 Hz), 8.29 (1H, s), 9.68 (1H, s); ^{13}C NMR (Chloroform-*d*, 126 MHz) δ 35.38, 55.44, 109.67, 114.29, 120.17, 125.12, 127.73, 129.11, 129.22, 129.32, 130.01, 131.98, 135.97, 137.18, 157.71, 159.12, 160.55, 166.71, 171.57; MS(ES+) *m/z* 428.3 $[M + H]^+$; HRMS calcd for $C_{25}H_{21}N_3O_2S$ $[M + H]^+$ 428.1427, found 428.1429.

2-Amino-*N*,6-diphenylpyrimidine-4-carboxamide (14a)

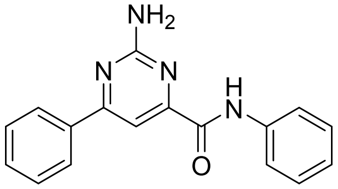

Compound **14a** was obtained following General procedure 13. Flash chromatography (5 to 20% EtOAc in PE) yielded compound **14a** as a yellow solid (24 mg, 0.08 mmol, 21%).

R_f = 0.44 (40% EtOAc in PE); mp = 127-129 °C; UV λ_{max} (EtOH/nm) 339.6, 236.0, 200.0; FTIR (cm^{-1}) ν 3310 (m, N-H amine), 3185 (m, N-H amide), 1683 (s, C=O amide), 1633 (s, C=C aromatic), 1522 (s, C=C aromatic); ^1H NMR (Chloroform-*d*, 500 MHz) δ 5.26 (2H, s), 7.17 (1H, t, *J* = 7.4 Hz), 7.40 (2H, t, *J* = 7.7 Hz), 7.51 (3H, dd, *J* = 5.7, 1.8 Hz), 7.77 (2H, d, *J* = 8.0 Hz), 7.99 (1H, s), 8.09 – 8.15 (2H, m), 9.77 (1H, s); ^{13}C NMR (Chloroform-*d*, 126 MHz) δ 105.35, 119.95 , 124.84, 127.51, 128.26, 129.03, 129.29, 131.38, 136.73, 137.47, 158.43, 161.23, 162.52, 168.31; MS(ES+) *m/z* 291.2 $[M + H]^+$; HRMS calcd for $C_{17}H_{14}N_4O$ $[M + H]^+$ 291.1241, found 291.1157.

2-Amino-*N*-(3,4-dimethoxyphenyl)-6-(4-methoxyphenyl)pyrimidine-4-carboxamide (14b)

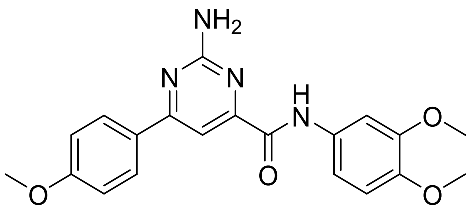

Compound **14b** was obtained following General procedure 13. Flash chromatography (30 to 70% EtOAc in PE) yielded compound **14b** as a yellow oily film (8 mg, 0.02 mmol, 36%).

R_f = 0.23 (40% EtOAc in PE); UV λ_{max} (EtOH/nm) 349.0, 294.4, 240.8, 206.2; FTIR (cm^{-1}) ν 3292 (m, N-H amide), 1539 (s, C-N amine), 1509 (s, C=C aromatic); ^{1}H NMR (Chloroform-*d*, 500 MHz) δ 3.88 (3H, s), 3.90 (3H, s), 3.95 (3H, s), 5.23 (2H, s), 6.88 (1H, d, *J* = 8.6 Hz), 7.01 (2H, dt, *J* = 8.8, 2.8 Hz), 7.14 (1H, dd, *J* = 8.6, 2.5 Hz), 7.63 (1H, d, *J* = 2.5 Hz), 7.93 (1H, s), 8.11 (2H, dt, *J* = 9.0, 2.9, 1.9 Hz), 9.70 (1H, s); ^{13}C NMR (Chloroform-*d*, 126 MHz) δ 55.60, 56.11, 56.29, 104.49, 104.61, 111.60, 111.87, 114.43, 129.07, 129.18, 131.24, 146.29, 149.35, 158.18, 161.10, 162.38, 162.54, 167.56; MS(ES+) *m/z* 381.4 $[M + H]^+$; HRMS calcd for $C_{20}H_{20}N_4O_4$ $[M + H]^+$ 381.1561, found 381.1564.

***N*-Acetonylpyridinium chloride (15')**

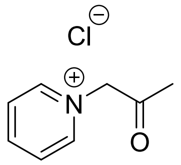

In a RB-flask, pyridine (4.9 mL, 60.8 mmol) was added to 10 mL anhydrous THF under inert atmosphere. Chloroacetone (5.0 mL, 73.0 mmol) was added dropwise. Stirr at rt for 24h under inert atmosphere. Solvent was removed under vacuum. Product was dried in vacuum oven overnight. This yielded **15'** as a white solid (8.50 g, 49.53 mmol, 97%).

R_f = 0.36 (5% MeOH in CH_2Cl_2); mp = 210-212 °C; UV λ_{max} (EtOH/nm) 399.8, 260.2, 204,8; FTIR (cm^{-1}) ν 2931 (m, C-H alkane), 1730 (s, C=O ketone), 1631 (s, C=C aromatic); ^{1}H NMR (DMSO-d_6, 500 MHz) δ 2.32 (3H, s), 5.94 (2H, d, *J* = 3.4 Hz), 8.15 – 8.32 (2H, m), 8.68 (1H, tt, *J* = 7.9, 1.4 Hz), 8.98 (2H, dt, *J* = 6.6, 1.9 Hz); ^{13}C NMR (DMSO-d_6, 126 MHz) δ 27.14, 68.16, 127.66 , 145.92 , 146.14, 199.44 .

NMR data from literature:[141] ^{1}H NMR (DMSO-d_6, 400 MHz) δ ppm 2.35 (s, 3 H), 5.93 (s, 2 H), 8.25 (t, *J* = 6.95 Hz, 2 H), 8.71 (t, *J* = 7.73 Hz, 1 H), 8.99 (d, *J* = 5.87 Hz, 2 H).

2-Methyl-*N*,6-diphenylisonicotinamide (15a)

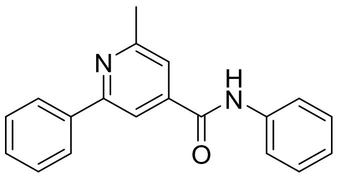

Compound **15a** was obtained following General procedure 14. Normal phase flash chromatography (10 to 30% EtOAc in PE) yielded compound **15a** as an orange solid (118 mg, 0.40 mmol, quant.).

R_f = 0.60 (60% EtOAc in PE); mp = 156-158 °C; UV λ_{max} (EtOH/nm) 253.4, 211.0; FTIR (cm^{-1}) ν 3240 (m, N-H amide), 1649 (s, C=O amide), 1595 (s, C=C aromatic), 1523 (s, C=C aromatic); ^{1}H NMR (Chloroform-*d*, 500 MHz) δ 2.68 (3H, s), 7.19 (1H, t, *J* = 7.5 Hz), 7.38 (2H, t, *J* = 7.8 Hz), 7.39 – 7.50 (3H, m), 7.66 (2H, d, *J* = 7.9 Hz), 7.87 (1H, s), 8.00 (2H, d, *J* = 7.2 Hz), 8.09 (1H, s); ^{13}C NMR (Chloroform-*d*, 126 MHz) δ 24.92, 115.13, 118.77, 120.55, 125.26, 127.22, 128.29, 129.32, 129.48, 137.53, 138.93, 143.48, 158.28, 159.80, 164.67; MS(ES+) *m/z* 289.2 $[M + H]^+$; HRMS calcd for $C_{19}H_{16}N_2O$ $[M + H]^+$ 289.13.36, found 289.1264.

***N*-(3,4-Dimethoxyphenyl)-2-(4-methoxyphenyl)-6-methylisonicotinamide (15b)**

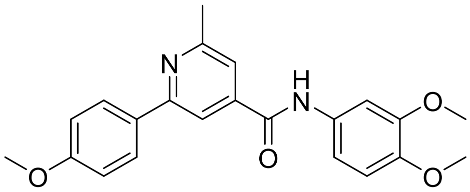

Compound **15b** was obtained following General procedure 14. Flash chromatography (10 to 50% EtOAc in PE) yielded compound **15b** as a beige solid (13 mg, 0.03 mmol, 60%).

R_f = 0.44 (60% EtOAc in PE); UV λ_{max} (EtOH/nm) 269.2, 205.6; FTIR (cm^{-1}) ν 3338 (w, N-H amide), 2928 (m, C-H methyl), 1650 (s, C=O amide), 1604 (s, C=C aromatic), 1508 (s, C=C aromatic); ^{1}H NMR (Chloroform-*d*, 500 MHz) δ 2.68 (3H, s), 3.87 (3H, s), 3.89 (3H, s), 3.92 (3H, s), 6.87 (1H, d, *J* = 8.6 Hz), 7.00 (2H, dt, *J* = 8.9, 3.0, 2.0 Hz), 7.04 (1H, dd, *J* = 8.6, 2.5 Hz), 7.39 (1H, d, *J* = 1.7 Hz), 7.47 (1H, d, *J* = 2.5 Hz), 7.84 – 7.88 (2H, m), 8.00 (2H, dt, *J* = 8.9, 3.0, 2.0 Hz); ^{13}C NMR (Chloroform-*d*, 126 MHz) δ 24.98, 55.53, 56.13, 56.27, 105.38, 111.51, 112.54, 114.29, 114.35, 117.82, 128.55, 131.11, 131.56, 143.39, 146.61, 149.34, 157.95, 159.63, 160.94, 164.54; MS(ES+) *m/z* 379.4 $[M + H]^+$; HRMS calcd for $C_{22}H_{22}N_2O_4$ $[M + H]^+$ 379.1653, found 379.1670.

2.4.4. Transformations: 3,4-Cyclisations

***trans*-2-Benzoyl-*N*-phenylcyclopropane-1-carboxamide (16a)**

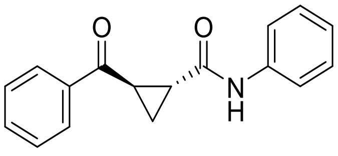

Compound **16a** was obtained following General procedure 15. Normal phase flash chromatography (0 to 10 % EtOAc in PE) yielded compound **16a** as a white solid (27 mg, 0.10 mmol, 51%).

R_f = 0.17 (10 % EtOAc in PE); mp = 139-142 °C; UV λ_{max} (EtOH/nm) = 246.0, 201.0; FTIR (cm^{-1}) ν 3288 (m, N-H amide), 1649 (s, C=O amide), 1597 (s, C=C aromatic); ^{1}H NMR (Chloroform-*d*, 500 MHz) δ 1.62 (1H, td, $^3J_{trans}$ = 5.7, $^2J_{gem}$ = 3.0 Hz), 1.76 (1H, ddd, $^3J_{cis}$ = 9.0, $^3J_{trans}$ = 5.7, $^2J_{gem}$ =3.0 Hz), 2.41 – 2.49 (1H, m), 3.33 (1H, ddd, $^3J_{cis}$ = 9.0, $^3J_{trans}$ = 5.7, $^3J_{trans}$ = 3.7 Hz), 7.10 (1H, t, *J* = 7.5 Hz), 7.31 (2H, t, *J* = 7.9 Hz), 7.47 (2H, t, *J* = 7.8 Hz), 7.54 – 7.61 (3H, m, H_2), 8.04 (2H, dd, *J* = 8.6, 0.8 Hz), 8.45 – 8.49 (1H, m); ^{13}C NMR (Chloroform-*d*, 126 MHz) δ 18.22, 26.11, 27.82, 119.99, 124.47, 128.53, 128.86, 129.12, 133.72, 137.06, 138.13, 169.16, 199.03; MS(ES+) *m/z* 266.2 [M + H]$^+$; HRMS calcd for $C_{17}H_{15}NO_2$ [M + H]$^+$ 266.1176, found 266.1170.

***trans*-*N*-(3,4-Dimethoxyphenyl)-2-(4-methoxybenzoyl)cyclopropane-1-carboxamide (16b)**

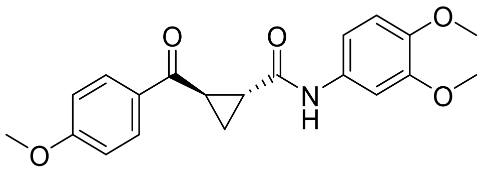

Compound **16b** was obtained following General procedure 11. Normal phase flash chromatography (20 to 50 % EtOAc in PE) yielded compound **16b** as a yellow solid (55.3 mg, 0.156 mmol, 53%).

R_f = 0.61 (50 % EtOAc in PE); mp = 120-122 °C; UV λ_{max} (EtOH/nm) = 270.6, 212.0; FTIR (cm^{-1}) ν 3275 (m, N-H amide), 1646 (s, C=O amide), 1599 (s, C=O ketone), 1510 (s, C=C aromatic); ^{1}H NMR (Chloroform-*d*, 500 MHz) δ 1.59 (1H, ddd, $^3J_{cis}$ = 8.8, $^3J_{trans}$ = 5.7, $^2J_{gem}$ = 3.3 Hz), 1.70 (1H, ddd, $^3J_{cis}$ = 8.8, $^3J_{trans}$ = 5.7, $^2J_{gem}$ = 3.3 Hz), 2.33 (1H, tdd, $^3J_{cis}$ = 8.9, $^3J_{trans}$ = 5.7, *J* = 1.9 Hz), 3.25 (1H, ddd, $^3J_{cis}$ = 9.0, $^3J_{trans}$ = 5.7, $^3J_{trans}$ = 3.8 Hz), 3.85 (3H, s), 3.86 (3H, s), 3.88 (3H, s), 6.80 (1H, d, *J* = 8.6 Hz), 6.89 (1H, dd, *J* = 8.6, 2.5 Hz), 6.95 (2H, dt, *J* = 8.8, 2.7, 2.2 Hz), 7.41 (1H, d, *J* = 2.5 Hz), 7.92 (1H, s), 8.03 (2H, dt, *J* = 8.9, 2.6 Hz); ^{13}C NMR (Chloroform-*d*, 126 MHz) δ 17.76, 25.71, 27.40, 55.68, 56.03, 56.27, 104.93, 111.52, 111.75, 114.05, 130.20, 130.86, 131.79, 146.05, 149.24, 164.09, 169.07, 196.75; MS(ES+) *m/z* 356.4 [M + H]$^+$; HRMS calcd for $C_{20}H_{21}NO_5$ [M + H]$^+$ 356.1496, found 356.1495.

(*rac*)-*trans*-3-Benzoyl-*N*-phenylbicyclo[2.2.1]hept-5-ene-2-carboxamide (17a)

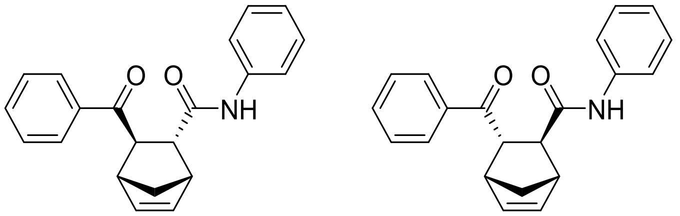

Compound **17a** was obtained following General procedure 16. Normal phase flash chromatography (5 to 15% EtOAc in PE) yielded compound **17a** as an inseparable mixture of isomers a white solid (37 mg, 0.12 mmol, 58%).

R_f = 0.63 (50% EtOAc in PE); UV λ_{max} (EtOH/nm) 243.4, 200.9; mp = 251 - 253 °C; FTIR (cm^{-1}) ν 3339 (m, N-H amide), 2970 (m, C-H alkane) 1655 (s, C=O amide), 1594 (s, C=O ketone), 1532 (s, C=C aromatic); MS(ES+) *m/z* 318.3 [M + H]$^+$; HRMS calcd for $C_{21}H_{19}NO_2$ [M + H]$^+$ 340.1308, found 340.1304.

Diastereoisomer 1 - Major

^{1}H NMR (Chloroform-*d*, 500 MHz) δ 1.54 (1H, d, *J* = 8.4 Hz), 2.19 (1H, d, *J* = 8.4 Hz), 2.90 (1H, d, $^3J_{trans}$ = 4.4 Hz), 3.16 (1H, s), 3.36 (1H, s), 4.28 (1H, t, $^3J_{trans}$ = 4.4 Hz), 5.86 (1H, q, *J* = 2.8 Hz), 6.32 (1H, d, *J* = 2.8 Hz), 7.08 (1H, t, *J* = 7.3 Hz), 7.30 (2H, t, *J* = 7.9 Hz), 7.49 (4H, m), 7.57 (1H, t, *J* = 7.4 Hz), 7.97 (2H, d, *J* = 7.5 Hz); ^{13}C NMR (Chloroform-*d*, 126 MHz)

δ 47.76, 47.83, 48.05, 48.39, 52.79, 119.66 , 124.23, 128.43 , 128.70 , 129.02 , 133.16, 134.12, 136.78, 137.12, 137.78, 172.64, 200.53 .

Diastereoisomer 2 - Minor

^{1}H NMR (Chloroform-*d*, 500 MHz) δ 1.43 – 1.49 (1H, m), 1.69 (1H, d, *J* = 8.7 Hz), 3.12 (1H, d, *J* = 3.1 Hz), 3.31 (1H, s), 3.68 (1H, t, $^3J_{trans}$ = 4.2 Hz), 3.83 (1H, dd, $^3J_{trans}$ = 4.4, *J* = 1.4 Hz), 6.24 (1H, dd, *J* = 5.6, 2.7 Hz), 6.48 (1H, dd, *J* = 5.7, 3.1 Hz), 7.07 (1H, m), 7.29 (2H, m), 7.44 – 7.52 (4H, m), 7.55 (1H, m), 8.05 (2H, d, *J* = 7.7 Hz).

***N*-(3,4-dimethoxyphenyl)-3-(4-methoxybenzoyl)bicyclo[2.2.1]hept-5-ene-2-carboxamide (17b)**

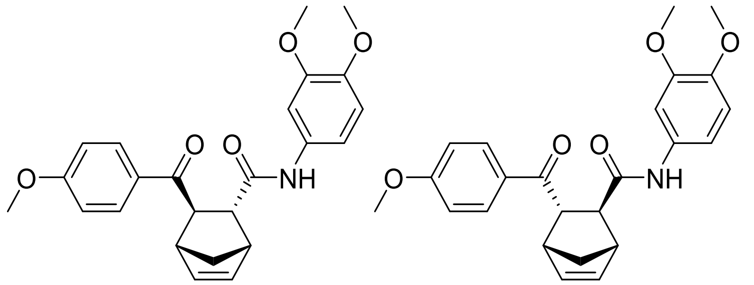

Compound **17b** was obtained following General procedure 16. Flash chromatography (0 to 10% MeOH in CH_2Cl_2) yielded compound **17b** as grey solid (119 mg, 0.293 mmol, 100%).

R_f = 0.61 (4% MeOH in CH_2Cl_2); mp = 158-160 °C; UV λ_{max} (EtOH/nm) = 266.4, 210.0, 203.2; IR: ν_{max} = 3256 (w, N-H amide), 3198 (w, C-H), 3141 (w, C-H), 2970 (w, C-H), 2841 (w, C-H), 1668 (m, C=C), 1649 (s, C=O amide), 1593 (s, C=O ketone); MS(ES+) *m/z* 406.2; [M + H]$^+$; HRMS calcd for $C_{24}H_{25}NO_5$ [M + H]$^+$ 408.1805, found 408.1817.

Major Isomer: ^{1}H NMR (Chloroform-*d*, 500 MHz) δ 1.48 (1H, dq, J = 8.5, 1.8 Hz), 2.15 (1H, d, *J* = 8.3 Hz), 2.86 (1H, dd, *J* = 4.7, 1.6 Hz), 3.09 (1H, d, *J* = 1.1 Hz), 3.29 (1H, p, *J* = 2.0 Hz), 3.78 (3H, s), 3.80 (3H, s), 3.81 (3H, s), 4.18 (1H, dd, *J* = 4.7, 3.4 Hz), 5.79 (1H, dd, *J* = 5.6, 2.8 Hz), 6.24 (1H, dd, *J* = 5.6, 3.1 Hz), 6.73 – 6.69 (1H, m), 6.79 (1H, dd, *J* = 8.6, 2.4 Hz), 6.89 (2H, d, *J* = 8.9 Hz), 7.36 – 7.29 (1H, m), 7.43 (1H, s), 7.93 (2H, d, *J* = 8.9 Hz); ^{13}C NMR (Chloroform-*d*, 126 MHz) δ 47.648.2, 48.2, 48.4, 52.3, 55.5, 55.9, 56.1, 104.5, 111.2, 111.4, 113.9, 129.6, 130.8, 131.8, 134.0, 137.8, 145.6, 149.0, 163.6, 172.7, 198.4, 1.63 (d, J = 8.6 Hz, 1H), 3.04 (s, 1H), 3.20 (s, 1H),

Minor Isomer: ^{1}H NMR (Chloroform-*d*, 500 MHz) δ 1.40 (1H, dd, *J* = 8.6, 1.5 Hz), 3.57 (1H, dd, *J* = 4.7, 3.5 Hz), 3.65 (1H, dd, *J* = 4.8, 1.2 Hz), 6.20 (1H, dd, *J* = 5.6, 2.8 Hz), 6.44 (1H, dd, *J* = 5.6, 3.1 Hz), 6.74 – 6.69 (1H, m), 6.88 (2H, d, *J* = 9.1 Hz), 7.36 – 7.30 (1H, m), 7.97 (2H, d, *J* = 8.9 Hz); ^{13}C NMR (Chloroform-*d*, 126 MHz) δ 47.0, 47.6, 48.4, 49.1, 50.6, 55.5, 55.9, 56.1, 104.5, 111.2, 111.4, 113.9, 129.6, 131.0, 131.8, 135.1, 137.1, 145.6, 149.0, 163.6, 172.7, 198.4.

2-(3,4-dimethoxyphenyl)-3-(4-methoxyphenyl)-5,6-dimethyl-2,3,4,7-tetrahydro-1*H*-isoindol-1-one (18b)

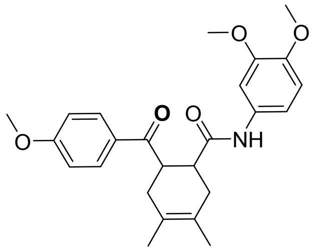

Compound **18b** was obtained following General procedure 17. Flash chromatography (10 to 30% EtOAc in PE) yielded compound **18b** as grey solid (20.9 mg, 49.3 µmol, 17%).

R_f = 0.46 (50% EtOAc in PE); mp = 77-79 °C; UV λ_{max} (EtOH/nm) 266.6; FTIR (cm^{-1}) ν 3317 (br, N-H), 2910 (w, C-H), 2839 (w, C-H), 1662 (m, C=C), 1600 (s, C=O), 1512 (s, C=O); ^{1}H NMR (Chloroform-*d*, 500 MHz) δ 1.65 (3H, s), 1.71 (3H, s), 2.12 (1H, t, *J* = 14.7 Hz), 2.27 (1H, s), 2.30 (1H, s), 2.59 (1H, t, *J* = 14.7 Hz), 2.97 (1H, td, *J* = 11.3, 5.4 Hz), 3.82 (3H, s), 3.84 (3H, s), 3.88 (3H, s), 3.93 (1H, td, *J* = 11.5, 5.6 Hz), 6.75 (1H, d, *J* = 8.6 Hz), 6.82 (1H, dd, *J* = 8.6, 2.4 Hz), 6.94 (2H, d, *J* = 9.0 Hz), 7.23 (1H, d, *J* = 2.4 Hz), 7.53 (1H, s), 8.00 (1H, d, *J* = 9.0 Hz); ^{13}C NMR (Chloroform-*d*, 126 MHz) δ 18.64, 18.72, 34.90, 36.25, 44.41, 44.80, 55.49, 55.83, 56.11, 104.88, 111.25, 111.78, 113.83, 123.90, 124.51, 129.24, 130.97, 131.65, 145.66, 148.91, 163.76, 173.13, 202.24; MS(ES+) *m/z* 424.4 [M + H]$^+$; HRMS calcd for $C_{25}H_{29}NO_5$ [M + H]$^+$ 424.2118, found 424.2102.

5,6-Dimethyl-2,3-diphenyl-2,3,4,7-tetrahydro-1*H*-isoindol-1-one (19a)

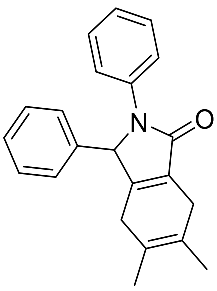

Compound **19a** was obtained following General procedure 17. Normal phase flash chromatography (0 to 10% MeOH in CH_2Cl_2) yielded compound **19a** as white crystals (34 mg, 0.11 mmol, 41%).

R_f = 0.37 (10% MeOH in CH_2Cl_2); mp = 229-231 °C; UV λ_{max} (EtOH/nm) = 200.3; FTIR (cm^{-1}) ν 2855 (w, C-H alkane), 1672 (s, C=O amide), 1596 (s, C=C aromatic), 1494 (s, C=C aromatic), 1452 (m, C-H methyl), 1358 (s, C-N amide), 1121 (s, C-N amide); ^{1}H NMR (Chloroform-*d*, 500 MHz) δ 1.65 (3H, s), 1.75 (3H, s), 2.45 (1H, dt, *J* = 22.6, 7.9 Hz), 2.83 (1H, dt, *J* = 22.6, 7.9 Hz), 2.96 (2H, d, *J* = 7.9 Hz), 5.45 (1H, s), 7.02 (1H, t, *J* = 7.4 Hz), 7.18 (2H, d, *J* = 7.5 Hz), 7.21 – 7.29 (3H, m), 7.31 (2H, t, *J* = 7.4 Hz), 7.55 (2H, d, *J* = 8.1 Hz); ^{13}C NMR (Chloroform-*d*, 126 MHz) δ 18.79, 18.87, 28.86, 31.33, 67.64, 120.99, 121.81, 123.55, 124.06, 126.87, 128.40, 128.87, 129.12, 129.22, 136.18, 138.13, 152.29, 170.70; MS(ES+) *m*/*z* 316.3 [M + H]$^+$; HRMS calcd for $C_{22}H_{21}NO$ [M + H]$^+$ 316.1696, found 316.1683.

4-Benzoyl-*N*-phenyl-1*H*-pyrrole-3-carboxamide (20a)

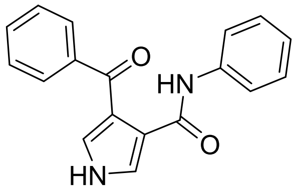

Compound **20a** was obtained following General procedure 18. Normal phase flash chromatography (20 to 80% EtOAC in PE) yielded compound **20a** as a beige crystalline solid (75 mg, 0.26 mmol, 65%).

R_f = 0.35 (60% EtOAc in PE); mp = 157-159 °C; UV λ_{max} (EtOH/nm) 247.2, 202.3; FTIR (cm^{-1}) ν 3116 (m, N-H amide), 2940 (m, N-H pyrrole), 1611 (s, C=C amide), 1557 (s, C=C ketone), 1500 (s, C=C aromatic), 1378 (s, C-N aromatic), 1317 (s, C-N amide), 1167 (s, C-N amide), 1087 (s, C=C aromatic); ^{1}H NMR (Chloroform-*d*, 500 MHz) δ 6.91 (1H, t, *J* = 7.4 Hz), 7.06 (1H, d, *J* = 2.2 Hz), 7.16 (2H, t, *J* = 7.8 Hz), 7.31 (2H, t, *J* = 7.6 Hz), 7.41 (1H, t, *J* = 7.4 Hz), 7.53 (1H, d, *J* = 2.2 Hz), 7.54 – 7.58 (2H, m), 7.60 (2H, d, *J* = 8.0 Hz); ^{13}C NMR (Chloroform-*d*, 126 MHz) δ 119.91, 120.20, 120.88, 123.77, 128.10, 128.63, 128.86, 131.75, 133.24, 138.48, 139.85, 162.45, 194.58; MS(ES+) *m*/*z* 291.2 [M + H]$^+$; HRMS calcd for $C_{18}H_{14}N_2O_2$ [M + H]$^+$ 291.1128, found 291.1132.

***N*-(3,4-dimethoxyphenyl)-4-(4-methoxybenzoyl)-1*H*-pyrrole-3-carboxamide (20b)**

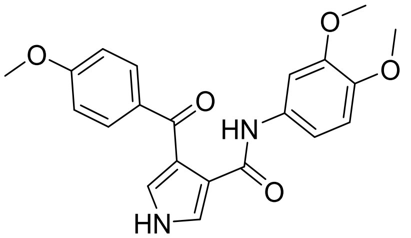

Compound **20b** was obtained following General procedure 18. Flash chromatography (0 to 10% MeOH in CH_2Cl_2) yielded compound **20b** as a yellow solid (82.2 mg, 0.216 mmol, 75%).

R_f = 0.47 (10% MeOH in CH_2Cl_2); mp = 191-193 °C; UV λ_{max} (EtOH/nm) 271.2, 202.8; FTIR (cm^{-1}) ν 3264 (s, N-H amide), 3067 (w, N-H pyrrole), 3004 (w, C-H), 2950 (w, C-H), 1661 (m, C=O, amide), 1592 (s, C=O ketone), 1562 (s, C=N); ^{1}H

NMR (Chloroform-*d*, 500 MHz) δ 3.87 (3H, s), 3.89 (3H, s), 3.90 (3H, s), 6.83 (1H, d, *J* = 8.7 Hz), 6.96 (2H, d, *J* = 8.8 Hz), 7.21 (1H, t, *J* = 2.6 Hz), 7.28 (1H, dd, *J* = 8.7, 2.4 Hz), 7.54 (1H, d, *J* = 2.4 Hz), 7.83 – 7.75 (3H, m), 11.20 (1H, s), 12.40 (s, 1H); ^{13}C NMR (Chloroform-*d*, 126 MHz) δ 55.5, 55.9, 56.1, 105.4, 111.4, 112.8, 113.7, 120.4, 128.2, 131.7, 132.1, 145.7, 149.0, 162.6, 163.1, 193.1; MS(ES+) *m/z* 381.2 [M + H]$^+$; HRMS calcd for $C_{21}H_{20}N_2O_5$ [M + H]$^+$ 381.1445, found 381.1429.

5-Benzoyl-1-benzyl-*N*-phenyl-1*H*-1,2,3-triazole-4-carboxamide (21a)

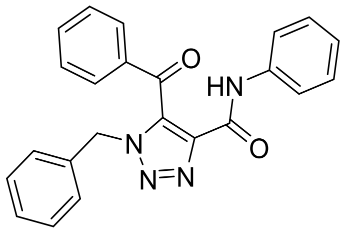

Compound **21a** was obtained following General procedure 19. Normal phase flash chromatography (0 to 10% EtOAc in PE) yielded compound **21a** as white crystals (5 mg, 0.01 mmol, 16%).

R_f = 0.27 (10% EtOAc in PE); mp = 160-162 °C; UV λ_{max} (EtOH/nm) v 258.8, 200.5; FTIR (cm^{-1}) ν 3028 (m, N-H amide), 2923 (m, C-H alkane), 1680 (s, C=O amide), 1622 (s, C=O ketone), 1596 (s, C=C aromatic), 1564 (s, C=C aromatic); ^{1}H NMR (Chloroform-*d*, 500 MHz) δ 6.21 (2H, s), 7.19 (1H, tt, *J* = 7.4, 1.1 Hz), 7.29 – 7.37 (3H, m), 7.40 (2H, tt, *J* = 8.5, 7.4, 1.9 Hz), 7.45 – 7.51 (2H, m), 7.54 (2H, t, *J* = 8.0 Hz), 7.67 (1H, tt, *J* = 8.0, 1.1 Hz$_2$), 7.78 (2H, dd, *J* = 8.2, 1.0 Hz), 8.21 (2H, dd, *J* = 8.5, 1.1 Hz), 12.05 (1H, s); ^{13}C NMR (Chloroform-*d*, 126 MHz) δ 54.46, 120.82, 125.38, 128.55, 128.72, 128.80, 128.97, 129.28, 131.65, 134.22, 134.31, 135.13, 136.69, 137.56, 142.96, 154.75, 190.66; MS(ES+) *m/z* 383.3 [M + H]$^+$; HRMS calcd for $C_{23}H_{18}N_4O_2$ [M + H]$^+$ 383.1503, found 383.1484.

1-benzyl-N-(3,4-dimethoxyphenyl)-4-(4-methoxybenzoyl)-1H-1,2,3-triazole-5-carboxamide (21b) HLS-681-242

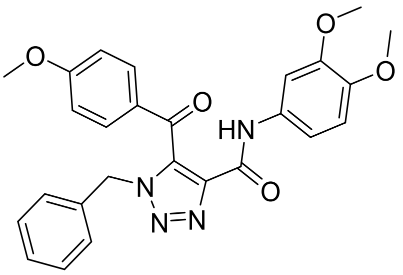

Compound **21b** was obtained following General procedure 19. Flash chromatography (0 to 10% MeOH in CH_2Cl_2) yielded compound **21b** as a brown solid (26.1 mg, 55.2 μmol, 19%).

R_f = 0.24 (CH_2Cl_2); mp = 121-122 °C; UV λ_{max} (EtOH/nm) v 303.2, 202.4; FTIR (cm^{-1}) ν 2924 (m, C-H), 2852 (m, C-H), 1674 (s, C=O amide), 1594 (s, C=O ketone), 1564 (s, C=C aromatic); ^{1}H NMR (DMSO-*d*$_6$, 500 MHz) δ 3.82 (3H, s), 3.85 (3H, s), 3.88 (3H, s), 6.13 (2H, s), 6.80 (1H, d, *J* = 8.7 Hz), 6.94 (1H, d, *J* = 9.0 Hz), 7.21 (1H, dd, *J* = 8.6, 2.5 Hz), 7.30 – 7.24 (3H, m), 7.34 (1H, d, *J* = 2.4 Hz), 7.41 (2H, dd, *J* = 8.0, 1.6 Hz), 8.21 (1H, d, *J* = 9.0 Hz), 11.98 (1H, s); ^{13}C NMR (Chloroform-*d*, 126 MHz) δ 54.17, 55.66, 56.07, 56.11, 105.12, 111.30, 112.92, 113.81, 128.52, 128.65, 128.80, 129.23, 130.99, 133.94, 134.28, 135.14, 143.13, 146.50, 149.12, 154.57, 164.68, 188.33; MS(ES+) *m/z* 473.3 [M + H]$^+$; HRMS calcd for $C_{26}H_{24}N_4O_5$ [M + H]$^+$ 473.1819 found 473.1806.

4-Benzoyl-*N*-phenyl-1*H*-1,2,3-triazole-5-carboxamide (22a)

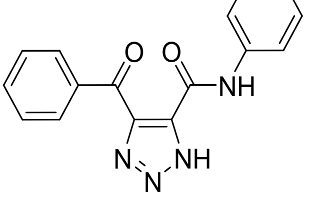

Compound **22a** was obtained following General procedure 20. Normal phase flash chromatography (0 to 10% MeOH in EtOAc) yielded **22a** as a beige solid (21 mg, 0.07 mmol, 36%).

R_f = 0.18 (5% MeOH in CH_2Cl_2); mp = 201-203 °C; UV λ_{max} (EtOH/nm) 252.4, 200.6; FTIR (cm^{-1}) ν 3395 (b, m, N-H amide), 2923 (m, N-H triazole), 1620 (s, C=O amide), 1595 (s, C=C aromatic), 1568 (s, C=C aromatic), 1397 (s, C-N aromatic); ^{1}H NMR (Methanol-*d*$_4$, 500 MHz) δ 7.09 (1H, t, *J* = 7.4 Hz), 7.32 (2H, t, *J* = 7.8 Hz), 7.49 (2H, t, *J* = 7.6 Hz), 7.61 (1H, t, *J* = 7.4 Hz), 7.76 (2H, d, *J* = 7.9 Hz), 8.11 (2H, d, *J* = 7.7 Hz); ^{13}C NMR (Methanol-*d*$_4$, 126 MHz) δ 121.43, 125.52, 129.22, 130.00 , 131.89, 134.21, 139.46, 139.63, 142.90, 144.15, 161.30, 192.72; MS(ES+) *m/z* 293.2 [M - H]$^+$; HRMS calcd for $C_{16}H_{12}N_4O_2$ [M + H]$^+$ 293.1033, found 293.1024.

***N*-(3,4-dimethoxyphenyl)-4-(4-methoxybenzoyl)-1*H*-1,2,3-triazole-5-carboxamide (22b)**

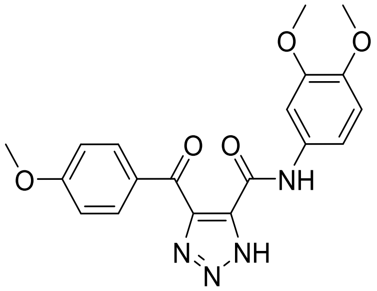

Compound **22b** was obtained following General procedure 20. Normal phase flash chromatography (0 to 10% MeOH in EtOAc) yielded **22b** as a beige solid (23.0 mg, 60.2 μmol, 21%).

R_f = 0.18 (10% MeOHc in CH_2Cl_2); mp = 216-218 °C; UV λ_{max} (EtOH/nm) 300.8, 200.2 FTIR (cm^{-1}) 3127 (w, N-H), 2918 (w, C-H), 2849 (w, C-H), 1628 (s, C=O), 1574 (s, C=O), 1511 (s, C=C); ^{1}H NMR (DMSO-*d*$_6$, 500 MHz) δ 3.74 (6H, s), 3.87 (3H, s), 6.93 (1H, d, *J* = 8.7 Hz), 7.11 (2H, d, *J* = 8.5 Hz), 7.25 (1H, d, *J* = 8.8 Hz), 7.43 (1H, s), 8.06 (2H, s), 10.86 (1H, s); ^{13}C NMR (DMSO-*d*$_6$,, 126 MHz) δ 55.89, 56.13, 56.17, 105.40, 112.39, 112.46, 114.38, 129.78, 132.32, 133.12, 141.87, 145.86, 145.88, 149.02, 157.83, 164.20, 186.73.; MS(ES+) *m/z* 383.2 [M + H]$^+$; HRMS calcd for $C_{19}H_{18}N_4O_5$ [M + H]$^+$ 383.1350, found 383.1363.

(*Z*)-*N*-Methylpropan-1-imine oxide (23')

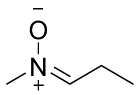

In a RB flask, N-hydroxylamine (835 mg, 10.0 mmol) and NaOMe (540 mg, 10.0 mmol) were dissolved in 15 mL of anhydrous EtOH. The mixture was stirred at rt under inert atmosphere for 10 min before propanal (725 μL, 10.0 mmol) was added dropwise. Stirr for 1h at rt under inert atmosphere. The solvent was removed under vacuum. The crude was dissolved in DCM and filtered. The solvent of the filtrate was removed under vacuum. This was purified by normal flash chromatography (0 to 5% MeOH in DCM) and yielded **23'** as a transparent oil (195 mg, 2.24 mmol, 22%).

R_f = 0.12 (5% MeOH in CH_2Cl_2); UV λ_{max} (EtOH/nm) 233.8; FTIR (cm^{-1}) ν 2967 (m, C-H alkane), 2877 (m, C-H alkane), 1659 (s, C=N imine), 1602 (s, N-O), 1458 (s, C-H CH_2), 1398 (s, C-H methyl), 1187 (s, C-N); ^{1}H NMR (Chloroform-*d*, 500 MHz) δ 1.03 (3H, t, *J* = 7.7 Hz), 2.42 (2H, p, *J* = 7.1 Hz), 3.61 (3H, s), 6.61 (1H, t, *J* = 5.7 Hz); ^{13}C NMR (Chloroform-*d*, 126 MHz) δ 9.82, 20.28, 52.25, 141.89.

(3*S*,4*R*,5*R*)/(3*R*,4*S*,5*S*)-5-Benzoyl-3-ethyl-2-methyl-*N*-phenylisoxazolidine-4-carboxamide (23a-A)

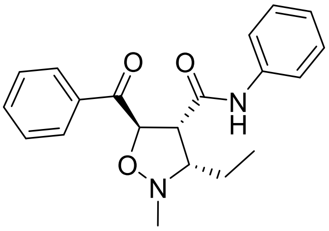

Compound **23a** was obtained following General procedure 21. Normal phase flash chromatography (0 to 10% EtOAc in PE) yielded compounds **23a-A**, **23a-C** and **23a-D** as a mixture. Semi-preparative HPLC yielded **23a-A** as transparent film (16 mg, 0.05 mmol, 16%).

R_f = 0.15 (10% EtOAc in PE); ^{1}H NMR (Chloroform-*d*, 500 MHz) δ 1.02 (3H, t, *J* = 7.5 Hz), 1.64 (2H, dq, *J* = 14.8, 7.4 Hz), 2.86 (4H, s), 3.77 (1H, dd, $^3J_{cis}$ = 5.9, $^3J_{trans}$ = 2.5 Hz), 5.57 (1H, d, $^3J_{trans}$ = 2.6 Hz), 7.13 (1H, t, *J* = 7.4 Hz), 7.35 (2H, t, *J* =

7.7 Hz), 7.50 (2H, t, *J* = 7.7 Hz), 7.60 (3H, m, H_2), 8.03 (2H, d, *J* = 7.7 Hz), 9.11 (1H, s); ^{13}C NMR (Chloroform-*d*, 126 MHz) δ 11.55, 21.05, 44.05, 56.98, 70.98, 80.68, 120.14 , 124.53, 129.01 , 129.18 , 129.28 , 134.16, 134.22, 137.91, 168.75, 195.22 ; MS(ES+) *m*/*z* 339.3 [M + H]$^+$.

(3*R*,4*R*,5*R*)/(3*S*,4*S*,5*S*)-4-Benzoyl-3-ethyl-2-methyl-*N*-phenylisoxazolidine-5-carboxamide (23a-B)

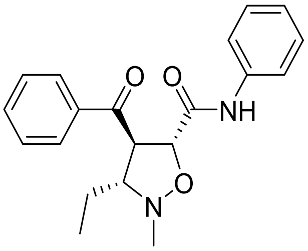

Compound **23a-B** was obtained following the above procedure for **23a-A**. Normal phase flash chromatography (0 to 10% EtOAc in PE) yielded compound **23a-B** as a white solid (21 mg, 0.06 mmol, 16%).

R_f = 0.20 (10% EtOAc in PE); ^{1}H NMR (Chloroform-*d*, 500 MHz) δ 0.84 (3H, t, *J* = 7.5 Hz), 1.51 (1H, dq, *J* = 14.6, 7.5 Hz), 1.61 (1H, dtd, *J* = 14.6, 7.5, 4.6 Hz), 2.88 (3H, s), 3.28 (1H, q, $^3J_{trans}$ = 7.7, 4.6 Hz), 4.53 (1H, dd, $^3J_{trans}$ = 6.7, $^3J_{trans}$ = 3.3 Hz), 4.61 (1H, d, $^3J_{trans}$ = 3.2 Hz), 7.14 (1H, t, *J* = 7.4 Hz), 7.36 (2H, t, *J* = 7.9 Hz), 7.52 (2H, t, *J* = 7.6 Hz), 7.56 – 7.64 (3H, m, H_2), 8.26 (2H, d, *J* = 7.1 Hz), 8.72 (1H, s); ^{13}C NMR (Chloroform-*d*, 126 MHz) δ 10.48, 24.82, 44.17, 59.07, 72.48, 79.54, 120.01, 124.71, 128.98, 129.19, 129.37, 133.84, 136.10, 137.33, 170.26, 198.01; MS(ES+) *m*/*z* 339.3 [M + H]$^+$.

(3*R*,4*R*,5*R*)/(3*S*,4*S*,5*S*)-5-Benzoyl-3-ethyl-2-methyl-*N*-phenylisoxazolidine-4-carboxamide (23a-C)

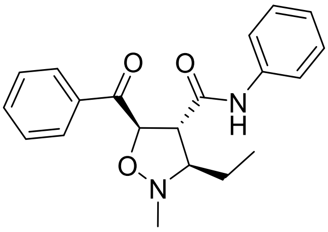

Compound **23a-C** was obtained following the above procedure for **23a-A**. Normal phase flash chromatography (0 to 10% EtOAc in PE) yielded compounds **23a-A**, **23a-C** and **23a-D** as a mixture. Semi-preparative HPLC yielded **23a-C** as transparent film (9 mg, 0.03 mmol, 15%).

R_f = 0.15 (10% EtOAc in PE); ^{1}H NMR (500 MHz, Chloroform-*d*) δ 0.85 (3H, t, *J* = 7.3 Hz), 1.10 (1H, s), 1.47 (1H, ddq, *J* = 14.5, 9.7, 7.3 Hz), 2.89 (3H, s), 3.37 (1H, d, *J* = 15.2 Hz), 4.99 (1H, s), 5.36 (1H, d, $^3J_{trans}$ = 7.0 Hz), 7.13 (1H, t, *J* = 7.4 Hz), 7.34 (2H, t, *J* = 7.8 Hz), 7.51 (2H, t, *J* = 7.6 Hz), 7.55 (2H, d, *J* = 8.0 Hz), 7.62 (1H, t, *J* = 7.4 Hz), 8.05 (2H, d, *J* = 7.6 Hz), 8.27 (1H, s); ^{13}C NMR (126 MHz, Chloroform-*d*) δ 11.48, 22.90, 46.42, 55.72, 72.73, 79.95, 119.70, 124.85, 128.52, 129.09, 129.26, 133.90, 136.92, 137.19, 170.14, 196.22; MS(ES+) *m*/*z* 339.3 [M + H]$^+$.

(3*S*,4*R*,5*R*)/(3*R*,4*S*,5*S*)-4-benzoyl-3-ethyl-2-methyl-*N*-phenylisoxazolidine-5-carboxamide (23a-D)

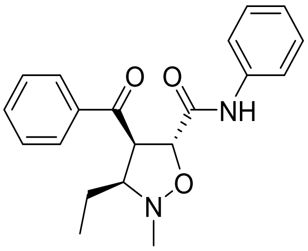

Compound **23a-D** was obtained following the above procedure for **23a-A**. Normal phase flash chromatography (0 to 10% EtOAc in PE) yielded compounds **23a-A**, **23a-C** and **23a-D** as a mixture. Semi-preparative HPLC yielded **23a-D** as transparent film (22 mg, 0.07 mmol, 33%).

R_f = 0.15 (10% EtOAc in PE); mp = 96-98 °C; UV λ_{max} (EtOH/nm) 245.5, 202.0; FTIR (cm^{-1}) ν 3056 (w, b, N-H amide), 1661 (s, C=O amide), 1592 (s, C=O ketone), 1523 (s, C=C aromatic); ^{1}H NMR (Chloroform-*d*, 500 MHz) δ 1.00 (3H, t, *J* = 7.5 Hz), 1.56 (1H, dt, *J* = 14.7, 7.4 Hz), 1.69 (1H, dt, *J* = 14.4, 7.3 Hz), 2.83 (3H, s), 3.29 (1H, q, $^3J_{cis}$ = 7.1 Hz), 3.70 (1H, t,

$^3J_{cis}$ = 7.0 Hz), 5.28 (1H, d, $^3J_{trans}$ = 6.5 Hz), 7.11 (1H, t, J = 7.4 Hz), 7.32 (2H, t, J = 7.8 Hz), 7.47 (2H, t, J = 7.7 Hz), 7.55 (2H, d, J = 8.0 Hz), 7.60 (1H, t, J = 7.4 Hz), 8.11 (2H, d, J = 8.2 Hz); ^{13}C NMR (Chloroform-*d*, 126 MHz) δ 10.65, 25.68, 44.25, 57.71, 72.27, 82.53, 119.85 (C_{15} and C_{19}), 124.64, 128.65, 129.17, 130.12, 134.19, 134.79, 137.83, 169.12, 199.49; MS(ES+) *m/z* 339.3 [M + H]$^+$; HRMS calcd for $C_{20}H_{22}N_2O_6$ [M + H]$^+$ 339.1703, found 339.1692.

(3S,4*R*,5*R*)-*N*-(3,4-dimethoxyphenyl)-3-ethyl-5-(4-methoxybenzoyl)-2-methylisoxazolidine-4-carboxamide (23b-A)

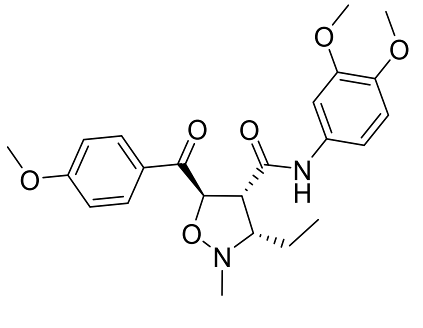

Compound **23b** was obtained following General procedure 21. Normal phase flash chromatography (0 to 10% EtOAc in PE) yielded compound **23b-A** as a white solid (41.9 mg, 97.9 μmol, 13%).

R_f = 0.42 (50% EtOAc in PE); mp = 141-142 °C; UV λ_{max} (EtOH/nm) 272.8; FTIR (cm^{-1}) ν 3330 (m, N-H), 2932 (m, C-H), 2836 (m, C-H), 1667 (s, C=O), 1597 (s, C=O), 1510 (s, C=C); ^{1}H NMR (Chloroform-*d*, 500 MHz) δ 0.78 (3H, t, J = 7.4 Hz), 1.40 – 1.50 (1H, m), 1.51 – 1.61 (1H, m), 2.82 (3H, s), 3.22 (1H, s), 3.80 (3H, s), 3.80 (3H, s), 3.84 (3H, s), 4.40 (1H, dd, $J_{trans,cis}$ = 6.7, 3.5 Hz), 4.52 (1H, d, J_{cis} = 3.3 Hz), 6.75 (1H, d, J = 8.6 Hz), 6.91 (2H, d, J = 8.9 Hz), 7.30 (1H, d, J = 2.4 Hz), 8.18 (2H, d, J = 8.9 Hz), 8.58 (1H, s); ^{13}C NMR (Chloroform-*d*, 126 MHz) δ 10.44, 24.73, 44.10, 55.54, 56.01, 56.15, 58.57, 72.43, 104.69, 111.33, 111.91, 114.05, 128.86, 130.86, 131.68, 146.03, 149.12, 164.06, 169.79, 196.08; MS(ES+) *m/z* 429.3 [M + H]$^+$; HRMS calcd for $C_{23}H_{28}N_2O_6$ [M + H]$^+$ 429.2020, found 429.22041.

(3*R*,4*R*,5*R*)-*N*-(3,4-dimethoxyphenyl)-3-ethyl-4-(4-methoxybenzoyl)-2-methylisoxazolidine-5-carboxamide (23b-B)

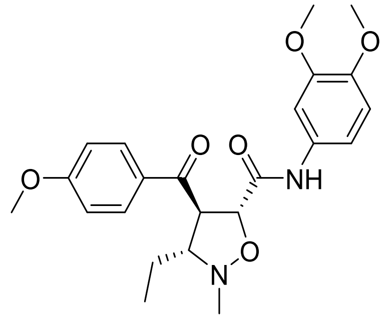

Compound **23b-B** was obtained following the above procedure for **23b-A**. Normal phase flash chromatography (0 to 10% EtOAc in PE) yielded compounds **23b-B**, **23b-C** and **23b-D** as a mixture. Semi-preparative HPLC yielded **23b-B** as transparent film (35.5 mg, 82.5 μmol, 11%).

R_f = 0.23 (50% EtOAc in PE); mp = 119-120 °C; UV λ_{max} (EtOH/nm) 282.2, 271.2, 211.4; FTIR (cm^{-1}) ν 3296 (m, N-H), 2933 (w, C-H), 2836 (w, C-H), 1654 (s, C=O), 1598 (s, C=O), 1511 (s, C=C); ^{1}H NMR (Chloroform-*d*, 500 MHz) δ 0.94 (3H, t, J = 7.4 Hz), 1.47 – 1.59 (1H, m), 1.64 (1H, q, J = 7.0 Hz), 2.79 (3H, s), 3.27 (1H, d, J_{trans} = 7.4 Hz), 3.67 (1H, t, J_{trans} = 7.1 Hz), 3.78 (3H, s), 3.80 (3H, s), 3.81 (3H, s), 5.20 (1H, d, J_{trans} = 6.8 Hz), 6.72 (1H, d, J = 8.6 Hz), 6.83 – 6.92 (3H, m), 7.31 (1H, d, J = 2.4 Hz), 8.04 (2H, d, J = 8.9 Hz), 8.17 (1H, s); ^{13}C NMR (Chloroform-*d*, 126 MHz) δ 10.60, 14.21, 25.49, 44.12, 55.55, 55.92, 56.12, 57.32, 72.17, 82.56, 104.46, 111.24, 111.54, 113.81, 127.62, 131.48, 132.53, 145.86, 149.00, 164.35, 168.57, 196.92; MS(ES+) *m/z* 429.3 [M + H]$^+$; HRMS calcd for $C_{23}H_{28}N_2O_6$ [M + H]$^+$ 429.2020, found 429.22041.

(3*R*,4*R*,5*R*)-*N*-(3,4-dimethoxyphenyl)-3-ethyl-5-(4-methoxybenzoyl)-2-methylisoxazolidine-4-carboxamide (23b-C)

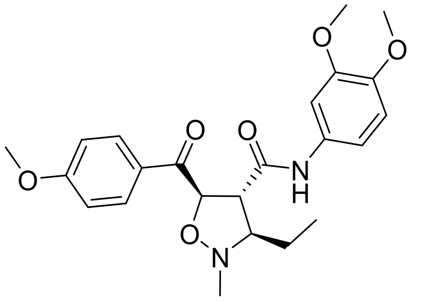

Compound **23b-C** was obtained following the above procedure for **23b-A**. Normal phase flash chromatography (0 to 10% EtOAc in PE) yielded compounds **23b-B**, **23b-C** and **23b-D** as a mixture. Semi-preparative HPLC yielded **23b-C** as transparent film (26.9 mg, 62.9 µmol, 9%).

R_f = 0.23 (50% EtOAc in PE); UV λ_{max} (EtOH/nm) 273.4, 211.2, 200.6; FTIR (cm^{-1}) ν 3326 (m, N-H), 2933 (m, C-H), 2836 (w, C-H), 1665 (s, C=O), 1597 (s, C=O), 1510 (s, C=C); ^{1}H NMR (Chloroform-*d*, 500 MHz) δ 0.80 (3H, t, *J* = 5.3 Hz), 0.98 – 1.10 (1H, m), 1.39 (1H, s), 2.85 (3H, s), 3.28 (1H, s), 3.79 (3H, s), 3.80 (3H, s), 3.82 (3H, s), 4.86 (1H, t, J_{trans} = 6.9 Hz), 5.26 (1H, br, s), 6.74 (1H, d, *J* = 8.5 Hz), 6.78 – 6.86 (1H, m), 6.91 (3H, d, *J* = 8.5 Hz), 7.41 (1H, s), 7.91 – 8.02 (2H, m), 8.18 (1H, d, *J*=15.0 Hz); ^{13}C NMR (Chloroform-*d*, 126 MHz) δ 11.39, 22.62, 46.36, 55.16, 55.58, 55.95, 56.13, 58.46, 72.65, 79.88, 104.39, 111.30, 111.37, 114.14, 129.61, 130.83, 131.34, 146.06, 149.12, 164.11, 169.82, 193.35; MS(ES+) *m/z* 429.4 [M + H]$^+$; HRMS calcd for $C_{23}H_{28}N_2O_6$ [M + H]$^+$ 429.2020, found 429.1998.

(3*S*,4*R*,5*R*)-*N*-(3,4-dimethoxyphenyl)-3-ethyl-4-(4-methoxybenzoyl)-2-methylisoxazolidine-5-carboxamide (23b-D)

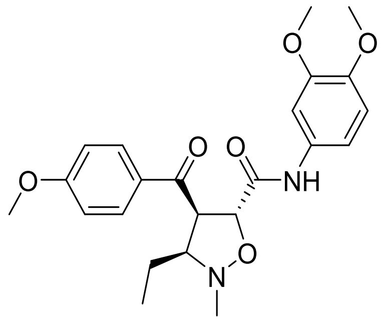

Compound **23b-D** was obtained following the above procedure for **23b-A**. Normal phase flash chromatography (0 to 10% EtOAc in PE) yielded compounds **23b-B**, **23b-C** and **23b-D** as a mixture. Semi-preparative HPLC yielded **23b-D** as transparent film (22.2 mg, 51.9 µmol, 7%).

R_f = 0.23 (50% EtOAc in PE); UV λ_{max} (EtOH/nm) 282.2; FTIR (cm^{-1}) ν 3295 (br, N-H), 2934 (w, C-H), 1655 (s, C=O), 1597 (s, C=O), 1510 (S, C=C); ^{1}H NMR (Chloroform-*d*, 500 MHz) δ 0.97 (3H, t, *J* = 7.4 Hz), 1.60 (2H, d, *J* = 9.0 Hz), 2.80 (3H, s), 2.88 (1H, s), 3.79 (7H, d, *J* = 3.0 Hz), 3.82 (3H, s), 5.51 (1H, s), 6.74 (1H, d, *J* = 8.6 Hz), 6.85 – 6.91 (2H, m), 6.91 – 6.96 (1H, m), 7.31 (1H, d, *J* = 2.2 Hz), 7.95 (2H, d, *J* = 8.3 Hz), 9.01 (1H, s); ^{13}C NMR (Chloroform-*d*, 126 MHz) δ 11.46, 21.07, 44.13, 55.57, 55.90, 55.99, 56.16, 56.45, 71.19, 80.79, 104.84, 111.33, 111.91, 114.10, 127.13, 131.37, 131.51, 131.62, 145.86, 149.05, 164.25, 168.24, 193.4; MS(ES+) *m/z* 429.3 [M + H]$^+$; HRMS calcd for $C_{23}H_{28}N_2O_6$ [M + H]$^+$ 429.2020, found 429.22041.

2.4.5. Transformations: Reductions

4-Oxo-*N*,4-diphenylbutanamide (24a)

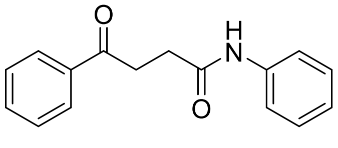

Compound **24a**was obtained following General procedure 22. Flash chromatography (0 to 20% EtOAc in PE) yielded compound **24a** as a beige solid (88 mg, 0.35 mmol, 87%).

R_f = 0.34 (20% EtOAc in PE); UV λ_{max} (EtOH/nm) 243.2, 204.3; FTIR (cm^{-1}) ν 3309 (m, N-H amide), 1699 (s, C=O amide), 1657 (s, C=O ketone), 1595 (s, C=C aromatic), 1531 (s, C=C aromatic), 1442 (s, C-H CH_2); ^{1}H NMR (Chloroform-*d*, 500 MHz) δ 2.82 (2H, t, *J* = 6.4 Hz), 3.46 (2H, t, *J* = 6.4 Hz), 7.08 (1H, t, *J* = 7.7 Hz), 7.30 (2H, t, *J* = 8.2, 7.7 Hz), 7.47 (2H, t, *J* = 7.8 Hz), 7.52 (2H, d, *J* = 7.7 Hz), 7.58 (1H, t, *J* = 7.7 Hz), 7.87 (1H, s), 8.00 (2H, d, *J* = 7.7 Hz); ^{13}C NMR (Chloroform-*d*, 126 MHz) δ 31.57, 34.26, 119.91, 124.29, 128.27, 128.81, 129.07, 133.60, 136.52, 138.10, 170.59, 199.41; MS(ES-) *m/z* 252.1 [M - H]$^-$; HRMS calcd for $C_{16}H_{15}NO_2$ [M + Na]$^+$ 276.0995, found 276.0919.

***N*-(3,4-dimethoxyphenyl)-4-(4-methoxyphenyl)-4-oxobutanamide (24b)**

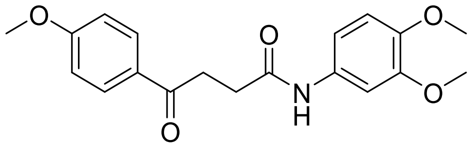

Compound **24b** was obtained following General procedure 22. Flash chromatography (20 to 100% EtOAc in PE) yielded compound **24b** as a white solid (10 mg, 0.03 mmol, 47%).

R_f = 0.24 (5% MeOH in CH_2Cl_2), mp = 148-150 °C; UV λ_{max} (EtOH/nm) 265.4, 211.4; FTIR (cm^{-1}) ν 3287 (m, N-H amide), 2908 (m, C-H alkane), 2836 (m, C-H alkane), 1670 (s, C=O amide), 1649 (s, C=O ketone) 1596 (s, C=C aromatic), 1507 (s, C=C aromatic); ^{1}H NMR (Chloroform-*d*, 500 MHz) δ 2.78 (2H, t, *J* = 6.4 Hz), 3.41 (2H, t, *J* = 6.4 Hz), 3.84 (3H, s), 3.86 (3H, s), 3.87 (3H, s), 6.77 (1H, d, *J* = 8.6 Hz), 6.88 (1H, dd, *J* = 8.6, 2.4 Hz), 6.94 (2H, dt, *J* = 5.0, 2.8Hz), 7.34 (1H, d, *J* = 2.4 Hz), 7.79 (1H, s), 7.98 (2H, dt, *J* = 4.9, 2.8 Hz); ^{13}C NMR (Chloroform-*d*, 126 MHz) δ 31.68, 33.97, 55.64, 56.04, 56.27, 104.97, 111.50, 111.79, 113.97 , 129.65, 130.57, 131.90, 145.85, 149.17, 163.95, 170.59, 197.88; MS(ES+) *m/z* 344.3 [M + H]$^+$; HRMS calcd for $C_{19}H_{21}NO_5$ [M + H]$^+$ 344.1496, found 344.1504.

(*E*)-4-hydroxy-*N*,4-diphenylbut-2-enamide (25a)

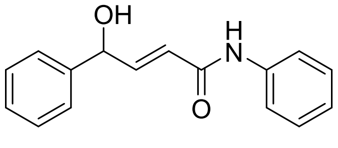

Compound **25a** was obtained following General procedure 23. Compound **25a** was yielded as a white solid (101 mg, 0.40 mmol, 100%).

R_f = 0.18 (20% EtOAc in PE); UV λ_{max} (EtOH/nm) ν 270.8, 201.9; FTIR (cm^{-1}) 3314 (m, N-H amide), 3255 (m, O-H alcohol) 2921 (m, C-H alkane), 1672 (s, C=O amide), 1598 (s, C=C aromatic); ^{1}H NMR (Chloroform-*d*, 500 MHz) δ 5.78 (1H, d, *J* = 4.9 Hz), 6.77 (1H, dd, *J* = 15.2, 1.8 Hz), 7.45 (1H, dd, *J* = 15.2, 4.9 Hz), 7.51 (1H, t, *J* = 7.5 Hz), 7.71 (3H, m), 7.75 – 7.83 (4H, m), 8.01 (2H, d, *J* = 8.1 Hz); ^{13}C NMR (Chloroform-*d*, 126 MHz) δ 72.89, 119.92 , 122.39, 124.00, 126.37, 127.61, 128.30, 128.51, 138.11, 141.34, 145.91, 164.80; MS(ES+) *m/z* 254.2 [M + H]$^+$; HRMS calcd for $C_{16}H_{15}NO_2$ [M + H]$^+$ 254.1179, found 254.1175.

***(E)-N*-(3,4-dimethoxyphenyl)-4-hydroxy-4-(4-methoxyphenyl)but-2-enamide (25b)**

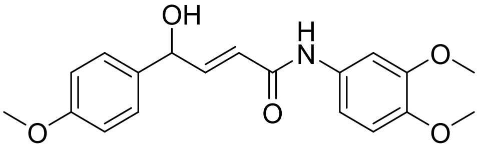

Compound **25b** was obtained following General procedure 23. Flash chromatography (50 to 70% EtOAc in PE) afforded **25b** as a white solid (14.6 mg, 0.04 mmol, 62%).

R_f = 0.18 (60 % EtOAc in PE); mp = 151 - 154 °C; UV λ_{max} (EtOH/nm) ν 300.4, 205.8; FTIR (cm^{-1}) ν 3254 (b, m, O-H alcohol), 2837 (m, C-H alkane), 1670 (s, C=O amide), 1605 (s, C=C aromatic), 1510 (s, C=C aromatic); ^{1}H-NMR (Methanol-d_4, 500 MHz) δ 3.79 (3H, s), 3.81 (3H, s), 3.82 (3H, s), 5.31 (1H, dd, *J* = 5.0, 1.7 Hz), 5.49 (1H, s), 6.34 (1H, dd, *J* = 15.2 Hz, 1.7 Hz), 6.87 - 6.94 (3H, m), 6.99 (1H, dd, *J* = 15.2, 5.0 Hz), 7.09 (1H, dd, *J* = 8.7 Hz, *J* = 2.4 Hz), 7.30 (2H, d, *J* = 8.7 Hz), 7.39 (1H, d, *J* = 2.4 Hz); ^{13}C NMR (Methanol-d_4, 126 MHz) δ 55.72, 56.40, 56.77, 73.90, 106.64, 113.31, 113.67, 114.98, 123.56, 129.09, 133.66, 135.40, 147.37, 147.72, 150.45, 160.90, 166.27 MS(ES+) *m/z* 344.2 [M + H]$^+$; HRMS calcd for $C_{19}H_{22}NO_5$ [M + H]$^+$ 344.1492, found 344.1495.

4-Hydroxy-*N*,4-diphenylbutanamide (26a)

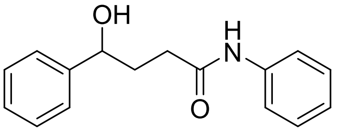

Compound **26a** was obtained following General procedure 24. Normal phase flash chromatography (10 to 40% EtOAc in PE) yielded **26a** as a yellow oil (96 mg, 0.38 mmol, 94%).

R_f = 0.12 (30% EtOAc in PE); mp = 145-147 °C; UV λ_{max} (EtOH/nm) 242.6, 202.7; FTIR (cm^{-1}) ν 3291 (b, m, O-H alcohol), 3075 (m, N-H amide), 1666 (s, C=O amide), 1596 (s, C=C aromatic); ^{1}H NMR (Chloroform-*d*, 500 MHz) δ 1.99 – 2.17 (2H, m), 2.40 – 2.53 (2H, m), 4.75 (1H, dd, *J* = 8.1, 4.3 Hz), 7.09 (1H, t, *J* = 7.4 Hz), 7.26 (3H, m), 7.32 (4H, d, *J* = 4.4 Hz), 7.49 (2H, d, *J* = 7.9 Hz), 8.24 (1H, s); ^{13}C NMR (Chloroform-*d*, 126 MHz) δ 33.94, 34.35, 73.59, 120.20, 124.40, 125.81, 127.56, 128.51 , 128.99, 137.96, 144.21, 172.31; MS(ES+) *m/z* 254.2 [M - H]$^+$; HRMS calcd for $C_{16}H_{17}NO_2$ [M + Na]$^+$ 278.1151, found 278.1149.

***N*-(3,4-dimethoxyphenyl)-4-hydroxy-4-(4-methoxyphenyl)butanamide (26b)**

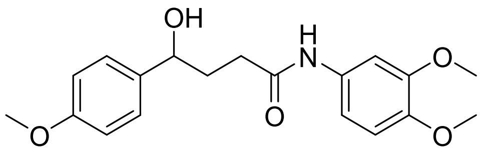

Compound **26b** was obtained following General procedure 24. Normal phase flash chromatography (35 to 55 % EtOAc in PE) yielded compound **26b** as a white solid (26 mg, 0.08 mmol, 85%).

R_f = 0.19 (50 % EtOAc in petrol ether); mp = 147 - 150 °C; UV λ_{max} (EtOH/nm) ν 254.0, 209.0; FTIR (cm^{-1}) ν 3395 (b, m, O-H alcohol), 3287 (m, N-H amide), 2929 (m, C-H alkane), 2834 (m, C-H alkane), 1658 (s, C=O amide), 1605 (s, C=C aromatic), 1510 (s, C=C aromatic); ^{1}H-NMR (Chloroform-*d*, 500 MHz) δ 2.09 - 2.18 (2H, m), 2.44 - 2.52 (2H, m), 3.80 (3H, s), 3.85 (3H, s), 3.87 (3H, s), 4.78 (1H, dd, *J* = 7.4, 5.2 Hz), 7.43 (1H, s), 6.76 - 6.85 (2H, m), 6.86 - 6.90 (2H, m), 7.27 - 7.31 (2H, m), 7.33 (1H, d, *J* = 2.4 Hz); ^{13}C-NMR (Chloroform-*d*, 126 MHz) δ 34.11, 34.32, 55.45, 56.08, 56.29, 73.42, 105.17, 111.50, 111.95, 114.05, 127.13, 131.61, 136.45, 146.06, 149.24, 159.25, 171.55; MS(ES-) *m/z* 344.1 [M - H]$^-$;

2.4.6. Transformations: 1,4-Additions

4-Oxo-*N*,4-diphenyl-2-(pyrrolidin-1-yl)butanamide (27a)

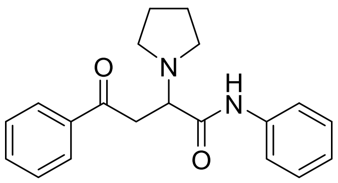

Compound **27a** was obtained following General procedure 25. Amine-functionalised silica flash chromatography (20 to 50% EtOAc in PE) yielded compound **27a** (40 mg, 0.12 mmol, 70%) as a yellow oil.

R_f = 0.28 (50% EtOAc in PE); UV λ_{max} (EtOH/nm) 240.8, 200.0; FTIR (cm^{-1}) ν 3312 (w, N-H amide), 2961 (w, C-H alkane), 1674 (s, C=O amide), 1596 (s, C=C ketone), 1515 (s, C=C aromatic); ^1H NMR (Chloroform-*d*, 500 MHz) δ 1.83 (4H, h, *J* = 6.3 Hz), 2.71 (4H, dtd, *J* = 14.1, 8.6, 4.1 Hz), 3.06 (1H, dd, *J* = 16.8, 5.3 Hz), 3.68 – 3.77 (1H, m), 4.35 (1H, dd, *J* = 6.7, 5.2 Hz), 7.08 (1H, t, *J* = 7.4 Hz), 7.30 (2H, t, *J* = 7.9 Hz), 7.47 (2H, t, *J* = 7.7 Hz), 7.52 – 7.59 (3H, m), 7.99 – 8.05 (2H, m), 9.22 (1H, s); ^{13}C NMR (Chloroform-*d*, 126 MHz) δ 23.88, 34.63, 49.91, 61.50, 119.57, 124.14, 128.37, 128.72, 129.05, 133.22, 137.08, 137.91, 170.98, 198.45; MS(ES+) *m/z* 323.3 $[M - H]^+$; HRMS calcd for $C_{20}H_{22}N_2O_2$ $[M + H]^+$ 323.1757, found 323.1677.

***N*-(3,4-dimethoxyphenyl)-4-(4-methoxyphenyl)-4-oxo-2-(pyrrolidin-1-yl)butanamide (27b)**

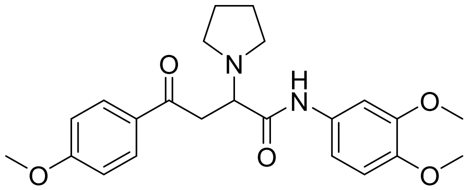

Compound **27b** was obtained following General procedure 25. Amine-functionalised silica flash chromatography (30% CH_2Cl_2 in PE) yielded compound **27b** as a yellow solid (117 mg, 0.283 mmol, 97%).

R_f = 0.32 (60% CH_2Cl_2 in PE, amine-functinalised silica); UV λ_{max} (EtOH/nm) 267.6; FTIR (cm^{-1}) ν 3316 (br, N-H), 2933 (w, C-H), 2833 (w, C-H), 1668 (s, C=O), 1597 (s, C=O), 1509 (s, C=C); ^1H NMR (Chloroform-*d*, 500 MHz) δ 1.68 – 1.80 (5H, m), 2.57 – 2.71 (4H, m), 3.00 (1H, dd, *J*=16.8, 5.4 Hz), 3.59 (1H, dd, *J* = 16.8, 6.5 Hz), 3.77 (3H, s), 3.78 (3H, s), 3.79 (3H, s), 4.21 (1H, dd, *J* = 6.4, 5.3 Hz), 6.71 (1H, d, *J* = 8.6 Hz), 6.81 (1H, dd, *J* = 8.6, 2.5 Hz), 6.86 (2H, d, *J* = 8.9 Hz), 7.33 (1H, d, *J* = 2.4 Hz), 7.93 (2H, d, *J* = 8.9 Hz), 9.07 (1H, s); ^{13}C NMR (Chloroform-*d*, 126 MHz) δ 23.76, 34.62, 49.90, 55.49, 55.93, 56.12, 61.40, 104.28, 111.21, 111.26, 113.76, 129.88, 130.56, 131.67, 145.55, 149.02, 163.56, 170.78, 196.75; MS(ES+) *m/z* 413.3 $[M + H]^+$; HRMS calcd for $C_{28}H_{28}N_2O_5$ $[M + H]^+$ 413.2071, found 413.2086.

2.5. NMR spectra

2.5.1. Build

^1H and ^{13}C NMR spectra of (*E*)-4-(4-methoxyphenyl)-4-oxobut-2-enoic acid (2b)

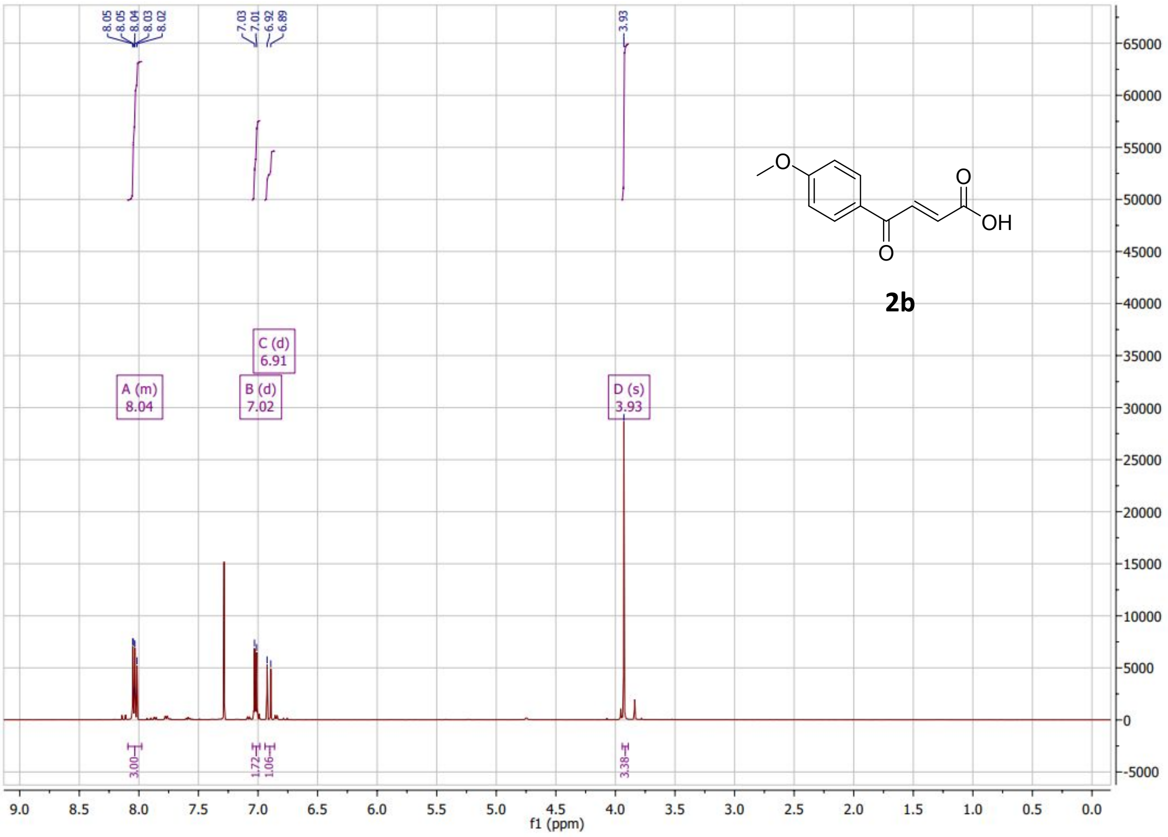

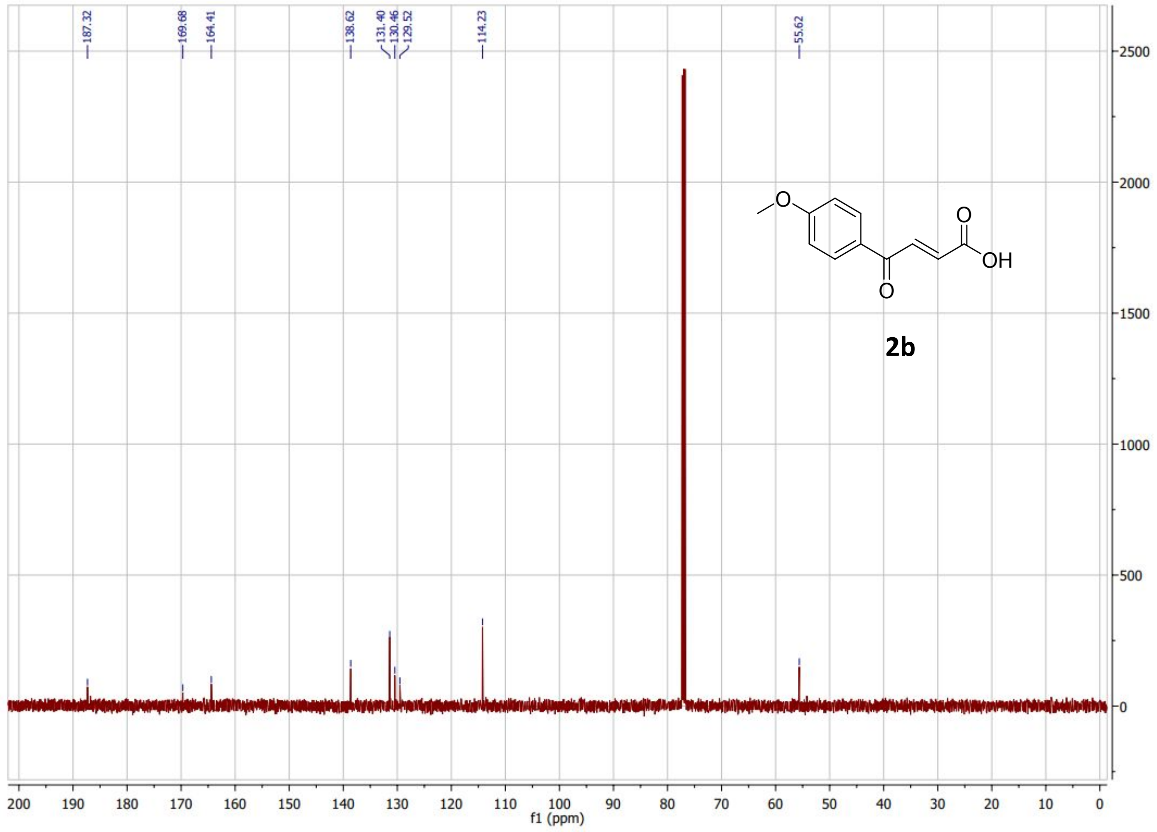

¹H and ¹³C NMR spectra of (*E*)-4-(4-cyanophenyl)-4-oxobut-2-enoic acid (2c)

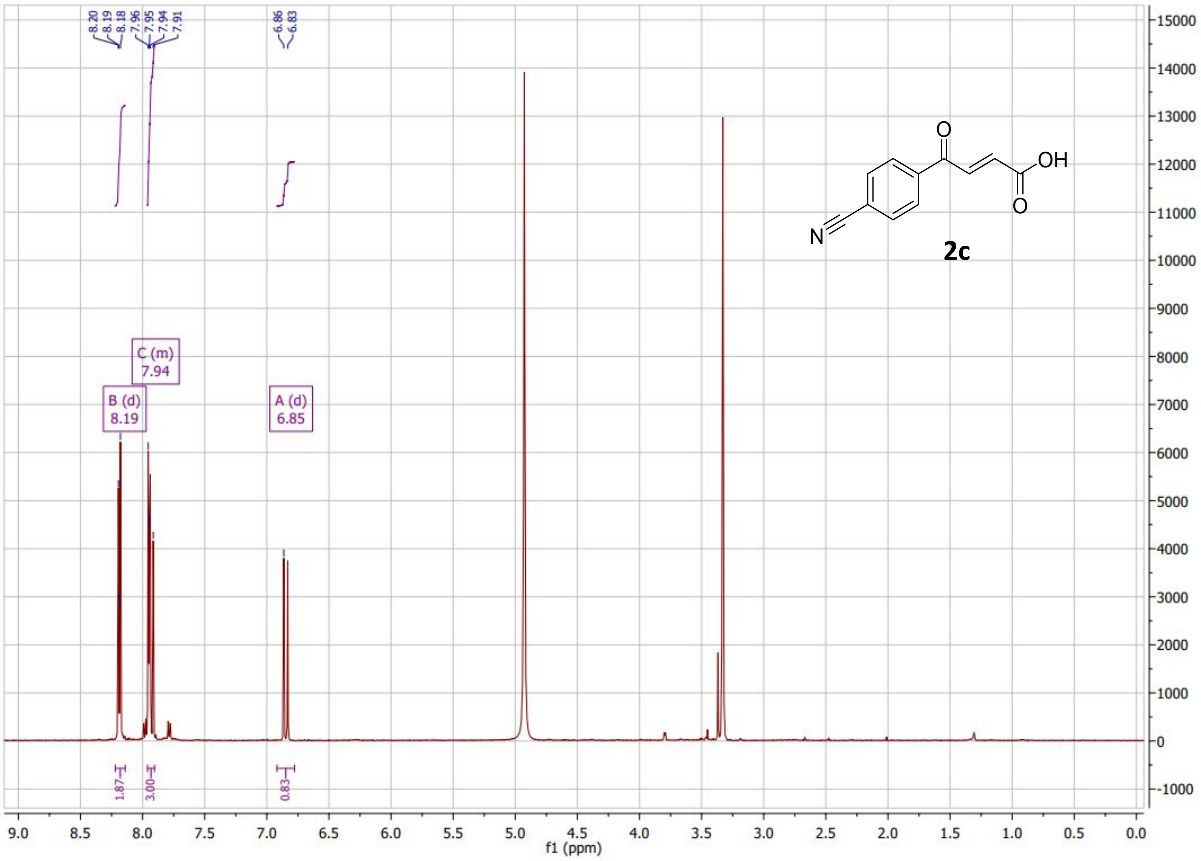

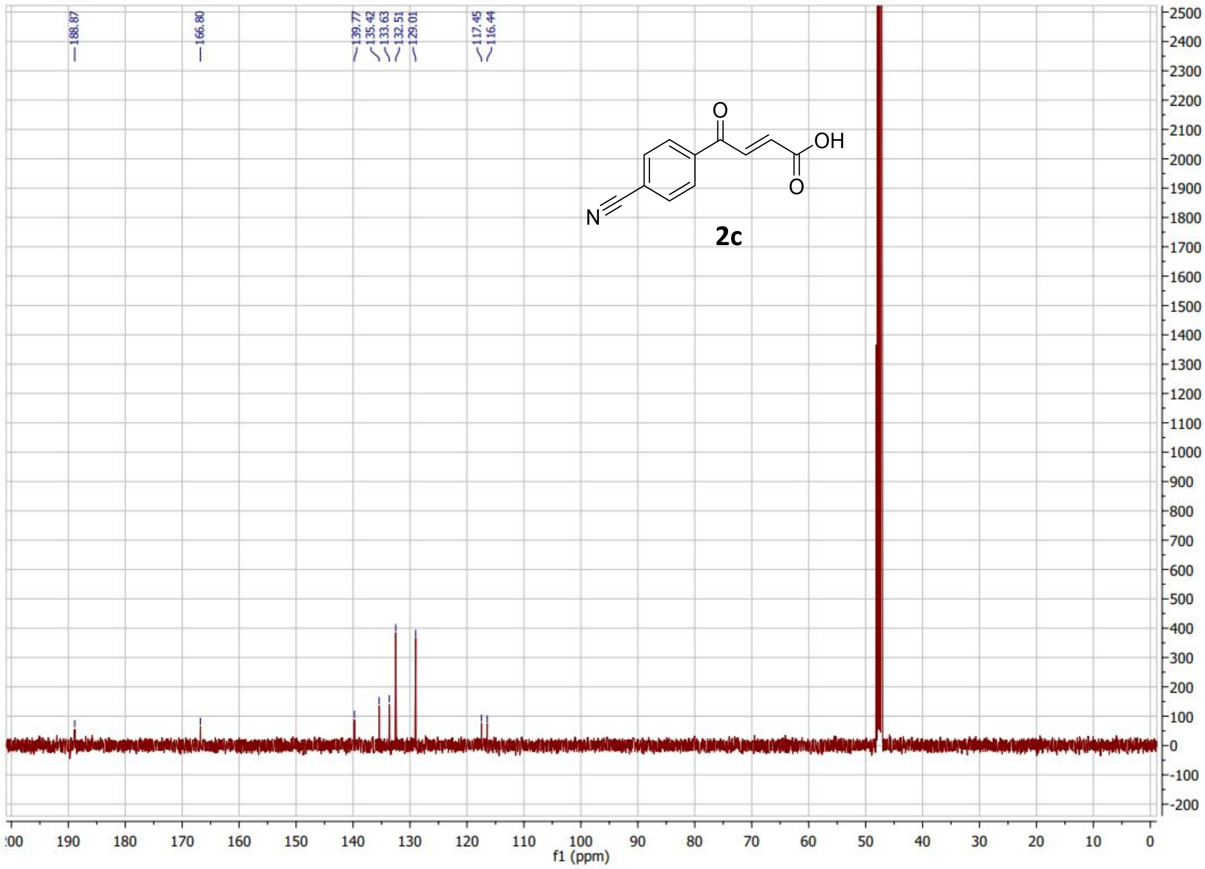

^{1}H and ^{13}C NMR spectra of (*E*)-4-Cyclohexyl-4-oxobut-2-enoic acid (2d)

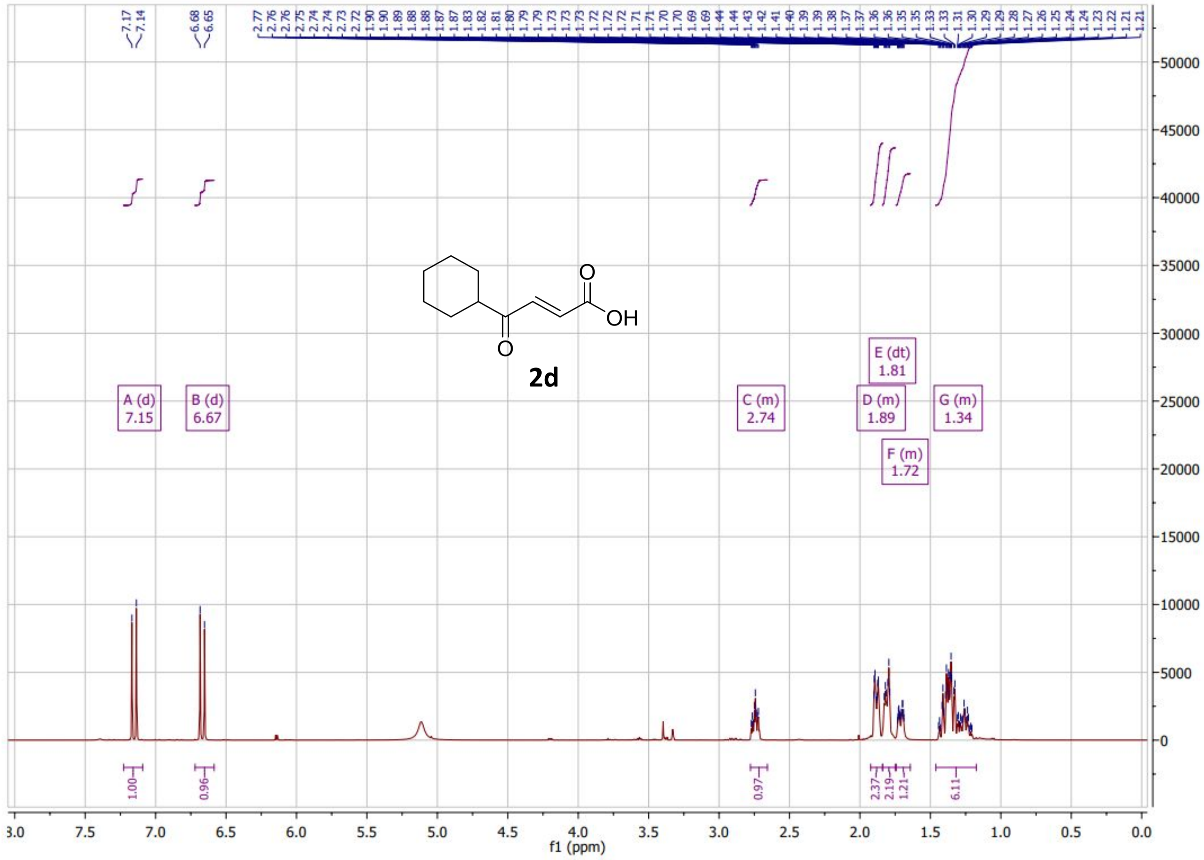

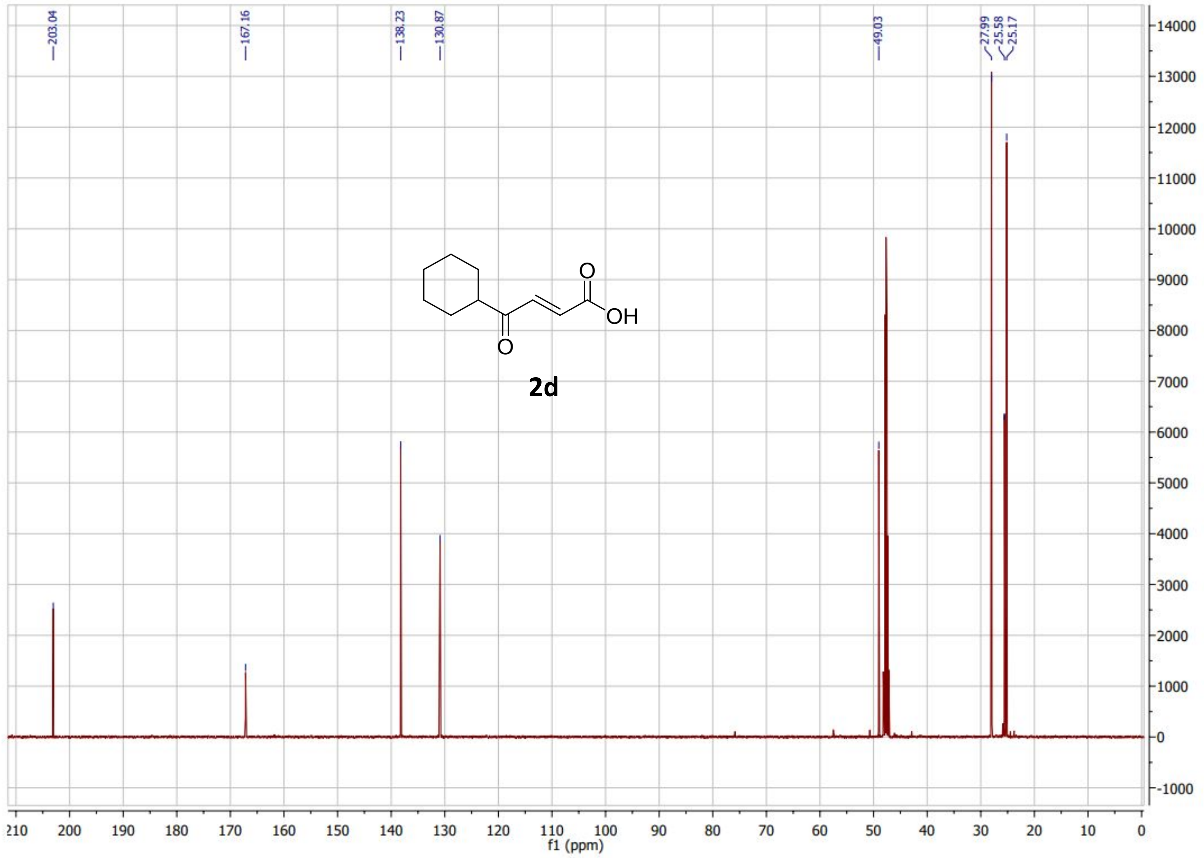

^{1}H and ^{13}C NMR spectra of (*E*)-4-Oxo-4-(tetrahydro-2H-pyran-4-yl)but-2-enoic acid (2e)

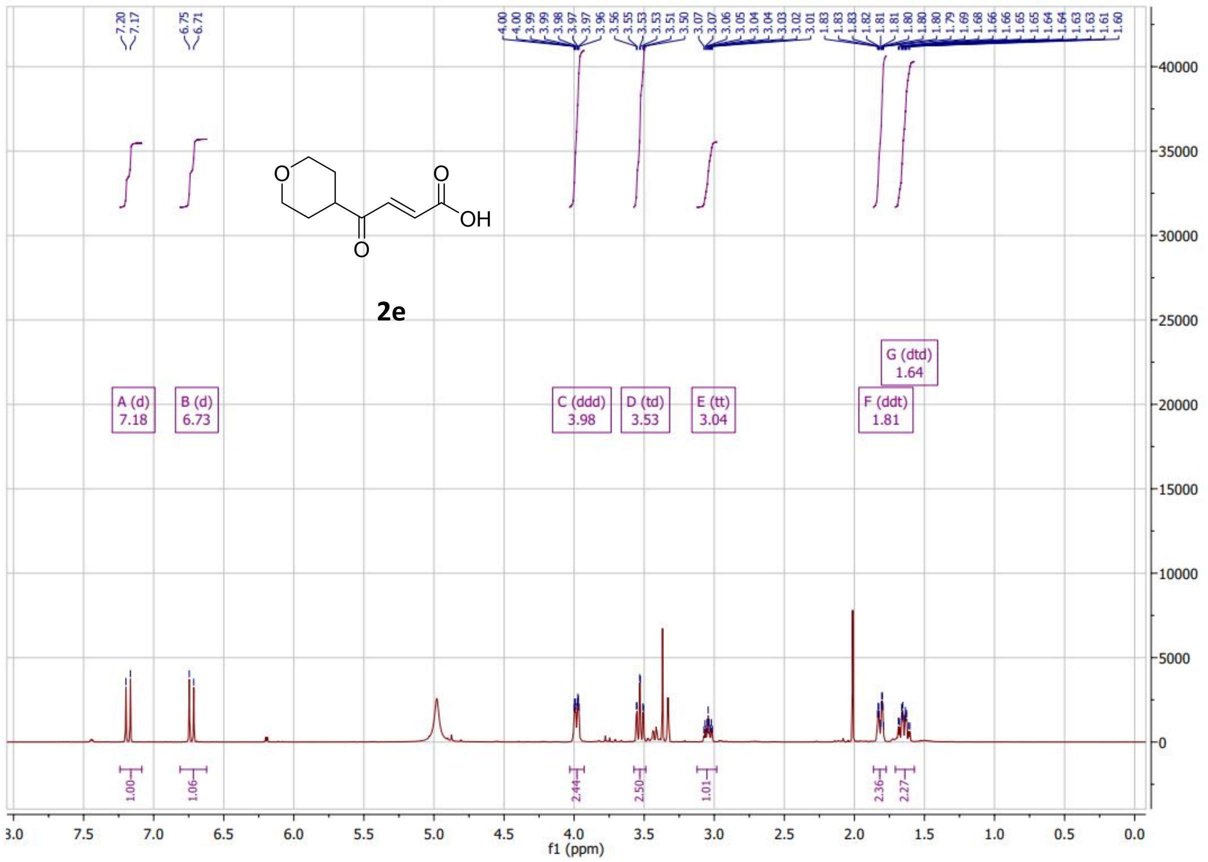

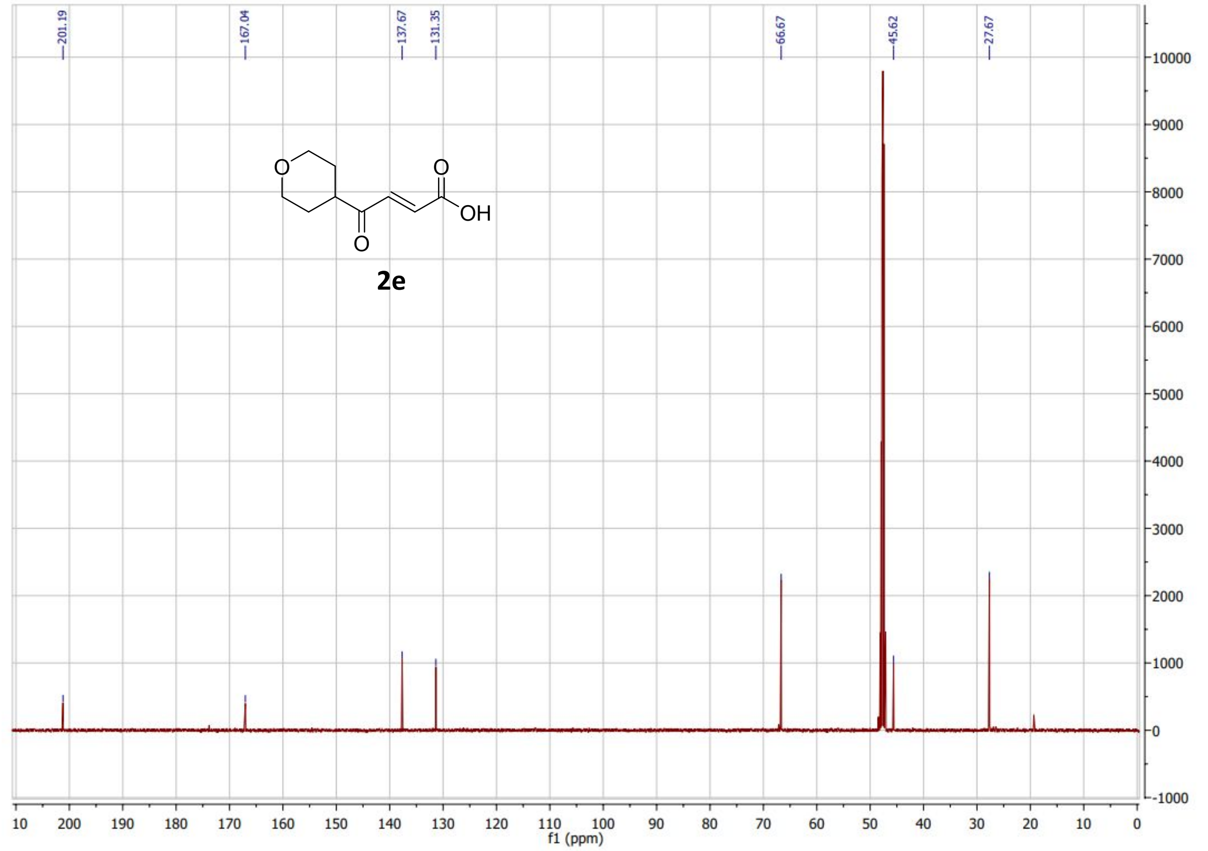

2.5.2. Couple

¹H and ¹³C NMR spectra of (*E*)-4-Oxo-*N*,4-diphenylbut-2-enamide (1a)

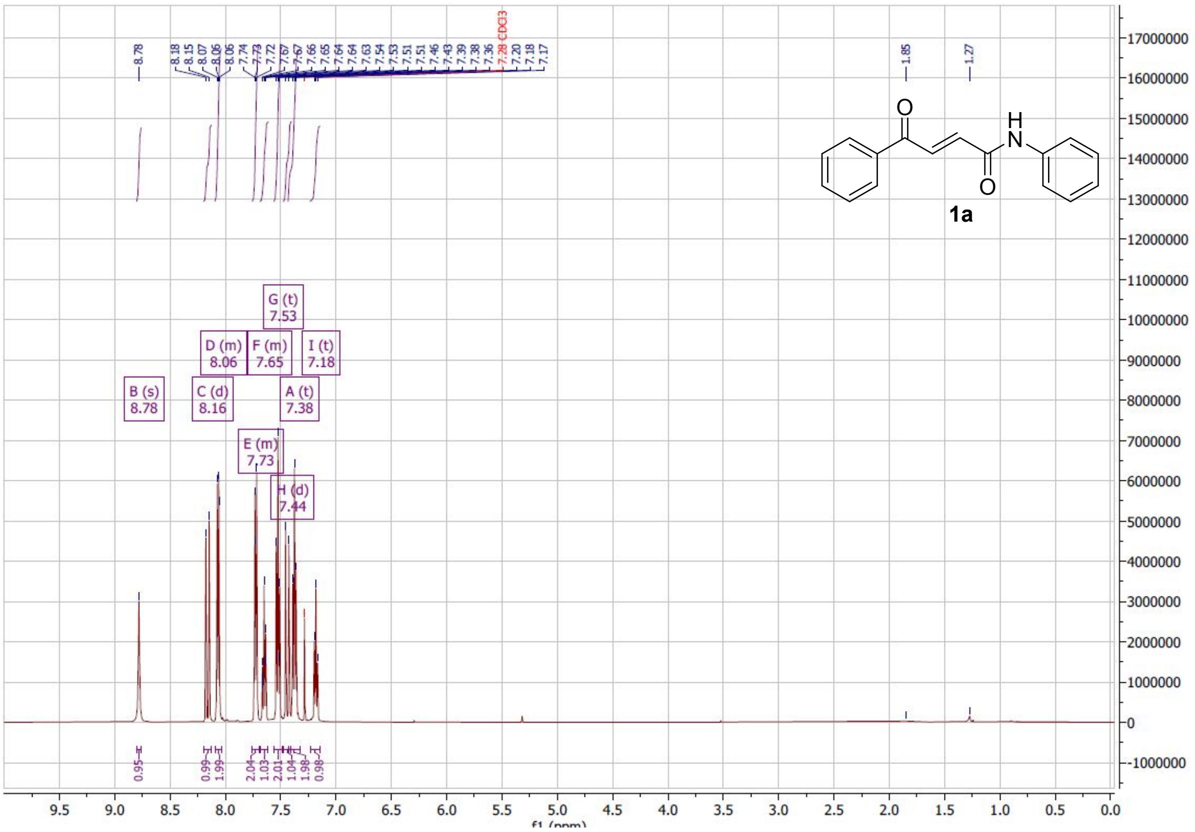

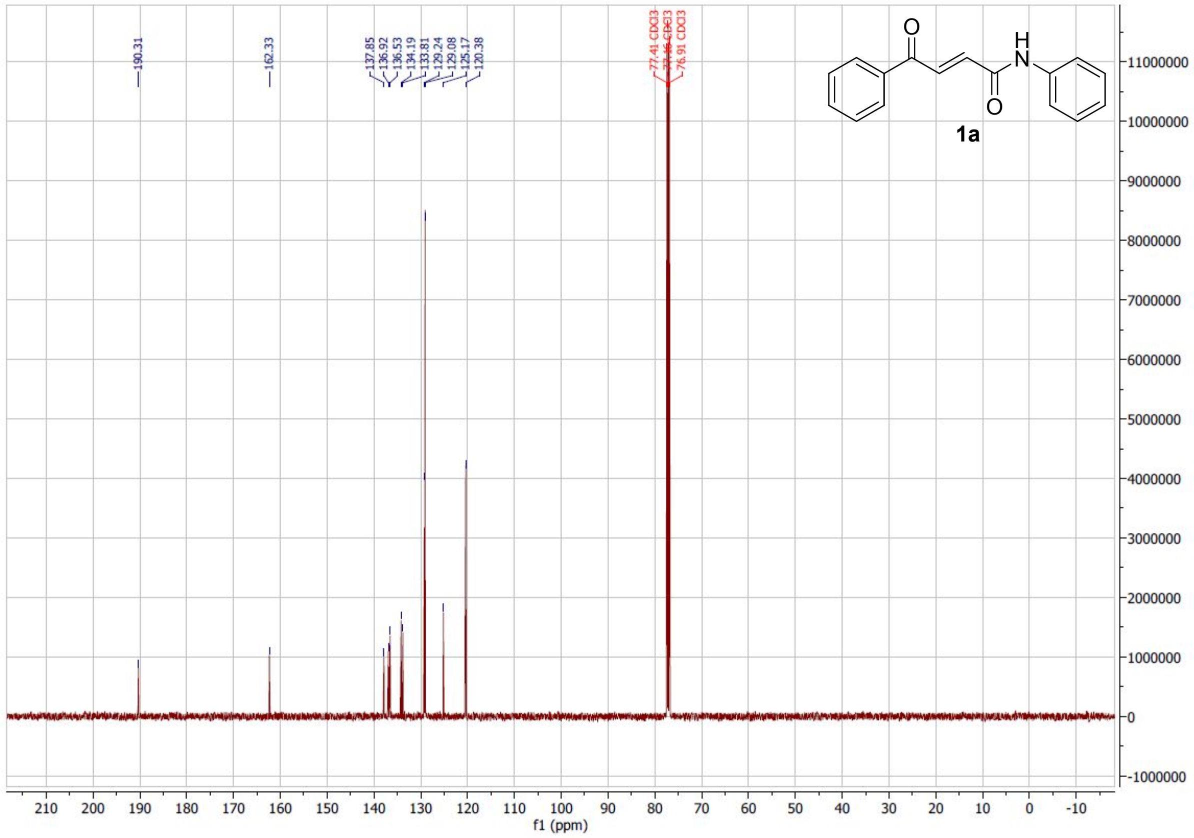

^{1}H and ^{13}C NMR spectra of (*E*)-*N*-(3,4-dimethoxyphenyl)-4-(4-methoxyphenyl)-4-oxobut-2-enamide (1b)

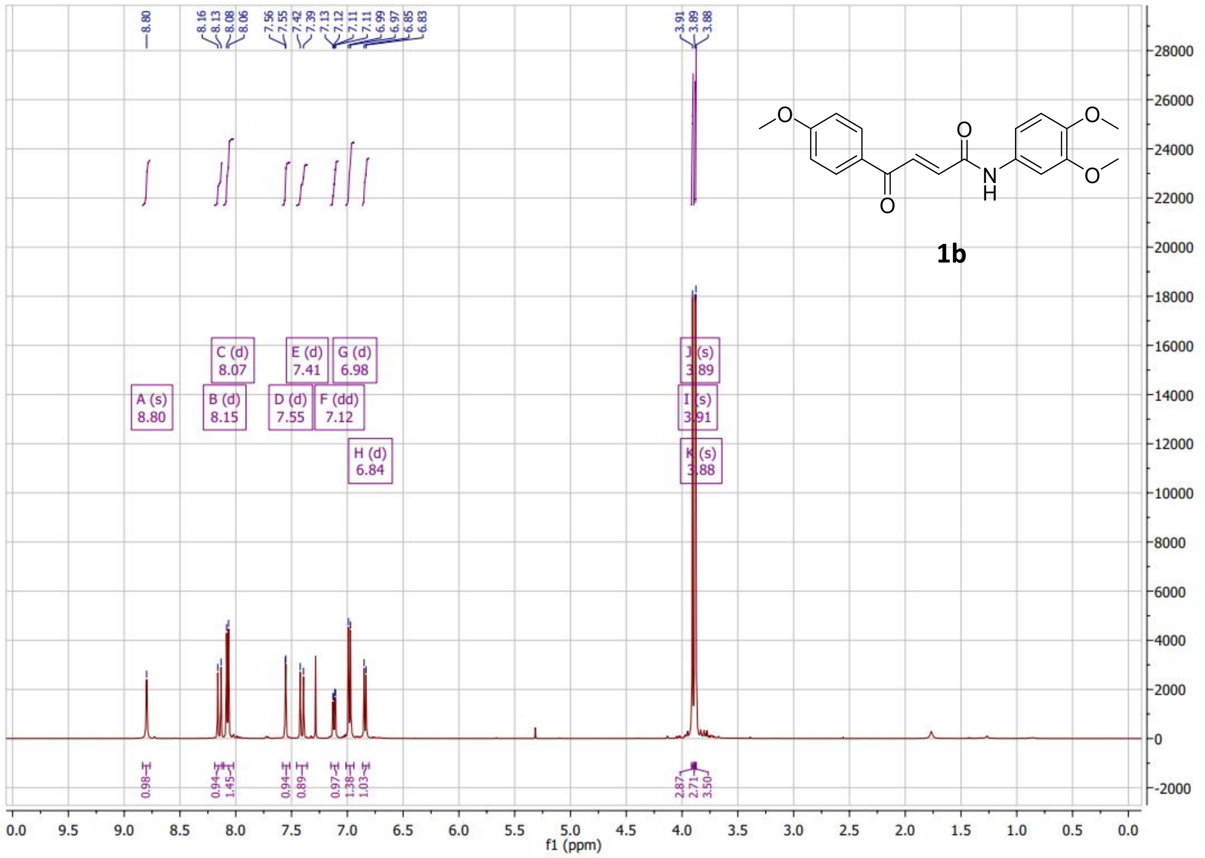

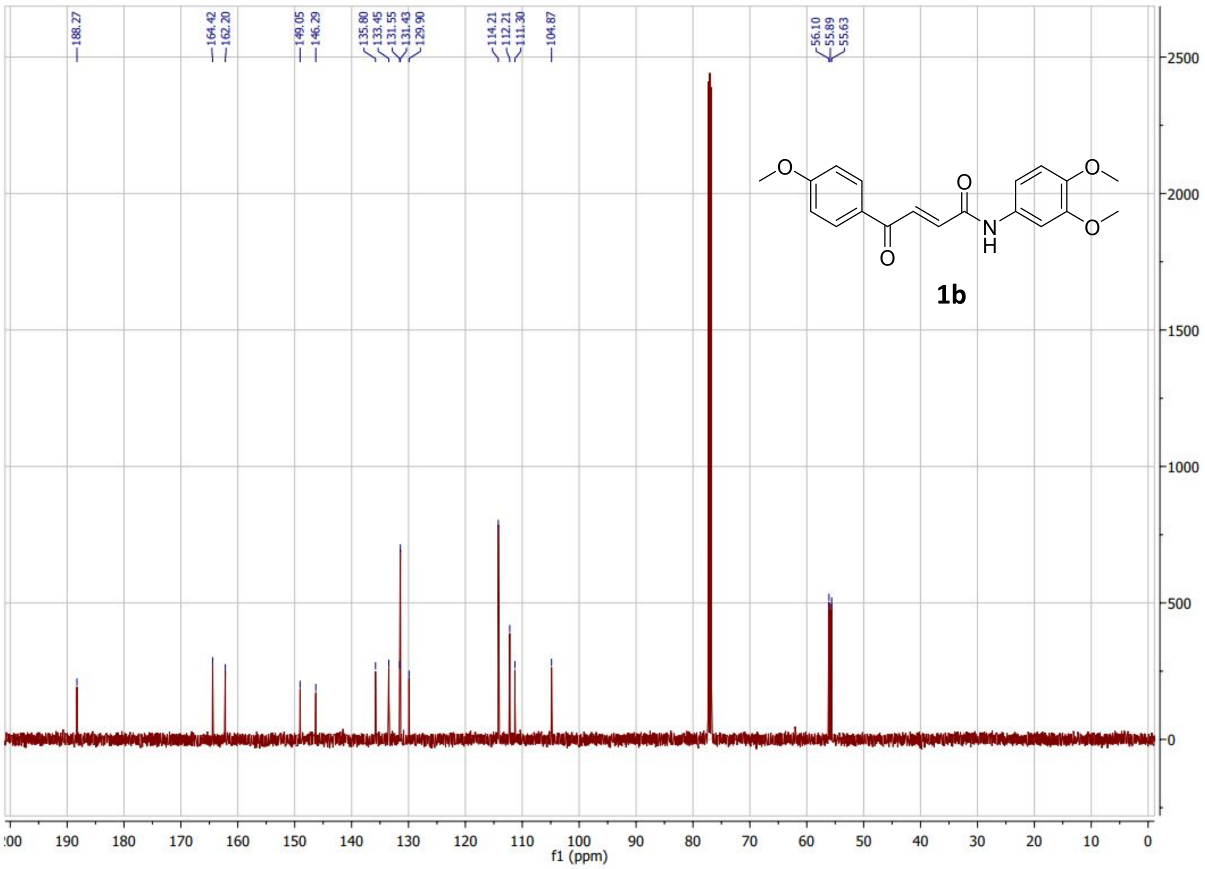

¹H and ¹³C NMR spectra of (*E*)-4-(4-Cyanophenyl)-4-oxo-*N*-phenylbut-2-enamide (1c)

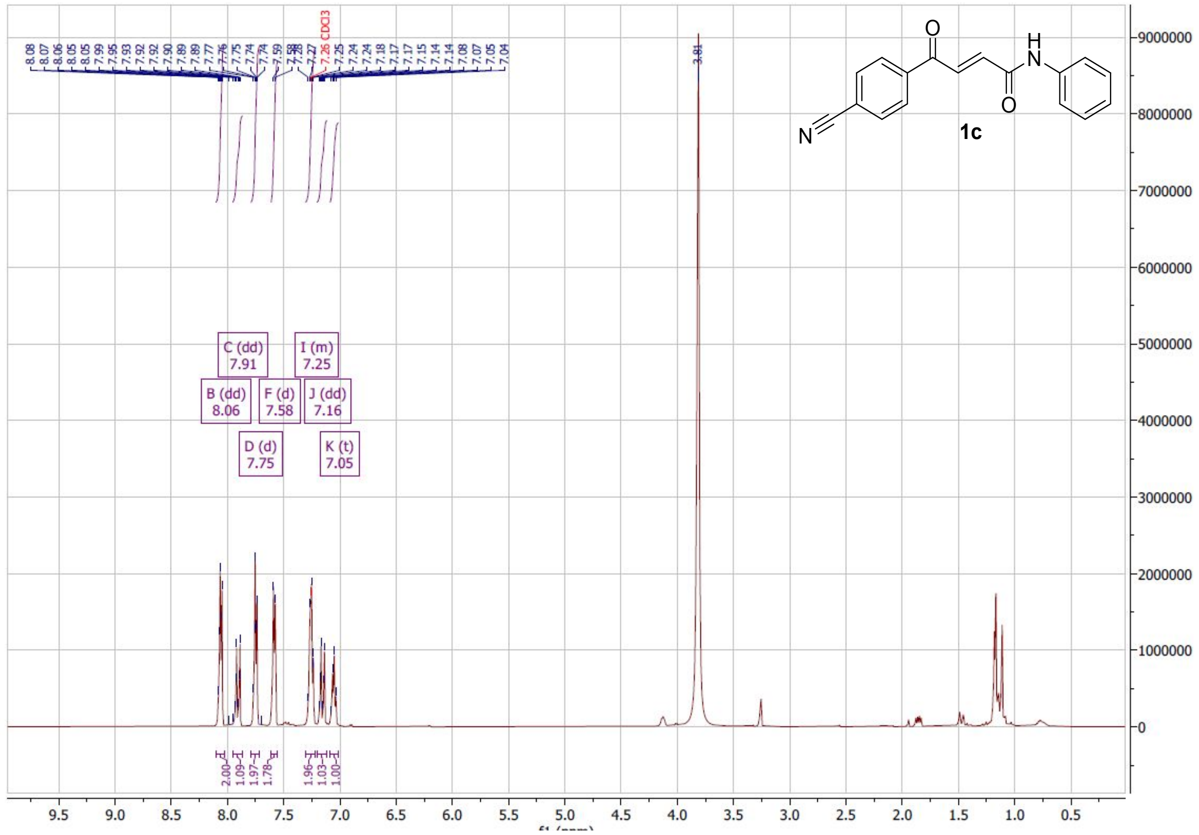

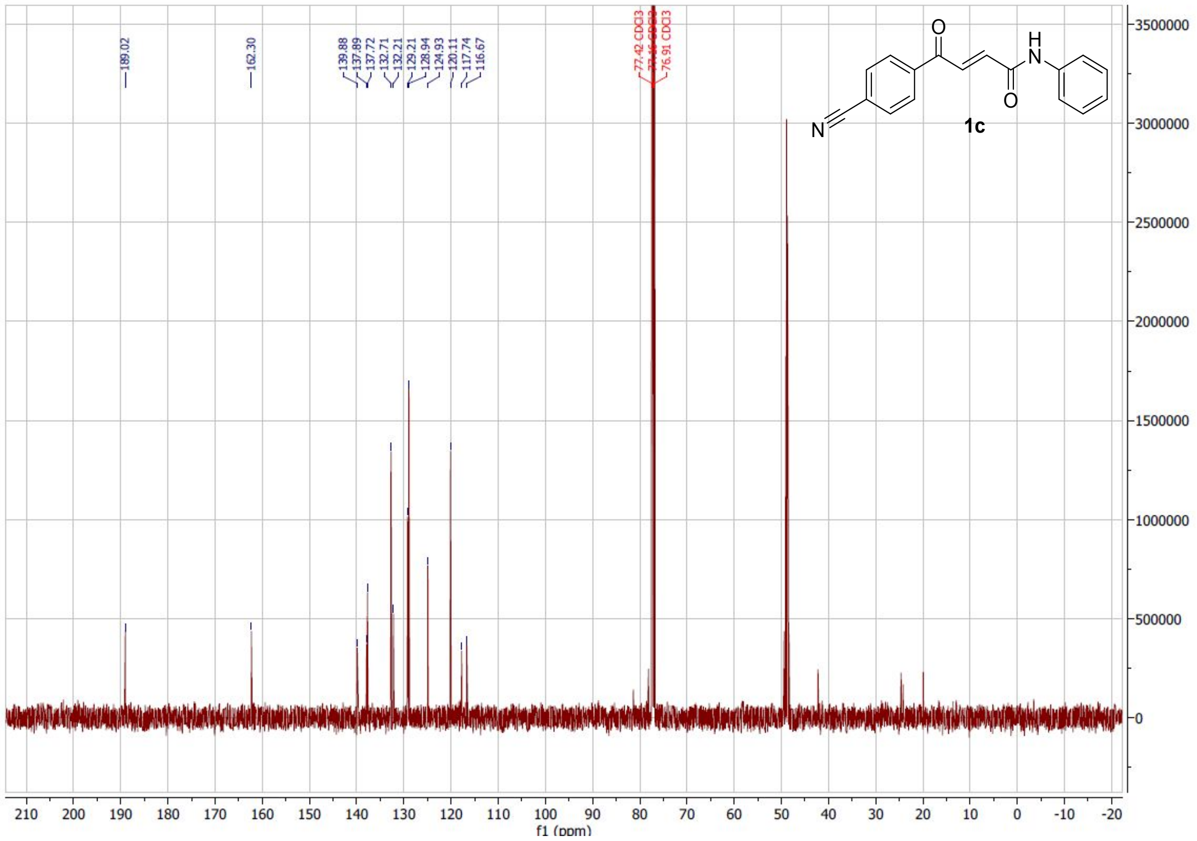

^{1}H and ^{13}C NMR spectra of (*E*)-4-Cyclohexyl-4-oxo-*N*-phenylbut-2-enamide (1d)

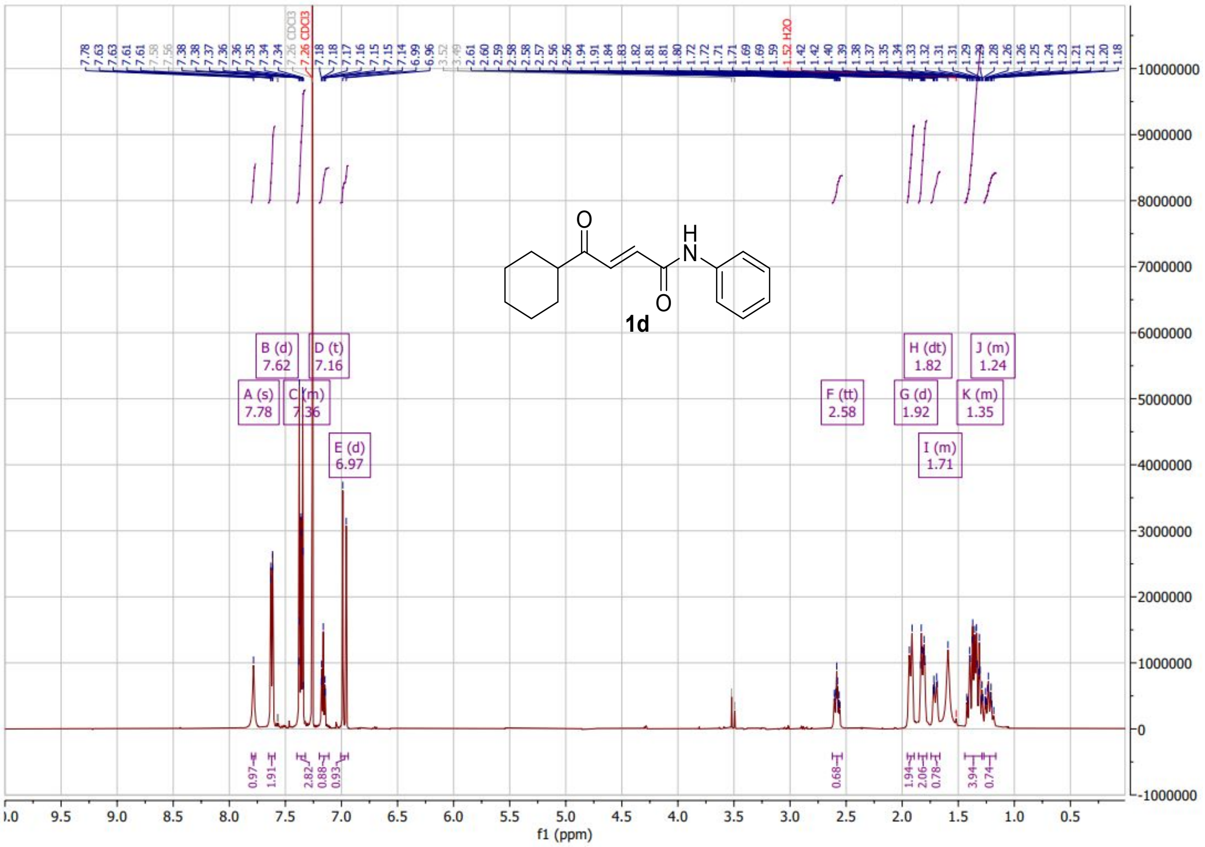

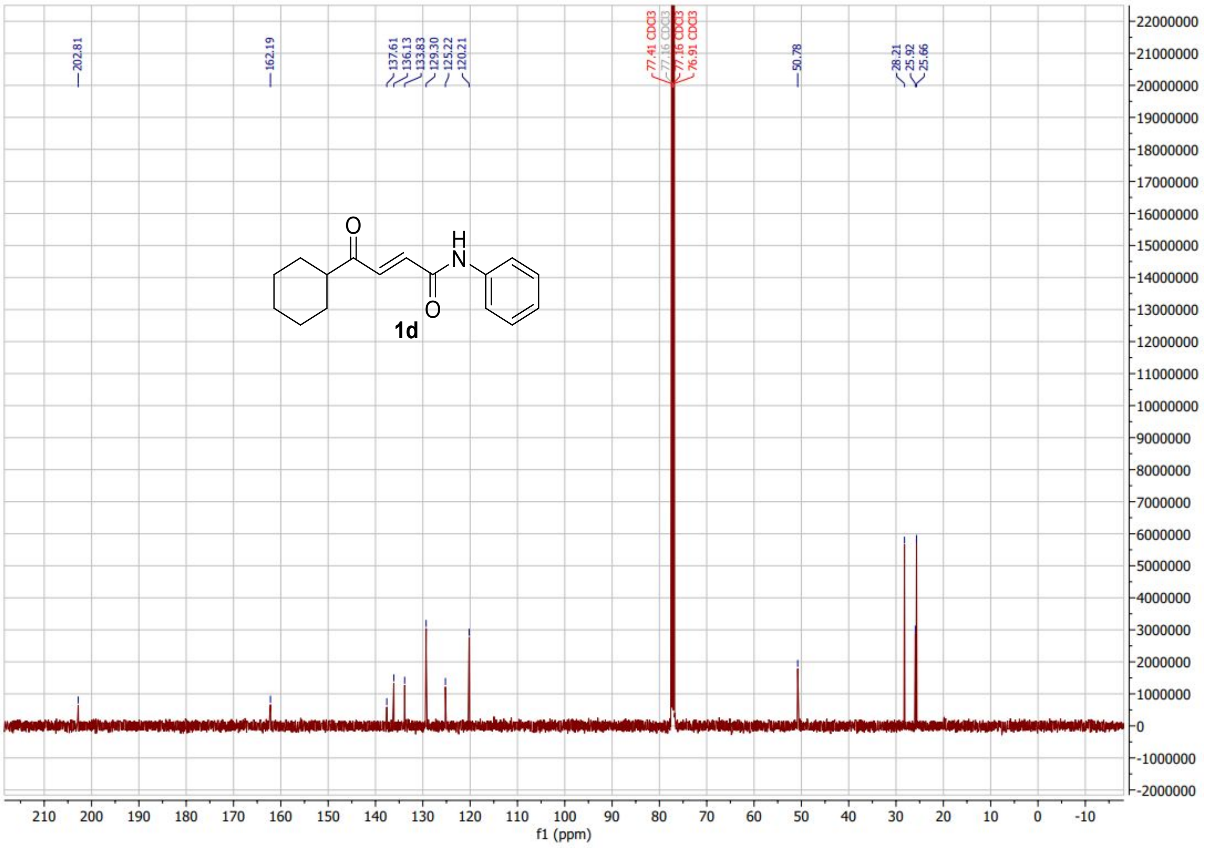

^{1}H and ^{13}C NMR spectra of (*E*)-4-oxo-*N*-phenyl-4-(tetrahydro-2*H*-pyran-4-yl)but-2-enamide (1e)

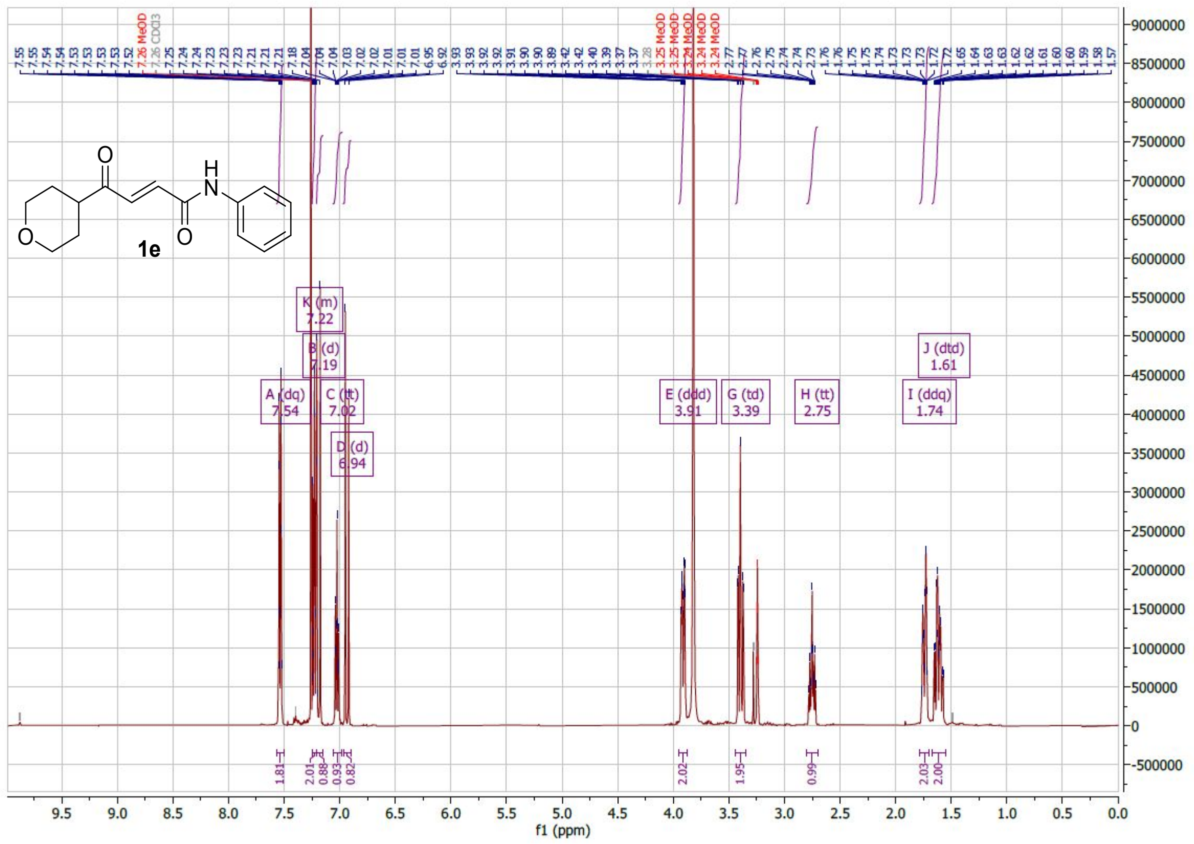

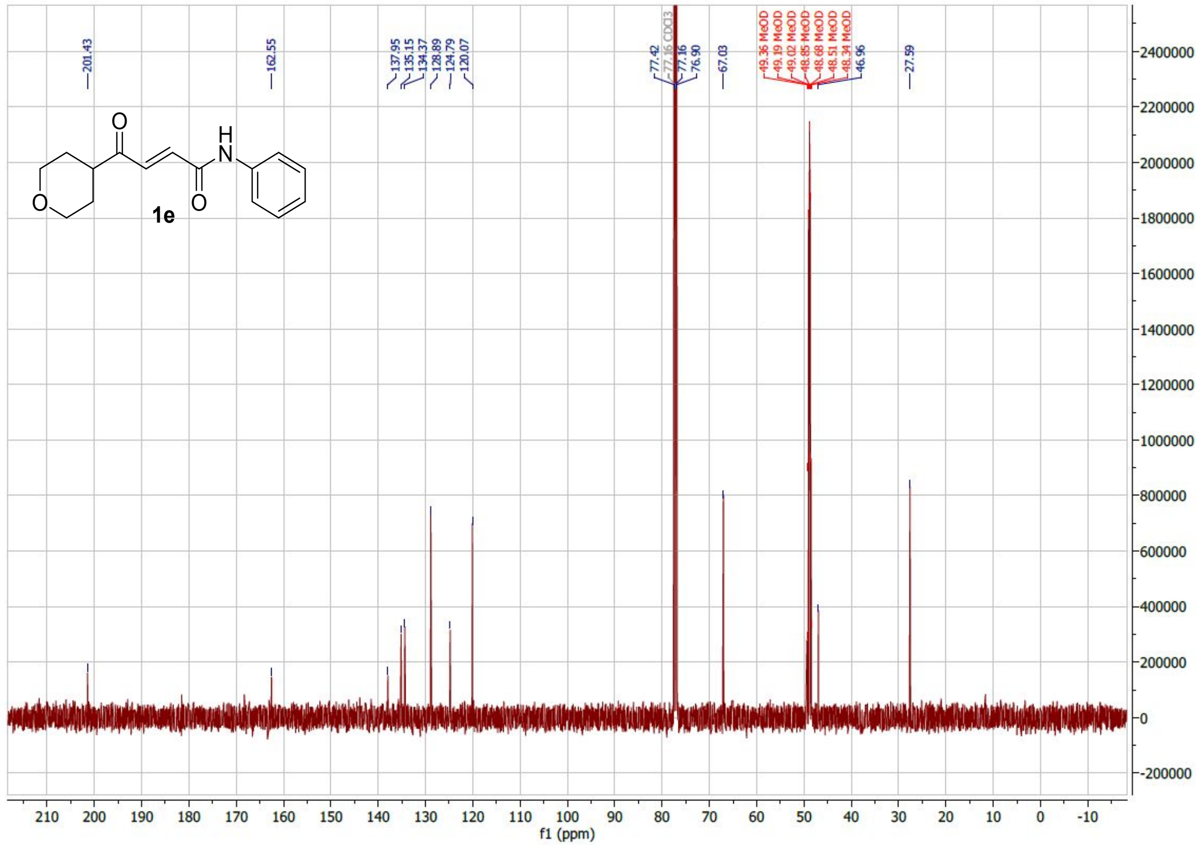

^{1}H and ^{13}C NMR spectra of (*E*)-4-(4-methoxyphenyl)-4-oxo-*N*-phenylbut-2-enamide (1f)

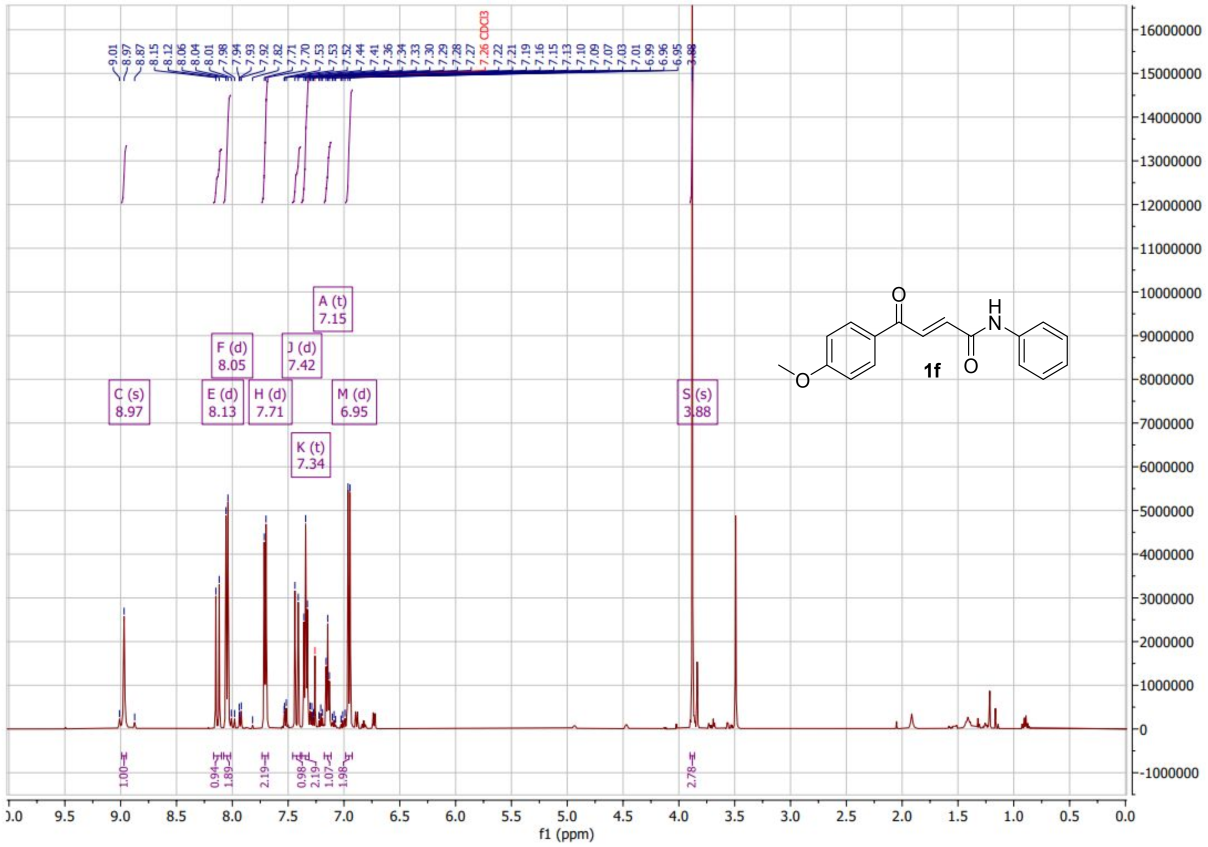

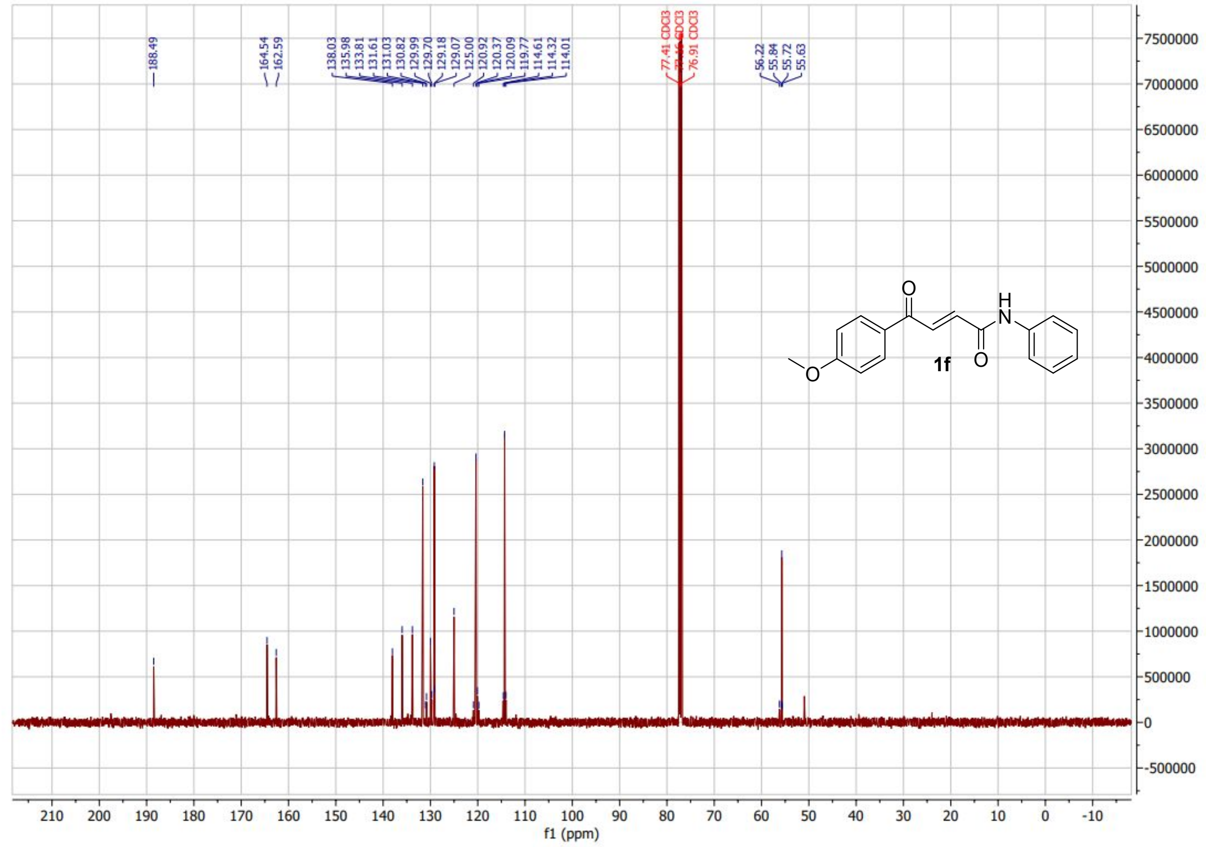

^{1}H and ^{13}C NMR spectra of (*E*)-1-Phenyl-4-(piperidin-1-yl)but-2-ene-1,4-dione (1h)

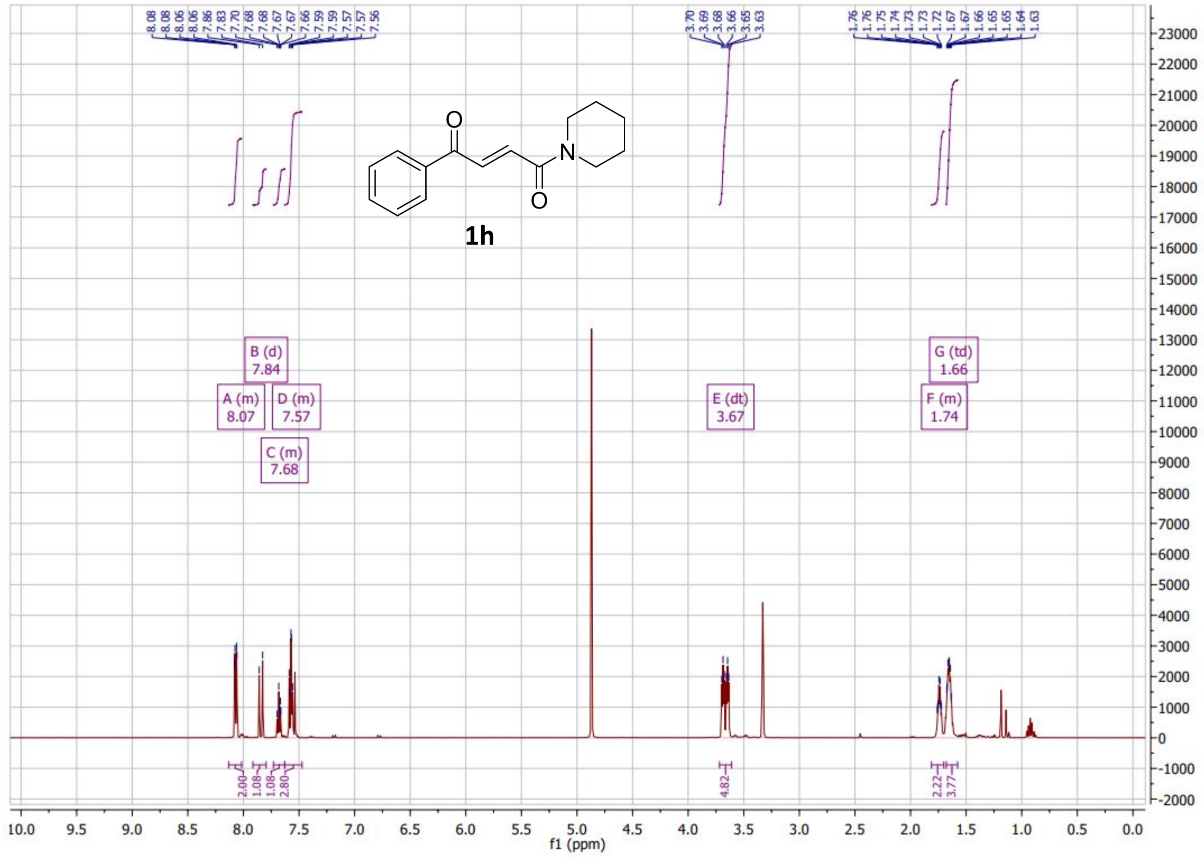

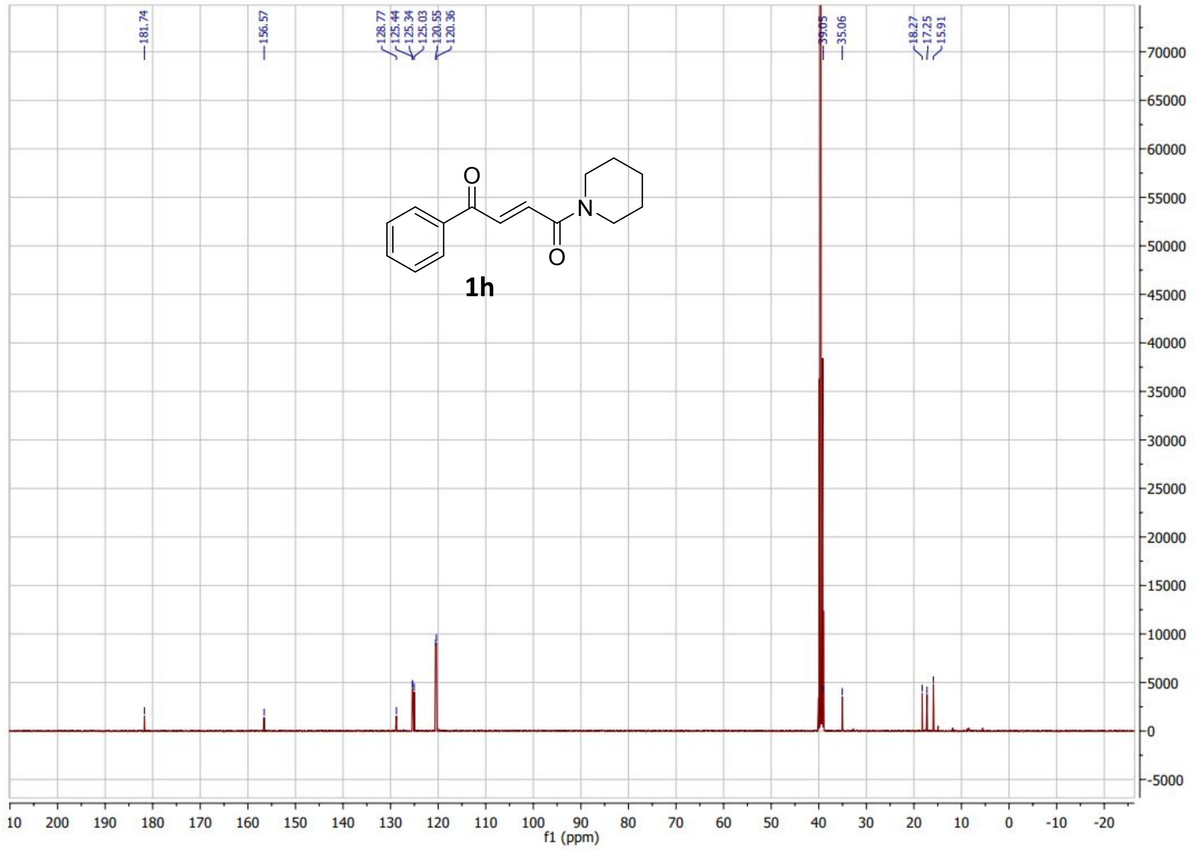

¹H and ¹³C NMR spectra of (*E*)-1-Morpholino-4-phenylbut-2-ene-1,4-dione (1i)

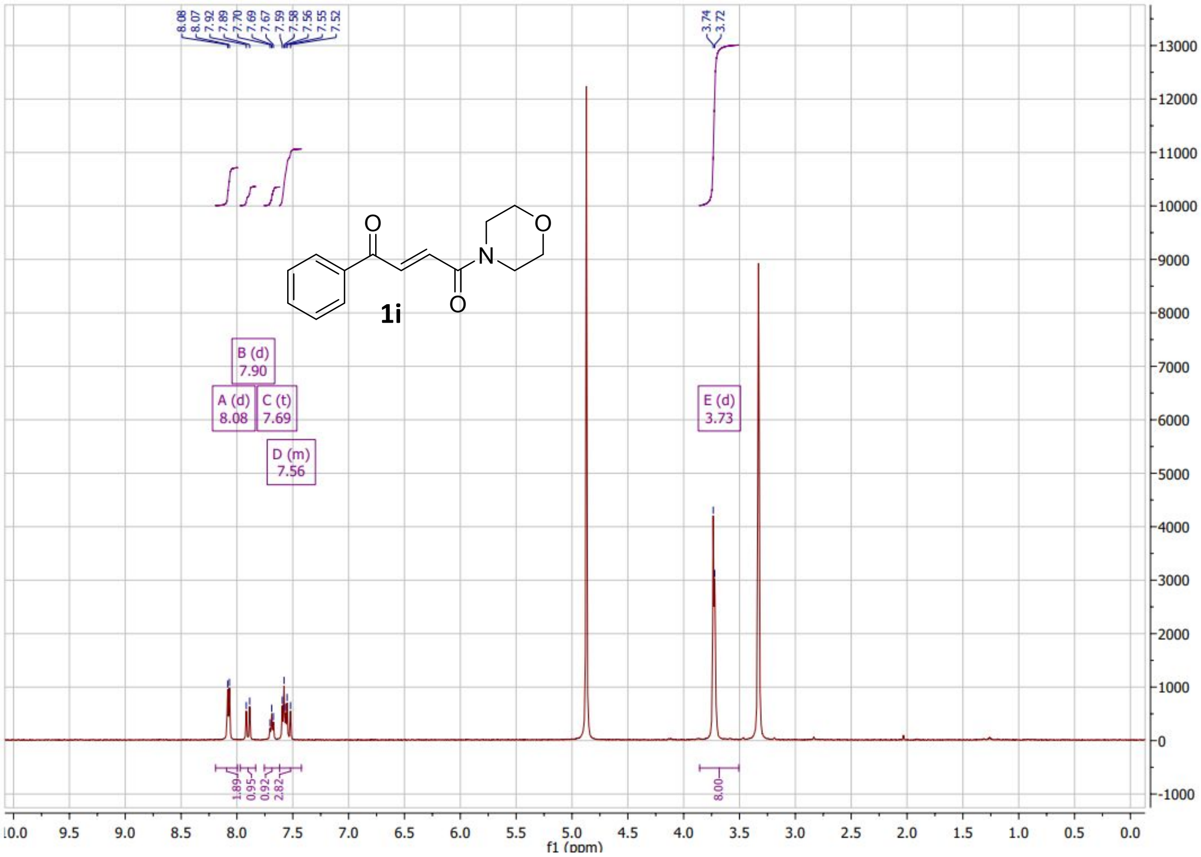

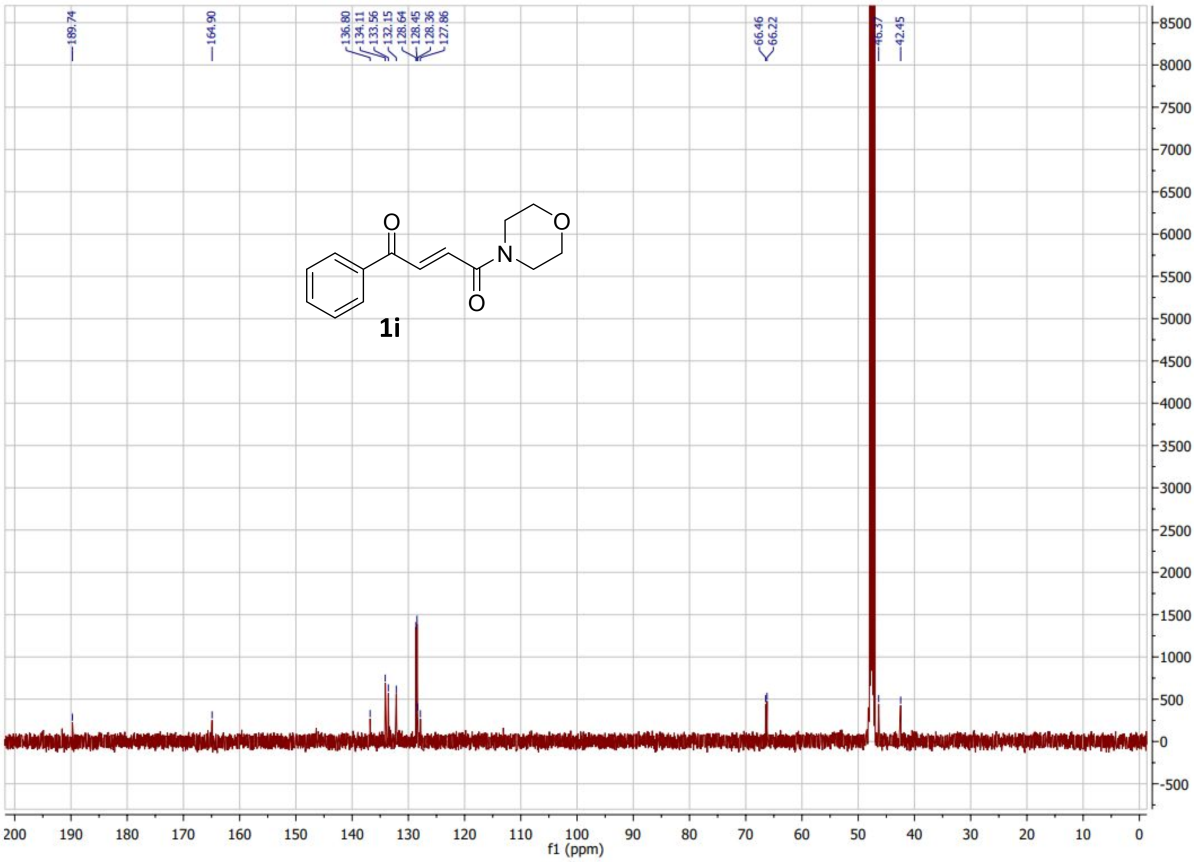

^{1}H and ^{13}C NMR spectra of (*E*)-*N*-(2-Methoxyethyl)-4-oxo-4-phenylbut-2-enamide (1j)

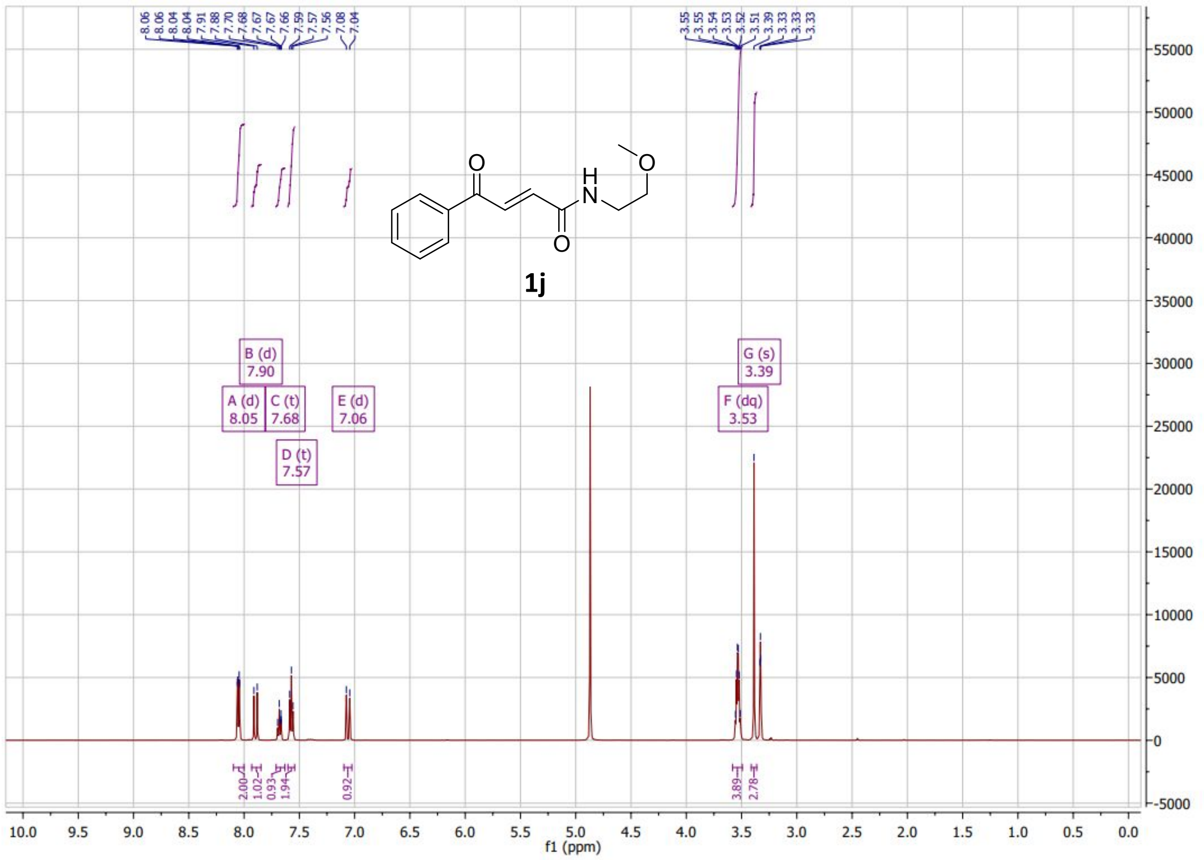

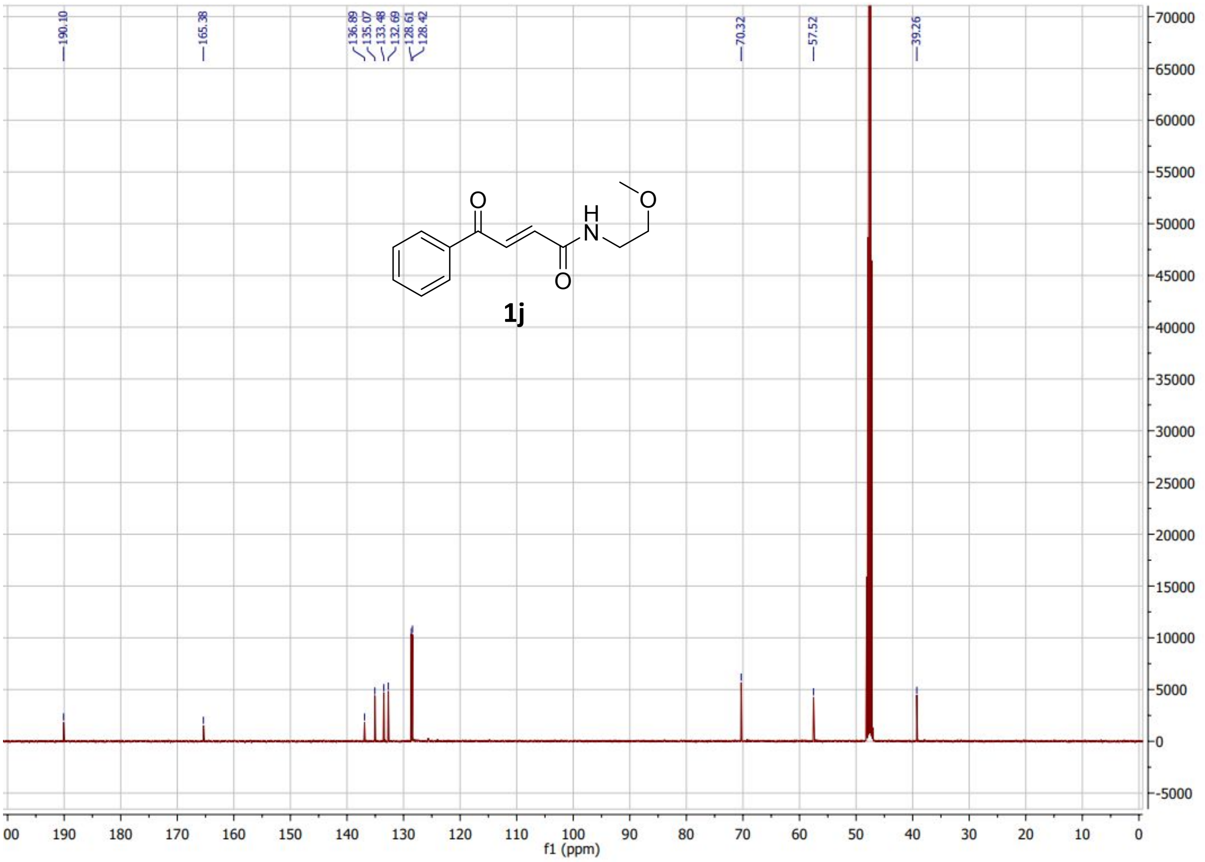

^{1}H and ^{13}C NMR spectra of (*E*)-*N*-(1-Methyl-1H-pyrazol-3-yl)-4-oxo-4-phenylbut-2-enamide (1k)

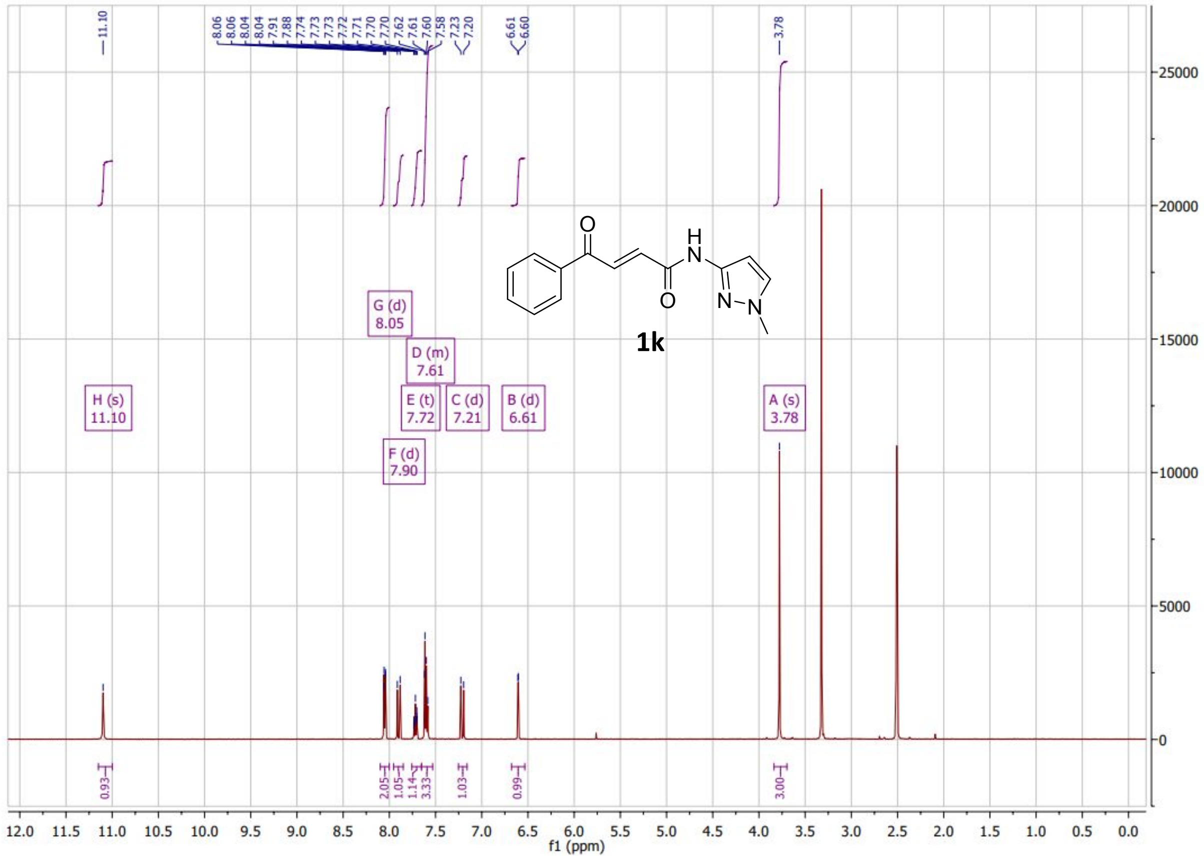

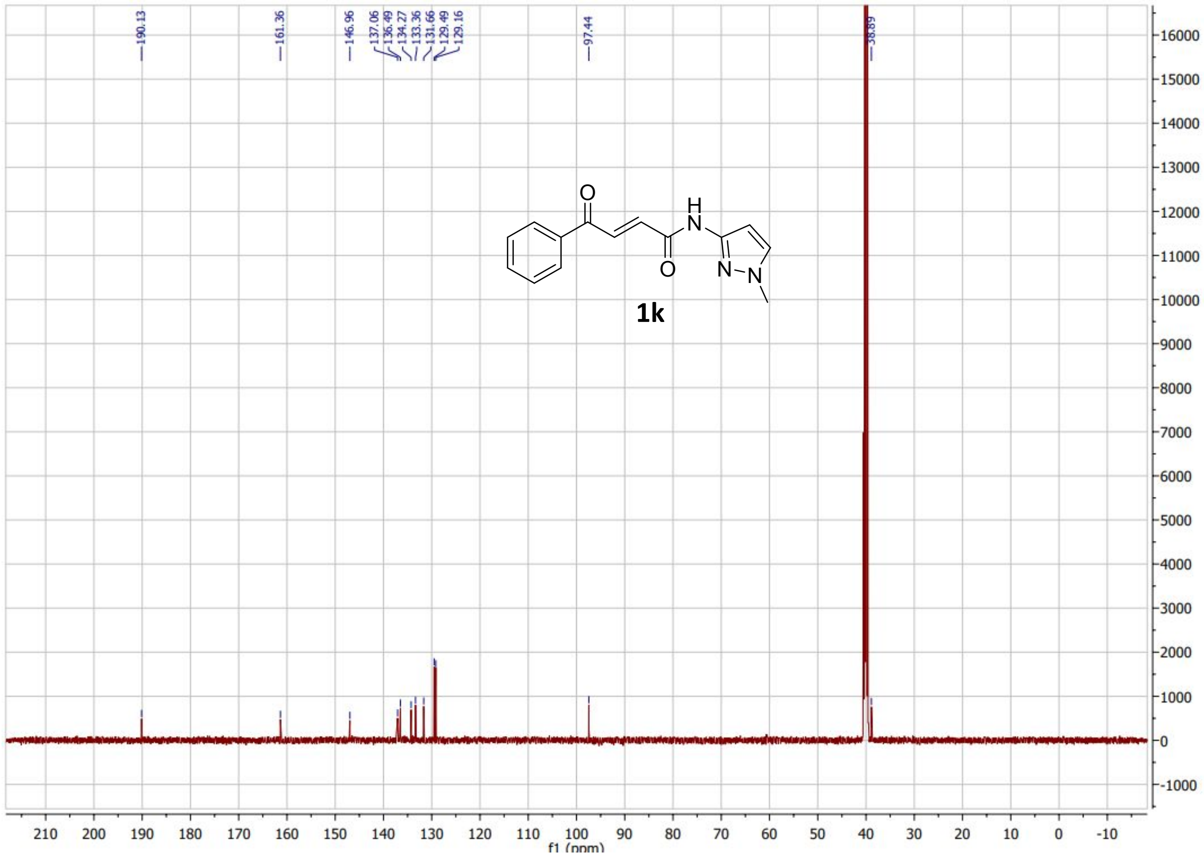

¹H and ¹³C NMR spectra of (*E*)-*N*-Benzyl-4-oxo-4-phenylbut-2-enamide (1l)

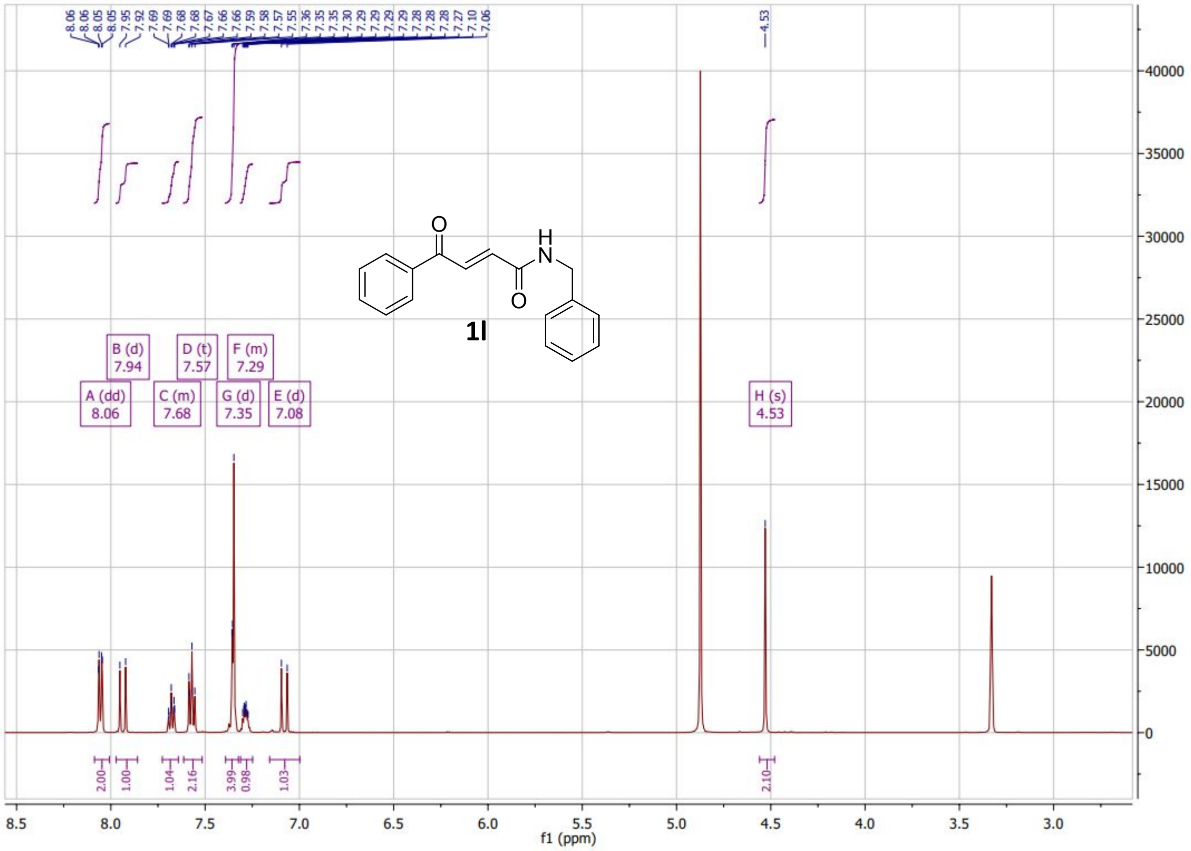

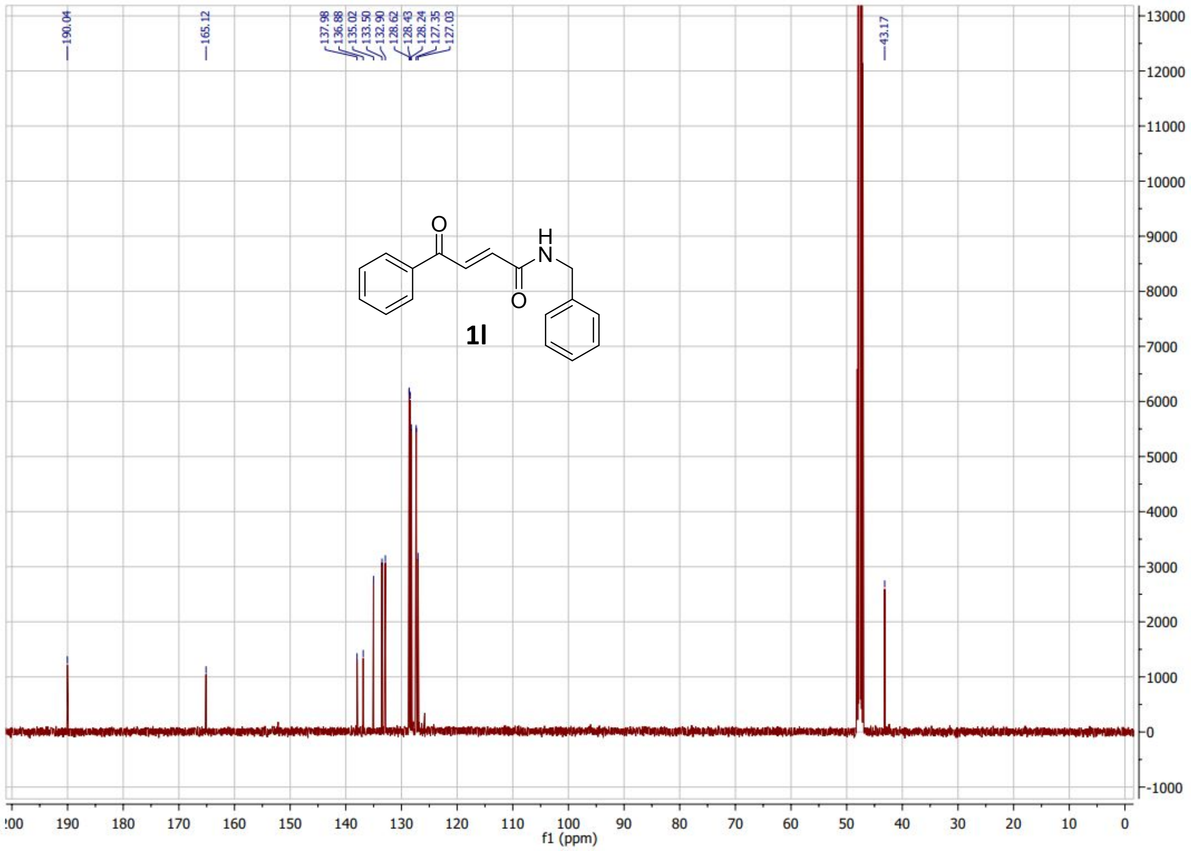

2.5.3. Transformations: 1,4-Cyclisations

^{1}H and ^{13}C NMR spectra of *N*,3-diphenyl-1*H*-pyrazole-5-carboxamide (7a)

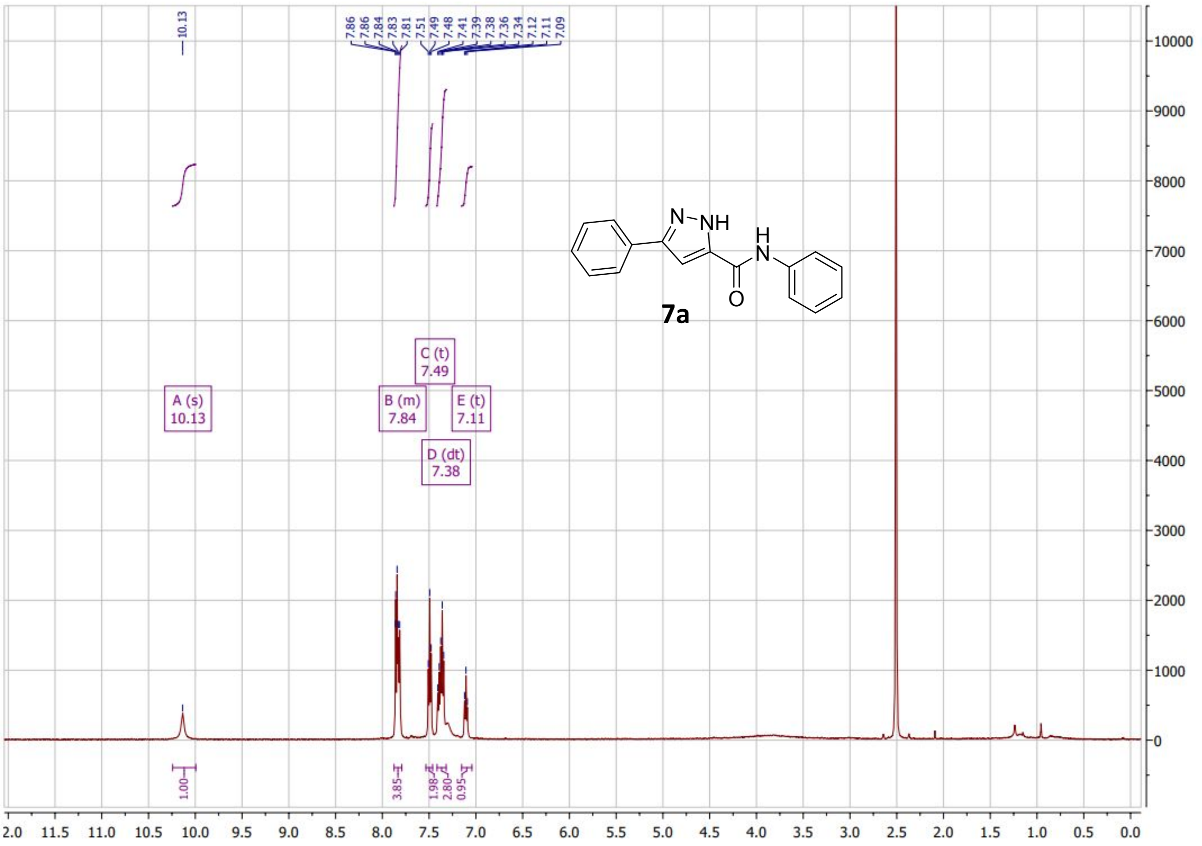

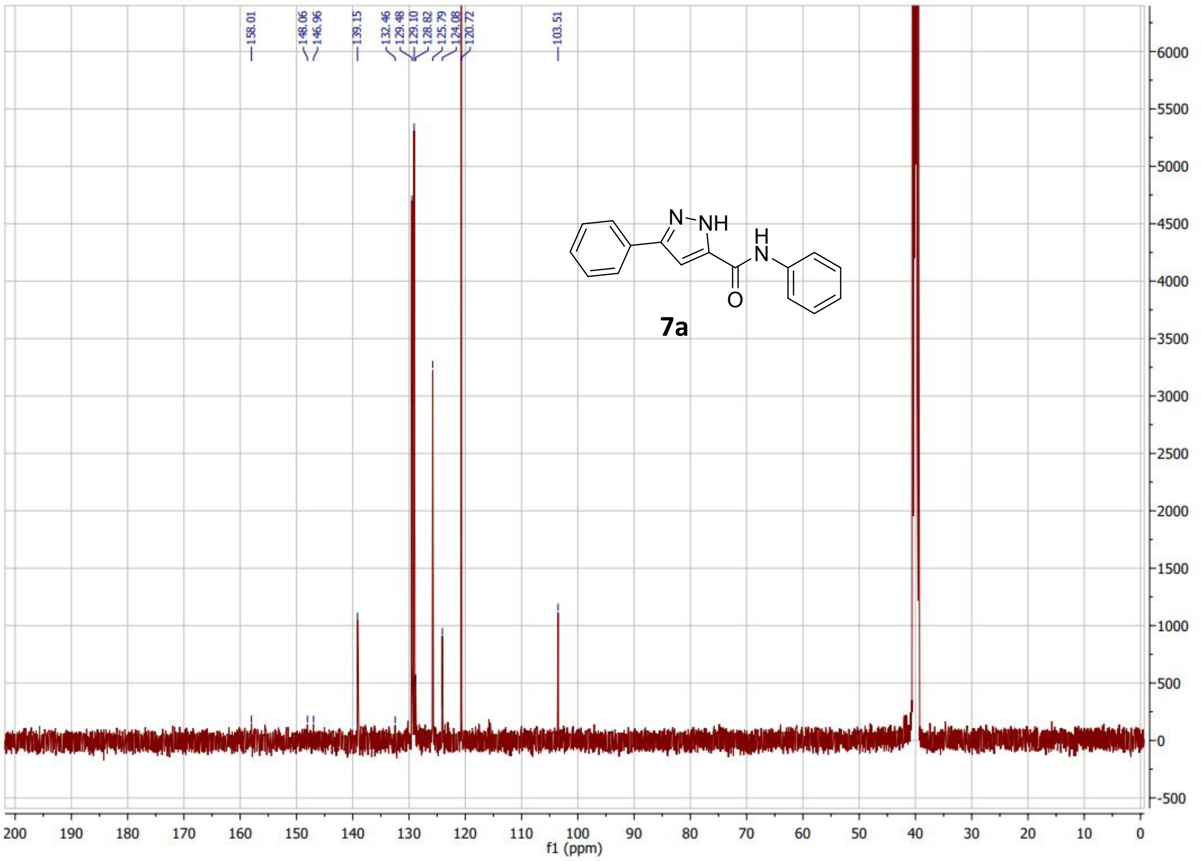

^{1}H and ^{13}C NMR spectra of *N*-(3,4-dimethoxyphenyl)-3-(4-methoxyphenyl)-1*H*-pyrazole-5-carboxamide (7b)

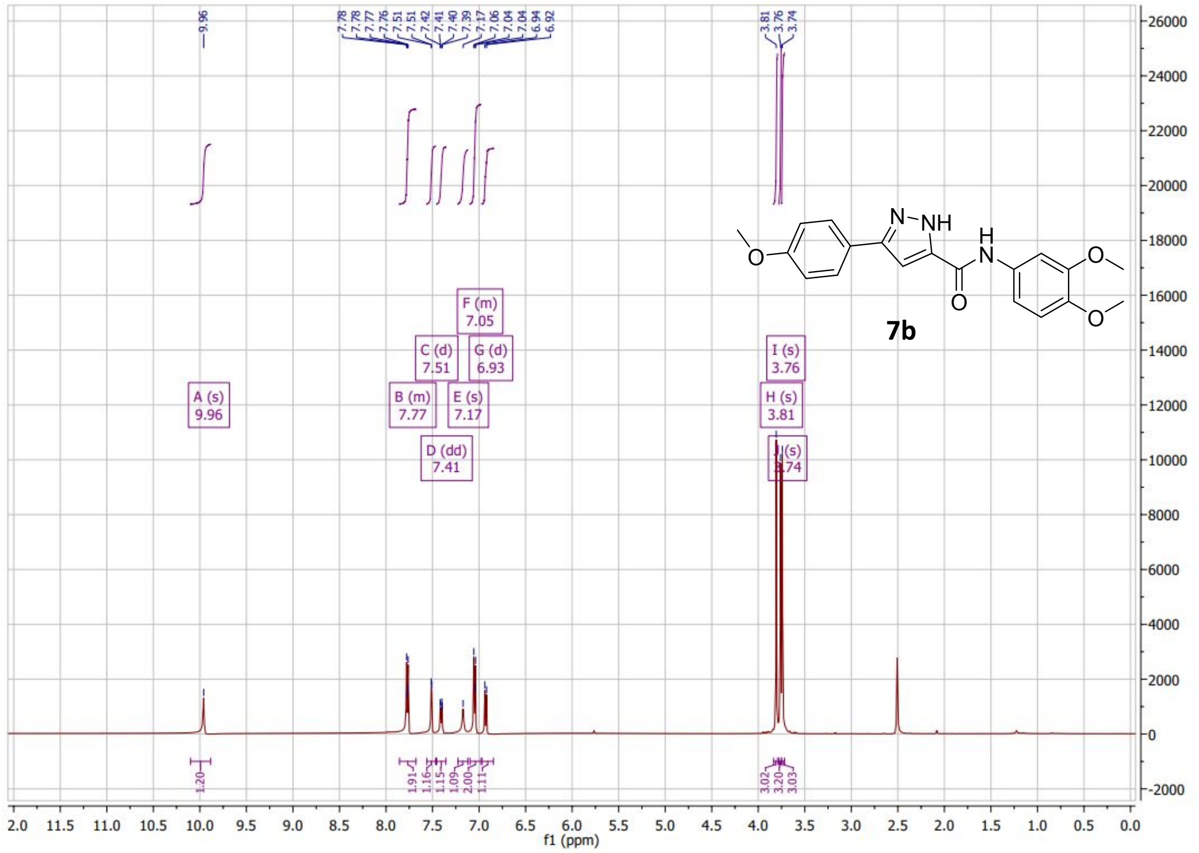

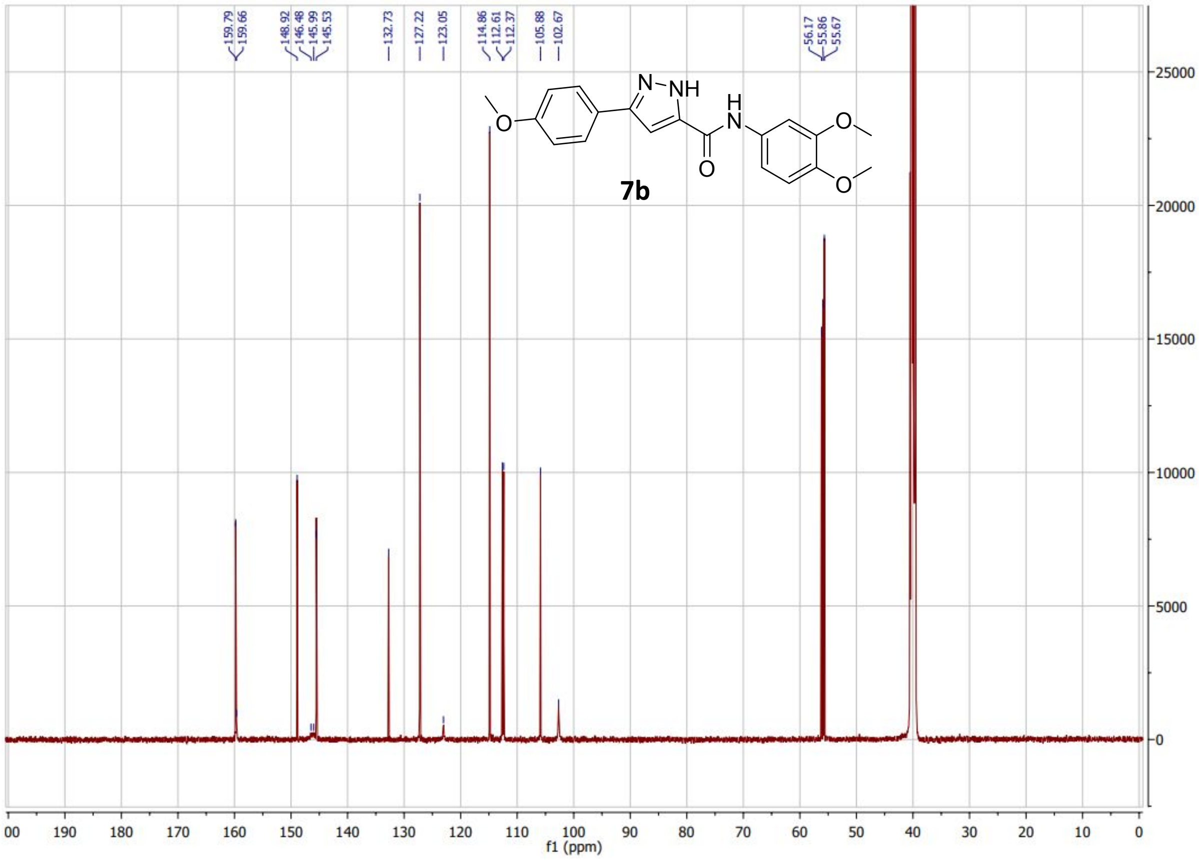

^{1}H and ^{13}C NMR spectra of *N*,3-Diphenylisoxazole-5-carboxamide (8a)

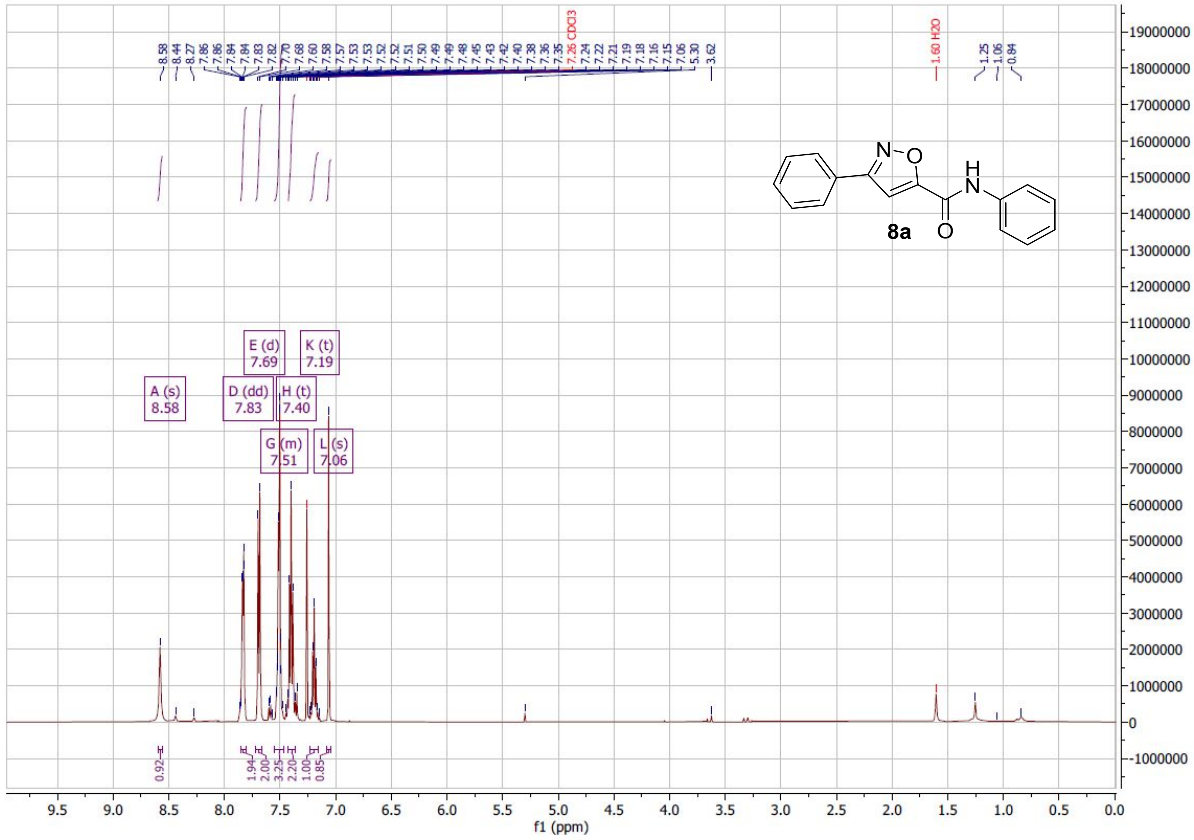

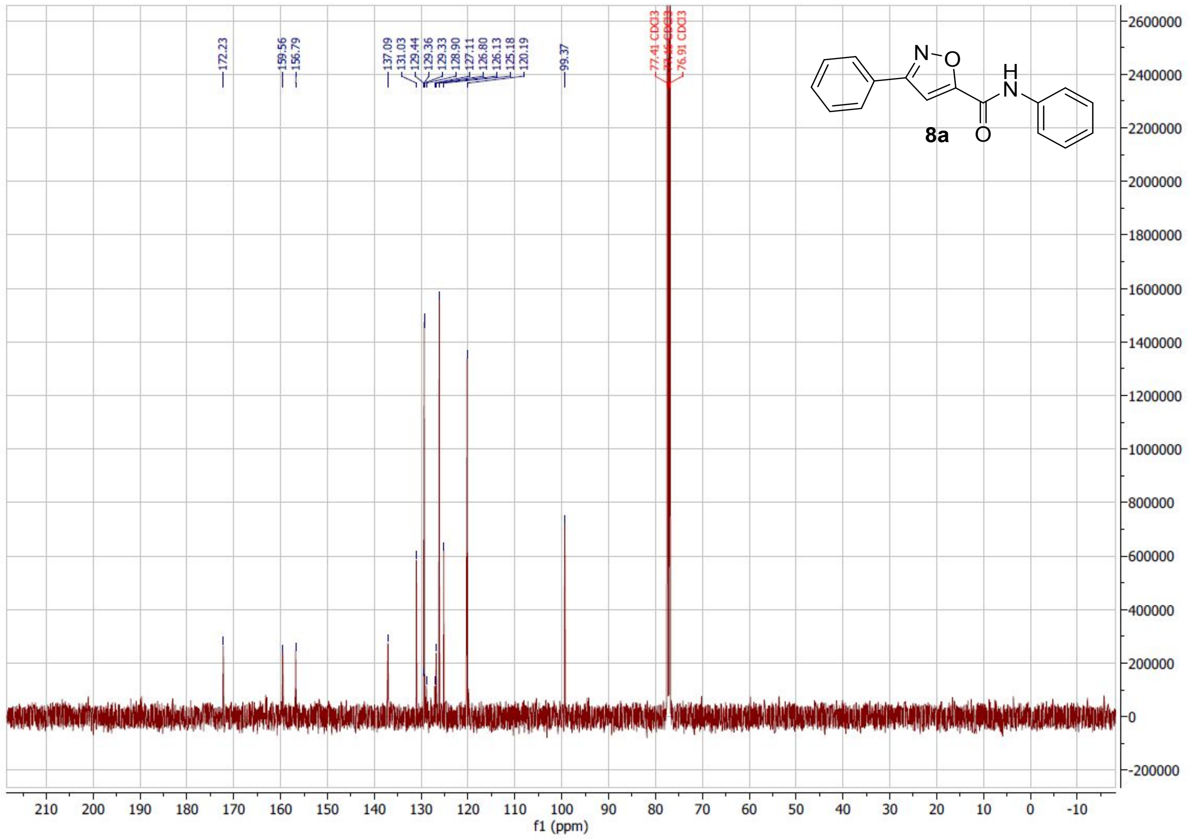

¹H and ¹³C NMR spectra of *N*,5-Diphenylpyrazolo[1,5-*a*]pyrimidine-7-carboxamide (9a)

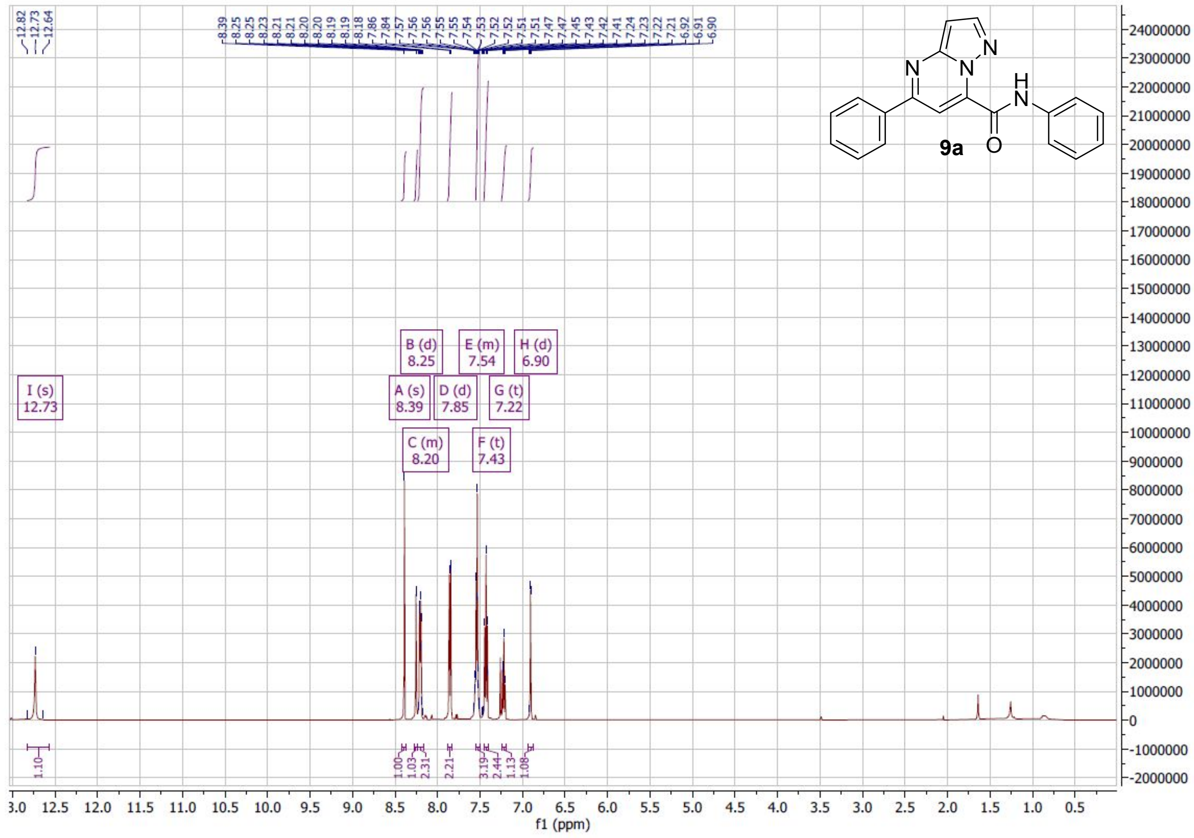

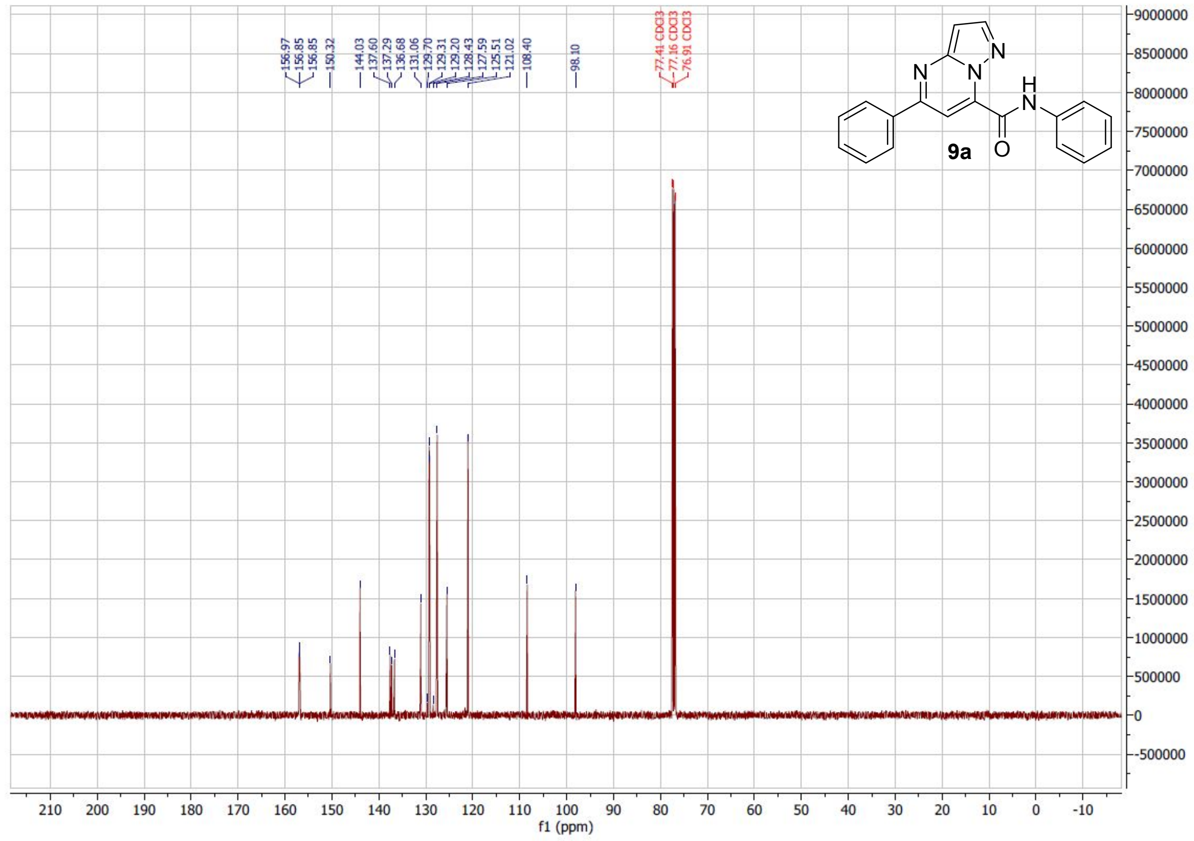

^{1}H and ^{13}C NMR spectra of *N*-(3,4-dimethoxyphenyl)-5-(4-methoxyphenyl)pyrazolo[1,5-*a*]pyrimidine-7-carboxamide (9b)

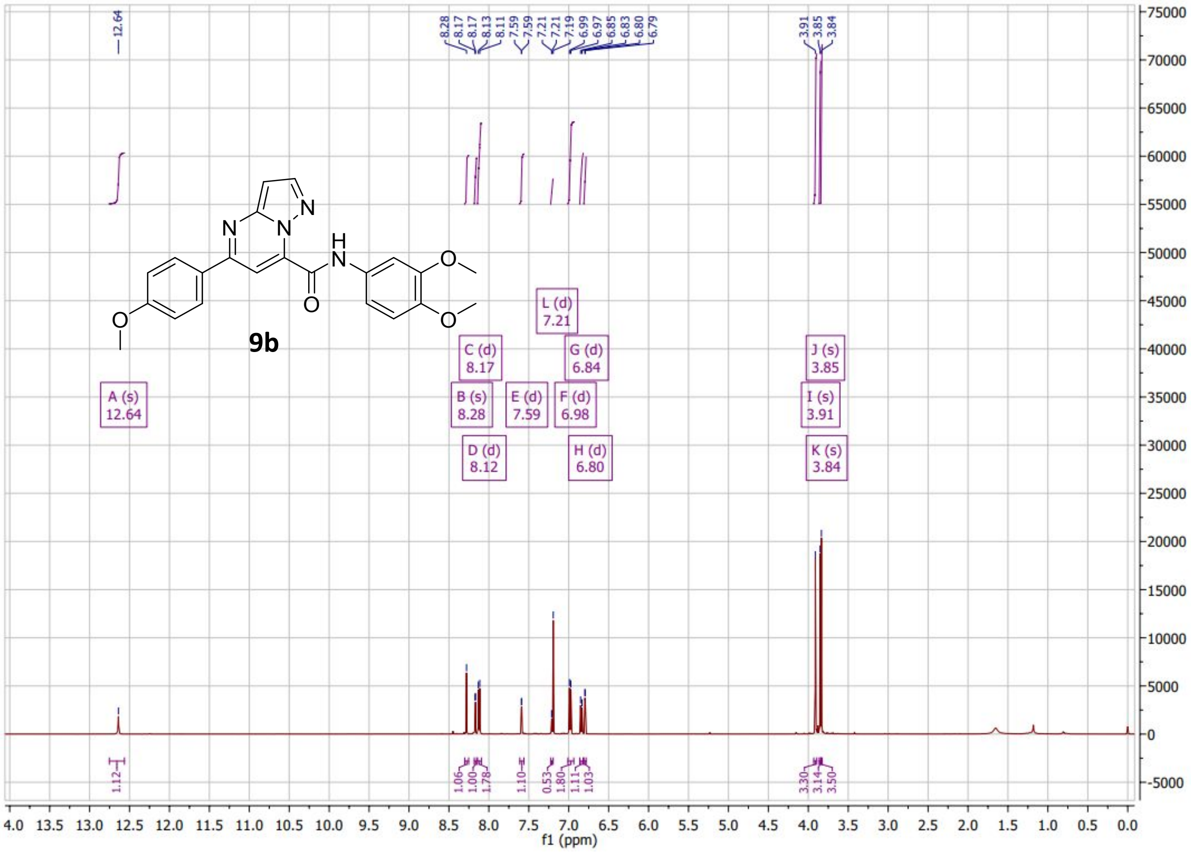

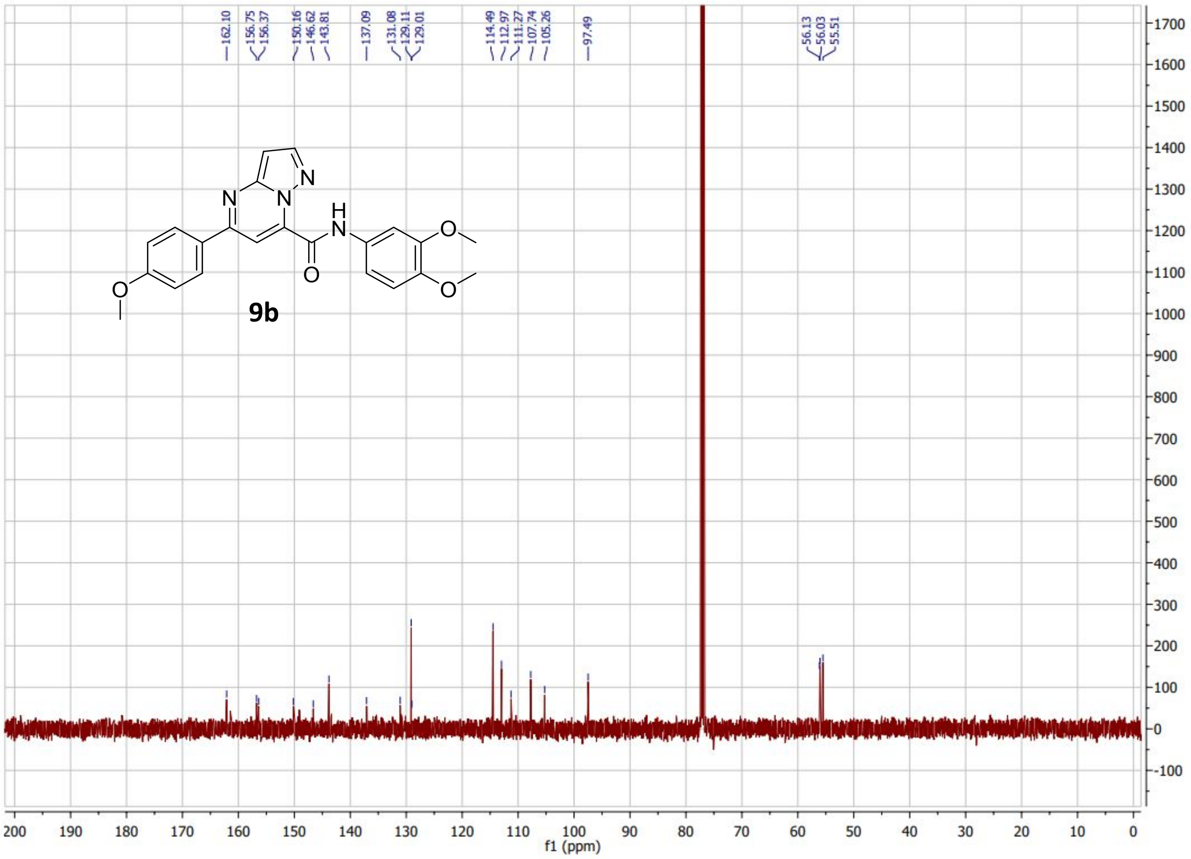

^{1}H and ^{13}C NMR spectra of *N*,7-Diphenylimidazo[1,2-*a*]pyridine-5-carboxamide (10a)

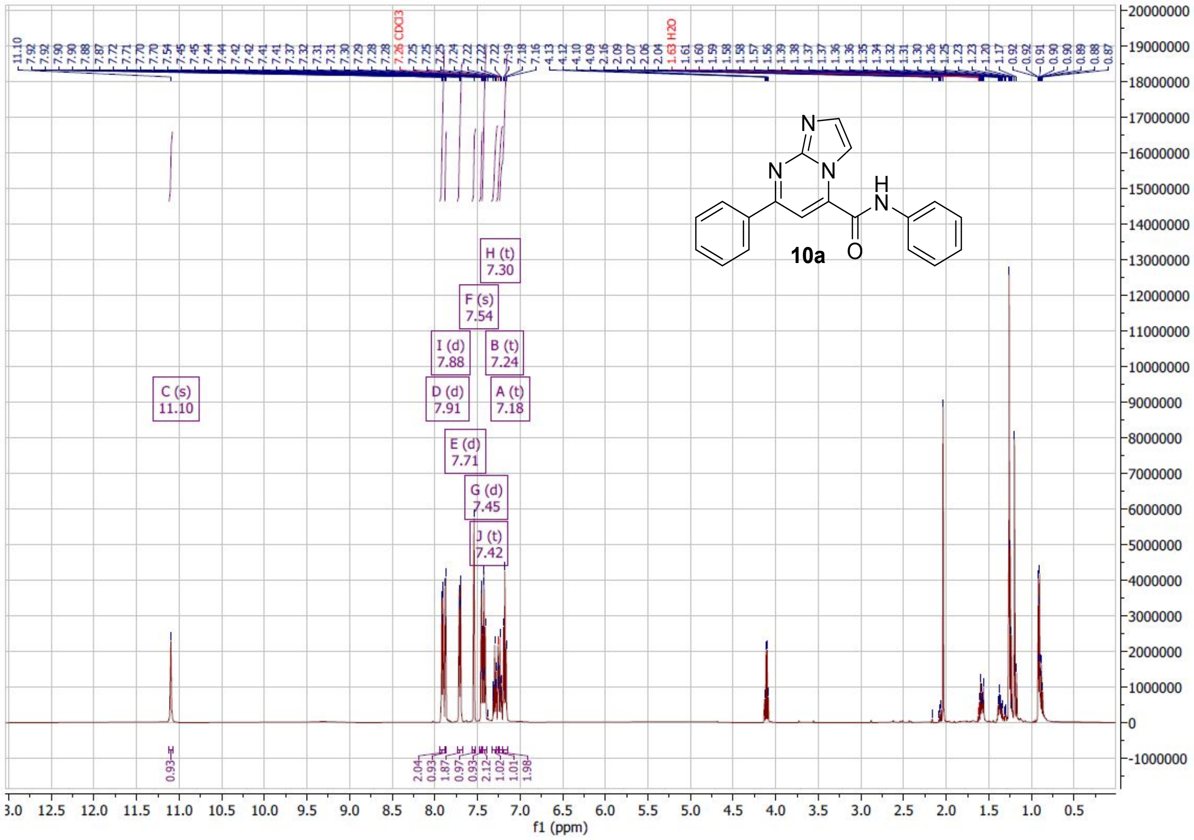

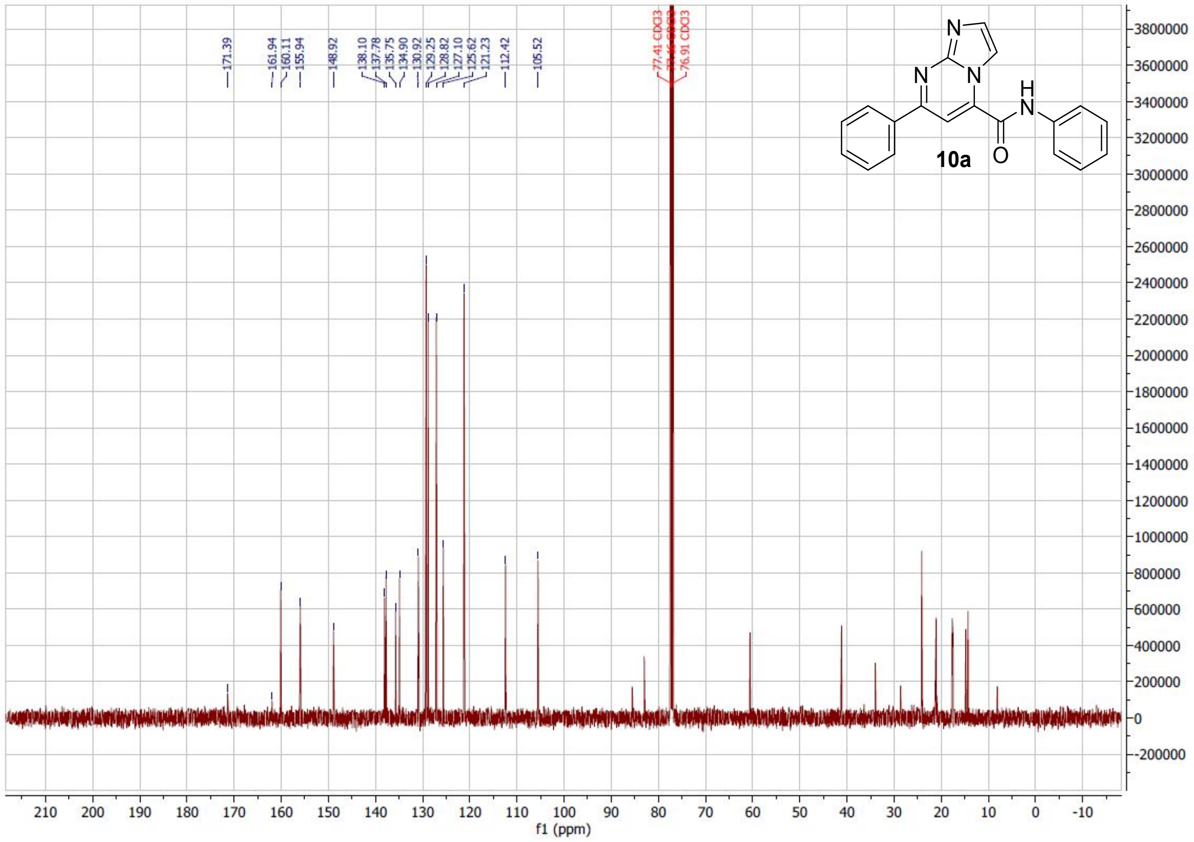

^{1}H and ^{13}C NMR spectra of *N*-(3,4-dimethoxyphenyl)-7-(4-methoxyphenyl)imidazo[1,2-*a*]pyrimidine-5-carboxamide (10b)

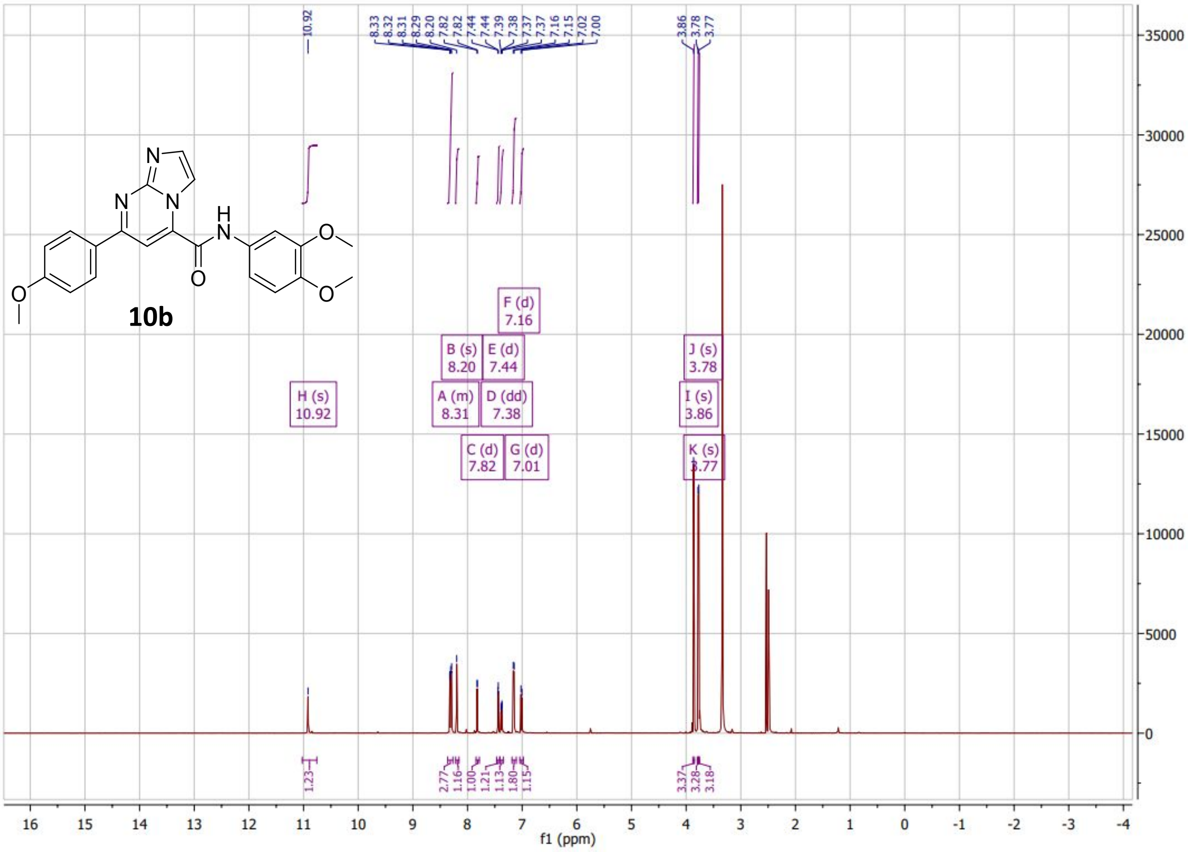

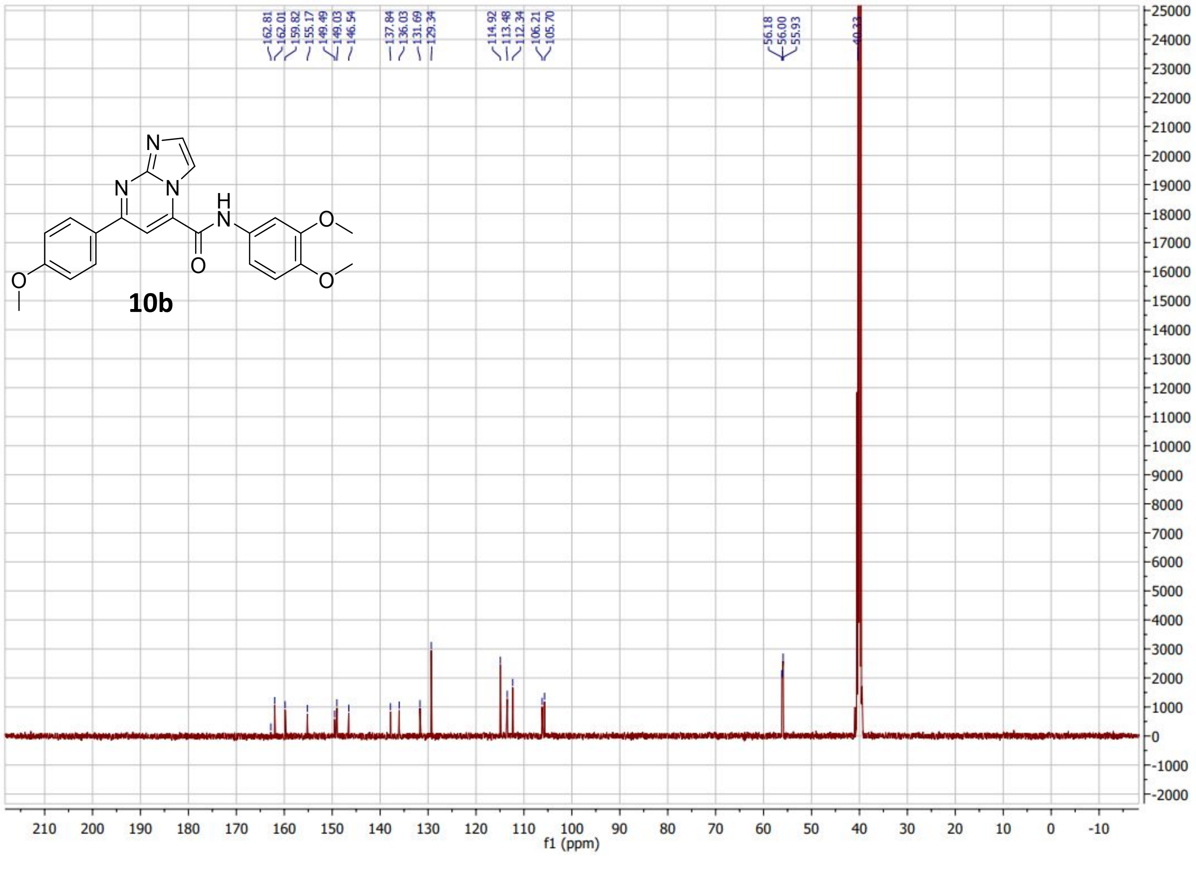

^{1}H and ^{13}C NMR spectra of methyl carbamimidate hydrochloride (11’)

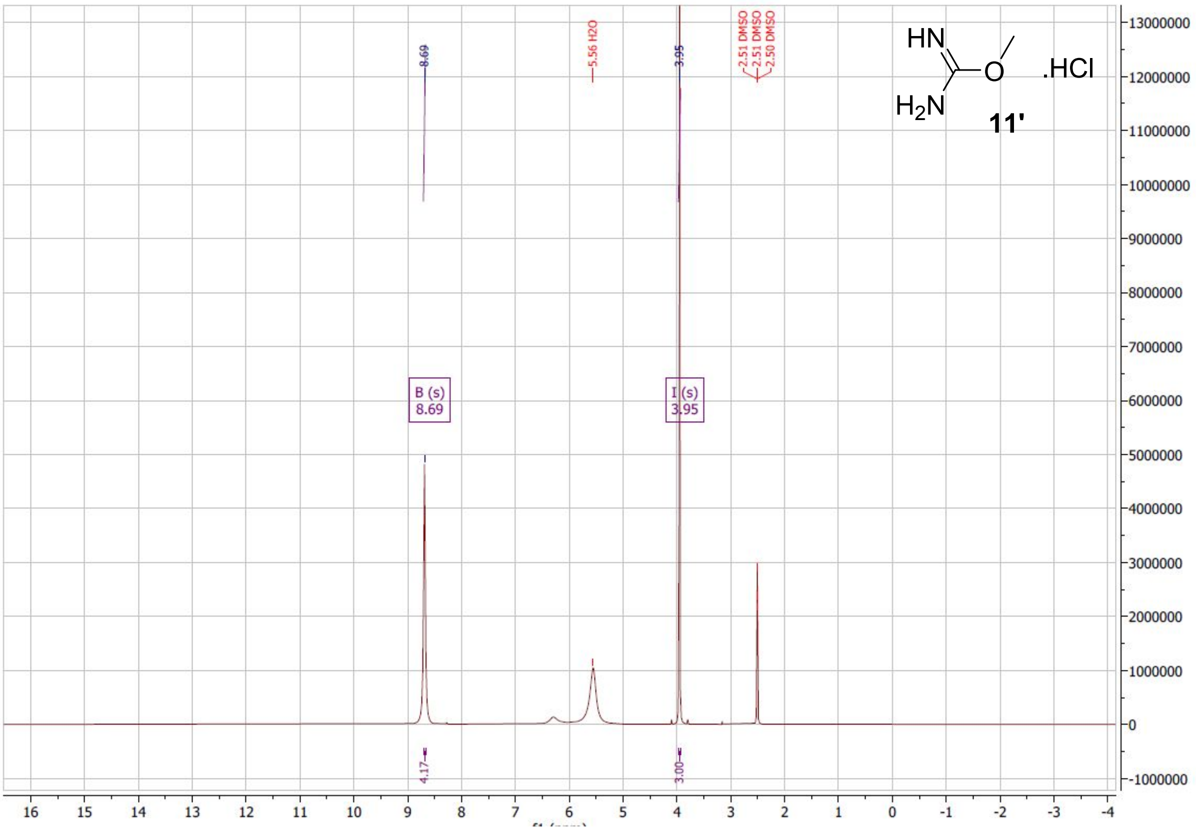

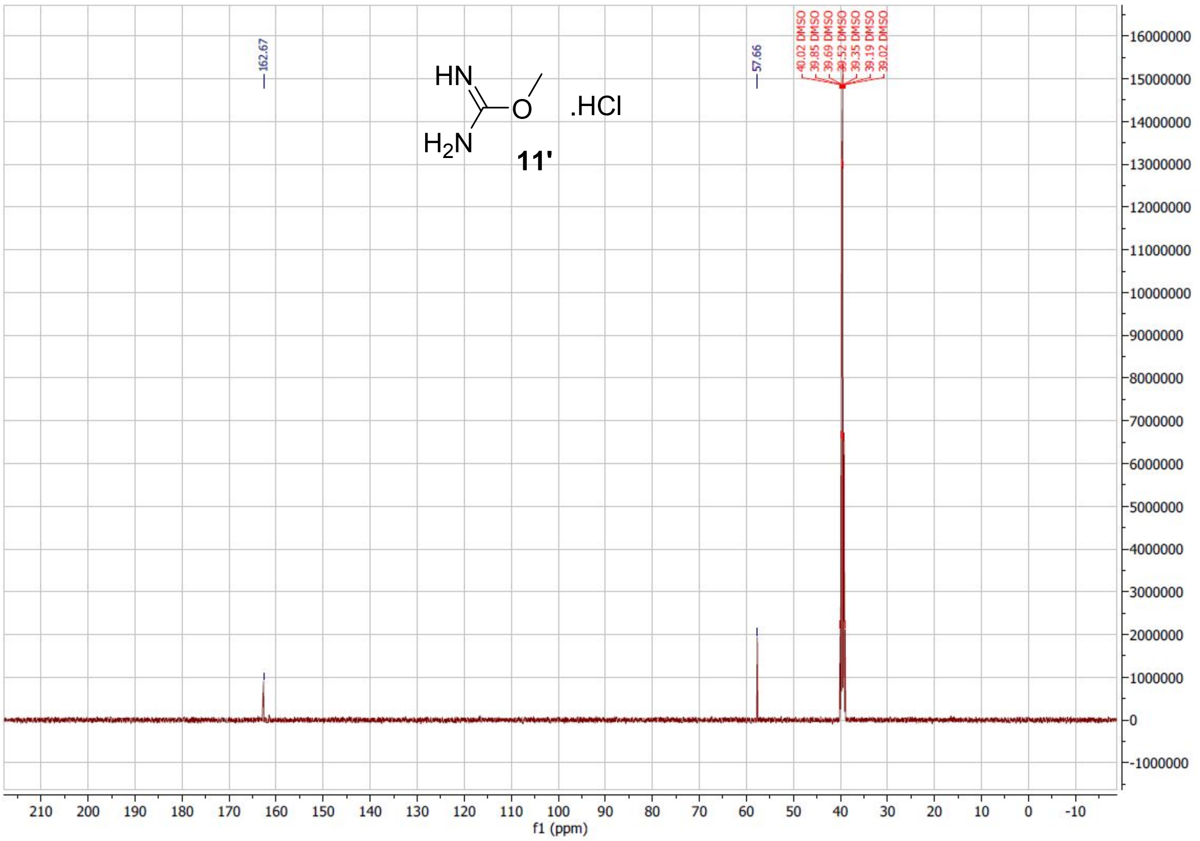

^{1}H and ^{13}C NMR spectra of 2-Methoxy-*N*,6-diphenylpyrimidine-4-carboxamide (11a)

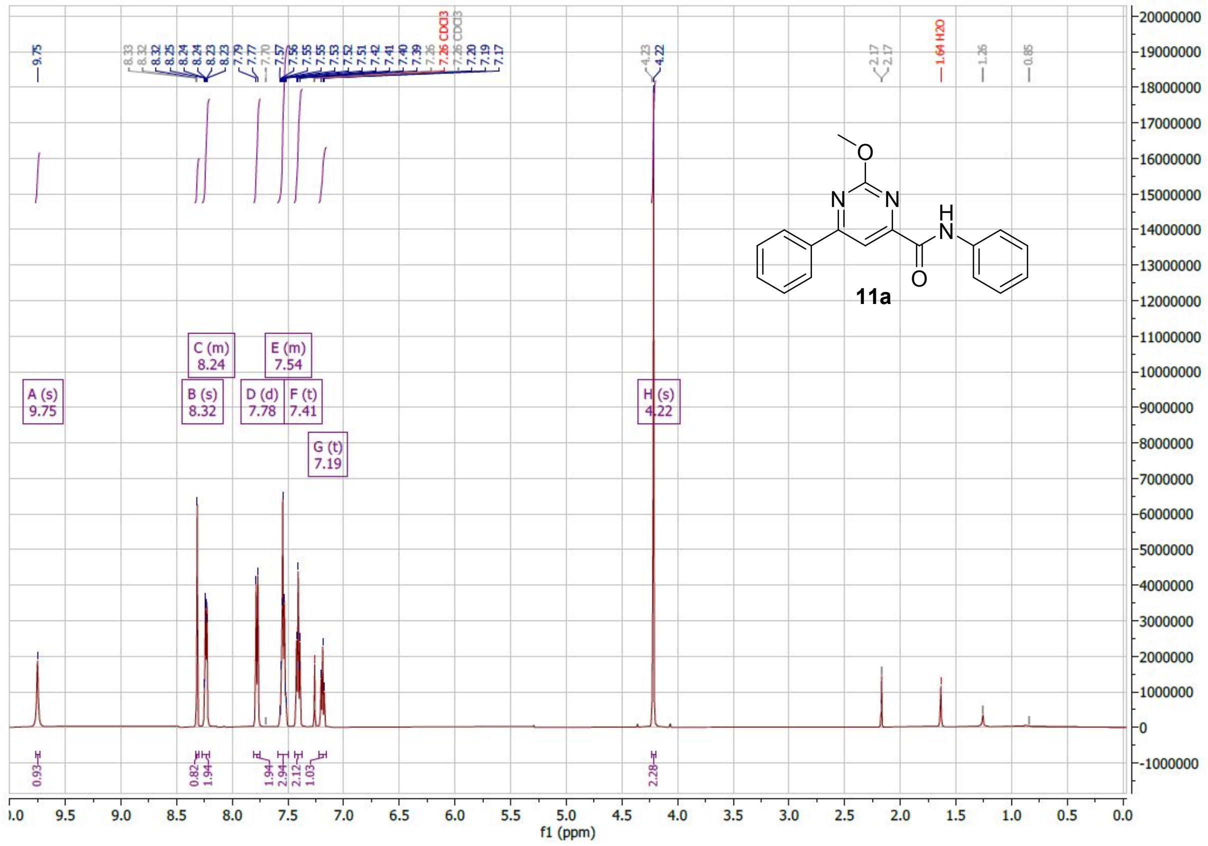

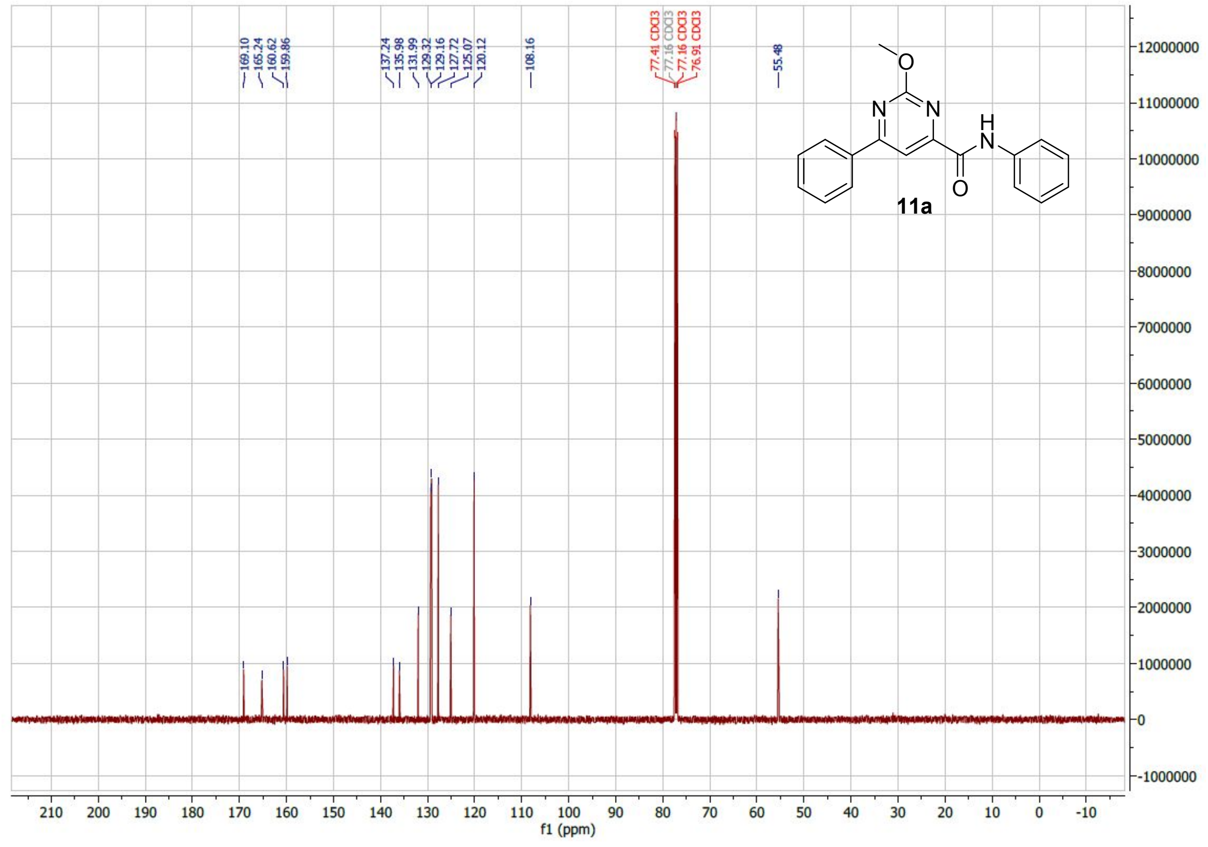

^{1}H and ^{13}C NMR spectra of *N*-(3,4-Dimethoxyphenyl)-2-methoxy-6-(4-methoxyphenyl)pyrimidine-4-carboxamide (11b)

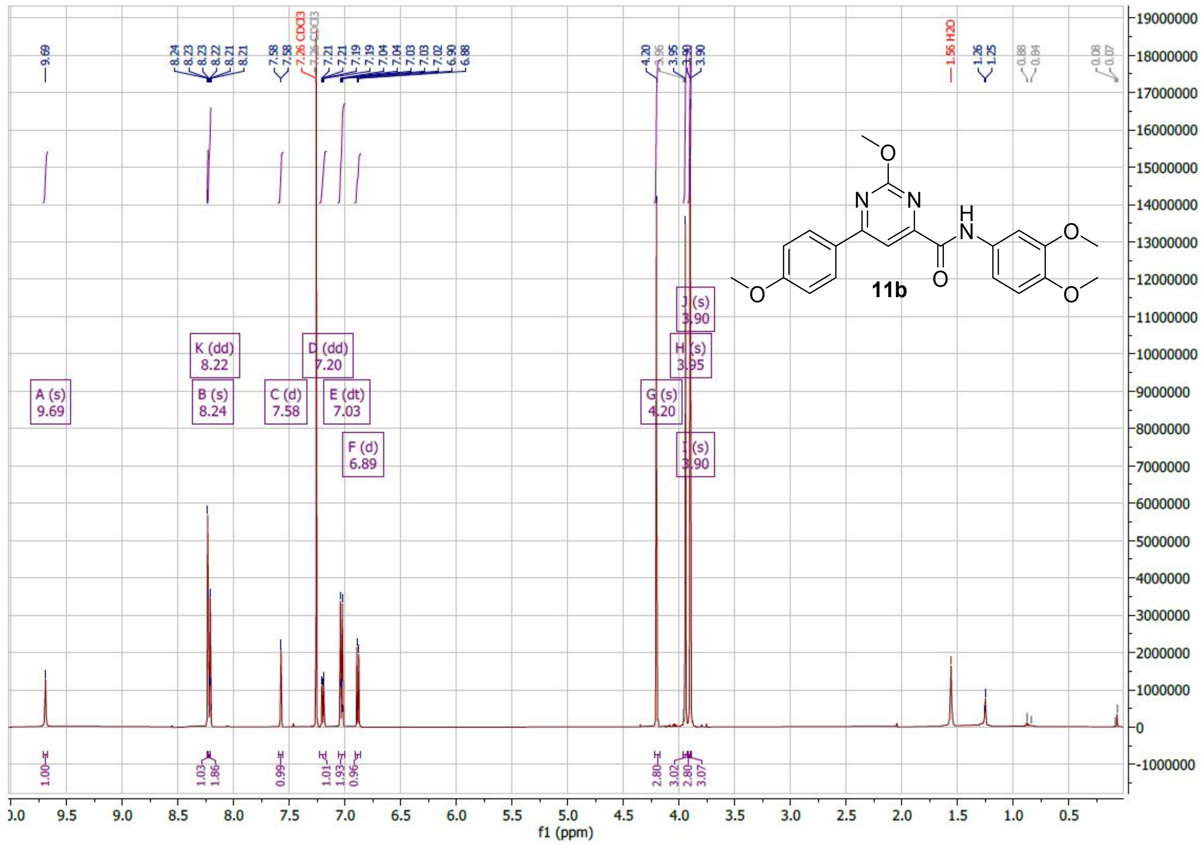

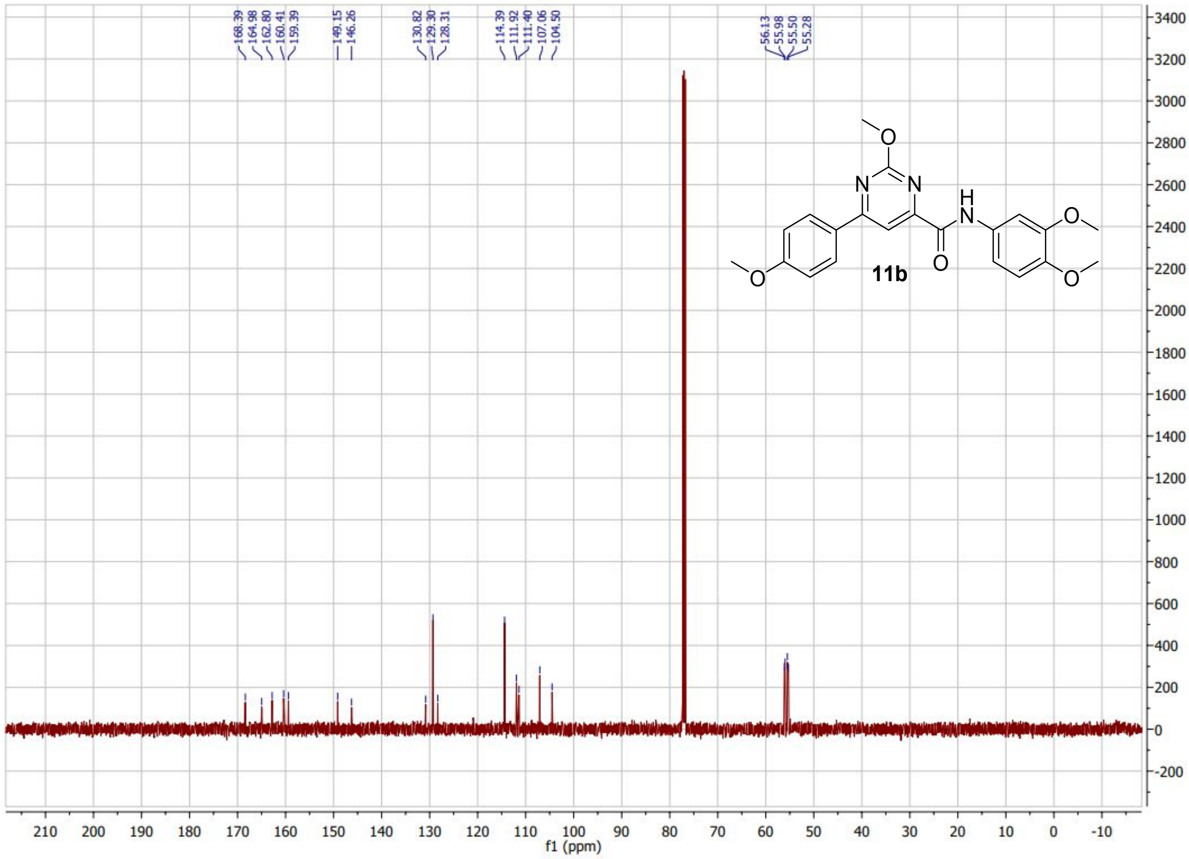

^{1}H and ^{13}C NMR spectra of 2-Oxo-*N*,6-diphenyl-2,3-dihydropyrimidine-4-carboxamide (12a)

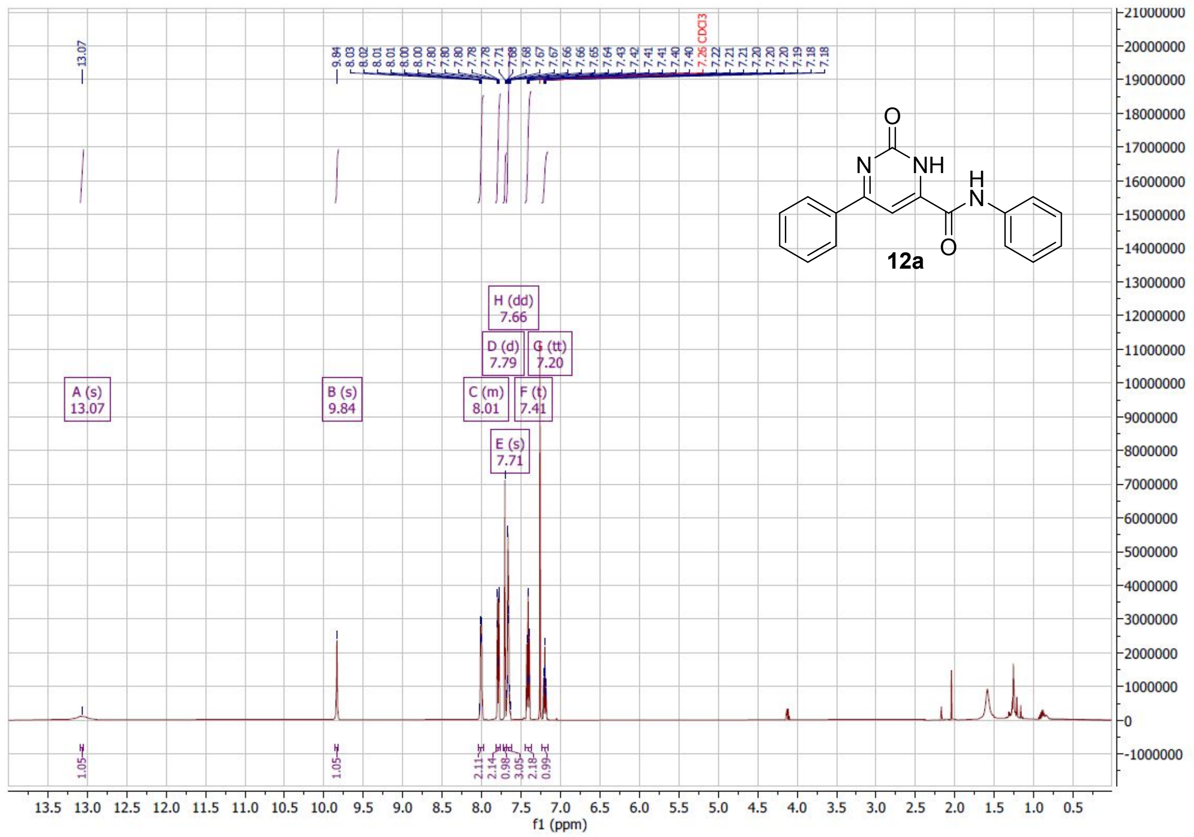

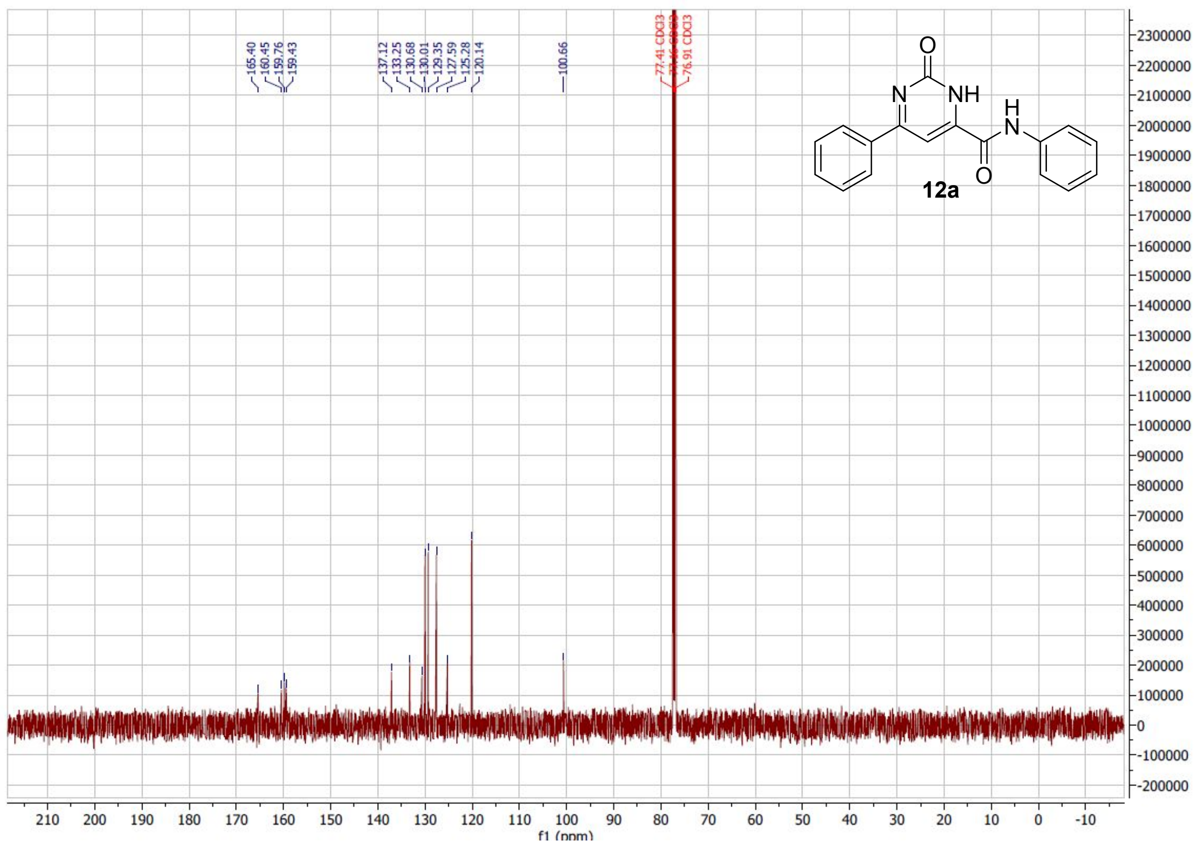

^{1}H and ^{13}C NMR spectra of *N*-(3,4-dimethoxyphenyl)-6-(4-methoxyphenyl)-2-oxo-2,3-dihydropyrimidine-4-carboxamide (12b)

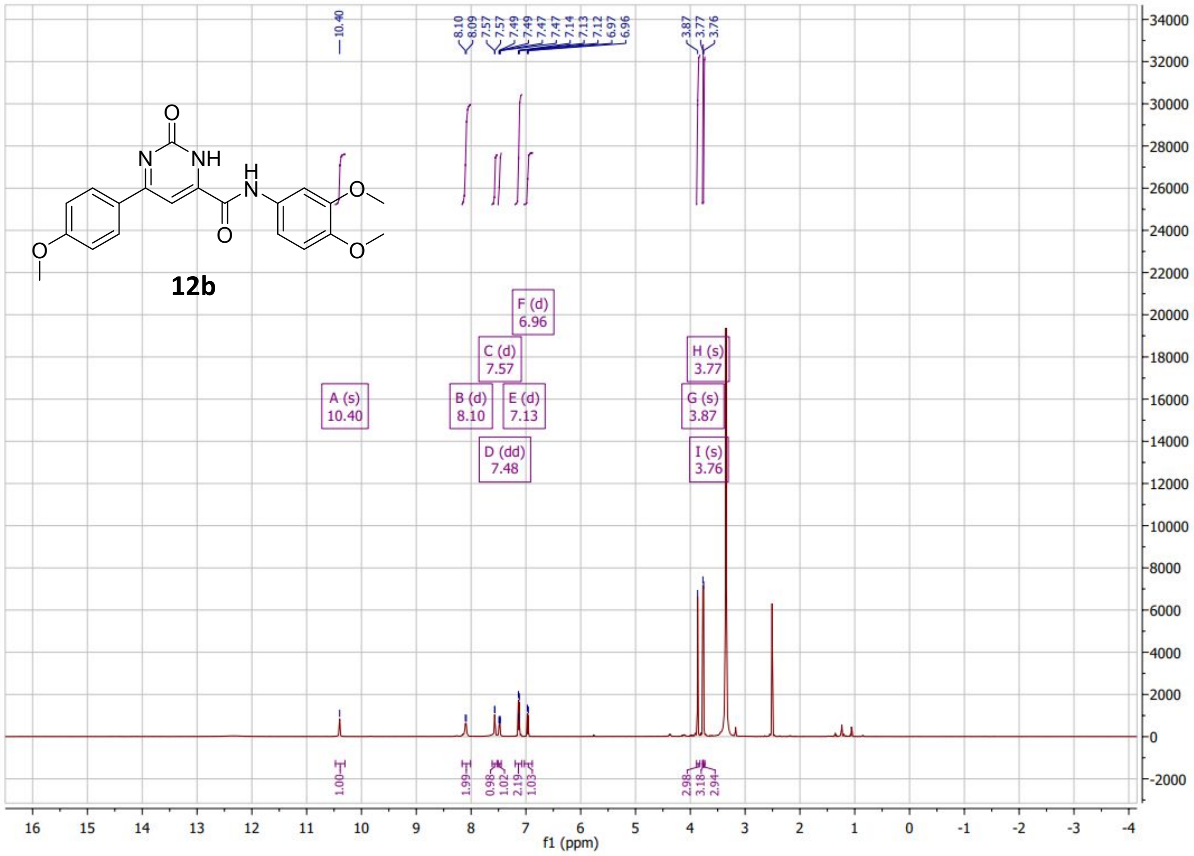

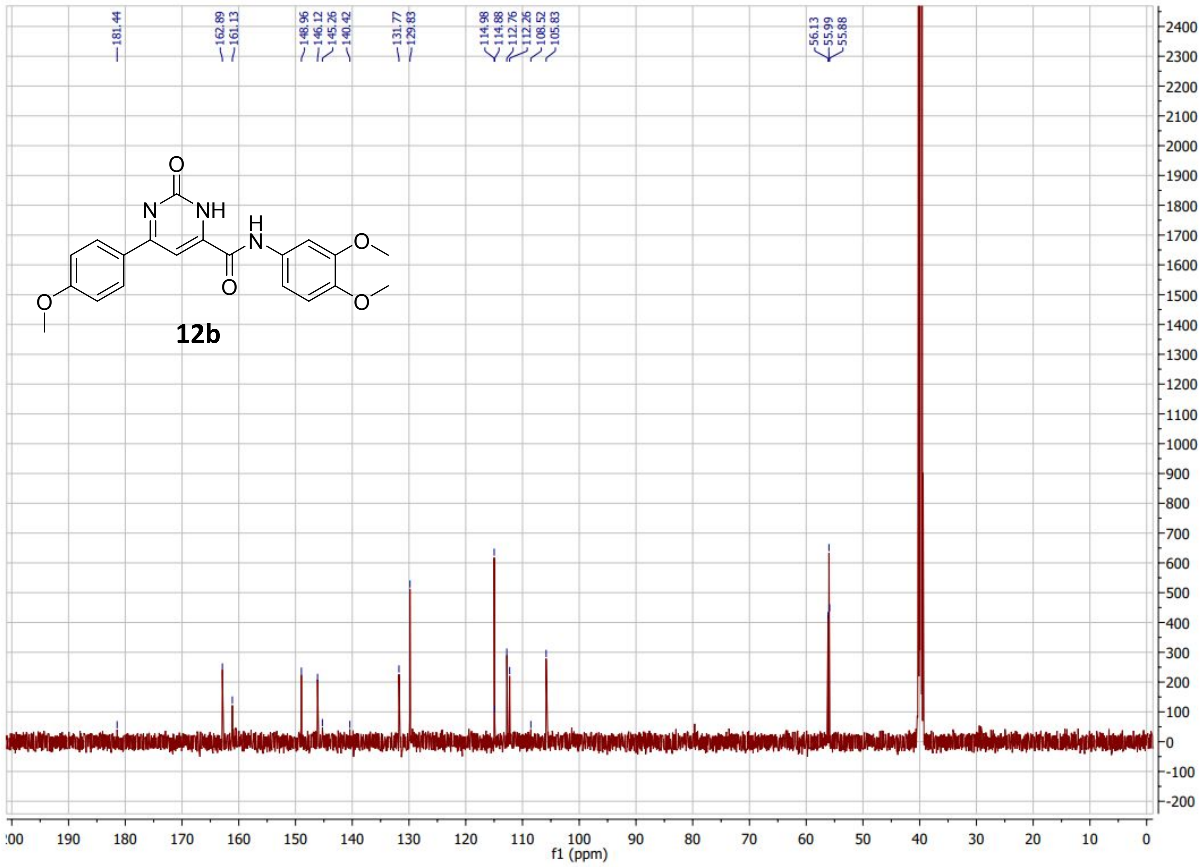

¹H and ¹³C NMR spectra of 2-(4-Methoxybenzyl)isothiouronium chloride (13')

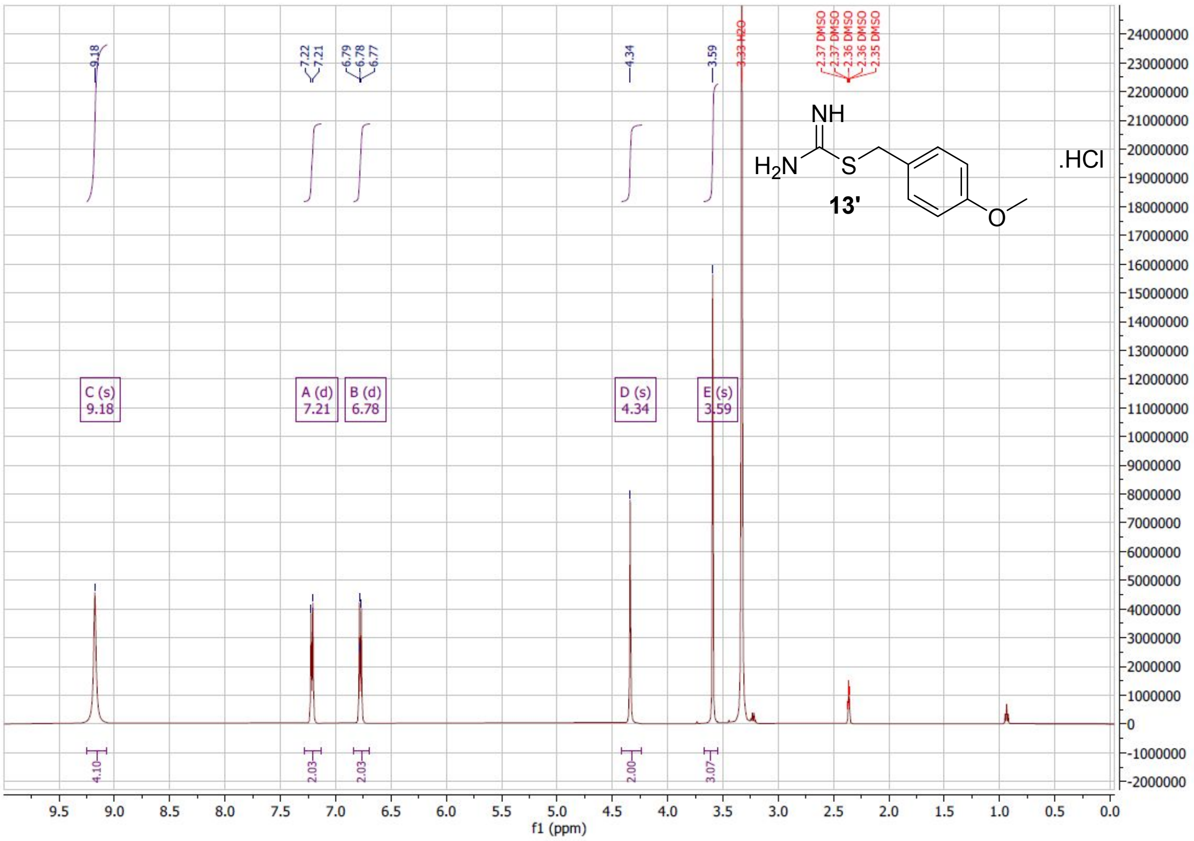

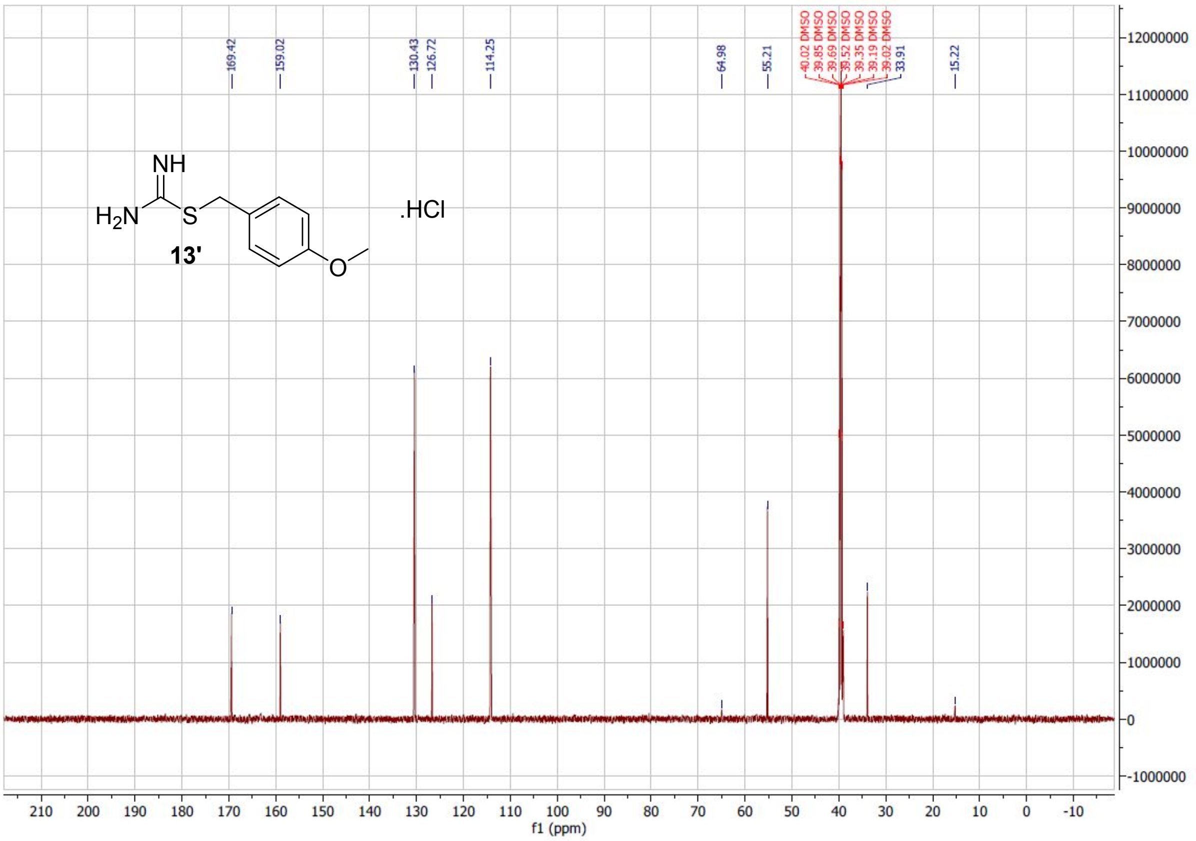

^{1}H and ^{13}C NMR spectra of 2-Methoxy-*N*,6-diphenylpyrimidine-4-carboxamide (13a)

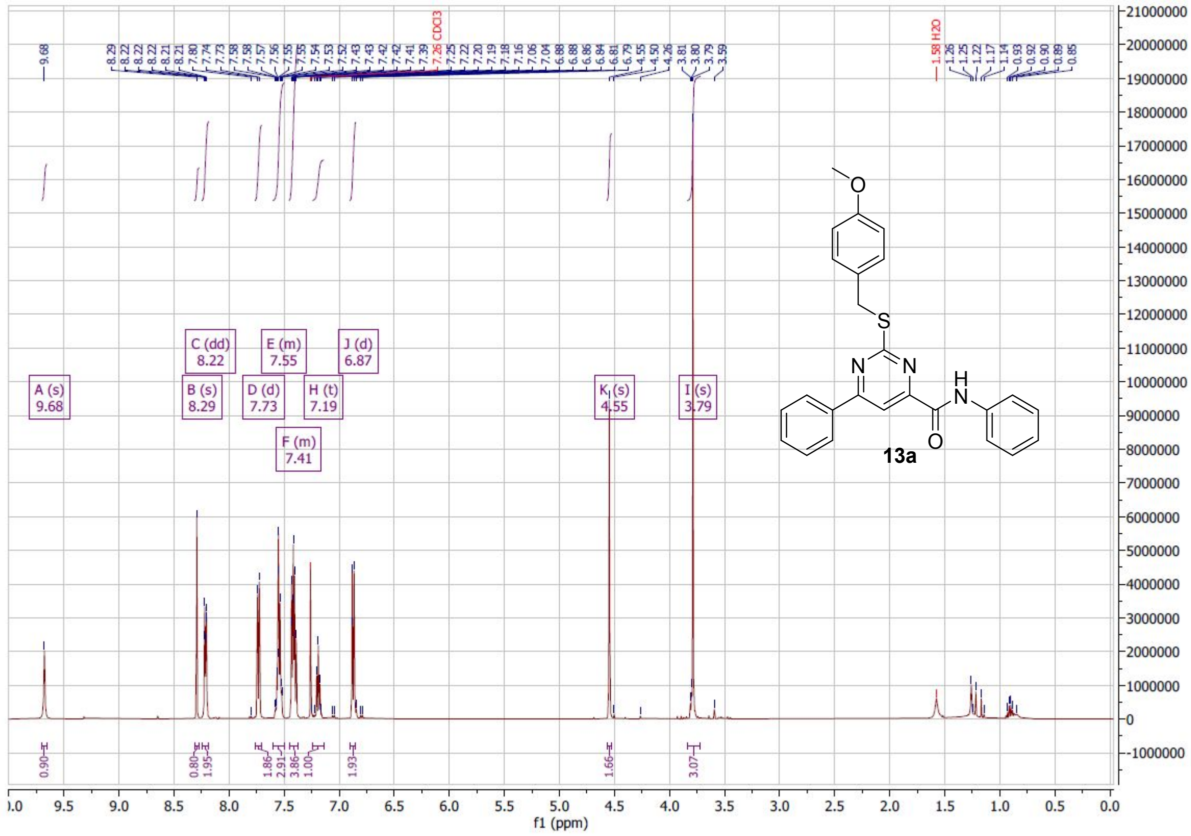

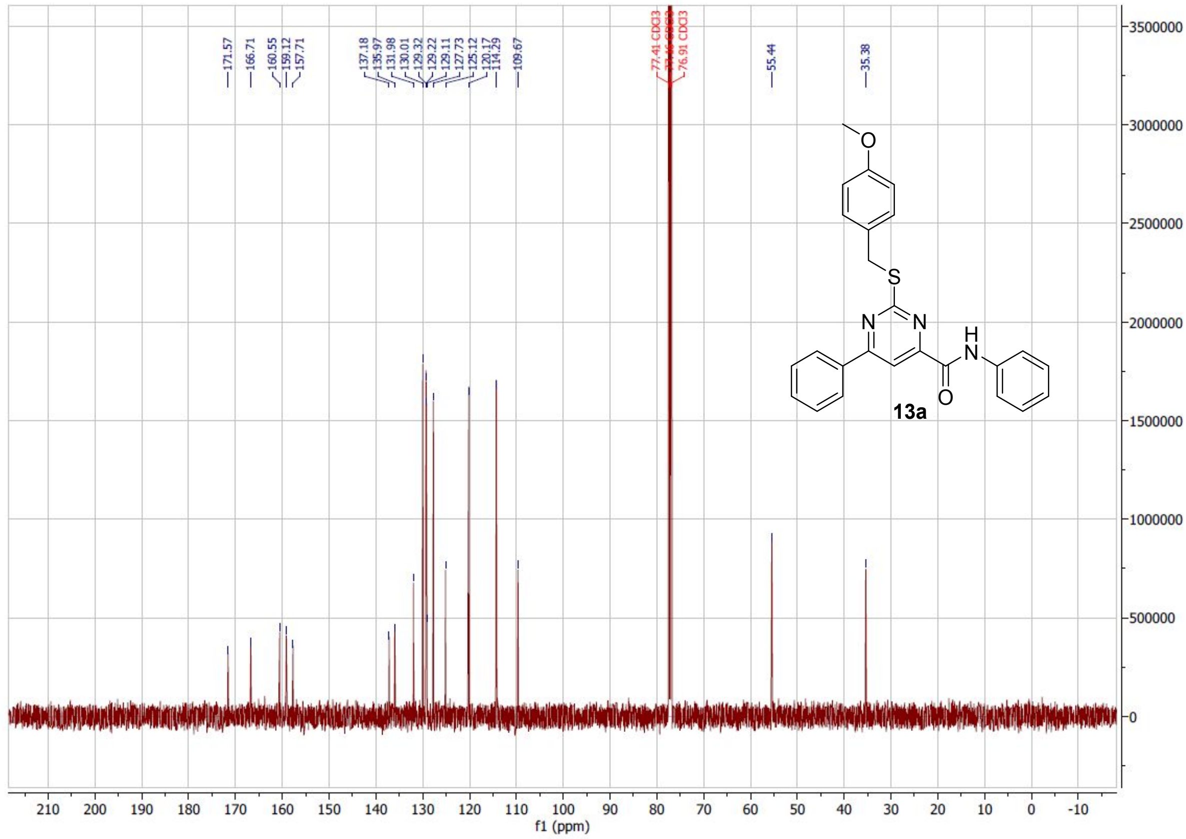

^{1}H and ^{13}C NMR spectra of 2-Amino-*N*,6-diphenylpyrimidine-4-carboxamide (14a)

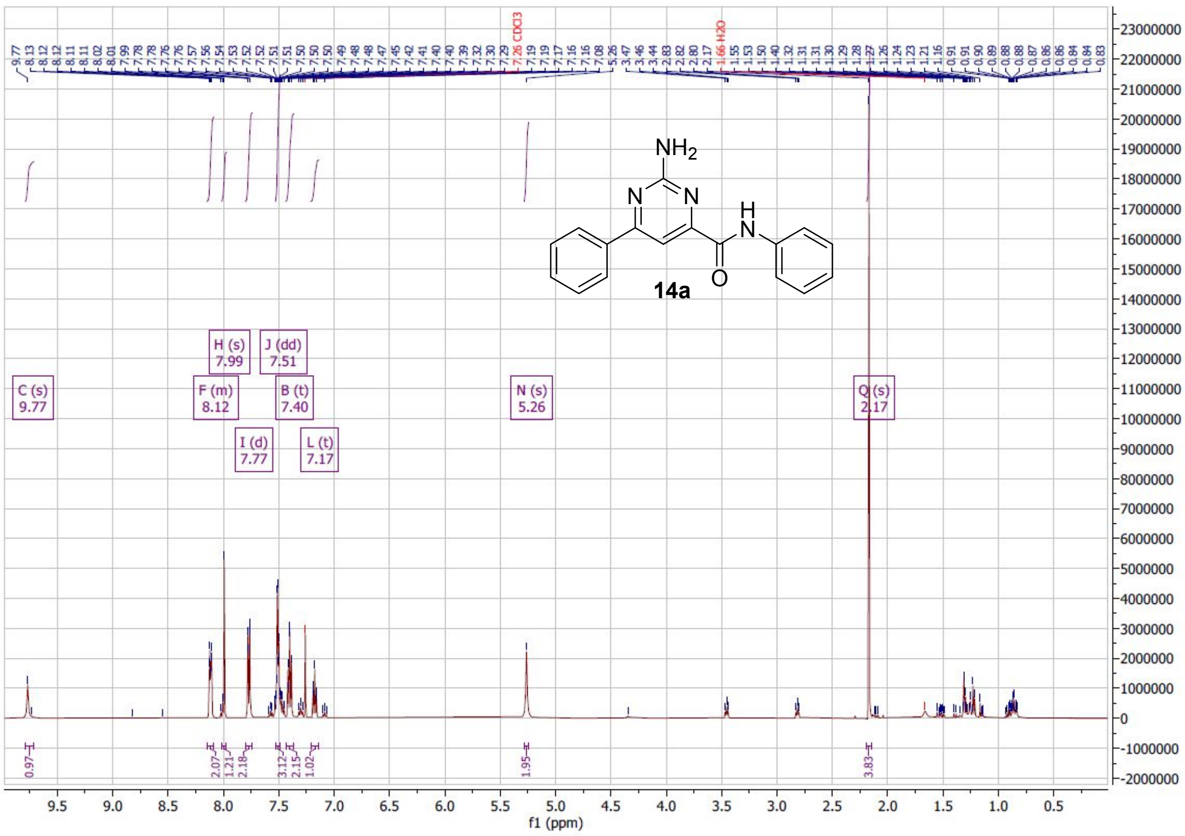

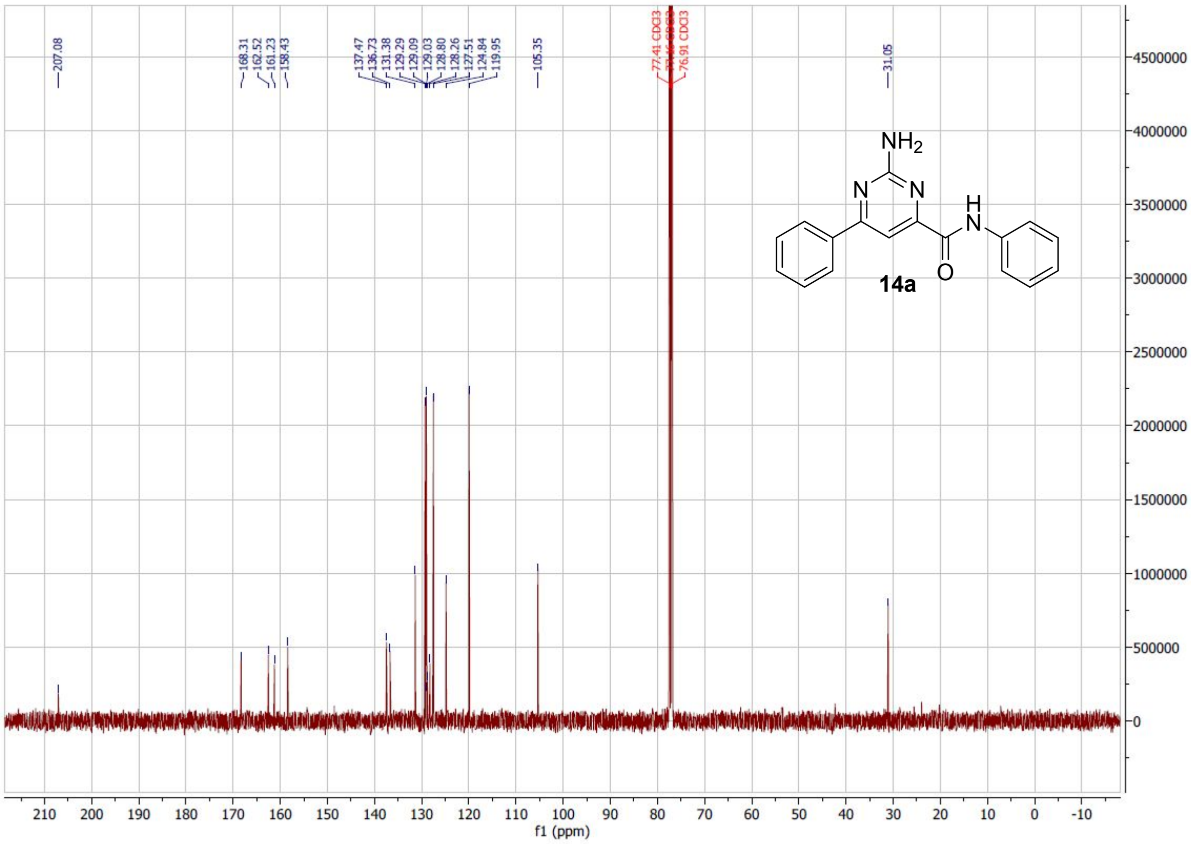

^{1}H and ^{13}C NMR spectra of 2-Amino-*N*-(3,4-dimethoxyphenyl)-6-(4-methoxyphenyl)pyrimidine-4-carboxamide (14b)

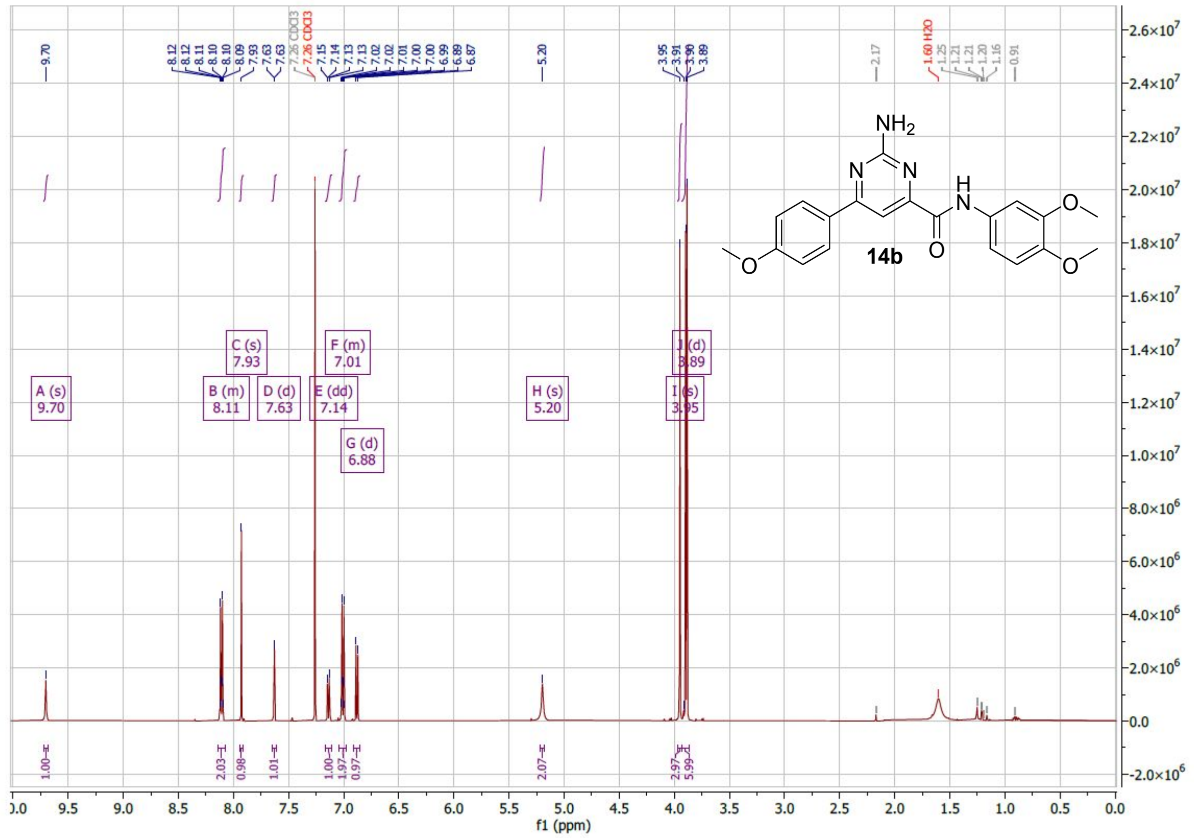

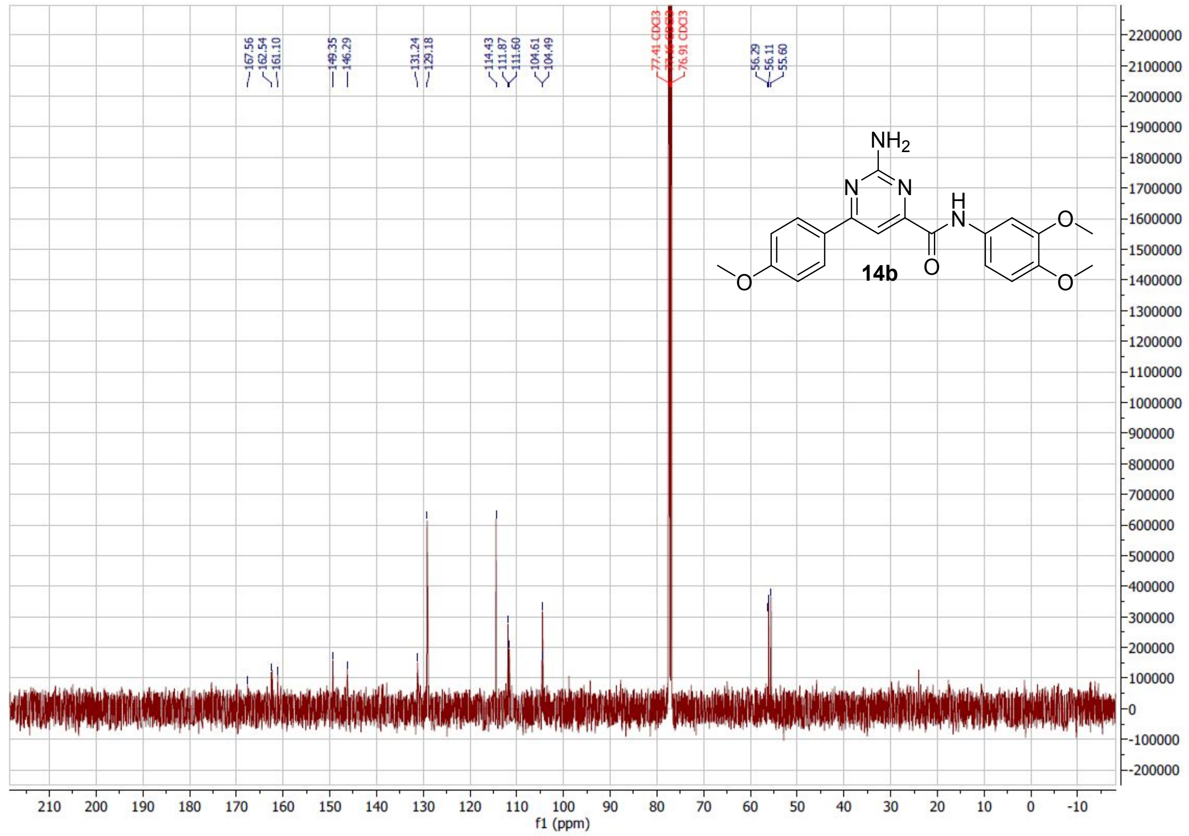

^{1}H and ^{13}C NMR spectra of N-acetonylpyridinium chloride (15')

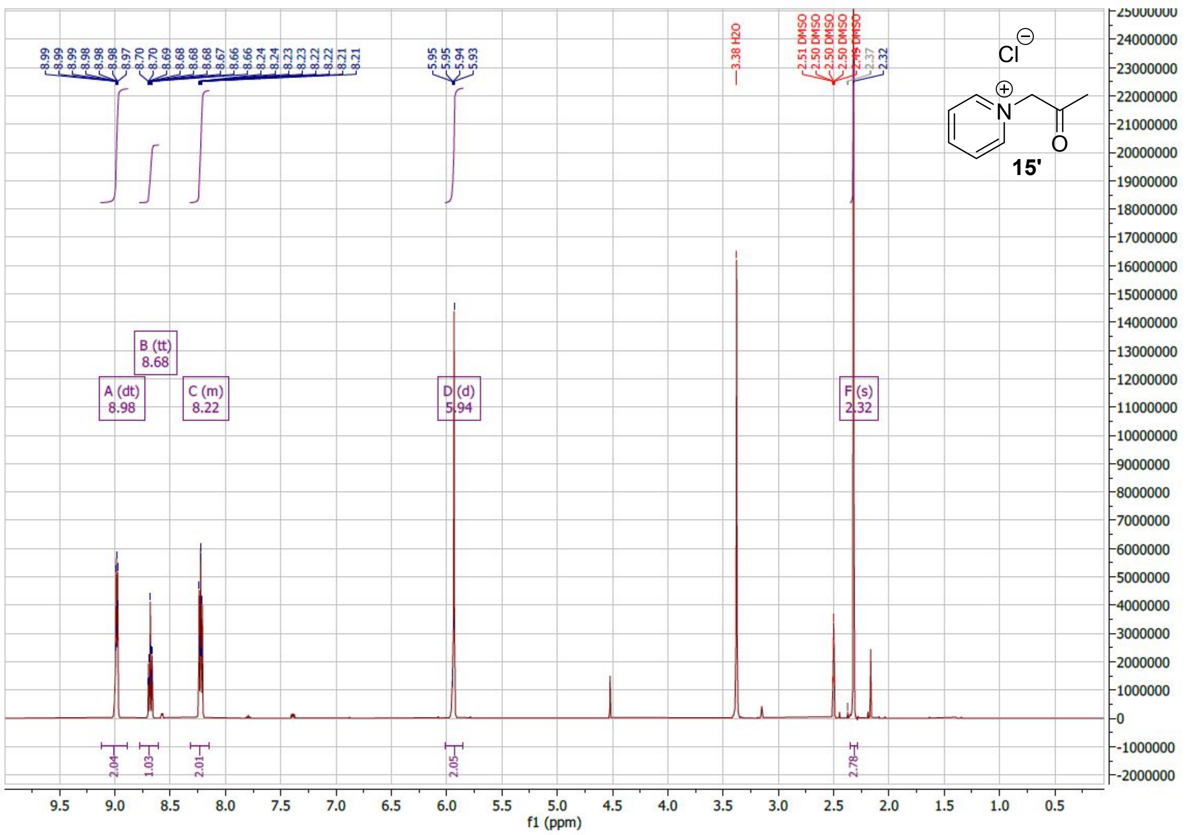

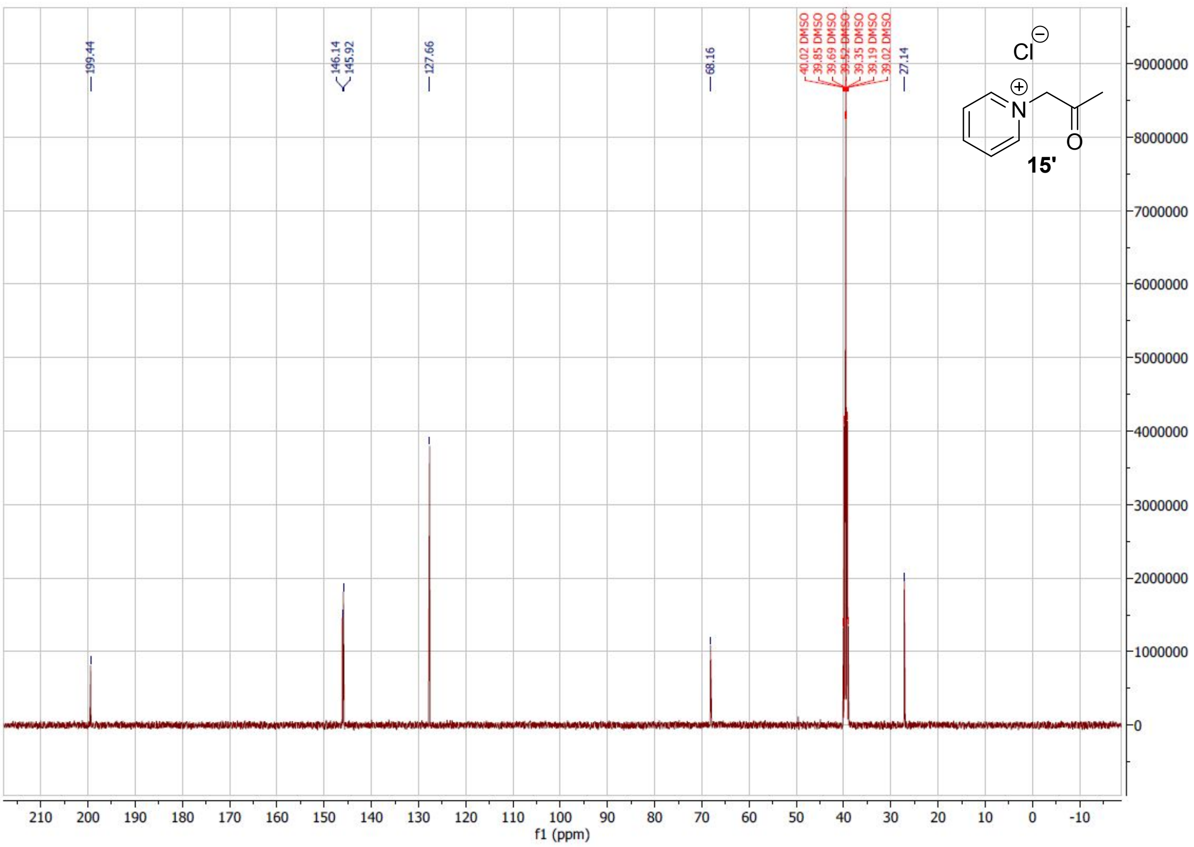

^{1}H and ^{13}C NMR spectra of 2-Methyl-*N*,6-diphenylisonicotinamide (15a)

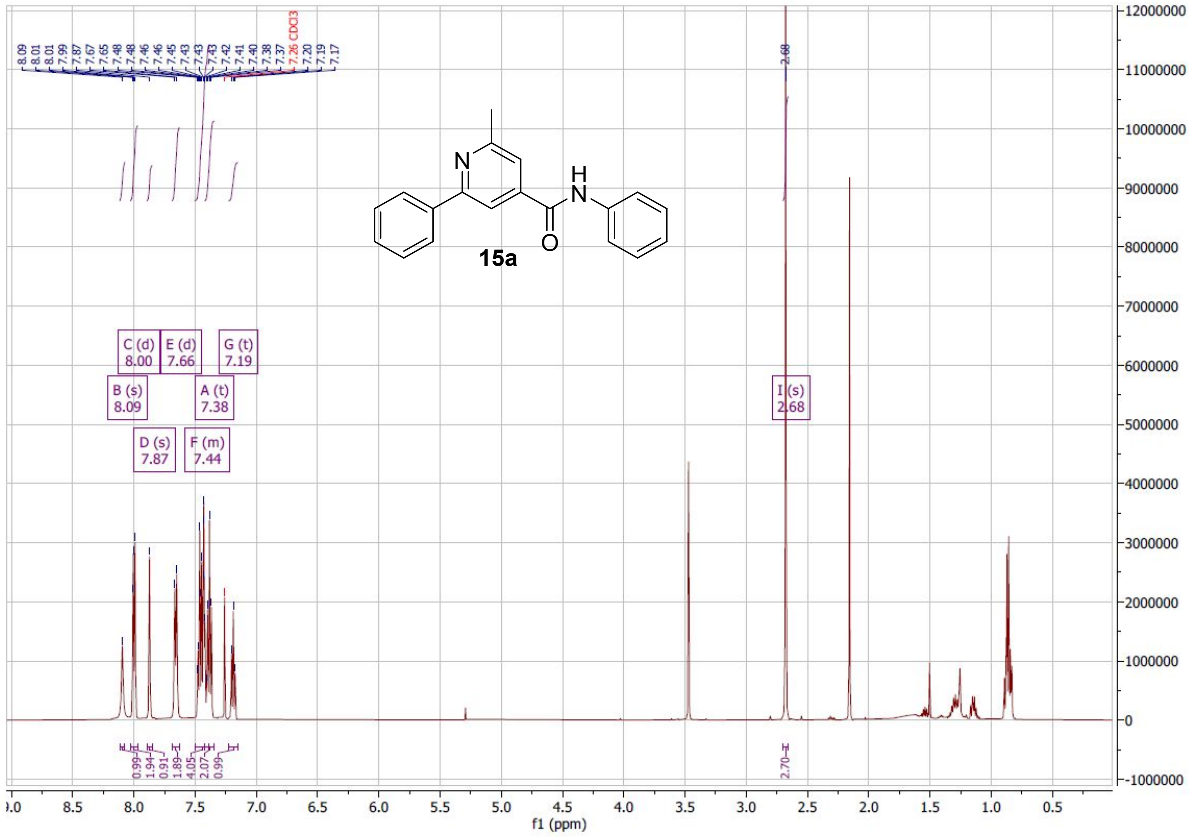

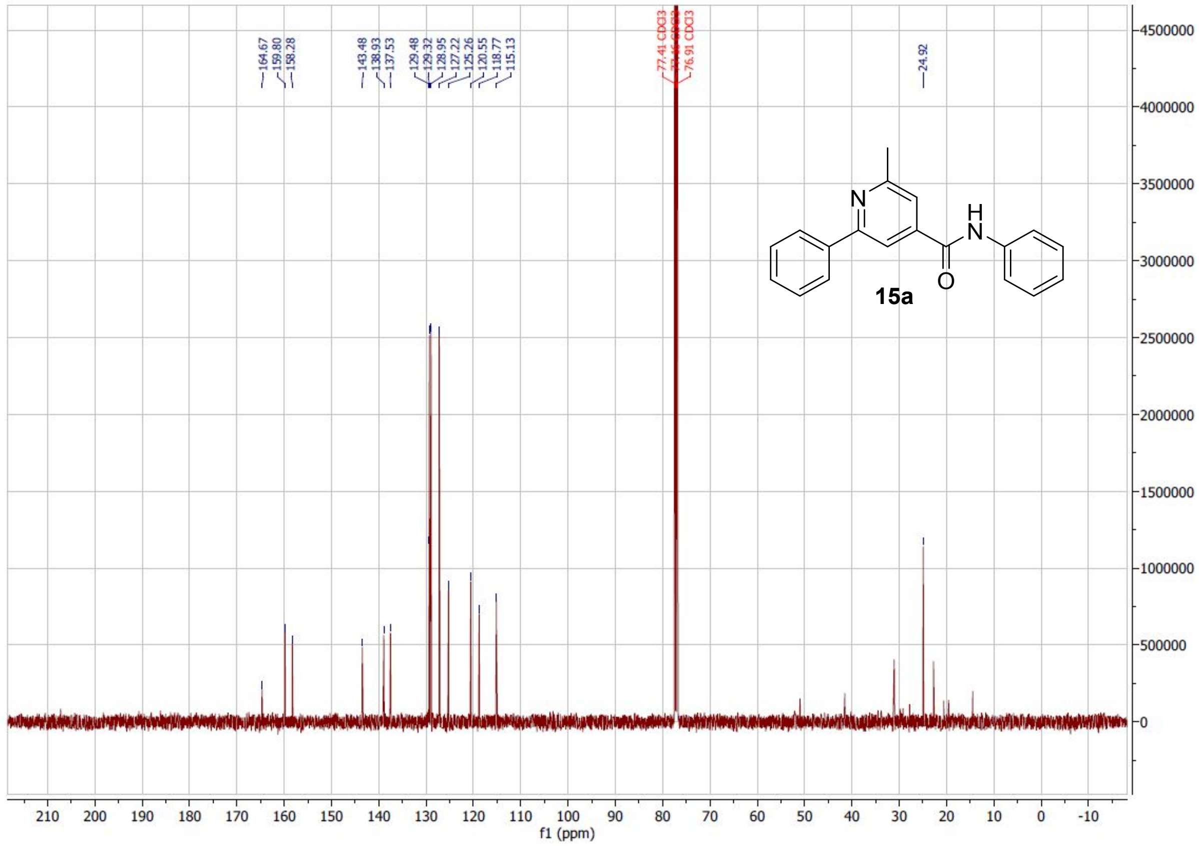

^{1}H and ^{13}C NMR spectra of *N*-(3,4-Dimethoxyphenyl)-2-(4-methoxyphenyl)-6-methylisonicotinamide (15b)

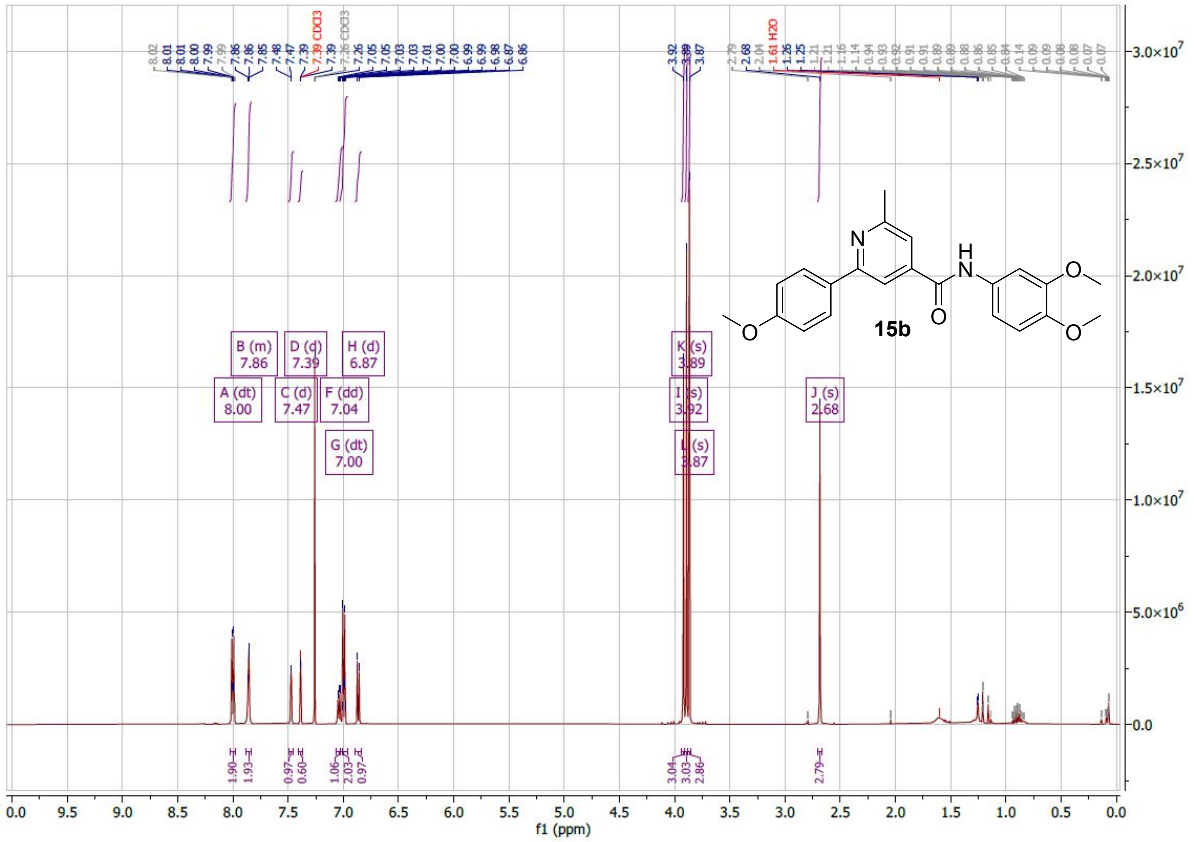

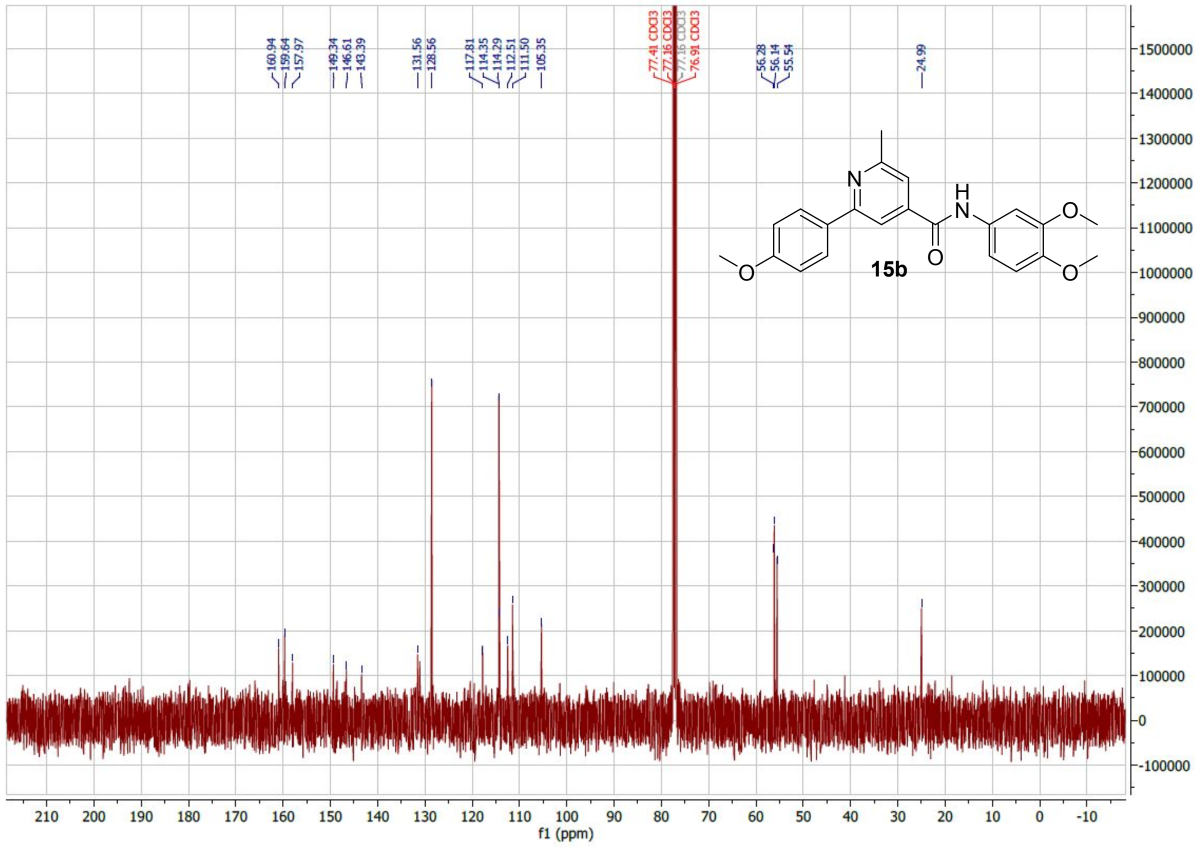

2.5.4. Transformations: 3,4-Cyclisations

^{1}H and ^{13}C NMR spectra of *trans*-2-Benzoyl-*N*-phenylcyclopropane-1-carboxamide (16a)

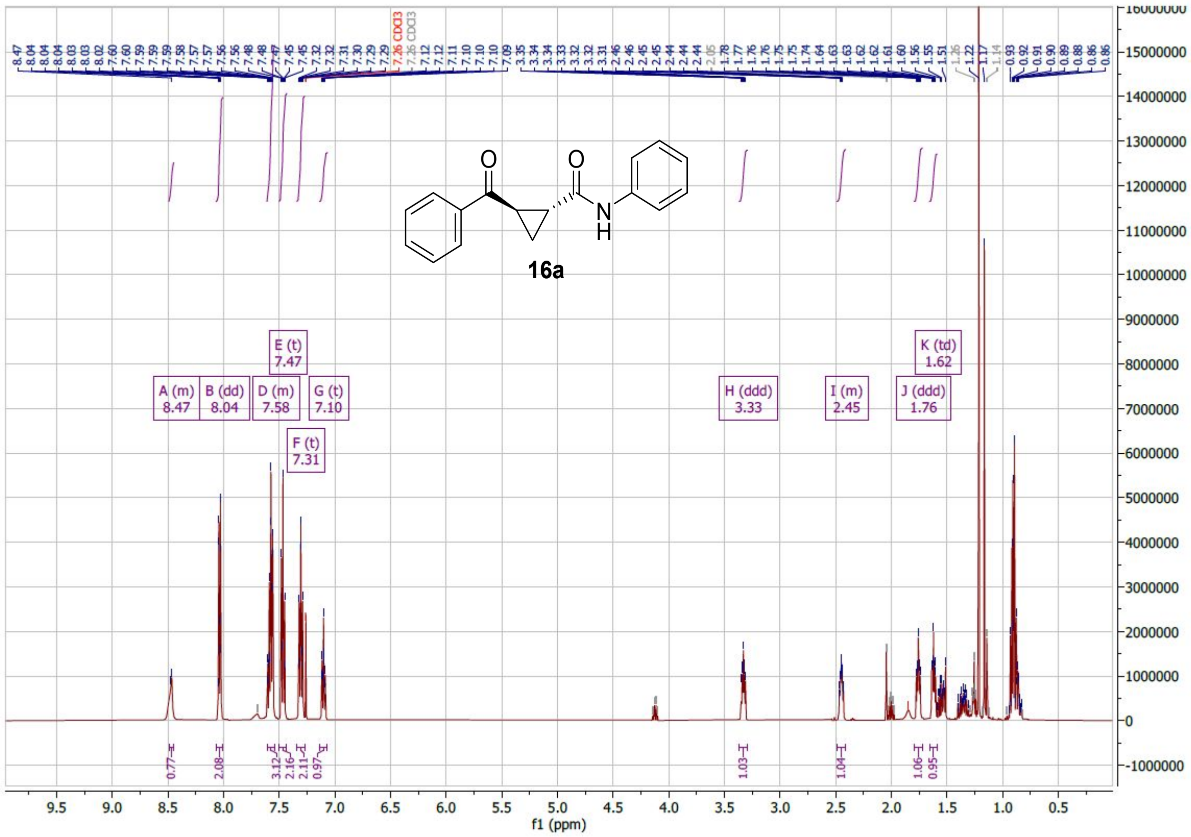

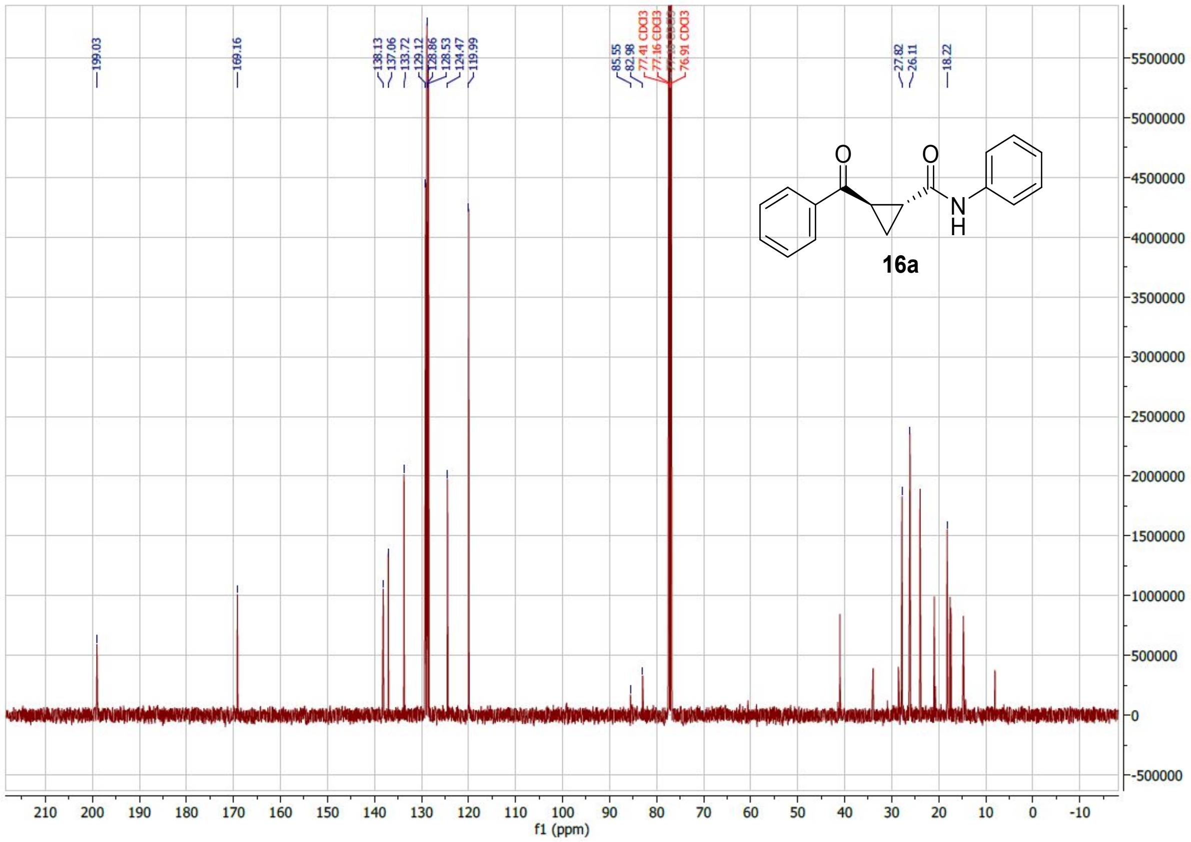

^{1}H and ^{13}C NMR spectra of *trans*-*N*-(3,4-Dimethoxyphenyl)-2-(4-methoxybenzoyl)cyclopropane-1-carboxamide (16b)

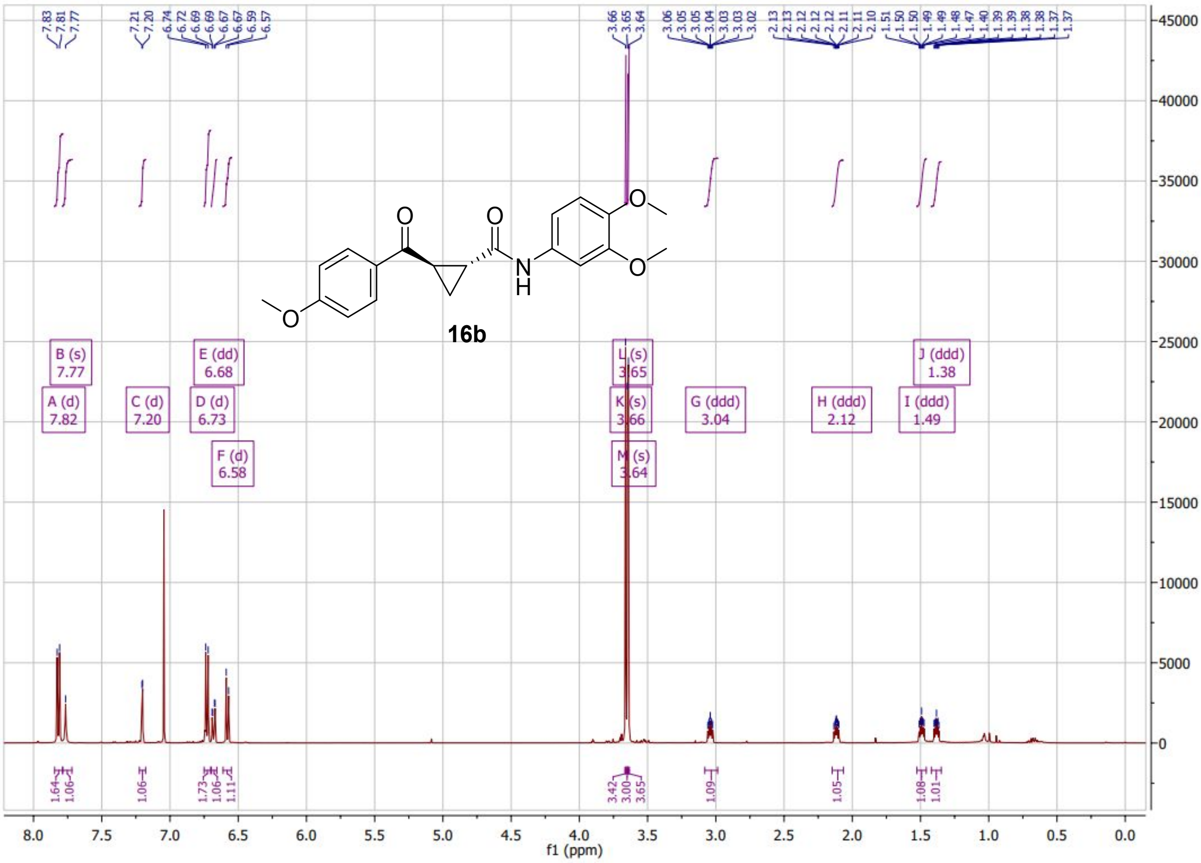

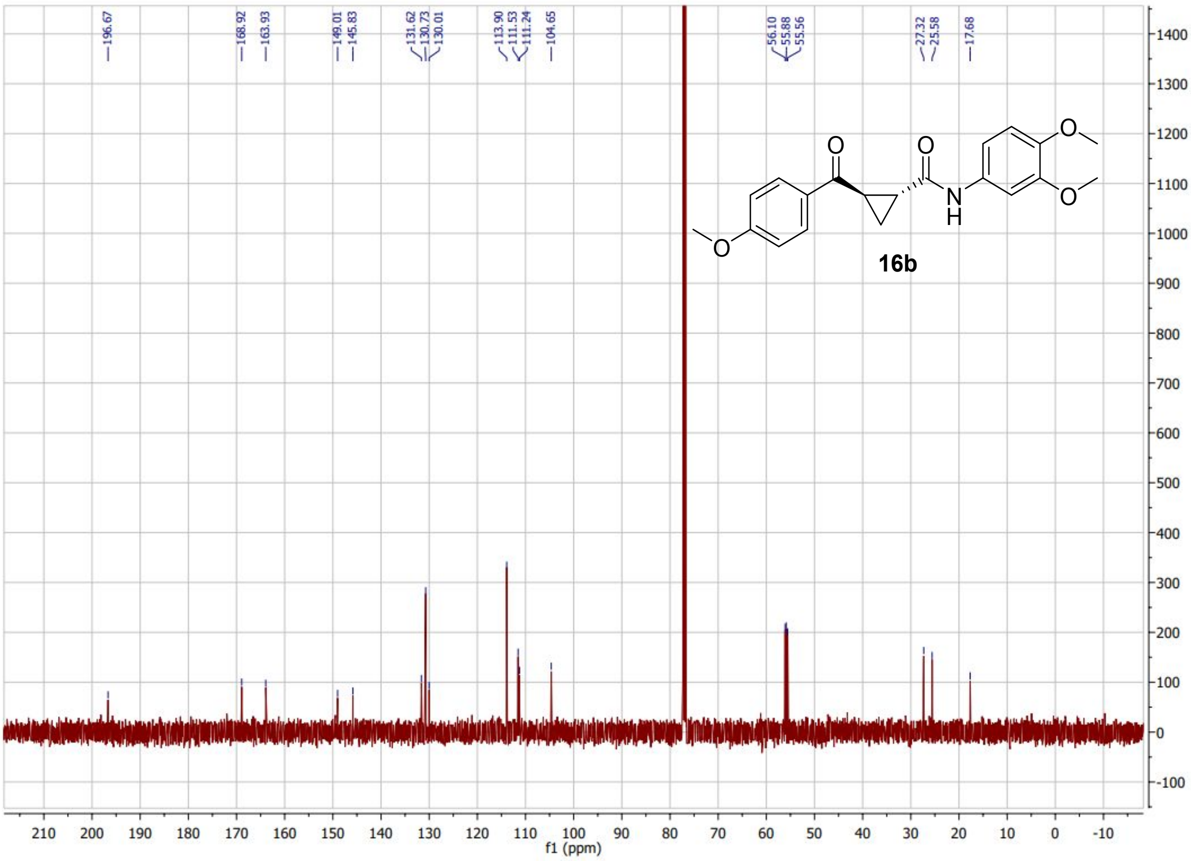

¹H and ¹³C NMR spectra of (*rac*)-*trans*-3-Benzoyl-*N*-phenylbicyclo[2.2.1]hept-5-ene-2-carboxamide (17a)

Diastereoisomer 1

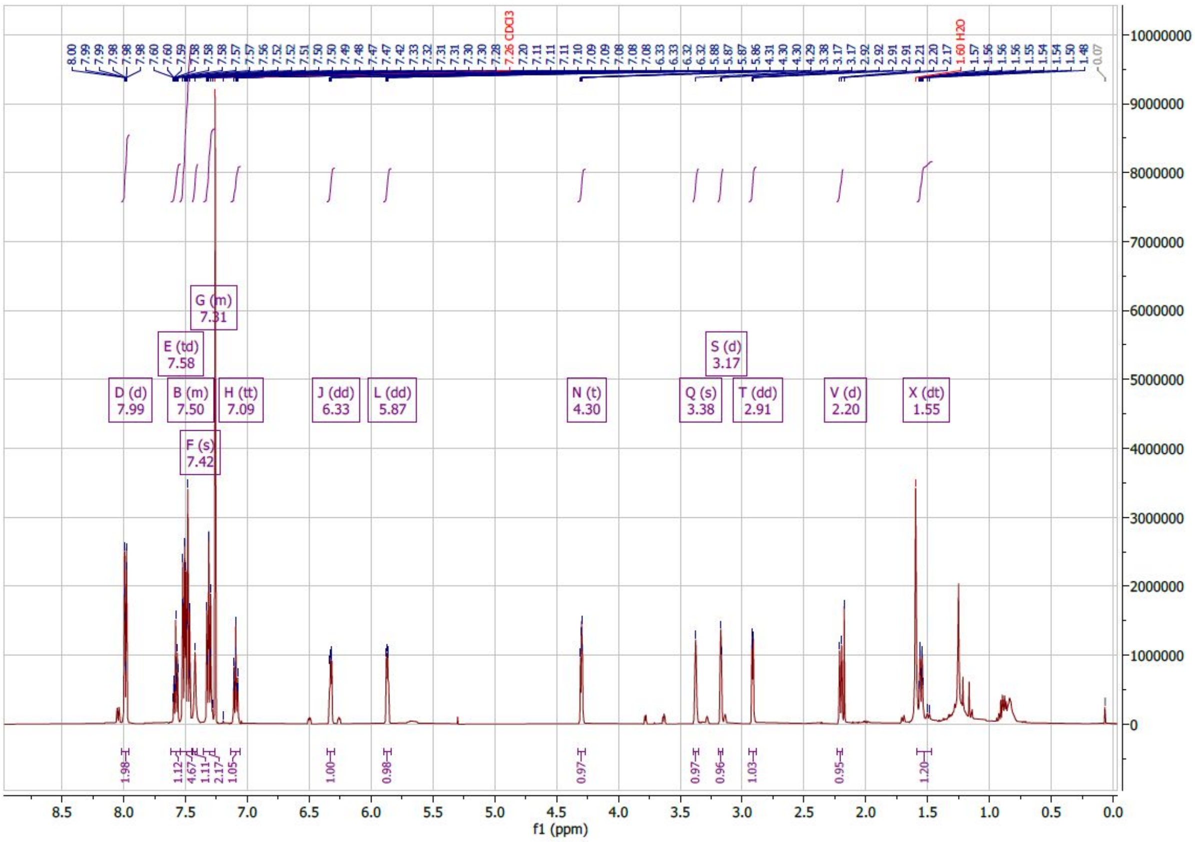

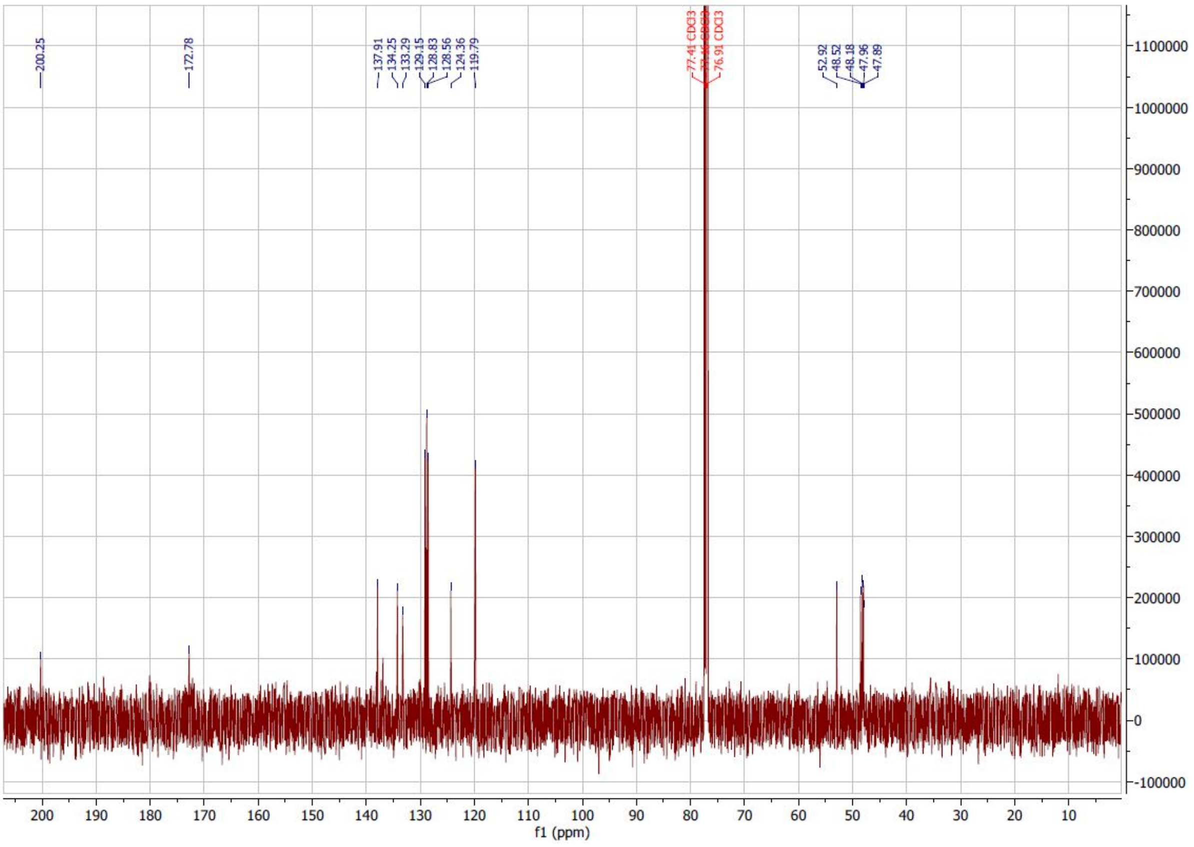

Mixture of diastereoisomers

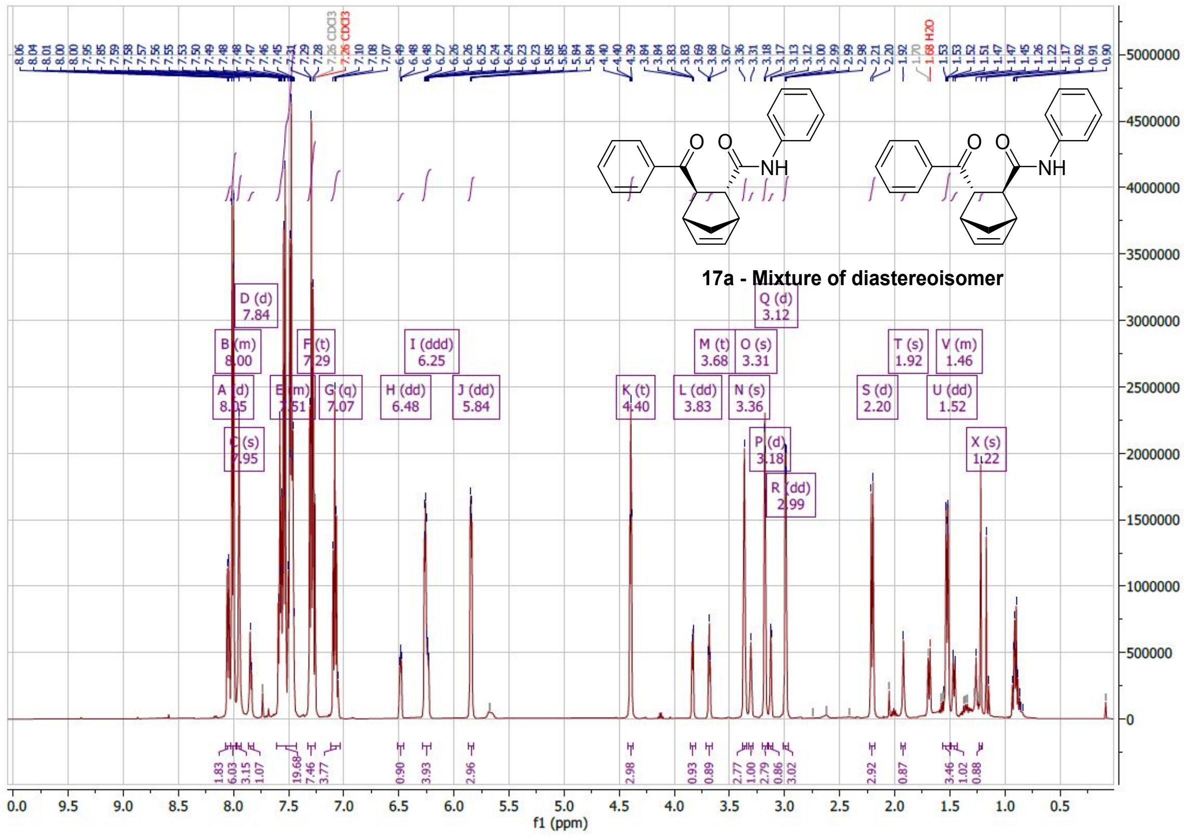

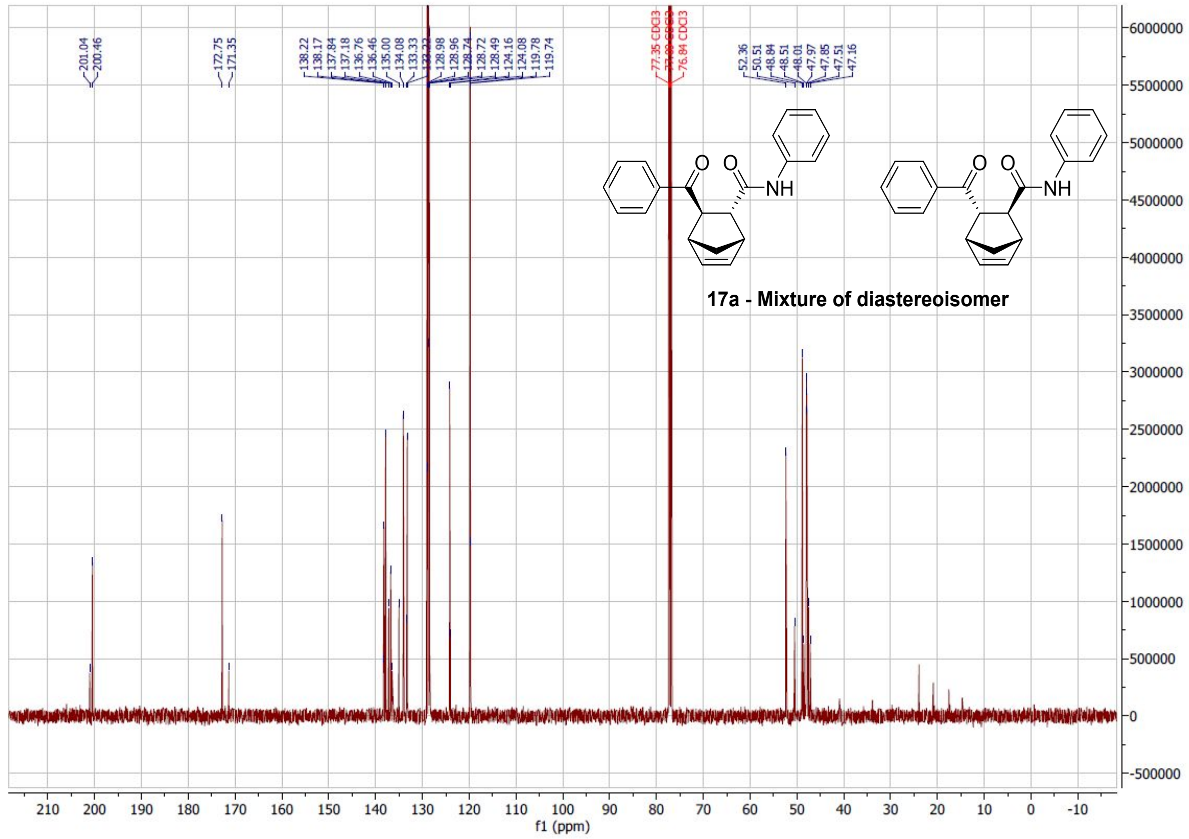

^{1}H and ^{13}C NMR spectra of *N*-(3,4-dimethoxyphenyl)-3-(4-methoxybenzoyl)bicyclo[2.2.1]hept-5-ene-2-carboxamide (17b)

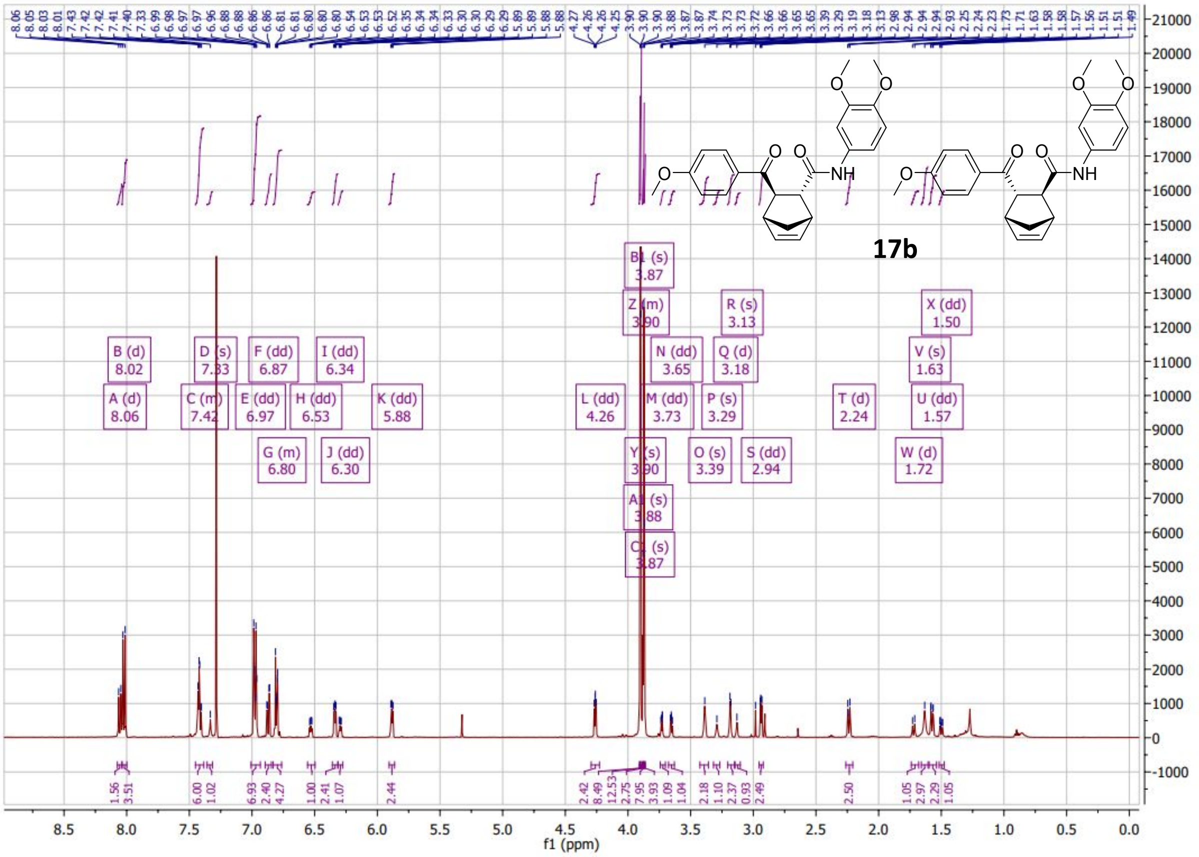

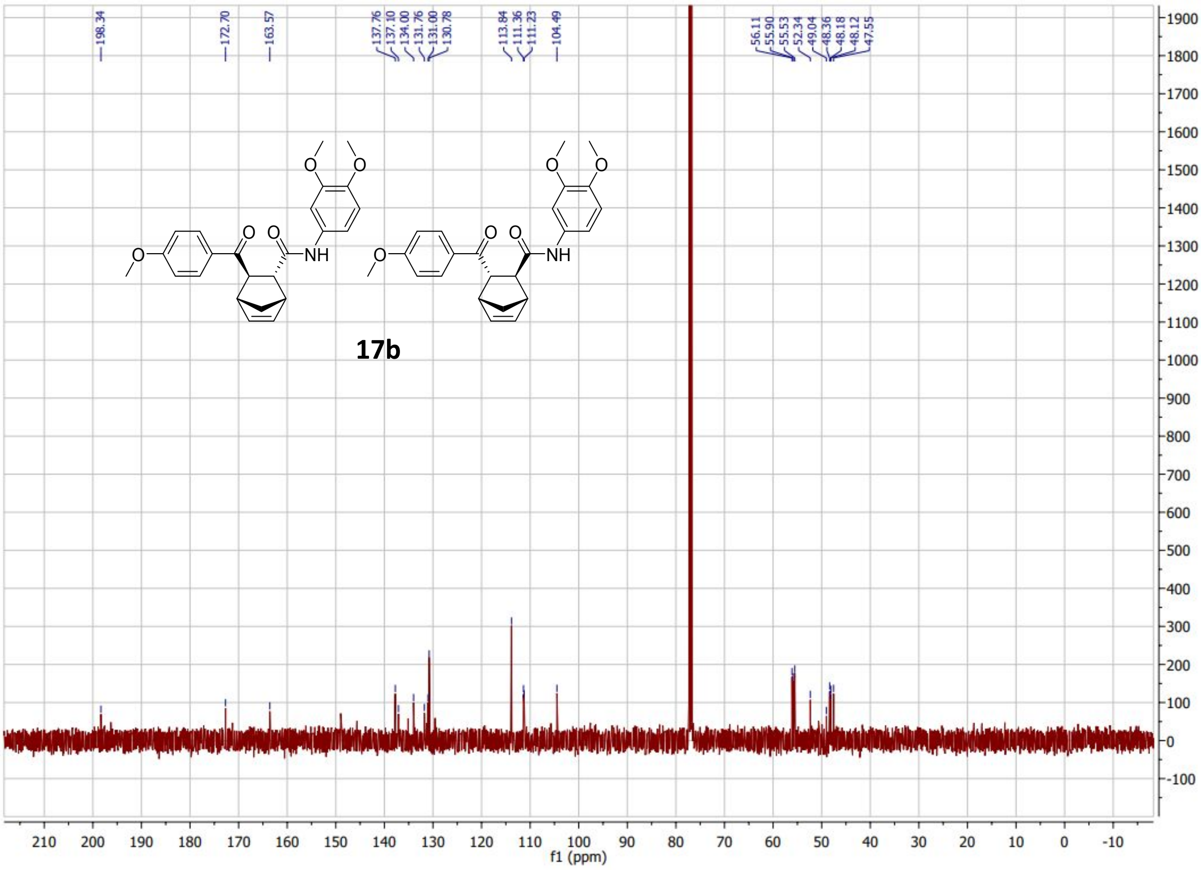

^{1}H and ^{13}C NMR spectra of 2-(3,4-dimethoxyphenyl)-3-(4-methoxyphenyl)-5,6-dimethyl-2,3,4,7-tetrahydro-1*H*-isoindol-1-one (18b)

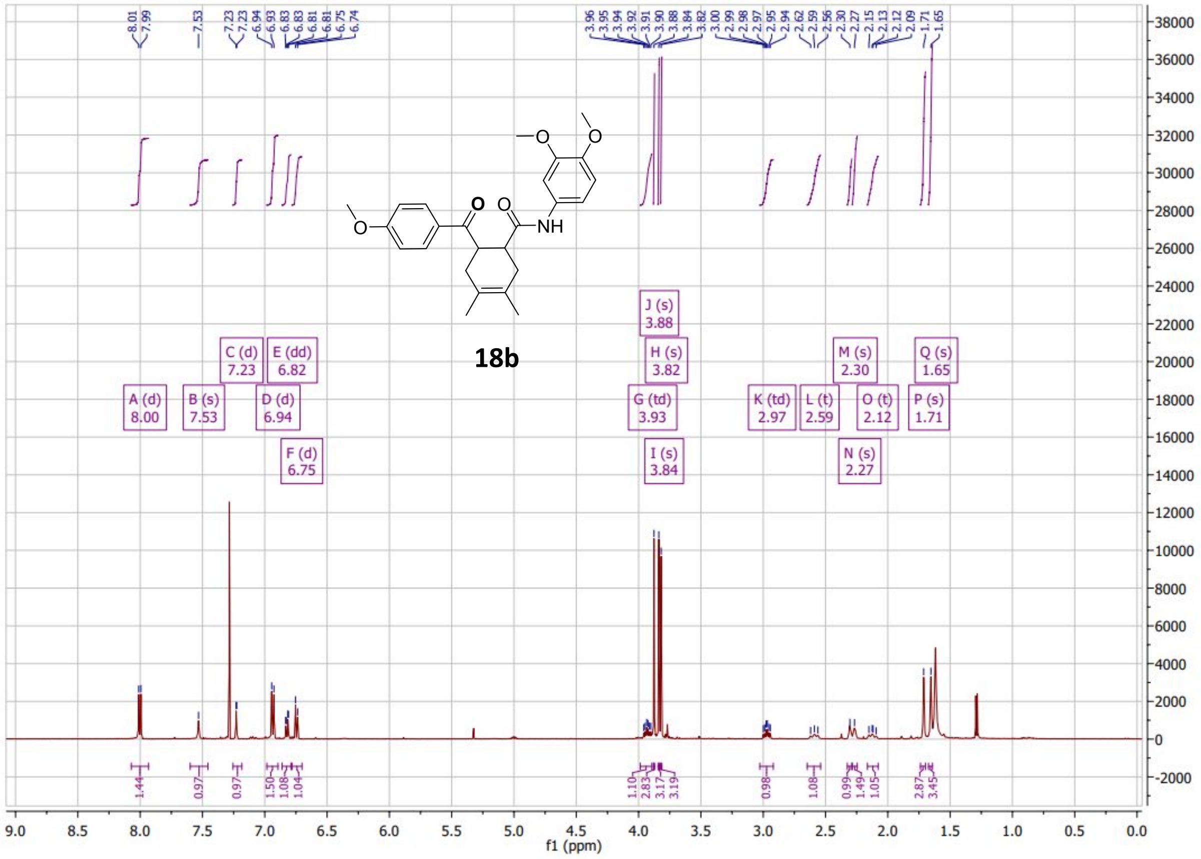

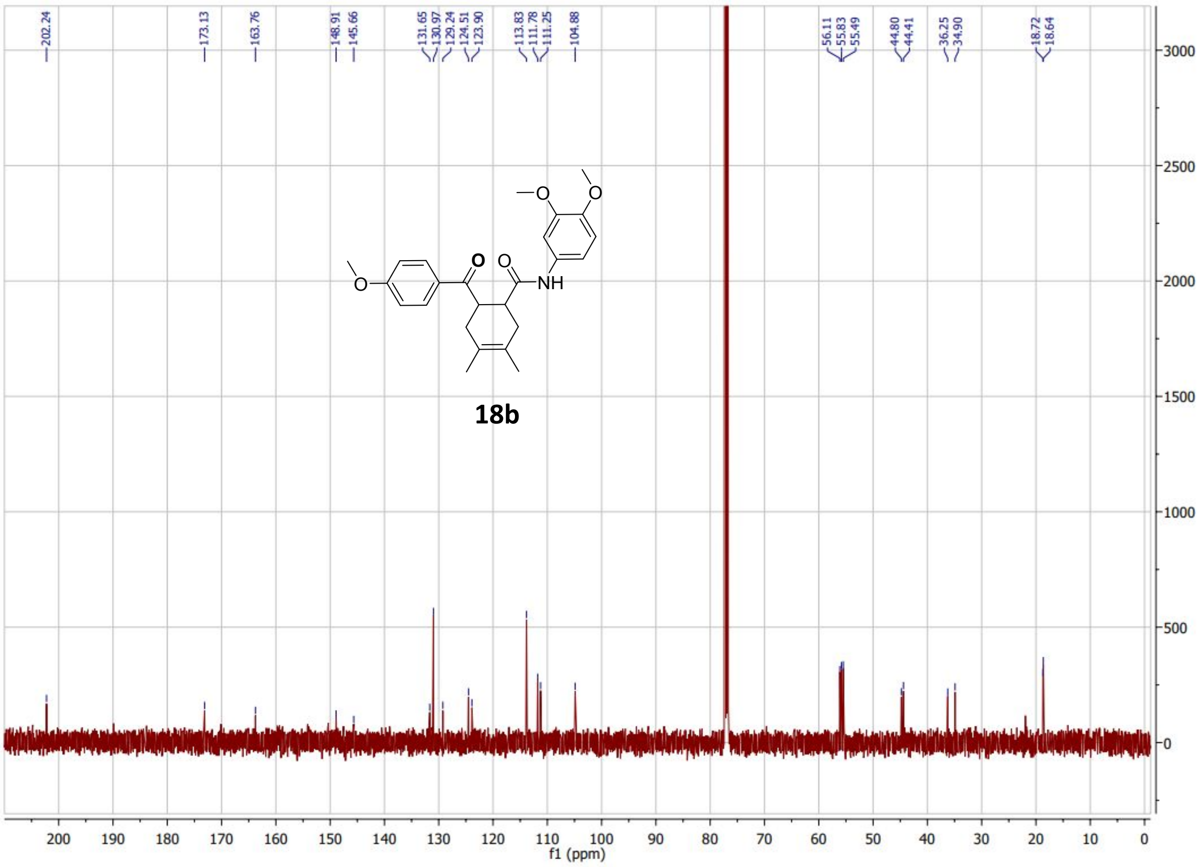

^{1}H and ^{13}C NMR spectra of 5,6-Dimethyl-2,3-diphenyl-2,3,4,7-tetrahydro-1*H*-isoindol-1-one (19a)

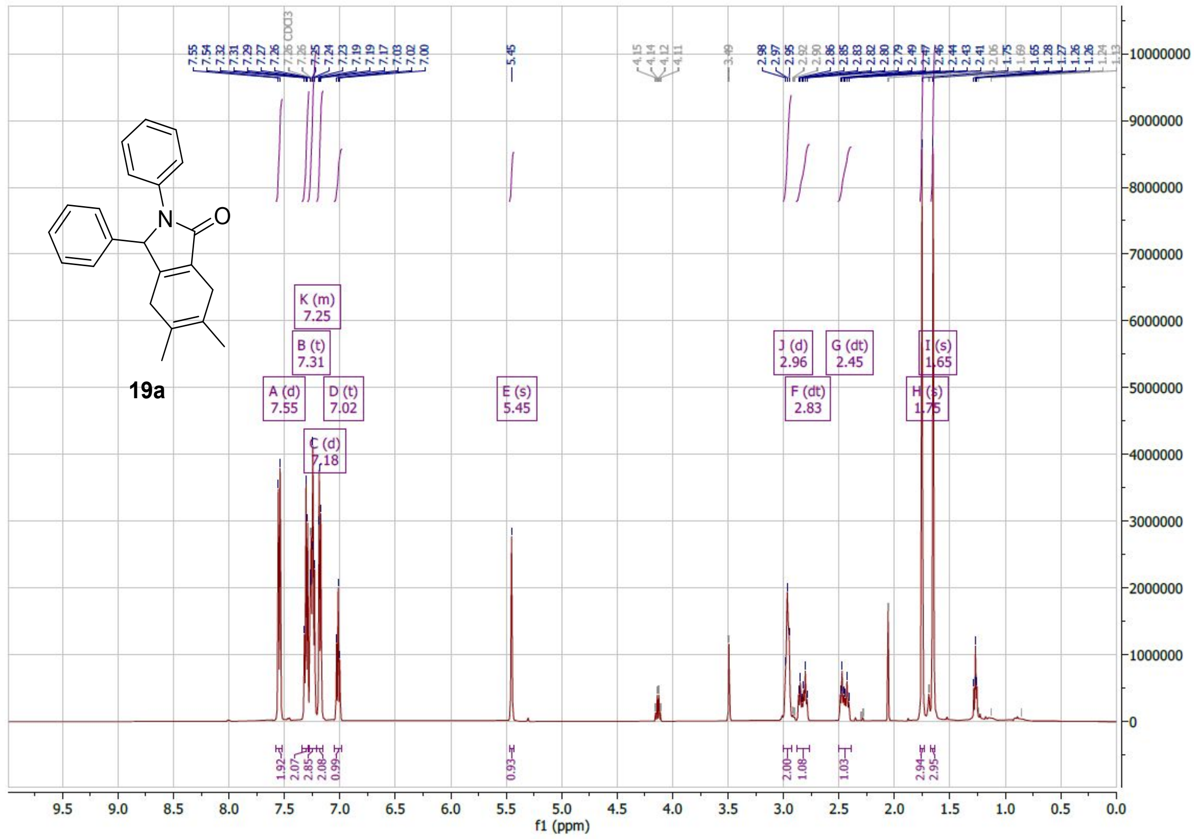

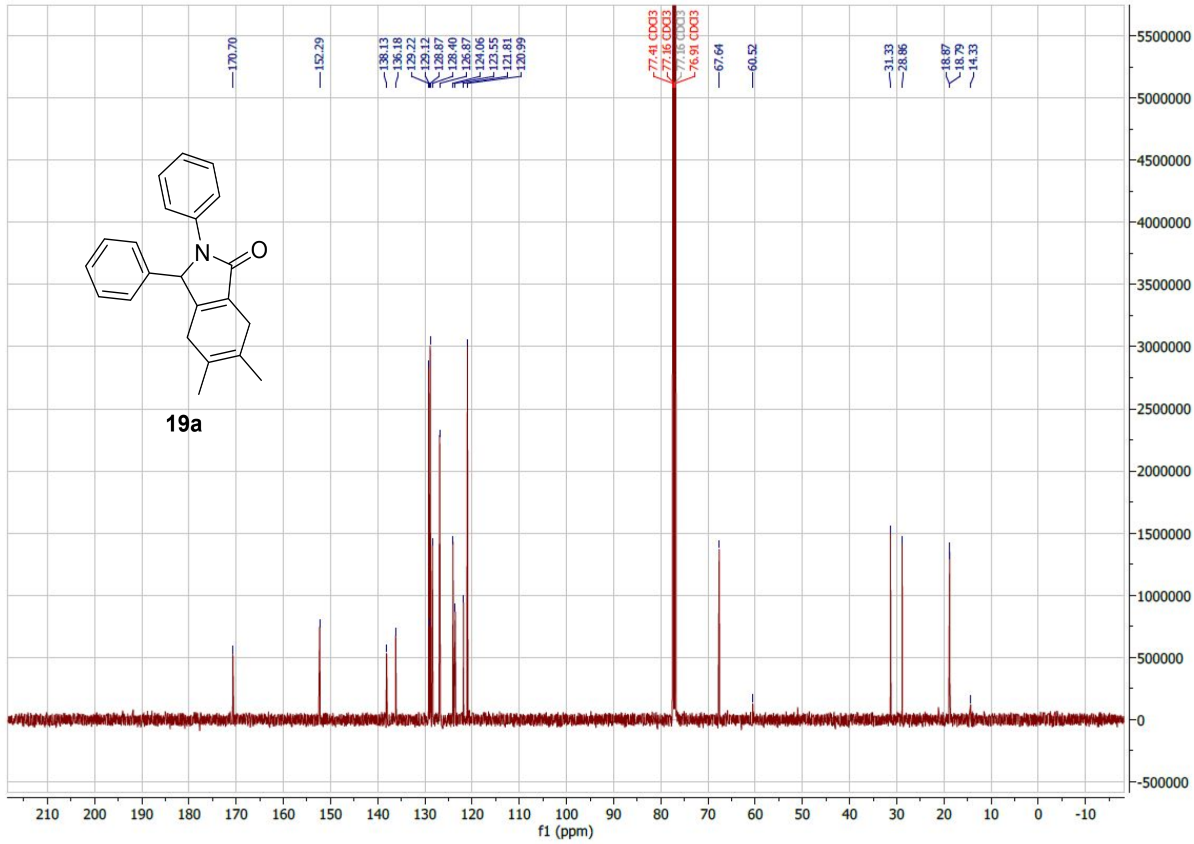

^{1}H and ^{13}C NMR spectra of 4-Benzoyl-*N*-phenyl-1*H*-pyrrole-3-carboxamide (20a)

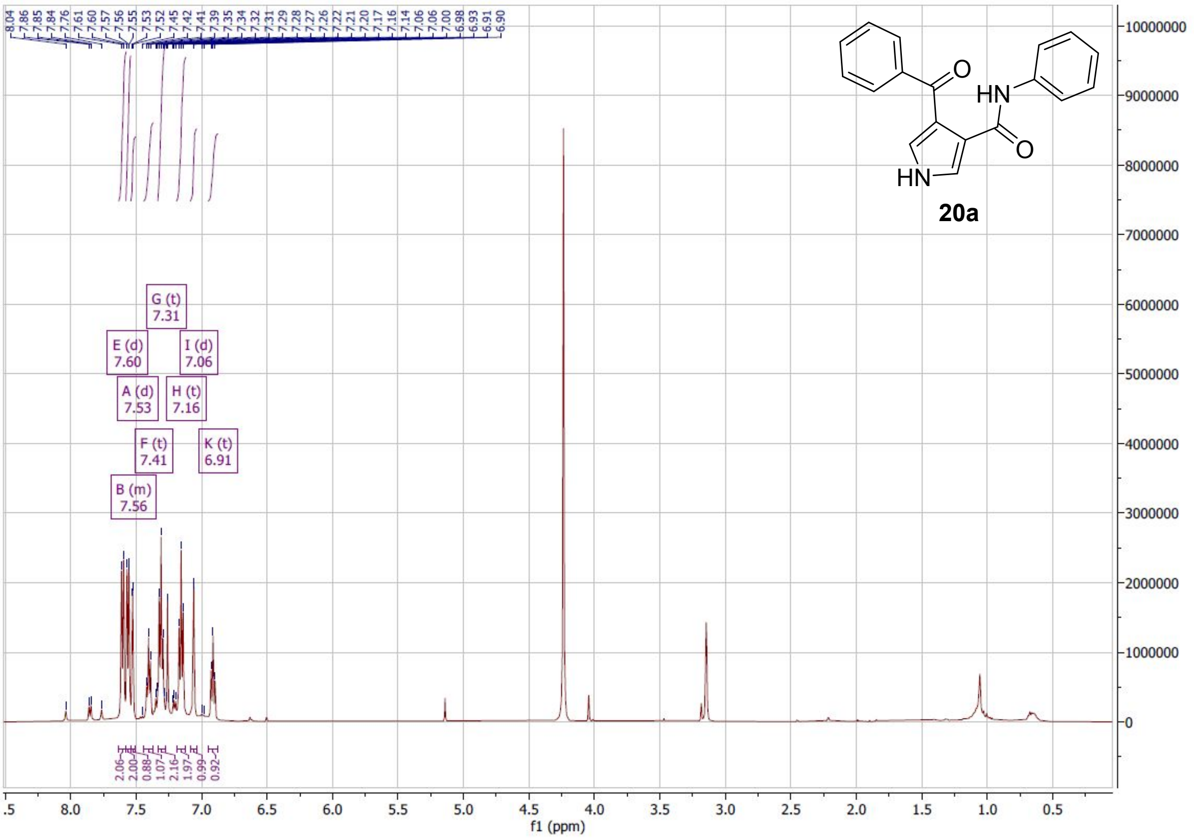

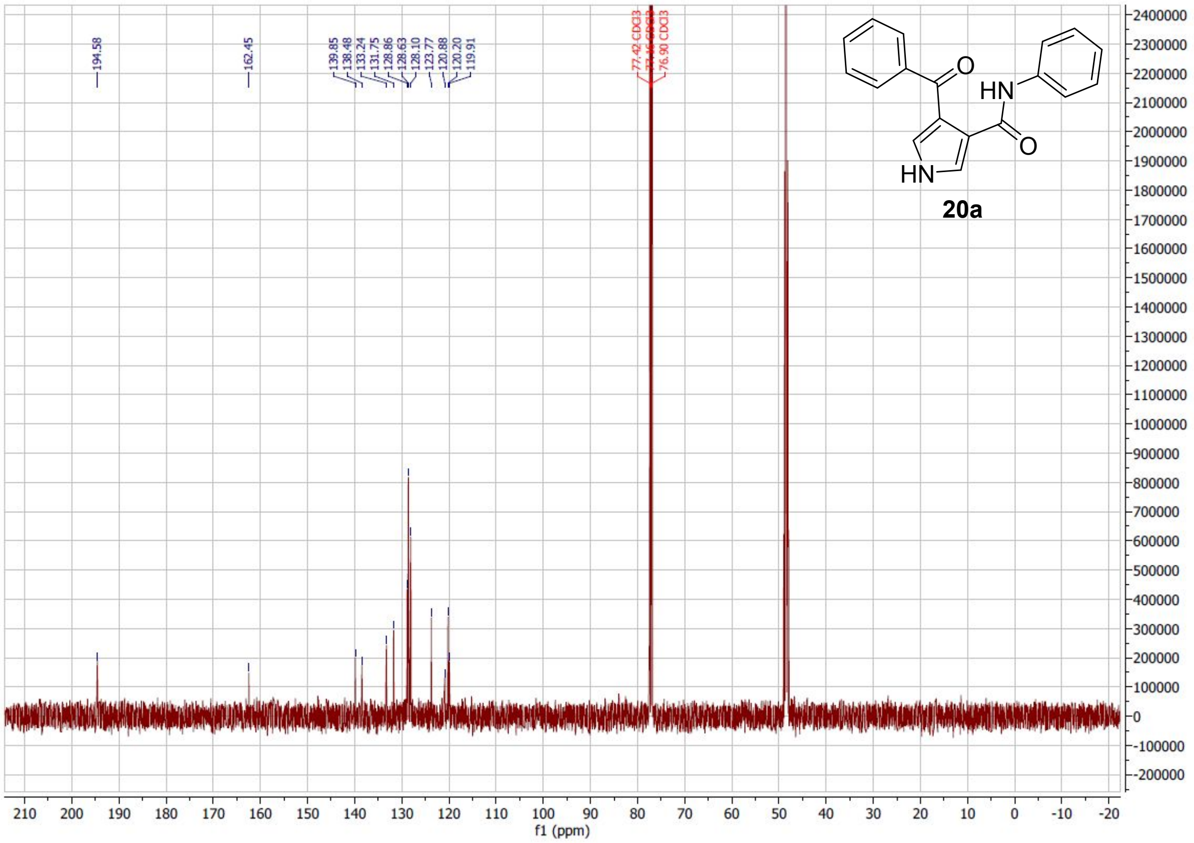

^{1}H and ^{13}C NMR spectra of *N*-(3,4-dimethoxyphenyl)-4-(4-methoxybenzoyl)-1*H*-pyrrole-3-carboxamide (20b)

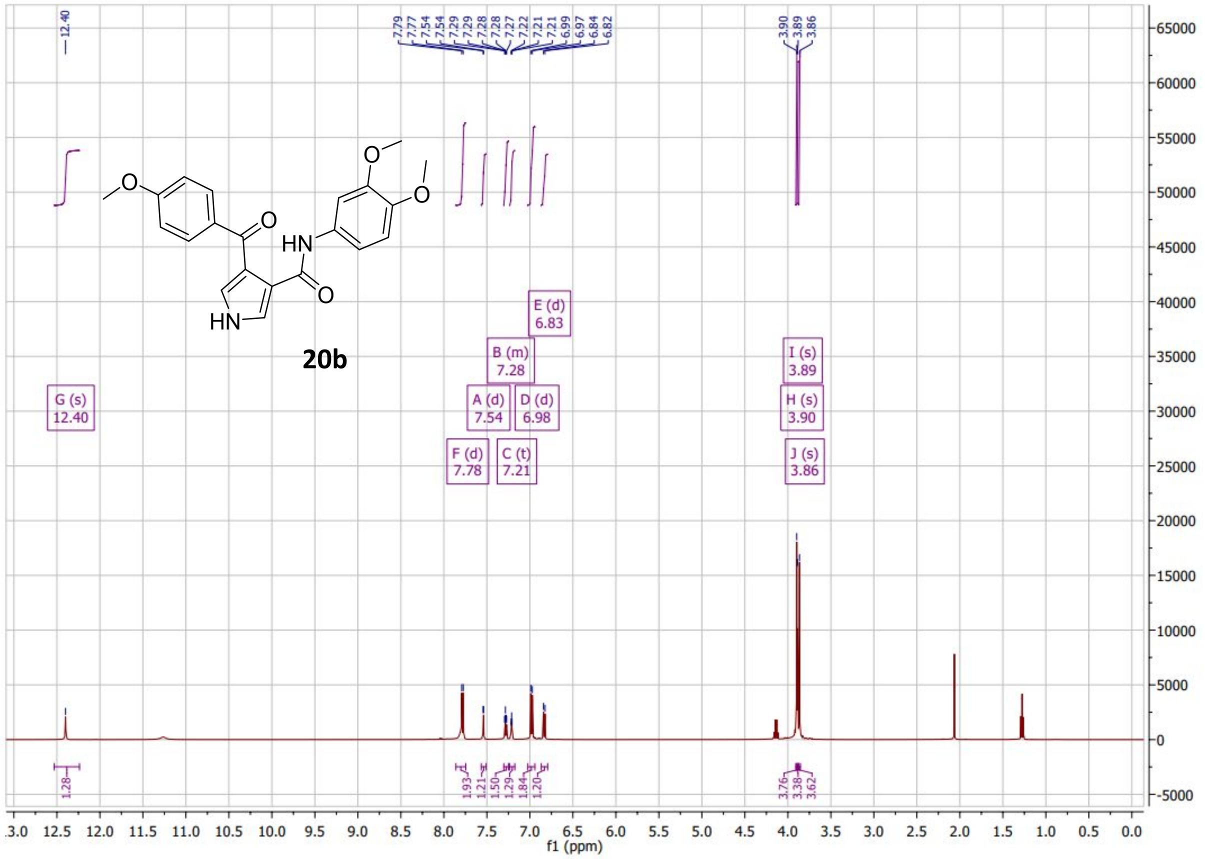

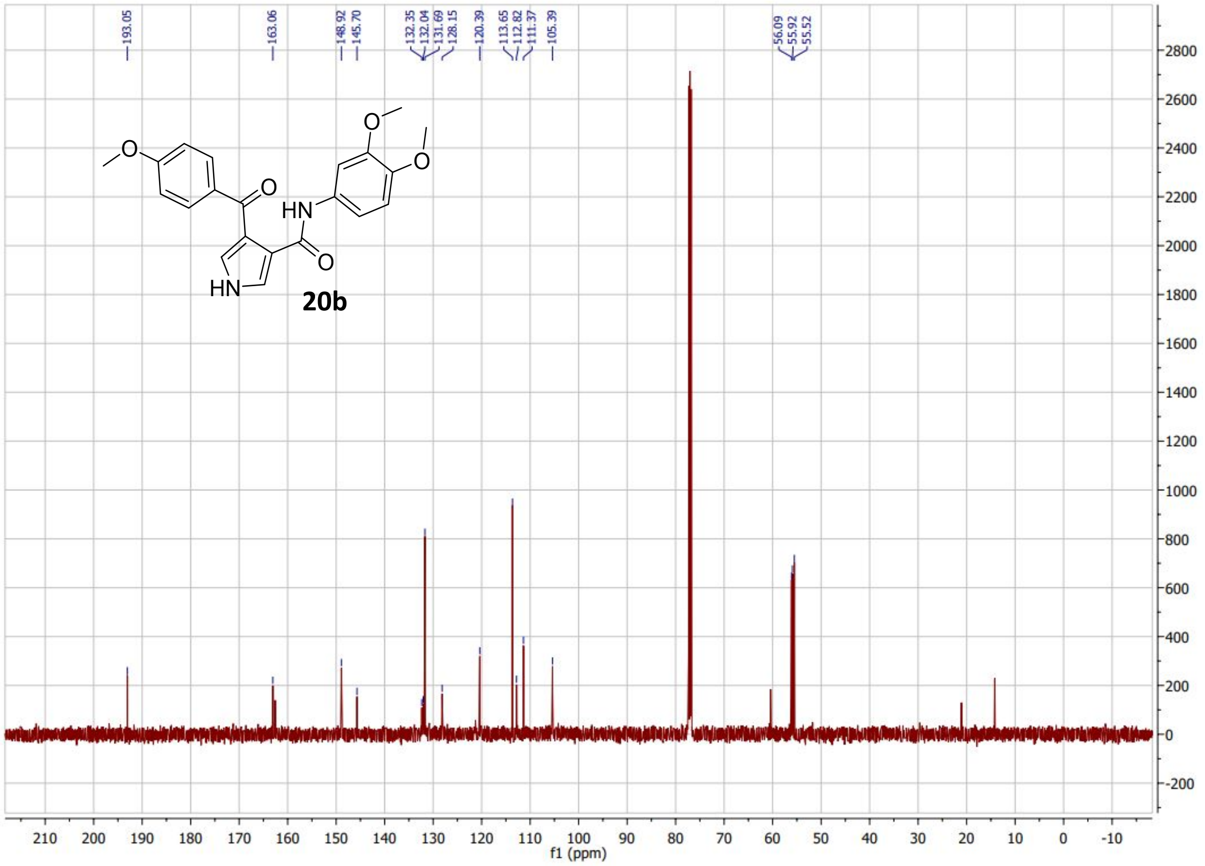

^{1}H and ^{13}C NMR spectra of 5-Benzoyl-1-benzyl-*N*-phenyl-1*H*-1,2,3-triazole-4-carboxamide (21a)

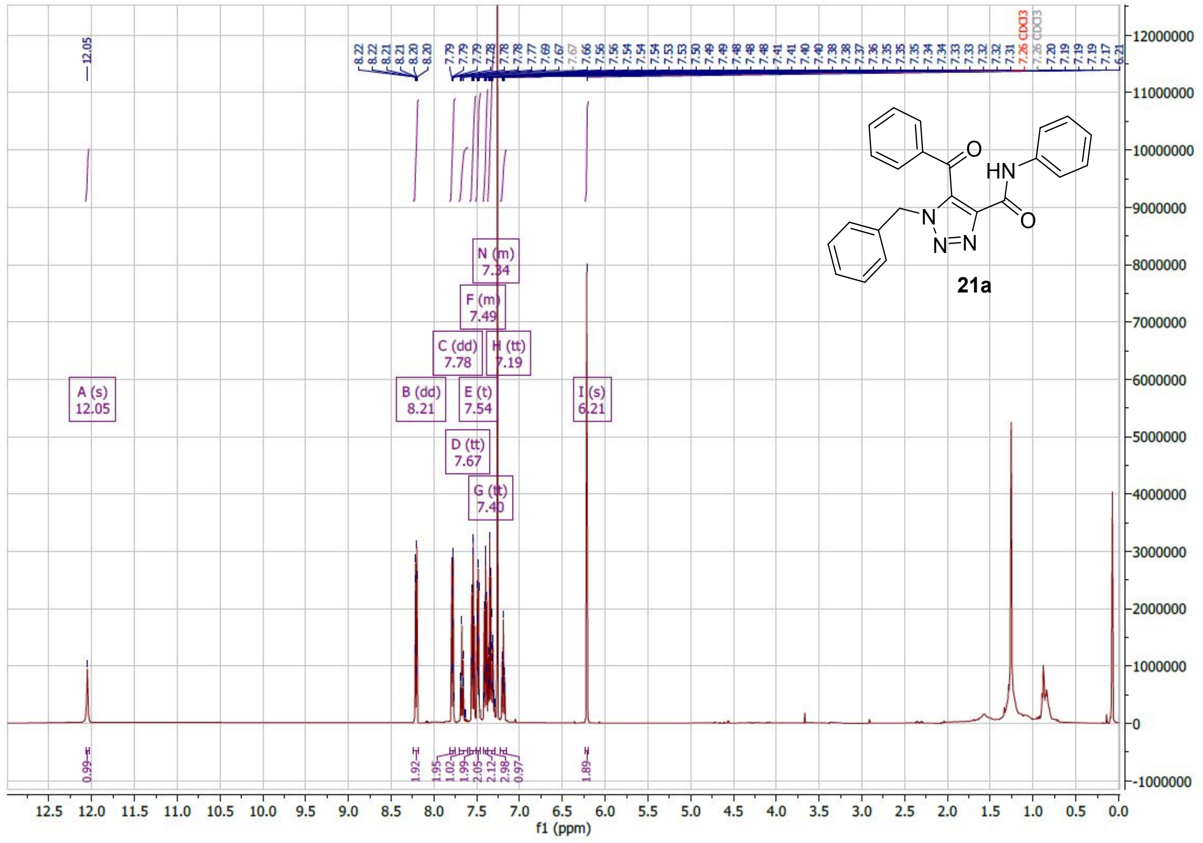

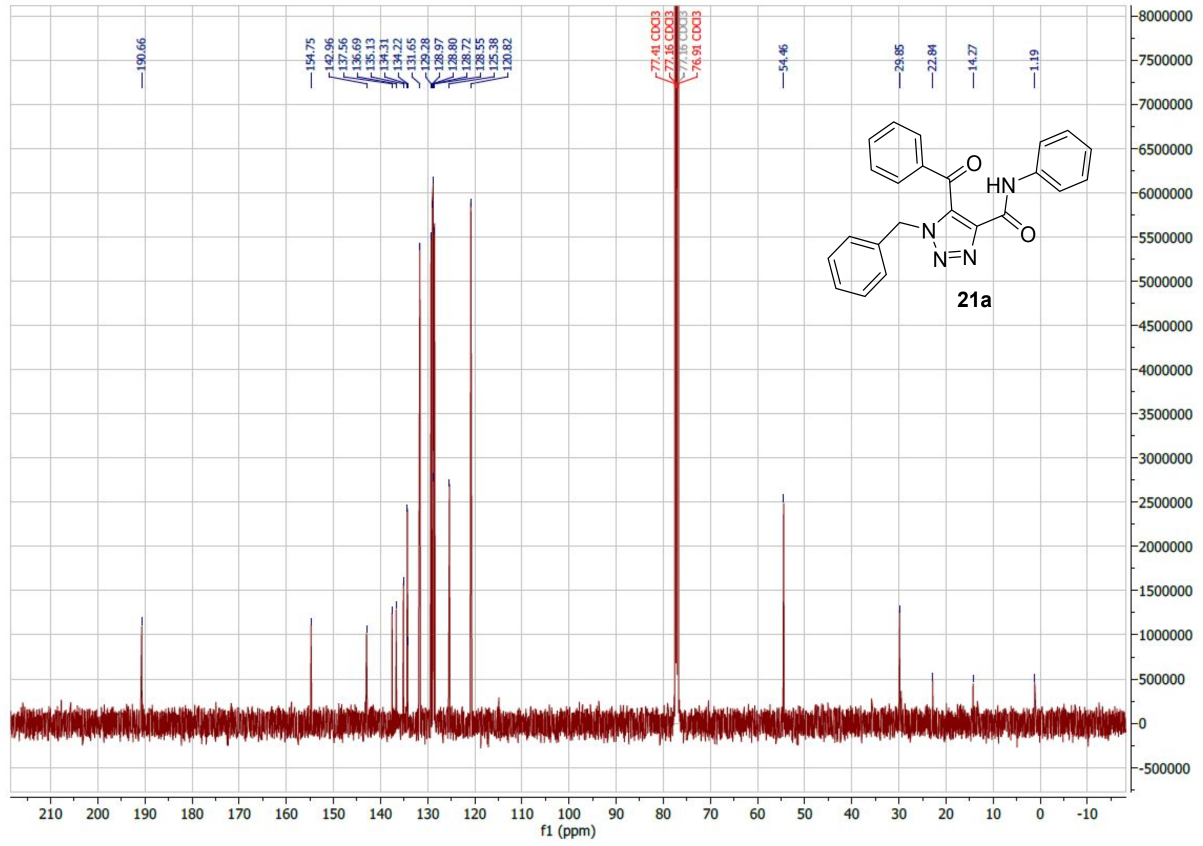

^{1}H and ^{13}C NMR spectra of 1-benzyl-N-(3,4-dimethoxyphenyl)-4-(4-methoxybenzoyl)-1H-1,2,3-triazole-5-carboxamide (21b)

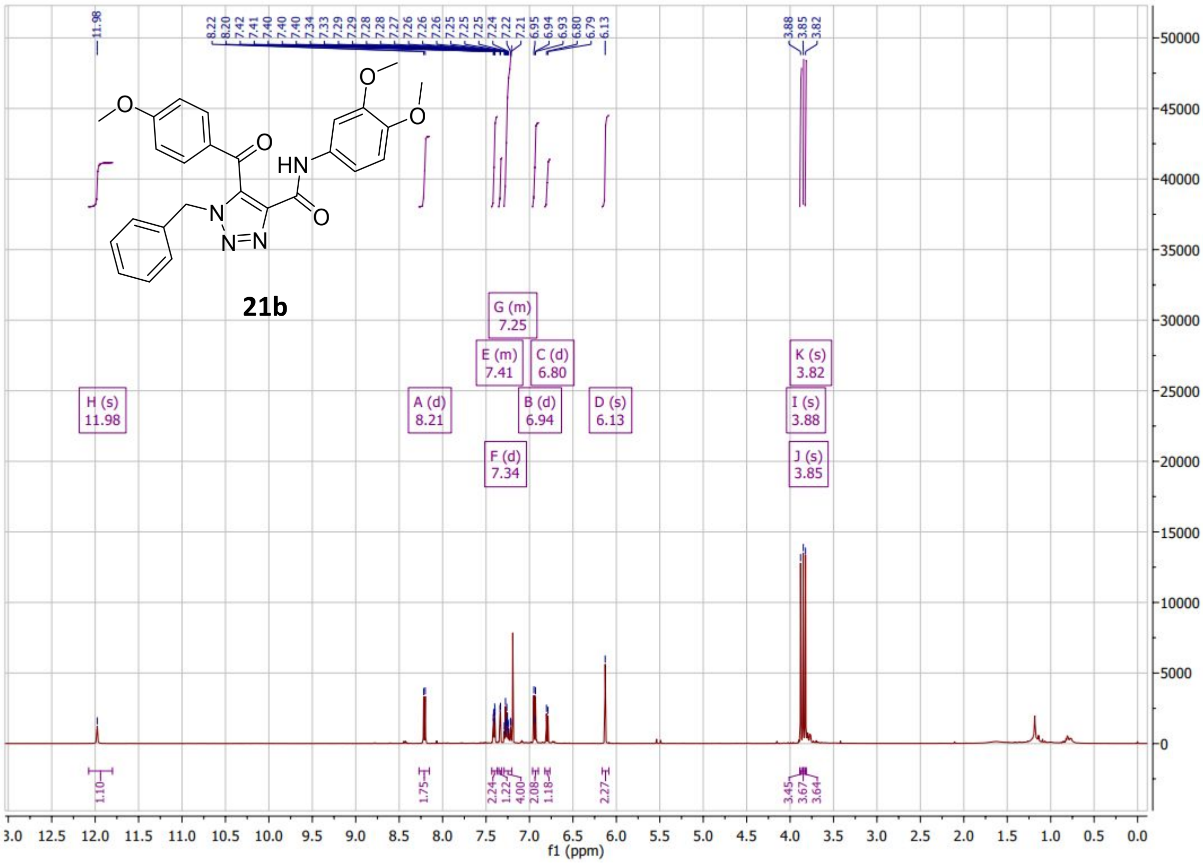

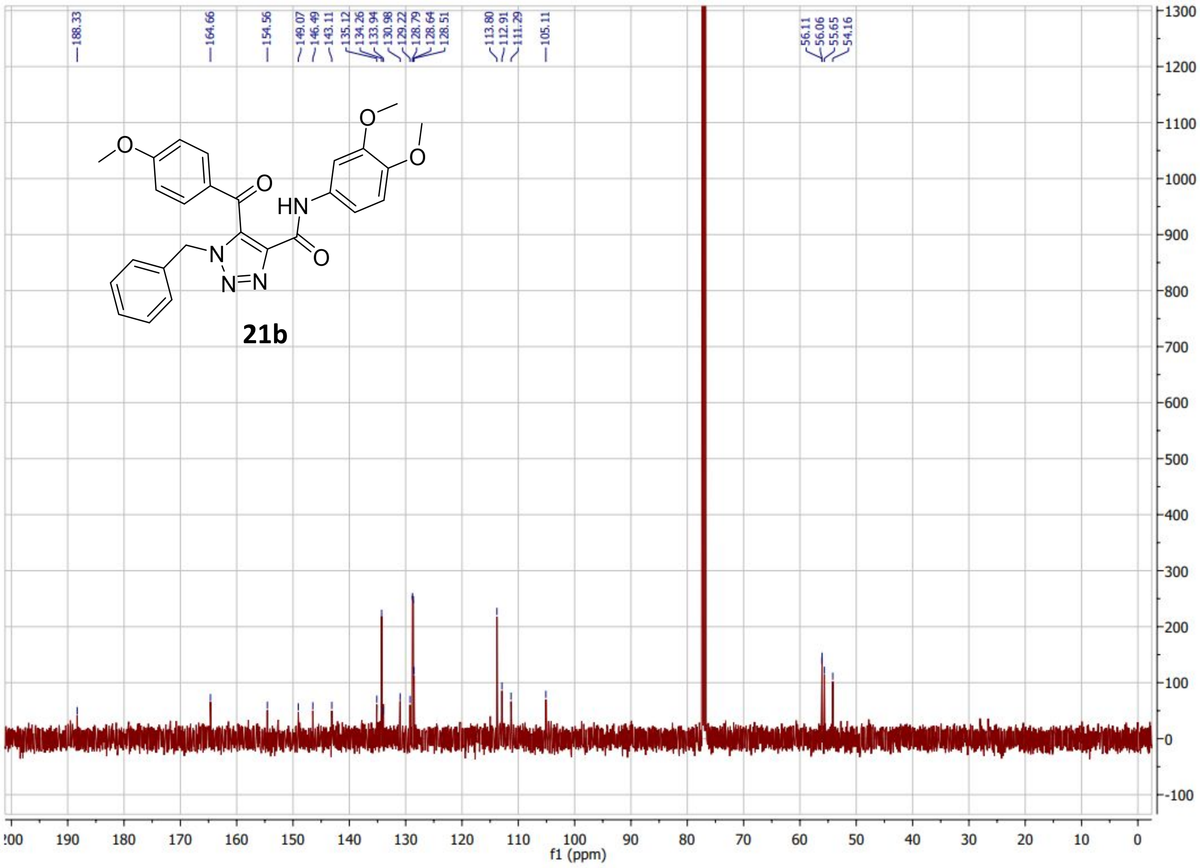

¹H and ¹³C NMR spectra of 4-Benzoyl-*N*-phenyl-1*H*-1,2,3-triazole-5-carboxamide (22a)

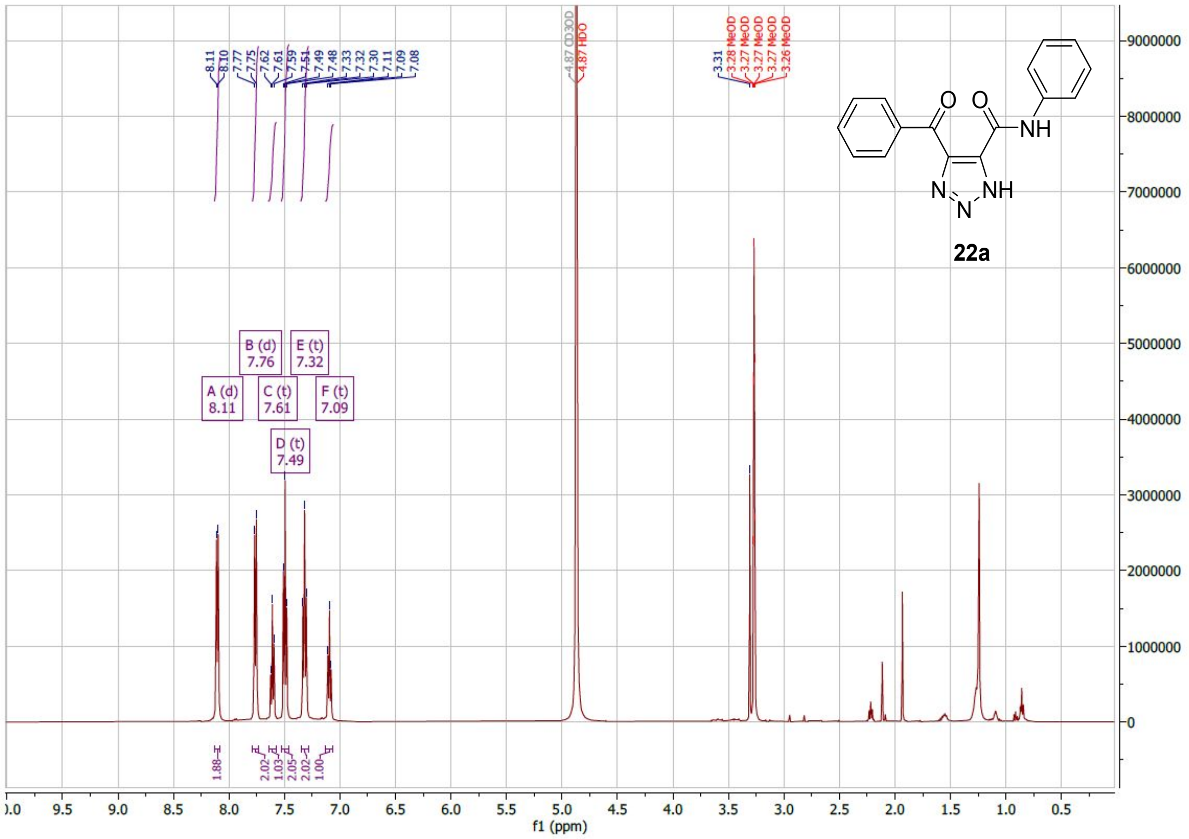

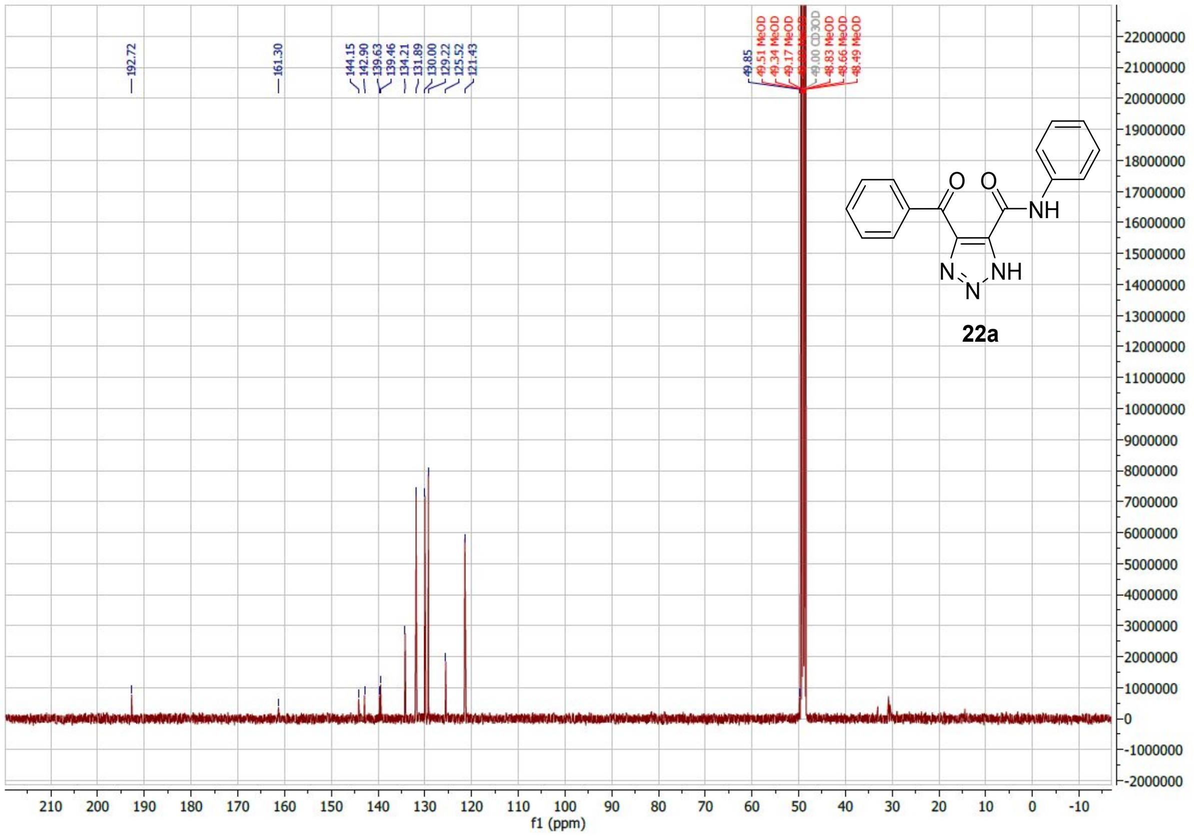

^{1}H and ^{13}C NMR spectra of *N*-(3,4-dimethoxyphenyl)-4-(4-methoxybenzoyl)-1*H*-1,2,3-triazole-5-carboxamide (22b)

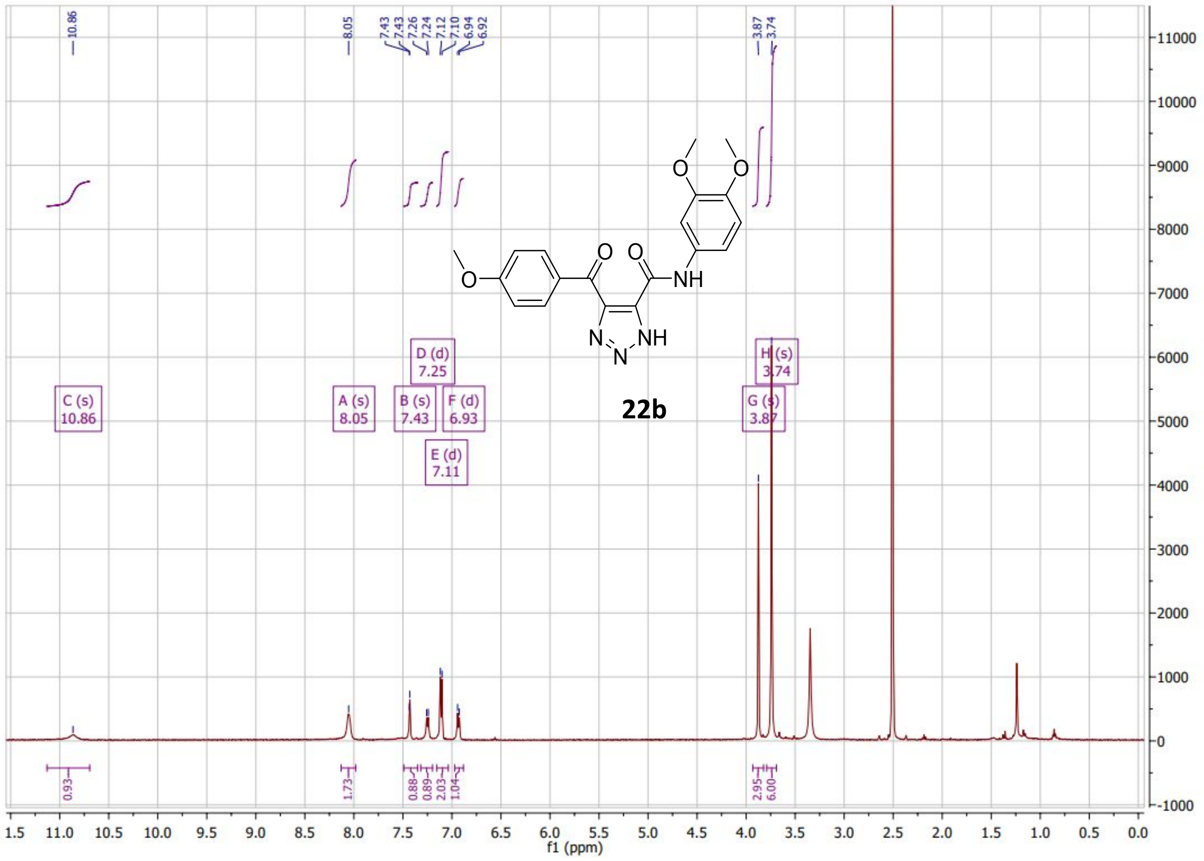

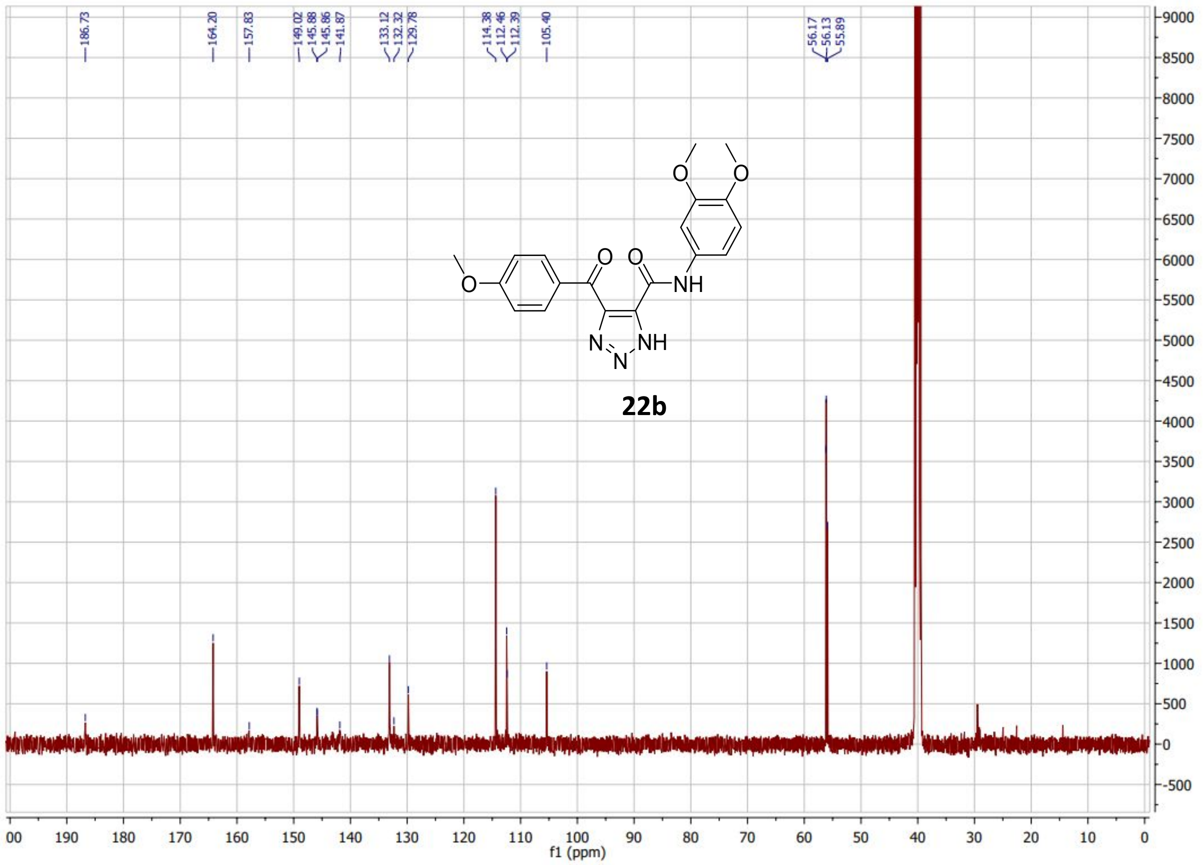

¹H and ¹³C NMR spectra of (*Z*)-*N*-Methylpropan-1-imine oxide (23')

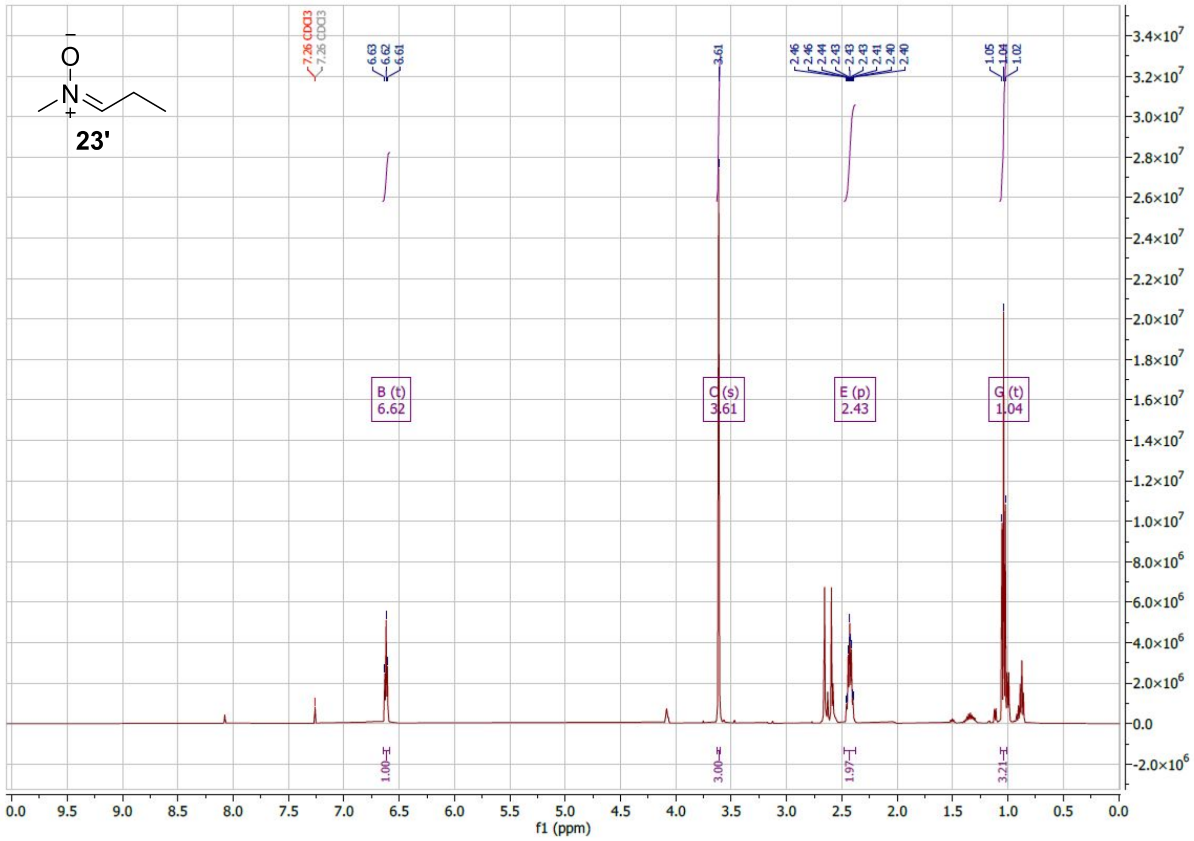

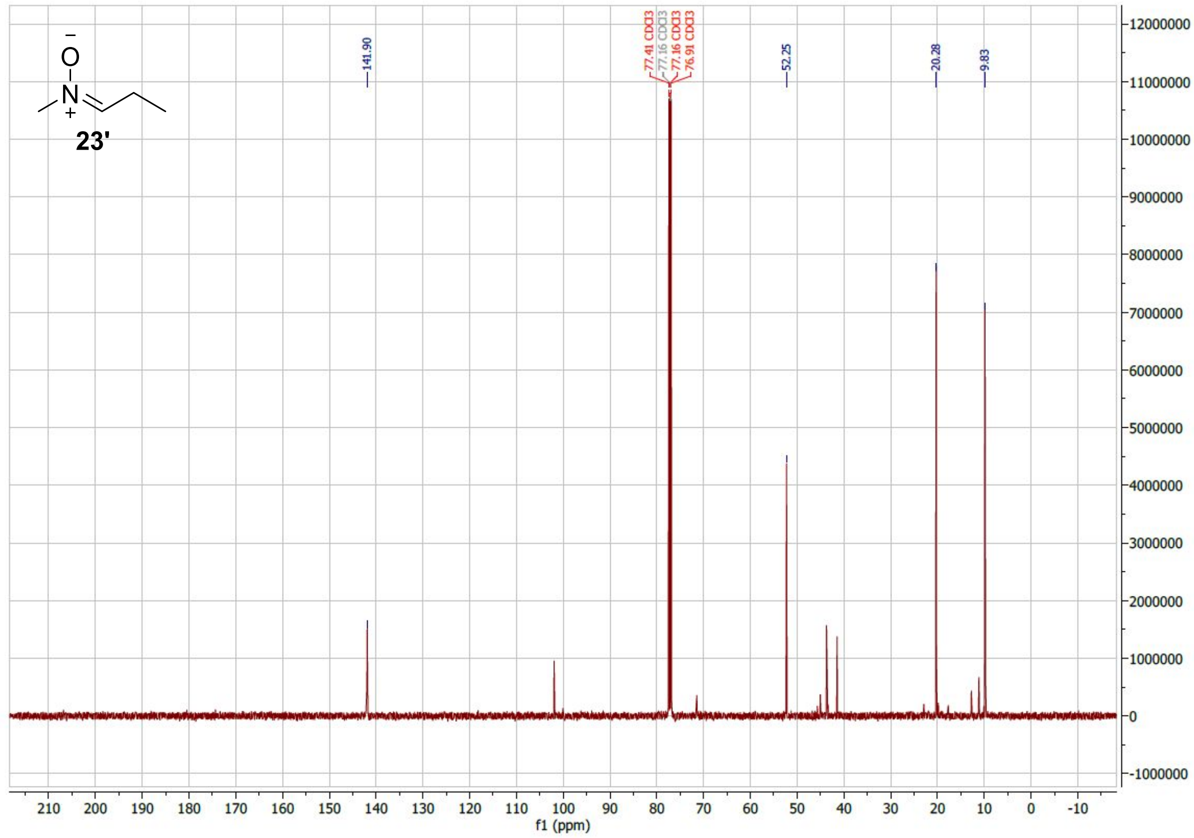

¹H and ¹³C NMR spectra of (3*S*,4*R*,5*R*)/(3*R*,4*S*,5*S*)-5-Benzoyl-3-ethyl-2-methyl-*N*-phenylisoxazolidine-4-carboxamide (23a-A)

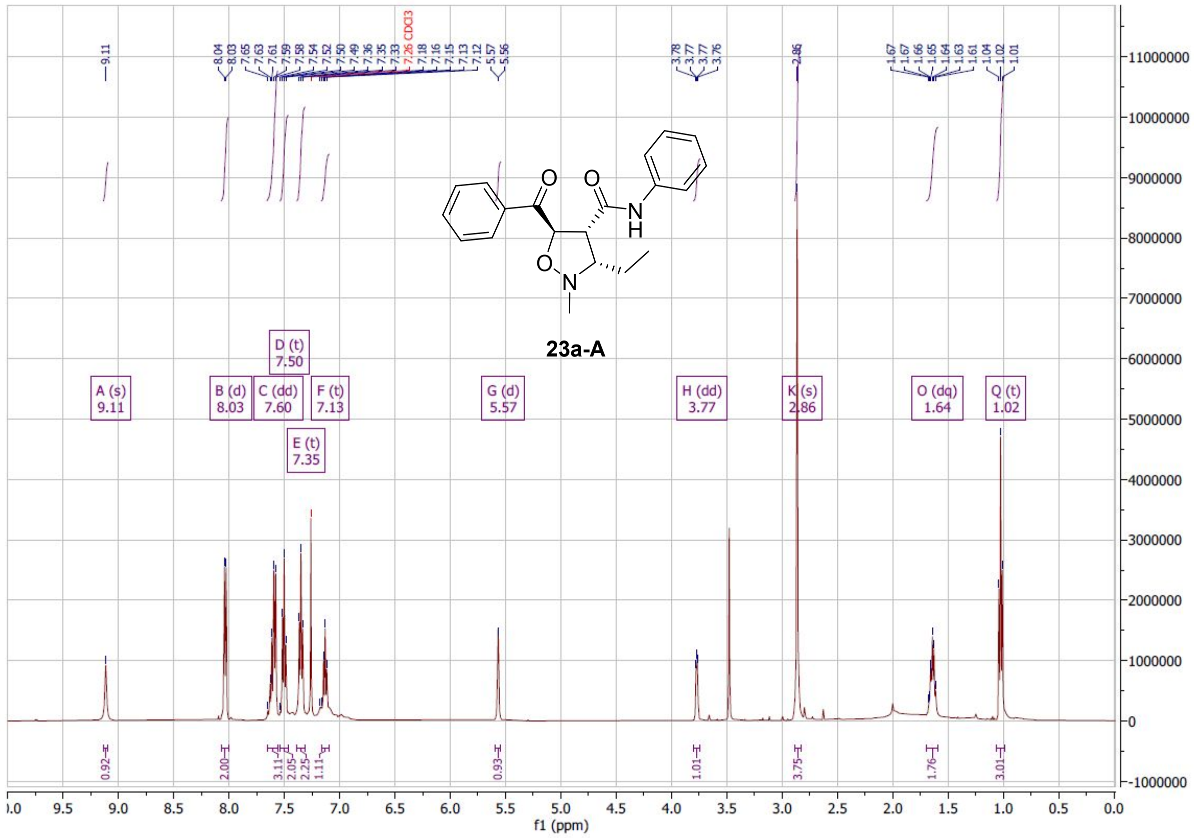

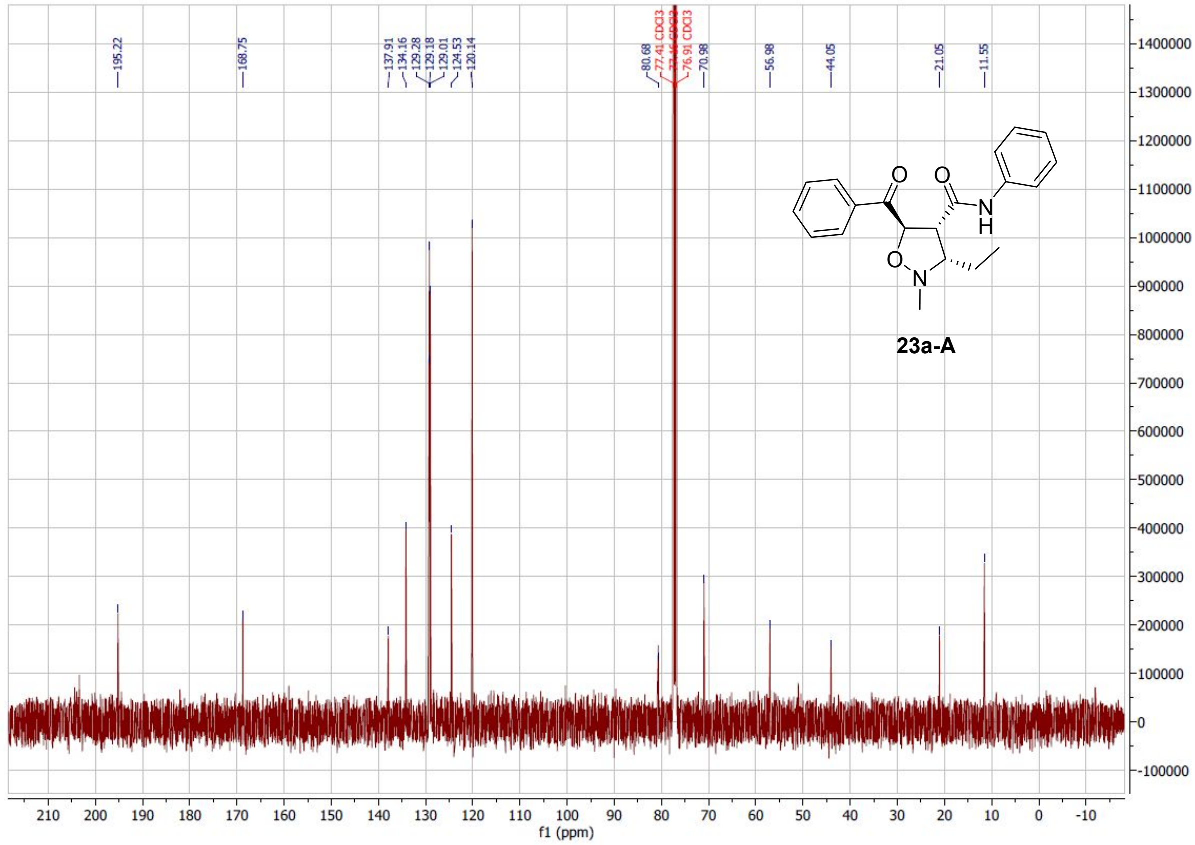

¹H and ¹³C NMR spectra of (3*R*,4*R*,5*R*)/(3*S*,4*S*,5*S*)-4-Benzoyl-3-ethyl-2-methyl-*N*-phenylisoxazolidine-5-carboxamide (23a-B)

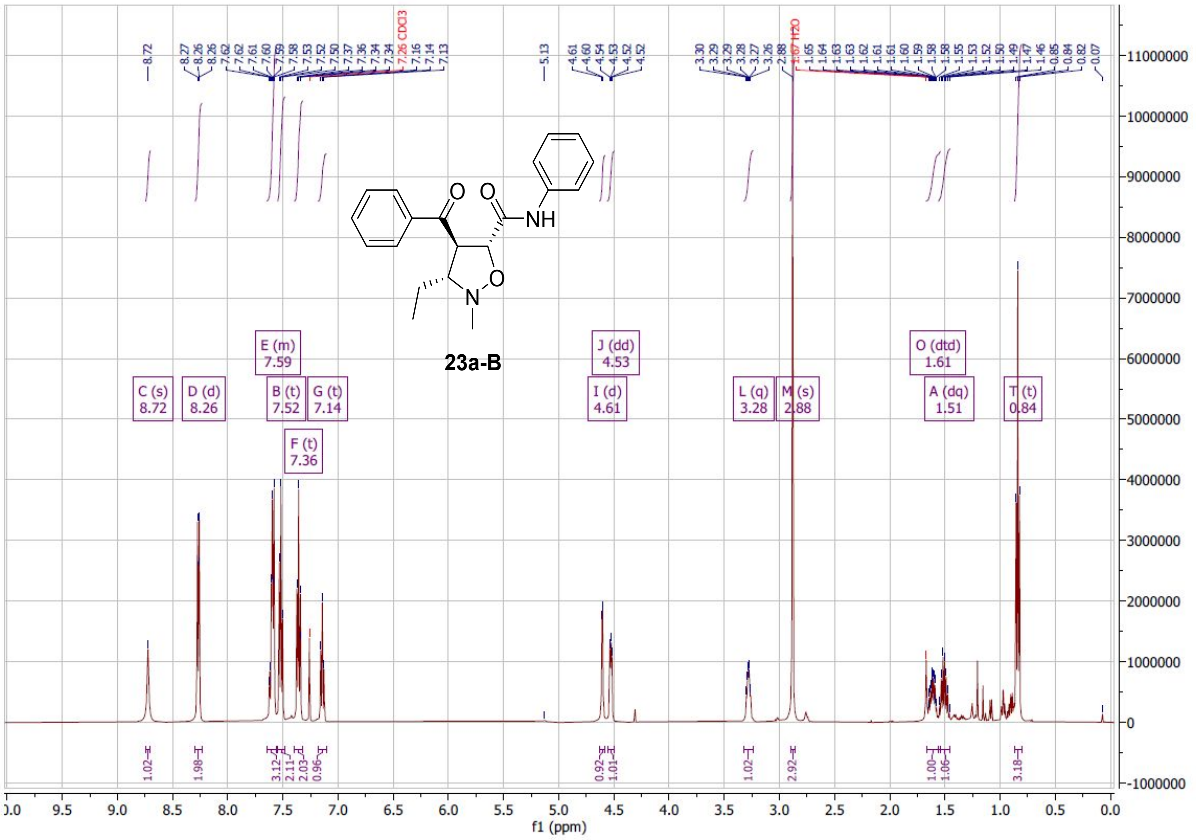

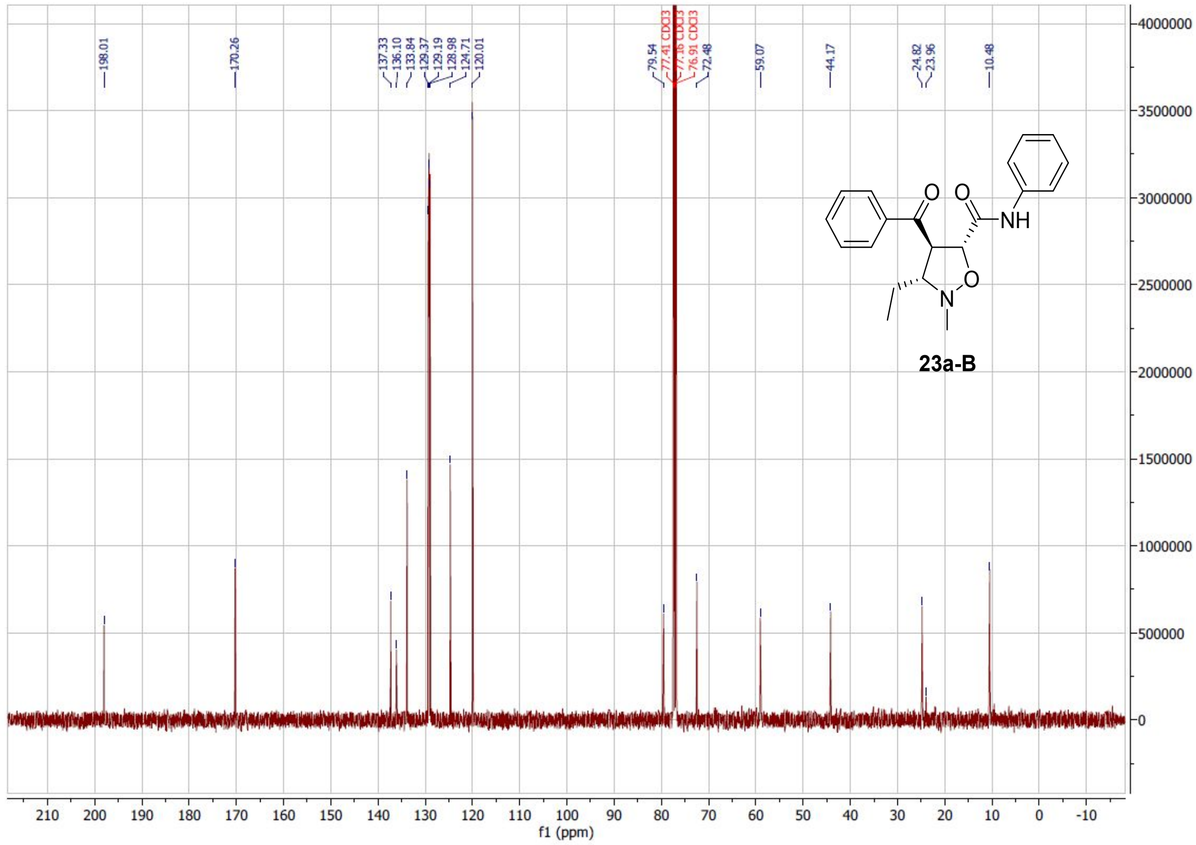

^{1}H and ^{13}C NMR spectra of (3*R*,4*R*,5*R*)/(3*S*,4*S*,5*S*)-5-Benzoyl-3-ethyl-2-methyl-*N*-phenylisoxazolidine-4-carboxamide (23a-C)

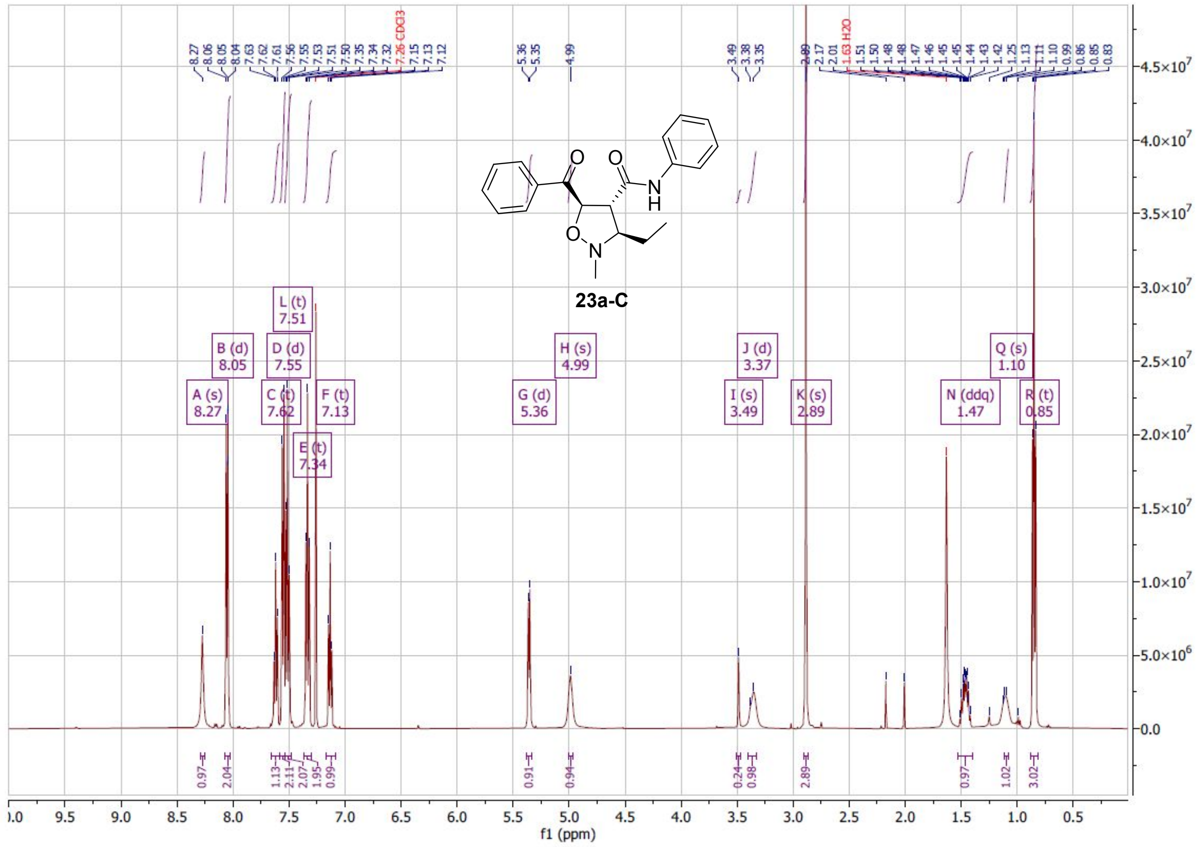

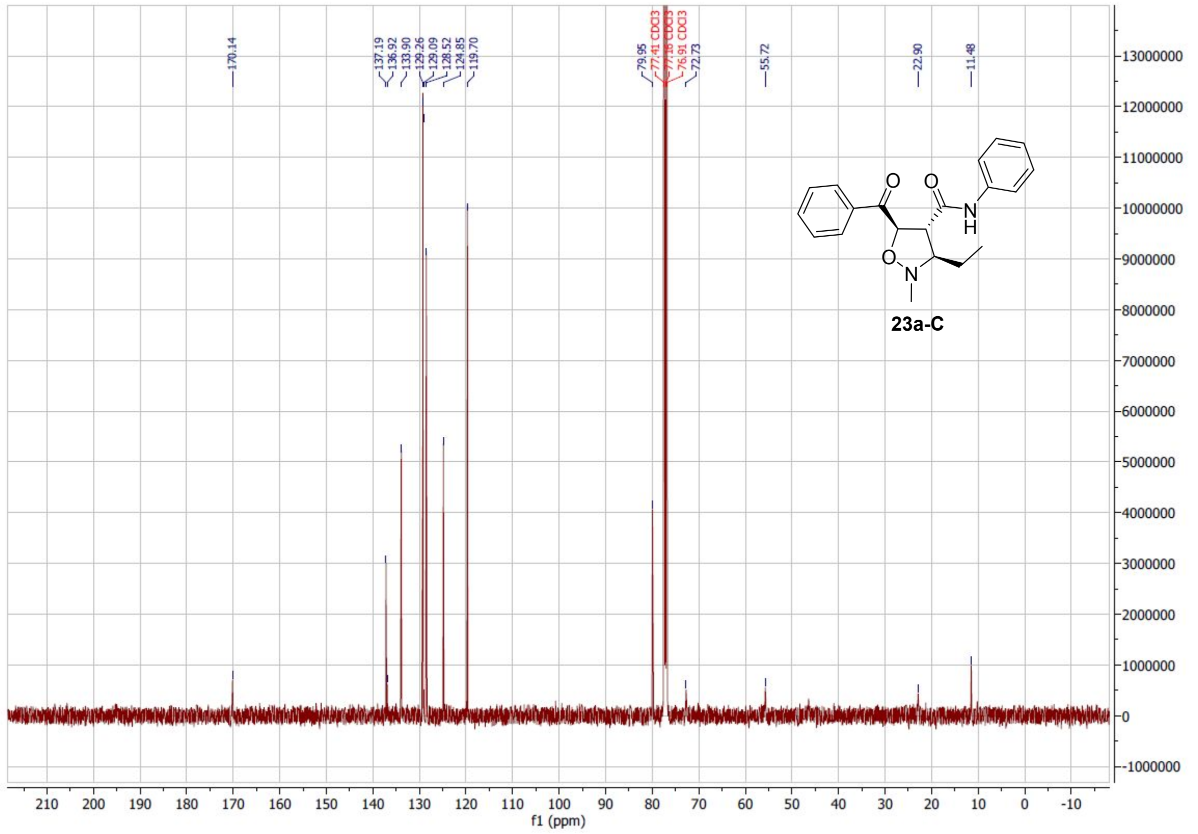

^{1}H and ^{13}C NMR spectra of (3*S*,4*R*,5*R*)/(3*R*,4*S*,5*S*)-4-benzoyl-3-ethyl-2-methyl-*N*-phenylisoxazolidine-5-carboxamide (23a-D)

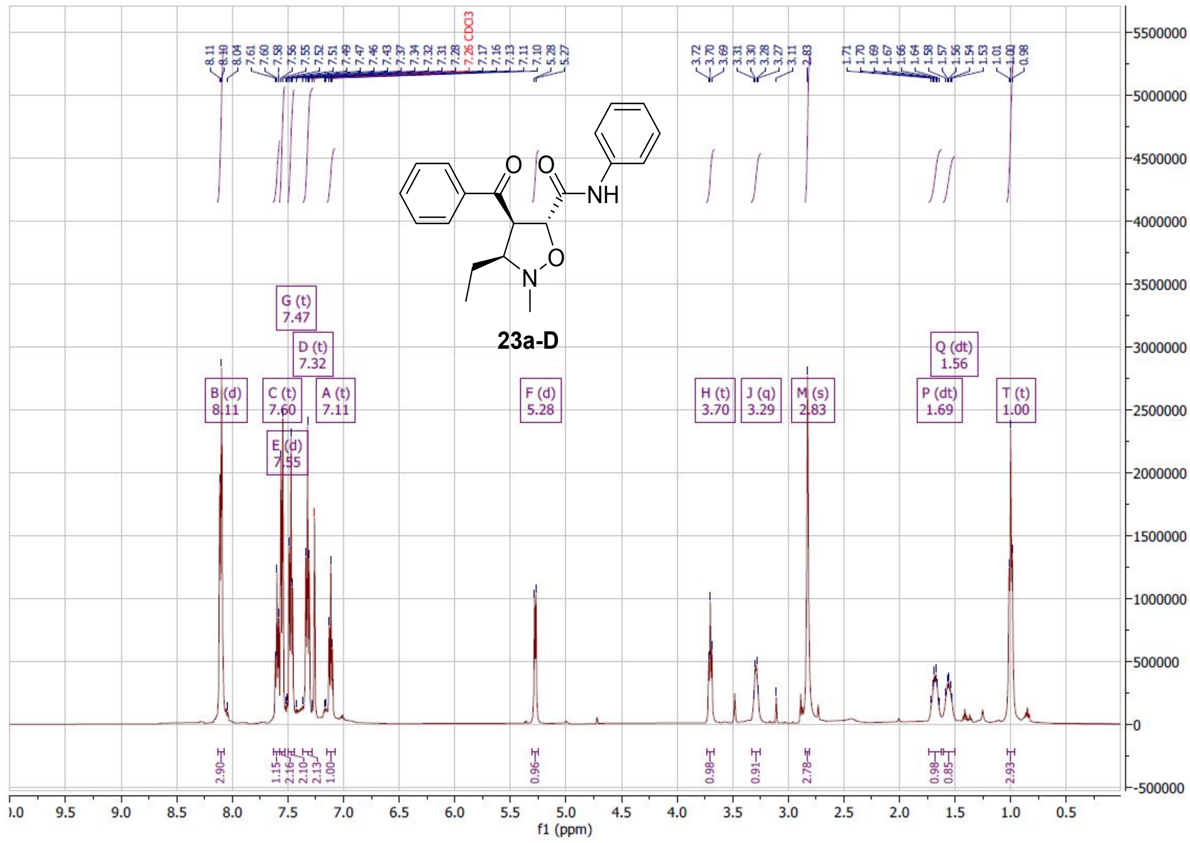

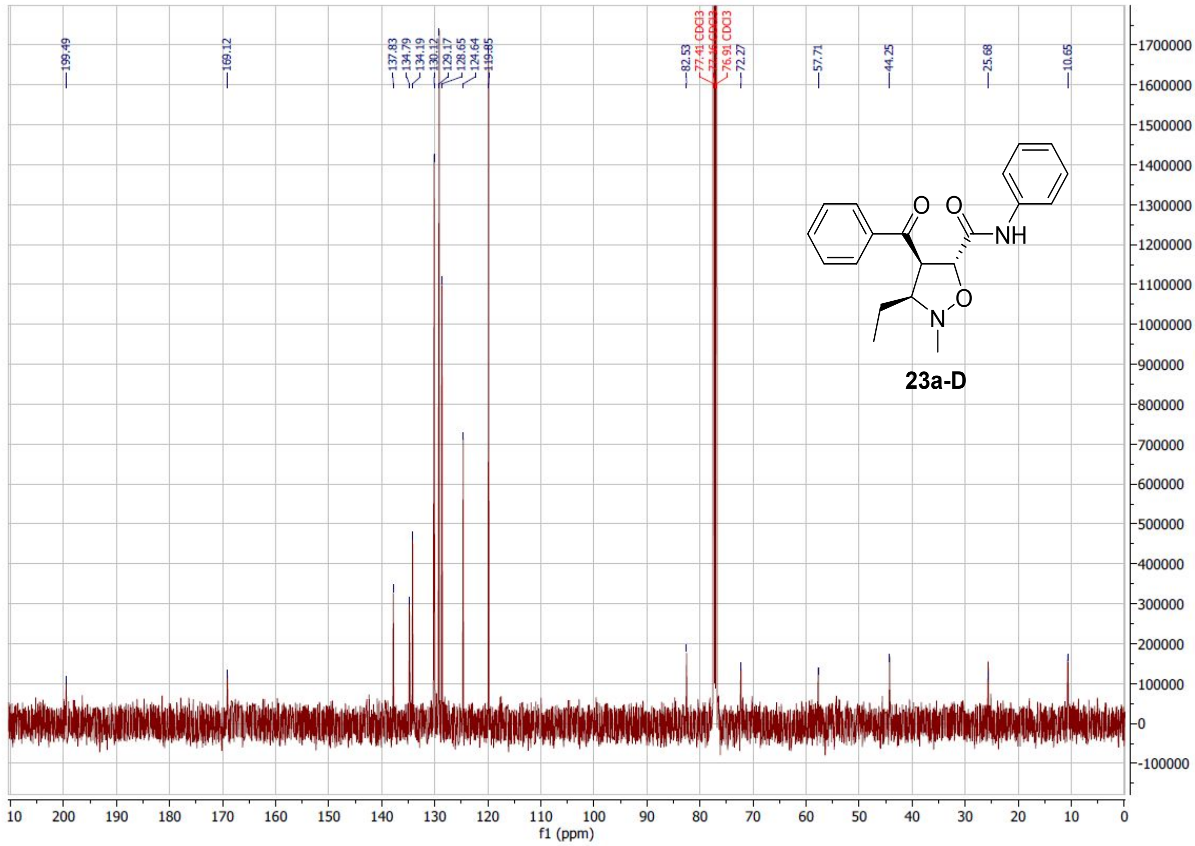

^{1}H and ^{13}C NMR spectra of (3*S*,4*R*,5*R*)-*N*-(3,4-dimethoxyphenyl)-3-ethyl-5-(4-methoxybenzoyl)-2-methylisoxazolidine-4-carboxamide (23b-A)

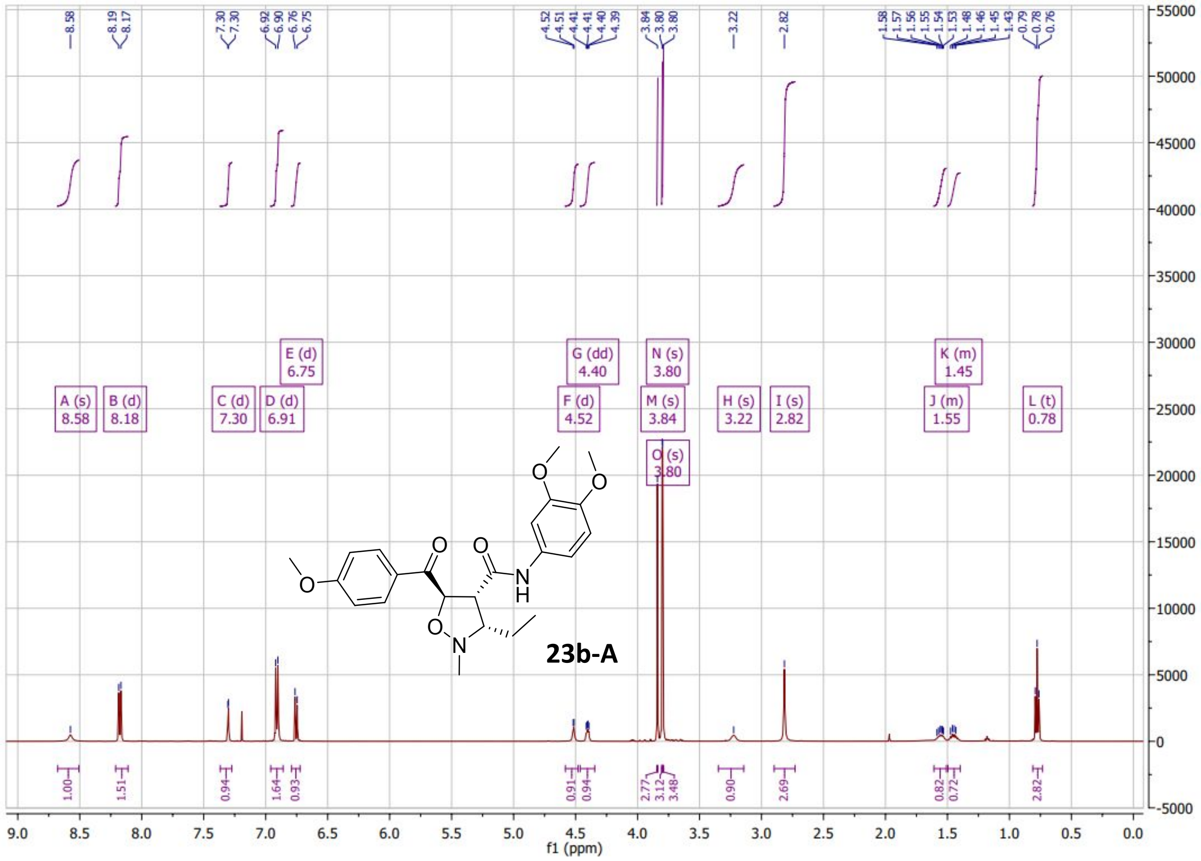

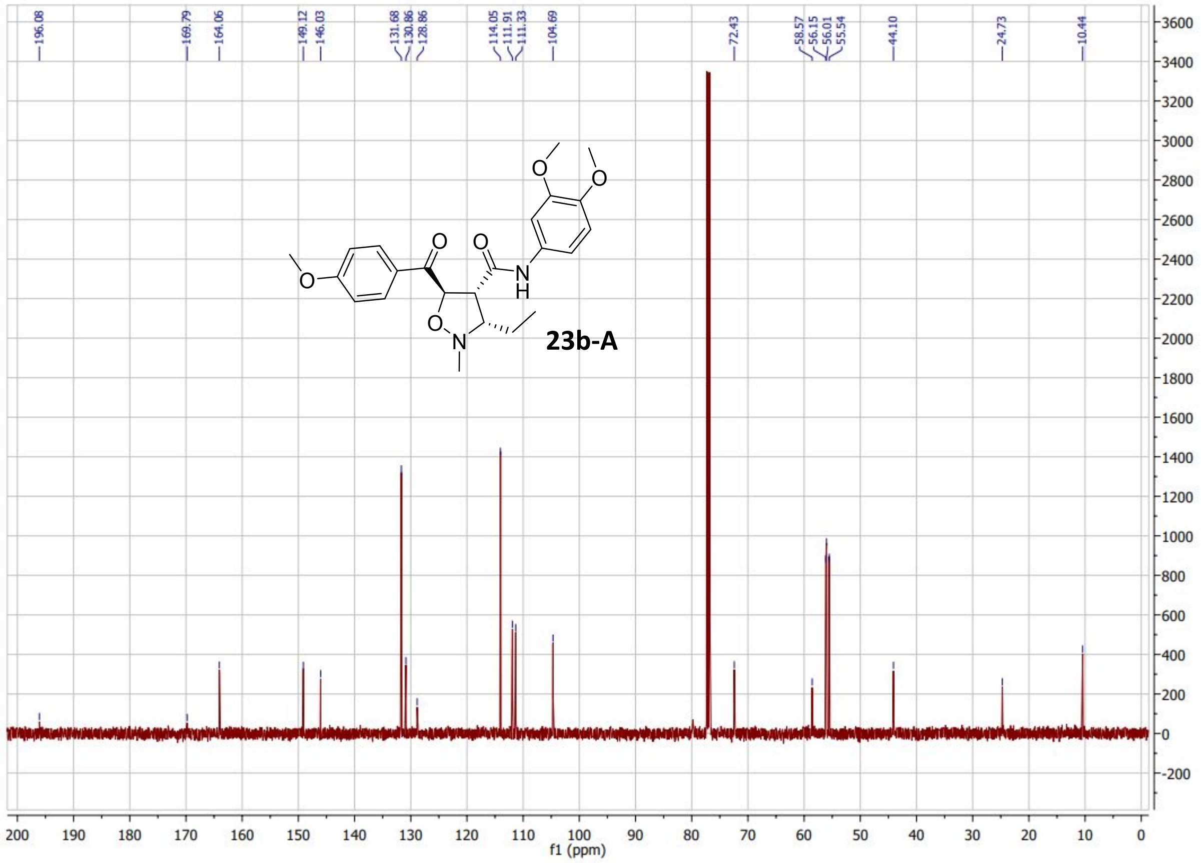

^{1}H and ^{13}C NMR spectra of (3*R*,4*R*,5*R*)-*N*-(3,4-dimethoxyphenyl)-3-ethyl-4-(4-methoxybenzoyl)-2-methylisoxazolidine-5-carboxamide (23b-B)

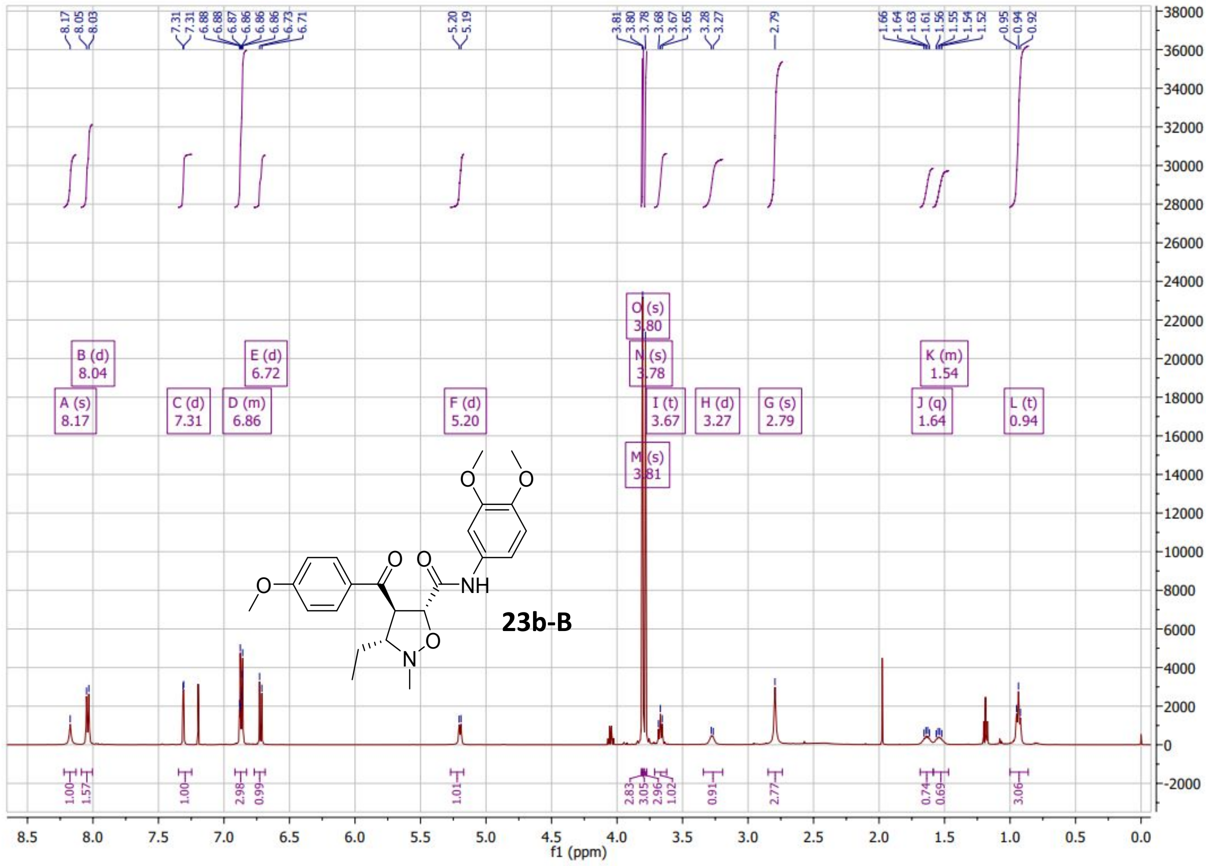

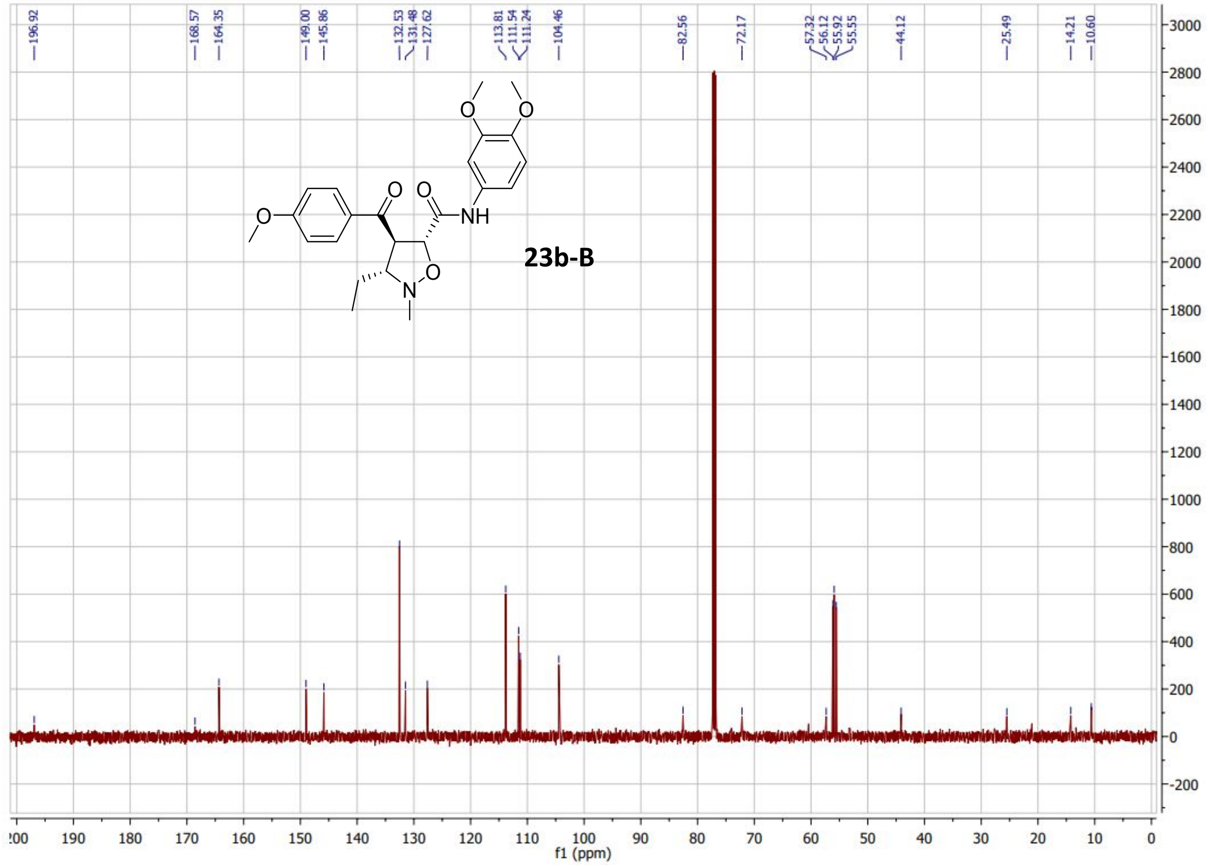

^{1}H and ^{13}C NMR spectra of (3*R*,4*R*,5*R*)-*N*-(3,4-dimethoxyphenyl)-3-ethyl-5-(4-methoxybenzoyl)-2-methylisoxazolidine-4-carboxamide (23b-C)

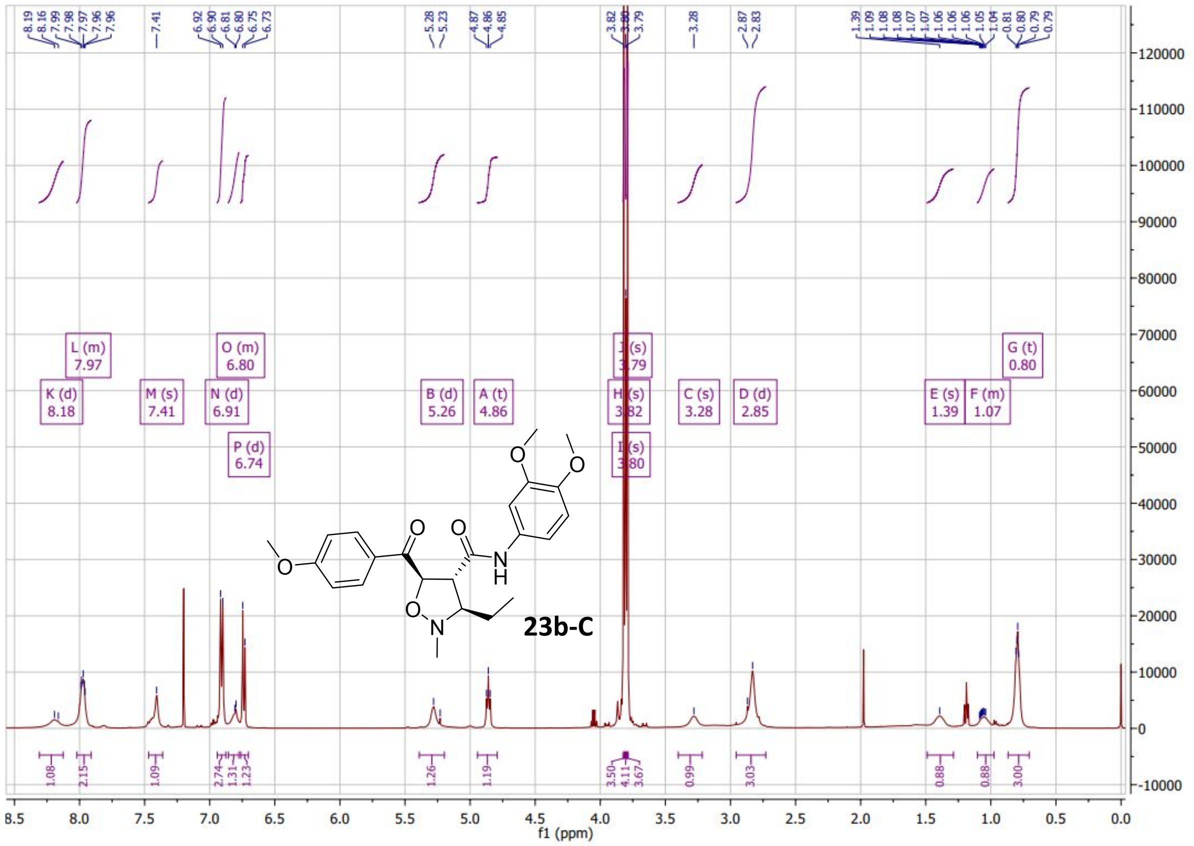

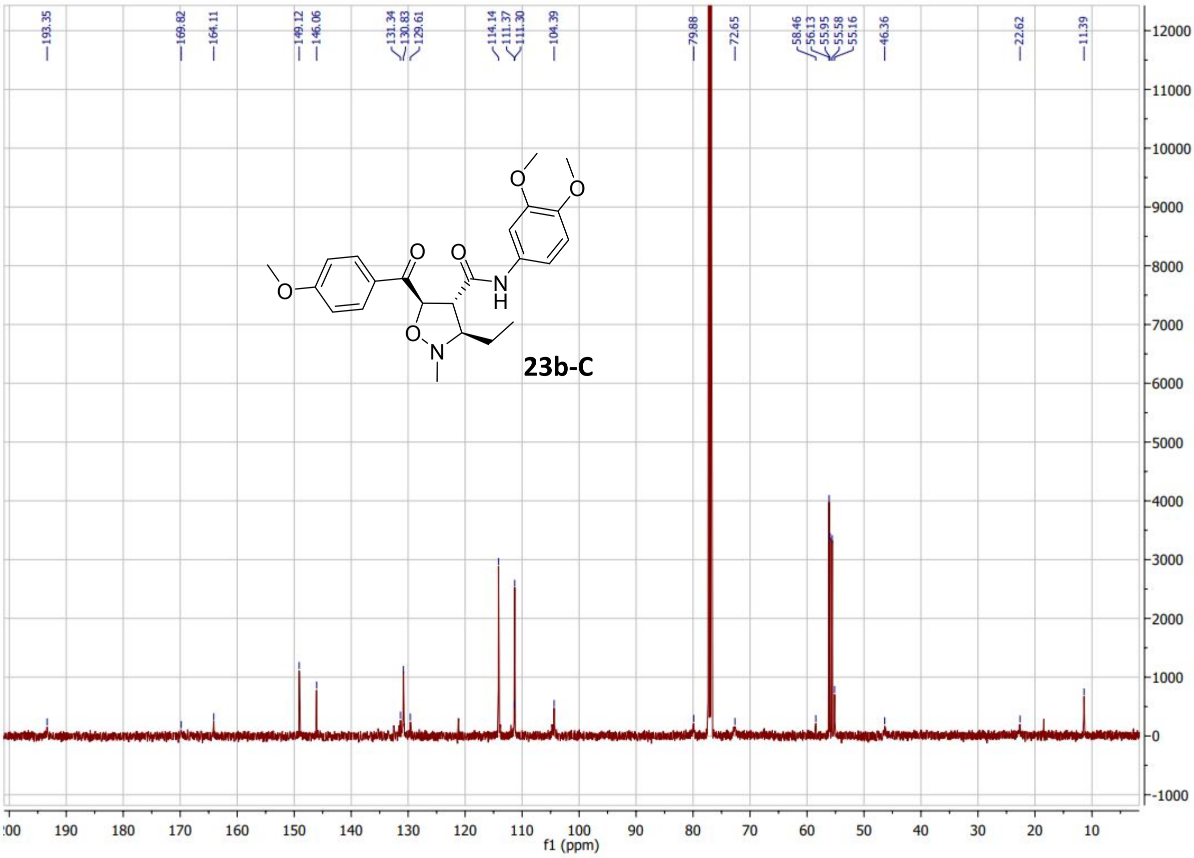

^{1}H and ^{13}C NMR spectra of (3*S*,4*R*,5*R*)-*N*-(3,4-dimethoxyphenyl)-3-ethyl-4-(4-methoxybenzoyl)-2-methylisoxazolidine-5-carboxamide (23b-D)

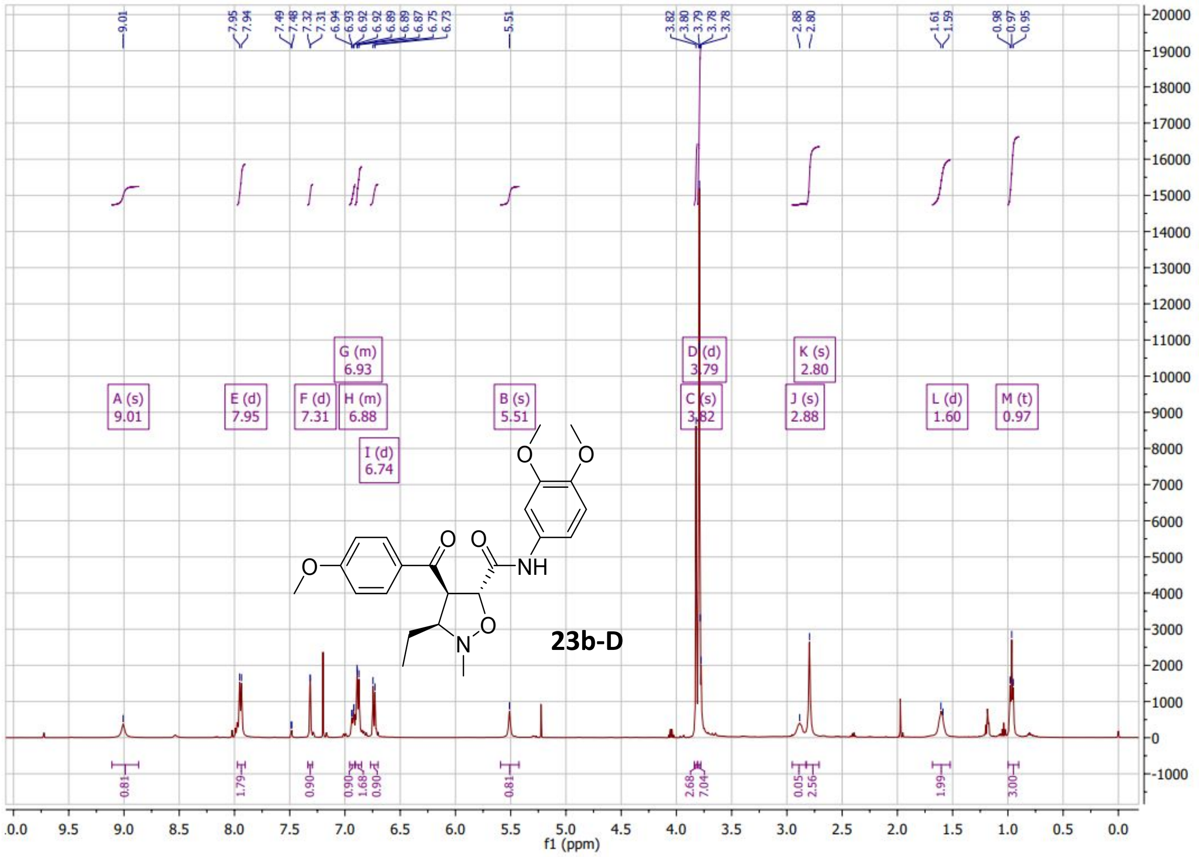

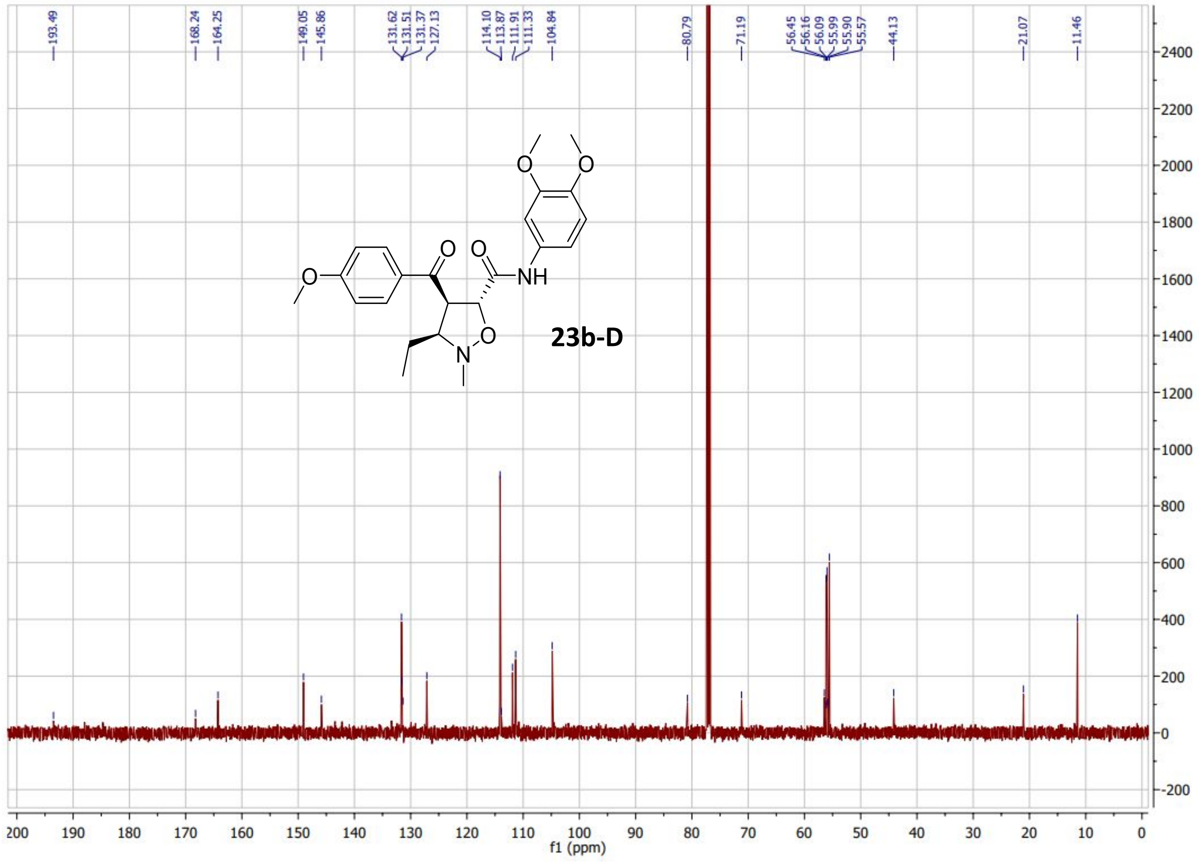

2.5.5. Transformations: Reductions

^{1}H and ^{13}C NMR spectra of 4-Oxo-*N*,4-diphenylbutanamide (24a)

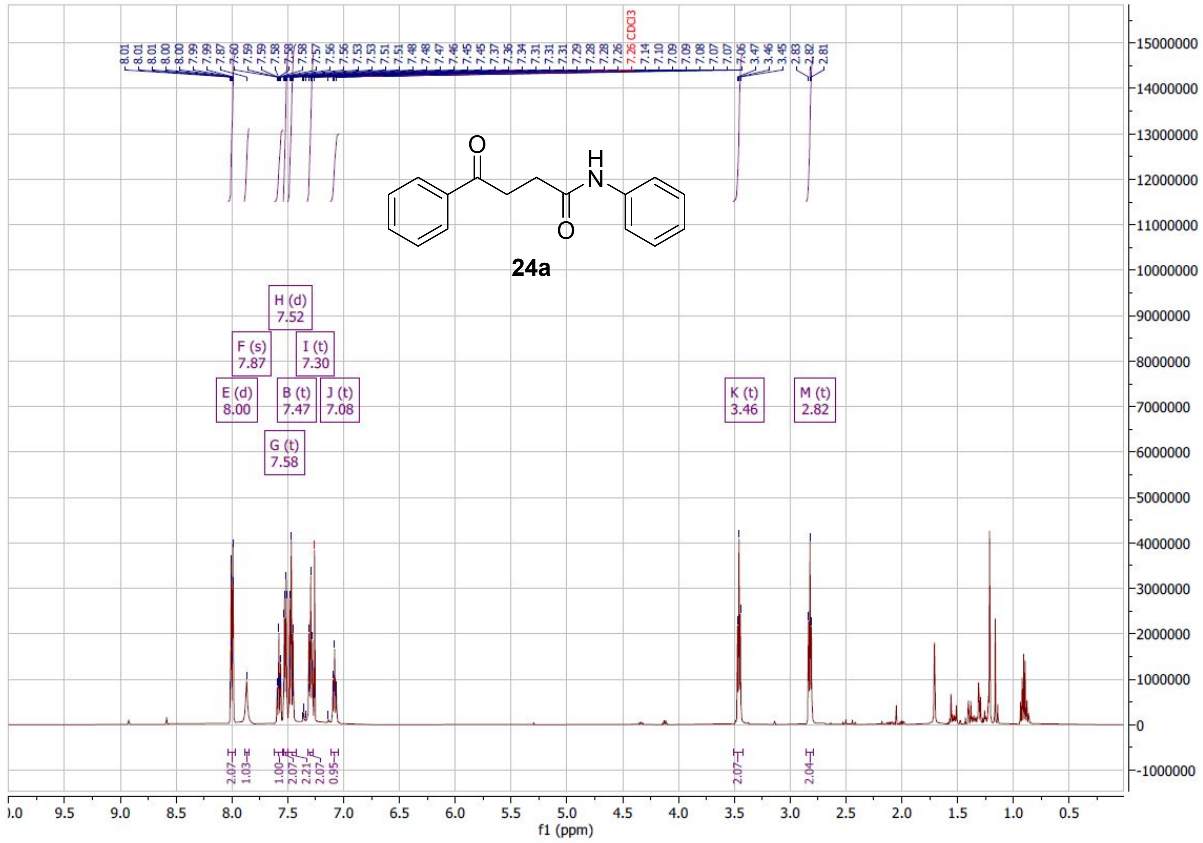

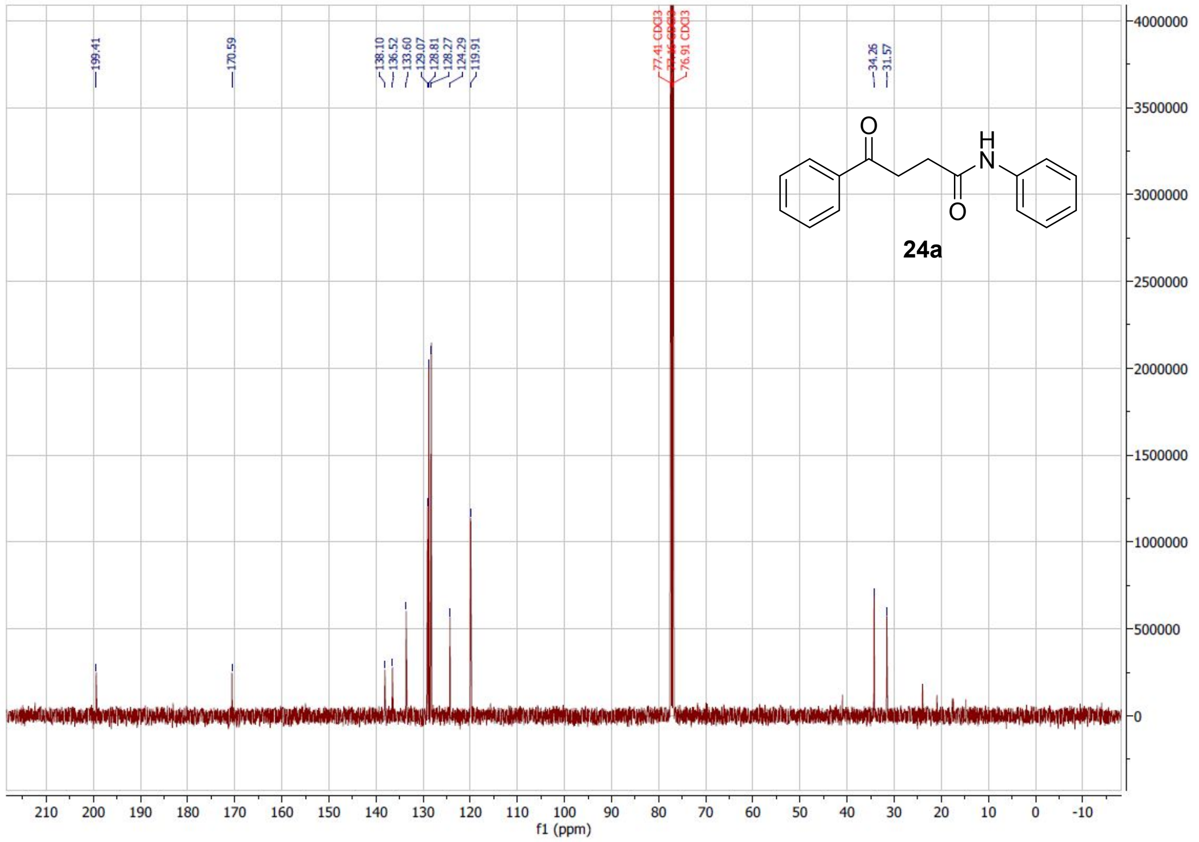

^{1}H and ^{13}C NMR spectra of *N*-(3,4-dimethoxyphenyl)-4-(4-methoxyphenyl)-4-oxobutanamide (24b)

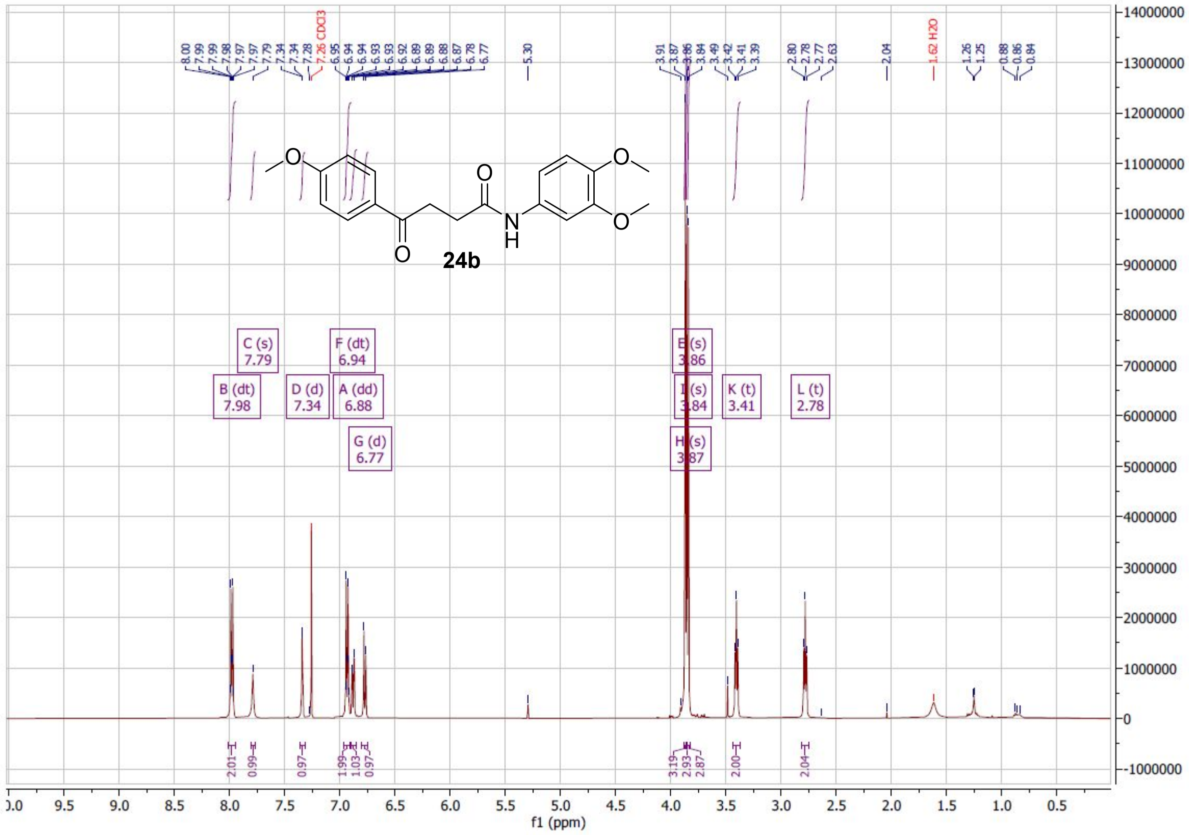

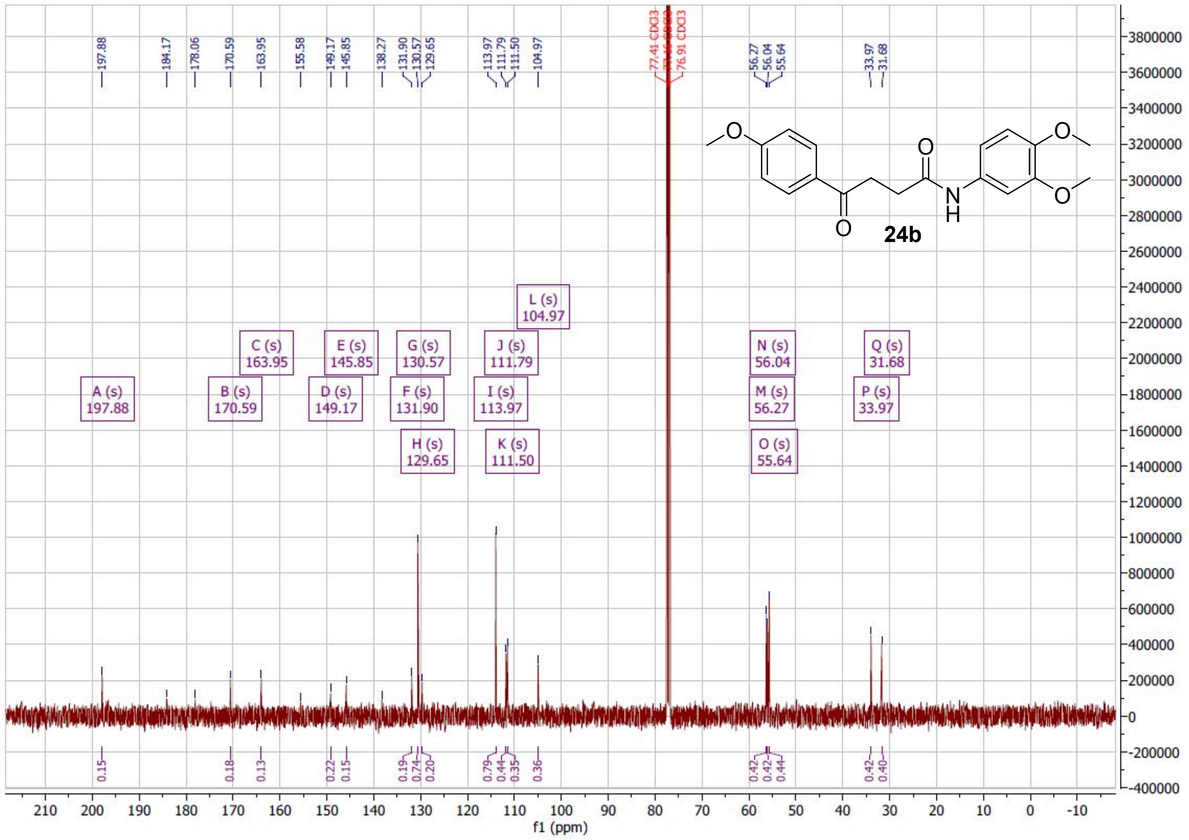

^{1}H and ^{13}C NMR spectra of (*E*)-4-hydroxy-*N*,4-diphenylbut-2-enamide (25a)

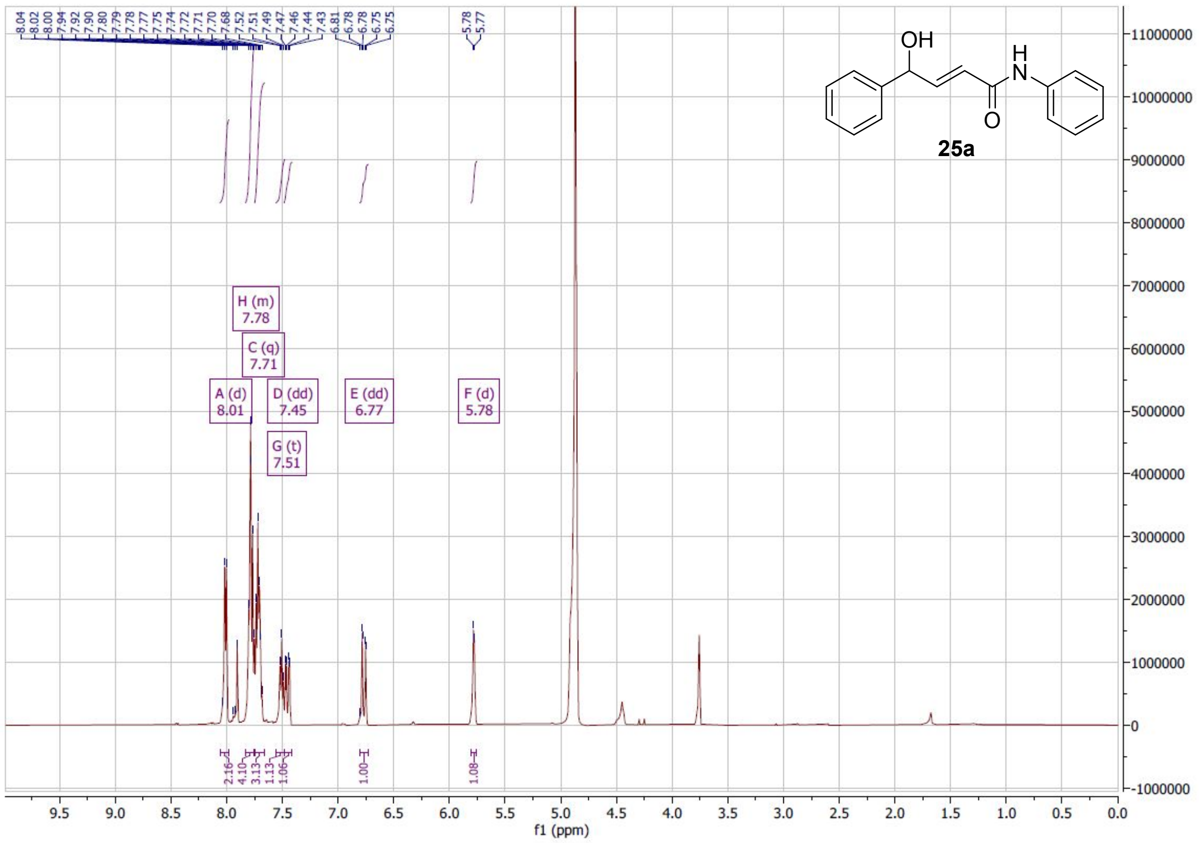

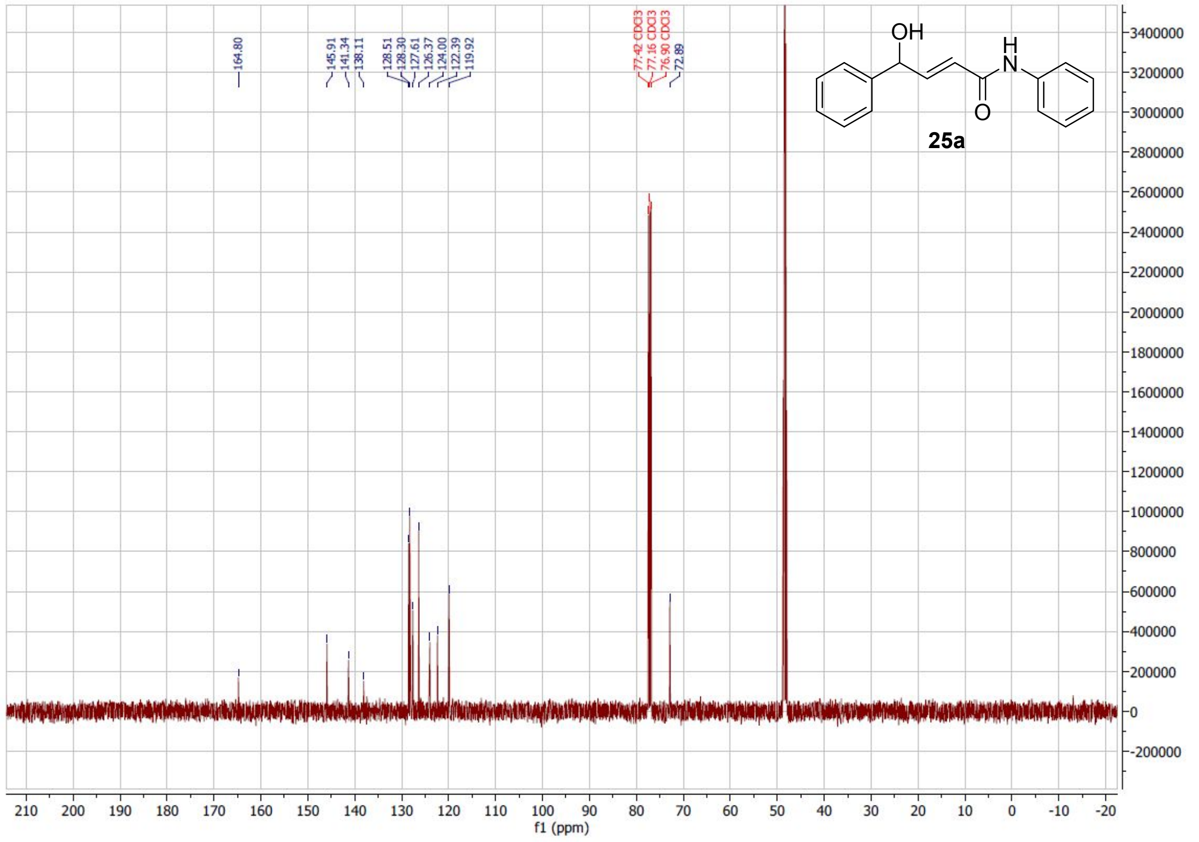

^{1}H and ^{13}C NMR spectra of *(E)-N*-(3,4-dimethoxyphenyl)-4-hydroxy-4-(4-methoxyphenyl)but-2-enamide (25b)

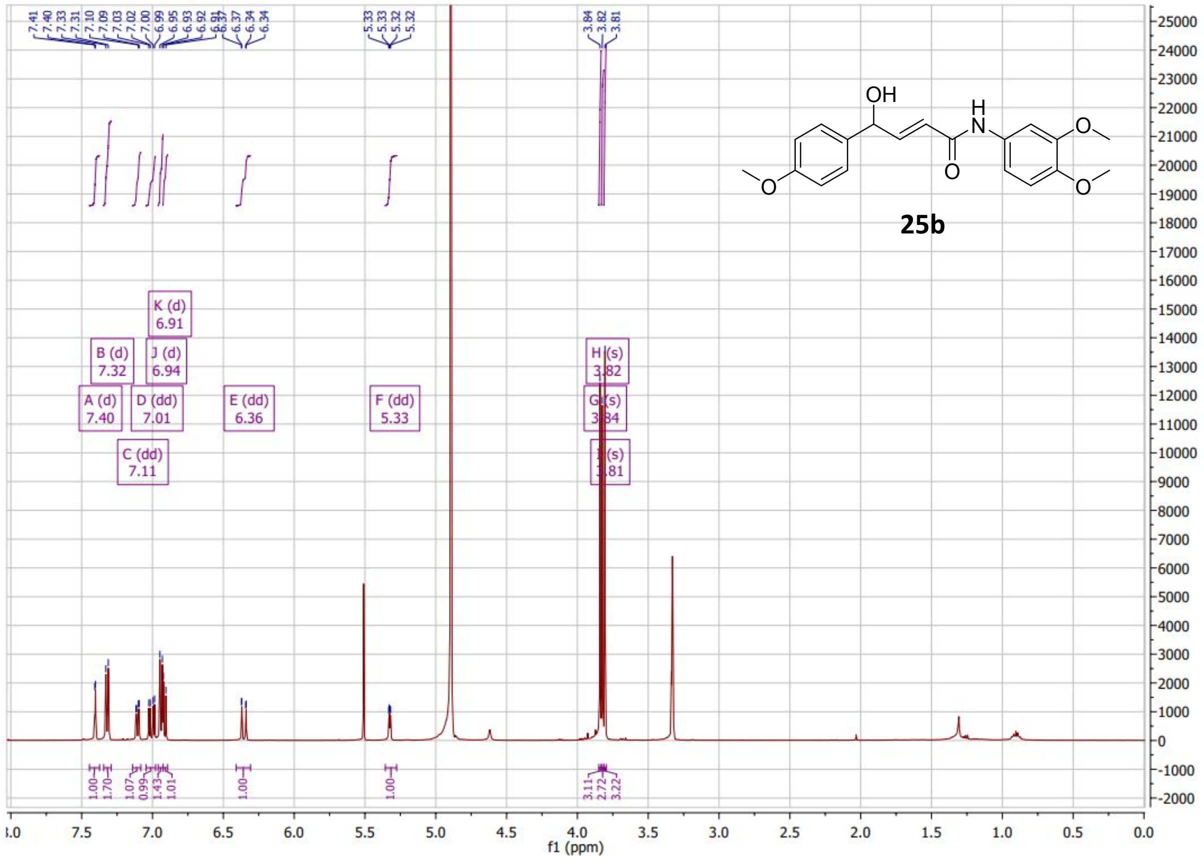

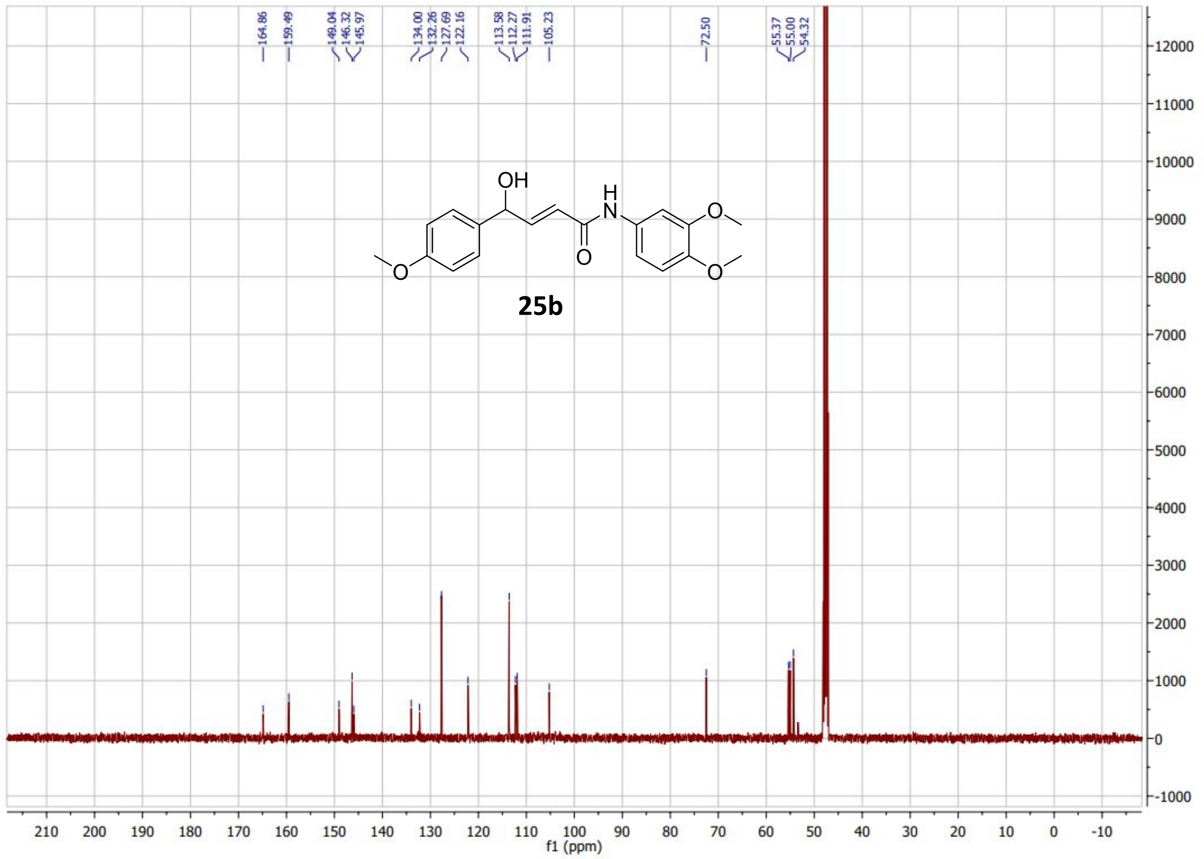

^{1}H and ^{13}C NMR spectra of 4-Hydroxy-*N*,4-diphenylbutanamide (26a)

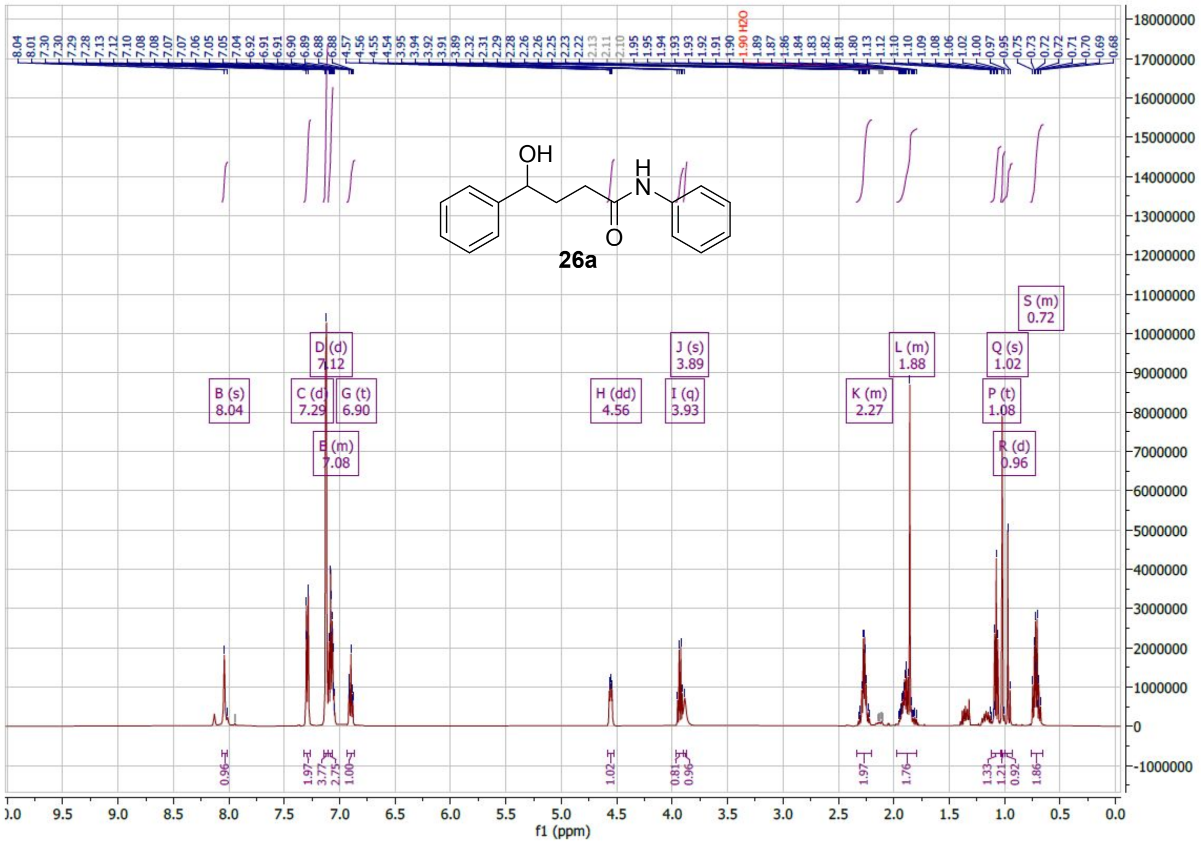

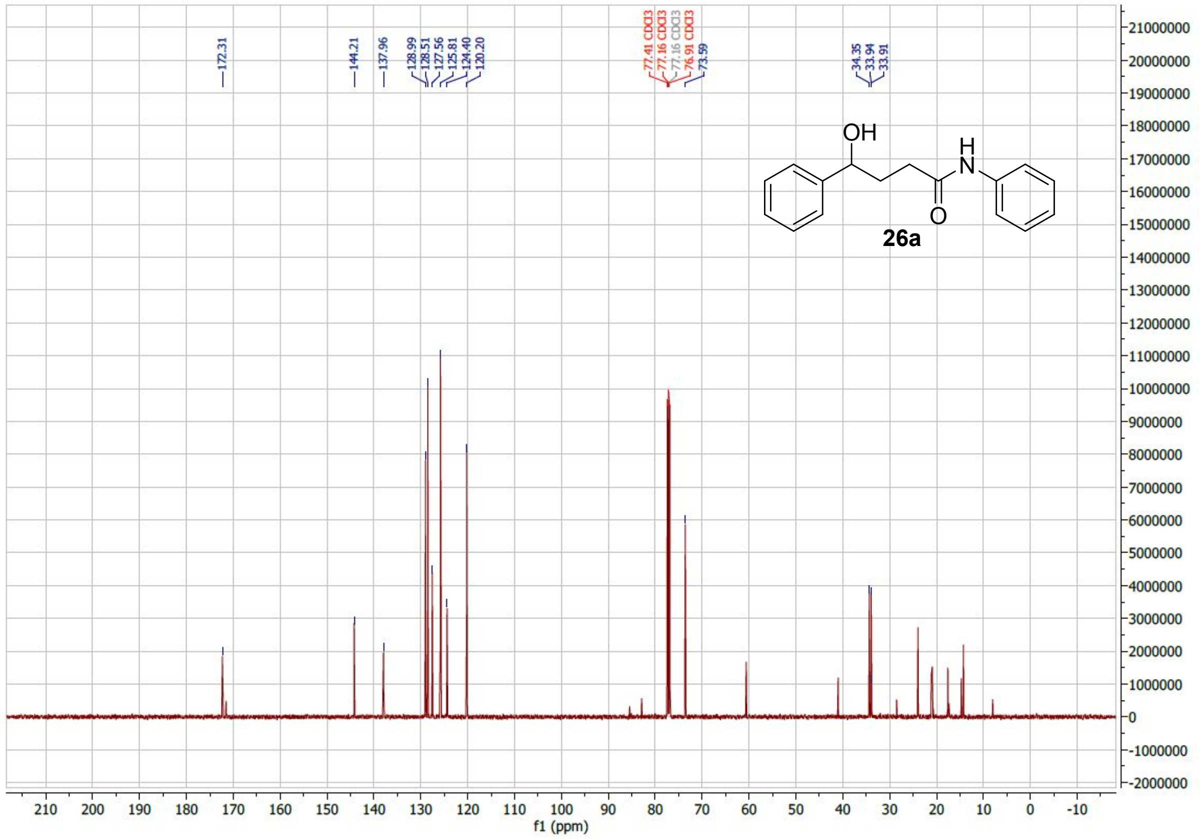

¹H and ¹³C NMR spectra of *N*-(3,4-dimethoxyphenyl)-4-hydroxy-4-(4-methoxyphenyl)butanamide (26b)

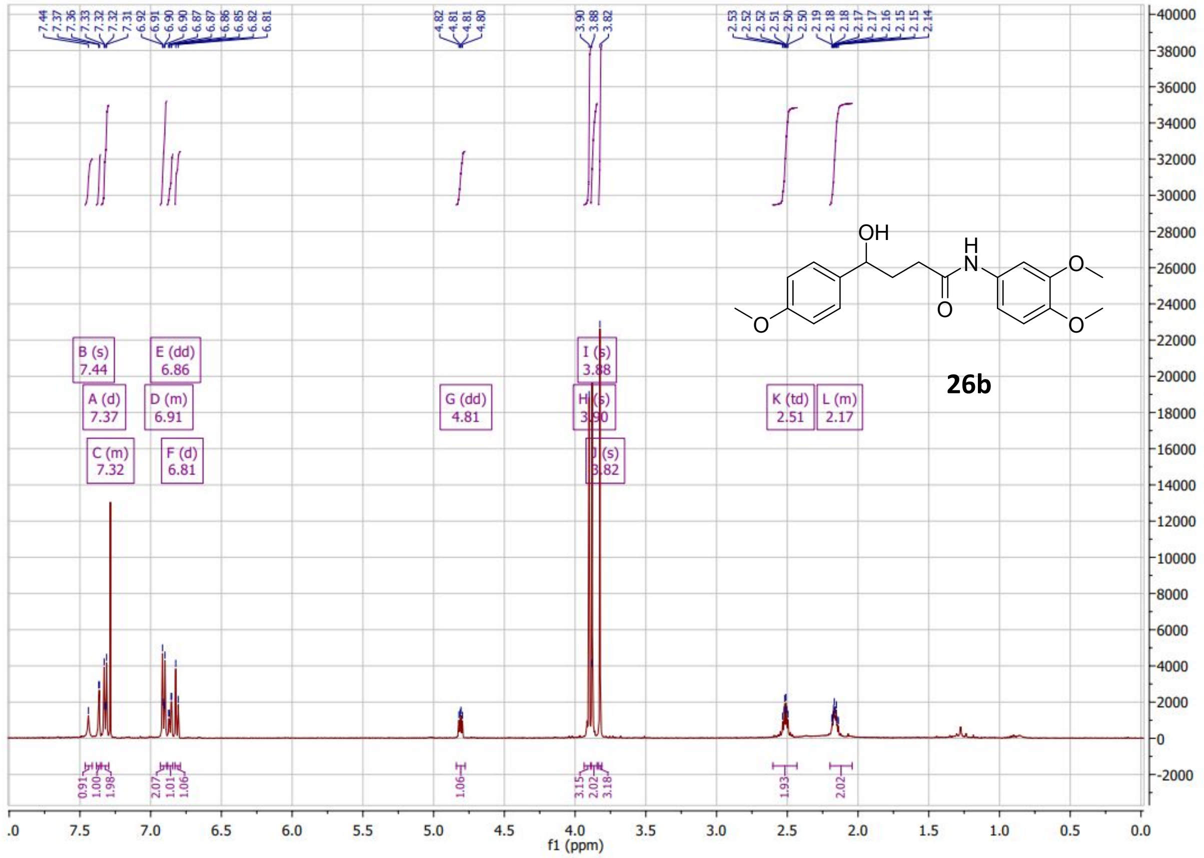

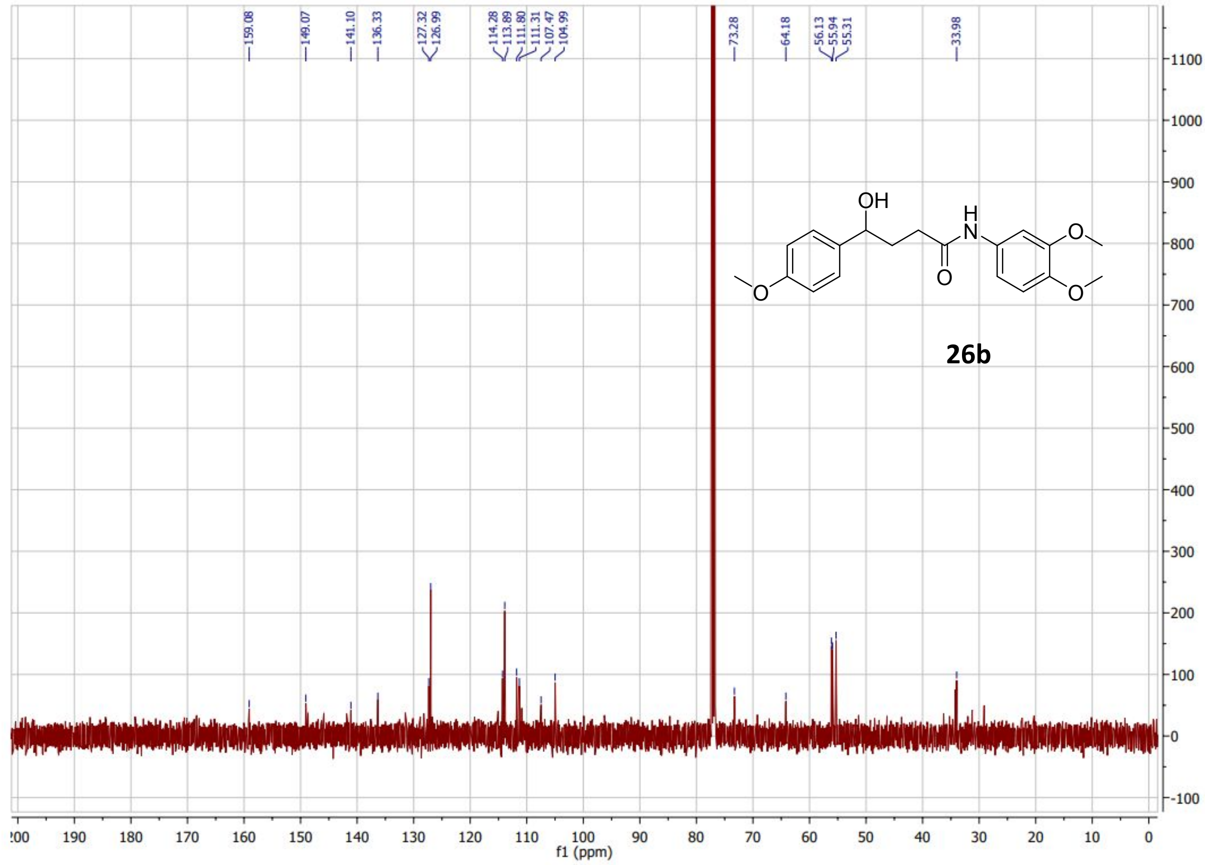

2.5.6. Transformations: 1,4-Additions

^{1}H and ^{13}C NMR spectra of 4-Oxo-*N*,4-diphenyl-2-(pyrrolidin-1-yl)butanamide (27a)

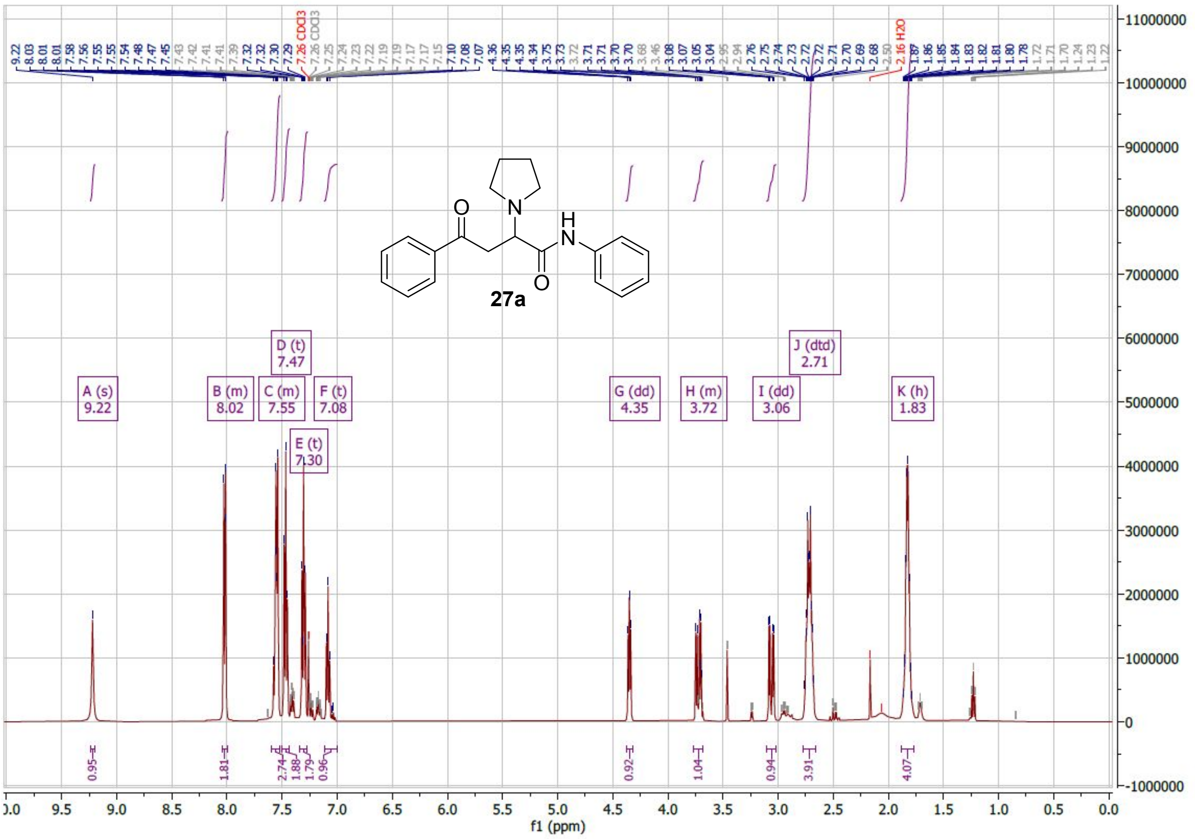

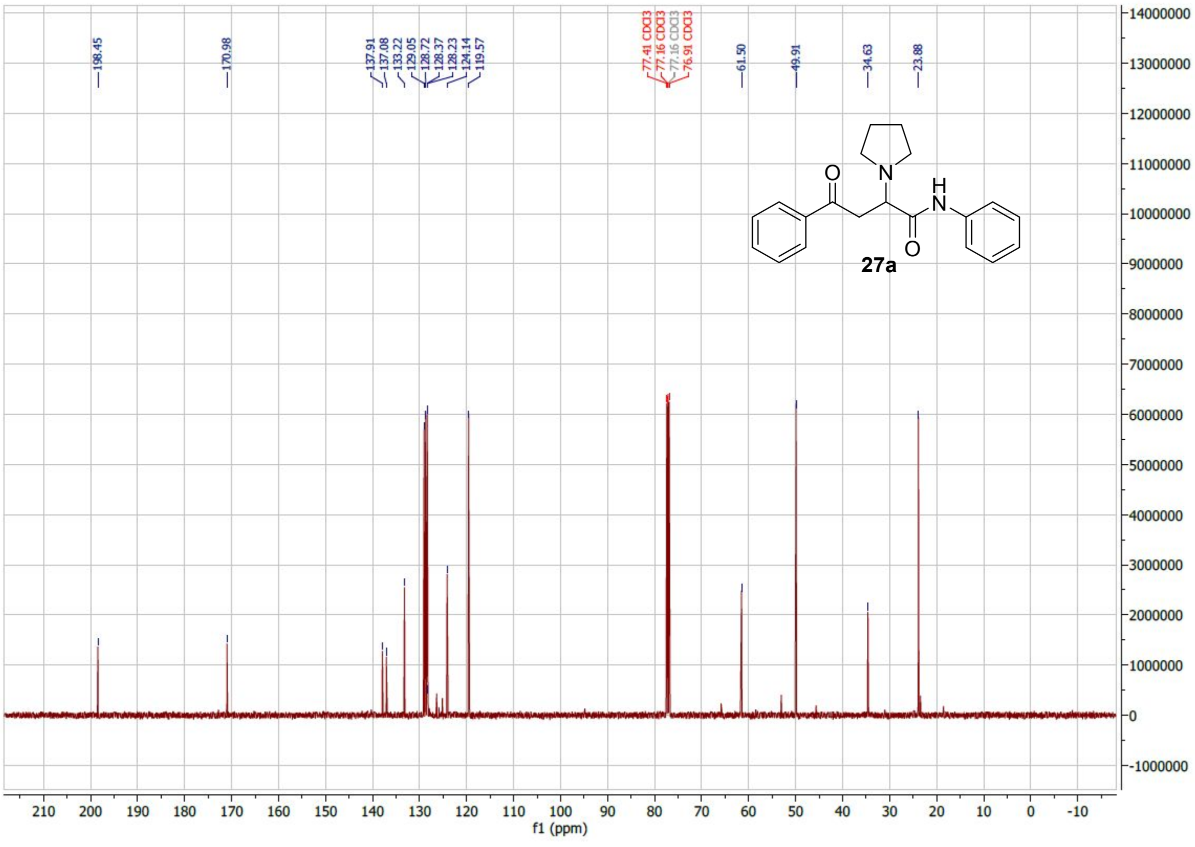

^{1}H and ^{13}C NMR spectra of *N*-(3,4-dimethoxyphenyl)-4-(4-methoxyphenyl)-4-oxo-2-(pyrrolidin-1-yl)butanamide (27b)

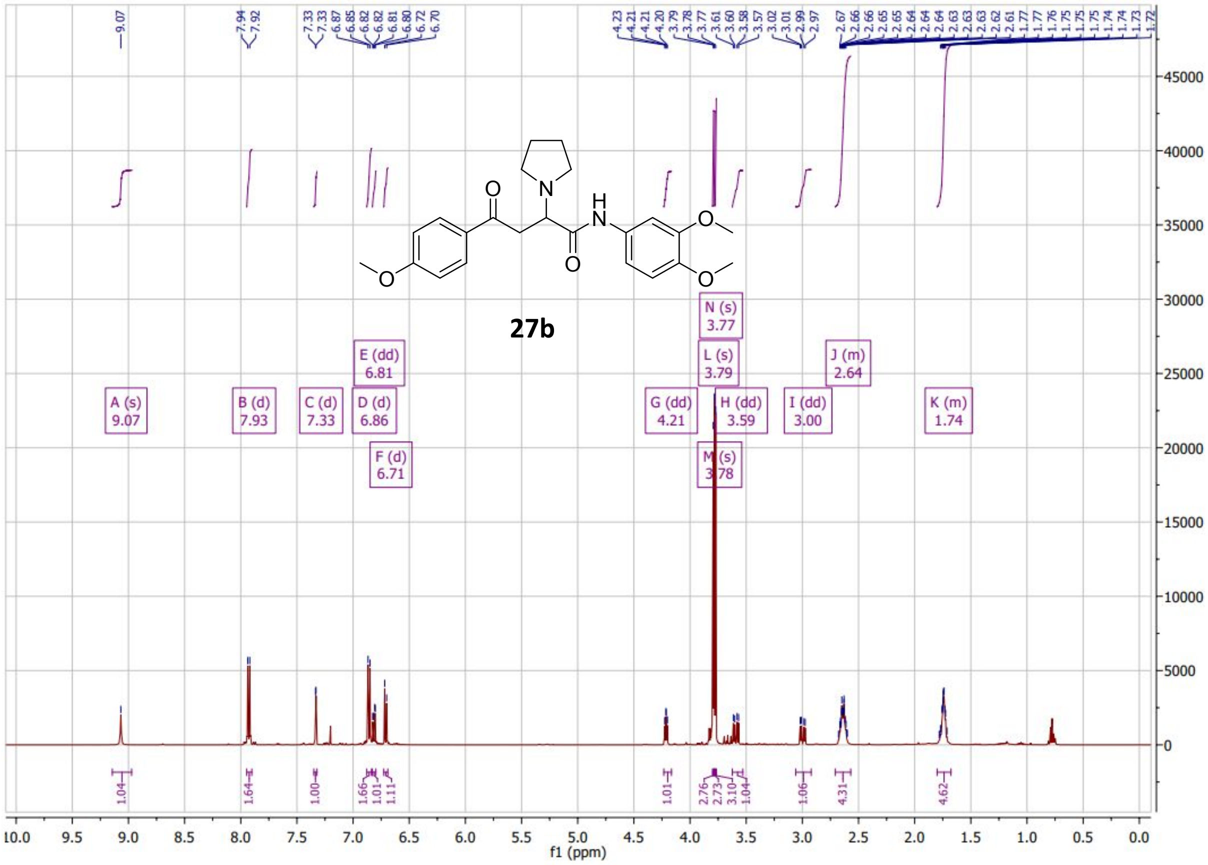

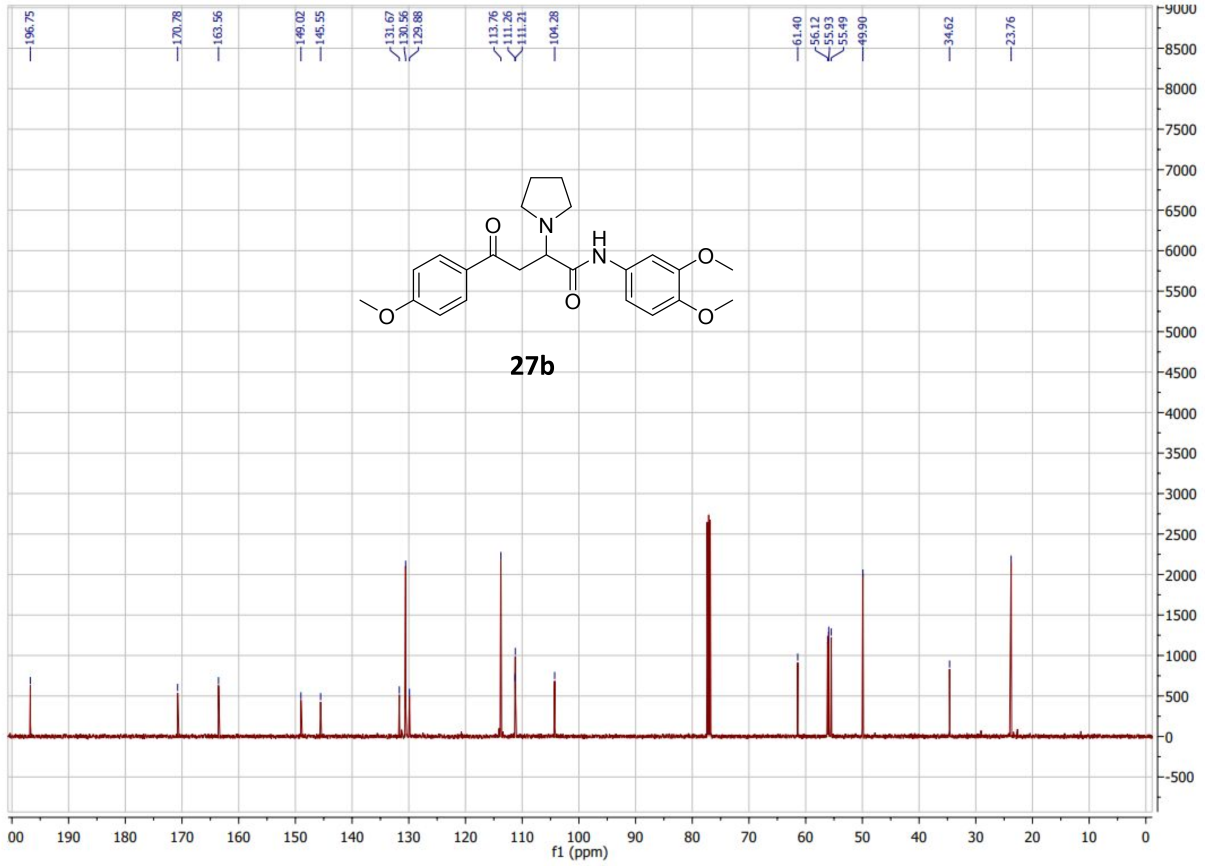

3. Scope Evaluation of the DOS Transformations

3.1 Analysis of Starting Materials

3.1.1. HPLC Analyses of Starting Materials of Set 1 Before Being Pooled

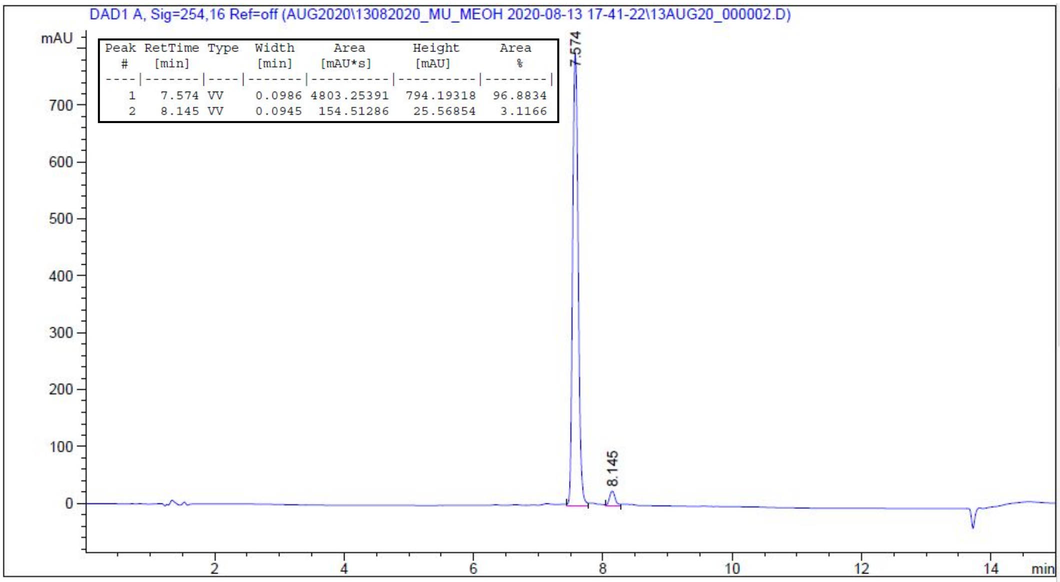

*HPLC chromatogram of **1c** in acidic conditions.*

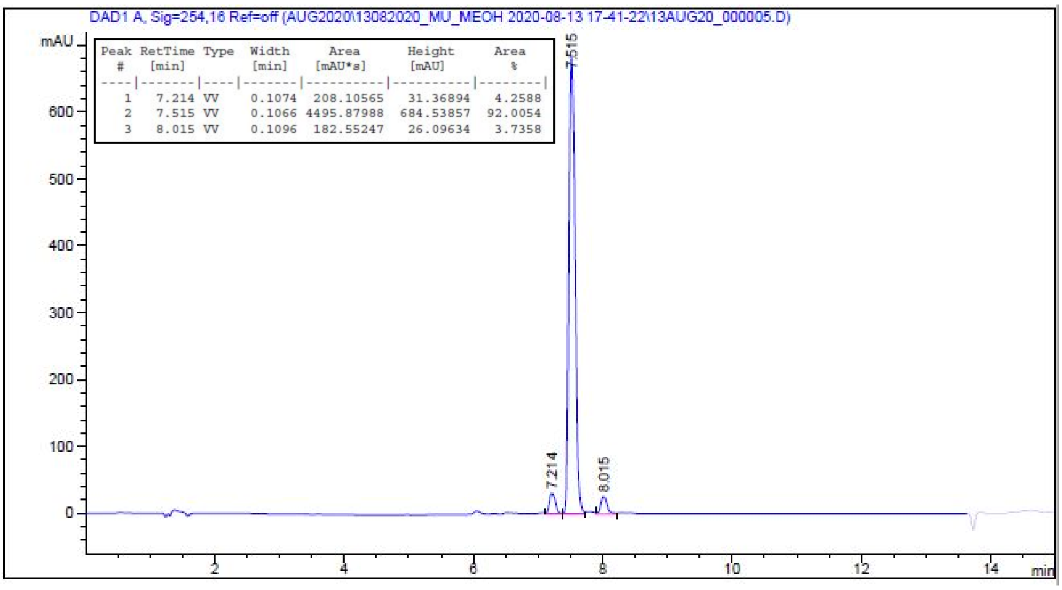

*HPLC chromatogram of **1c** in basic conditions.*

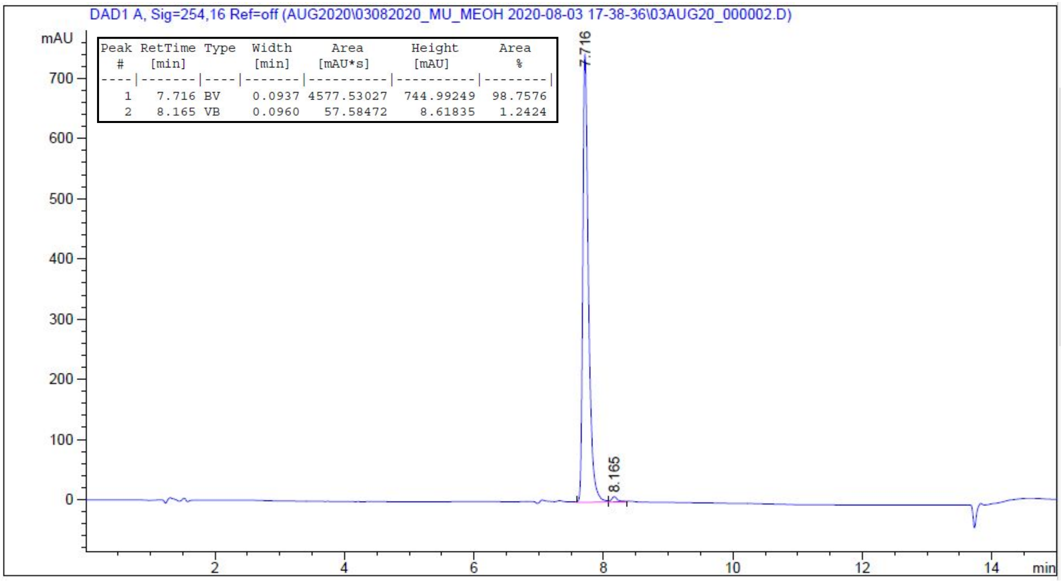

*HPLC chromatogram of **1f** in acidic conditions.*

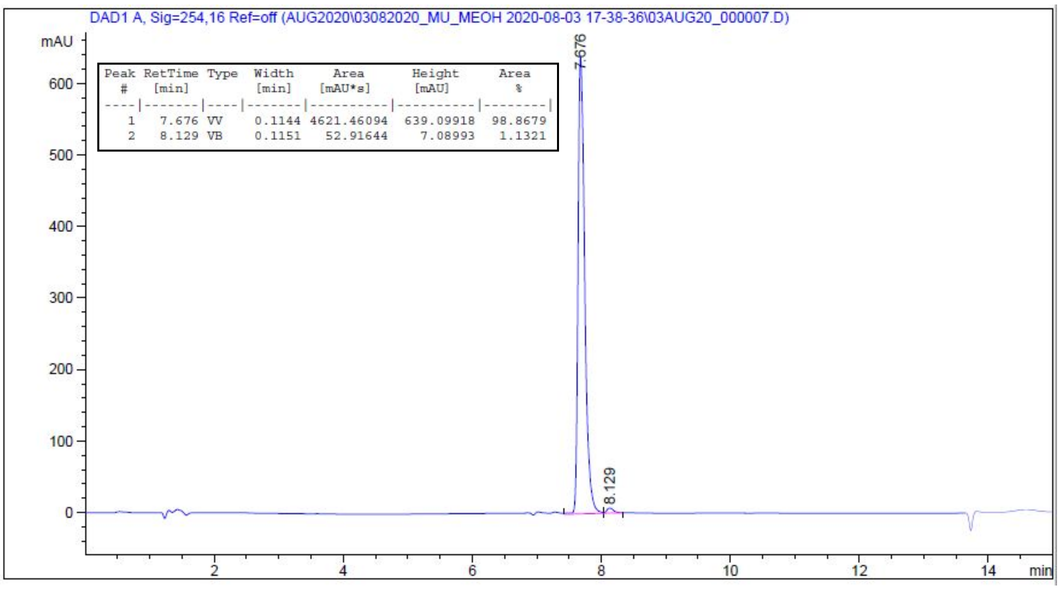

*HPLC chromatogram of **1f** in basic conditions.*

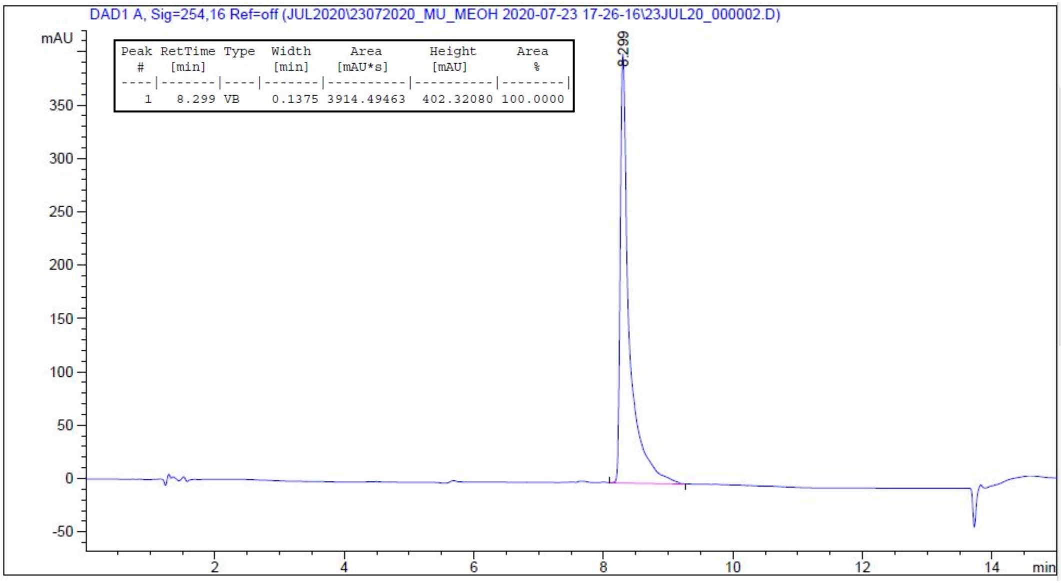

*HPLC chromatogram of **1d** in acidic conditions.*

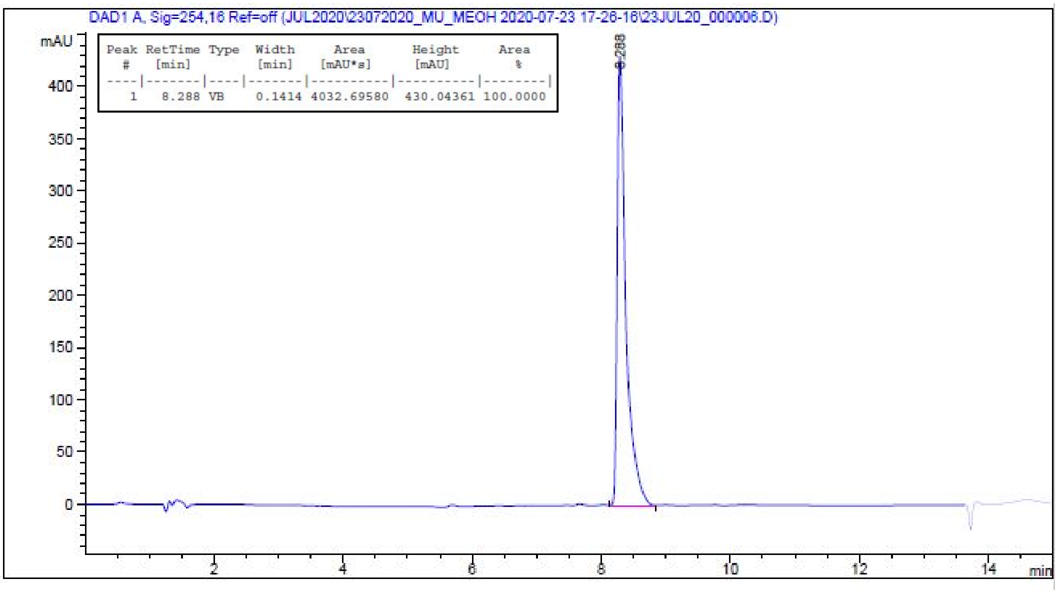

*HPLC chromatogram of **1d** in basic conditions.*

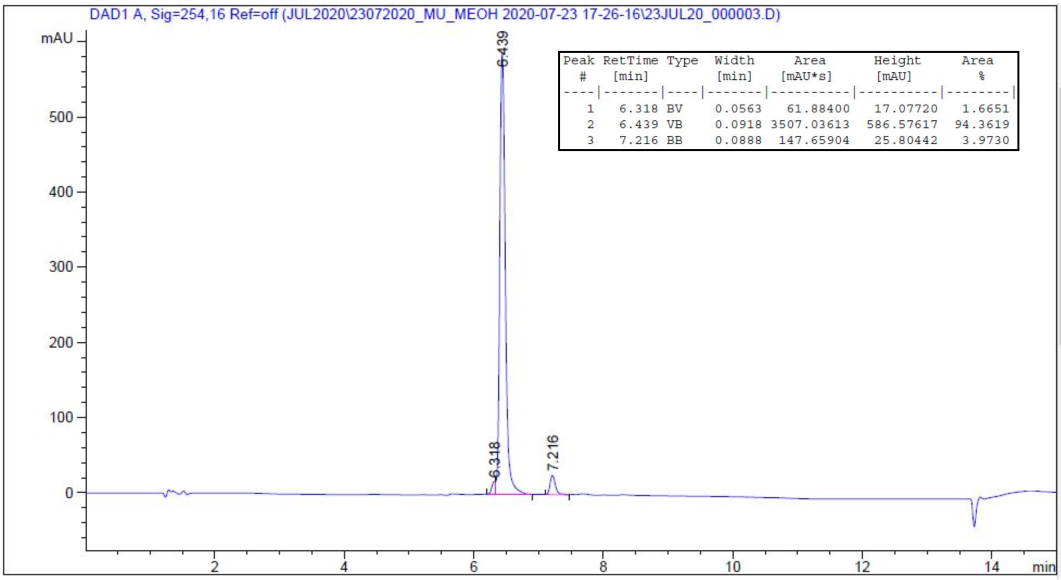

*HPLC chromatogram of **1e** in acidic conditions.*

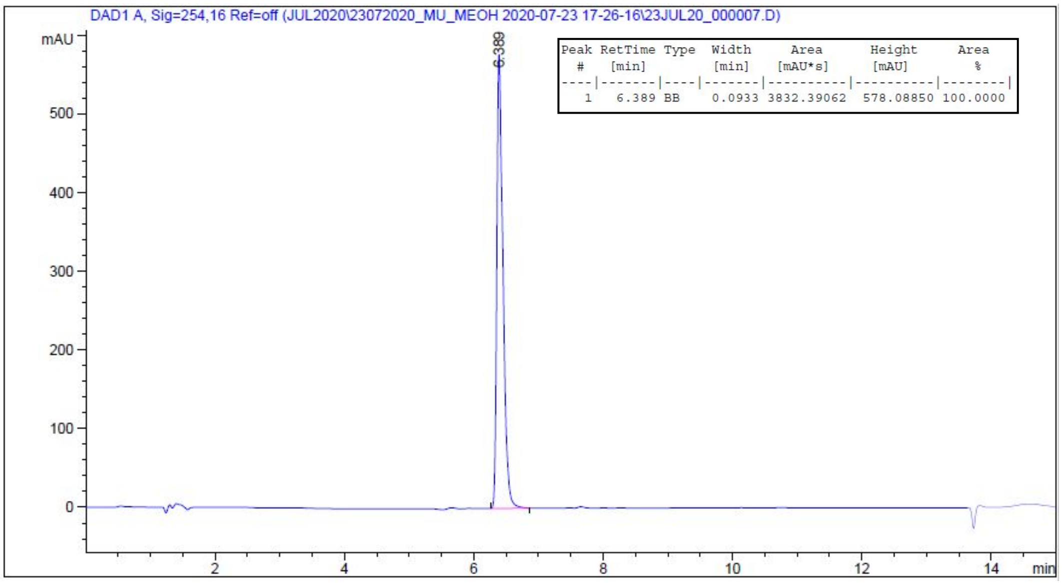

*HPLC chromatogram of **1e** in basic conditions.*

3.1.2. HPLC Analyses of Set 1 After Being Pooled

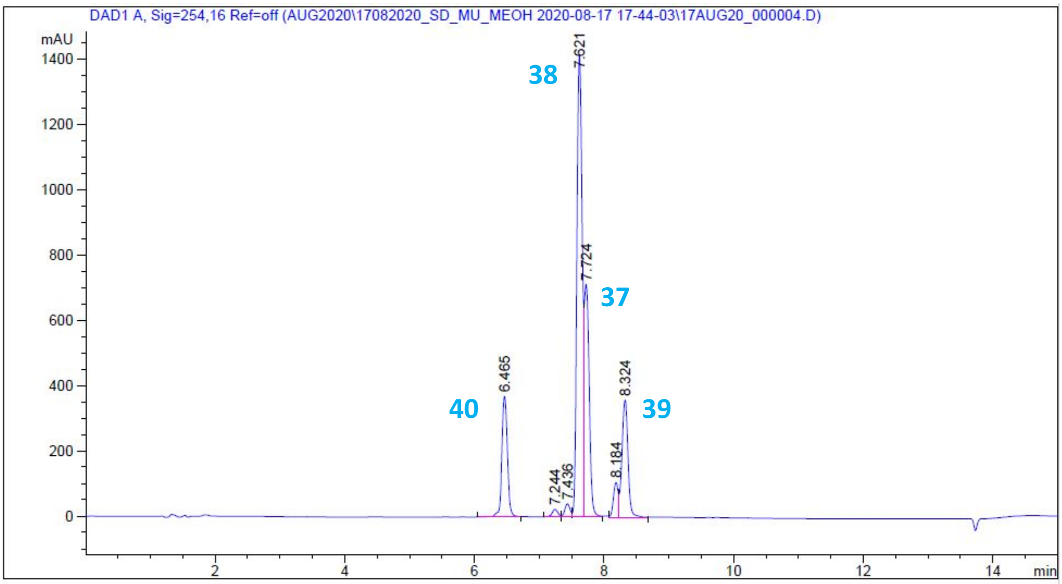

HPLC chromatogram of pooled Set 1 in acidic conditions.

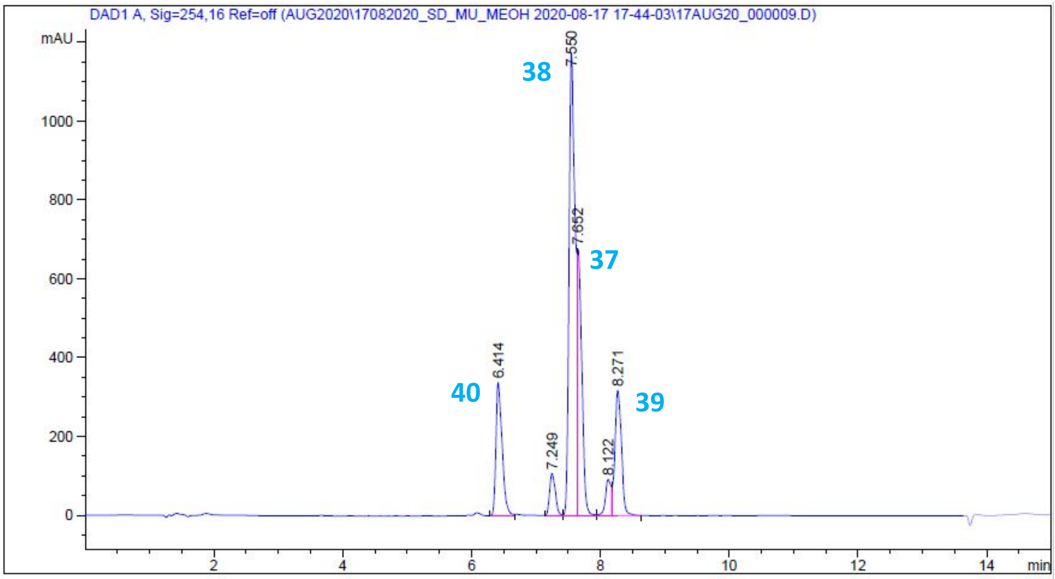

HPLC chromatogram of pooled Set 1 in basic conditions.

3.1.3. HPLC Analyses of Starting Materials of Set 2 Before Being Pooled.

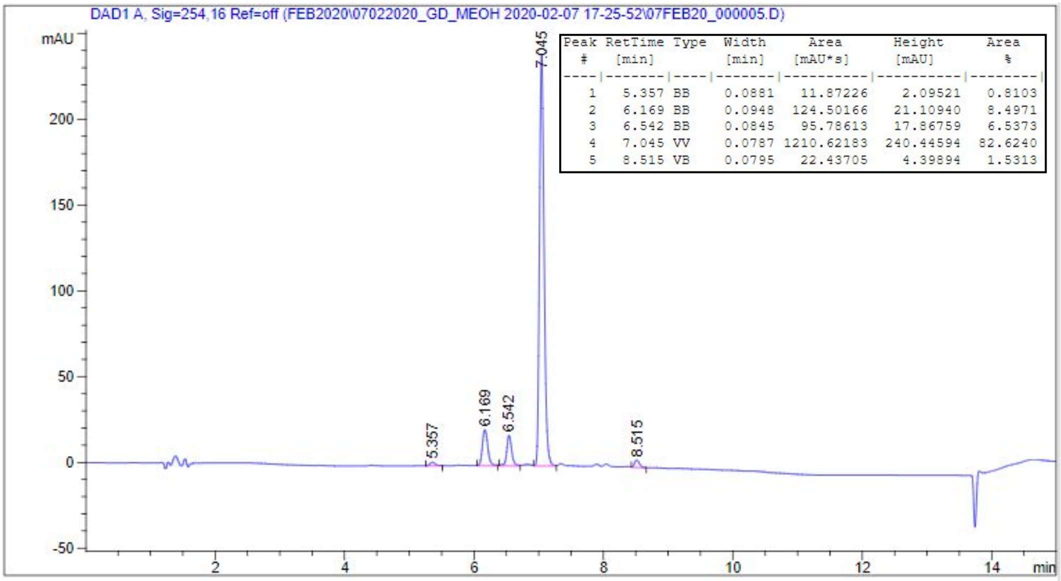

Peak #	RetTime [min]	Type	Width [min]	Area [mAU*s]	Height [mAU]	Area %
1	5.357	BB	0.0881	11.87226	2.09521	0.8103
2	6.169	BB	0.0948	124.50166	21.10940	8.4971
3	6.542	BB	0.0845	95.78613	17.86759	6.5373
4	7.045	VV	0.0787	1210.62183	240.44594	82.6240
5	8.515	VB	0.0795	22.43705	4.39894	1.5313

*HPLC chromatogram of **1h** in acidic conditions.*

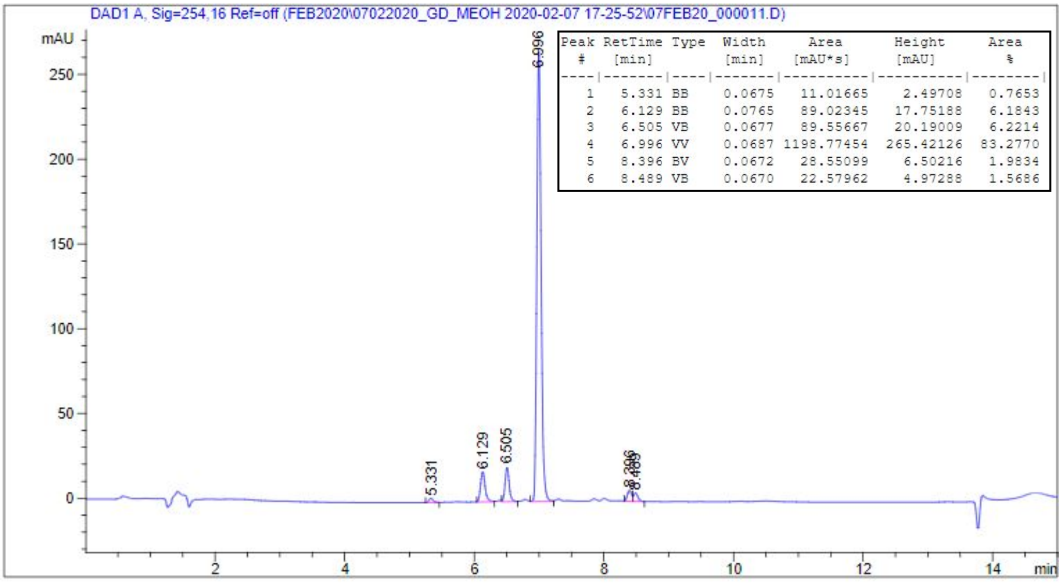

Peak #	RetTime [min]	Type	Width [min]	Area [mAU*s]	Height [mAU]	Area %
1	5.331	BB	0.0675	11.01665	2.49708	0.7653
2	6.129	BB	0.0765	89.02345	17.75188	6.1843
3	6.505	VB	0.0677	89.55667	20.19009	6.2214
4	6.996	VV	0.0687	1198.77454	265.42126	83.2770
5	8.396	BV	0.0672	28.55099	6.50216	1.9834
6	8.489	VB	0.0670	22.57962	4.97288	1.5686

*HPLC chromatogram of **1h** in basic conditions.*

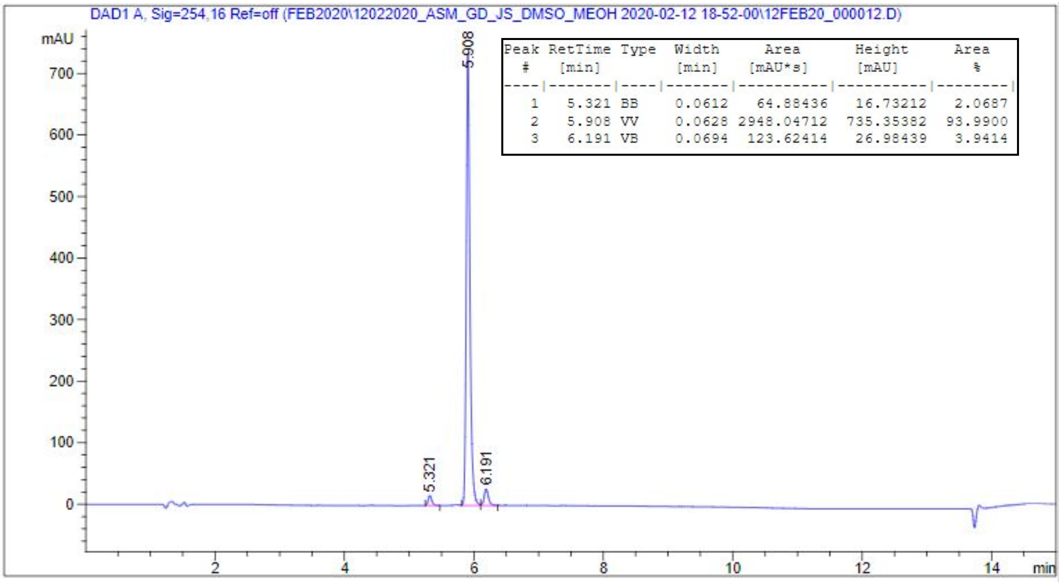

*HPLC chromatogram of **1i** in acidic conditions.*

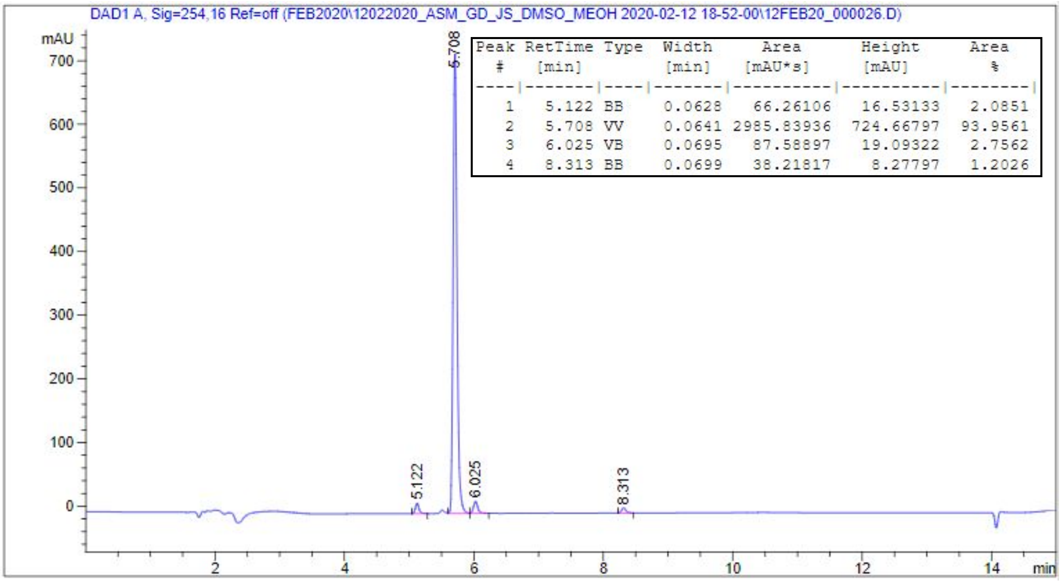

*HPLC chromatogram of **1i** in basic conditions.*

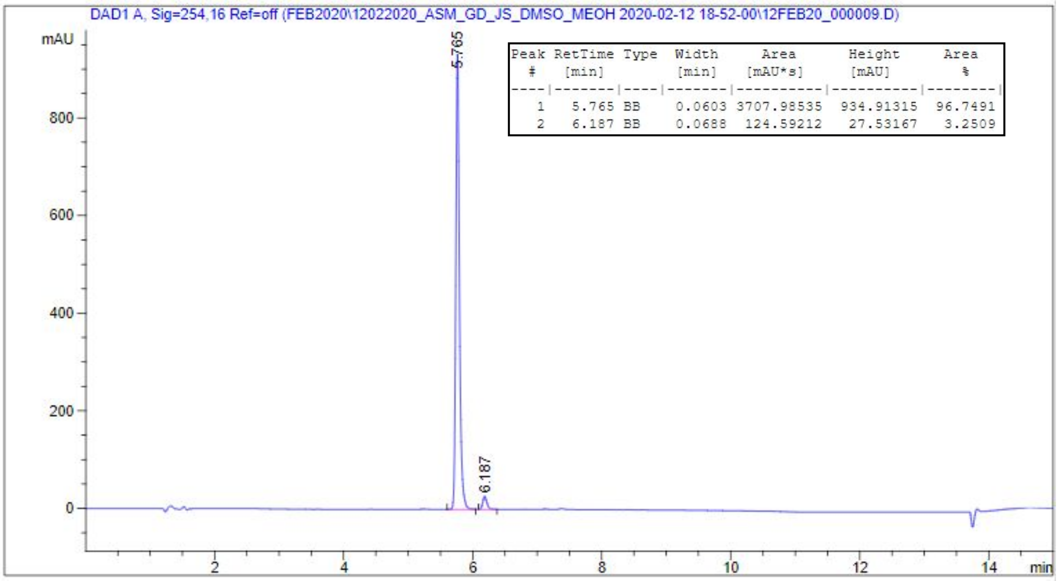

*HPLC chromatogram of **1j** in acidic conditions.*

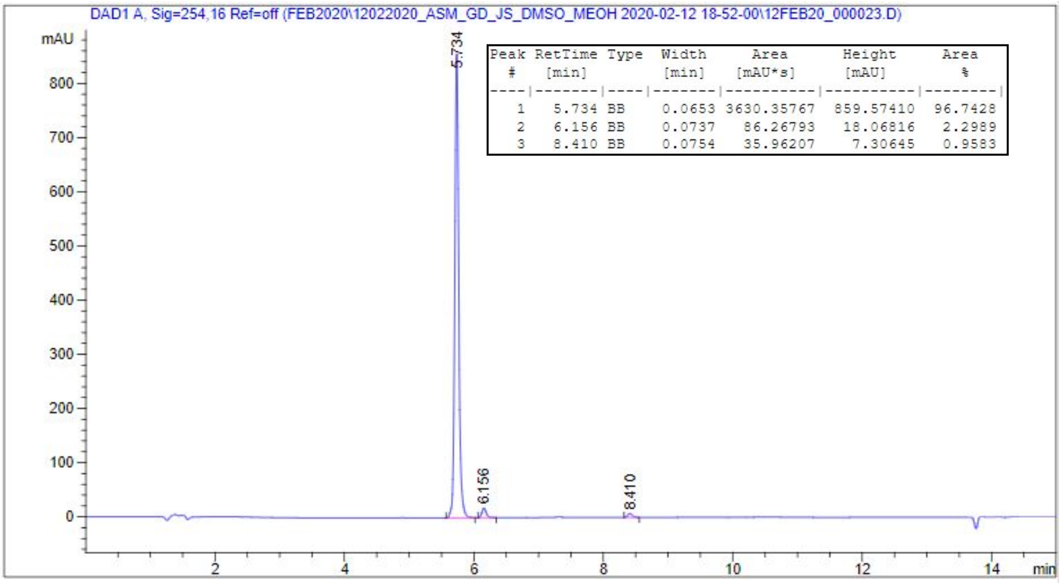

*HPLC chromatogram of **1j** in basic conditions.*

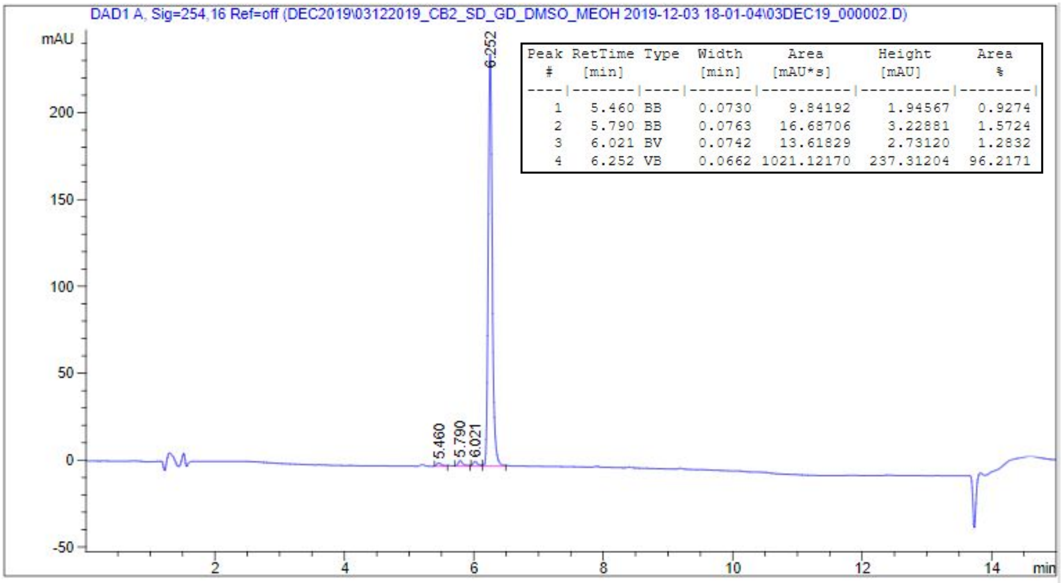

Peak #	RetTime [min]	Type	Width [min]	Area [mAU*s]	Height [mAU]	Area %
1	5.460	BB	0.0730	9.84192	1.94567	0.9274
2	5.790	BB	0.0763	16.68706	3.22881	1.5724
3	6.021	BV	0.0742	13.61829	2.73120	1.2832
4	6.252	VB	0.0662	1021.12170	237.31204	96.2171

*HPLC chromatogram of **1k** in acidic conditions.*

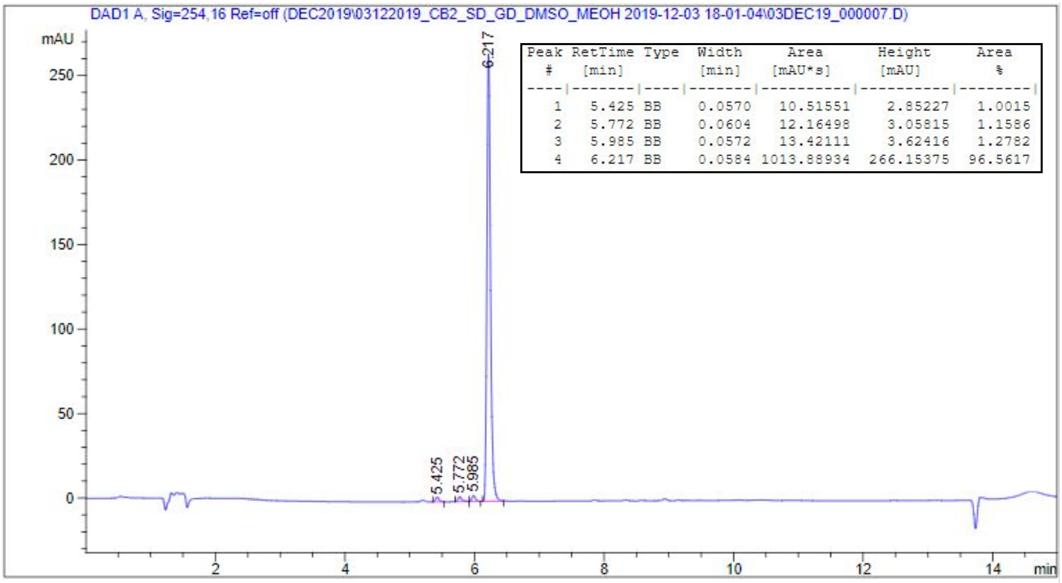

Peak #	RetTime [min]	Type	Width [min]	Area [mAU*s]	Height [mAU]	Area %
1	5.425	BB	0.0570	10.51551	2.85227	1.0015
2	5.772	BB	0.0604	12.16498	3.05815	1.1586
3	5.985	BB	0.0572	13.42111	3.62416	1.2782
4	6.217	BB	0.0584	1013.88934	266.15375	96.5617

*HPLC chromatogram of **1k** in basic conditions.*

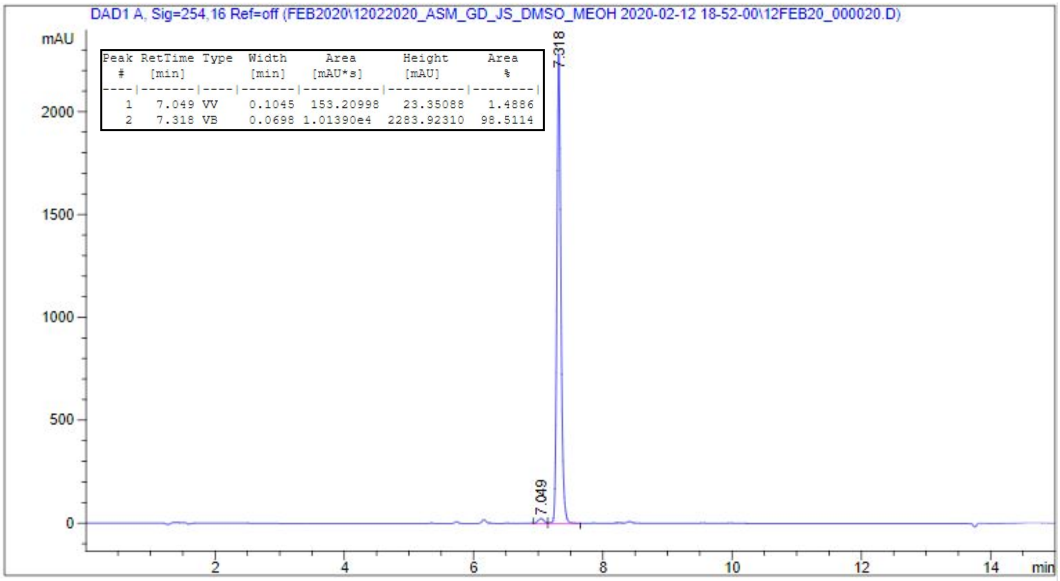

Peak #	RetTime [min]	Type	Width [min]	Area [mAU*s]	Height [mAU]	Area %
1	7.049	VV	0.1045	153.20998	23.35088	1.4886
2	7.318	VB	0.0698	1.01390e4	2283.92310	98.5114

HPLC chromatogram of ***1l*** *in acidic conditions.*

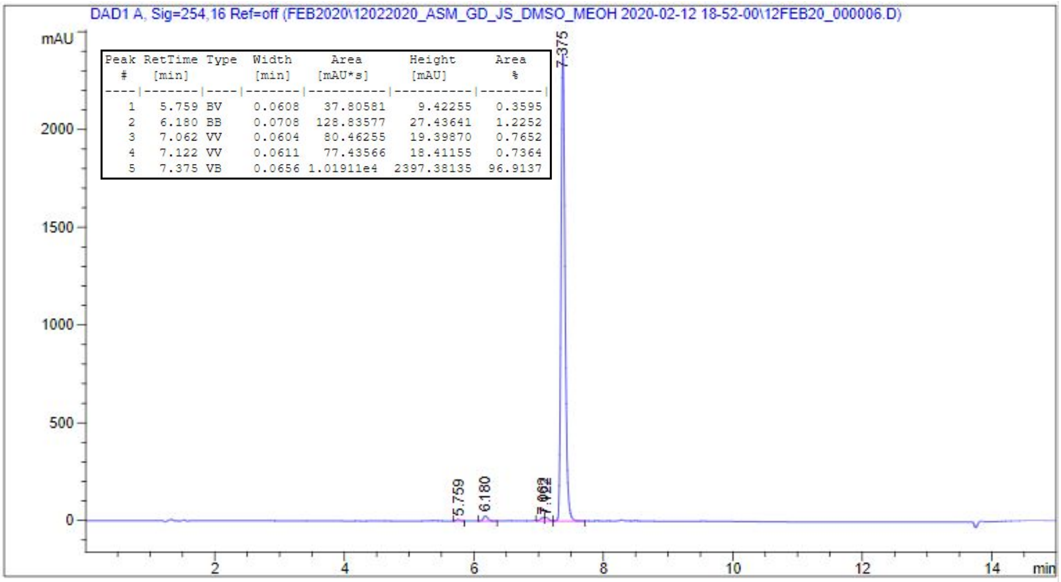

Peak #	RetTime [min]	Type	Width [min]	Area [mAU*s]	Height [mAU]	Area %
1	5.759	BV	0.0608	37.80581	9.42255	0.3595
2	6.180	BB	0.0708	128.83577	27.43641	1.2252
3	7.062	VV	0.0604	80.46255	19.39870	0.7652
4	7.122	VV	0.0611	77.43566	18.41155	0.7364
5	7.375	VB	0.0656	1.01911e4	2397.38135	96.9137

HPLC chromatogram of ***1l*** *in basic conditions.*

3.2. HRMS and HPLC Analysis of DOS transformation on Pools 1 & 2

3.2.1. Transformations: 1,4-Cyclisations:

HRMS Analysis of Set 1 After Pyrazole Formation

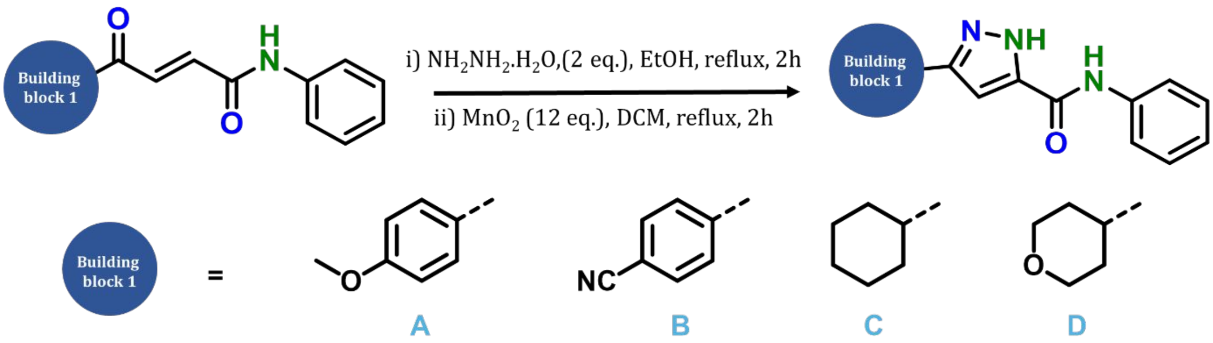

Product	Formula	Calculated mass $[M + H]^+$	Mass found $[M + H]^+$
A	$C_{17}H_{15}N_3O_2$	294.1240	294.1188
B	$C_{17}H_{12}N_4O$	289.1087	289.1099
C	$C_{16}H_{19}N_3O$	270.1604	270.1597
D	$C_{15}H_{17}N_3O_2$	272.1396	272.1402

HRMS Analysis of Set 2 After Pyrazole Formation

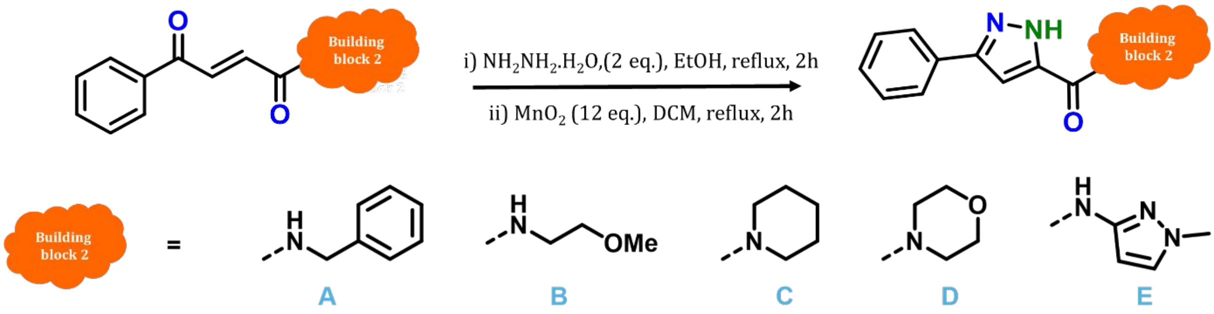

Product	Formula	Calculated mass $[M + H]^+$	Mass found $[M + H]^+$
A	$C_{17}H_{15}N_3O$	278.1291	278.1285
B	$C_{13}H_{15}N_3O_2$	246.1240	246.1229
C	$C_{15}H_{17}N_3O$	256.1447	256.1451
D	$C_{14}H_{15}N_3O_2$	258.1240	258.1229
E	$C_{14}H_{13}N_5O$	268.1196	268.1195

HPLC Analyses of Set 1 After the Pyrazole Formation Transformation

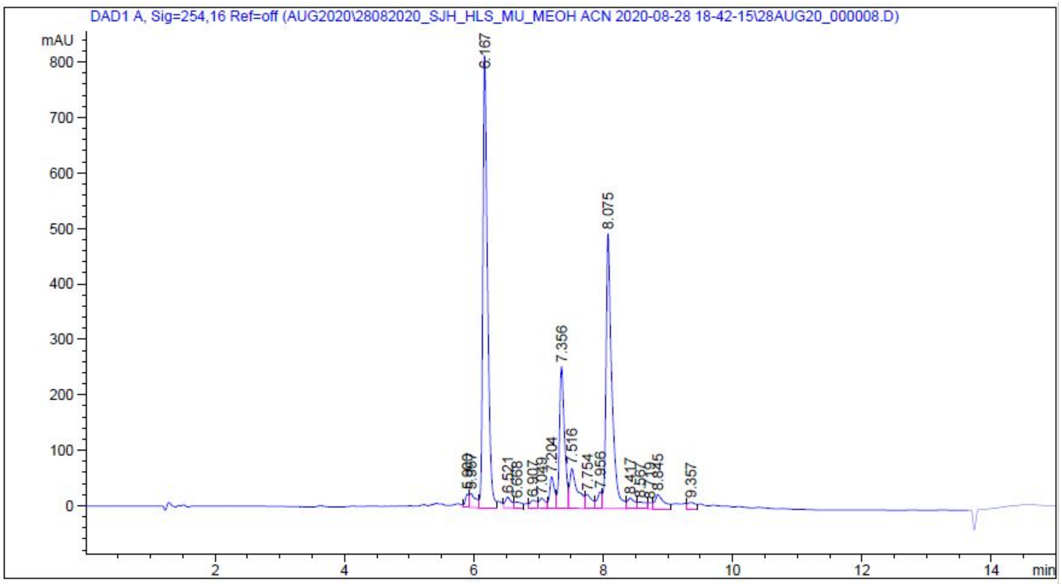

HPLC chromatogram of the resulting crude mixture after pyrazole formation applied to Set 1 in acidic conditions.

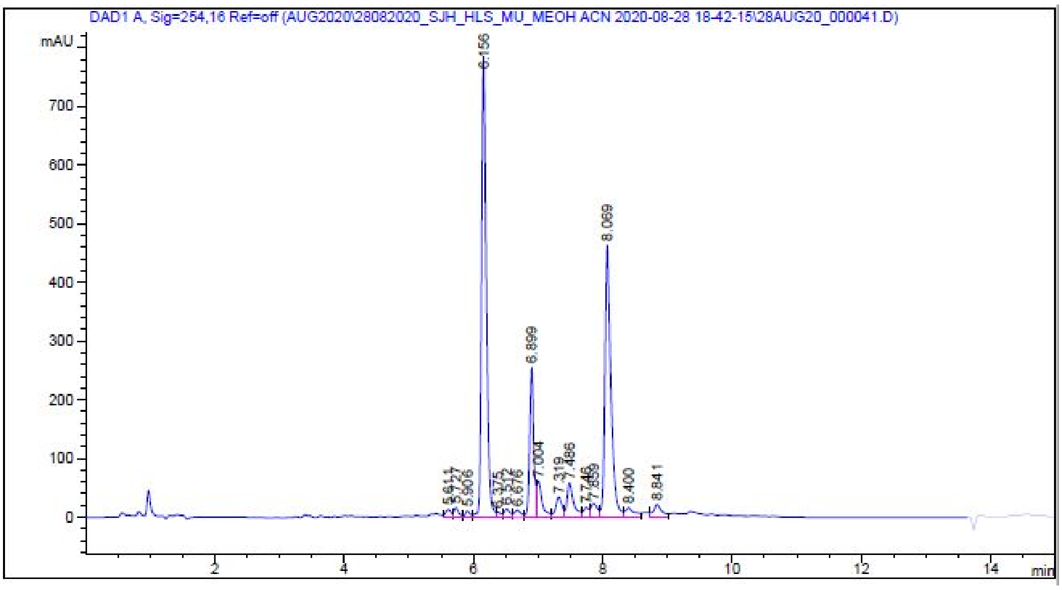

HPLC chromatogram of the resulting crude mixture after pyrazole formation applied to Set 1 in basic conditions.

HPLC Analyses of Set 2 After the Pyrazole Formation Transformation

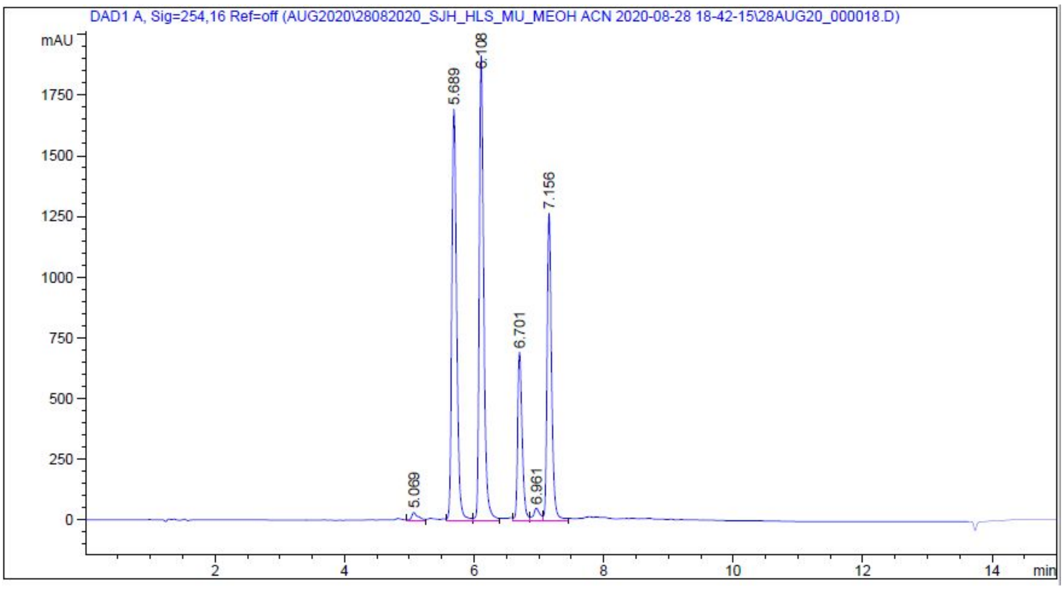

HPLC chromatogram of the resulting crude mixture after pyrazole formation applied to Set 2 in acidic conditions.

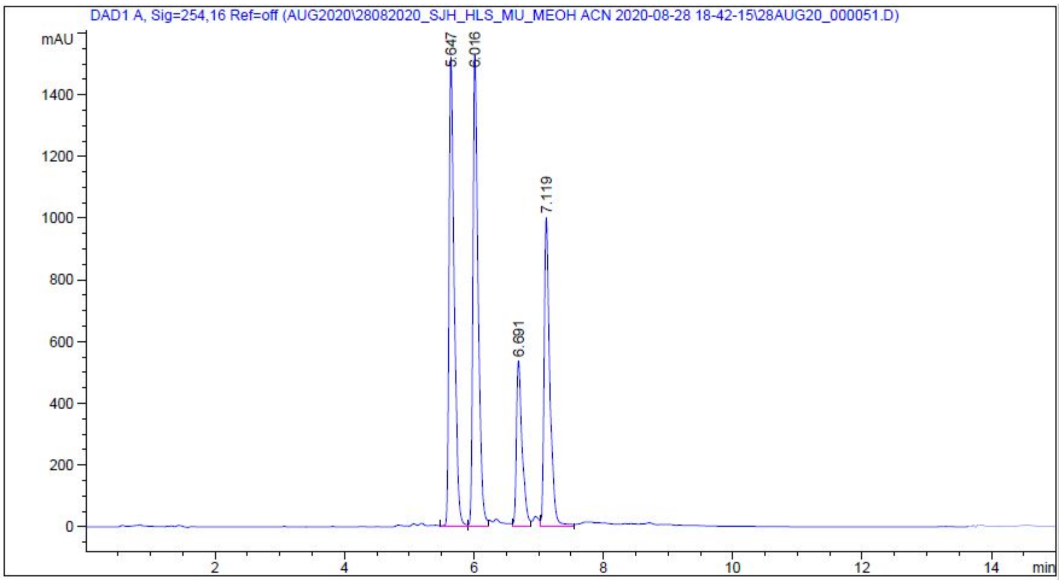

HPLC chromatogram of the resulting crude mixture after pyrazole formation applied to Set 2 in basic conditions.

HRMS Analysis of Set 1 After Isoxazole Formation

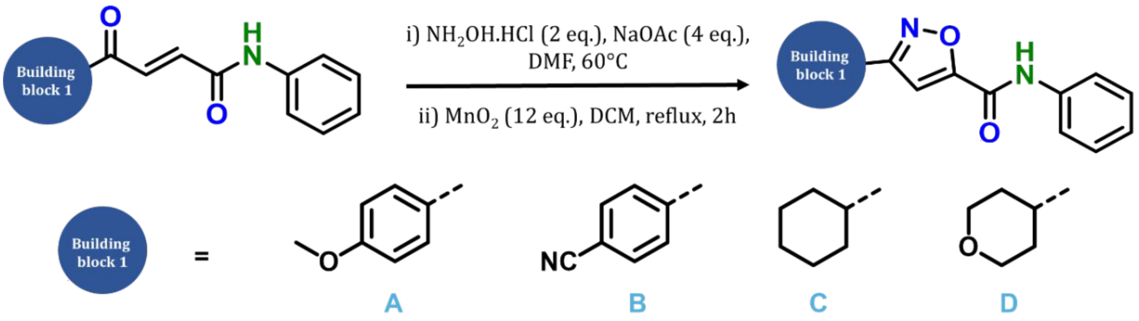

Product	Formula	Calculated mass $[M + H]^+$	Mass found $[M + H]^+$
A	$C_{17}H_{14}N_2O_3$	295.1080	Not found
B	$C_{17}H_{11}N_3O_2$	290.0927	Not found
C	$C_{16}H_{18}N_2O_2$	271.1444	Not found
D	$C_{15}H_{16}N_2O_3$	273.1236	274.2736
A + 2	$C_{17}H_{16}N_2O_3$	296.1161	Not found
B + 2	$C_{17}H_{13}N_3O_2$	291.1008	Not found
C + 2	$C_{16}H_{20}N_2O_2$	272.1525	Not found
D + 2	$C_{15}H_{18}N_2O_3$	274.1317	Not found

HRMS Analysis of Set 2 After Isoxazole Formation

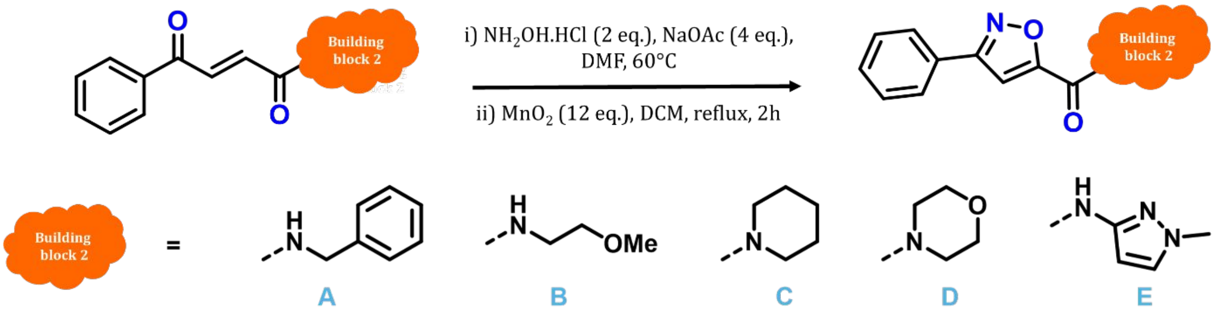

Product	Formula	Calculated mass $[M + H]^+$	Mass found $[M + H]^+$
A	$C_{17}H_{14}N_2O_2$	279.1131	279.1118
B	$C_{13}H_{14}N_2O_3$	247.1080	247.1059
C	$C_{15}H_{16}N_2O_2$	257.1287	257.1281
D	$C_{14}H_{14}N_2O_3$	259.1080	259.1460
E	$C_{14}H_{12}N_4O_2$	269.1036	269.1017

HPLC Analyses of Set 1 After the Isoxazole Formation Transformation

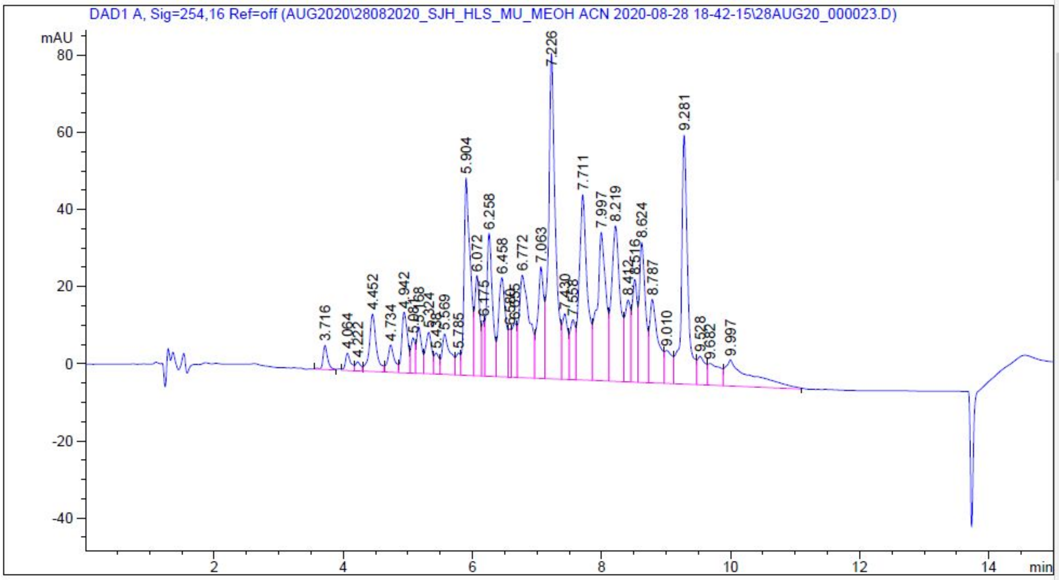

HPLC chromatogram of the resulting crude mixture after isoxazole formation applied to Set 1 in acidic conditions.

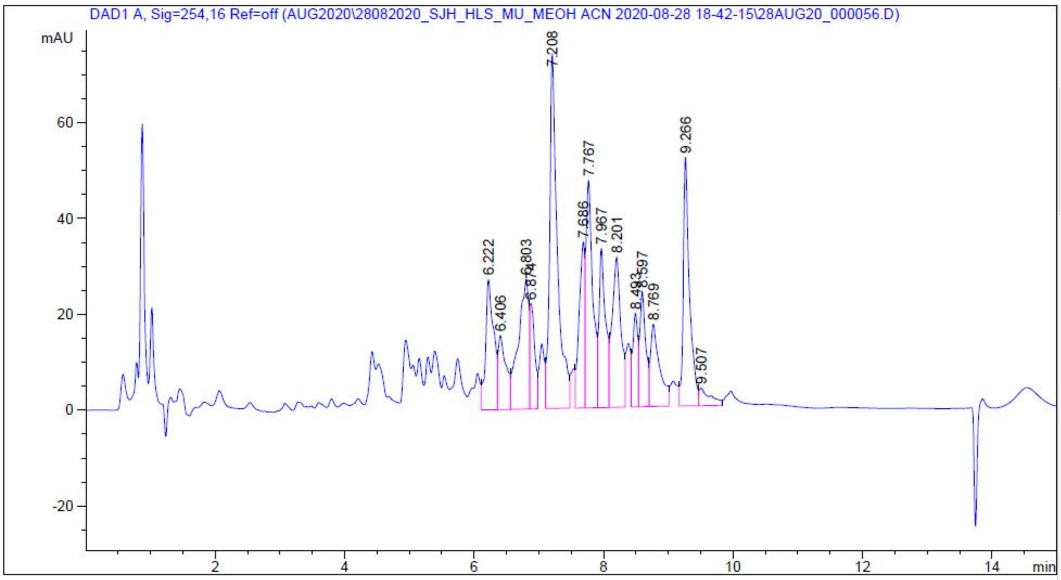

HPLC chromatogram of the resulting crude mixture After isoxazole formation applied to Set 1 in basic conditions.

HPLC Analyses of Set 2 After the Isoxazole Formation Transformation

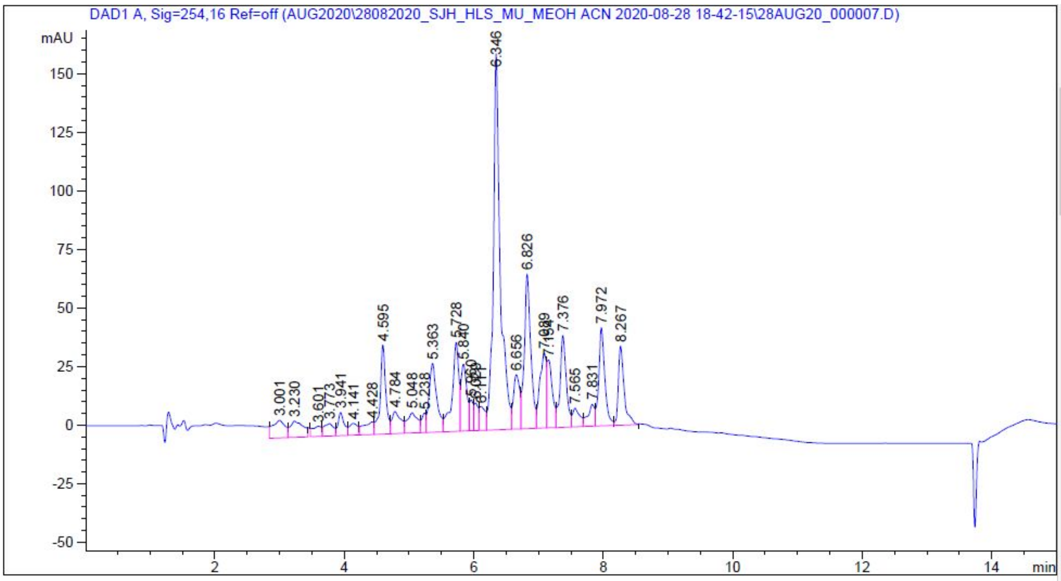

HPLC chromatogram of the resulting crude mixture after isoxazole formation applied to Set 2 in acidic conditions.

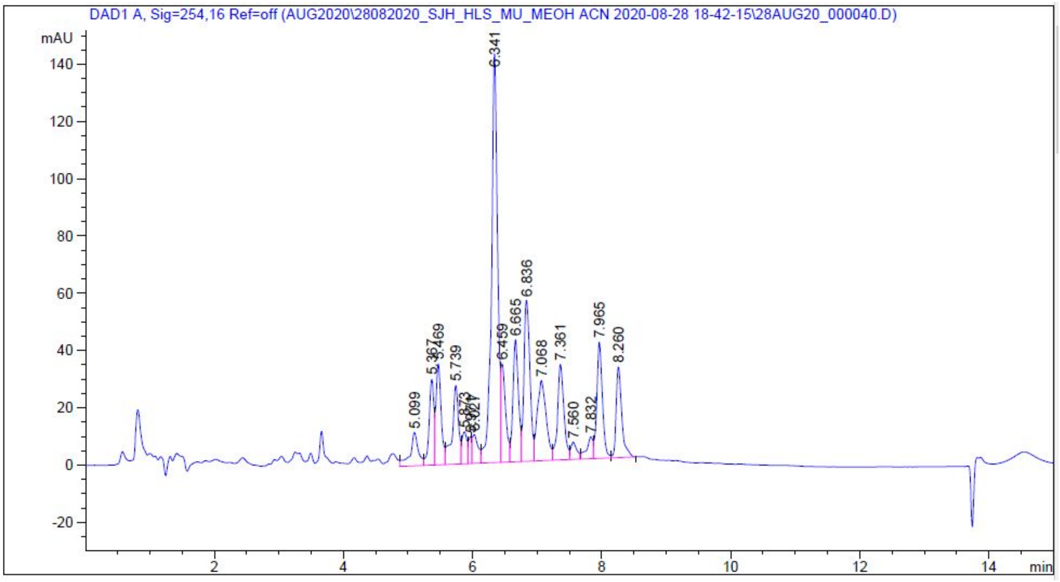

HPLC chromatogram of the resulting crude mixture after isoxazole formation applied to Set 2 in basic conditions.

HRMS Analysis of Set 1 After Pyrazolo[1.5-*a*]pyrimidine Formation

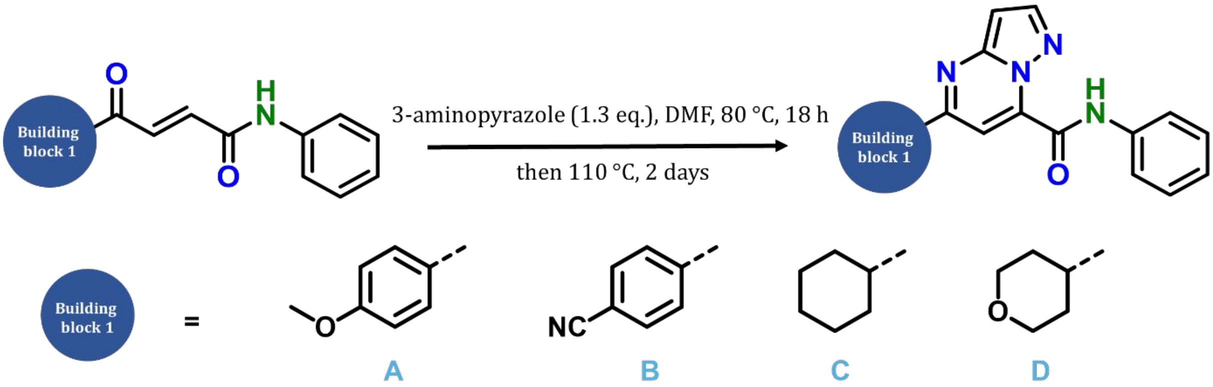

Product	Formula	Calculated mass [M + H]$^+$	Mass found [M + H]$^+$
A	$C_{20}H_{16}N_4O_2$	345.1349	345.2358
B	$C_{20}H_{13}N_5O$	340.1196	Not found
C	$C_{19}H_{20}N_4O$	321.1713	321.1744
D	$C_{18}H_{18}N_4O_2$	323.1505	323.1496
B + 2	$C_{20}H_{15}N_5O$	341.1277	341.1989

HRMS Analysis of Set 2 After Pyrazolo[1.5-*a*]pyrimidine Formation

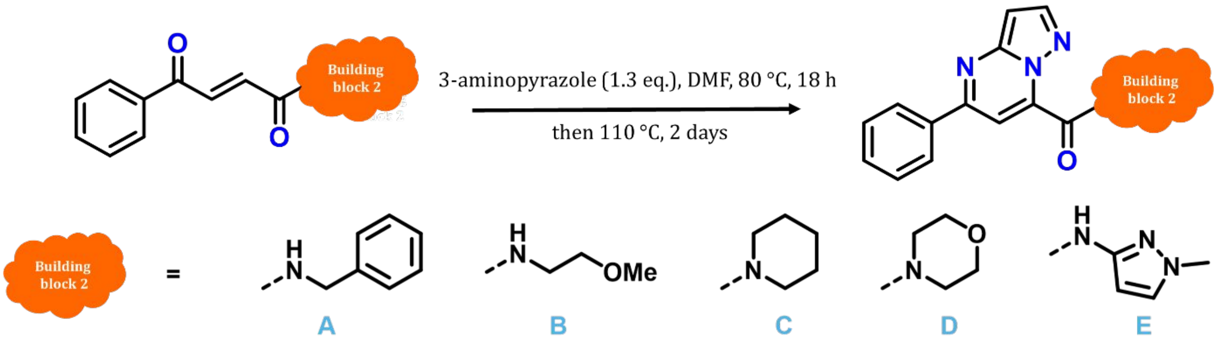

Product	Formula	Calculated mass [M + H]$^+$	Mass found [M + H]$^+$
A	$C_{20}H_{16}N_4O$	329.1400	329.1412
B	$C_{16}H_{16}N_4O_2$	297.1349	297.1364
C	$C_{18}H_{18}N_4O$	307.1556	307.1554
D	$C_{17}H_{16}N_4O_2$	309.1349	309.1338
E	$C_{17}H_{14}N_6O$	319.1305	319.1285

HPLC Analyses of Set 1 After the Pyrazolo[1,5-*a*]pyrimidine Formation Transformation

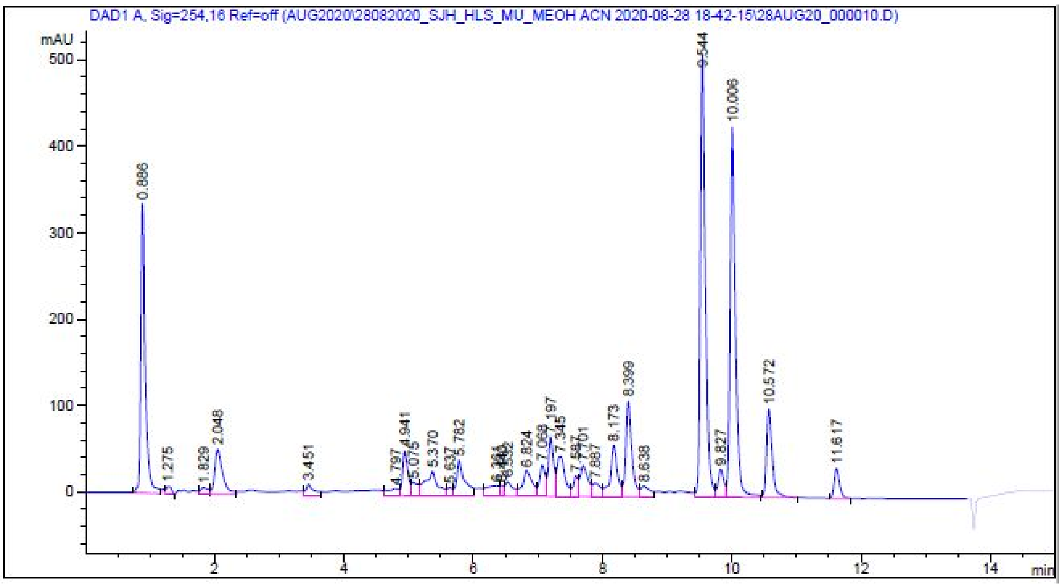

HPLC chromatogram of the resulting crude mixture after pyrazolo[1,5-a]pyrimidine synthesis applied to Set 1 in acidic conditions.

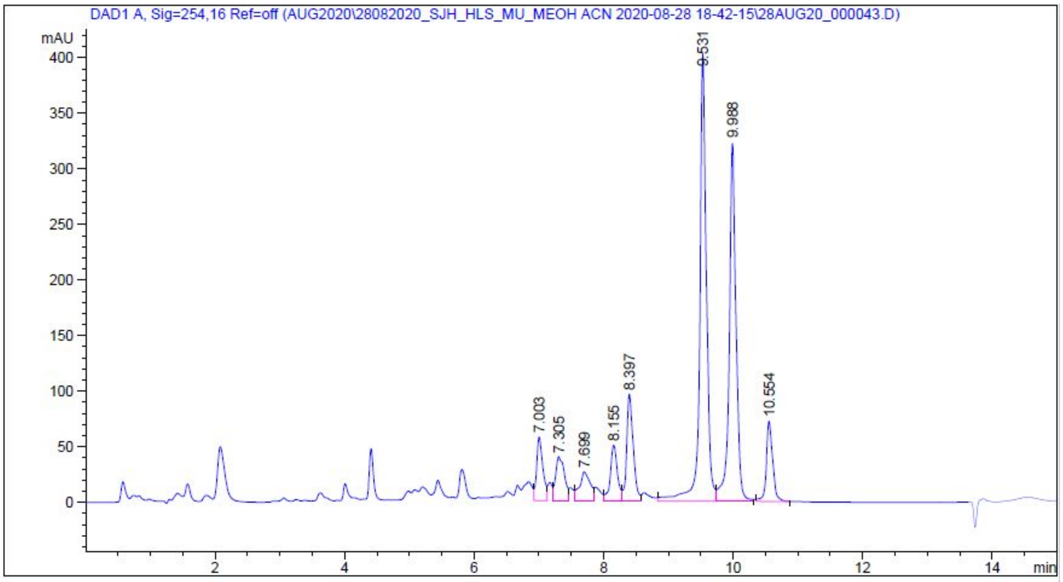

HPLC chromatogram of the resulting crude mixture after pyrazolo[1,5-a]pyrimidine synthesis applied to Set 1 in basic conditions.

HPLC Analyses of Set 2 After the Pyrazolo[1,5-*a*]pyrimidine Formation Transformation

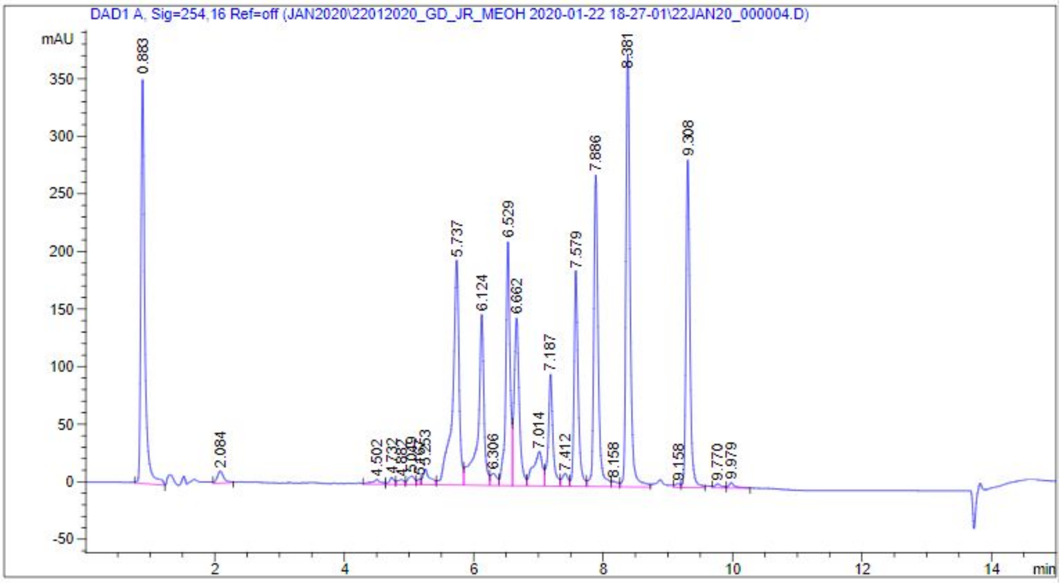

HPLC chromatogram of the resulting crude mixture after pyrazolo[1,5-a]pyrimidine synthesis applied to Set 2 in acidic conditions.

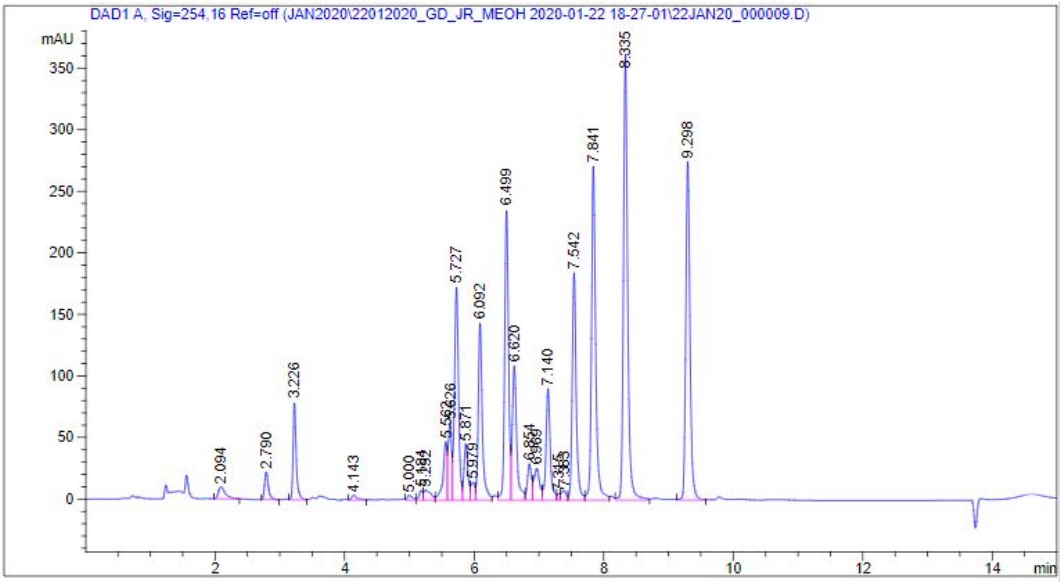

HPLC chromatogram of the resulting crude mixture after pyrazolo[1,5-a]pyrimidine synthesis applied to Set 2 in basic conditions.

HRMS Analysis of Set 1 After Imidazo[1.2-*a*]pyridine Formation

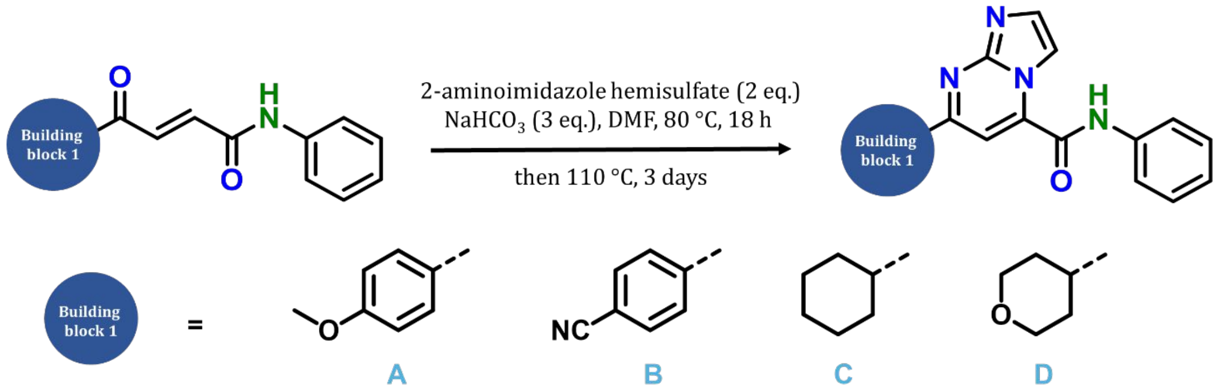

Product	Formula	Calculated mass [M + H]+	Mass found [M + H]+
A	$C_{20}H_{16}N_4O_2$	345.1349	345.1358
B	$C_{20}H_{13}N_5O$	340.1196	340.1191
C	$C_{19}H_{20}N_4O$	321.1713	321.1707
D	$C_{18}H_{18}N_4O_2$	323.1505	323.1496

HRMS Analysis of Set 2 After Imidazo[1.2-*a*]pyridine Formation

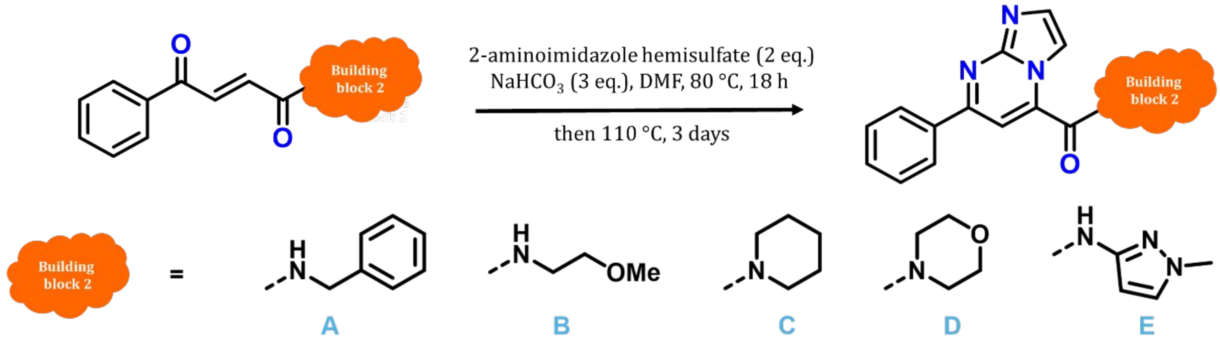

Product	Formula	Calculated mass [M + H]+	Mass found [M + H]+
A	$C_{20}H_{16}N_4O$	329.1400	329.1412
B	$C_{16}H_{16}N_4O_2$	297.1349	297.1364
C	$C_{18}H_{18}N_4O$	307.1556	307.1554
D	$C_{17}H_{16}N_4O_2$	309.1349	309.1338
E	$C_{17}H_{14}N_6O$	319.1305	319.1322

HPLC Analyses of Set 1 After the Imidazo[1,2-*a*]pyridine Formation Transformation

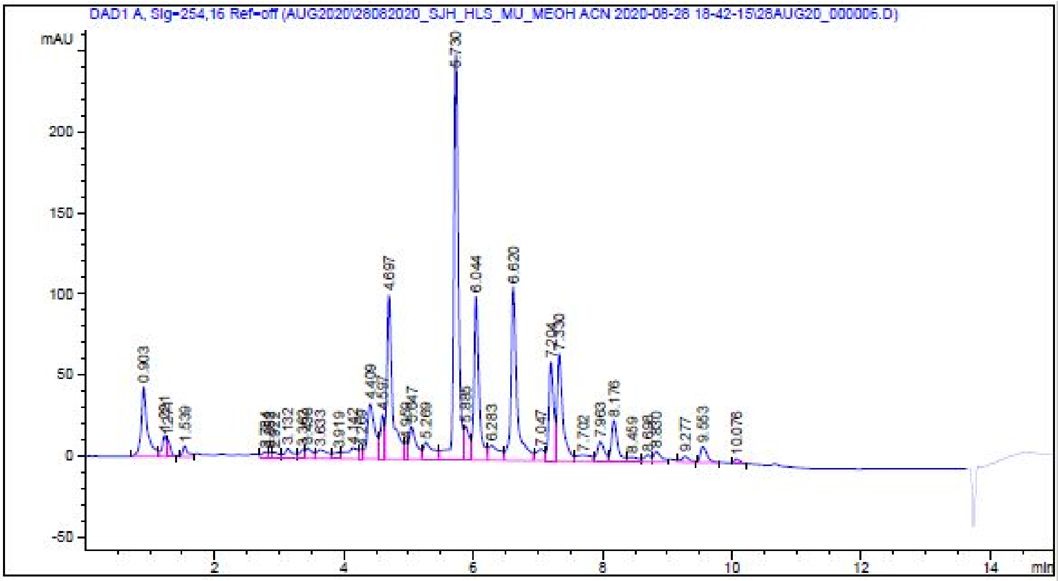

HPLC chromatogram of the resulting crude mixture after imidazo[1,2-a]pyridine synthesis applied to Set 1 in acidic conditions.

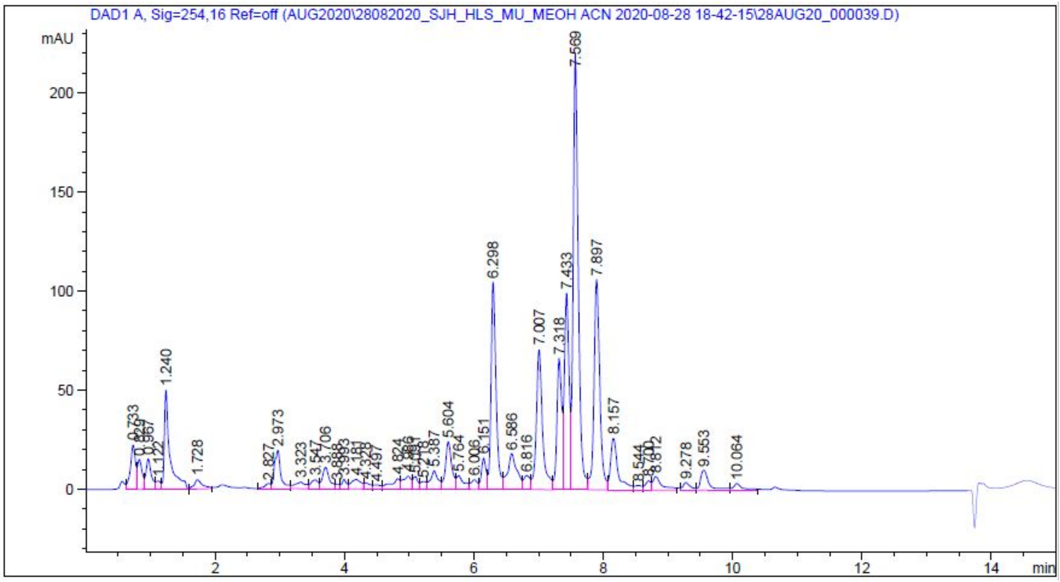

HPLC chromatogram of the resulting crude mixture after imidazo[1,2-a]pyridine synthesis applied to Set 1 in basic conditions.

HPLC Analyses of Set 2 After the Imidazo[1,2-*a*]pyridine Formation Transformation

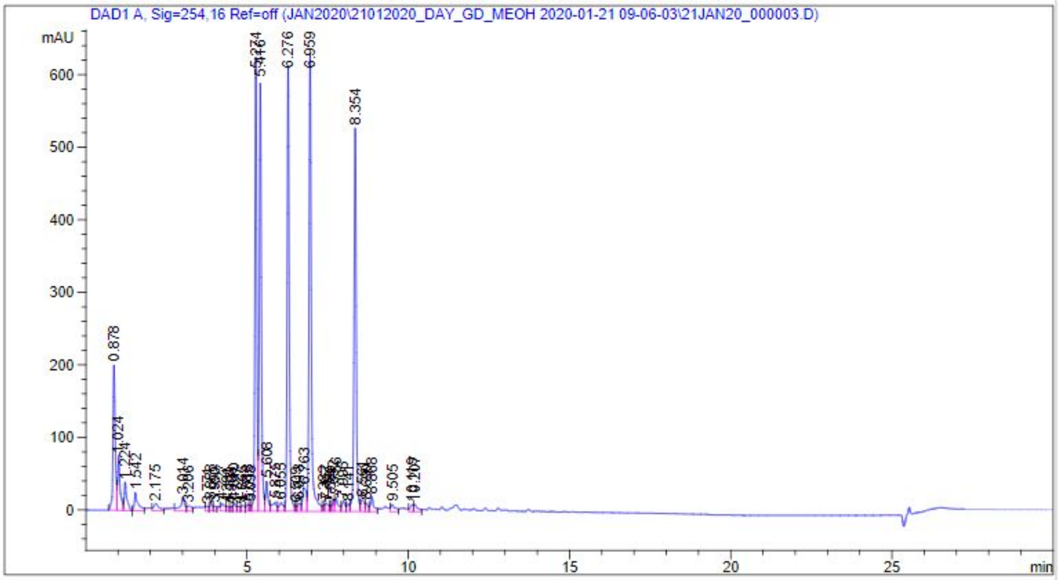

HPLC chromatogram of the resulting crude mixture after imidazo[1,2-a]pyridine synthesis applied to Set 2 in acidic conditions.

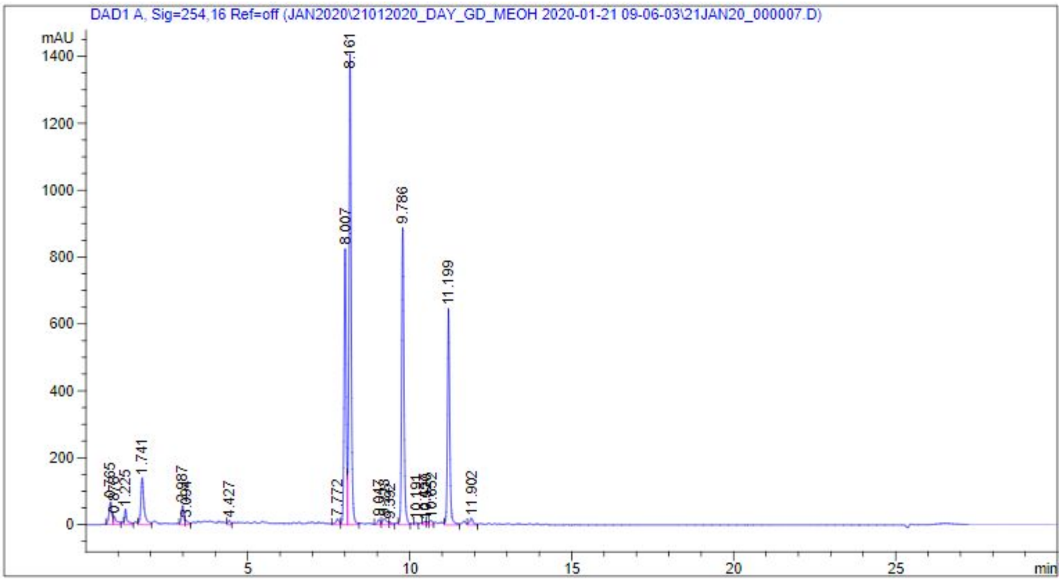

HPLC chromatogram of the resulting crude mixture after imidazo[1,2-a]pyridine synthesis applied to Set 2 in basic conditions.

HRMS Analysis of Set 1 After 2-Methoxypyrimidine Formation

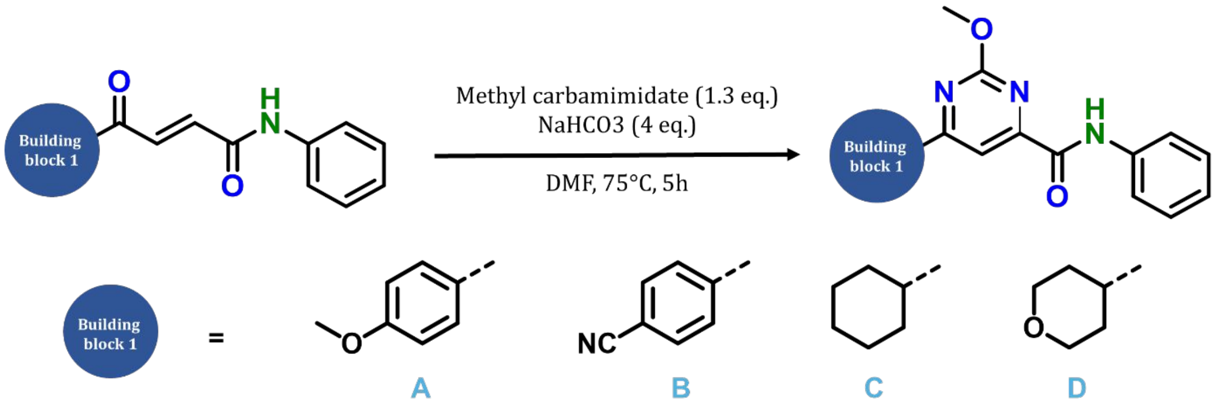

Product	Formula	Calculated mass $[M + H]^+$	Mass found $[M + H]^+$
A	$C_{19}H_{17}N_3O_3$	336.1345	336.1322
B	$C_{19}H_{14}N_4O_2$	331.1192	Not found
C	$C_{18}H_{21}N_3O_2$	312.1709	312.173
D	$C_{17}H_{19}N_3O_3$	314.1502	314.1494

HRMS Analysis of Set 2 After 2-Methoxypyrimidine Formation

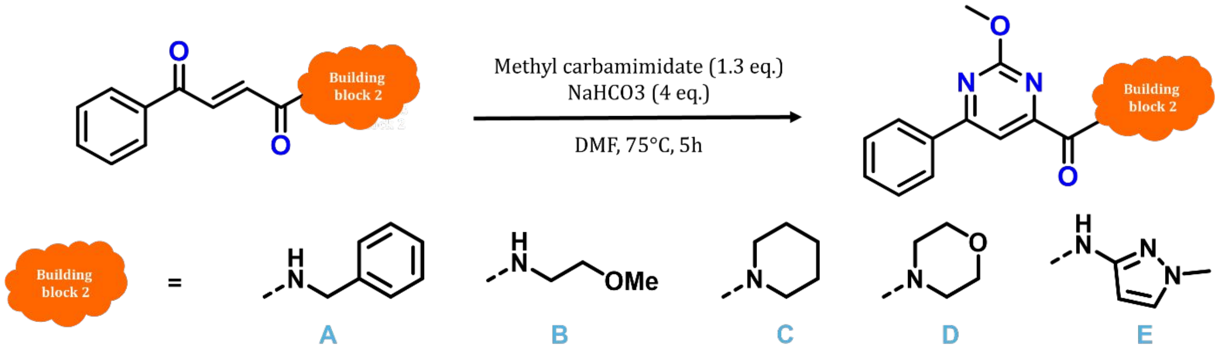

Product	Formula	Calculated mass $[M + H]^+$	Mass found $[M + H]^+$
A	$C_{20}H_{18}N_2O$	303.1495	303.1499
B	$C_{16}H_{18}N_2O_2$	271.1444	271.1457
C	$C_{18}H_{20}N_2O$	281.1651	281.1660
D	$C_{17}H_{18}N_2O_2$	283.1444	283.1450
E	$C_{17}H_{16}N_4O$	293.1400	293.1409

HPLC Analyses of Set 1 After the 2-Methoxypyrimidine Formation Transformation

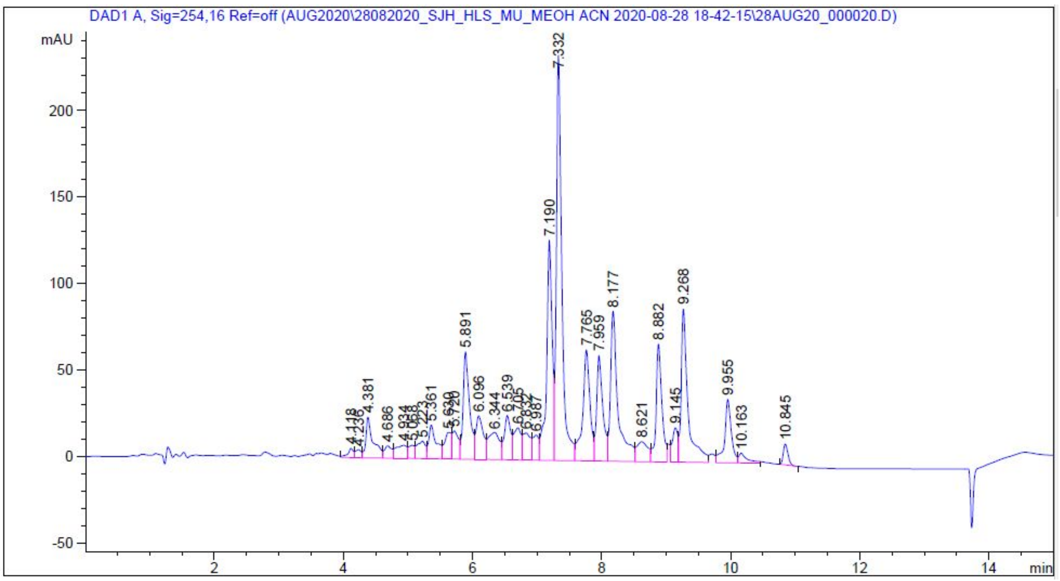

HPLC chromatogram of the resulting crude mixture after 2-methoxypyrimidine formation applied to Set 1 in acidic conditions.

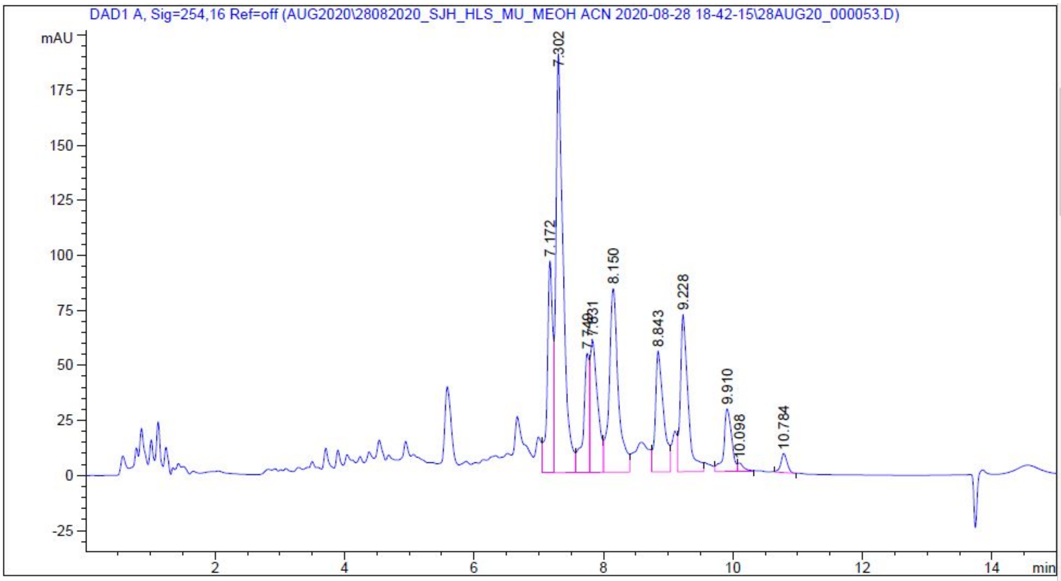

HPLC chromatogram of the resulting crude mixture after 2-methoxypyrimidine formation applied to Set 1 in basic conditions.

HPLC Analyses of Set 2 After the 2-Methoxypyrimidine Formation Transformation

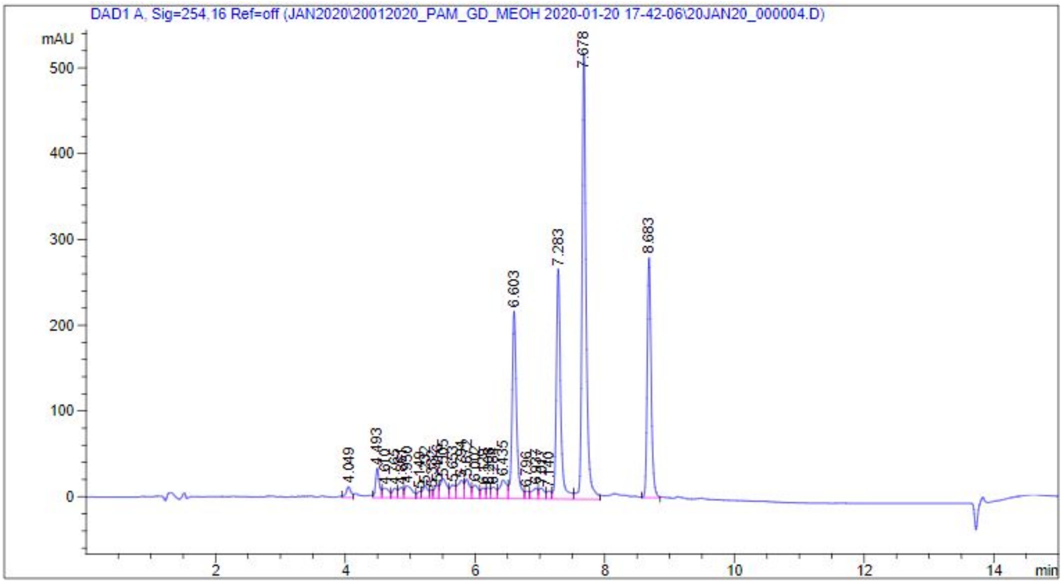

HPLC chromatogram of the resulting crude mixture after 2-methoxypyrimidine formation applied to Set 2 in acidic conditions.

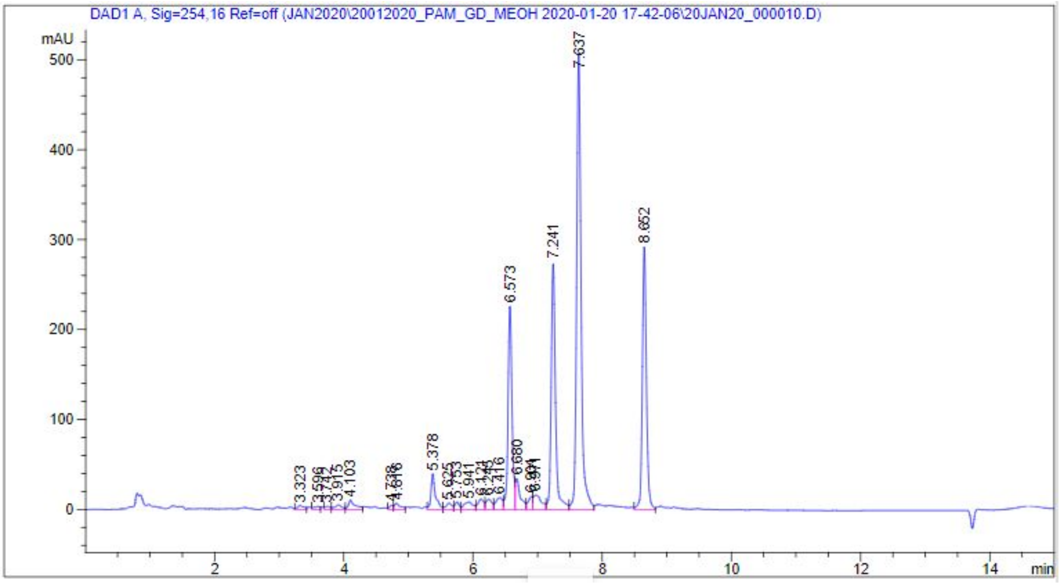

HPLC chromatogram of the resulting crude mixture after 2-methoxypyrimidine formation applied to Set 2 in basic conditions.

HRMS Analysis of Set 1 After 2-PMB-thiopyrimidine Formation

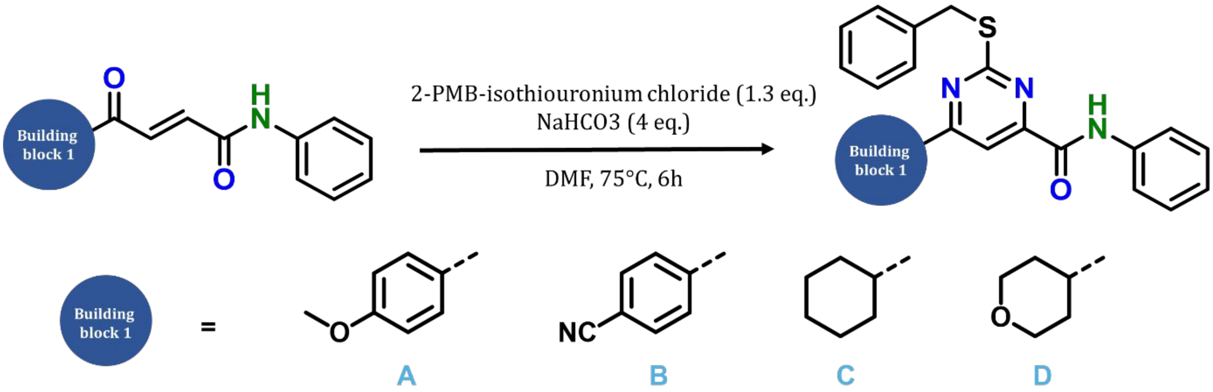

Product	Formula	Calculated mass [M + H]+	Mass found [M + H]+
A	$C_{26}H_{23}N_3O_3S$	458.1536	458.1508
B	$C_{26}H_{20}N_4O_2S$	453.1383	454.1234
C	$C_{25}H_{27}N_3O_2S$	434.1899	434.1868
D	$C_{24}H_{25}N_3O_3S$	436.1692	436.1667
A Michael addition	$C_{25}H_{25}NO_4S$	436.1580	436.1539
B Michael addition	$C_{25}H_{22}N_2O_3S$	431.1427	Not found
C Michael addition	$C_{24}H_{29}NO_3S$	412.1944	412.1942
D Michael addition	$C_{23}H_{27}NO_4S$	414.1736	414.1733

B.16. HRMS Analysis of Set 2 After 2-PMB-thiopyrimidine Formation

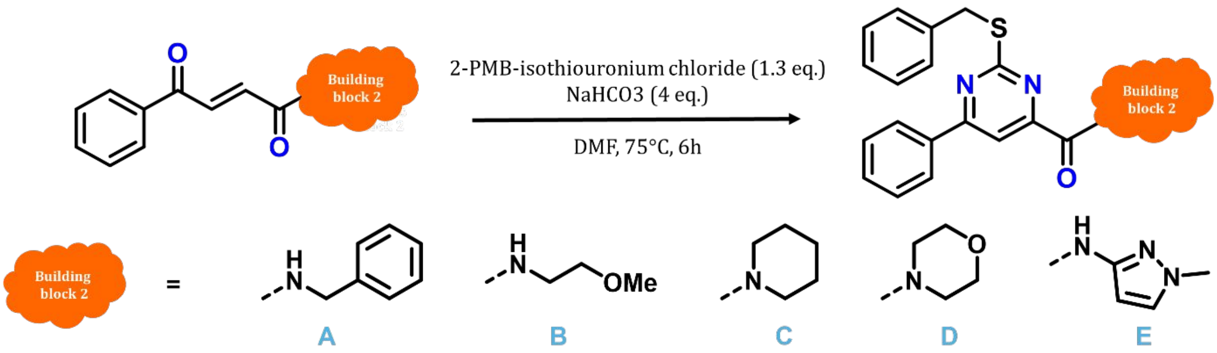

Product	Formula	Calculated mass [M + H]+	Mass found [M + H]+
A	$C_{26}H_{23}N_3O_2S$	442.1586	442.1592
B	$C_{22}H_{23}N_3O_3S$	410.1536	410.1536
C	$C_{24}H_{25}N_3O_2S$	420.1743	420.1726
D	$C_{23}H_{23}N_3O_3S$	422.1536	422.154
E	$C_{23}H_{21}N_5O_2S$	432.1491	432.1476
A Michael addition	$C_{25}H_{25}NO_3S$	420.1631	420.1601
B Michael addition	$C_{21}H_{25}NO_4S$	388.1580	388.1534
C Michael addition	$C_{23}H_{27}NO_3S$	398.1787	398.1792
D Michael addition	$C_{22}H_{25}NO_4S$	400.1580	400.1572
E Michael addition	$C_{22}H_{24}N_4O_2S$	409.1695	Not found

HPLC Analyses of Set 1 After the 2-PMB-thiopyrimidine Formation Transformation

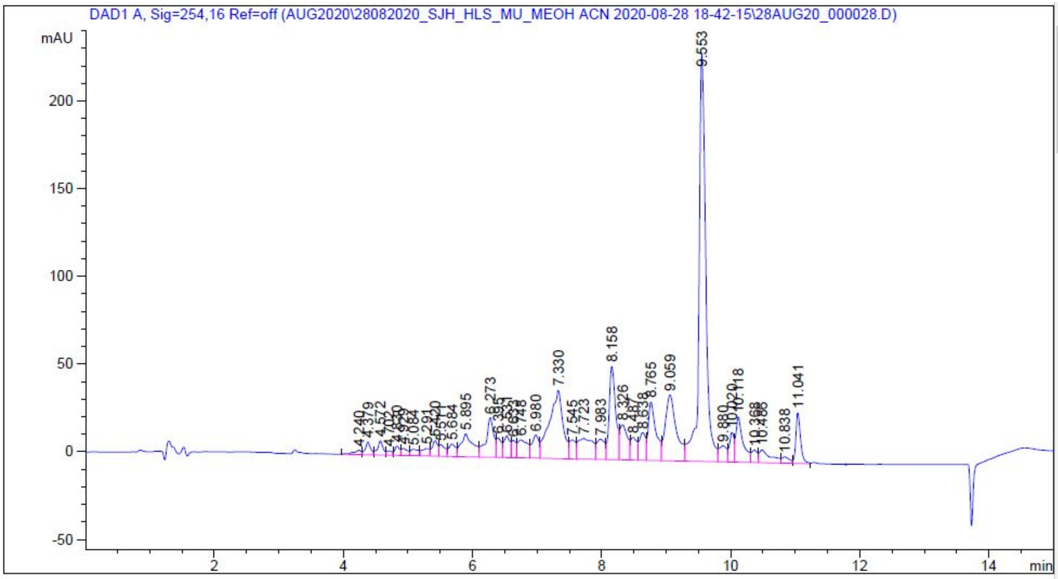

HPLC chromatogram of the resulting crude mixture after 2-PMB-thiopyrimidine formation applied to Set 1 in acidic conditions.

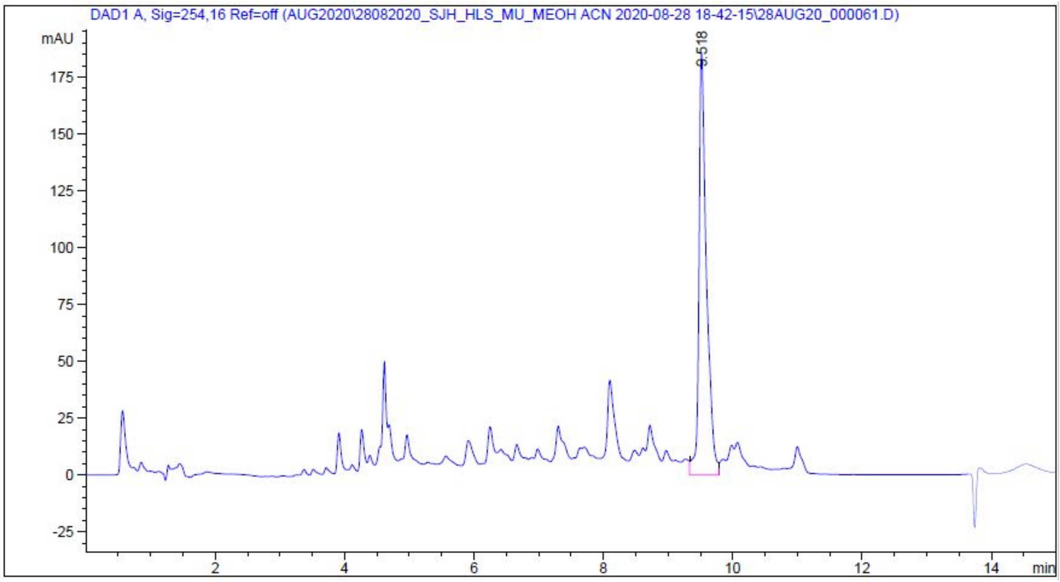

HPLC chromatogram of the resulting crude mixture after 2-PMB-thiopyrimidine formation applied to Set 1 in basic conditions.

HPLC Analyses of Set 2 After the 2-PMB-thiopyrimidine Formation Transformation

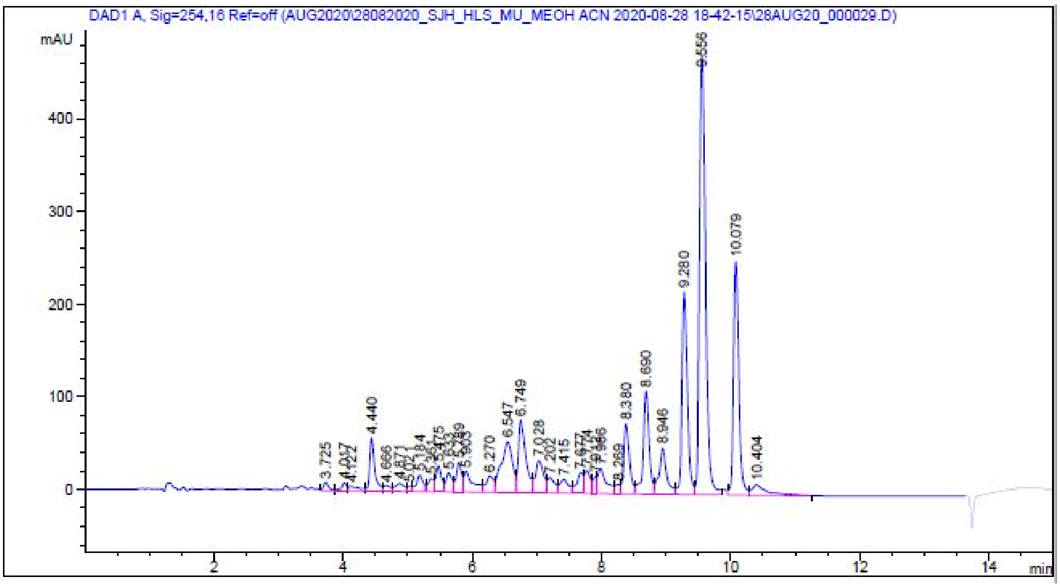

HPLC chromatogram of the resulting crude mixture after 2-PMB-thiopyrimidine formation applied to Set 2 in acidic conditions.

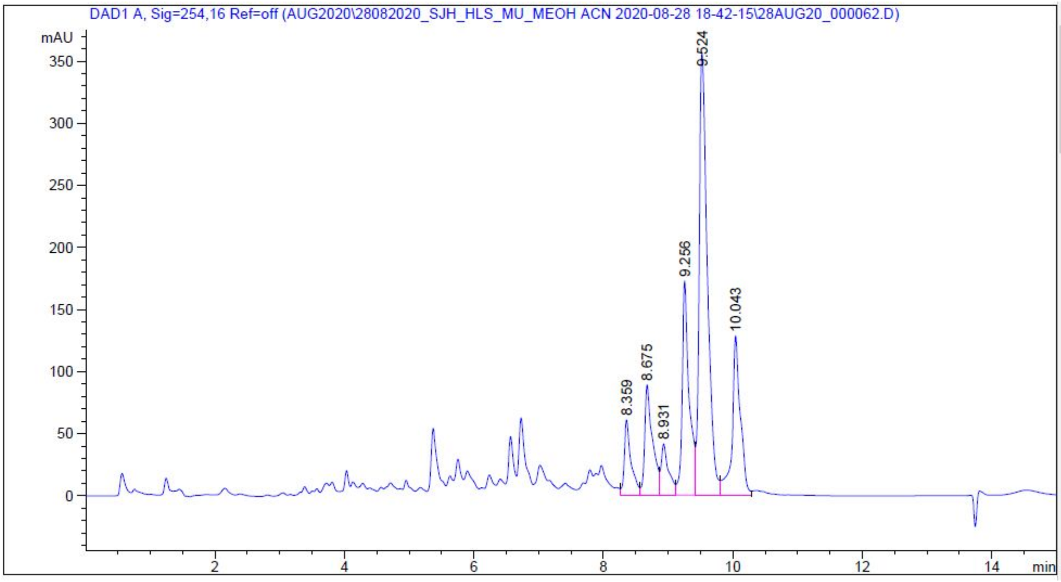

HPLC chromatogram of the resulting crude mixture after 2-PMB-thiopyrimidine formation applied to Set 2 in basic conditions.

HRMS Analysis of Set 1 After 2-Aminopyrimidine Formation

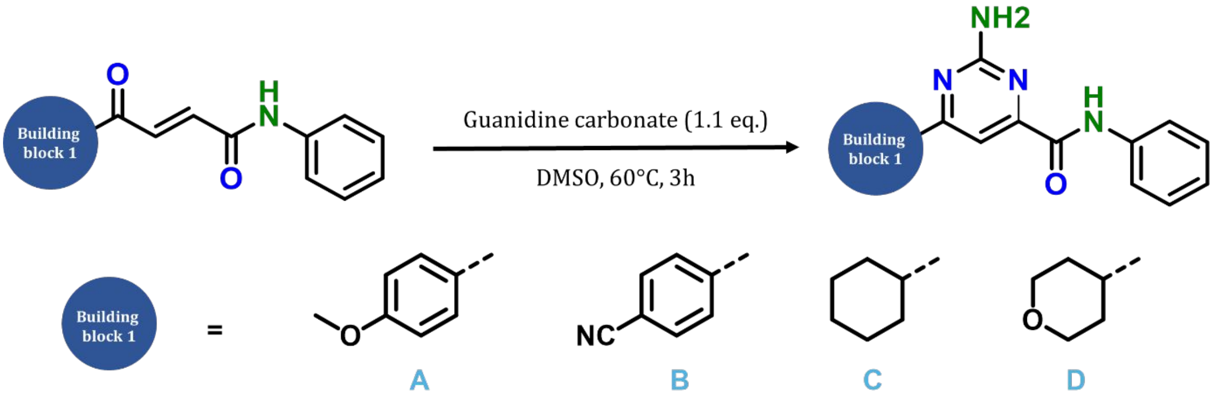

Product	Formula	Calculated mass [M + H]+	Mass found [M + H]+
A	$C_{18}H_{16}N_4O_2$	321.1349	321.1341
B	$C_{18}H_{13}N_5O$	316.1196	316.1174
C	$C_{17}H_{20}N_4O$	297.1713	297.1716
D	$C_{16}H_{18}N_4O_2$	299.1505	299.1495

HRMS Analysis of Set 2 After 2-Aminopyrimidine Formation

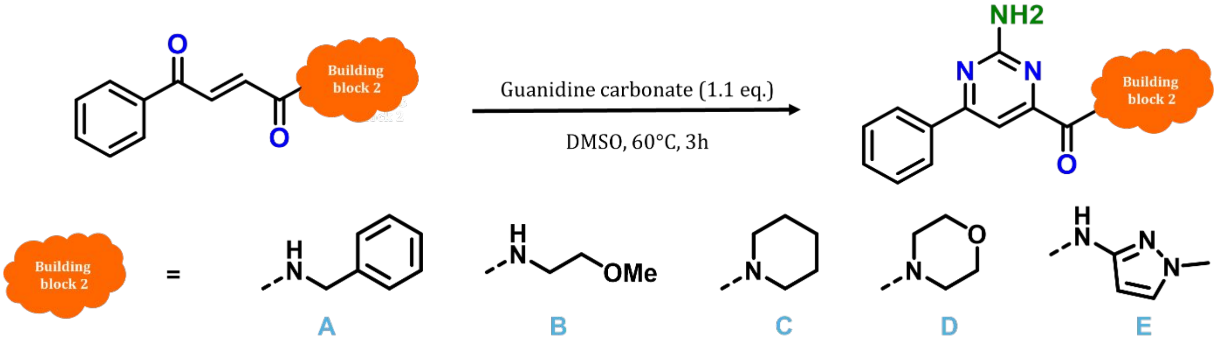

Product	Formula	Calculated mass [M + H]+	Mass found [M + H]+
A	$C_{18}H_{16}N_4O$	305.1400	305.1404
B	$C_{14}H_{16}N_4O_2$	273.1349	273.1366
C	$C_{16}H_{18}N_4O$	283.1556	283.1553
D	$C_{15}H_{16}N_4O_2$	285.1349	285.1345
E	$C_{15}H_{14}N_6O$	295.1305	295.1299

HPLC Analyses of Set 1 After the 2-Aminopyrimidine Formation Transformation

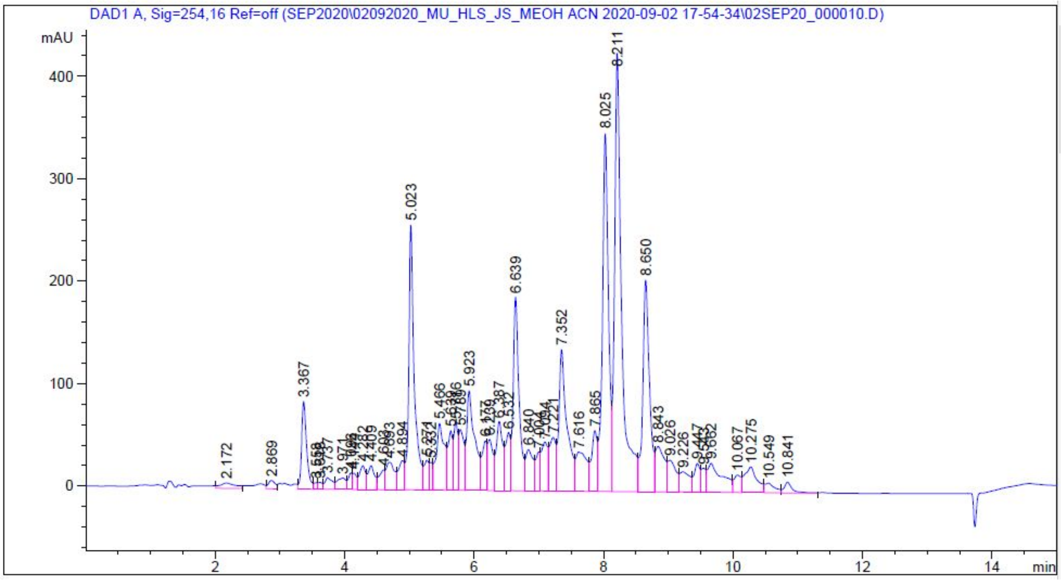

HPLC chromatogram of the resulting crude mixture after 2-aminopyrimidine formation applied to Set 1 in acidic conditions.

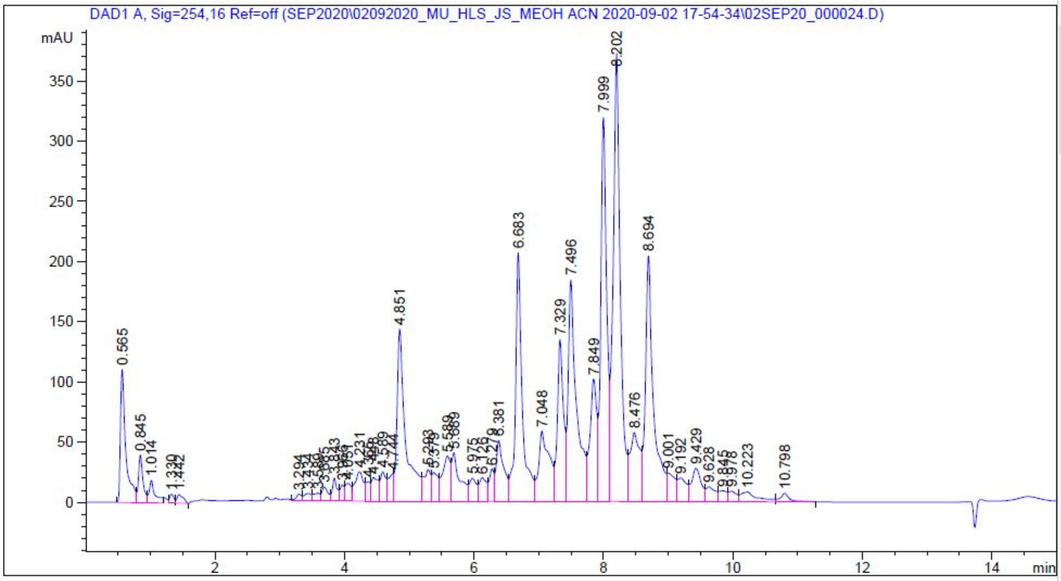

HPLC chromatogram of the resulting crude mixture after 2-aminopyrimidine formation applied to Set 1 in basic conditions.

HPLC Analyses of Set 2 After the 2-Aminopyrimidine Formation Transformation

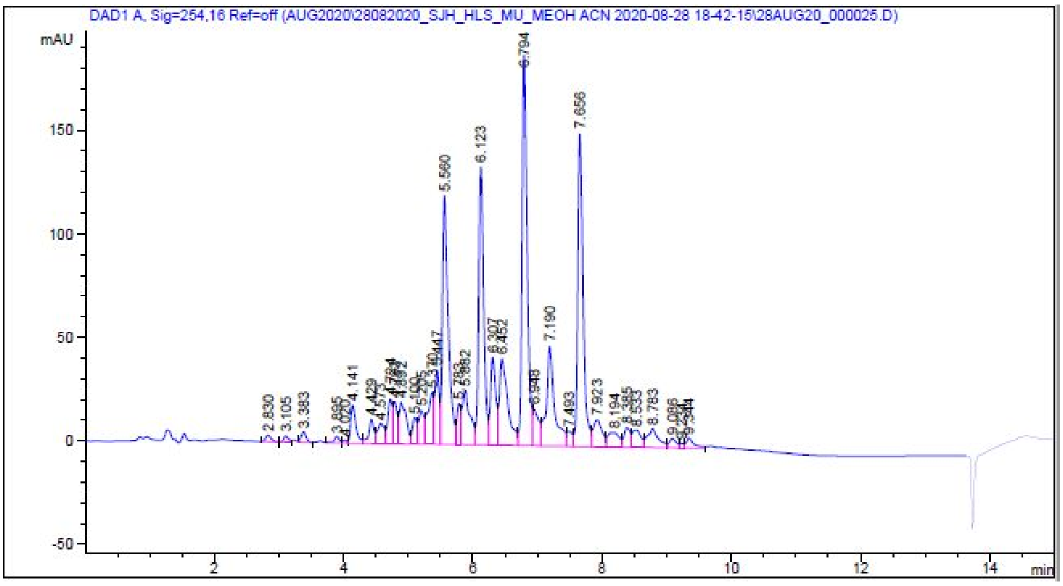

HPLC chromatogram of the resulting crude mixture after 2-aminopyrimidine formation applied to Set 2 in acidic conditions.

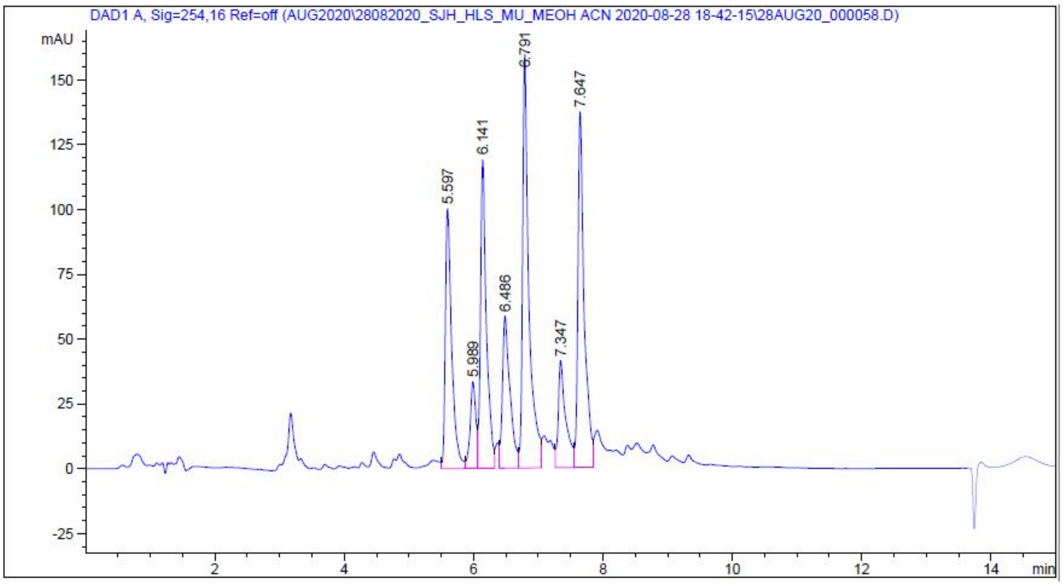

HPLC chromatogram of the resulting crude mixture after 2-aminopyrimidine formation applied to Set 2 in basic conditions.

HRMS Analysis of Set 1 After Kröhnke Pyridine Synthesis

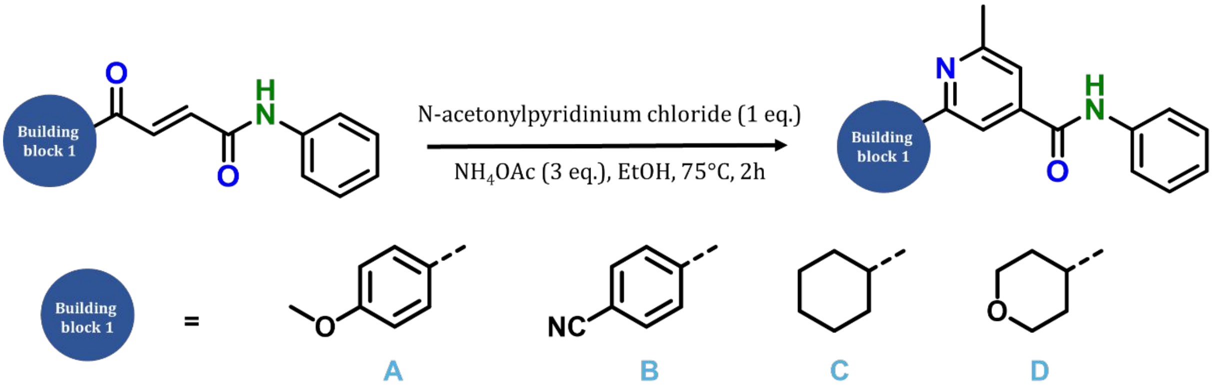

Product	Formula	Calculated mass $[M + H]^+$	Mass found $[M + H]^+$
A	$C_{20}H_{18}N_2O_2$	319.1444	319.1468
B	$C_{20}H_{15}N_3O$	314.1291	314.1312
C	$C_{19}H_{22}N_2O$	295.1808	295.1827
D	$C_{18}H_{20}N_2O_2$	297.1600	297.1610

HRMS Analysis of Set 2 After Kröhnke Pyridine Synthesis

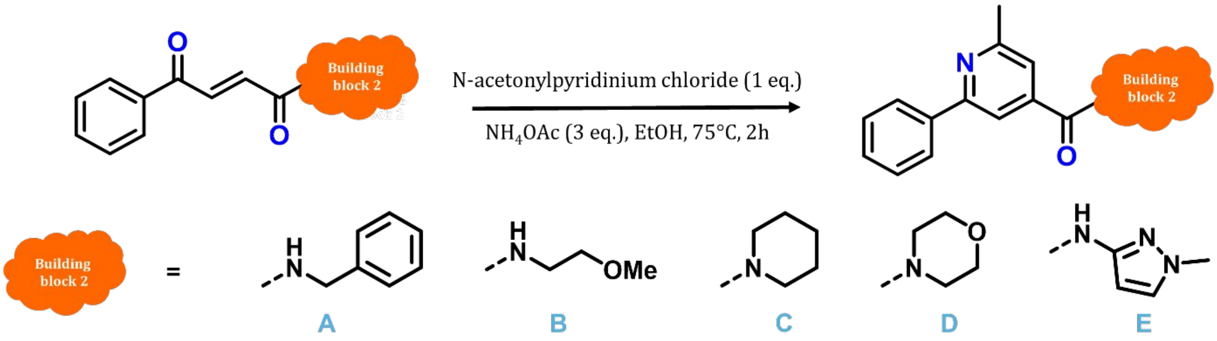

Product	Formula	Calculated mass $[M + H]^+$	Mass found $[M + H]^+$
A	$C_{20}H_{18}N_2O$	303.1495	303.1499
B	$C_{16}H_{18}N_2O_2$	271.1444	271.1457
C	$C_{18}H_{20}N_2O$	281.1651	281.1660
D	$C_{17}H_{18}N_2O_2$	283.1444	283.1450
E	$C_{17}H_{16}N_4O$	293.1400	293.1409

HPLC Analyses of Set 1 After the Kröhnke Pyridine Synthesis Transformation

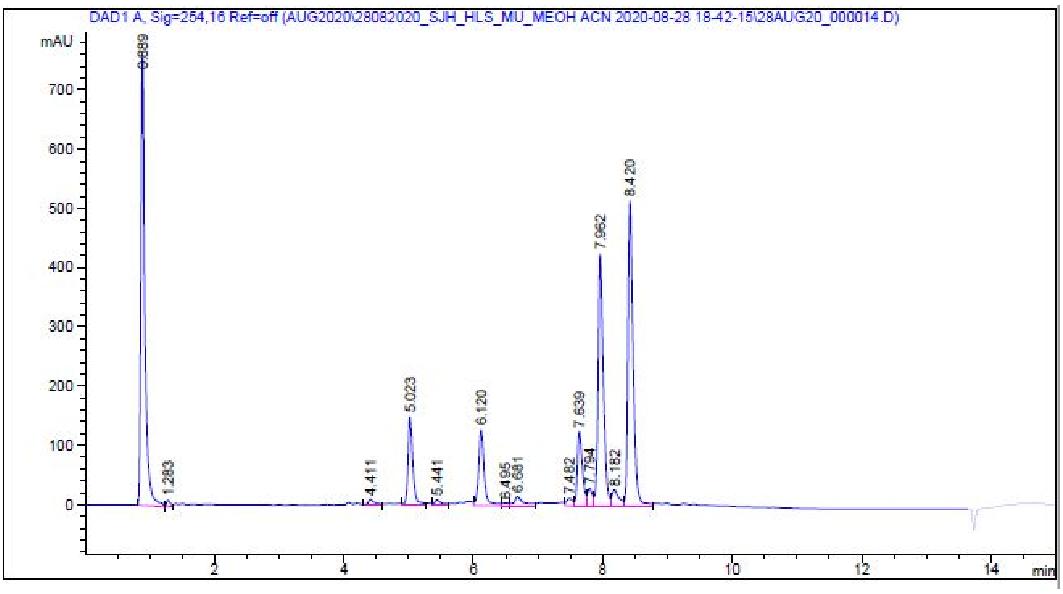

HPLC chromatogram of the resulting crude mixture after Kröhnke pyridine synthesis applied to Set 1 in acidic conditions.

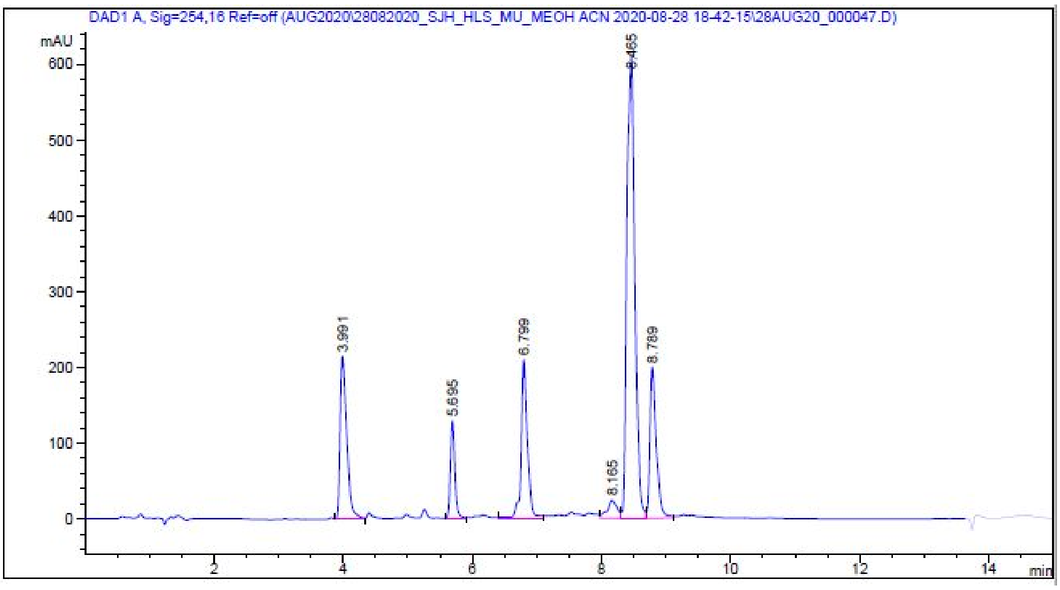

HPLC chromatogram of the resulting crude mixture after Kröhnke pyridine synthesis applied to Set 1 in basic conditions.

HPLC Analyses of Set 2 After the Kröhnke Pyridine Synthesis Transformation

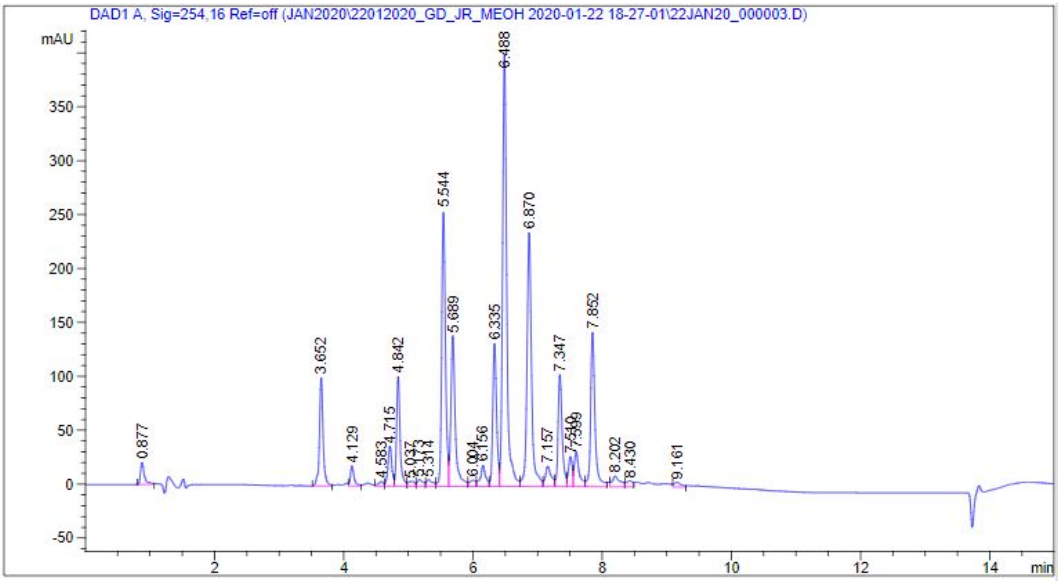

HPLC chromatogram of the resulting crude mixture after Kröhnke pyridine synthesis applied to Set 2 in acidic conditions.

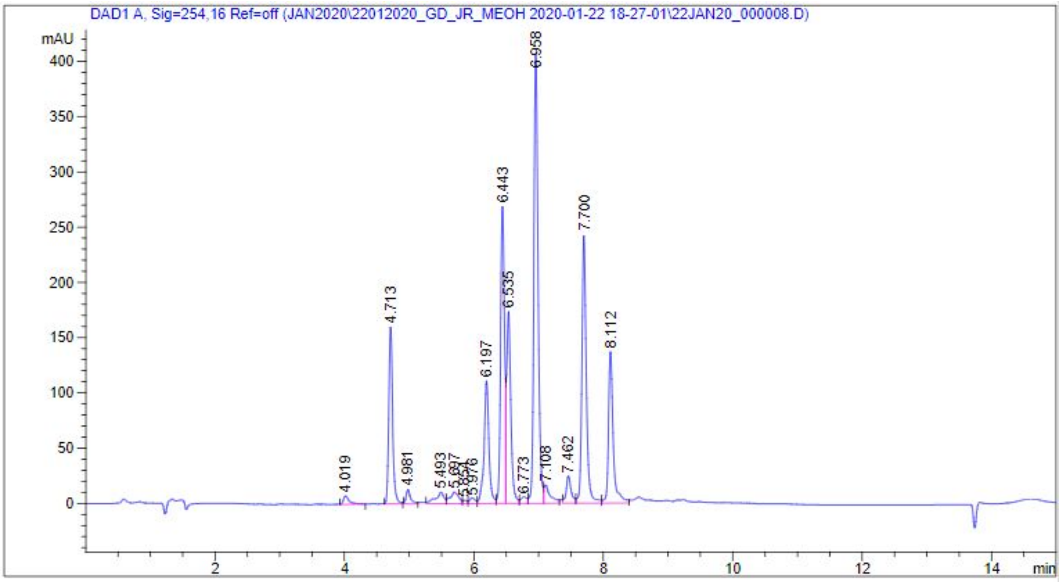

HPLC chromatogram of the resulting crude mixture after Kröhnke pyridine synthesis applied to Set 2 in basic conditions.

3.2.2. Transformations: 3,4-Cycloadditions

HRMS Analysis of Set 1 After Corey-Chaykovski Cyclopropanation

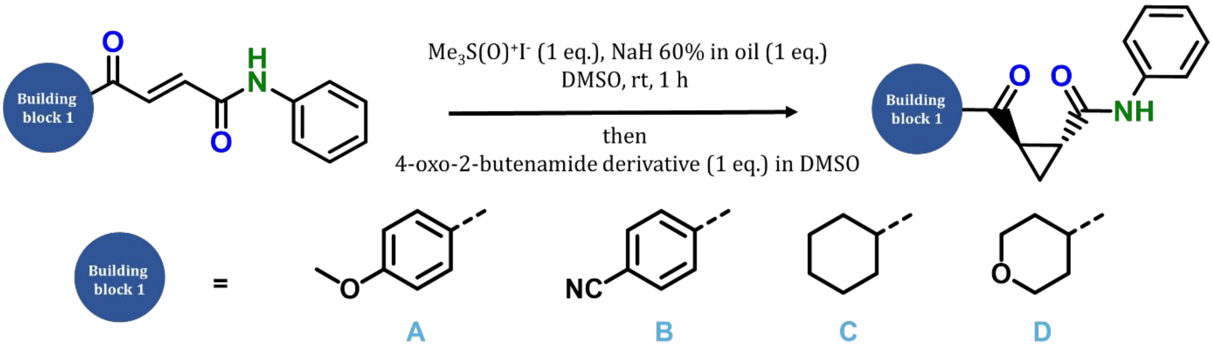

Product	Formula	Calculated mass [M + H]+	Mass found [M + H]+
A	$C_{18}H_{17}NO_3$	296.1284	296.1288
B	$C_{18}H_{14}N_2O_2$	291.1131	291.1132
C	$C_{17}H_{21}NO_2$	272.1648	272.1672
D	$C_{16}H_{19}NO_3$	274.134	274.1415

HRMS Analysis of Set 2 After Corey-Chaykovski Cyclopropanation

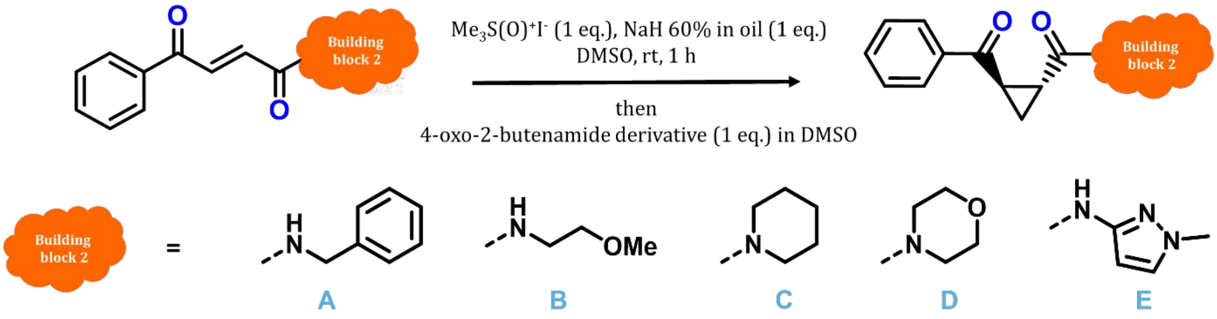

Product	Formula	Calculated mass [M + H]+	Mass found [M + H]+
A	$C_{18}H_{17}NO_2$	280.1335	280.1345
B	$C_{14}H_{17}NO_3$	248.1284	248.1294
C	$C_{16}H_{19}NO_2$	258.1491	258.1492
D	$C_{15}H_{17}NO_3$	260.1284	260.1282
E	$C_{15}H_{15}N_3O_2$	270.1240	270.1227

HPLC Analyses of Set 1 After the Corey-Chaykovski Cyclopropanation Transformation

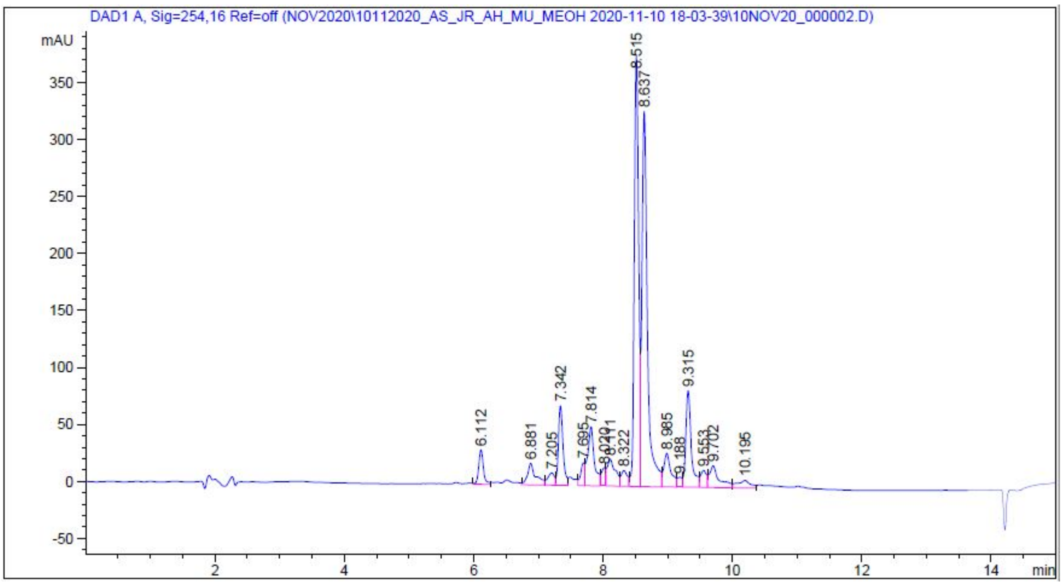

HPLC chromatogram of the resulting crude mixture after Corey-Chaykovski cyclopropanation applied to Set 1 in acidic conditions.

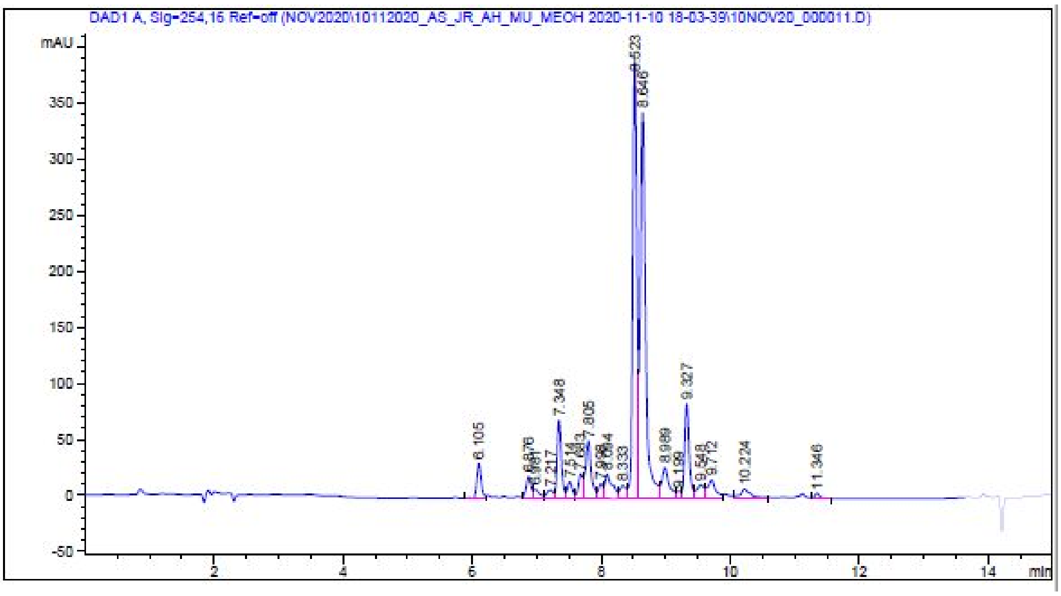

HPLC chromatogram of the resulting crude mixture after Corey-Chaykovski cyclopropanation applied to Set 1 in basic conditions.

HPLC Analyses of Set 2 After the Corey-Chaykovski Cyclopropanation Transformation

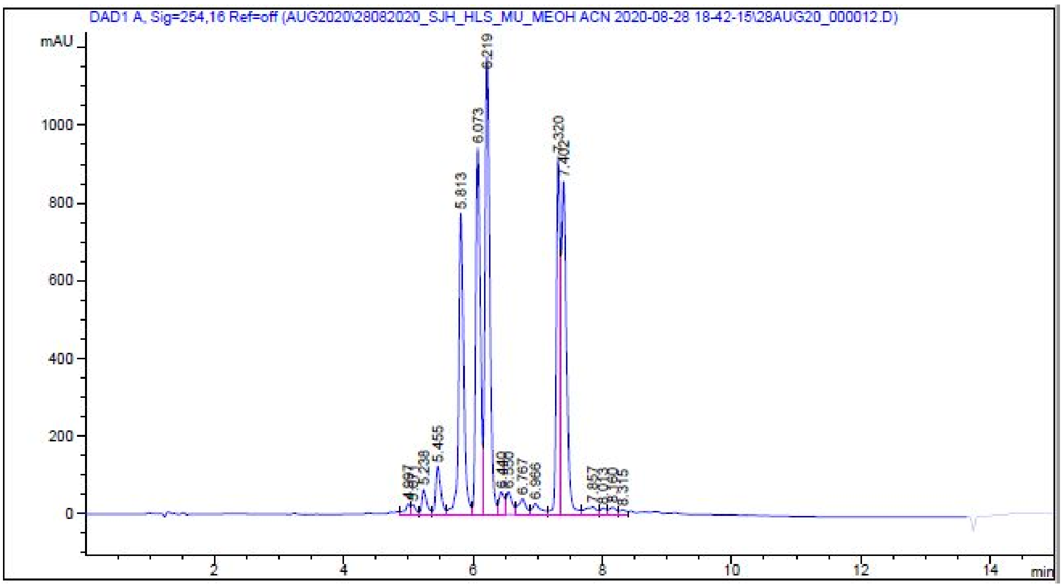

HPLC chromatogram of the resulting crude mixture after Corey-Chaykovski cyclopropanation applied to Set 2 in acidic conditions.

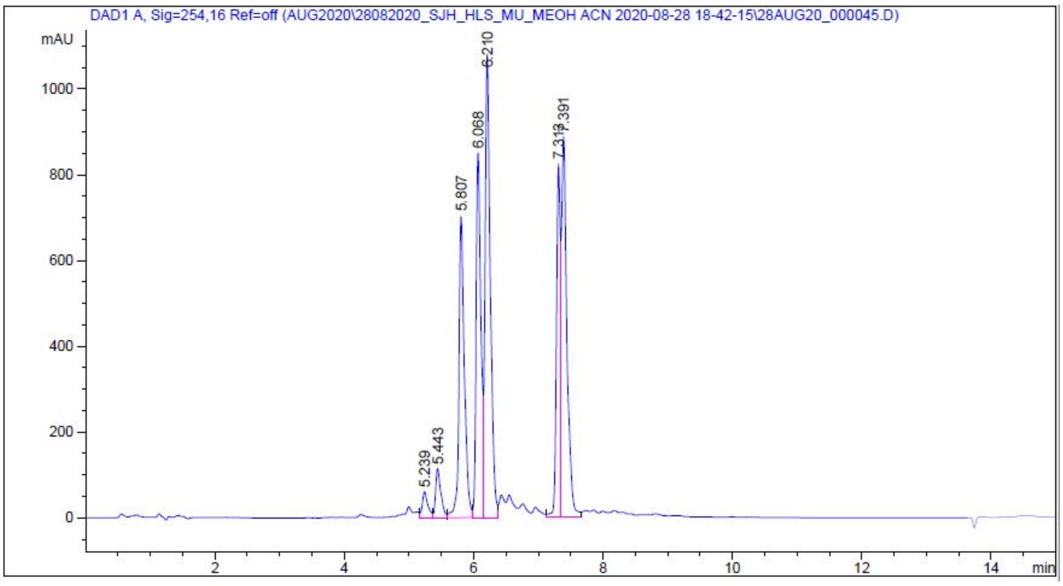

HPLC chromatogram of the resulting crude mixture after Corey-Chaykovski cyclopropanation applied to Set 2 in basic conditions.

HRMS Analysis of Set 1 After Diels/Alder with Cyclopentadiene

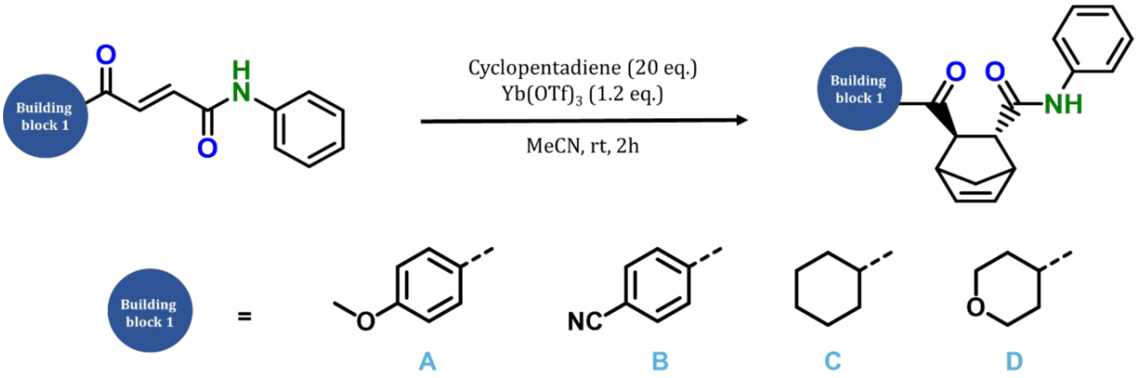

Product	Formula	Calculated mass $[M + H]^+$	Mass found $[M + H]^+$
A	$C_{22}H_{21}NO_3$	348.1597	348.1597
B	$C_{22}H_{18}N_2O_2$	343.1344	343.1437
C	$C_{21}H_{25}NO_2$	324.1961	324.1947
D	$C_{20}H_{23}NO_3$	326.1753	326.1755

HRMS Analysis of Set 2 After Diels/Alder with Cyclopentadiene

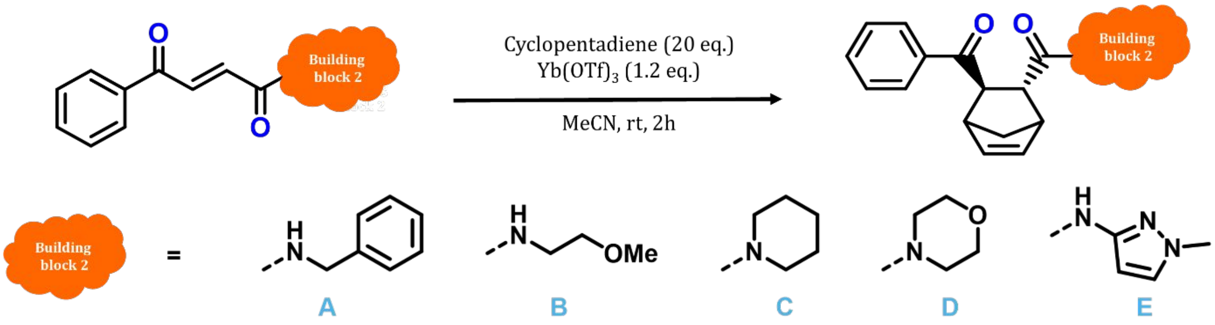

Product	Formula	Calculated mass $[M + H]^+$	Mass found $[M + H]^+$
A	$C_{22}H_{21}NO_2$	332.1648	332.1651
B	$C_{18}H_{21}NO_3$	300.1597	300.1586
C	$C_{20}H_{23}NO_2$	310.1804	310.1812
D	$C_{19}H_{21}NO_3$	312.1597	312.1585
E	$C_{19}H_{19}N_3O_2$	322.1553	322.1576

HPLC Analyses of Set 1 After the Diels-Alder Reaction with Cyclopentadiene Transformation

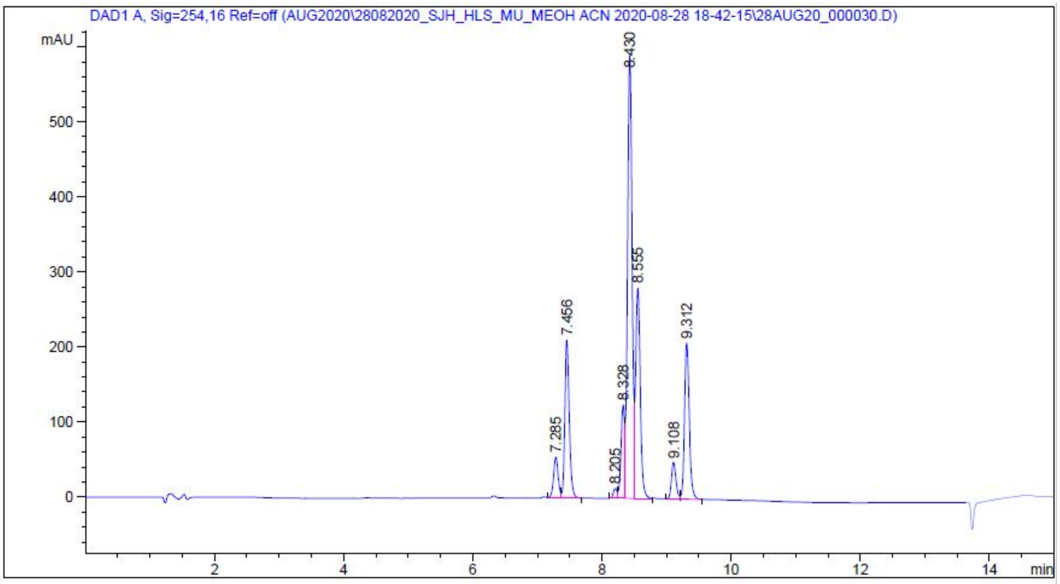

HPLC chromatogram of the resulting crude mixture after Diels-Alder reaction with cyclopentadiene applied to Set 1 in acidic conditions.

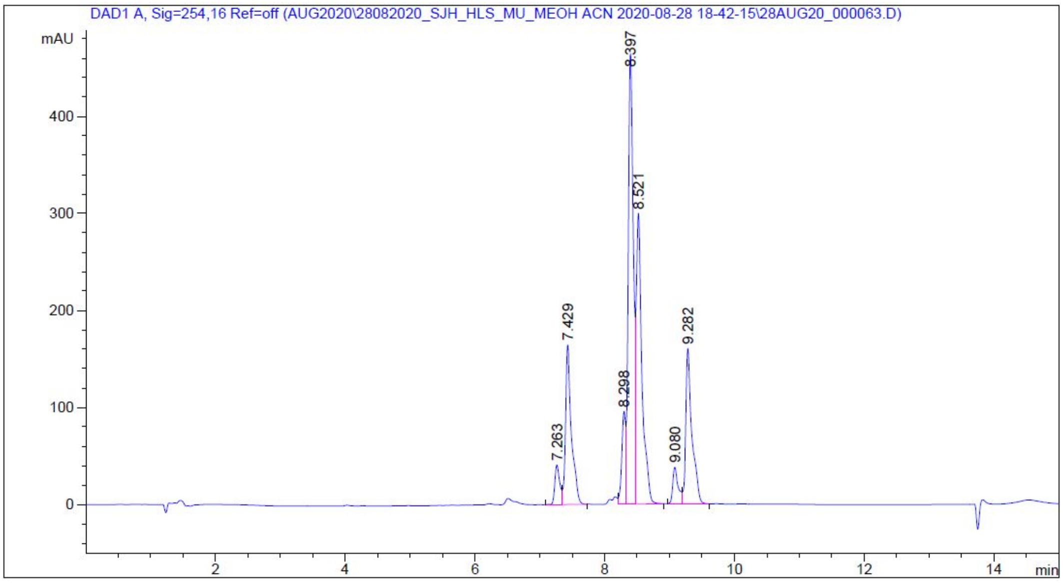

HPLC chromatogram of the resulting crude mixture after Diels-Alder reaction with cyclopentadiene applied to Set 1 in basic conditions.

HPLC Analyses of Set 2 After the Diels-Alder Reaction with Cyclopentadiene Transformation

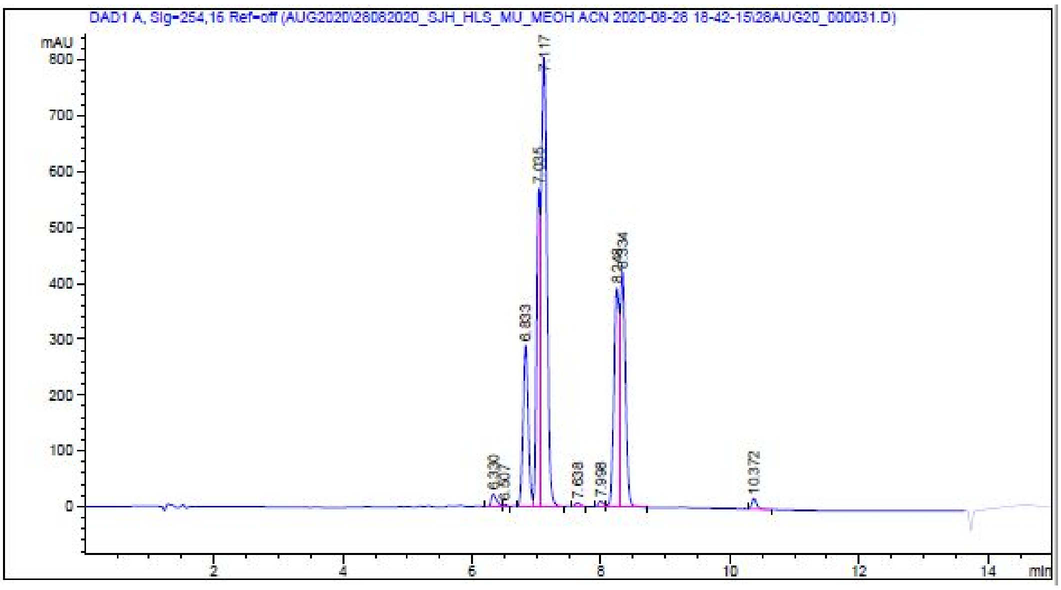

HPLC chromatogram of the resulting crude mixture after Diels-Alder reaction with cyclopentadiene applied to Set 2 in acidic conditions.

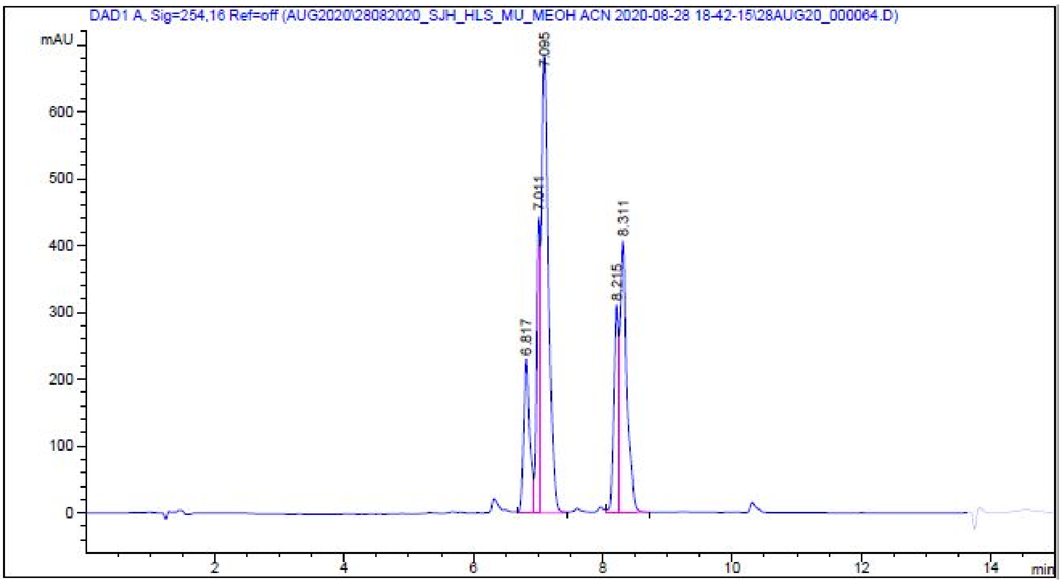

HPLC chromatogram of the resulting crude mixture after Diels-Alder reaction with cyclopentadiene applied to Set 2 in basic conditions.

HRMS Analysis of Set 1 After Diels/Alder with 2,3-Dimethyl-1,3-butadiene

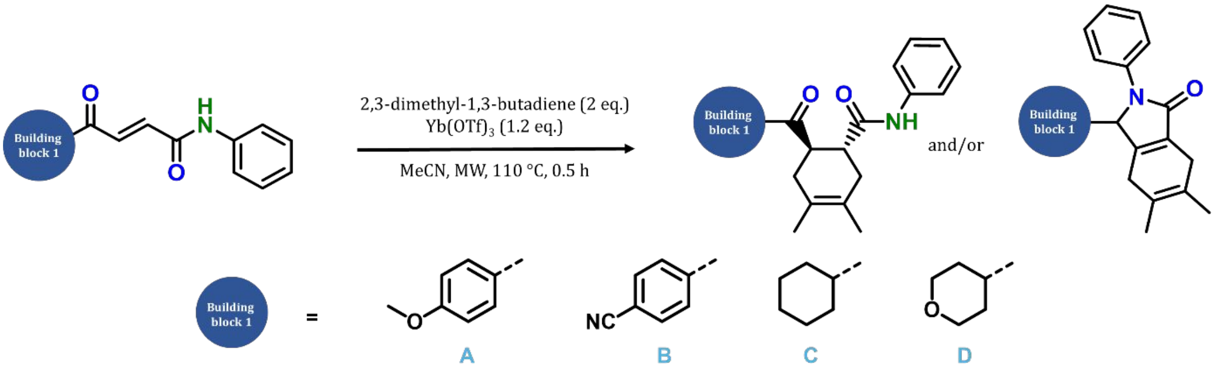

Product	Formula	Calculated mass [M + H]+	Mass found [M + H]+
A	$C_{23}H_{25}NO_3$	364.1910	364.1866
B	$C_{23}H_{22}N_2O_2$	359.1757	Not found
C	$C_{22}H_{29}NO_2$	340.2274	Not found
D	$C_{21}H_{27}NO_3$	342.2066	Not found
A cyclised	$C_{23}H_{23}NO_2$	346.1804	346.1816
B cylised	$C_{23}H_{20}N_2O$	341.1651	341.1649
C cyclised	$C_{21}H_{25}NO_2$	322.2169	322.2163
D cyclised	$C_{21}H_{25}NO_2$	324.1961	324.1984

HRMS Analysis of Set 2 After Diels/Alder with 2,3-Dimethyl-1,3-butadiene

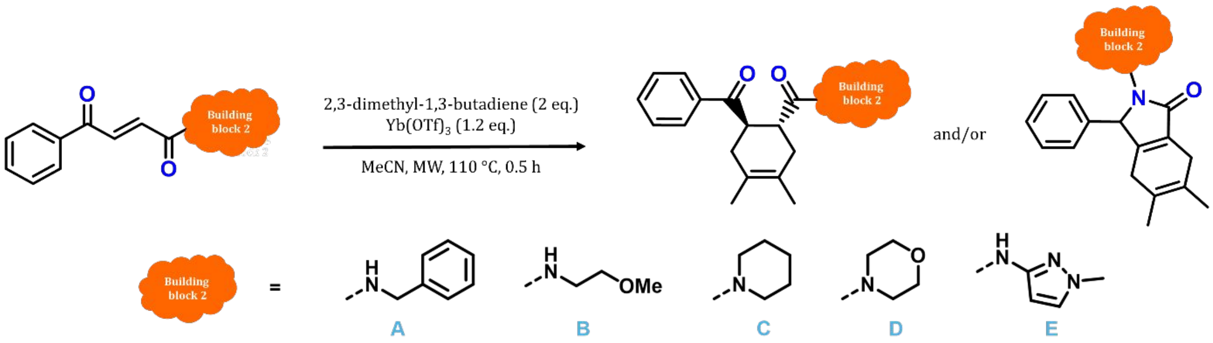

Product	Formula	Calculated mass [M + H]+	Mass found [M + H]+
A	$C_{23}H_{25}NO_2$	348.1961	348.1940
B	$C_{19}H_{25}NO_3$	316.1910	316.1938
C	$C_{21}H_{27}NO_2$	326.2117	326.2124
D	$C_{20}H_{25}NO_3$	328.1910	328.1882
E	$C_{20}H_{23}N_3O_2$	338.1866	338.1867
A cylised	$C_{23}H_{23}NO$	330.1855	330.1848
B cyclised	$C_{19}H_{23}NO_2$	298.1804	298.1809
E cyclised	$C_{20}H_{21}N_3O$	320.1760	320.1781

HPLC Analyses of Set 1 After the Diels-Alder Reaction with 2,3-Dimethyl-1,3-butadiene Transformation

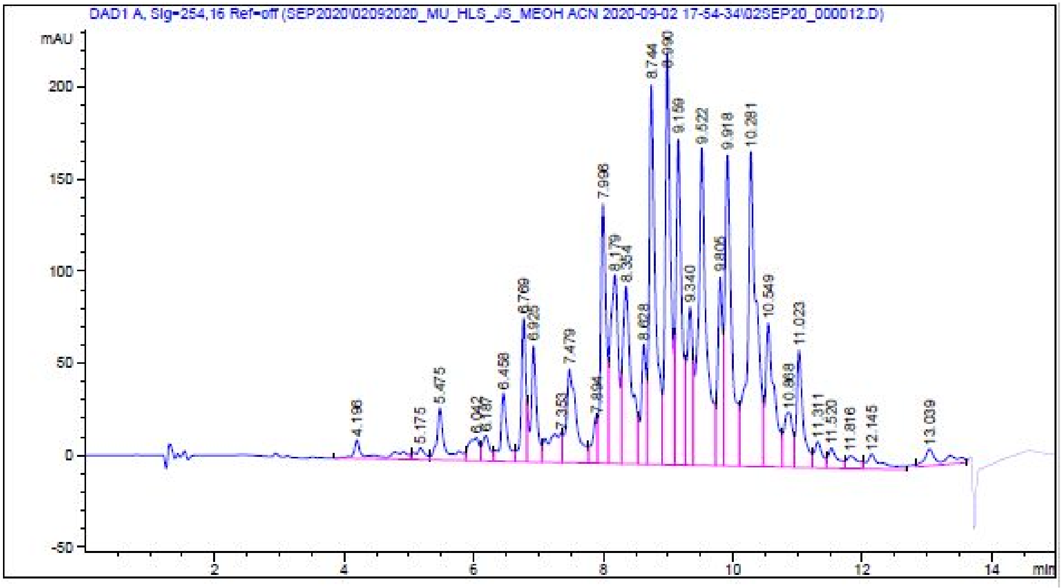

HPLC chromatogram of the resulting crude mixture after Diels-Alder reaction with 2,3-dimethyl-1,3-butadiene applied to Set 1 in acidic conditions.

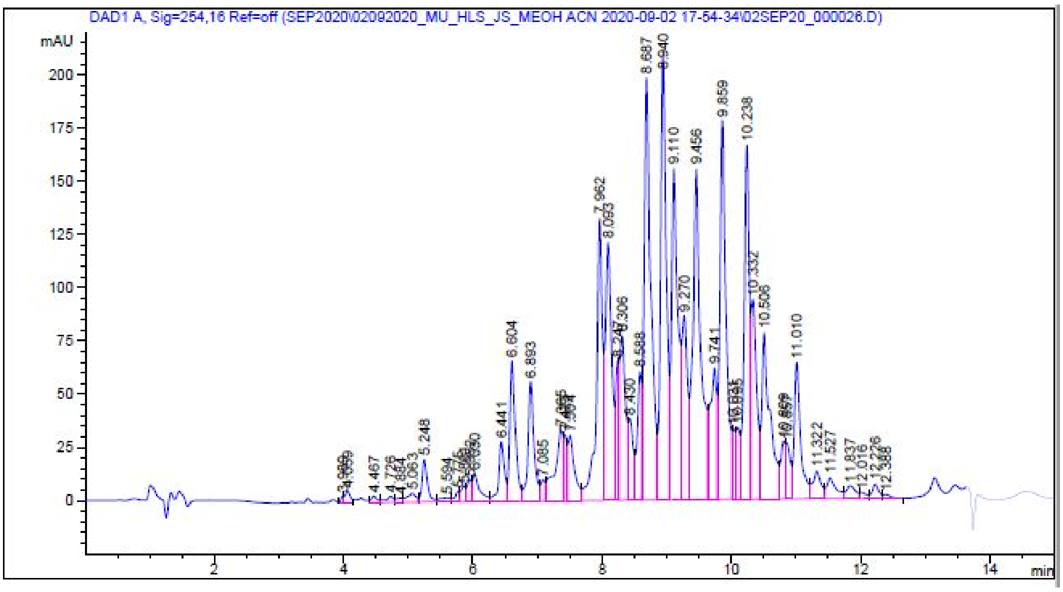

HPLC chromatogram of the resulting crude mixture after Diels-Alder reaction with 2,3-dimethyl-1,3-butadiene applied to Set 1 in basic conditions.

HPLC Analyses of Set 2 After the Diels-Alder Reaction with 2,3-Dimethyl-1,3-butadiene Transformation

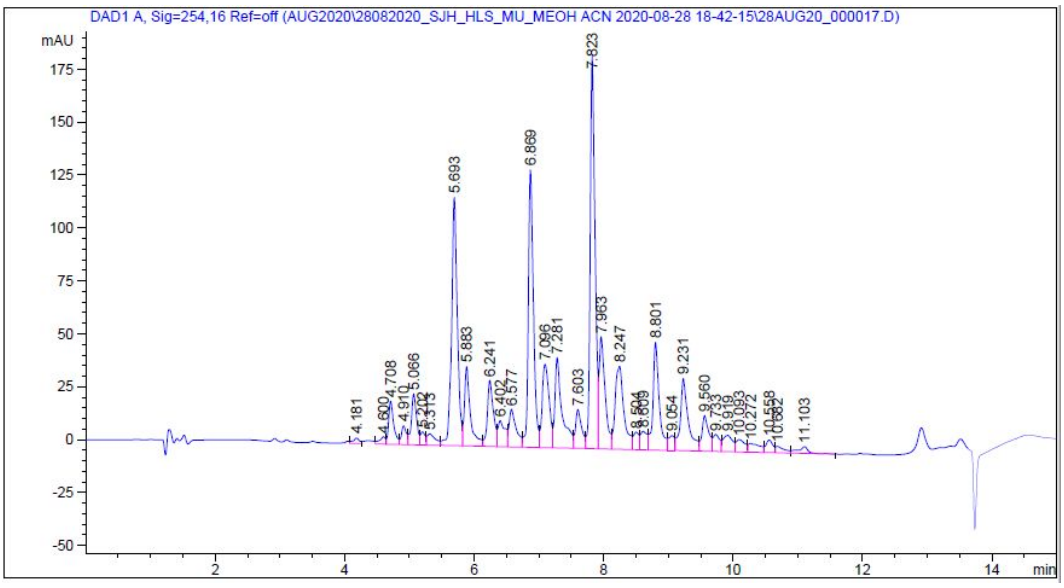

HPLC chromatogram of the resulting crude mixture after Diels-Alder reaction with 2,3-dimethyl-1,3-butadiene applied to Set 2 in acidic conditions.

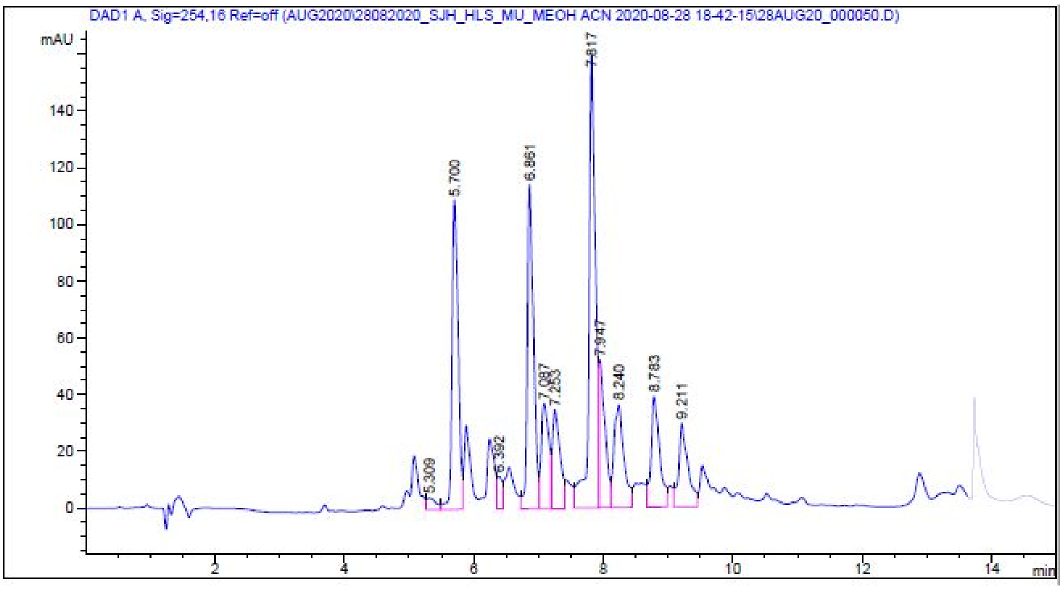

HPLC chromatogram of the resulting crude mixture after Diels-Alder reaction with 2,3-dimethyl-1,3-butadiene applied to Set 2 in basic conditions.

HRMS Analysis of Set 1 After Van Leusen Pyrrole Synthesis

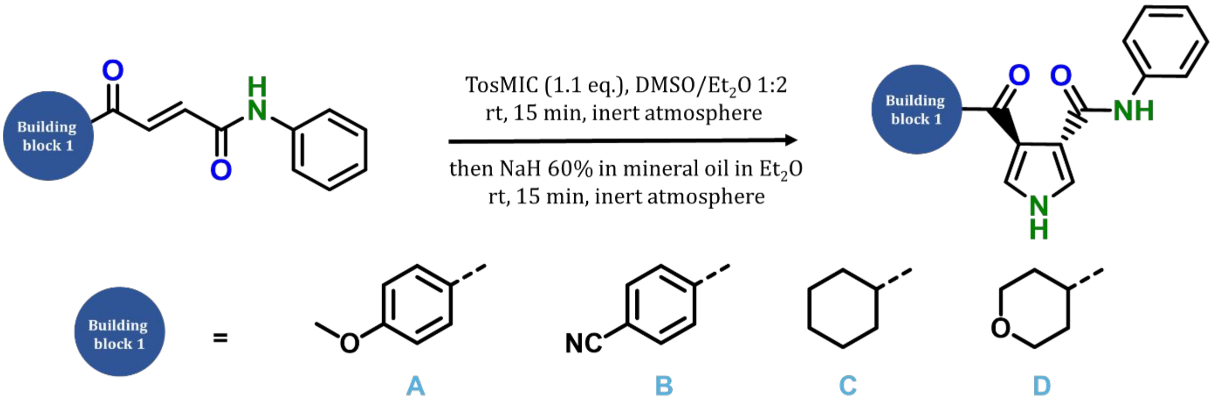

Product	Formula	Calculated mass $[M + H]^+$	Mass found $[M + H]^+$
A	$C_{19}H_{16}N_2O_3$	321.1236	321.1231
B	$C_{19}H_{13}N_3O_2$	316.1083	316.1065
C	$C_{18}H_{20}N_2O_2$	297.1600	297.1610
D	$C_{17}H_{18}N_2O_3$	299.1393	299.1386

HRMS Analysis of Set 2 After Van Leusen Pyrrole Synthesis

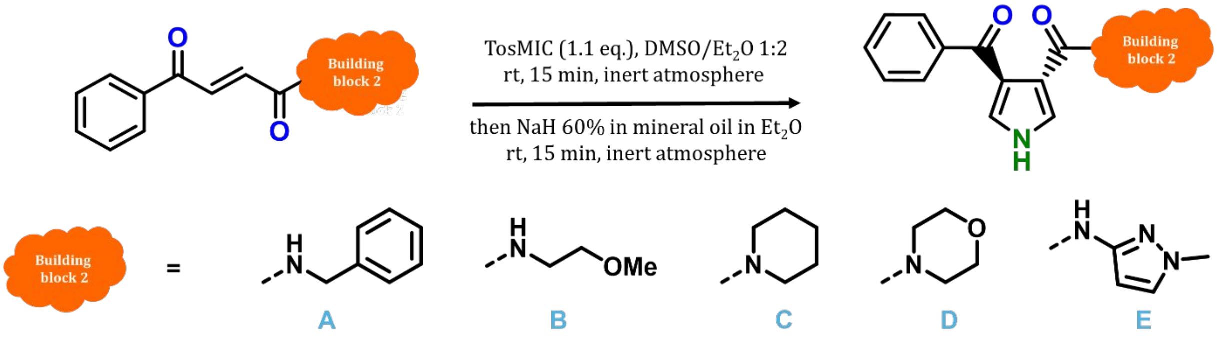

Product	Formula	Calculated mass $[M + H]^+$	Mass found $[M + H]^+$
A	$C_{19}H_{16}N_2O_2$	305.1287	305.1297
B	$C_{15}H_{16}N_2O_3$	273.1236	273.1230
C	$C_{17}H_{18}N_2O_2$	283.1444	283.1450
D	$C_{16}H_{16}N_2O_3$	285.1236	285.1241
E	$C_{16}H_{14}N_4O_2$	295.1192	295.1194

HPLC Analyses of Set 1 After the Van Leusen Pyrrole Synthesis Transformation

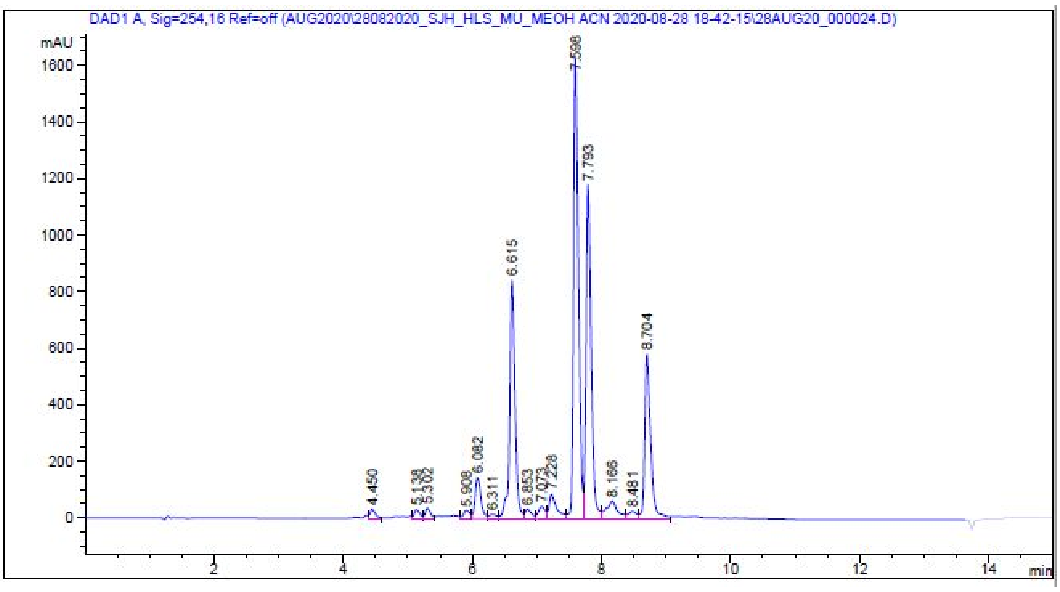

HPLC chromatogram of the resulting crude mixture after Van Leusen pyrrole synthesis applied to Set 1 in acidic conditions.

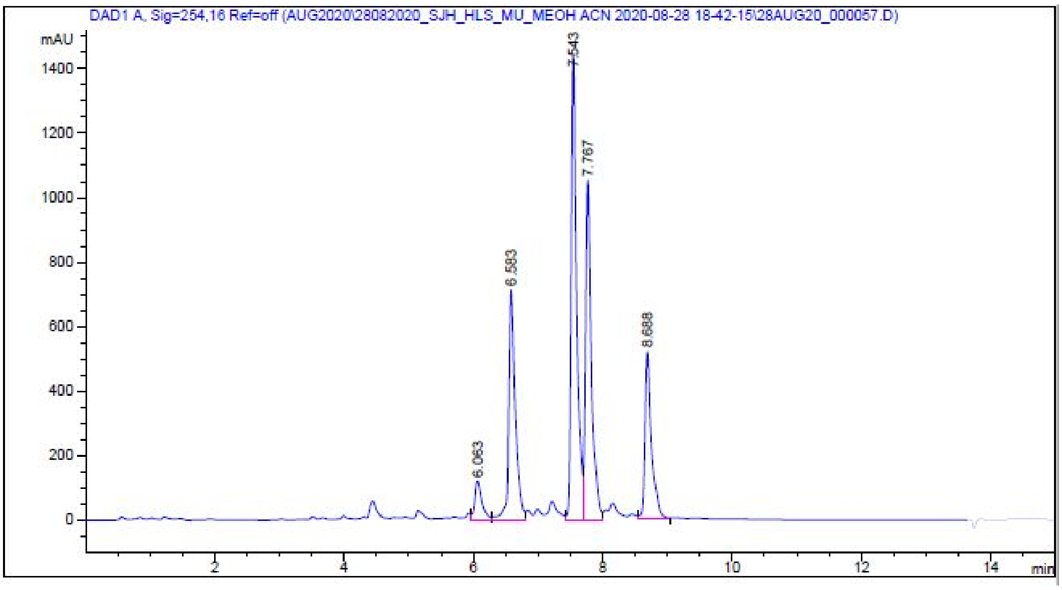

HPLC chromatogram of the resulting crude mixture after Van Leusen pyrrole synthesis applied to Set 1 in basic conditions.

HPLC Analyses of Set 2 After the Van Leusen Pyrrole Synthesis Transformation

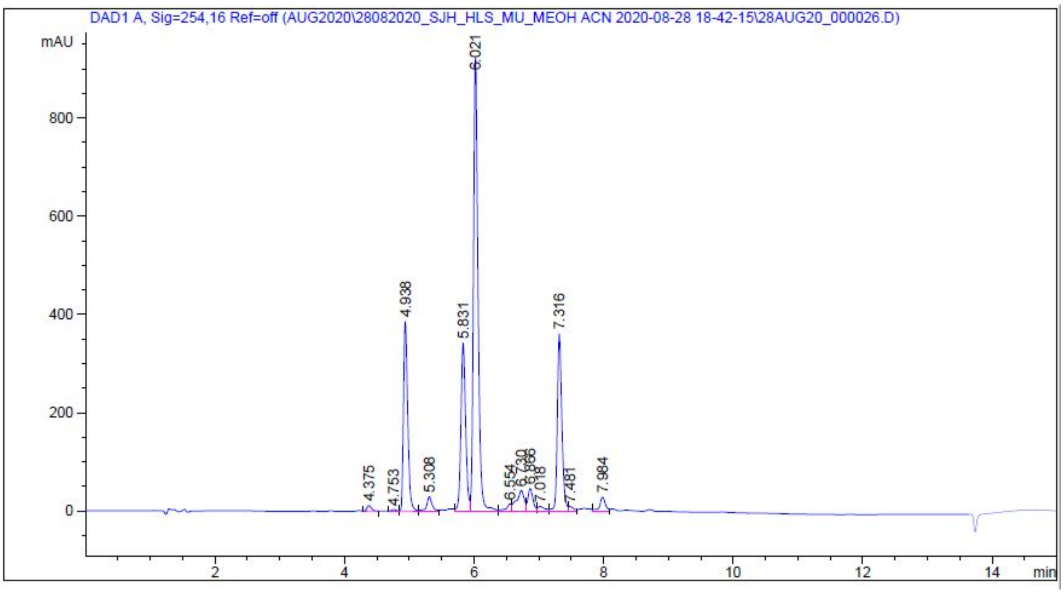

HPLC chromatogram of the resulting crude mixture after Van Leusen pyrrole synthesis applied to Set 2 in acidic conditions.

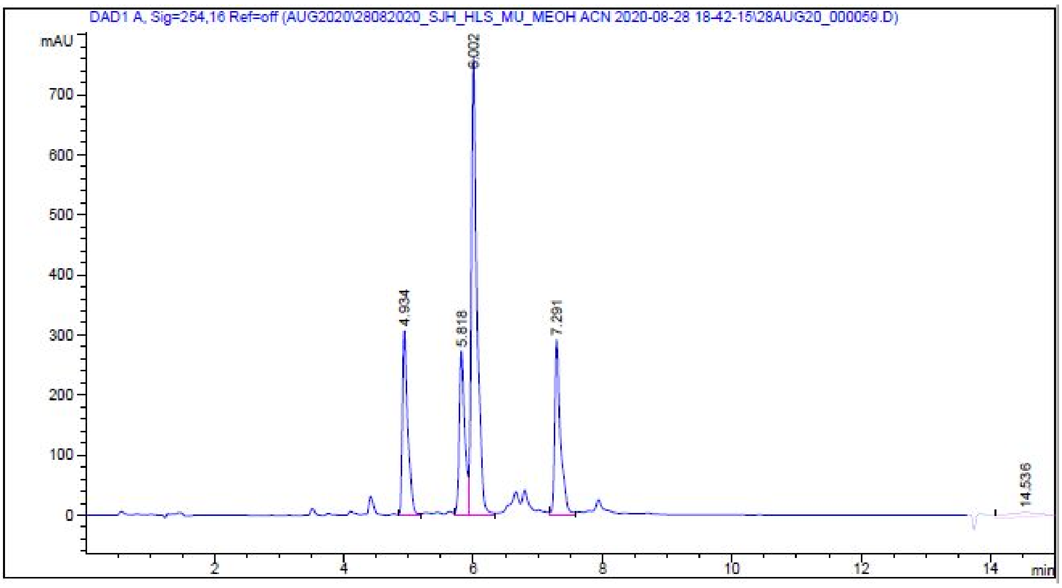

HPLC chromatogram of the resulting crude mixture after Van Leusen pyrrole synthesis applied to Set 2 in basic conditions.

HRMS Analysis of Set 1 After Benzyltriazole Formation

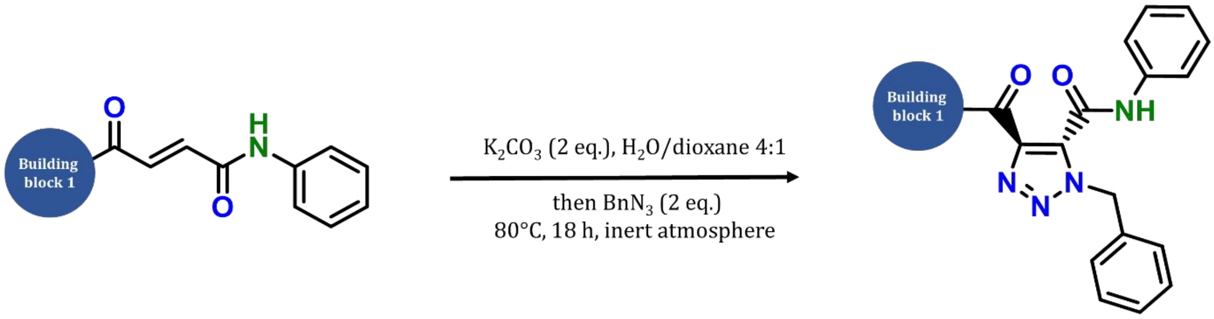

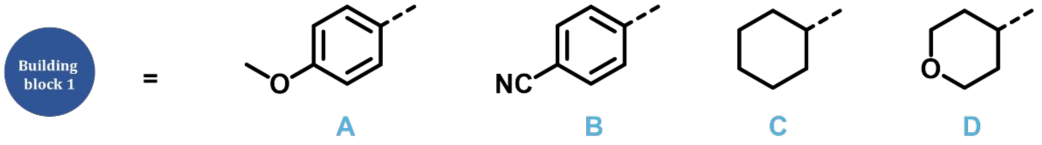

Product	Formula	Calculated mass [M + H]+	Mass found [M + H]+
A	$C_{24}H_{20}N_4O_3$	413.1611	413.1582
B	$C_{24}H_{17}N_5O_2$	408.1458	Not found
C	$C_{23}H_{24}N_4O_2$	389.1975	Not found
D	$C_{22}H_{22}N_4O_3$	391.1767	Not found

HRMS Analysis of Set 2 After Benzyltriazole Formation

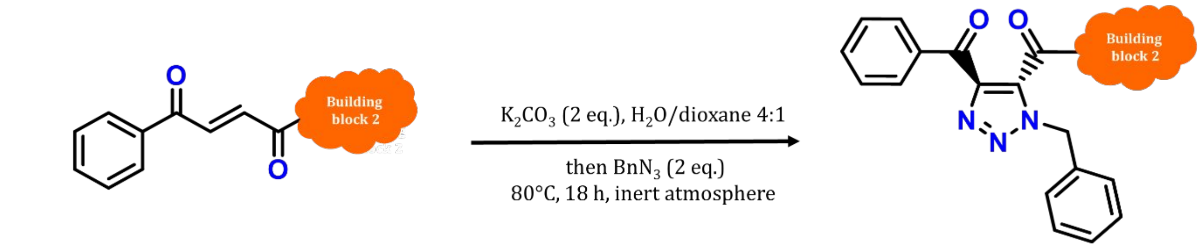

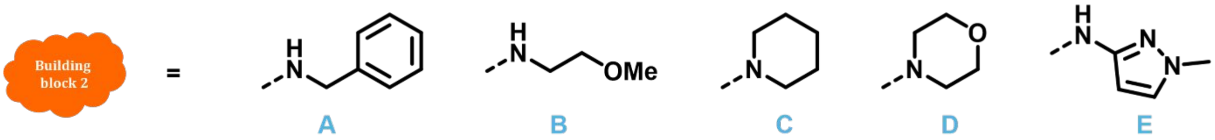

Product	Formula	Calculated mass [M + H]+	Mass found [M + H]+
A	$C_{24}H_{20}N_4O_2$	396.1586	397.1676
B	$C_{20}H_{20}N_4O_3$	365.1611	365.1592
C	$C_{22}H_{22}N_4O_2$	375.1818	375.1826
D	$C_{21}H_{20}N_4O_3$	377.1611	377.1622
E	$C_{21}H_{18}N_6O_2$	387.1567	387.1547

HPLC Analyses of Set 1 After the Benzyltriazole Formation Transformation

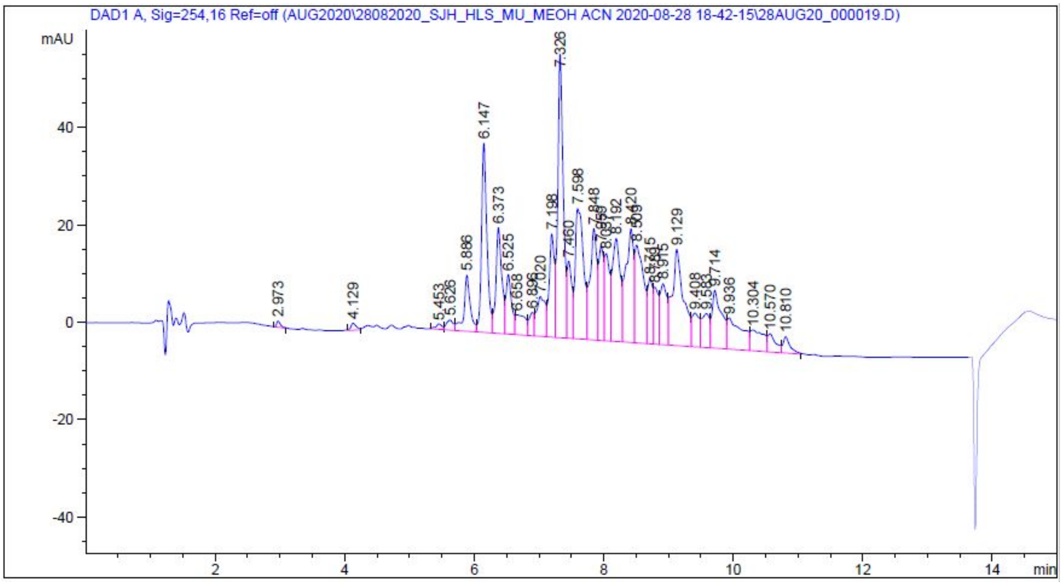

HPLC chromatogram of the resulting crude mixture after benzyltriazole formation applied to Set 1 in acidic conditions.

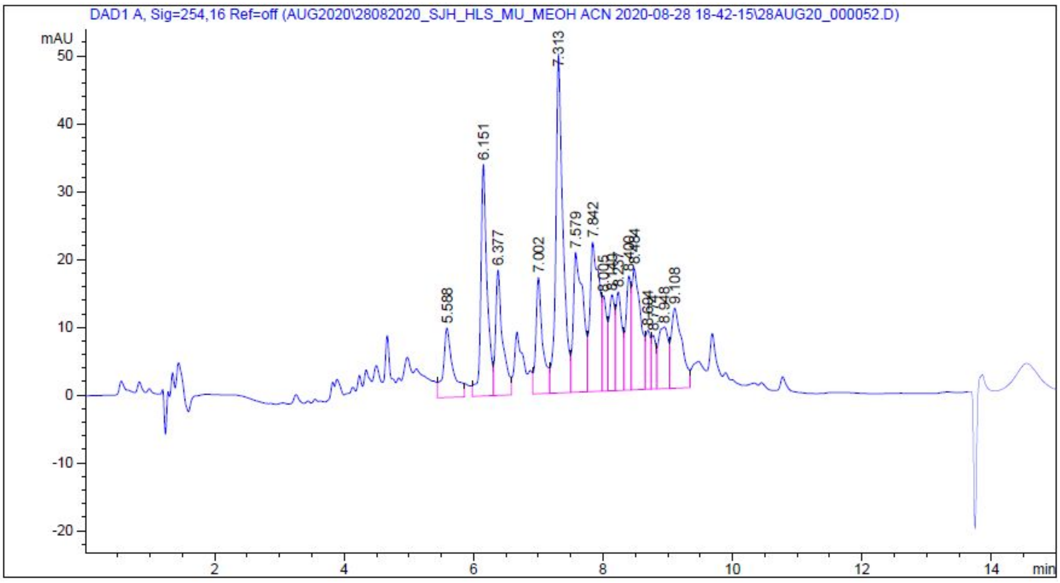

HPLC chromatogram of the resulting crude mixture after benzyltriazole formation applied to Set 1 in basic conditions.

HPLC Analyses of Set 2 After the Benzyltriazole Formation Transformation

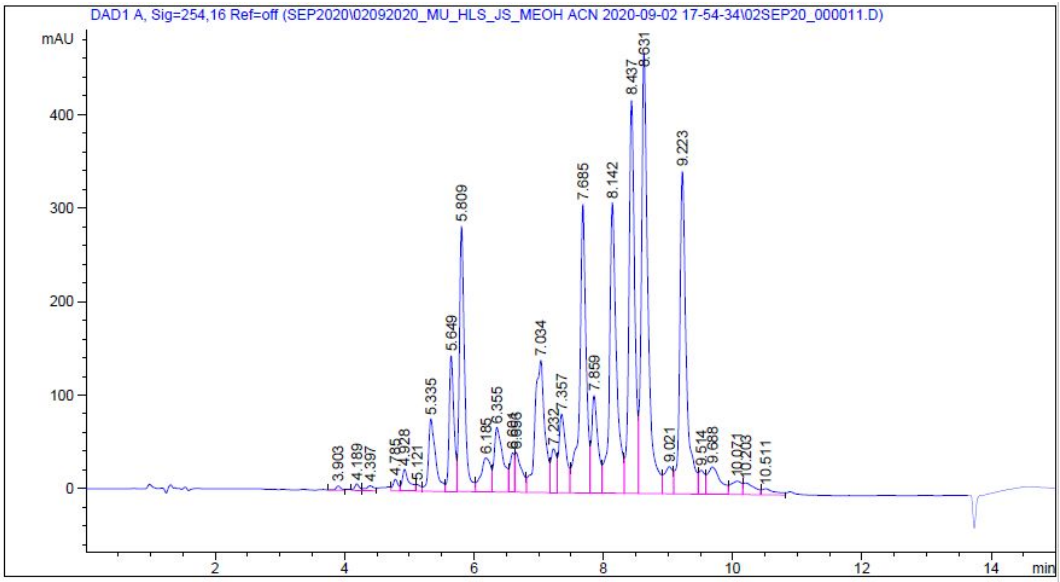

HPLC chromatogram of the resulting crude mixture after benzyltriazole formation applied to Set 2 in acidic conditions.

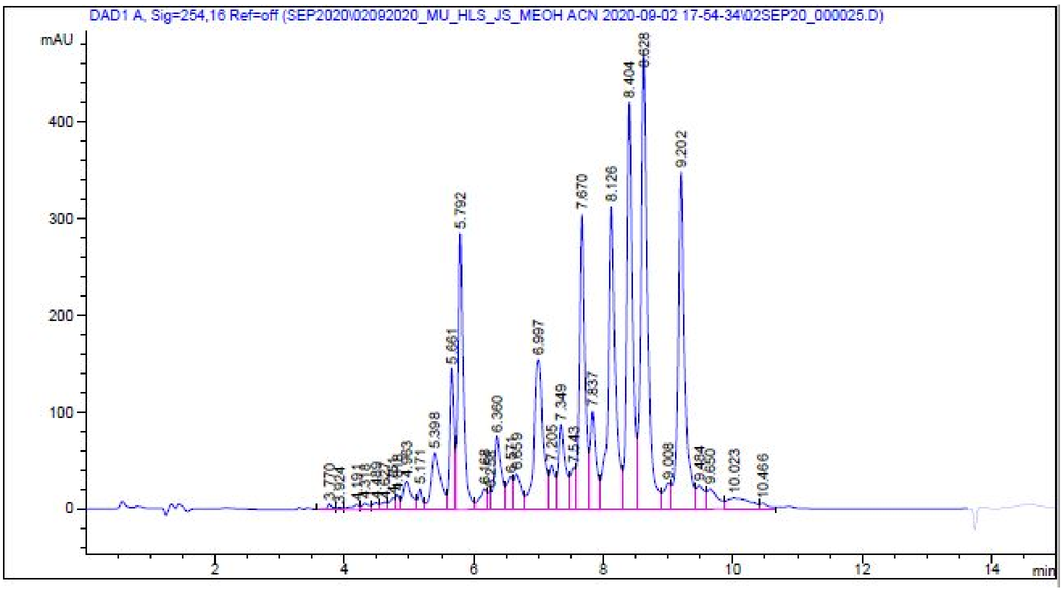

HPLC chromatogram of the resulting crude mixture after benzyltriazole formation applied to Set 2 in basic conditions.

HRMS Analysis of Set 1 After Triazole Formation

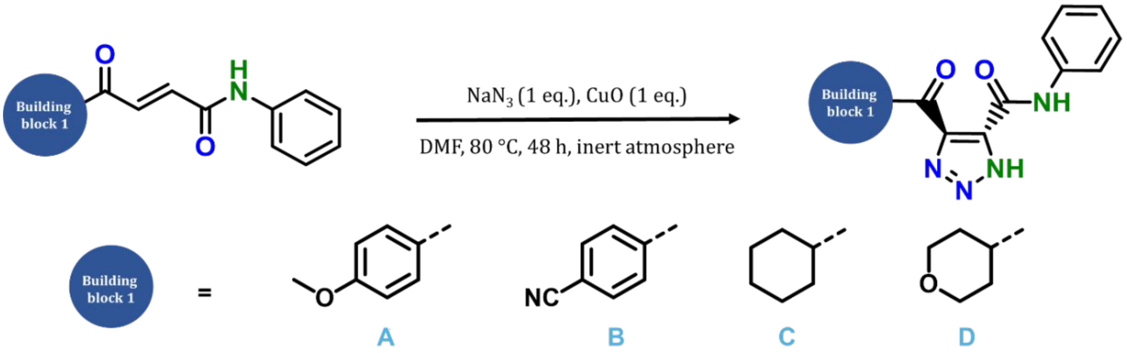

Product	Formula	Calculated mass [M + H]+	Mass found [M + H]+
A	$C_{17}H_{14}N_4O_3$	323.1141	323.1128
B	$C_{17}H_{11}N_5O_2$	318.0988	318.0989
C	$C_{16}H_{18}N_4O_2$	299.1505	299.1495
D	$C_{15}H_{16}N_4O_3$	301.1298	301.1304

HRMS Analysis of Set 2 After Triazole Formation

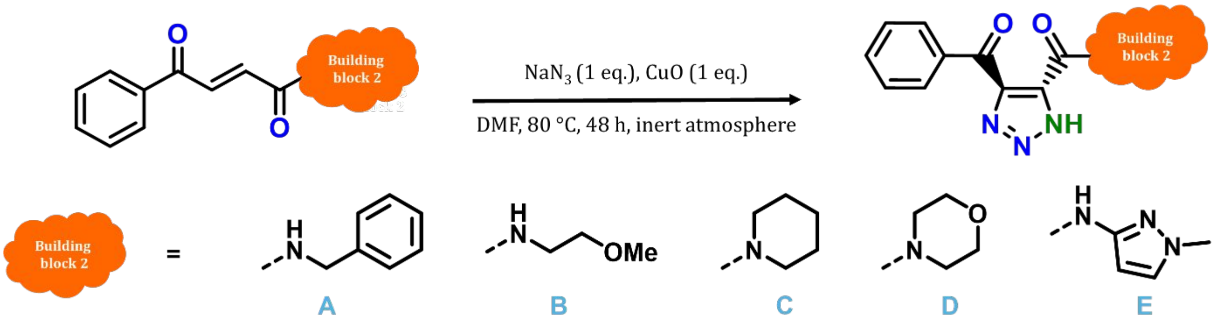

Product	Formula	Calculated mass [M + H]+	Mass found [M + H]+
A	$C_{17}H_{14}N_4O_2$	307.1192	307.1159
B	$C_{13}H_{14}N_4O_3$	275.1141	275.1143
C	$C_{15}H_{16}N_4O_2$	285.1349	285.1345
D	$C_{14}H_{14}N_4O_3$	287.1141	Not found
E	$C_{14}H_{12}N_6O_2$	297.1097	297.094

HPLC Analyses of Set 1 After the Triazole Formation Transformation

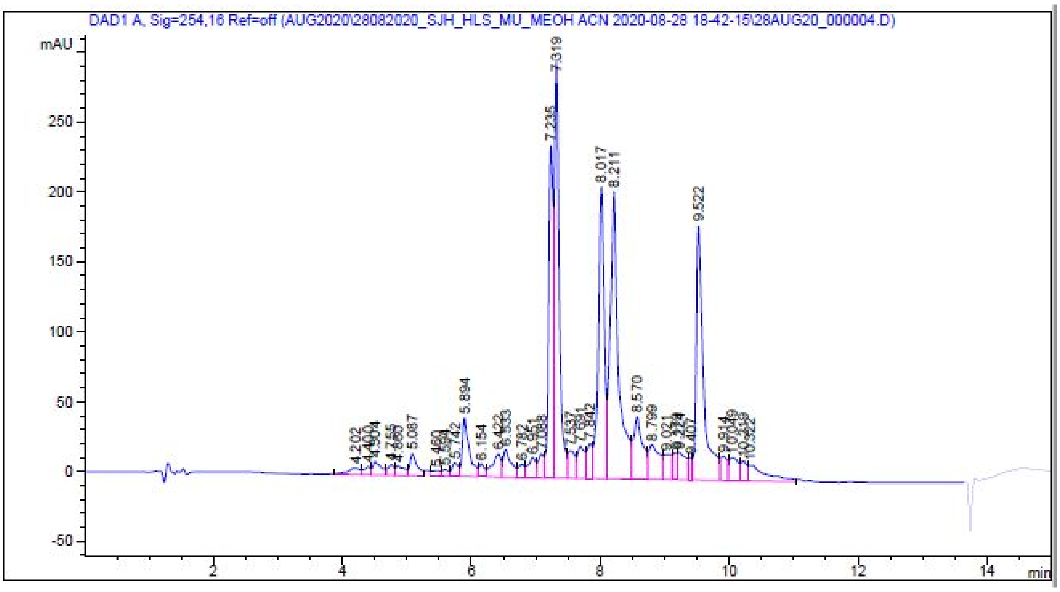

HPLC chromatogram of the resulting crude mixture after triazole formation applied to Set 1 in acidic conditions.

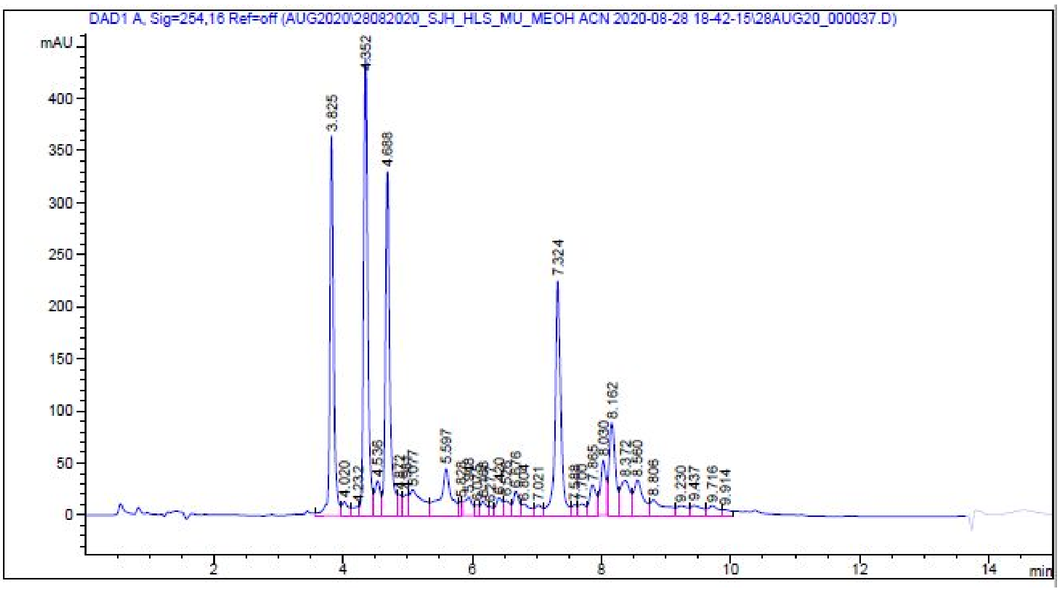

HPLC chromatogram of the resulting crude mixture after triazole formation applied to Set 1 in basic conditions.

HPLC Analyses of Set 2 After the Triazole Formation Transformation

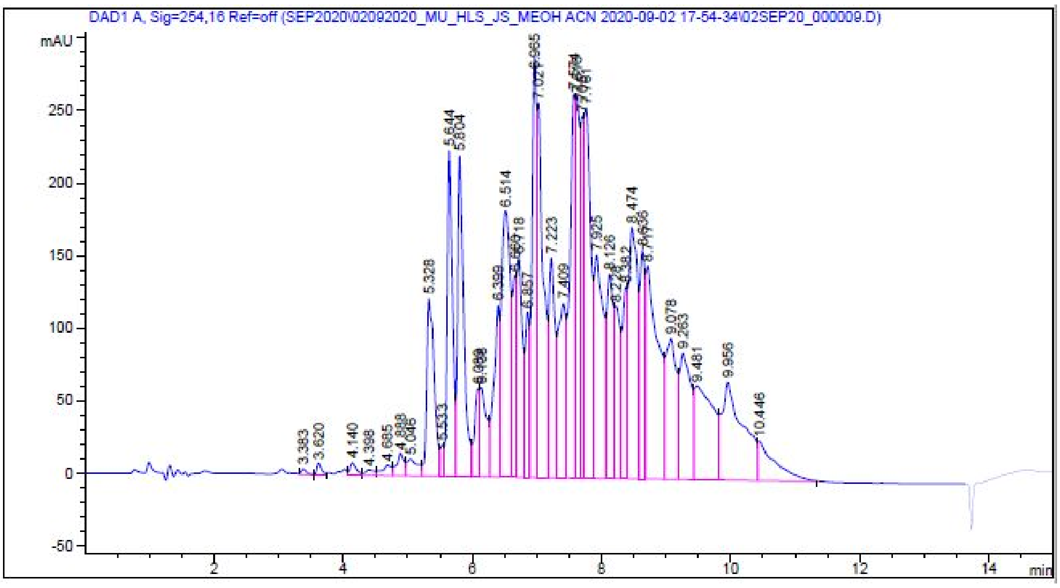

HPLC chromatogram of the resulting crude mixture after triazole formation applied to Set 2 in acidic conditions.

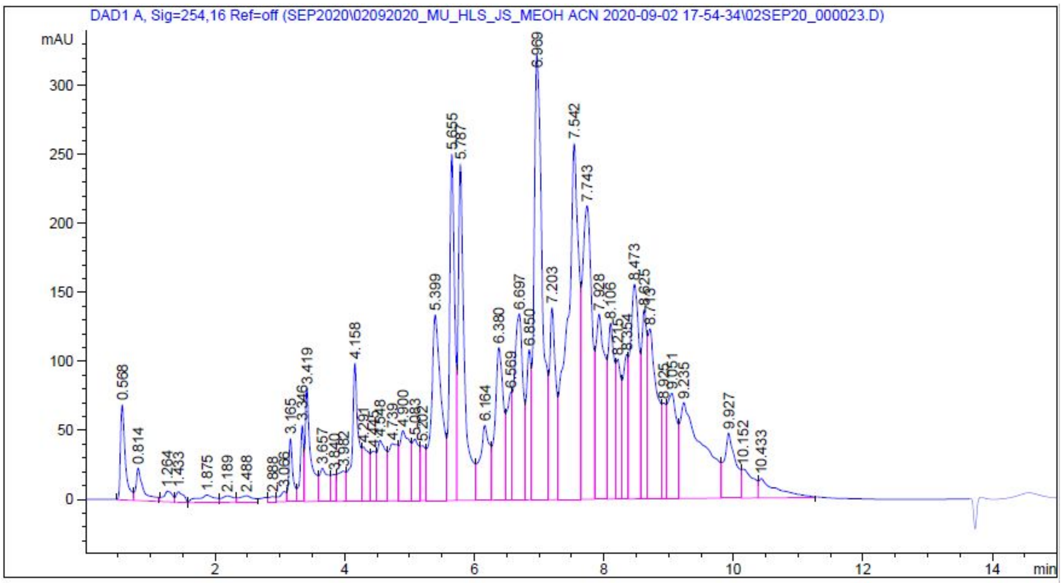

HPLC chromatogram of the resulting crude mixture after triazole formation applied to Set 2 in basic conditions.

HRMS Analysis of Set 1 After Isoxazolidine Formation

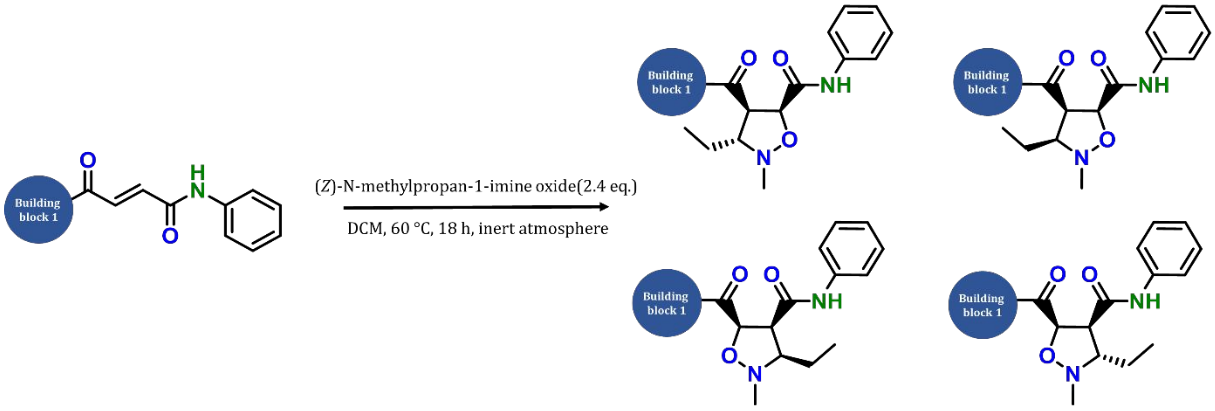

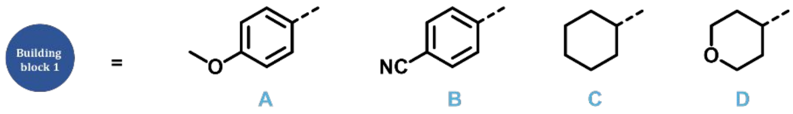

Product	Formula	Calculated mass [M + H]+	Mass found [M + H]+
A	$C_{21}H_{24}N_2O_4$	369.1812	369.1806
B	$C_{21}H_{21}N_3O_3$	364.1658	364.1671
C	$C_{20}H_{28}N_2O_3$	345.2175	345.2194
D	$C_{19}H_{26}N_2O_4$	347.1968	347.1947

HRMS Analysis of Set 2 After Isoxazolidine Formation

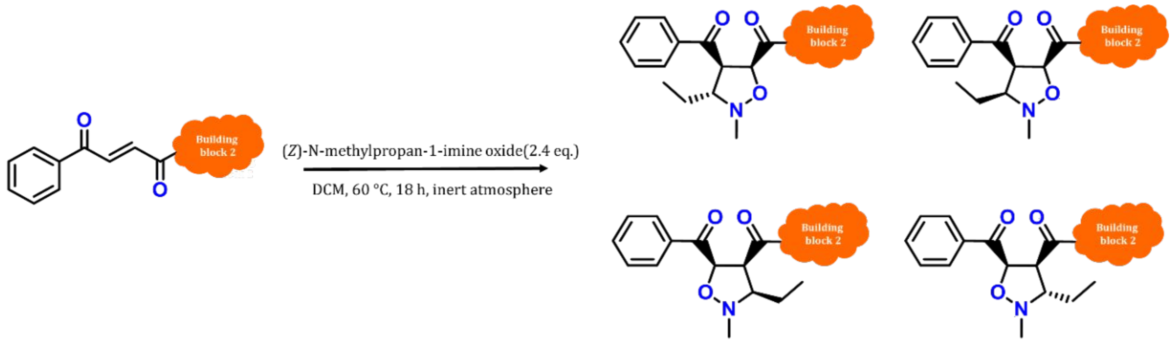

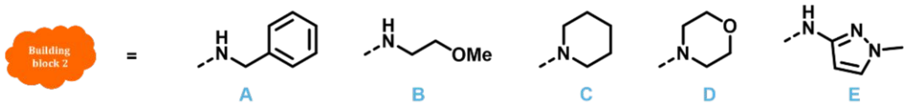

Product	Formula	Calculated mass [M + H]+	Mass found [M + H]+
A	$C_{21}H_{24}N_2O_3$	353.1862	353.1851
B	$C_{17}H_{24}N_2O_4$	321.1812	321.1781
C	$C_{19}H_{26}N_2O_3$	331.2019	331.2003
D	$C_{18}H_{24}N_2O_4$	333.1812	333.1799
E	$C_{18}H_{22}N_4O_3$	343.1767	343.1740

HPLC Analyses of Set 1 After the Isoxazolidine Formation Transformation

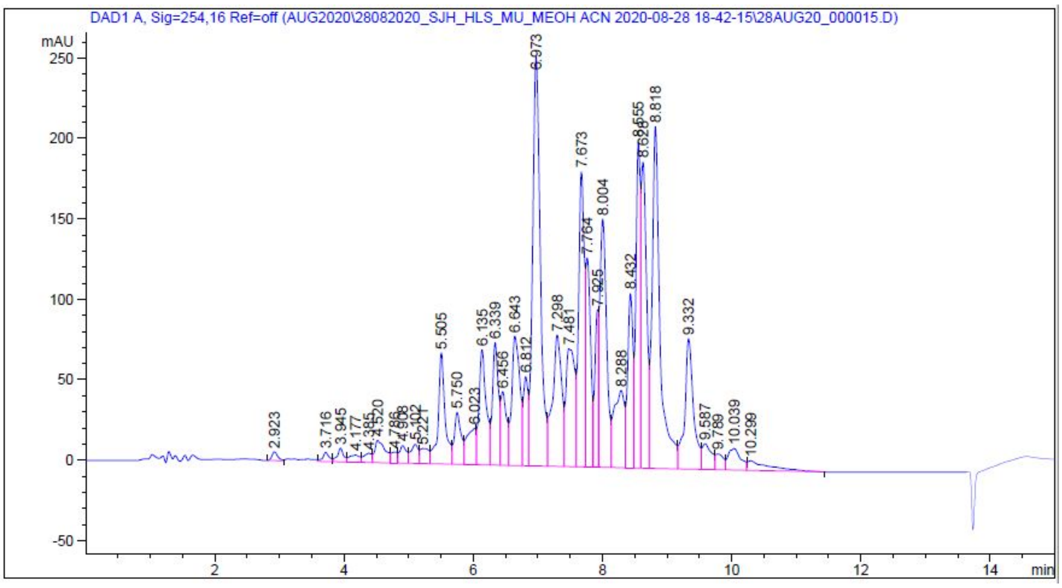

HPLC chromatogram of the resulting crude mixture after isoxazolidine formation applied to Set 1 in acidic conditions.

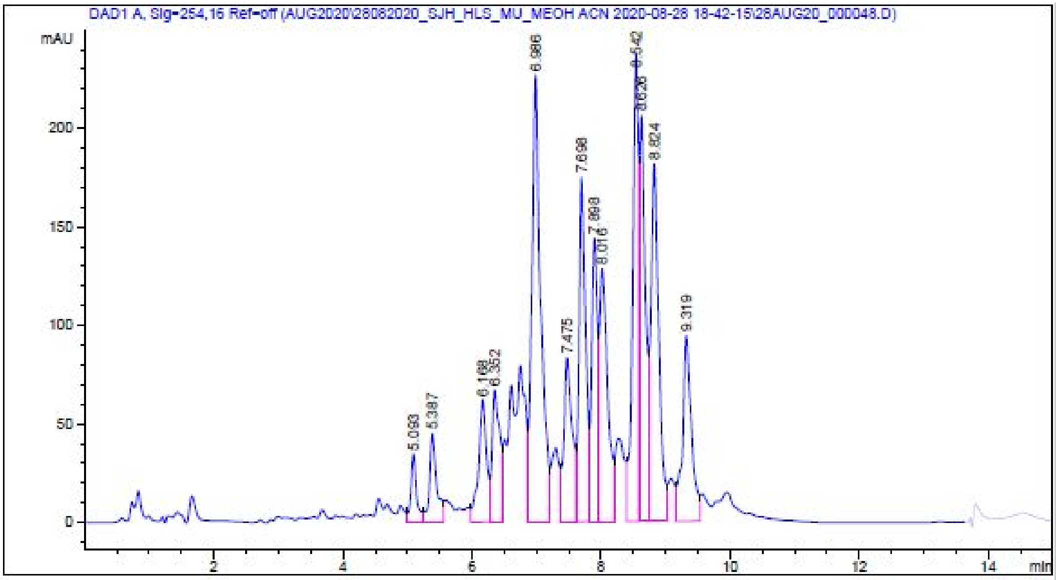

HPLC chromatogram of the resulting crude mixture after isoxazolidine formation applied to Set 1 in basic conditions.

HPLC Analyses of Set 2 After the Isoxazolidine Formation Transformation

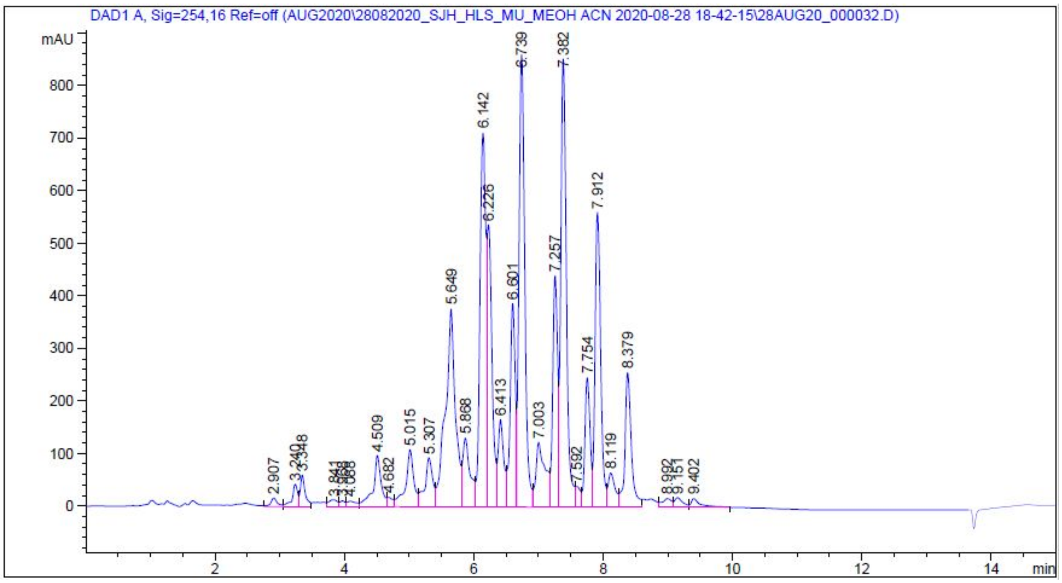

HPLC chromatogram of the resulting crude mixture after isoxazolidine formation applied to Set 2 in acidic conditions.

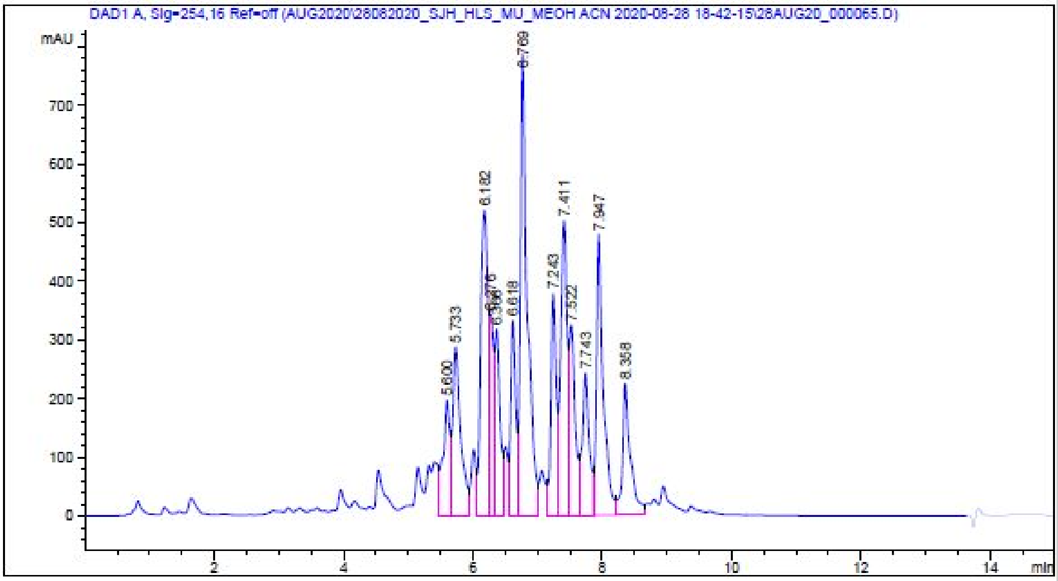

HPLC chromatogram of the resulting crude mixture after isoxazolidine formation applied to Set 2 in basic conditions.

3.2.3. Transformations: Reductions

HRMS Analysis of Set 1 After Hydrogenation

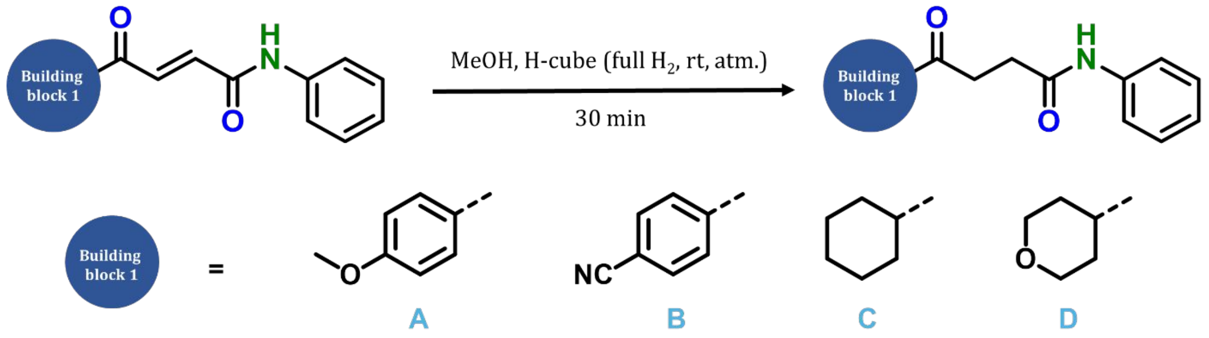

Product	Formula	Calculated mass [M + H]$^+$	Mass found [M + H]$^+$
A	$C_{17}H_{17}NO_3$	284.1284	284.1268
B	$C_{17}H_{14}N_2O_2$	279.1131	Not found
C	$C_{16}H_{21}NO_2$	260.1648	260.1645
D	$C_{15}H_{19}NO_3$	262.1340	262.1809
B reduced CN	$C_{17}H_{16}N_2O_2$	281.1288	281.1145

HRMS Analysis of Set 2 After Hydrogenation

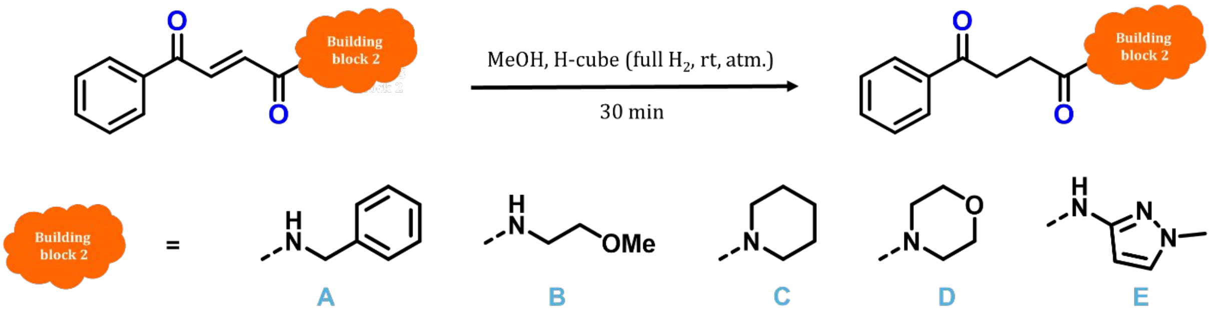

Product	Formula	Calculated mass [M + H]$^+$	Mass found [M + H]$^+$
A	$C_{17}H_{17}NO_2$	268.1335	268.1329
B	$C_{13}H_{17}NO_3$	236.1284	236.1278
C	$C_{15}H_{19}NO_2$	246.1491	246.1486
D	$C_{14}H_{17}NO_3$	248.1284	248.1262
E	$C_{14}H_{15}N_3O_2$	258.1240	258.1098

HPLC Analyses of Set 1 After the Hydrogenation Transformation

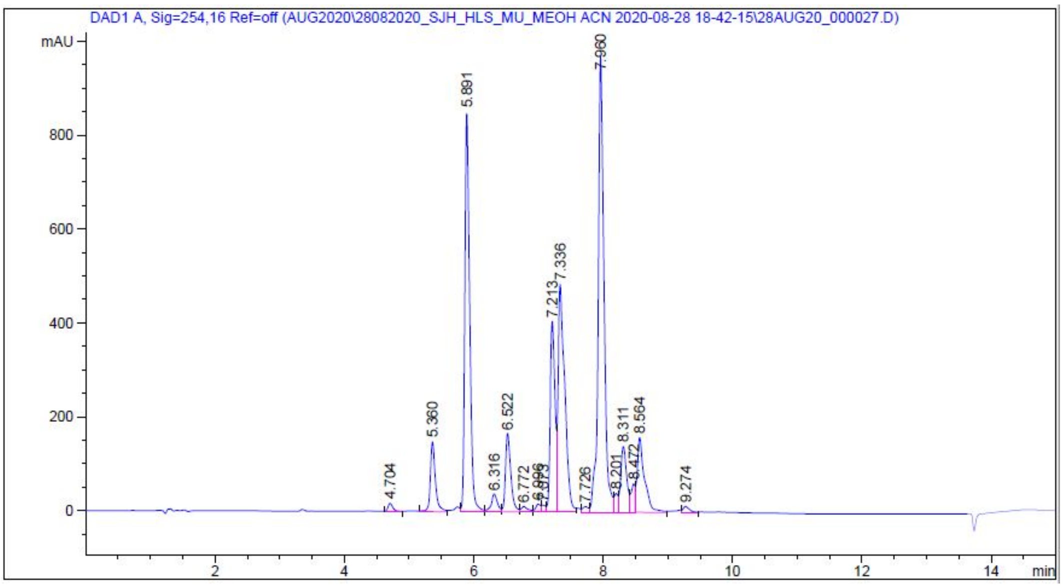

HPLC chromatogram of the resulting crude mixture after hydrogenation of Set 1 in acidic conditions.

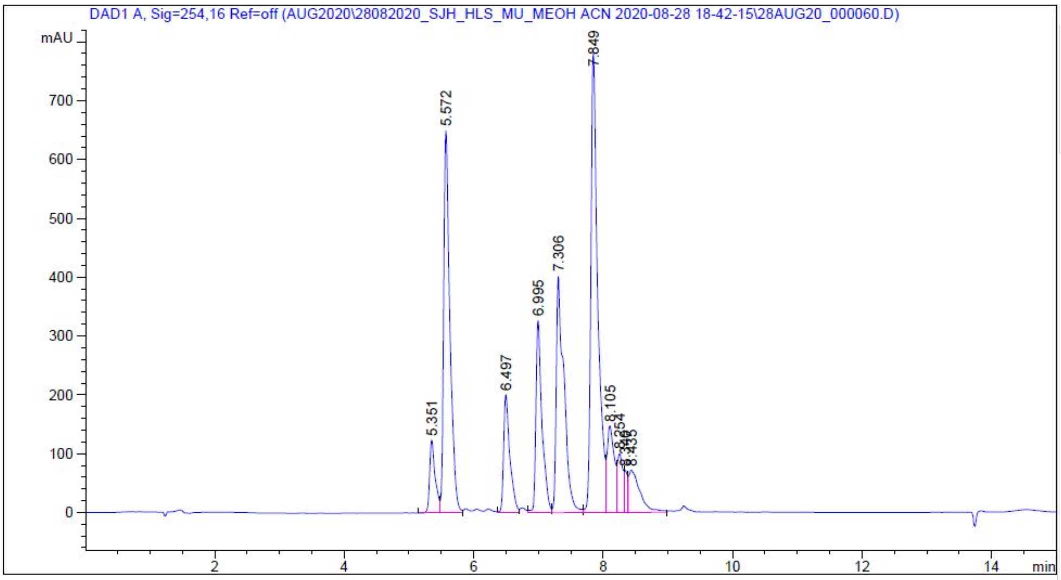

HPLC chromatogram of the resulting crude mixture after hydrogenation of Set 1 in basic conditions.

HPLC Analyses of Set 2 After the Hydrogenation Transformation

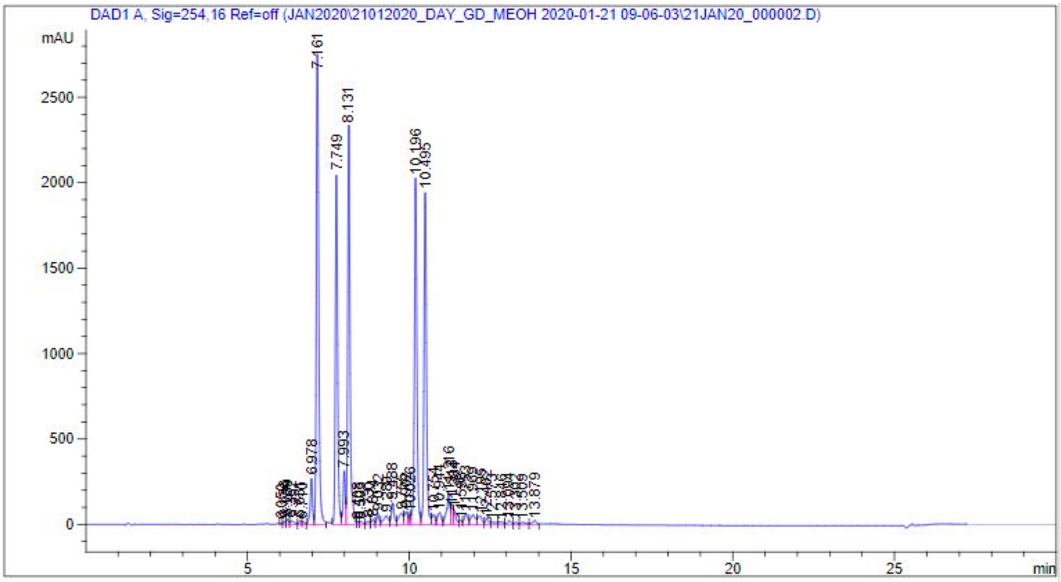

HPLC chromatogram of the resulting crude mixture after hydrogenation of Set 2 in acidic conditions.

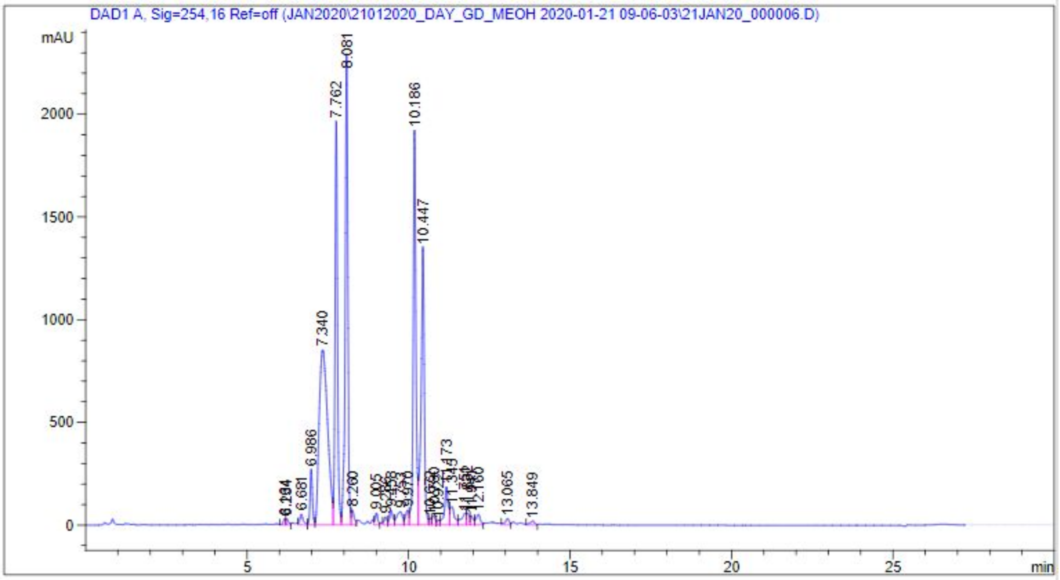

HPLC chromatogram of the resulting crude mixture after hydrogenation of Set 2 in basic conditions.

HRMS Analysis of Set 1 After Luche Reduction

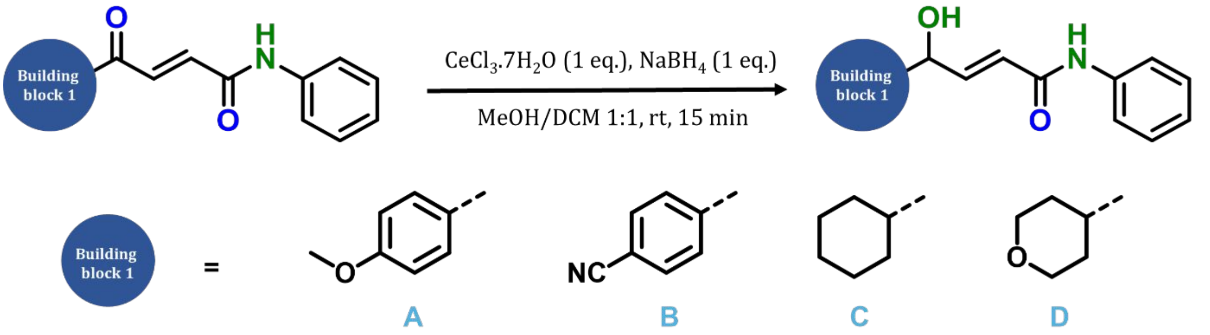

Product	Formula	Calculated mass [M + H]$^+$	Mass found [M + H]$^+$
A	$C_{17}H_{17}NO_3$	284.1284	284.1268
B	$C_{17}H_{14}N_2O_2$	279.1131	279.1152
C	$C_{16}H_{21}NO_2$	260.1648	260.1645
D	$C_{15}H_{19}NO_3$	262.1440	262.1411

HRMS Analysis of Set 2 After Luche Reduction

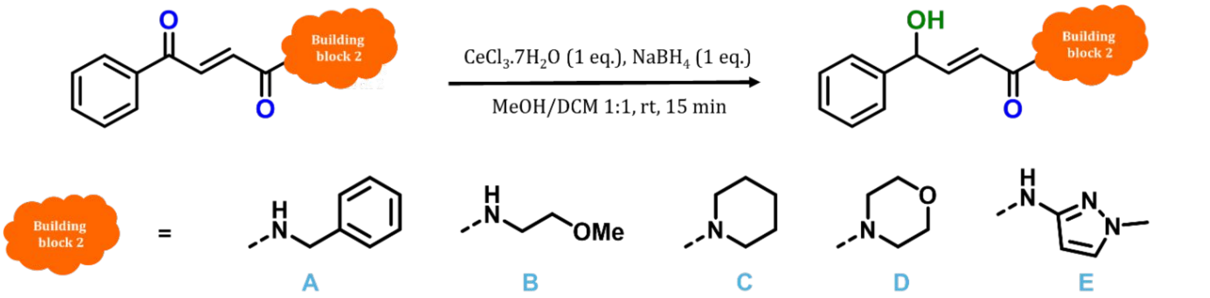

Product	Formula	Calculated mass [M + H]$^+$	Mass found [M + H]$^+$
A	$C_{17}H_{17}NO_2$	268.1335	268.1329
B	$C_{13}H_{17}NO_3$	236.1284	236.1278
C	$C_{15}H_{19}NO_2$	246.1291	246.1518
D	$C_{14}H_{17}NO_3$	248.1284	248.1294
E	$C_{14}H_{15}N_3O_2$	258.1240	258.1229

HPLC Analyses of Set 1 After the Luche Reduction Transformation

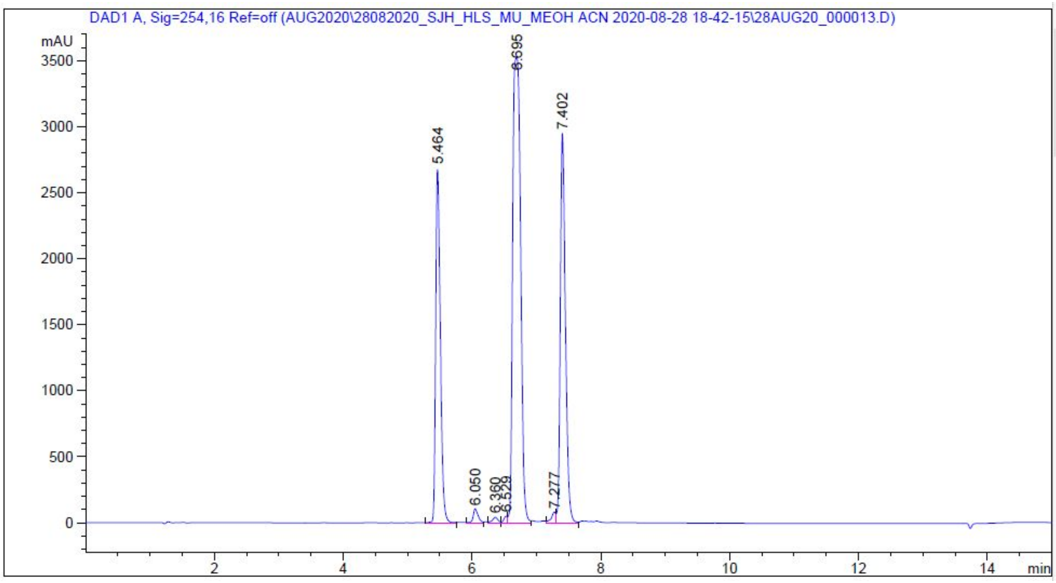

HPLC chromatogram of the resulting crude mixture after Luche reduction of Set 1 in acidic conditions.

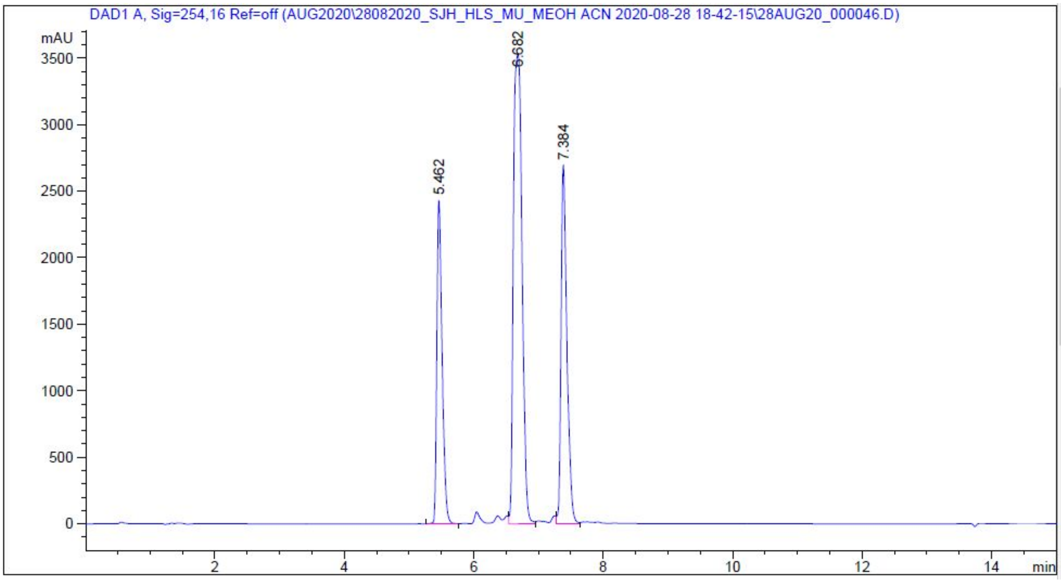

HPLC chromatogram of the resulting crude mixture after Luche reduction of Set 1 in basic conditions.

HPLC Analyses of Set 2 After the Luche Reduction Transformation

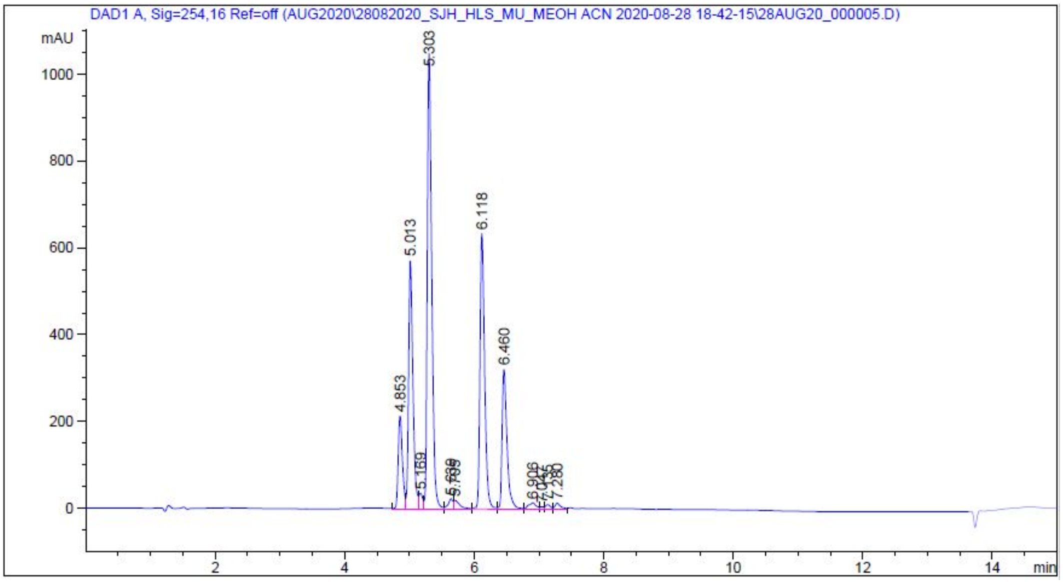

HPLC chromatogram of the resulting crude mixture after Luche reduction of Set 2 in acidic conditions.

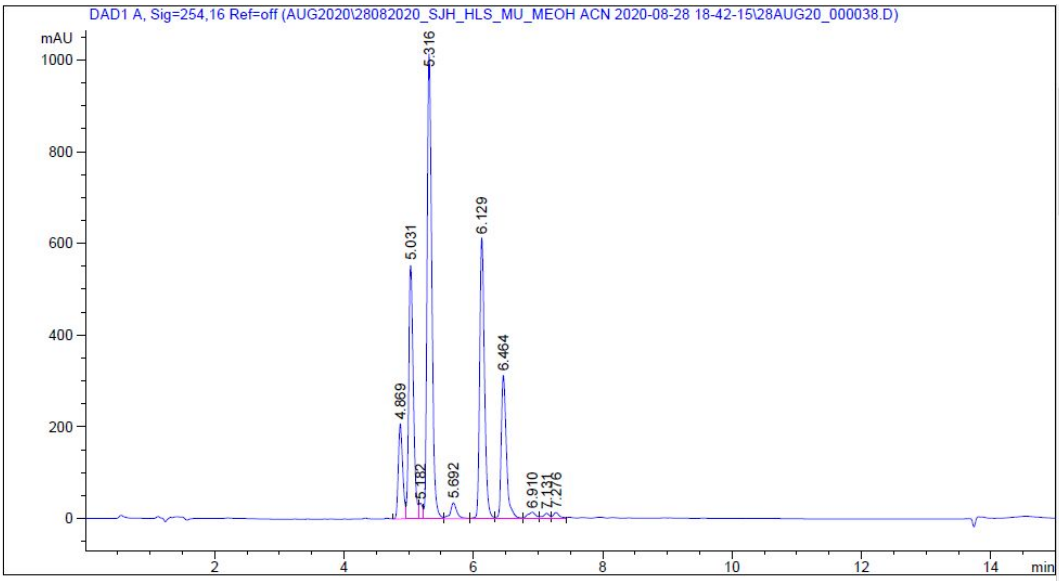

HPLC chromatogram of the resulting crude mixture after Luche reduction of Set 2 in basic conditions.

HRMS Analysis of Set 1 After $NiCl_2/NaBH_4$ Reduction

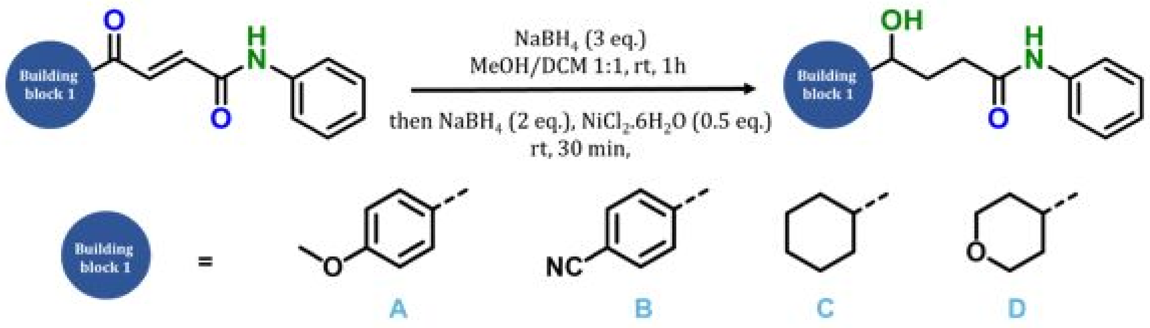

Product	Formula	Calculated mass [M + H]+	Mass found [M + H]+
A	$C_{17}H_{19}NO_3$	286.1440	286.1404
B	$C_{17}H_{16}N_2O_2$	281.1287	Not found
C	$C_{16}H_{23}NO_2$	262.1804	262.1809
D	$C_{15}H_{21}NO_3$	264.1597	264.1586

HRMS Analysis of Set 2 After $NiCl_2/NaBH_4$ Reduction

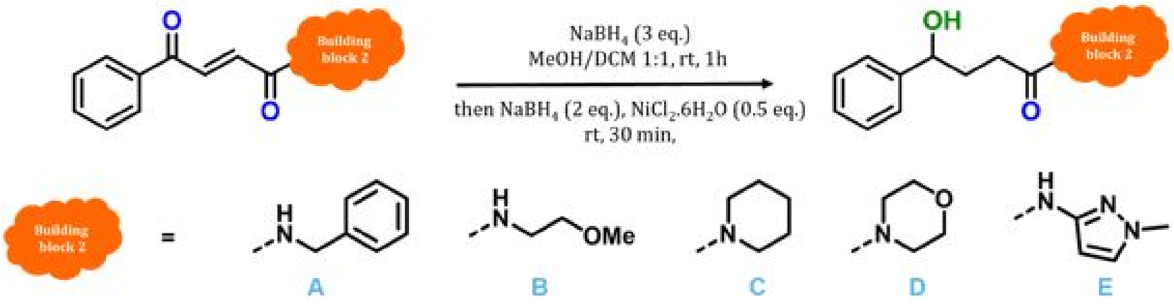

Product	Formula	Calculated mass [M + H]+	Mass found [M + H]+
A	$C_{17}H_{19}NO_2$	270.1491	270.1463
B	$C_{13}H_{19}NO_3$	238.1440	Not found
C	$C_{15}H_{21}NO_2$	248.1648	Not found
D	$C_{14}H_{19}NO_3$	250.1440	Not found
E	$C_{14}H_{17}N_3O_2$	260.1396	260.1249
A – 18	$C_{17}H_{17}NO$	251.1316	252.1408
B – 18	$C_{13}H_{17}NO_2$	219.1256	220.1330
C – 18	$C_{15}H_{19}NO$	229.1466	230.1564
D – 18	$C_{14}H_{17}NO_2$	231.1256	232.1342
E – 18	$C_{14}H_{15}N_3O$	241.1216	242.1312

HPLC Analyses of Set 1 After the $NiCl_2/NaBH_4$ Reduction Transformation

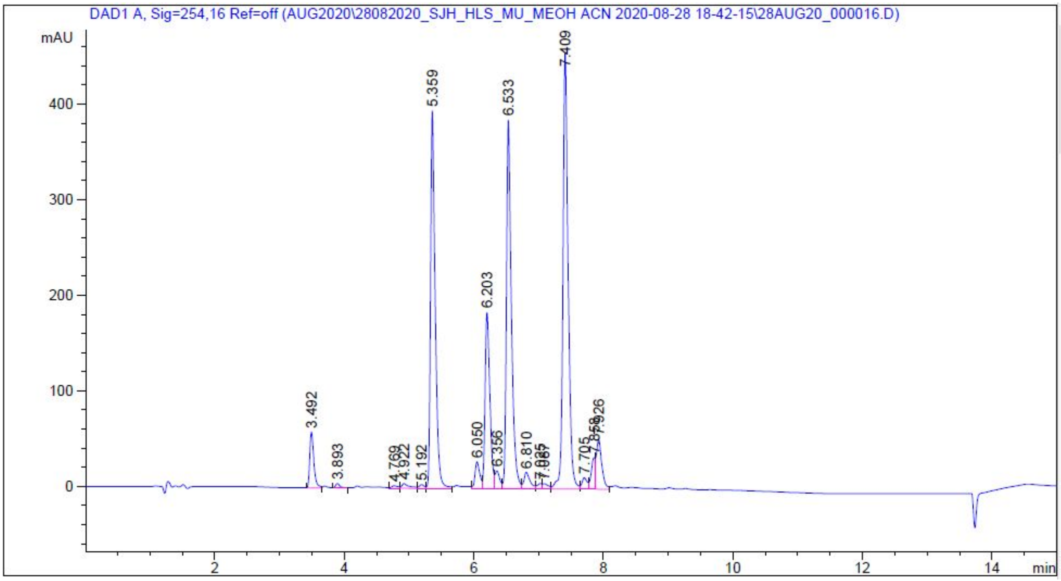

HPLC chromatogram of the resulting crude mixture after $NiCl_2/NaBH_4$ reduction of Set 1 in acidic conditions.

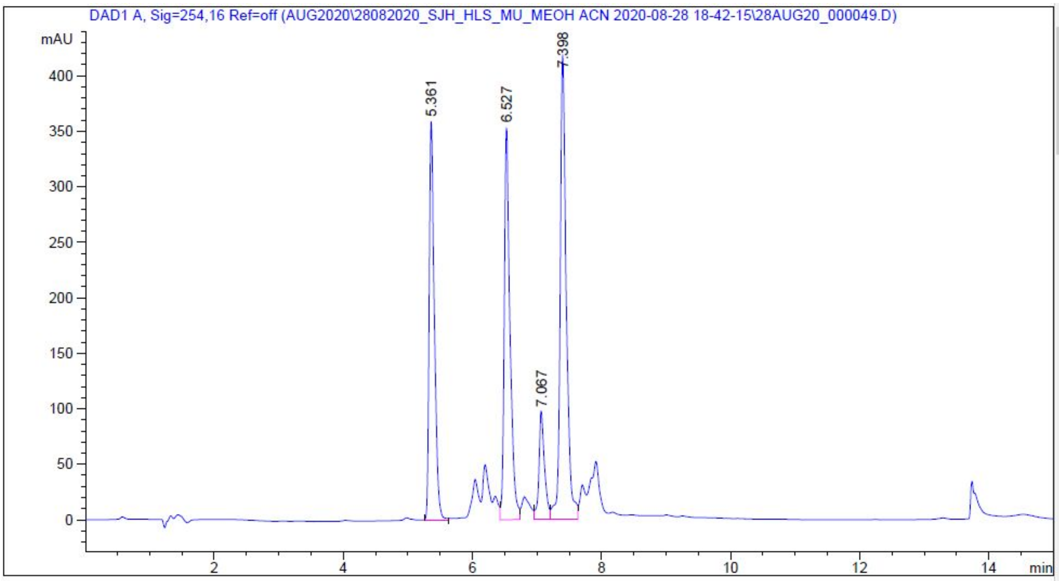

HPLC chromatogram of the resulting crude mixture after $NiCl_2/NaBH_4$ reduction of Set 1 in basic conditions.

HPLC Analyses of Set 2 After the $NiCl_2/NaBH_4$ Reduction Transformation

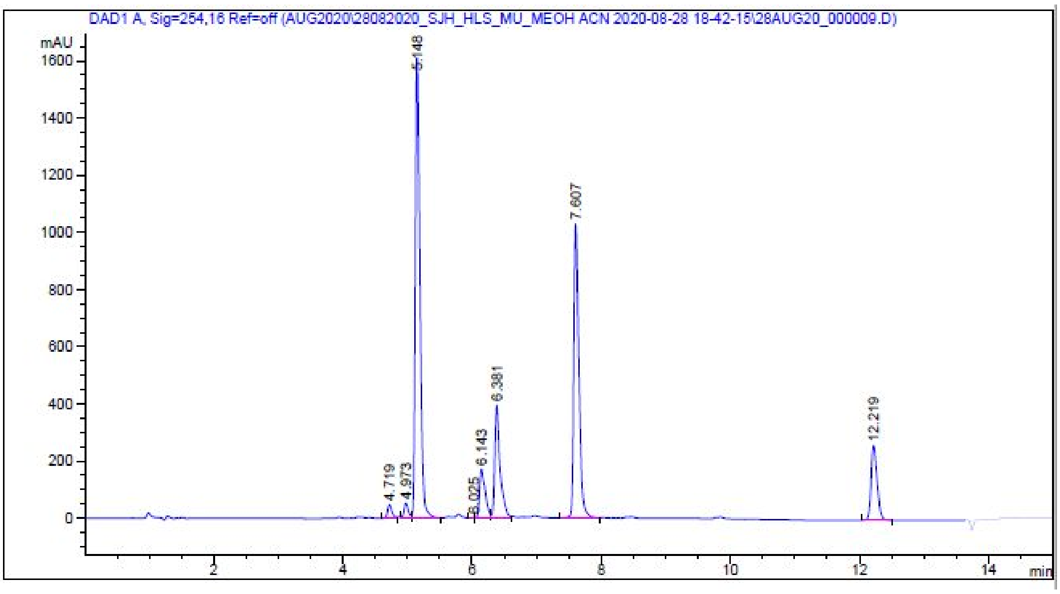

HPLC chromatogram of the resulting crude mixture after $NiCl_2/NaBH_4$ reduction of Set 2 in acidic conditions.

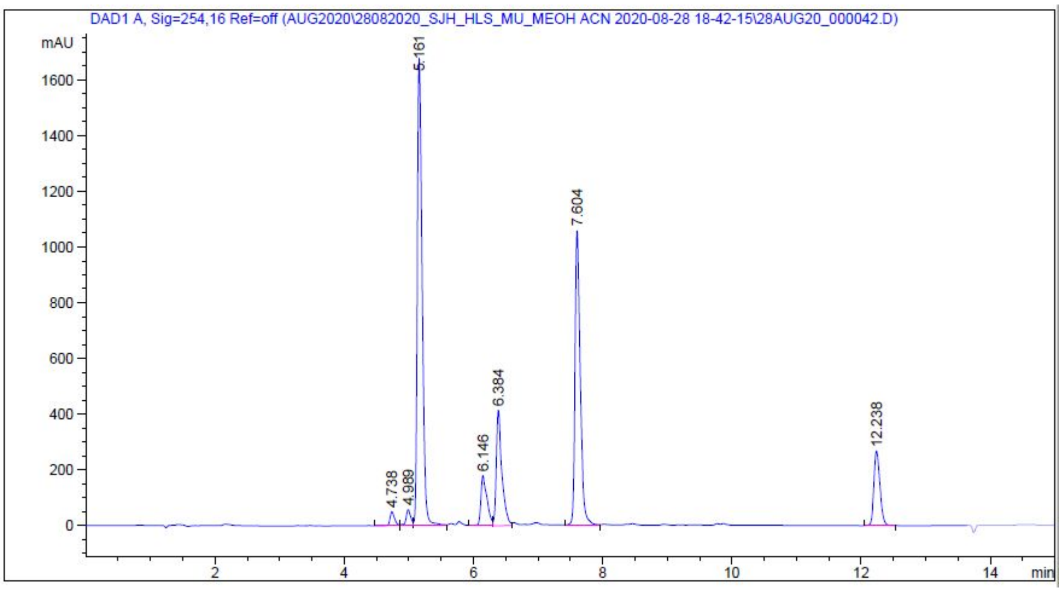

HPLC chromatogram of the resulting crude mixture after $NiCl_2/NaBH_4$ reduction of Set 2 in basic conditions.

3.2.4. Transformations: 1,4-Additions

HRMS Analysis of Set 1 After 1,4-Addition of Pyrrolidine

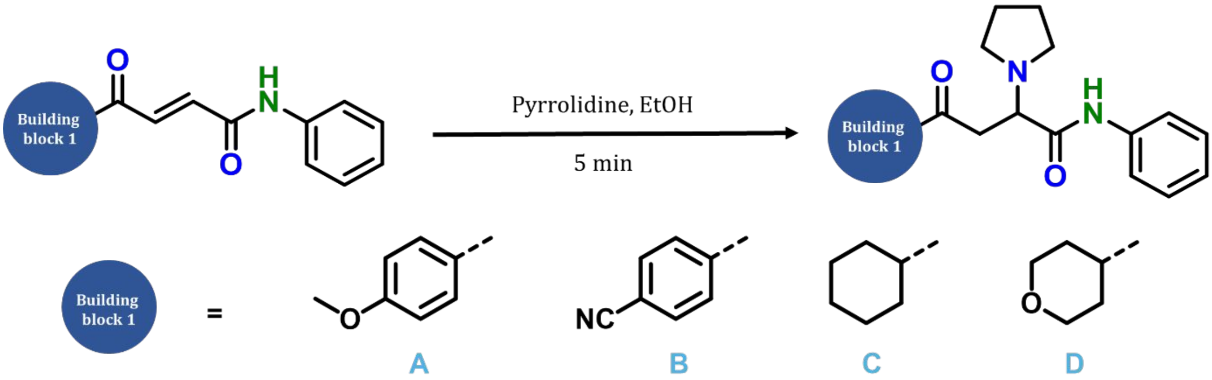

Product	Formula	Calculated mass [M + H]+	Mass found [M + H]+
A	$C_{21}H_{24}N_2O_3$	353.1862	Not found
B	$C_{21}H_{21}N_3O_2$	348.1709	Not found
C	$C_{20}H_{28}N_2O_2$	329.2226	Not found
D	$C_{19}H_{26}N_2O_3$	331.2019	Not found

HRMS Analysis of Set 2 After 1,4-Addition of Pyrrolidine

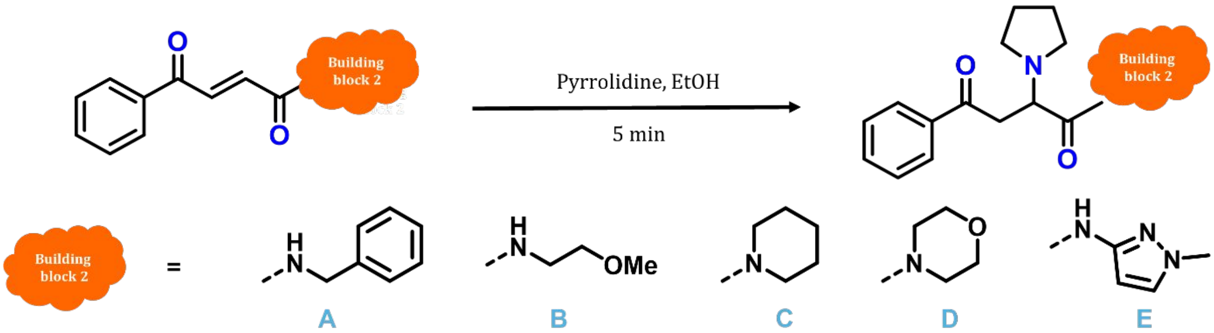

Product	Formula	Calculated mass [M + H]+	Mass found [M + H]+
A	$C_{21}H_{24}N_2O_2$	337.1913	Not found
B	$C_{17}H_{24}N_2O_3$	305.1862	Not found
C	$C_{19}H_{26}N_2O_2$	315.2070	Not found
D	$C_{18}H_{24}N_2O_3$	317.1862	Not found
E	$C_{18}H_{22}N_4O_2$	327.1818	Not found

HPLC Analyses of Set 1 After 1,4-Addition of Pyrrolidine

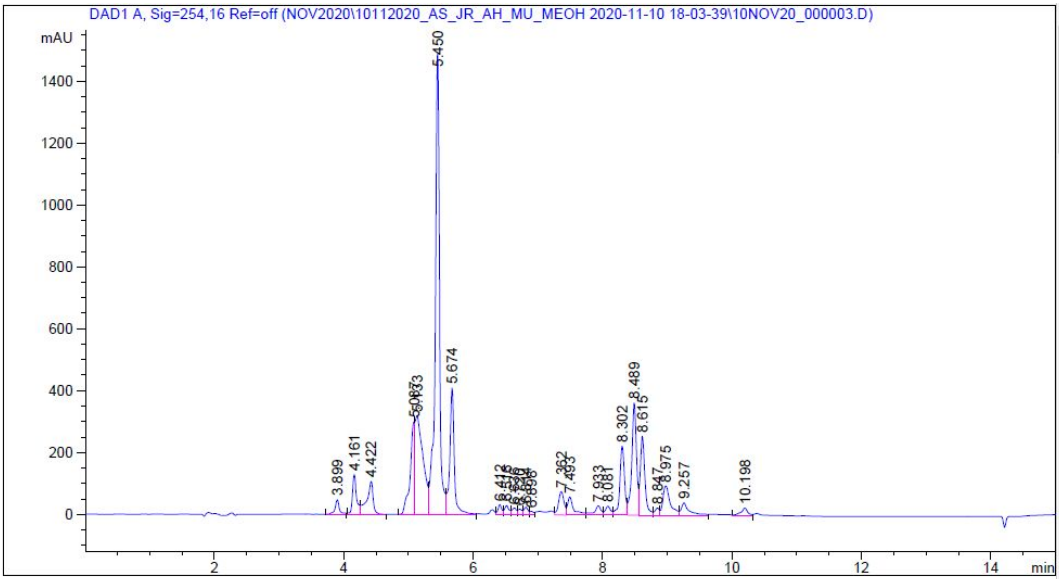

HPLC chromatogram of the resulting crude mixture after 1,4-addition of pyrrolidine applied to Set 1 in acidic conditions.

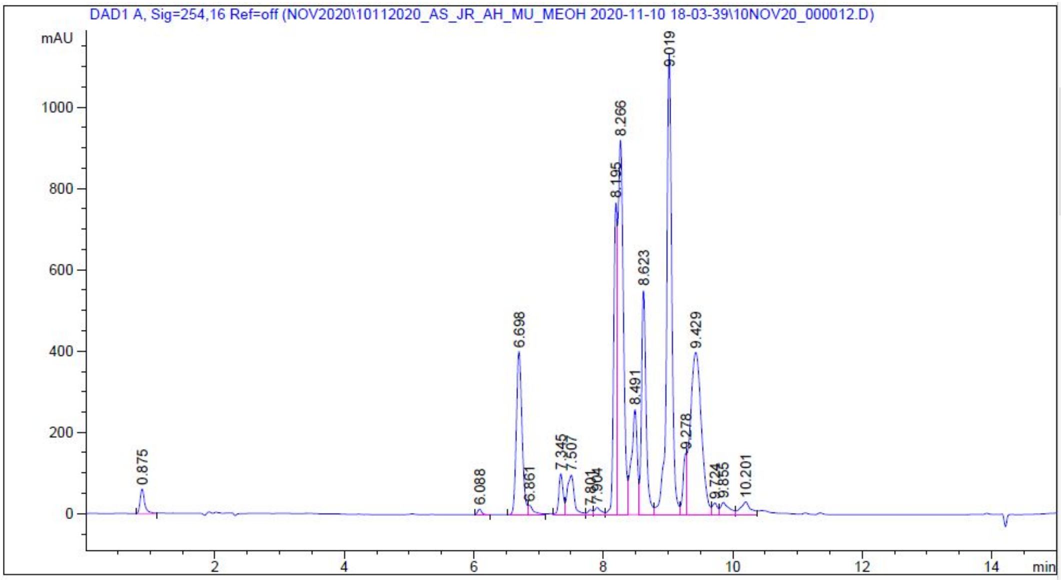

HPLC chromatogram of the resulting crude mixture after 1,4-addition of pyrrolidine applied to Set 1 in basic conditions.

HPLC Analyses of Set 2 After 1,4-Addition of Pyrrolidine

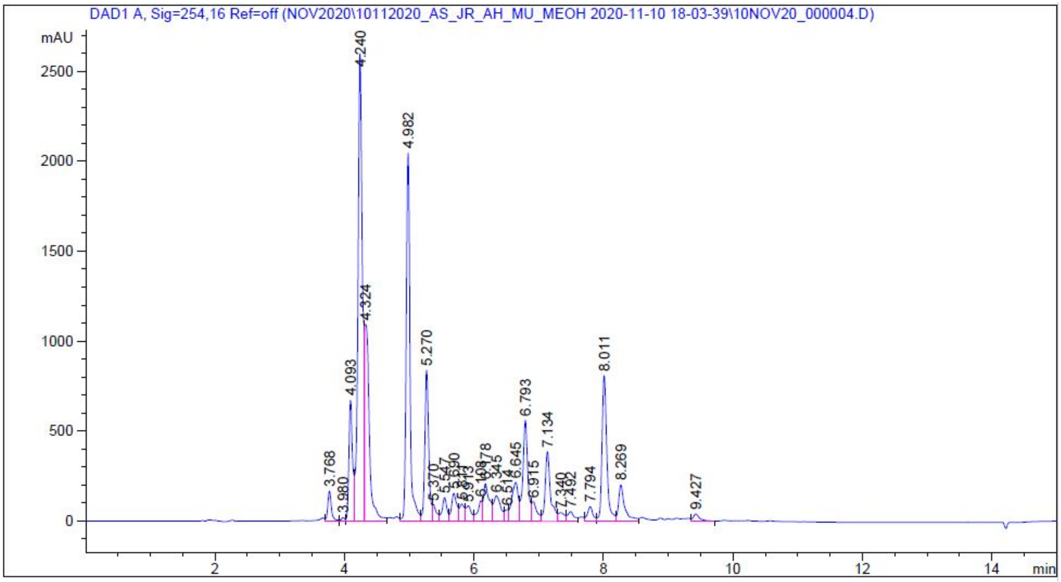

HPLC chromatogram of the resulting crude mixture after 1,4-addition of pyrrolidine applied to Set 2 in acidic conditions.

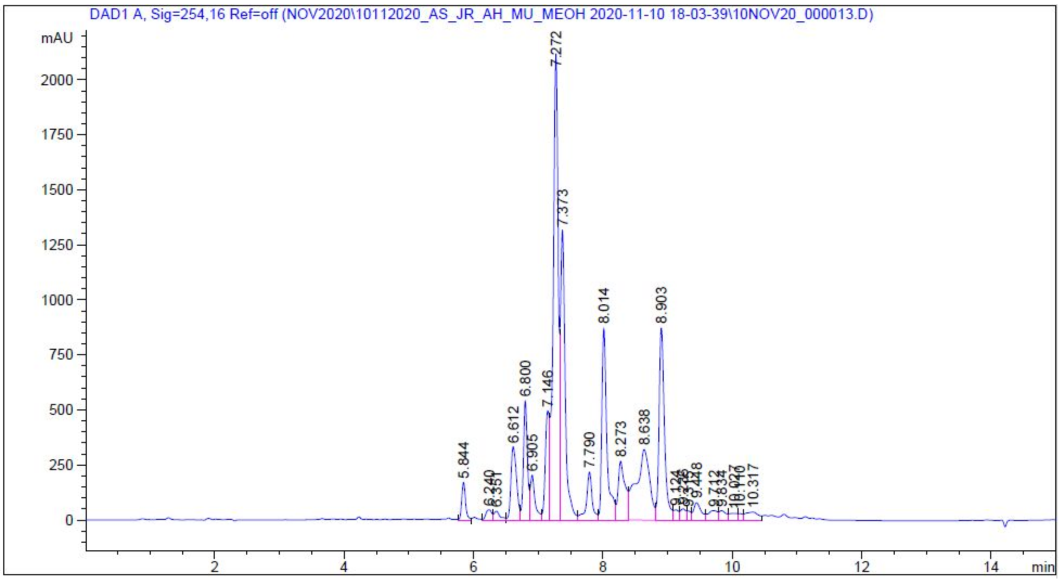

HPLC chromatogram of the resulting crude mixture after 1,4-addition of pyrrolidine applied to Set 2 in basic conditions.

4. Computational Analysis

4.1 General details

Property analyses and PCA analysis was generated with DataWarrior software.[2]

Moment of Inertia (PIM) was generated using LLAMA software.[3]

4.2 Compound collections analysed

Collection 1: Intermediates

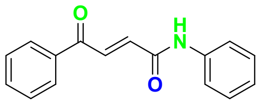

1a

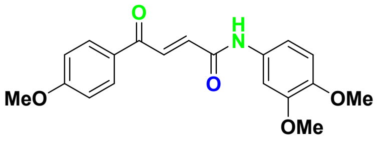

1b

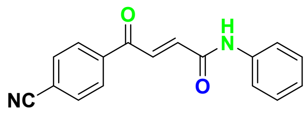

1c

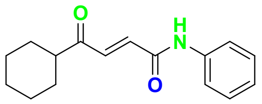

1d

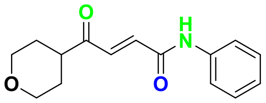

1e

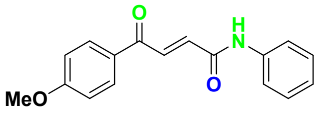

1f

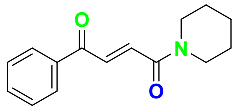

1h

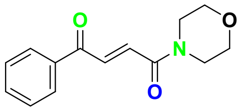

1i

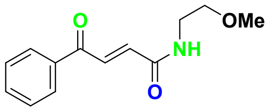

1j

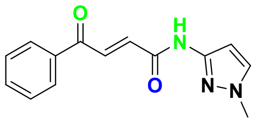

1k

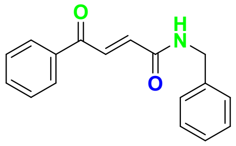

1l

Collection 2: DOS library

3.2.1. Transformations: 1,4-Cyclisations:

After Pyrazole Formation (7)

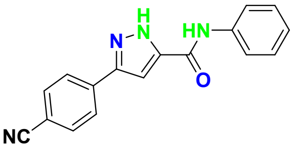

7c

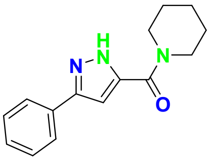

7h

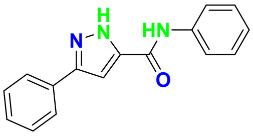

7a

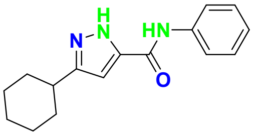

7d

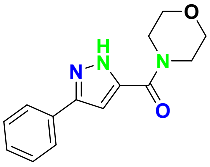

7i

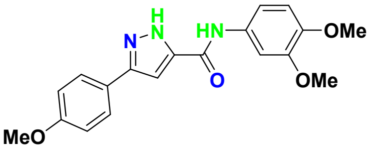

7b

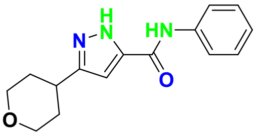

7e

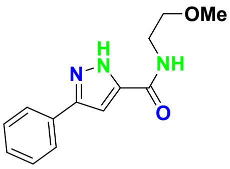

7j

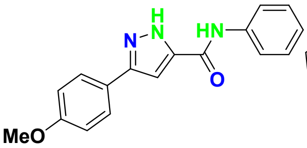

7f

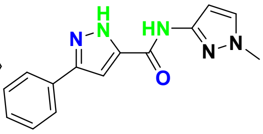

7k

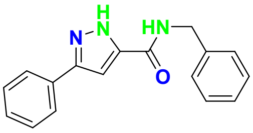

7l

After Isoxazole Formation (8)

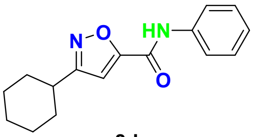

8d

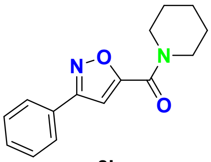

8l

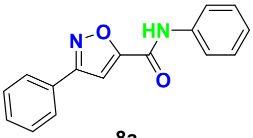

8a

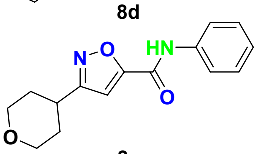

8e

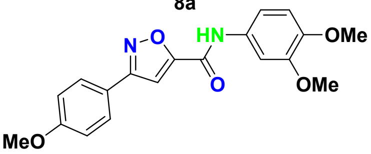

8b

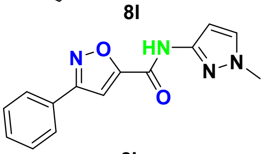

8k

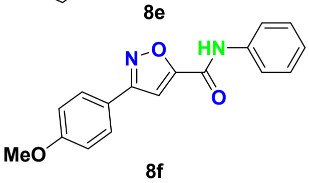

8f

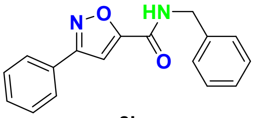

8l

After Pyrazolo[1.5-*a*]pyrimidine Formation (9)

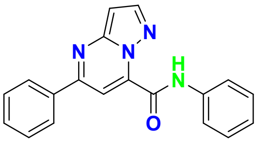

9a

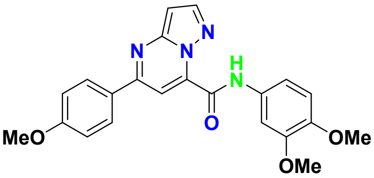

9b

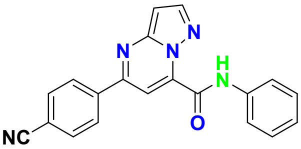

9c

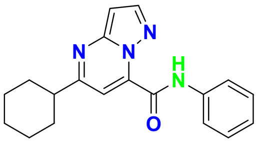

9d

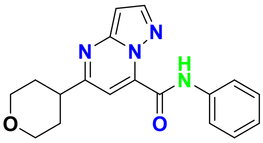

9e

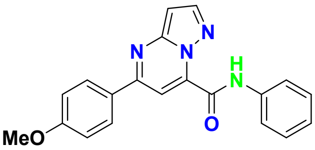

9f

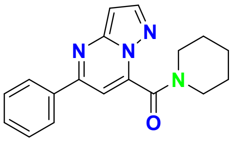

9h

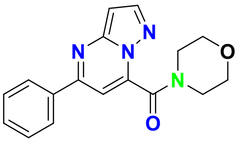

9i

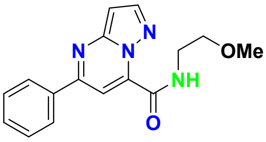

9j

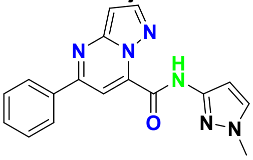

9k

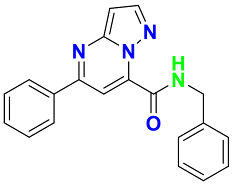

9l

After Imidazo[1.2-*a*]pyridine Formation (10)

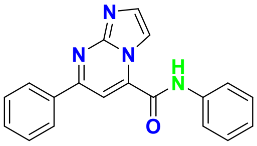

10a

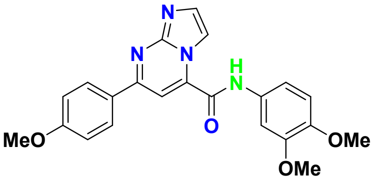

10b

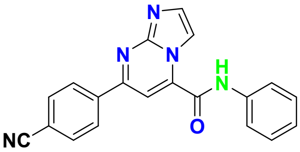

10c

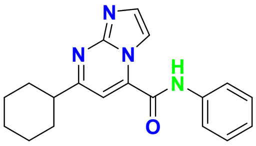

10d

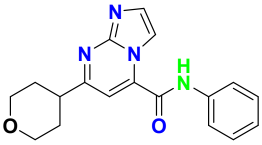

10e

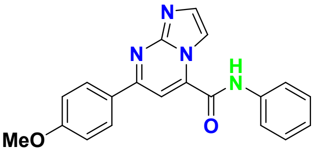

10f

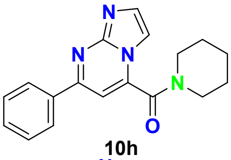

10h

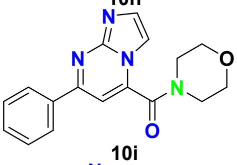

10i

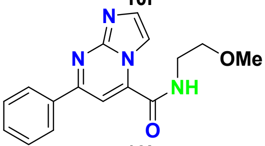

10j

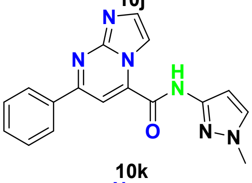

10k

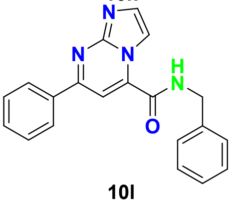

10l

After 2-Methoxypyrimidine Formation (11)

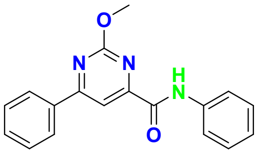

11a

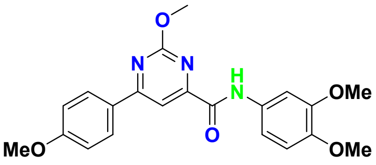

11b

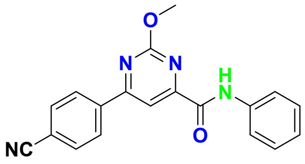

11c

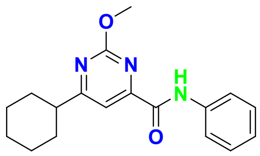

11d

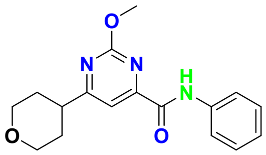

11e

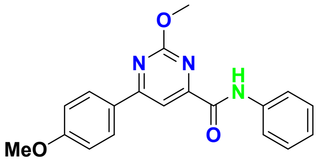

11f

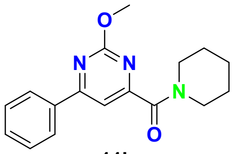

11h

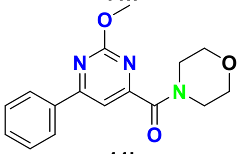

11i

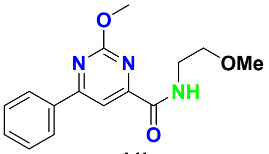

11j

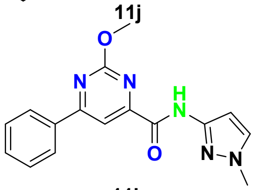

11k

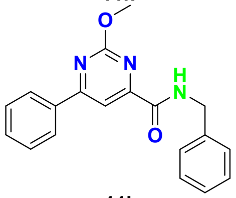

11l

After Pyrimidone Formation (12)

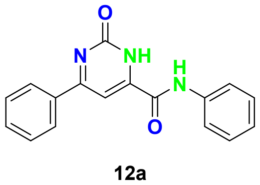

12a

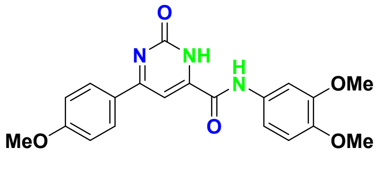

12b

After 2-PMB-thiopyrimidine Formation (13)

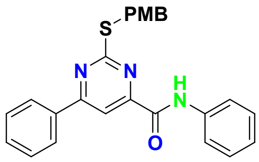

13a

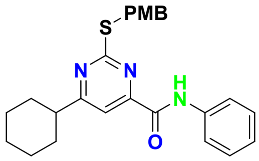

13d

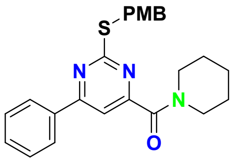

13h

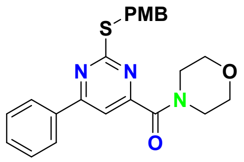

13i

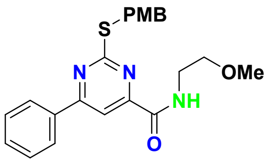

13j

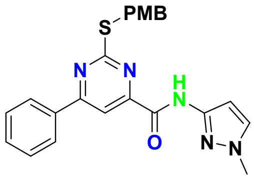

13k

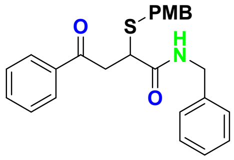

13l

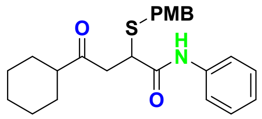

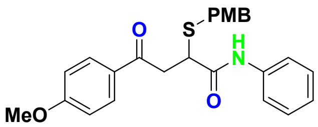

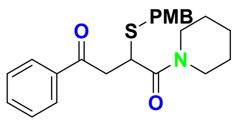

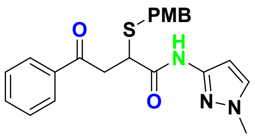

After 2-Aminopyrimidine Formation (14)

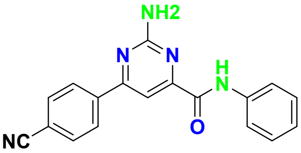

14c

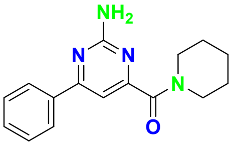

14h

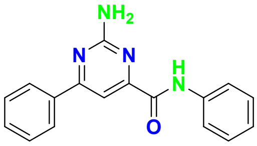

14a

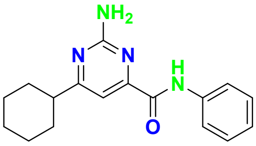

14d

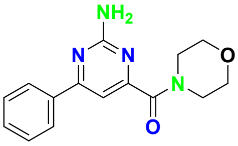

14i

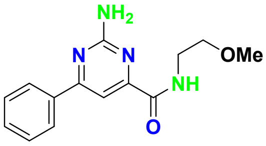

14j

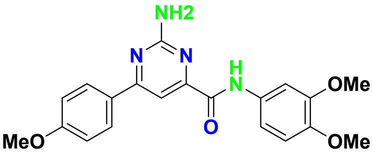

14b

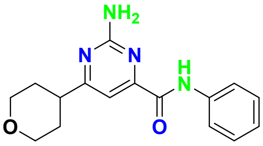

14e

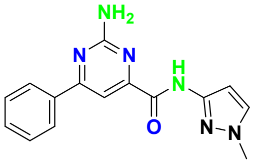

14k

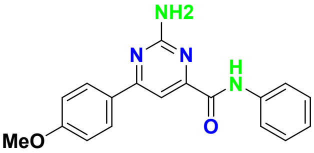

14f

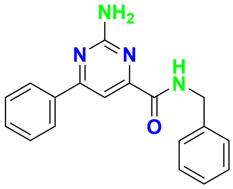

14l

After Kröhnke Pyridine Synthesis (15)

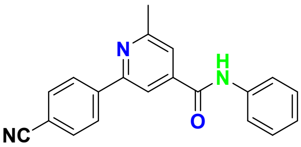

15c

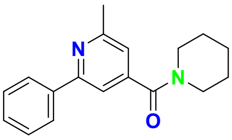

15h

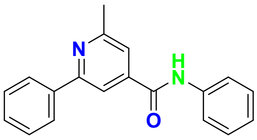

15a

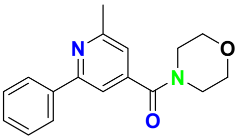

15i

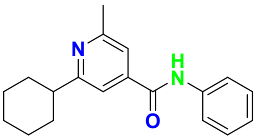

15d

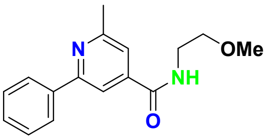

15j

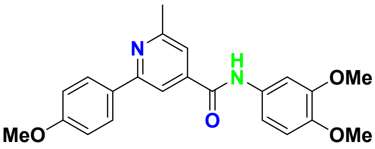

15b

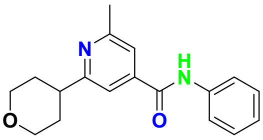

15e

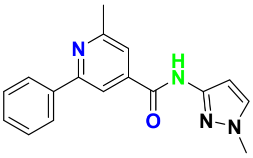

15k

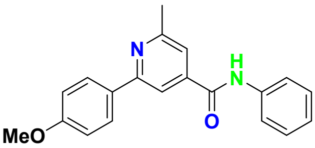

15f

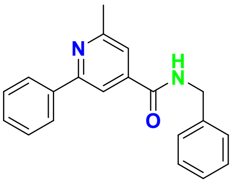

15l

3.2.2. Transformations: 3,4-Cycloadditions

After Corey-Chaykovski Cyclopropanation (16)

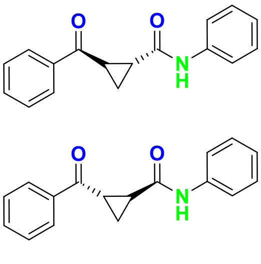

16a

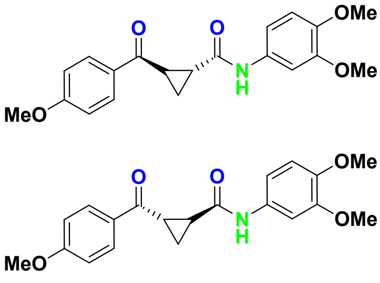

16b

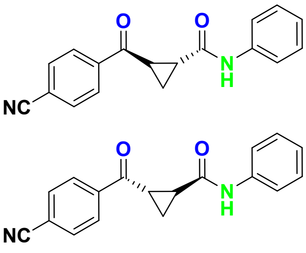

16c

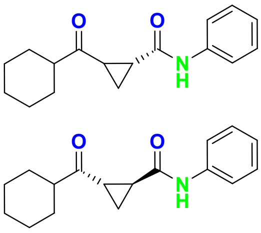

16d

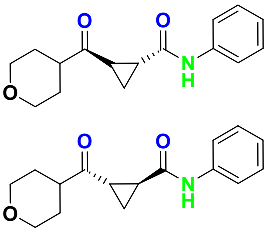

16e

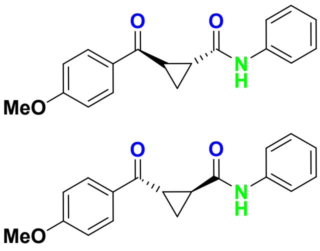

16f

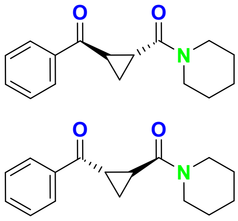

16h

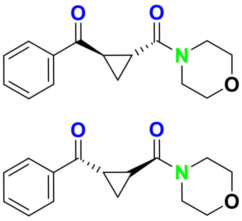

16i

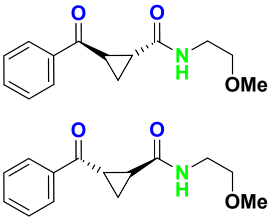

16j

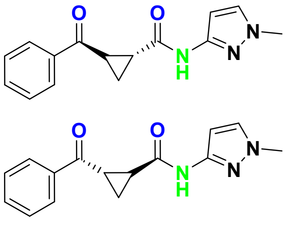

16k

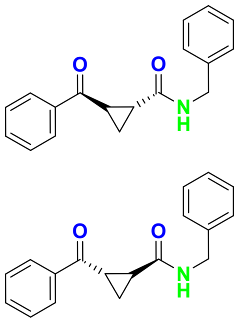

16l

After Diels/Alder with Cyclopentadiene (17)

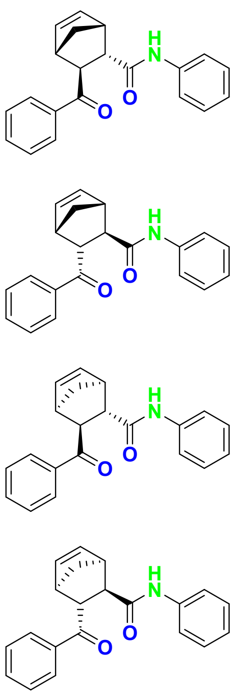

17a

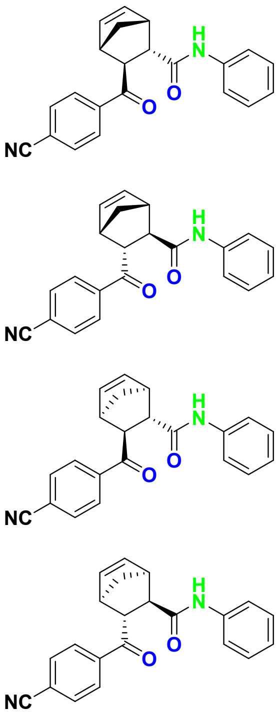

17c

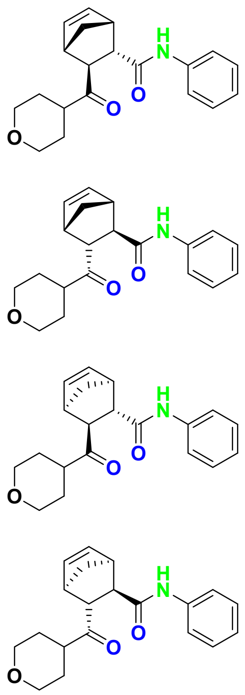

17e

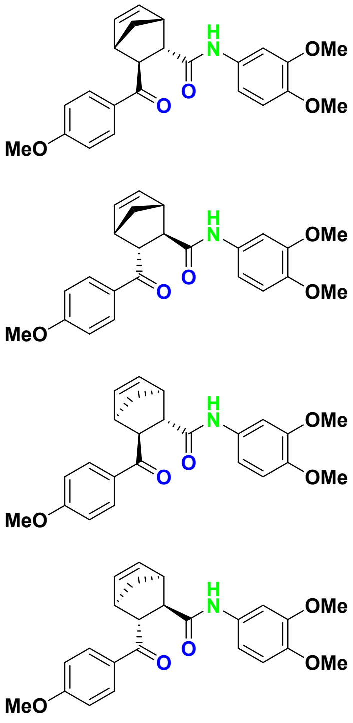

17b

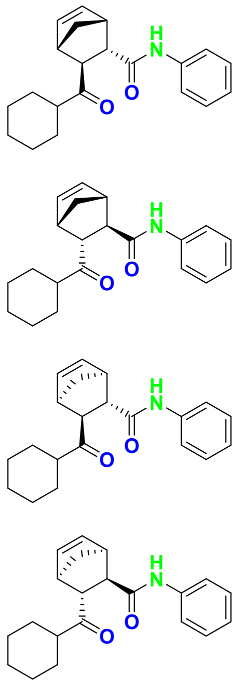

17d

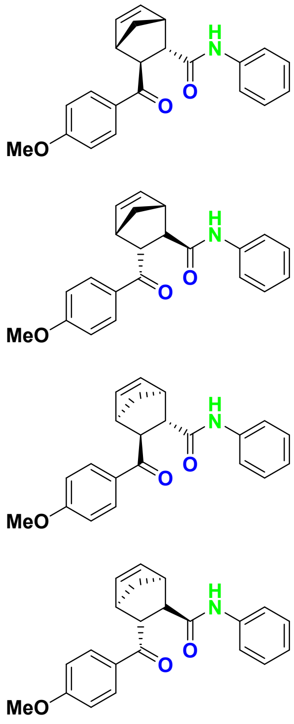

17f

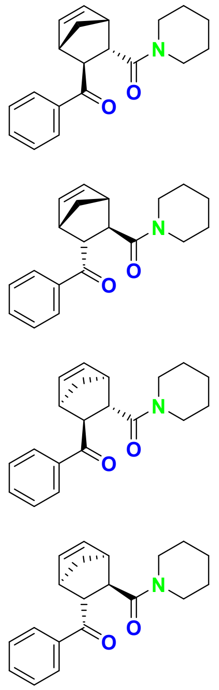

17h

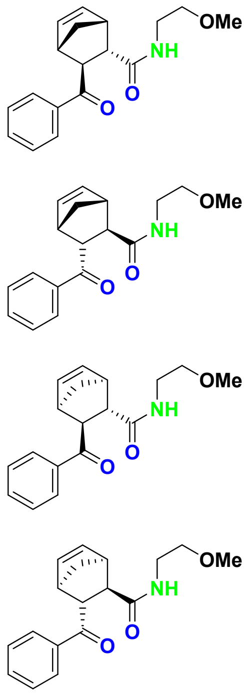

17j

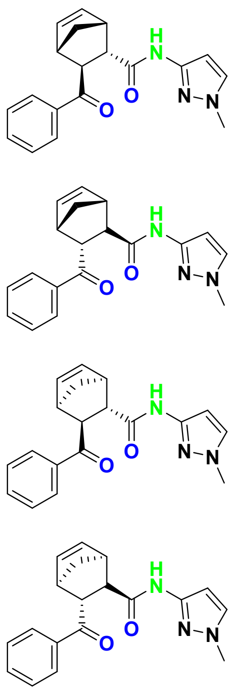

17k

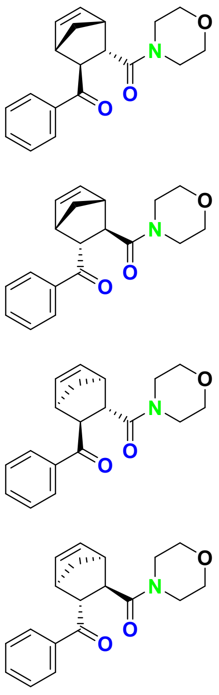

17i

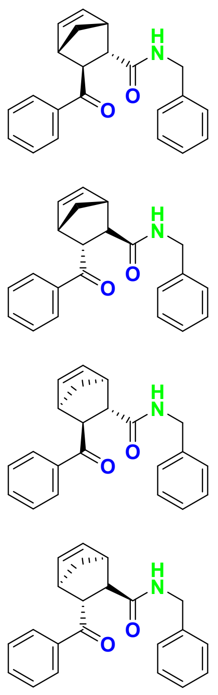

17l

After Diels/Alder with 2,3-Dimethyl-1,3-butadiene (18 & 19)

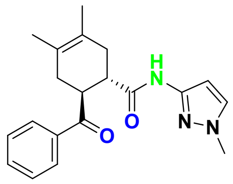

18k

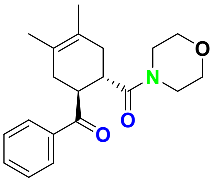

18i

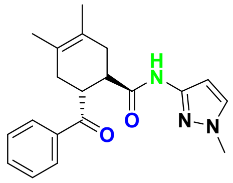

18k

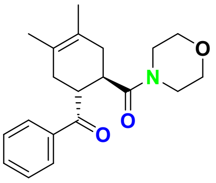

18i

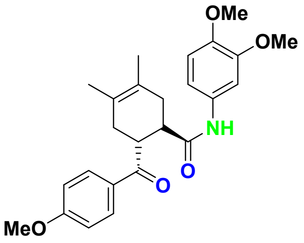

18b

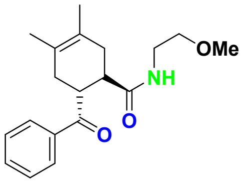

18j

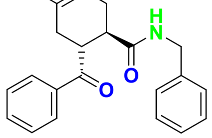

18l

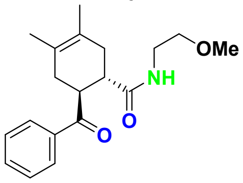

18j

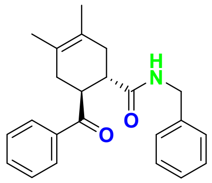

18l

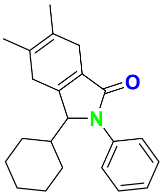

19d

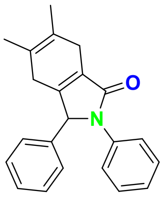

19a

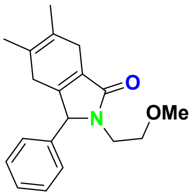

19j

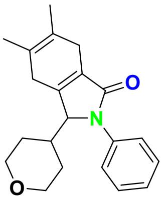

19e

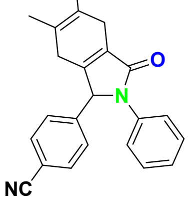

19c

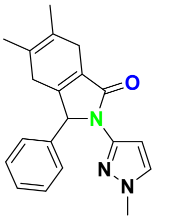

19k

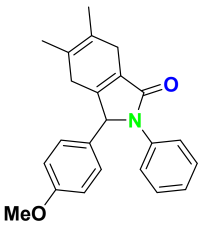

19f

After Van Leusen Pyrrole Synthesis (20)

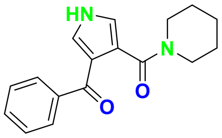

20h

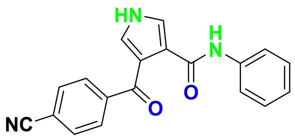

20c

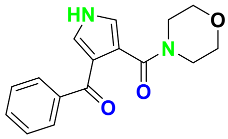

20i

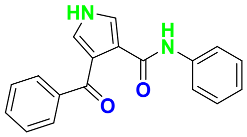

20a

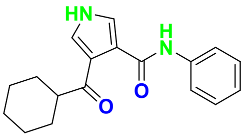

20d

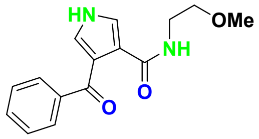

20j

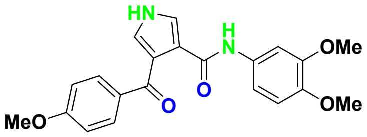

20b

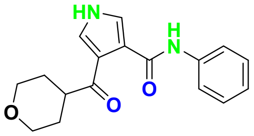

20e

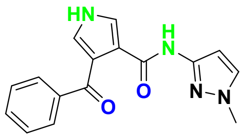

20k

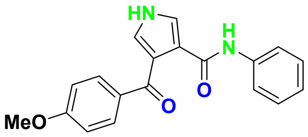

20f

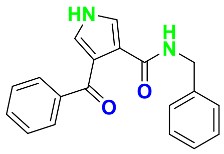

20l

After Benzyltriazole Formation (21)

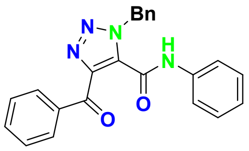

21a

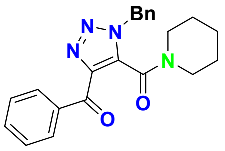

21h

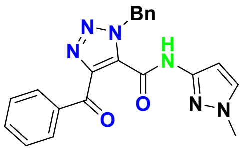

21k

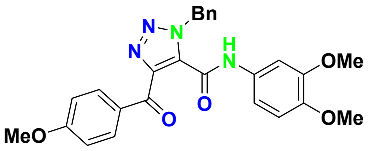

21b

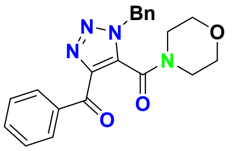

21i

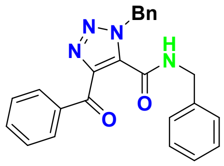

21l

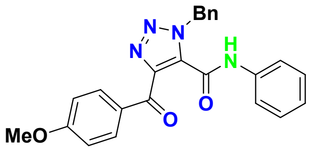

21d

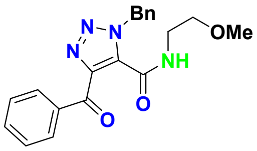

21j

After Triazole Formation (22)

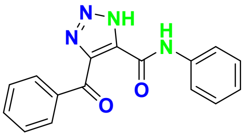

22a

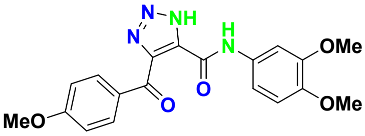

22b

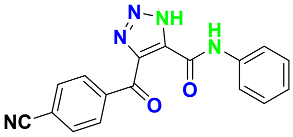

22c

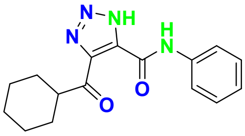

22d

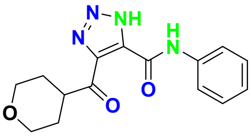

22e

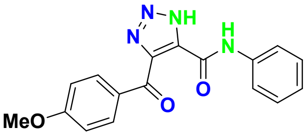

22f

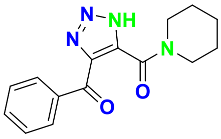

22h

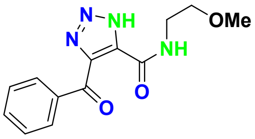

22j

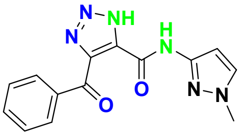

22k

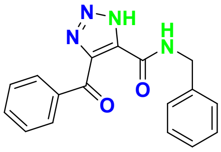

22l

After Isoxazolidine Formation (23)

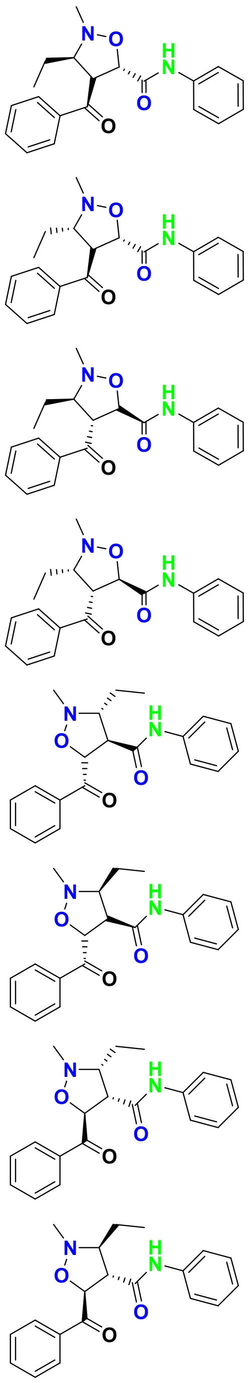

23a

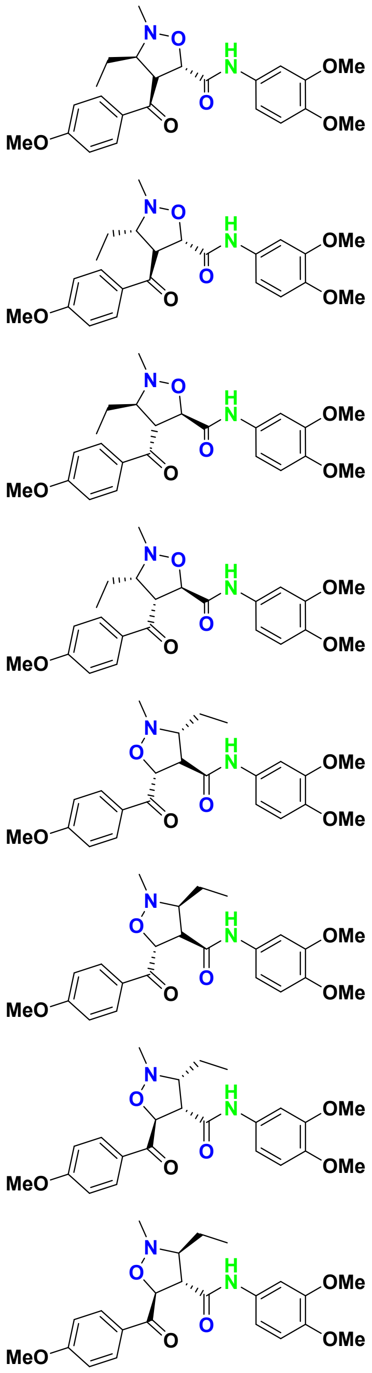

23b

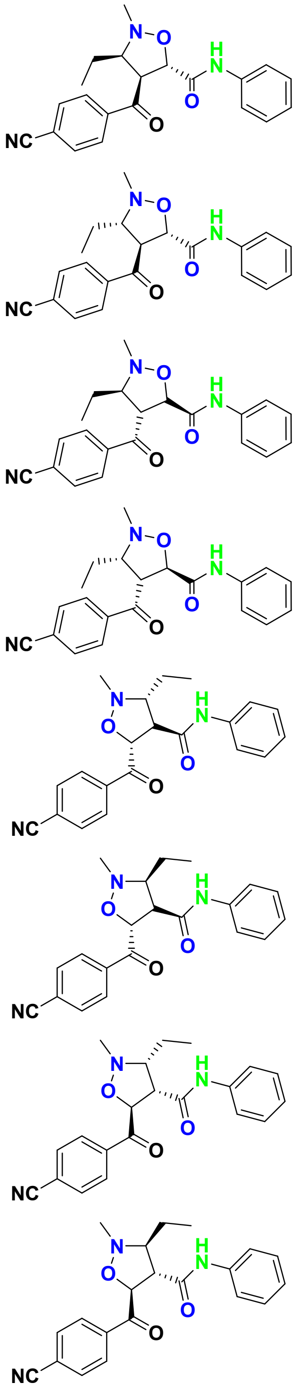

23c

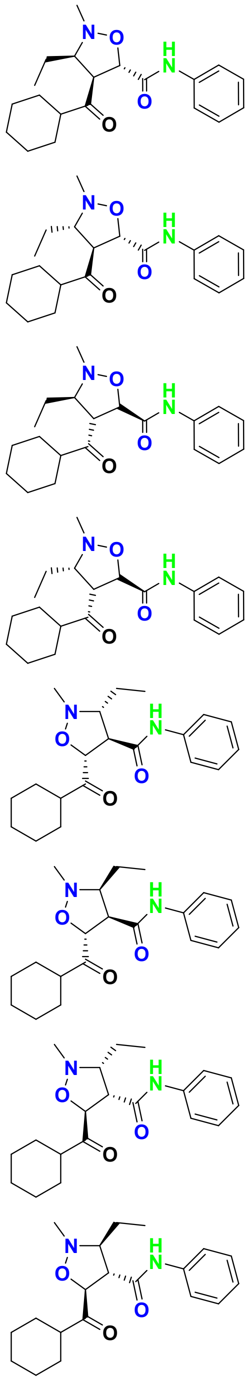

23d

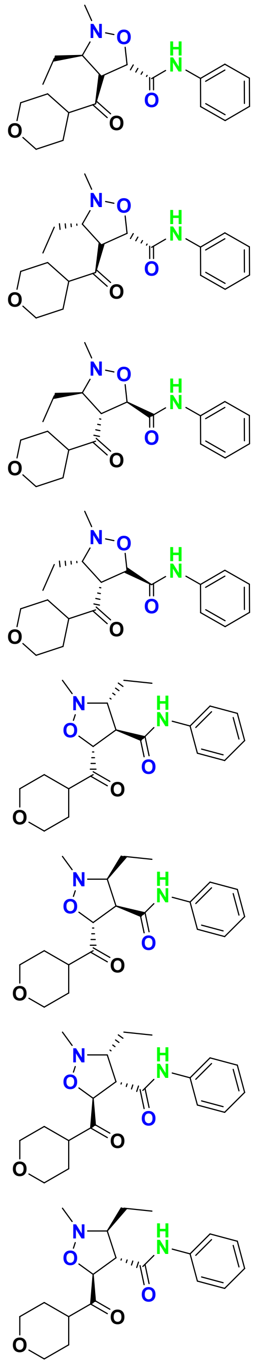

23e

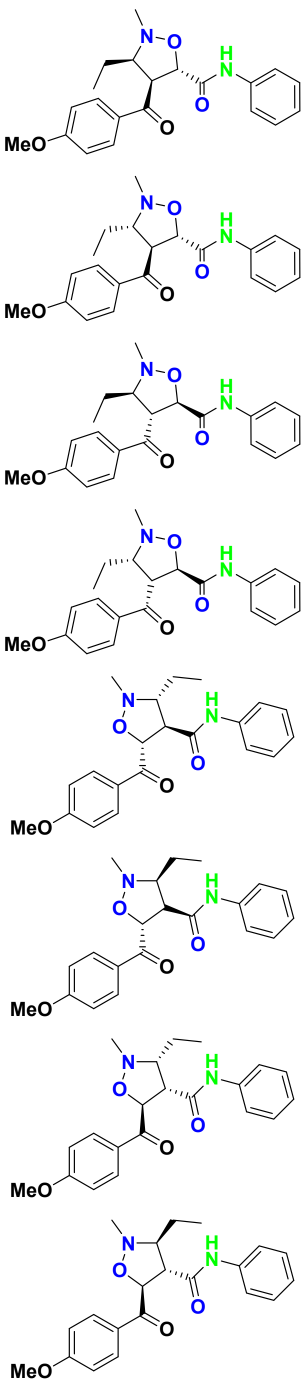

23f

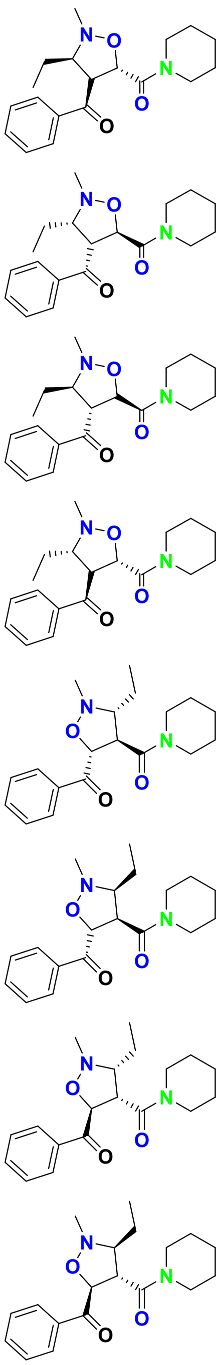

23h

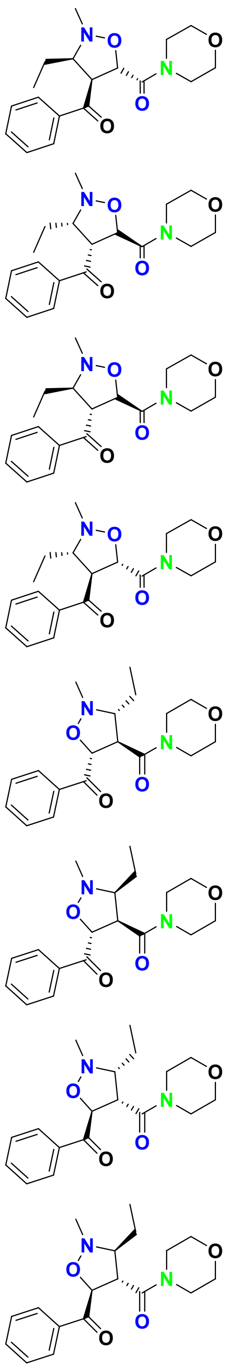

23i

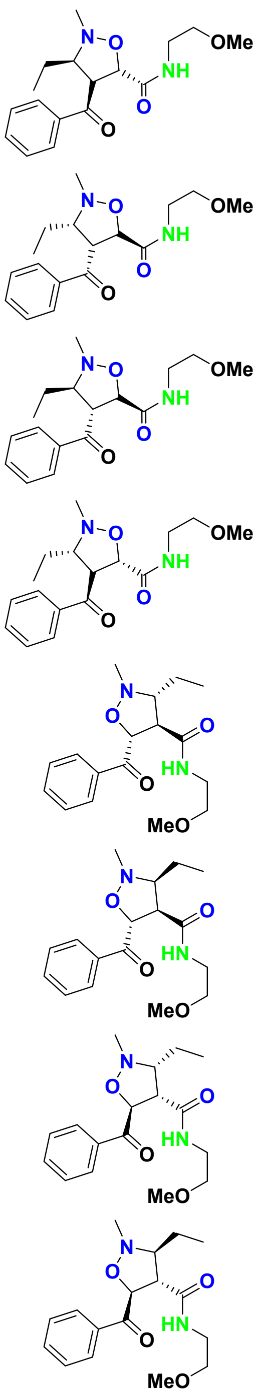

23j

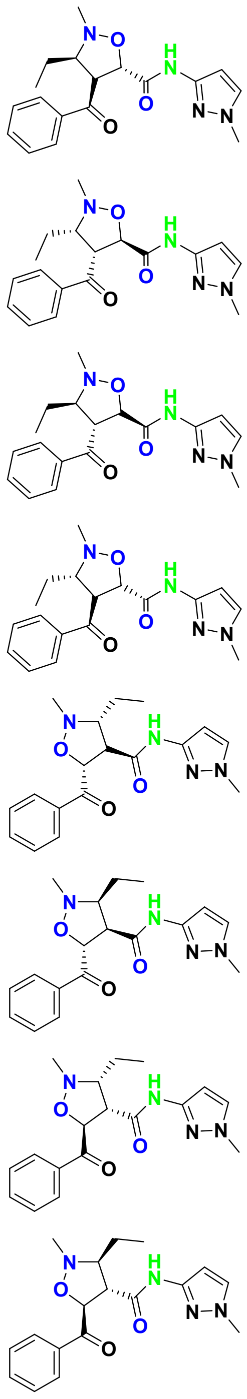

23k

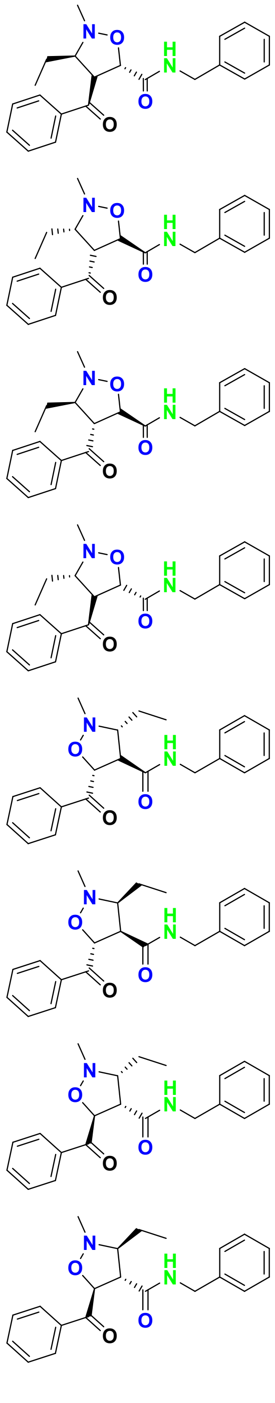

23l

3.2.3. Transformations: Reductions

After Hydrogenation (24)

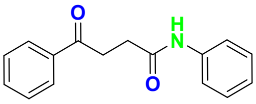

24a

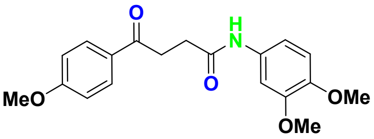

24b

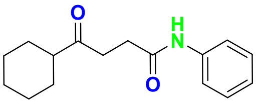

24d

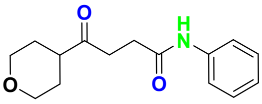

24e

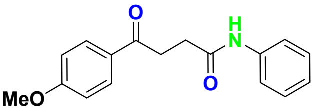

24f

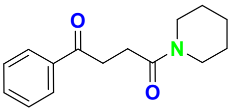

24h

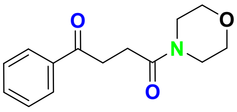

24i

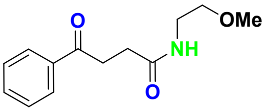

24j

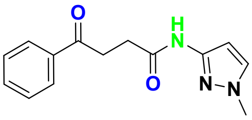

24k

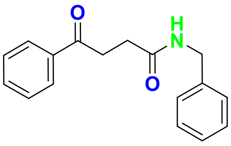

24l

After Luche Reduction (25)

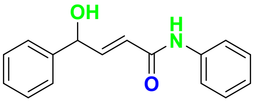

25a

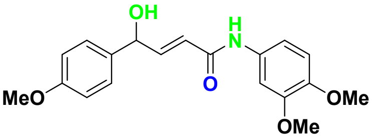

25b

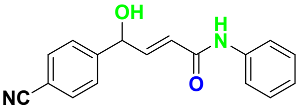

25c

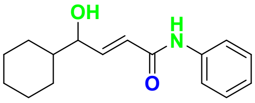

25d

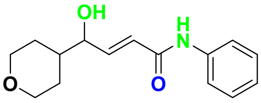

25e

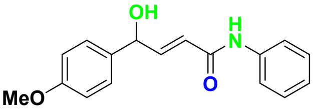

25f

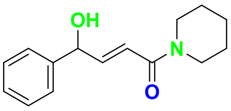

25h

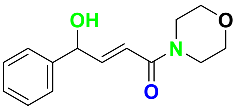

25i

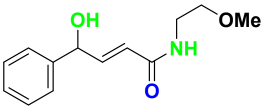

25j

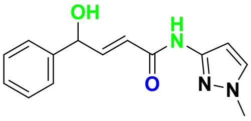

25k

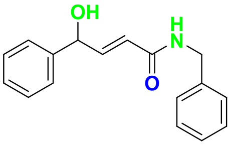

25l

After $NiCl_2/NaBH_4$ Reduction (26)

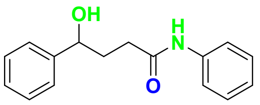

26a

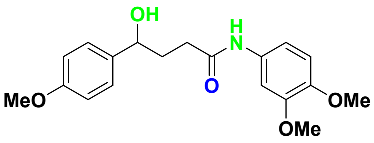

26b

3.2.4. Transformations: 1,4-Additions

After 1,4-Addition of Pyrrolidine (27)

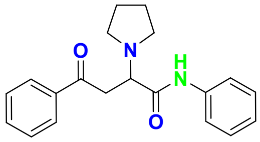

27a

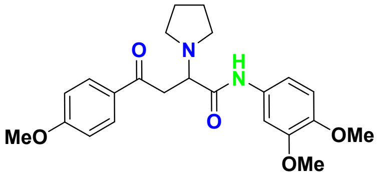

27b

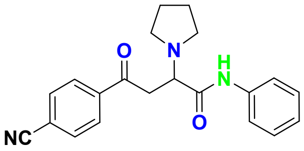

27c

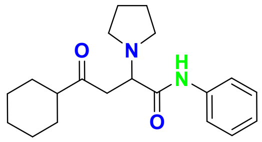

27d

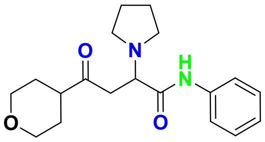

27e

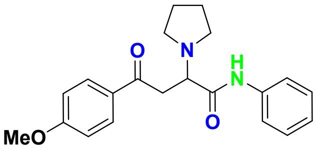

27f

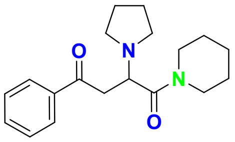

27h

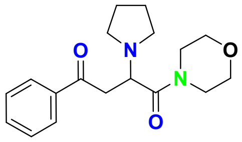

27i

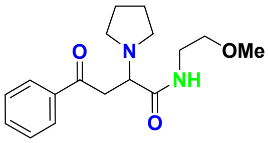

27j

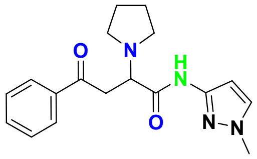

27k

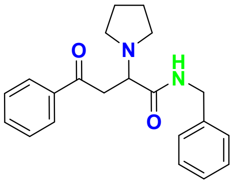

27l

5. Materials and Methods for Biological Evaluation

5.1 Expression and purification of CDK2.

Human CDK2 was expressed and purified as previously described (Martin et al, 2012). Full length CDK2 (residues 1-298) and Avi-tagged CDK2 were cloned into pGEX6p-1 vector (GE Healthcare), transformed into *E. coli* BL21(DE3) Star, grown at 37 °C for 2-3 hrs to OD 0.4-0.6 before induction at 18 °C with 0.2 mM IPTG (Sigma) for a further 16-20 hours. Cells were then harvested by centrifugation at 4,000 rpm 4 °C for 20 mins before resuspension in 50 mM HEPES, 150 mM NaCl, 2 mM DTT, pH 7.4. 2 mg mL-1 DNase I, 10 mg mL-1 RNase A, 25 mg mL-1 lysozyme and 5 mM $MgCl_2$ were added to cell suspensions before lysis via sonication (5 mins total, pulsed 20s on and 40s off, 30 % amp). The lysate was clarified by centrifugation at 20,000 rpm for 1hr at 4 °C and syringe filtered (0.2 μm). GST-3C-CDK2 was purified by batch binding to glutathione Sepharose 4B resin (pre-equilibrated in 50 mM HEPES, 150 mM NaCl, 2 mM DTT, pH 7.4) for 1hr at 4 °C then eluted in 50 mM HEPES, 150 mM NaCl, 2 mM DTT, 20 mM reduced glutathione, pH 7.4. Eluted GST-3C-CDK2 was cleaved overnight (~16 hours) at 4 °C using 1:100 (w/w) of 3C protease:GST-CDK2. Cleaved product was further purified by gel filtration (S75 pg Superdex® HiLoad® (GE Healthcare)). CDK2 peak fractions were pooled and remaining free GST removed by glutathione affinity subtraction. Purified CDK2 was then buffer exchanged, using a HiTrap desalting column (GE Healthcare), into 100 mM 4:1 $K_2HPO_4.3H_2O:NaH_2PO_4.H_2O$, 2 mM DTT buffer, concentrated to 9.7-10 mg mL-1 and stored at -80 °C. Purified Avi-CDK2 was biotinylated as described (PMID: 29091774) and stored at 1mg mL-1 at -80 °C.

5.2 Surface Plasmon resonance Screening of DOS library against CDK2.

DOS library screening were performed on a Biacore S200 (Cyvita) at 20˚C using multi-cycle settings with a running buffer of 20 mM HEPES, 150 mM NaCl, 0.01 % tween-20, pH 7.4, 1% DMSO. Avi-tagged-CDK2 (50μg/mL) was immobilised onto the surface of a SA chip (Cyvita). The 31 DOS library screening compound cocktails were then injected over the reference and active surfaces at three concentrations 2.5μM, 20 μM, and 200μM. A buffer run was performed after each injection, alongside positive (SU9516 200nM) and negative controls (DMSO) run at beginning and end of each concentration run. A solvent correction control was also performed to account for any bulk flow interactions. All responses were subtracted from the reference flow cell, and analysed using Biacore S200 Evaluation Software 1.0 (Cyvita). Results Plot were fitted using blank subtraction and adjustment for controls, and affinity fit used to determine the Kd and standard error (SE) of the fit.

5.3 Crystalisation and ligand soaking of CDK2.

Monomeric CDK2 was crystallised as described in Martin et al 2021, using hanging drop vapour diffusion. Drops at a 1:1 ratio of 10 mg mL-1 CDK2:precipitant solution (100 mM HEPES pH 7.4, 8 % PEG 3350) were set up above a reservoir of 50 mM HEPES, 50 mM 1:1 $K_2HPO_4.H_2O:NaH_2PO_4.H_2O$ and incubated for a minimum of 2 days at 20 °C. Crystals were transferred into precipitant solution (50 mM HEPES pH 7.4, 8 % PEG 3350) containing a final concentration of 2-2.5 mM DOS ligand at 10 % DMSO and incubated for 3 days before harvesting into 30 % EG cryo-protectant in precipitant solution. X-ray diffraction data was collected at Diamond Light Source, Oxford UK. The CCP4i2 software package (Potterman et al, 2018; Winn et al, 2011) was used for structure determination and analysis. Data was processed using DIALS (Waterman et al, 2013) and XDS (Kabsch, 2010) in Xia2, molecular replacement using Dimple pipeline (Wojdry et al, 2013) and REFMAC (Murshudov et al 2011) for further refinement. Models were built using COOT (Emsley et al, 2010).

5.4 Crystalisation table.

**Complex**	**CDK2 + DOS9K**
**PDB code**	**7ZPC**
Data Collection	
Space Group	P 21 21 21
Unit Cell dimensions (a,b,c)(Å)	53.2,72.0,72.0
Unit Cell dimensions (α,β,γ)(°)	90,90,90
Resolution (Å)	50.98-1.40 (1.42-1.40)
Rmerge	0.04 (0.46)
I/σ(I)	28.2 (4.3)
Half-set correlation CC(1/2)	100 (100)
Completeness	100.0 (100.0)
Redundancy	11.8 (12.5)
Refinement	
No. reflections all/free	55172 / 2735
R-factor/R-free	0.201 /0.221
Protein B-factor (No. atoms)	25.6 (2433)
Ligand B-factor (No. atoms)	64.6 (24)
Water B-factor (No. atoms)	35.7 (277)
RMS Deviations	
Bonds (Å)	0.0123
Angles (°)	1.815

6. References

1. M. Uguen, C. Gai, L. J. Sprenger, H. Liu, A. G. Leach and M. J. Waring, *RSC Adv.*, 2021, **11**, 30229–30236.
2. Sander, T.; Freyss, J.; Von Korff, M.; Rufener, C. DataWarrior: An Open-Source Program for Chemistry Aware Data Visualization and Analysis. J. Chem. Inf. Model. 2015, 55 (2), 460–473
3. Colomer, I.; Empson, C. J.; Craven, P.; Owen, Z.; Doveston, R. G.; Churcher, I.; Marsden, S. P.; Nelson, A. A Divergent Synthetic Approach to Diverse Molecular Scaffolds: Assessment of Lead-Likeness Using LLAMA, an Open-Access Computational Tool. Chem. Commun. 2016, 52 (45), 7209–7212
